# Supplementary material for: Fine-Mapping of Sorghum Stay-Green QTL on Chromosome10 Revealed Genes Associated with Delayed Senescence
Source: Genes (Basel). 2020 Sep 1;11(9):1026. doi: 10.3390/genes11091026 (PMC7565436; doi:10.3390/genes11091026)
Supplement: Supplementary file 1 [file genes-11-01026-s001.zip › STGFM_Supple Figures_17082020.pptx]

## Slide 1
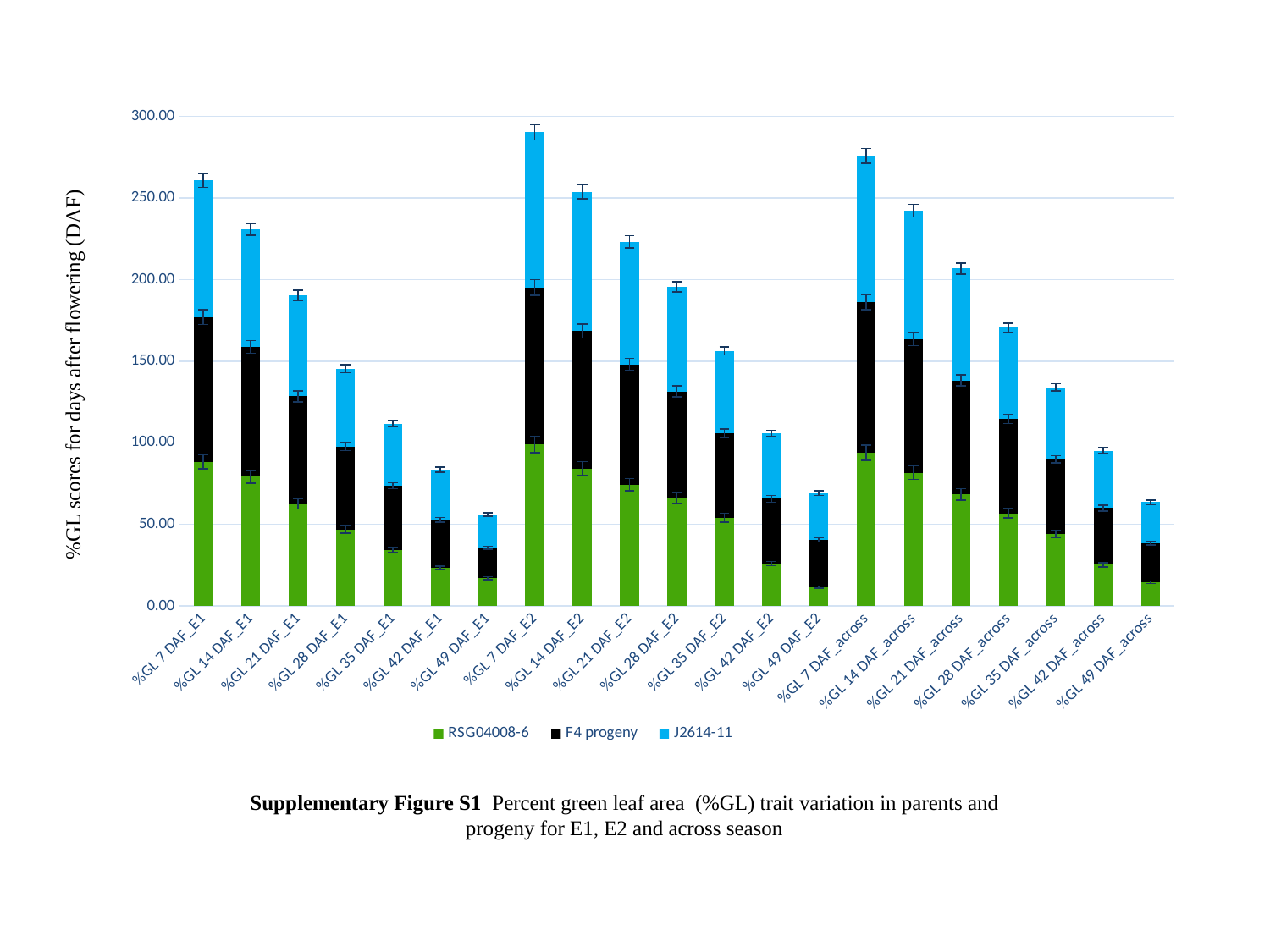

### Chart
| Category | RSG04008-6 | F4 progeny | J2614-11 |
|---|---|---|---|
| %GL 7 DAF_E1 | 88.38 | 88.67 | 83.68 |
| %GL 14 DAF_E1 | 79.18 | 79.58 | 72.06 |
| %GL 21 DAF_E1 | 62.52 | 65.96 | 61.88 |
| %GL 28 DAF_E1 | 47.0 | 50.72 | 47.68 |
| %GL 35 DAF_E1 | 34.46 | 39.49 | 37.72 |
| %GL 42 DAF_E1 | 23.44 | 29.41 | 30.73 |
| %GL 49 DAF_E1 | 17.12 | 18.56 | 20.33 |
| %GL 7 DAF_E2 | 99.03 | 96.05 | 95.24 |
| %GL 14 DAF_E2 | 84.22 | 84.29 | 85.26 |
| %GL 21 DAF_E2 | 74.39 | 73.73 | 75.05 |
| %GL 28 DAF_E2 | 66.49 | 65.03 | 64.04 |
| %GL 35 DAF_E2 | 54.17 | 51.57 | 50.54 |
| %GL 42 DAF_E2 | 25.96 | 39.73 | 40.1 |
| %GL 49 DAF_E2 | 11.68 | 29.07 | 28.4 |
| %GL 7 DAF_across | 93.86 | 92.36 | 89.56 |
| %GL 14 DAF_across | 81.71 | 81.94 | 78.65 |
| %GL 21 DAF_across | 68.43 | 69.85 | 68.48 |
| %GL 28 DAF_across | 56.83 | 57.88 | 55.78 |
| %GL 35 DAF_across | 44.39 | 45.53 | 44.19 |
| %GL 42 DAF_across | 25.28 | 34.71 | 35.25 |
| %GL 49 DAF_across | 14.67 | 24.01 | 24.94 |%GL scores for days after flowering (DAF)
Supplementary Figure S1 Percent green leaf area (%GL) trait variation in parents and progeny for E1, E2 and across season

## Slide 2
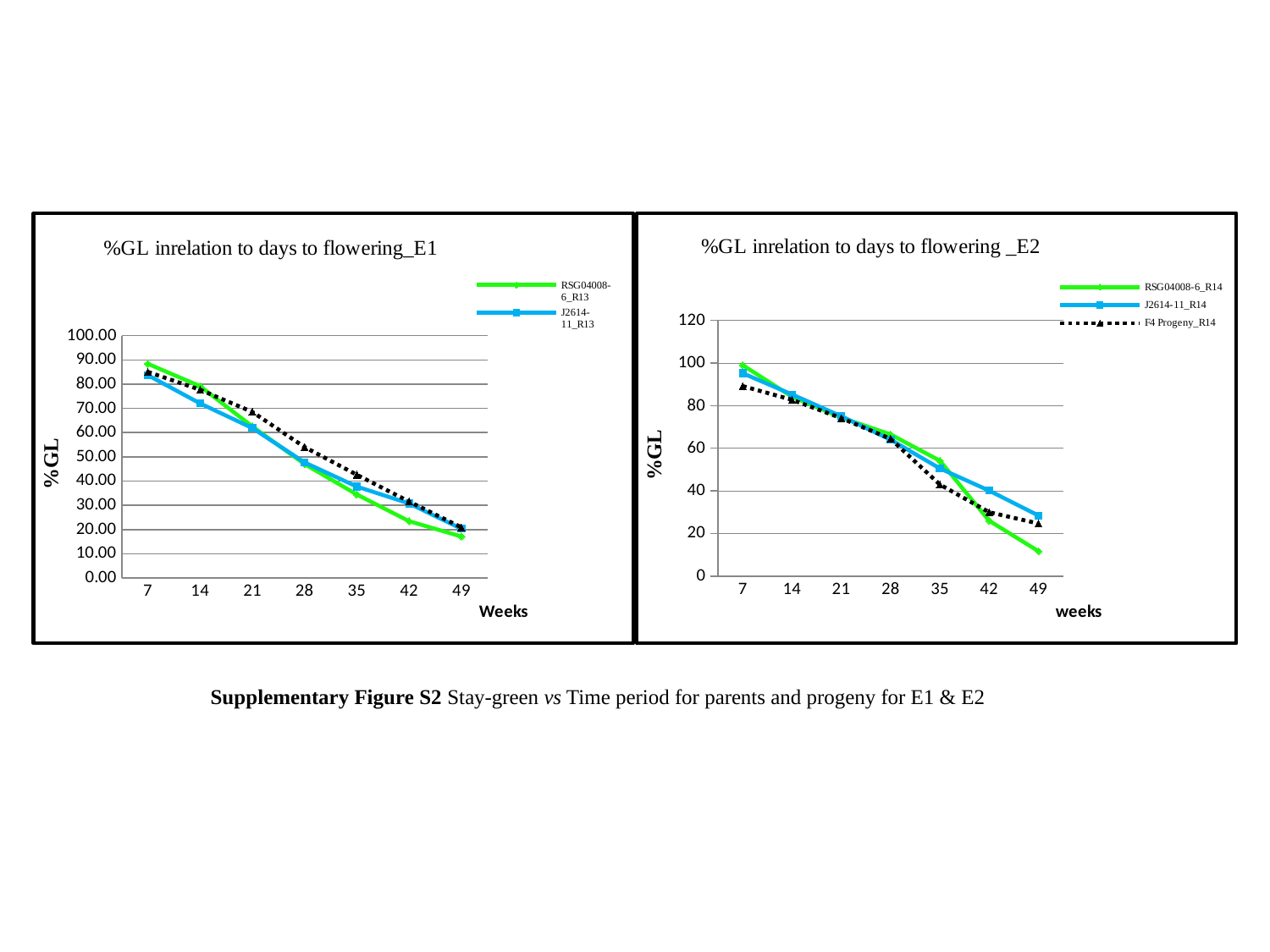

### Chart: %GL inrelation to days to flowering_E1
| Category | RSG04008-6_R13 | J2614-11_R13 | F4 Progeny_R13 |
|---|---|---|---|
| 7 | 88.38 | 83.67999999999998 | 85.05499999999999 |
| 14 | 79.17999999999998 | 72.06 | 77.68499999999999 |
| 21 | 62.52 | 61.879999999999995 | 68.53 |
| 28 | 47.0 | 47.68 | 54.015 |
| 35 | 34.46 | 37.72000000000001 | 42.575 |
| 42 | 23.439999999999987 | 30.73 | 31.535000000000004 |
| 49 | 17.12 | 20.329999999999988 | 20.9 |
### Chart: %GL inrelation to days to flowering _E2
| Category | RSG04008-6_R14 | J2614-11_R14 | F4 Progeny_R14 |
|---|---|---|---|
| 7 | 99.03 | 95.24000000000002 | 89.2 |
| 14 | 84.22 | 85.26 | 82.765 |
| 21 | 74.39 | 75.05 | 74.12499999999999 |
| 28 | 66.49 | 64.04 | 64.425 |
| 35 | 54.17 | 50.54 | 42.97 |
| 42 | 25.959999999999987 | 40.1 | 29.990000000000002 |
| 49 | 11.68 | 28.4 | 24.67 |Supplementary Figure S2 Stay-green vs Time period for parents and progeny for E1 & E2

## Slide 3
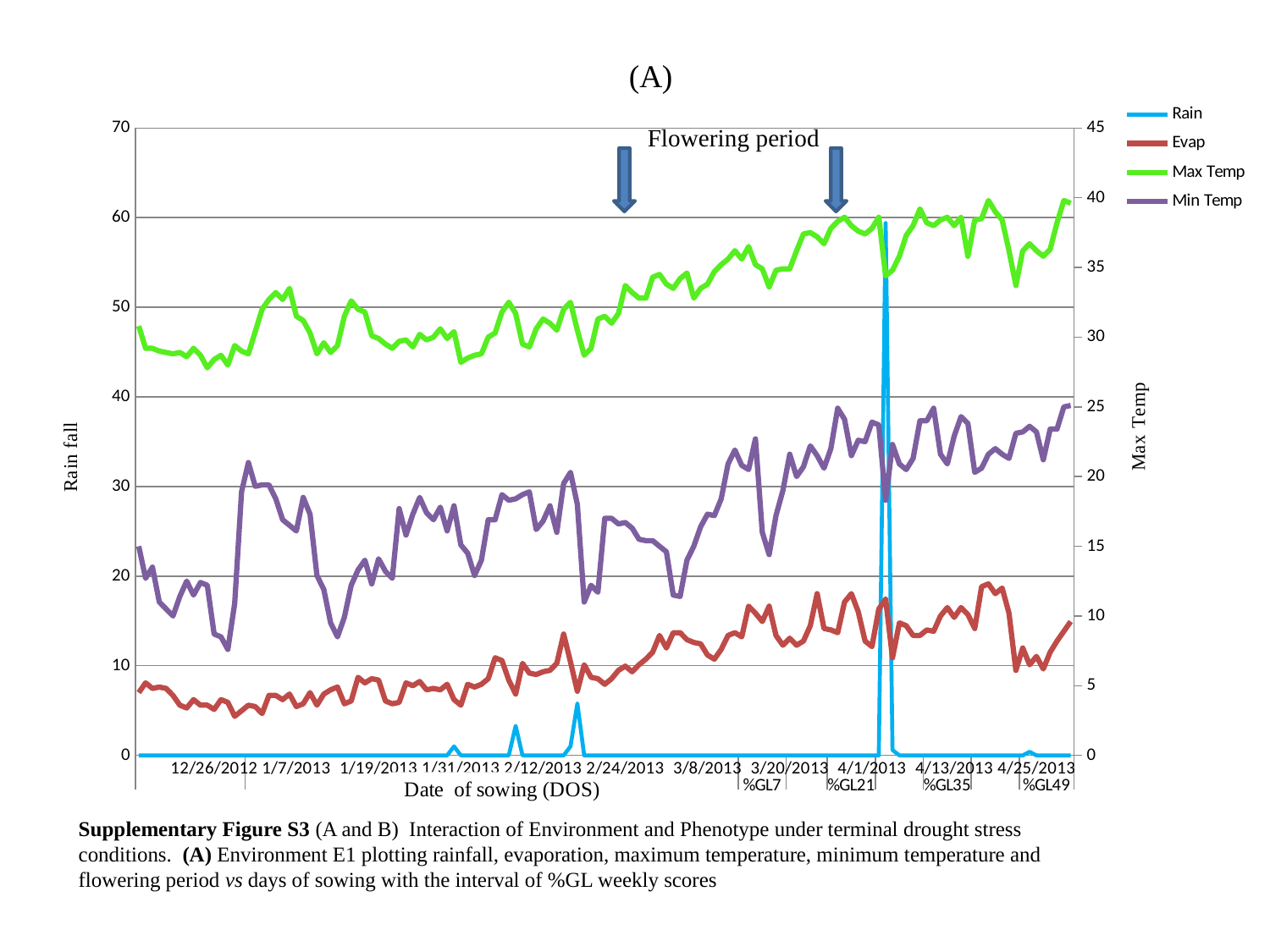

### Chart: (A)
| Category | Rain | Evap | Max Temp | Min Temp |
|---|---|---|---|---|
| 12/15/2012 | 0.0 | 4.5 | 30.8 | 15.0 |
| 12/16/2012 | 0.0 | 5.2 | 29.2 | 12.7 |
| 12/17/2012 | 0.0 | 4.8 | 29.2 | 13.5 |
| 12/18/2012 | 0.0 | 4.9 | 29.0 | 11.0 |
| 12/19/2012 | 0.0 | 4.8 | 28.9 | 10.5 |
| 12/20/2012 | 0.0 | 4.3 | 28.8 | 10.0 |
| 12/21/2012 | 0.0 | 3.6 | 28.9 | 11.4 |
| 12/22/2012 | 0.0 | 3.4 | 28.6 | 12.5 |
| 12/23/2012 | 0.0 | 4.0 | 29.2 | 11.5 |
| 12/24/2012 | 0.0 | 3.6 | 28.7 | 12.4 |
| 12/25/2012 | 0.0 | 3.6 | 27.8 | 12.2 |
| 12/26/2012 | 0.0 | 3.3 | 28.4 | 8.7 |
| 12/27/2012 | 0.0 | 4.0 | 28.7 | 8.5 |
| 12/28/2012 | 0.0 | 3.8 | 28.0 | 7.6 |
| 12/29/2012 | 0.0 | 2.8 | 29.4 | 10.9 |
| 12/30/2012 | 0.0 | 3.2 | 29.0 | 18.9 |
| 12/31/2012 | 0.0 | 3.6 | 28.8 | 21.0 |
| 1/1/2013 | 0.0 | 3.5 | 30.4 | 19.3 |
| 1/2/2013 | 0.0 | 3.0 | 32.0 | 19.4 |
| 1/3/2013 | 0.0 | 4.3 | 32.7 | 19.4 |
| 1/4/2013 | 0.0 | 4.3 | 33.2 | 18.4 |
| 1/5/2013 | 0.0 | 4.0 | 32.7 | 16.9 |
| 1/6/2013 | 0.0 | 4.4 | 33.5 | 16.5 |
| 1/7/2013 | 0.0 | 3.5 | 31.5 | 16.1 |
| 1/8/2013 | 0.0 | 3.7 | 31.2 | 18.5 |
| 1/9/2013 | 0.0 | 4.5 | 30.3 | 17.3 |
| 1/10/2013 | 0.0 | 3.6 | 28.8 | 12.9 |
| 1/11/2013 | 0.0 | 4.4 | 29.6 | 11.9 |
| 1/12/2013 | 0.0 | 4.7 | 28.9 | 9.5 |
| 1/13/2013 | 0.0 | 4.9 | 29.4 | 8.5 |
| 1/14/2013 | 0.0 | 3.7 | 31.5 | 9.9 |
| 1/15/2013 | 0.0 | 3.9 | 32.6 | 12.2 |
| 1/16/2013 | 0.0 | 5.6 | 32.0 | 13.3 |
| 1/17/2013 | 0.0 | 5.2 | 31.8 | 14.0 |
| 1/18/2013 | 0.0 | 5.5 | 30.1 | 12.3 |
| 1/19/2013 | 0.0 | 5.4 | 29.9 | 14.1 |
| 1/20/2013 | 0.0 | 3.9 | 29.5 | 13.2 |
| 1/21/2013 | 0.0 | 3.7 | 29.2 | 12.7 |
| 1/22/2013 | 0.0 | 3.8 | 29.7 | 17.7 |
| 1/23/2013 | 0.0 | 5.2 | 29.8 | 15.8 |
| 1/24/2013 | 0.0 | 5.0 | 29.3 | 17.3 |
| 1/25/2013 | 0.0 | 5.3 | 30.2 | 18.5 |
| 1/26/2013 | 0.0 | 4.7 | 29.8 | 17.4 |
| 1/27/2013 | 0.0 | 4.8 | 30.0 | 16.9 |
| 1/28/2013 | 0.0 | 4.7 | 30.6 | 17.8 |
| 1/29/2013 | 0.0 | 5.1 | 29.9 | 16.1 |
| 1/30/2013 | 1.0 | 4.0 | 30.4 | 17.9 |
| 1/31/2013 | 0.0 | 3.6 | 28.2 | 15.1 |
| 2/1/2013 | 0.0 | 5.1 | 28.5 | 14.5 |
| 2/2/2013 | 0.0 | 4.9 | 28.7 | 12.9 |
| 2/3/2013 | 0.0 | 5.1 | 28.8 | 14.0 |
| 2/4/2013 | 0.0 | 5.5 | 30.0 | 16.9 |
| 2/5/2013 | 0.0 | 7.0 | 30.3 | 16.9 |
| 2/6/2013 | 0.0 | 6.8 | 31.8 | 18.7 |
| 2/7/2013 | 0.0 | 5.4 | 32.5 | 18.3 |
| 2/8/2013 | 3.3 | 4.4 | 31.7 | 18.4 |
| 2/9/2013 | 0.0 | 6.6 | 29.5 | 18.7 |
| 2/10/2013 | 0.0 | 5.9 | 29.3 | 18.9 |
| 2/11/2013 | 0.0 | 5.8 | 30.6 | 16.2 |
| 2/12/2013 | 0.0 | 6.0 | 31.3 | 16.8 |
| 2/13/2013 | 0.0 | 6.1 | 31.0 | 17.9 |
| 2/14/2013 | 0.0 | 6.6 | 30.5 | 16.0 |
| 2/15/2013 | 0.0 | 8.7 | 32.0 | 19.5 |
| 2/16/2013 | 1.0 | 6.7 | 32.5 | 20.3 |
| 2/17/2013 | 5.8 | 4.6 | 30.5 | 18.0 |
| 2/18/2013 | 0.0 | 6.5 | 28.7 | 11.0 |
| 2/19/2013 | 0.0 | 5.6 | 29.2 | 12.2 |
| 2/20/2013 | 0.0 | 5.5 | 31.3 | 11.7 |
| 2/21/2013 | 0.0 | 5.1 | 31.5 | 17.0 |
| 2/22/2013 | 0.0 | 5.5 | 31.0 | 17.0 |
| 2/23/2013 | 0.0 | 6.1 | 31.7 | 16.6 |
| 2/24/2013 | 0.0 | 6.4 | 33.7 | 16.7 |
| 2/25/2013 | 0.0 | 6.0 | 33.2 | 16.3 |
| 2/26/2013 | 0.0 | 6.5 | 32.8 | 15.5 |
| 2/27/2013 | 0.0 | 6.9 | 32.8 | 15.4 |
| 2/28/2013 | 0.0 | 7.4 | 34.3 | 15.4 |
| 3/1/2013 | 0.0 | 8.6 | 34.5 | 15.0 |
| 3/2/2013 | 0.0 | 7.7 | 33.8 | 14.6 |
| 3/3/2013 | 0.0 | 8.8 | 33.5 | 11.5 |
| 3/4/2013 | 0.0 | 8.8 | 34.2 | 11.4 |
| 3/5/2013 | 0.0 | 8.3 | 34.6 | 14.0 |
| 3/6/2013 | 0.0 | 8.1 | 32.8 | 15.0 |
| 3/7/2013 | 0.0 | 8.0 | 33.5 | 16.4 |
| 3/8/2013 | 0.0 | 7.2 | 33.8 | 17.3 |
| 3/9/2013 | 0.0 | 6.9 | 34.7 | 17.2 |
| 3/10/2013 | 0.0 | 7.6 | 35.2 | 18.4 |
| 3/11/2013 | 0.0 | 8.6 | 35.6 | 20.9 |
| 3/12/2013 | 0.0 | 8.8 | 36.2 | 21.9 |
| 3/13/2013 | 0.0 | 8.5 | 35.6 | 20.8 |
| 3/14/2013 | 0.0 | 10.7 | 36.5 | 20.5 |
| 3/15/2013 | 0.0 | 10.2 | 35.2 | 22.7 |
| 3/16/2013 | 0.0 | 9.6 | 34.9 | 16.0 |
| 3/17/2013 | 0.0 | 10.7 | 33.6 | 14.4 |
| 3/18/2013 | 0.0 | 8.6 | 34.8 | 17.2 |
| 3/19/2013 | 0.0 | 7.9 | 34.9 | 19.0 |
| 3/20/2013 | 0.0 | 8.4 | 34.9 | 21.6 |
| 3/21/2013 | 0.0 | 7.9 | 36.2 | 20.0 |
| 3/22/2013 | 0.0 | 8.2 | 37.4 | 20.7 |
| 3/23/2013 | 0.0 | 9.3 | 37.5 | 22.2 |
| 3/24/2013 | 0.0 | 11.6 | 37.2 | 21.5 |
| 3/25/2013 | 0.0 | 9.1 | 36.7 | 20.6 |
| 3/26/2013 | 0.0 | 9.0 | 37.8 | 22.0 |
| 3/27/2013 | 0.0 | 8.8 | 38.3 | 24.9 |
| 3/28/2013 | 0.0 | 11.0 | 38.6 | 24.1 |
| 3/29/2013 | 0.0 | 11.6 | 38.0 | 21.5 |
| 3/30/2013 | 0.0 | 10.3 | 37.6 | 22.6 |
| 3/31/2013 | 0.0 | 8.2 | 37.4 | 22.5 |
| 4/1/2013 | 0.0 | 7.8 | 37.8 | 23.9 |
| 4/2/2013 | 0.0 | 10.5 | 38.6 | 23.7 |
| 4/3/2013 | 59.4 | 11.2 | 34.4 | 18.3 |
| 4/4/2013 | 0.6 | 7.0 | 34.8 | 22.3 |
| 4/5/2013 | 0.0 | 9.5 | 35.8 | 20.9 |
| 4/6/2013 | 0.0 | 9.3 | 37.3 | 20.5 |
| 4/7/2013 | 0.0 | 8.6 | 38.0 | 21.3 |
| 4/8/2013 | 0.0 | 8.6 | 39.2 | 24.0 |
| 4/9/2013 | 0.0 | 9.0 | 38.2 | 24.0 |
| 4/10/2013 | 0.0 | 8.9 | 38.0 | 24.9 |
| 4/11/2013 | 0.0 | 10.0 | 38.4 | 21.6 |
| 4/12/2013 | 0.0 | 10.6 | 38.6 | 20.9 |
| 4/13/2013 | 0.0 | 9.9 | 38.0 | 22.9 |
| 4/14/2013 | 0.0 | 10.6 | 38.6 | 24.3 |
| 4/15/2013 | 0.0 | 10.1 | 35.8 | 23.8 |
| 4/16/2013 | 0.0 | 9.1 | 38.4 | 20.3 |
| 4/17/2013 | 0.0 | 12.1 | 38.5 | 20.6 |
| 4/18/2013 | 0.0 | 12.3 | 39.8 | 21.6 |
| 4/19/2013 | 0.0 | 11.6 | 39.0 | 22.0 |
| 4/20/2013 | 0.0 | 12.0 | 38.4 | 21.6 |
| 4/21/2013 | 0.0 | 10.2 | 36.2 | 21.3 |
| 4/22/2013 | 0.0 | 6.1 | 33.7 | 23.1 |
| 4/23/2013 | 0.0 | 7.7 | 36.2 | 23.2 |
| 4/24/2013 | 0.4 | 6.5 | 36.7 | 23.6 |
| 4/25/2013 | 0.0 | 7.1 | 36.2 | 23.2 |
| 4/26/2013 | 0.0 | 6.2 | 35.8 | 21.2 |
| 4/27/2013 | 0.0 | 7.4 | 36.3 | 23.4 |
| 4/28/2013 | 0.0 | 8.2 | 38.2 | 23.4 |
| 4/29/2013 | 0.0 | 8.9 | 39.8 | 25.0 |
| 4/30/2013 | 0.0 | 9.6 | 39.6 | 25.1 |Flowering period
Supplementary Figure S3 (A and B) Interaction of Environment and Phenotype under terminal drought stress conditions. (A) Environment E1 plotting rainfall, evaporation, maximum temperature, minimum temperature and flowering period vs days of sowing with the interval of %GL weekly scores

## Slide 4
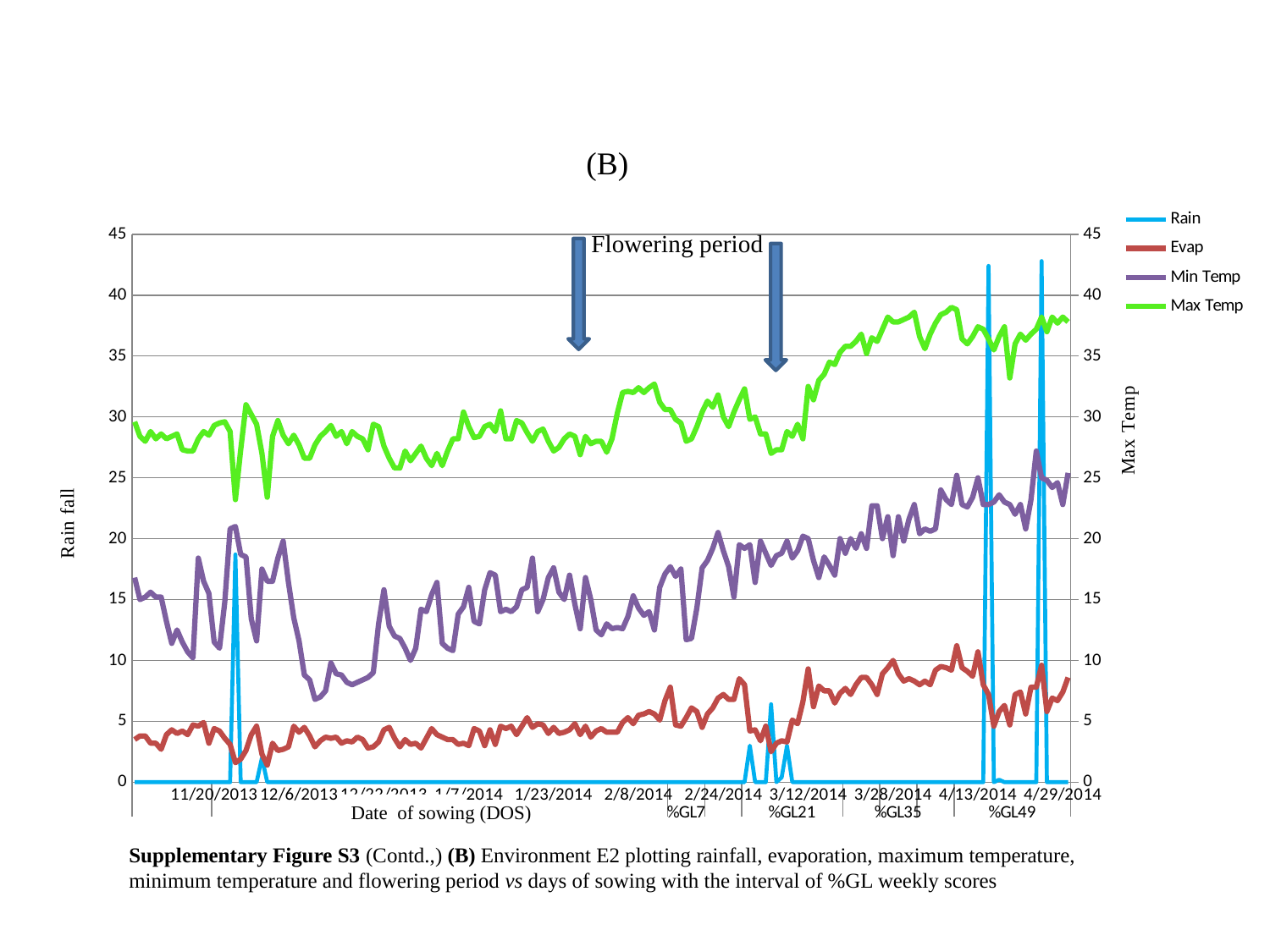

### Chart: (B)
| Category | Rain | Evap | Min Temp | Max Temp |
|---|---|---|---|---|
| 11/5/2013 | 0.0 | 3.5 | 16.8 | 29.6 |
| 11/6/2013 | 0.0 | 3.8 | 15.0 | 28.4 |
| 11/7/2013 | 0.0 | 3.8 | 15.2 | 28.0 |
| 11/8/2013 | 0.0 | 3.2 | 15.6 | 28.8 |
| 11/9/2013 | 0.0 | 3.2 | 15.2 | 28.2 |
| 11/10/2013 | 0.0 | 2.7 | 15.2 | 28.6 |
| 11/11/2013 | 0.0 | 3.9 | 13.2 | 28.2 |
| 11/12/2013 | 0.0 | 4.3 | 11.4 | 28.4 |
| 11/13/2013 | 0.0 | 4.0 | 12.5 | 28.6 |
| 11/14/2013 | 0.0 | 4.2 | 11.5 | 27.3 |
| 11/15/2013 | 0.0 | 3.9 | 10.7 | 27.2 |
| 11/16/2013 | 0.0 | 4.7 | 10.2 | 27.2 |
| 11/17/2013 | 0.0 | 4.6 | 18.4 | 28.2 |
| 11/18/2013 | 0.0 | 4.9 | 16.5 | 28.8 |
| 11/19/2013 | 0.0 | 3.2 | 15.5 | 28.5 |
| 11/20/2013 | 0.0 | 4.4 | 11.5 | 29.3 |
| 11/21/2013 | 0.0 | 4.2 | 11.0 | 29.5 |
| 11/22/2013 | 0.0 | 3.6 | 14.8 | 29.6 |
| 11/23/2013 | 0.0 | 3.1 | 20.8 | 28.8 |
| 11/24/2013 | 18.7 | 1.6 | 21.0 | 23.2 |
| 11/25/2013 | 0.0 | 1.9 | 18.7 | 27.3 |
| 11/26/2013 | 0.0 | 2.6 | 18.5 | 31.0 |
| 11/27/2013 | 0.0 | 3.9 | 13.4 | 30.2 |
| 11/28/2013 | 0.0 | 4.6 | 11.6 | 29.4 |
| 11/29/2013 | 2.0 | 2.3 | 17.5 | 27.0 |
| 11/30/2013 | 0.0 | 1.4 | 16.5 | 23.4 |
| 12/1/2013 | 0.0 | 3.2 | 16.5 | 28.4 |
| 12/2/2013 | 0.0 | 2.6 | 18.4 | 29.7 |
| 12/3/2013 | 0.0 | 2.7 | 19.8 | 28.5 |
| 12/4/2013 | 0.0 | 2.9 | 16.4 | 27.8 |
| 12/5/2013 | 0.0 | 4.6 | 13.5 | 28.5 |
| 12/6/2013 | 0.0 | 4.1 | 11.6 | 27.7 |
| 12/7/2013 | 0.0 | 4.5 | 8.8 | 26.6 |
| 12/8/2013 | 0.0 | 3.8 | 8.4 | 26.6 |
| 12/9/2013 | 0.0 | 2.9 | 6.8 | 27.7 |
| 12/10/2013 | 0.0 | 3.4 | 7.0 | 28.4 |
| 12/11/2013 | 0.0 | 3.7 | 7.5 | 28.8 |
| 12/12/2013 | 0.0 | 3.6 | 9.8 | 29.3 |
| 12/13/2013 | 0.0 | 3.7 | 8.9 | 28.4 |
| 12/14/2013 | 0.0 | 3.2 | 8.8 | 28.8 |
| 12/15/2013 | 0.0 | 3.4 | 8.2 | 27.8 |
| 12/16/2013 | 0.0 | 3.3 | 8.0 | 28.8 |
| 12/17/2013 | 0.0 | 3.7 | 8.2 | 28.4 |
| 12/18/2013 | 0.0 | 3.5 | 8.4 | 28.2 |
| 12/19/2013 | 0.0 | 2.8 | 8.6 | 27.3 |
| 12/20/2013 | 0.0 | 2.9 | 9.0 | 29.4 |
| 12/21/2013 | 0.0 | 3.3 | 13.0 | 29.2 |
| 12/22/2013 | 0.0 | 4.3 | 15.8 | 27.6 |
| 12/23/2013 | 0.0 | 4.5 | 12.8 | 26.6 |
| 12/24/2013 | 0.0 | 3.6 | 12.0 | 25.8 |
| 12/25/2013 | 0.0 | 2.9 | 11.8 | 25.8 |
| 12/26/2013 | 0.0 | 3.5 | 11.0 | 27.2 |
| 12/27/2013 | 0.0 | 3.1 | 10.0 | 26.4 |
| 12/28/2013 | 0.0 | 3.2 | 11.0 | 27.0 |
| 12/29/2013 | 0.0 | 2.8 | 14.2 | 27.6 |
| 12/30/2013 | 0.0 | 3.6 | 14.0 | 26.6 |
| 12/31/2013 | 0.0 | 4.4 | 15.4 | 26.0 |
| 1/1/2014 | 0.0 | 3.9 | 16.4 | 27.0 |
| 1/2/2014 | 0.0 | 3.7 | 11.4 | 26.0 |
| 1/3/2014 | 0.0 | 3.5 | 11.0 | 27.2 |
| 1/4/2014 | 0.0 | 3.5 | 10.8 | 28.2 |
| 1/5/2014 | 0.0 | 3.1 | 13.8 | 28.2 |
| 1/6/2014 | 0.0 | 3.2 | 14.4 | 30.4 |
| 1/7/2014 | 0.0 | 3.0 | 16.0 | 29.2 |
| 1/8/2014 | 0.0 | 4.4 | 13.2 | 28.3 |
| 1/9/2014 | 0.0 | 4.2 | 13.0 | 28.4 |
| 1/10/2014 | 0.0 | 3.0 | 15.8 | 29.2 |
| 1/11/2014 | 0.0 | 4.3 | 17.2 | 29.4 |
| 1/12/2014 | 0.0 | 3.1 | 17.0 | 28.8 |
| 1/13/2014 | 0.0 | 4.6 | 14.0 | 30.5 |
| 1/14/2014 | 0.0 | 4.4 | 14.2 | 28.2 |
| 1/15/2014 | 0.0 | 4.6 | 14.0 | 28.2 |
| 1/16/2014 | 0.0 | 3.9 | 14.4 | 29.7 |
| 1/17/2014 | 0.0 | 4.6 | 15.8 | 29.5 |
| 1/18/2014 | 0.0 | 5.3 | 16.0 | 28.7 |
| 1/19/2014 | 0.0 | 4.5 | 18.4 | 28.0 |
| 1/20/2014 | 0.0 | 4.8 | 14.0 | 28.8 |
| 1/21/2014 | 0.0 | 4.7 | 15.0 | 29.0 |
| 1/22/2014 | 0.0 | 4.0 | 16.8 | 28.0 |
| 1/23/2014 | 0.0 | 4.5 | 17.6 | 27.2 |
| 1/24/2014 | 0.0 | 4.0 | 15.6 | 27.5 |
| 1/25/2014 | 0.0 | 4.1 | 15.0 | 28.2 |
| 1/26/2014 | 0.0 | 4.3 | 17.0 | 28.6 |
| 1/27/2014 | 0.0 | 4.8 | 14.6 | 28.4 |
| 1/28/2014 | 0.0 | 3.9 | 12.6 | 26.9 |
| 1/29/2014 | 0.0 | 4.6 | 16.8 | 28.4 |
| 1/30/2014 | 0.0 | 3.7 | 15.0 | 27.8 |
| 1/31/2014 | 0.0 | 4.2 | 12.5 | 28.0 |
| 2/1/2014 | 0.0 | 4.4 | 12.1 | 28.0 |
| 2/2/2014 | 0.0 | 4.1 | 13.0 | 27.1 |
| 2/3/2014 | 0.0 | 4.1 | 12.6 | 28.2 |
| 2/4/2014 | 0.0 | 4.1 | 12.7 | 30.3 |
| 2/5/2014 | 0.0 | 4.9 | 12.6 | 32.0 |
| 2/6/2014 | 0.0 | 5.3 | 13.6 | 32.1 |
| 2/7/2014 | 0.0 | 4.8 | 15.3 | 32.0 |
| 2/8/2014 | 0.0 | 5.5 | 14.3 | 32.4 |
| 2/9/2014 | 0.0 | 5.6 | 13.7 | 32.0 |
| 2/10/2014 | 0.0 | 5.8 | 14.0 | 32.4 |
| 2/11/2014 | 0.0 | 5.6 | 12.5 | 32.7 |
| 2/12/2014 | 0.0 | 5.1 | 16.0 | 31.2 |
| 2/13/2014 | 0.0 | 6.7 | 17.1 | 30.6 |
| 2/14/2014 | 0.0 | 7.8 | 17.7 | 30.6 |
| 2/15/2014 | 0.0 | 4.7 | 16.9 | 29.8 |
| 2/16/2014 | 0.0 | 4.6 | 17.5 | 29.5 |
| 2/17/2014 | 0.0 | 5.3 | 11.7 | 28.0 |
| 2/18/2014 | 0.0 | 6.1 | 11.8 | 28.2 |
| 2/19/2014 | 0.0 | 5.8 | 14.3 | 29.2 |
| 2/20/2014 | 0.0 | 4.5 | 17.6 | 30.4 |
| 2/21/2014 | 0.0 | 5.6 | 18.2 | 31.3 |
| 2/22/2014 | 0.0 | 6.1 | 19.2 | 30.8 |
| 2/23/2014 | 0.0 | 6.9 | 20.5 | 31.8 |
| 2/24/2014 | 0.0 | 7.2 | 19.0 | 30.0 |
| 2/25/2014 | 0.0 | 6.8 | 17.7 | 29.2 |
| 2/26/2014 | 0.0 | 6.8 | 15.2 | 30.4 |
| 2/27/2014 | 0.0 | 8.5 | 19.5 | 31.4 |
| 2/28/2014 | 0.0 | 8.0 | 19.2 | 32.3 |
| 3/1/2014 | 3.0 | 4.2 | 19.5 | 29.8 |
| 3/2/2014 | 0.0 | 4.3 | 16.4 | 30.0 |
| 3/3/2014 | 0.0 | 3.4 | 19.8 | 28.6 |
| 3/4/2014 | 0.0 | 4.6 | 18.8 | 28.6 |
| 3/5/2014 | 6.4 | 2.5 | 17.8 | 27.0 |
| 3/6/2014 | 0.0 | 3.2 | 18.6 | 27.3 |
| 3/7/2014 | 0.4 | 3.4 | 18.8 | 27.3 |
| 3/8/2014 | 3.0 | 3.3 | 19.8 | 28.8 |
| 3/9/2014 | 0.0 | 5.1 | 18.4 | 28.4 |
| 3/10/2014 | 0.0 | 4.8 | 19.0 | 29.4 |
| 3/11/2014 | 0.0 | 6.6 | 20.2 | 28.2 |
| 3/12/2014 | 0.0 | 9.3 | 20.0 | 32.5 |
| 3/13/2014 | 0.0 | 6.2 | 18.2 | 31.4 |
| 3/14/2014 | 0.0 | 7.9 | 16.8 | 33.0 |
| 3/15/2014 | 0.0 | 7.5 | 18.5 | 33.5 |
| 3/16/2014 | 0.0 | 7.5 | 17.8 | 34.5 |
| 3/17/2014 | 0.0 | 6.5 | 17.0 | 34.3 |
| 3/18/2014 | 0.0 | 7.3 | 20.0 | 35.3 |
| 3/19/2014 | 0.0 | 7.7 | 18.8 | 35.8 |
| 3/20/2014 | 0.0 | 7.2 | 20.0 | 35.8 |
| 3/21/2014 | 0.0 | 8.0 | 19.2 | 36.2 |
| 3/22/2014 | 0.0 | 8.6 | 20.4 | 36.8 |
| 3/23/2014 | 0.0 | 8.6 | 19.2 | 35.2 |
| 3/24/2014 | 0.0 | 8.0 | 22.7 | 36.5 |
| 3/25/2014 | 0.0 | 7.2 | 22.7 | 36.2 |
| 3/26/2014 | 0.0 | 8.9 | 20.0 | 37.2 |
| 3/27/2014 | 0.0 | 9.4 | 21.8 | 38.2 |
| 3/28/2014 | 0.0 | 10.0 | 18.6 | 37.8 |
| 3/29/2014 | 0.0 | 8.9 | 21.8 | 37.8 |
| 3/30/2014 | 0.0 | 8.3 | 19.8 | 38.0 |
| 3/31/2014 | 0.0 | 8.5 | 21.6 | 38.2 |
| 4/1/2014 | 0.0 | 8.3 | 22.8 | 38.6 |
| 4/2/2014 | 0.0 | 8.0 | 20.4 | 36.6 |
| 4/3/2014 | 0.0 | 8.3 | 20.8 | 35.6 |
| 4/4/2014 | 0.0 | 8.0 | 20.6 | 36.8 |
| 4/5/2014 | 0.0 | 9.2 | 20.8 | 37.7 |
| 4/6/2014 | 0.0 | 9.5 | 24.0 | 38.4 |
| 4/7/2014 | 0.0 | 9.4 | 23.2 | 38.6 |
| 4/8/2014 | 0.0 | 9.2 | 22.8 | 39.0 |
| 4/9/2014 | 0.0 | 11.2 | 25.2 | 38.8 |
| 4/10/2014 | 0.0 | 9.4 | 22.8 | 36.4 |
| 4/11/2014 | 0.0 | 9.1 | 22.6 | 36.0 |
| 4/12/2014 | 0.0 | 8.7 | 23.4 | 36.6 |
| 4/13/2014 | 0.0 | 10.7 | 25.0 | 37.4 |
| 4/14/2014 | 0.0 | 8.0 | 22.8 | 37.2 |
| 4/15/2014 | 42.4 | 7.2 | 22.8 | 36.4 |
| 4/16/2014 | 0.0 | 4.6 | 23.0 | 35.5 |
| 4/17/2014 | 0.2 | 5.8 | 23.6 | 36.6 |
| 4/18/2014 | 0.0 | 6.3 | 23.0 | 37.4 |
| 4/19/2014 | 0.0 | 4.7 | 22.8 | 33.2 |
| 4/20/2014 | 0.0 | 7.2 | 22.0 | 36.0 |
| 4/21/2014 | 0.0 | 7.4 | 22.8 | 36.8 |
| 4/22/2014 | 0.0 | 5.6 | 20.8 | 36.3 |
| 4/23/2014 | 0.0 | 7.8 | 23.2 | 36.8 |
| 4/24/2014 | 0.0 | 7.8 | 27.2 | 37.2 |
| 4/25/2014 | 42.8 | 9.6 | 25.0 | 38.2 |
| 4/26/2014 | 0.0 | 5.8 | 24.8 | 37.0 |
| 4/27/2014 | 0.0 | 6.9 | 24.2 | 38.2 |
| 4/28/2014 | 0.0 | 6.7 | 24.6 | 37.7 |
| 4/29/2014 | 0.0 | 7.4 | 22.8 | 38.2 |
| 4/30/2014 | 0.0 | 8.6 | 25.4 | 37.8 |Flowering period
Date of sowing (DOS)
Supplementary Figure S3 (Contd.,) (B) Environment E2 plotting rainfall, evaporation, maximum temperature, minimum temperature and flowering period vs days of sowing with the interval of %GL weekly scores

## Slide 5
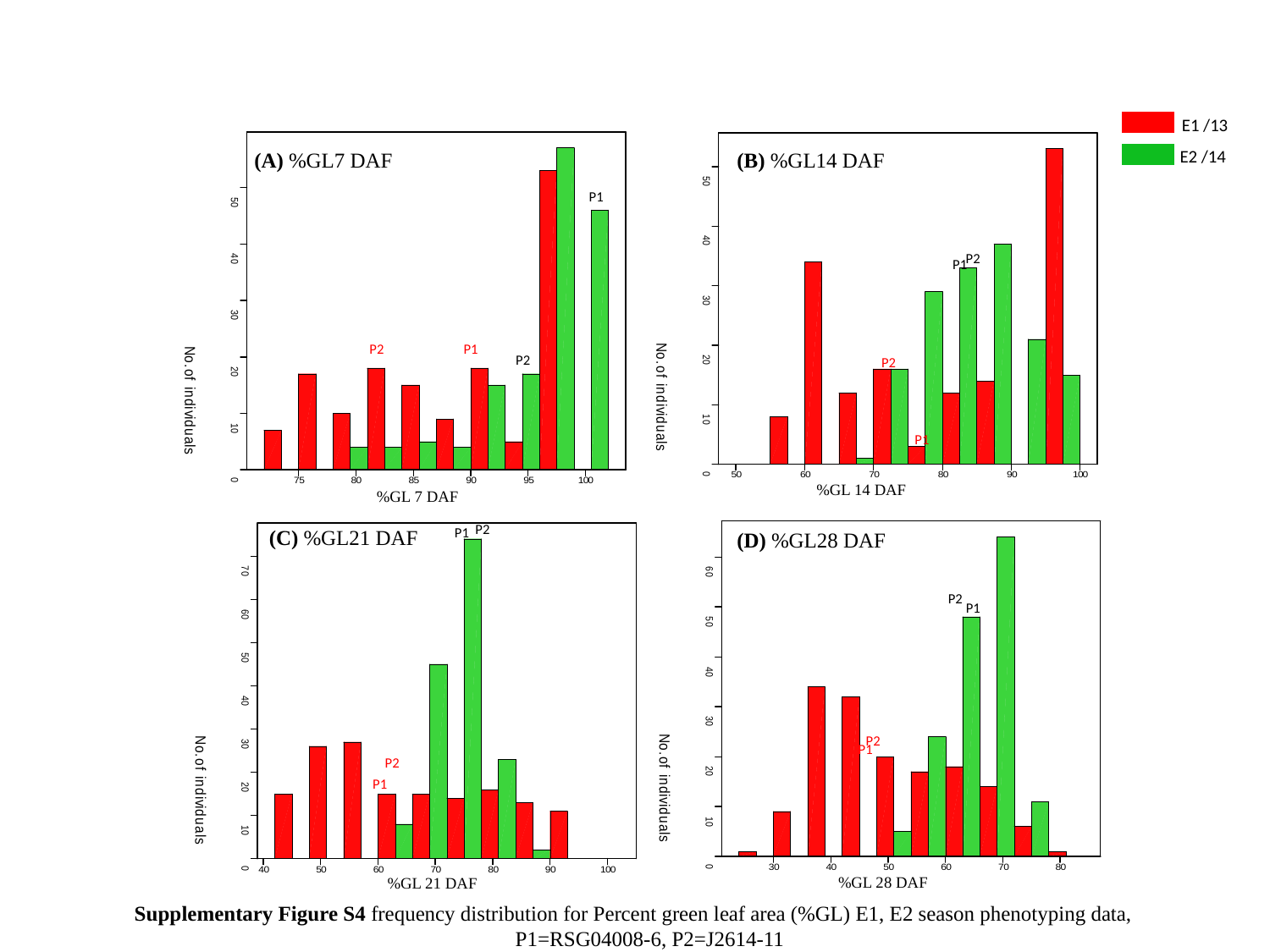

E1 /13
E2 /14
P1
P2
P1
P2
P2
P1
P2
P1
P2
P1
P2
P1
P2
P1
P2
P1
(A) %GL7 DAF
(B) %GL14 DAF
%GL 14 DAF
%GL 7 DAF
(C) %GL21 DAF
(D) %GL28 DAF
 %GL 28 DAF
%GL 21 DAF
Supplementary Figure S4 frequency distribution for Percent green leaf area (%GL) E1, E2 season phenotyping data,
 			P1=RSG04008-6, P2=J2614-11

## Slide 6
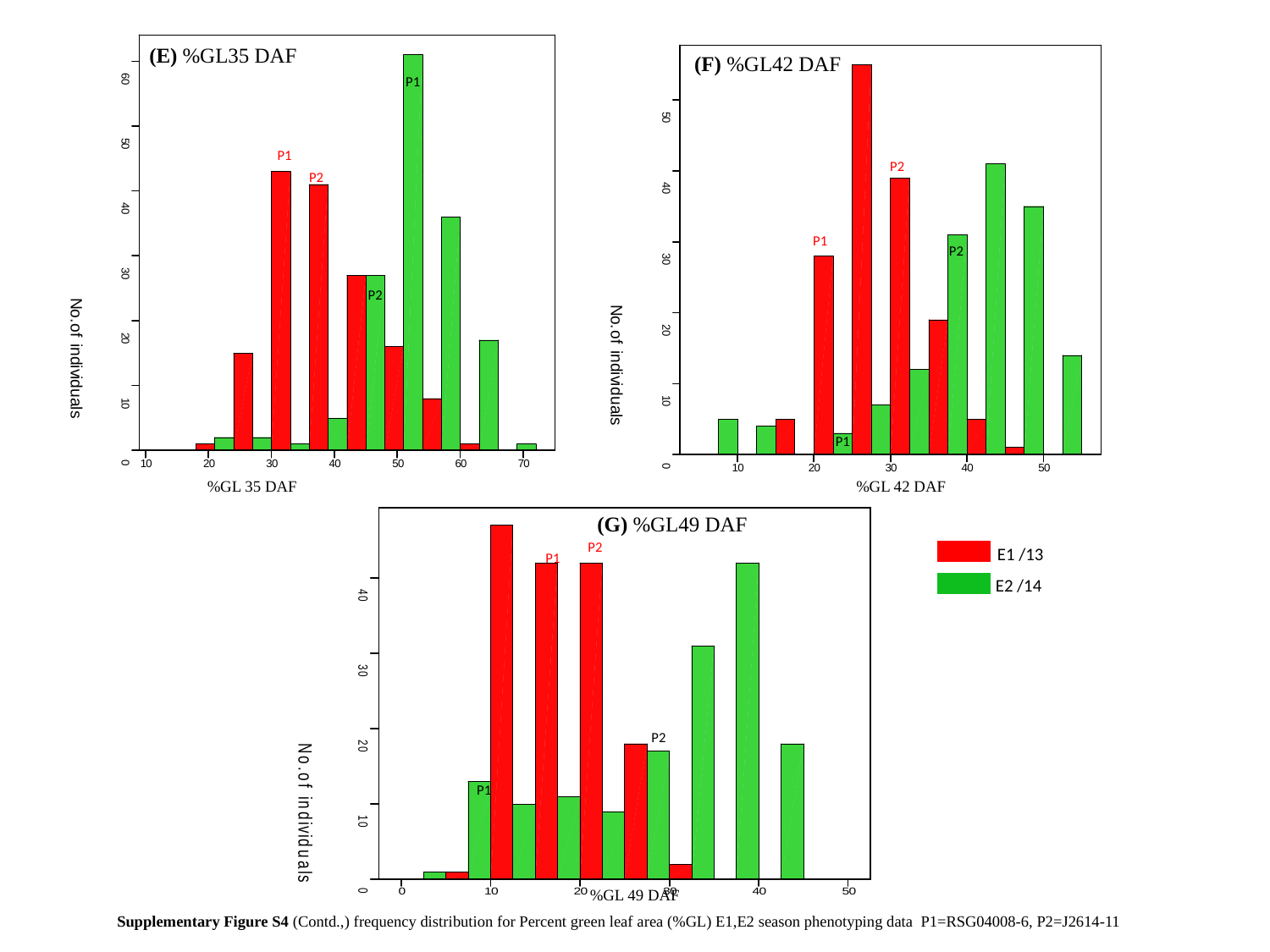

P1
P1
P2
P2
P2
P1
P2
P1
P2
P1
P2
P1
(E) %GL35 DAF
(F) %GL42 DAF
(G) %GL49 DAF
%GL 35 DAF
%GL 42 DAF
E1 /13
E2 /14
%GL 49 DAF
# Supplementary Figure S4 (Contd.,) frequency distribution for Percent green leaf area (%GL) E1,E2 season phenotyping data P1=RSG04008-6, P2=J2614-11

## Slide 7
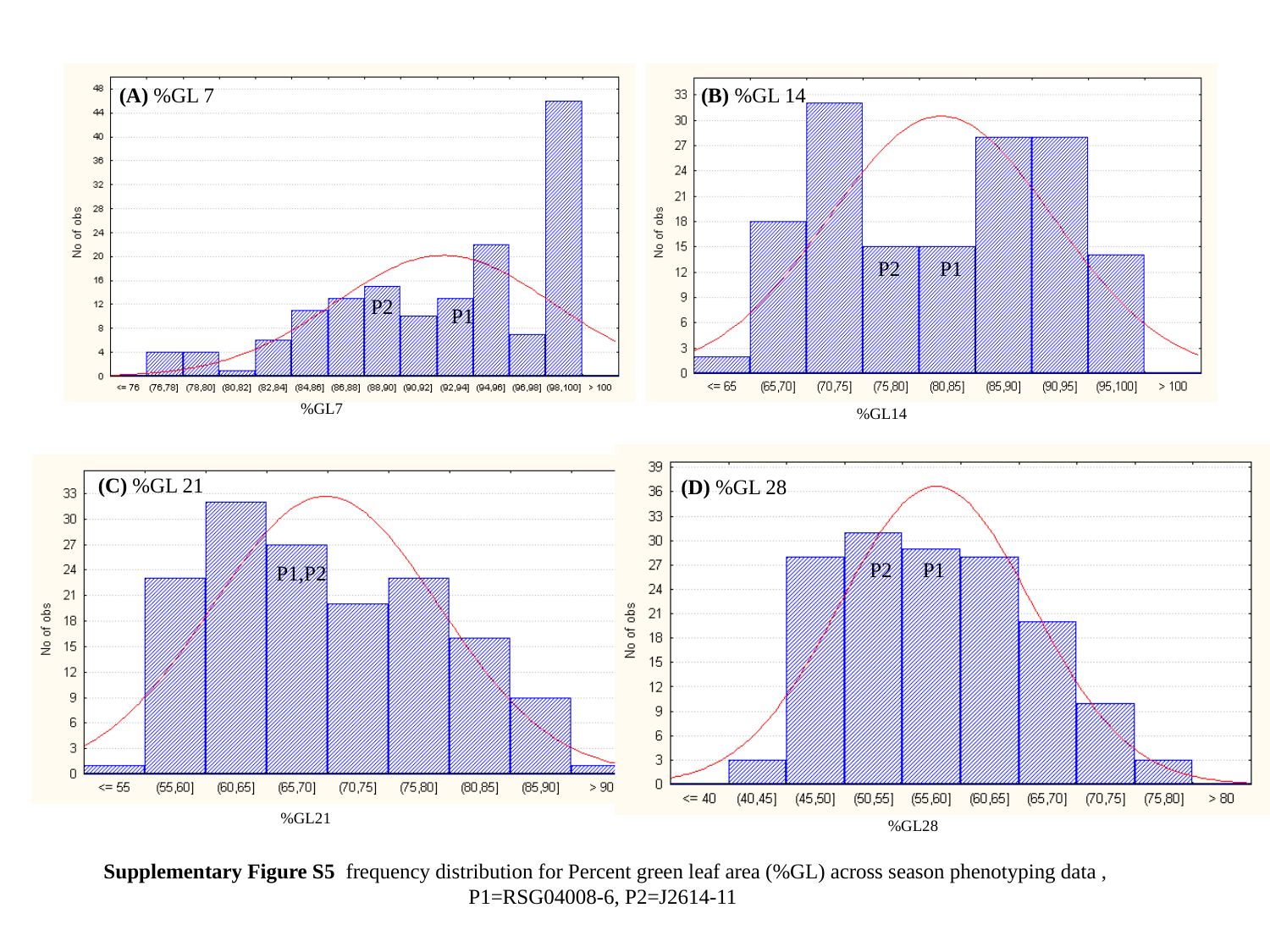

P2
P1
P2
P1
(A) %GL 7
(B) %GL 14
(C) %GL 21
(D) %GL 28
%GL7
 %GL14
P2
P1
P1,P2
 %GL21
 %GL28
# Supplementary Figure S5 frequency distribution for Percent green leaf area (%GL) across season phenotyping data , P1=RSG04008-6, P2=J2614-11

## Slide 8
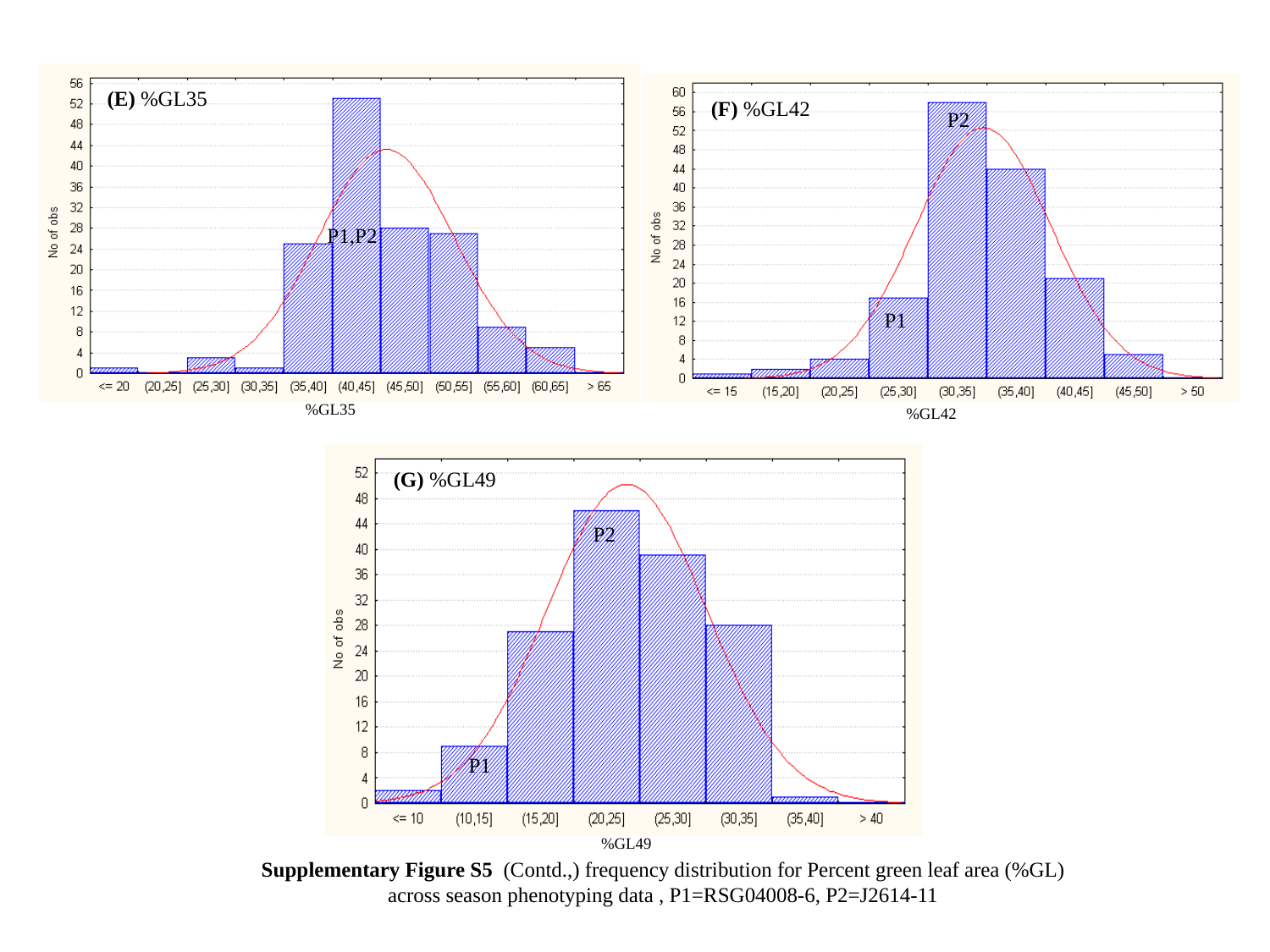

P2
P1
P1,P2
P1
P2
(E) %GL35
(F) %GL42
(G) %GL49
 %GL35
%GL42
%GL49
# Supplementary Figure S5 (Contd.,) frequency distribution for Percent green leaf area (%GL) across season phenotyping data , P1=RSG04008-6, P2=J2614-11

## Slide 9
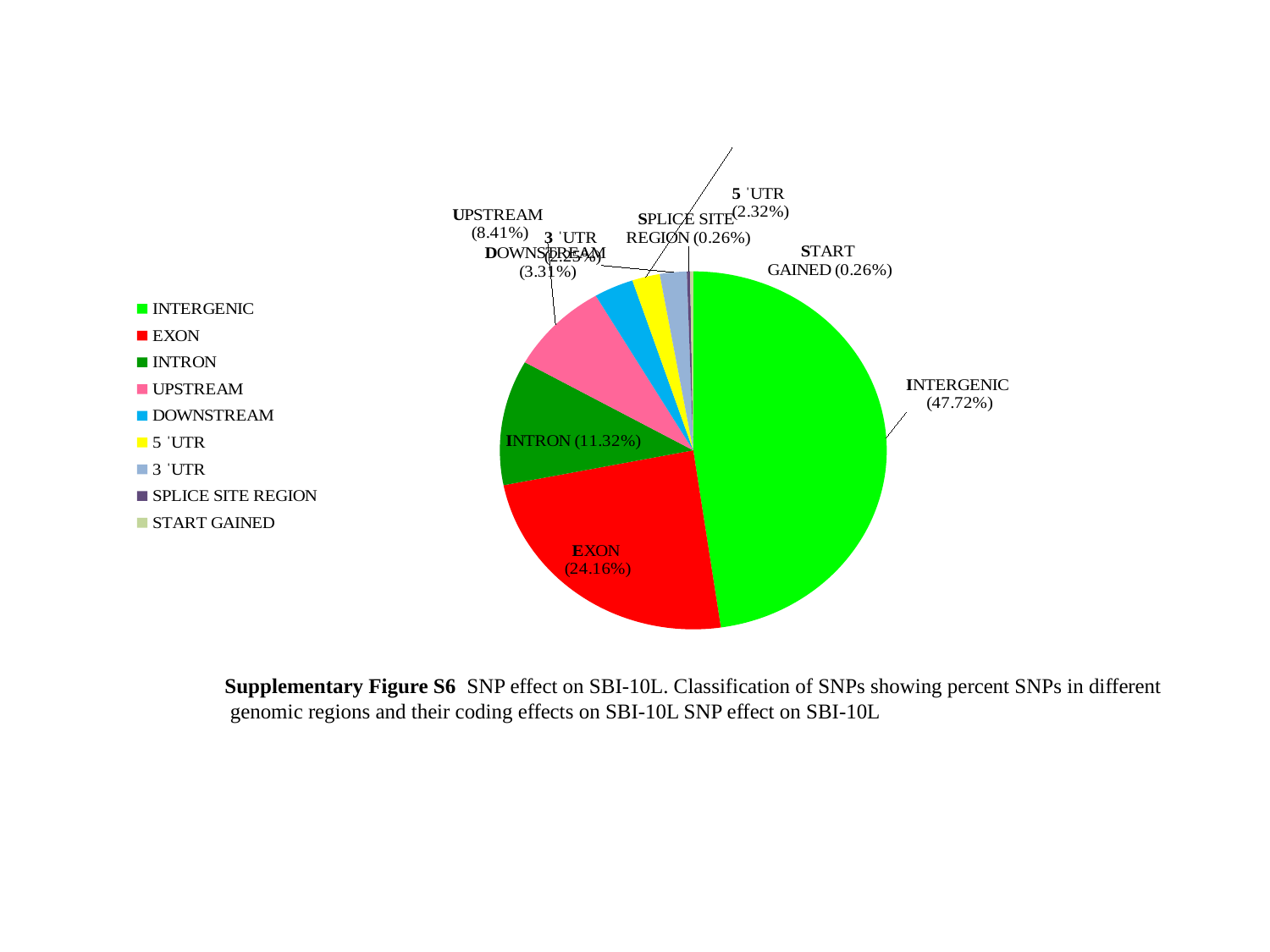

[unsupported chart]
Supplementary Figure S6 SNP effect on SBI-10L. Classification of SNPs showing percent SNPs in different
 genomic regions and their coding effects on SBI-10L SNP effect on SBI-10L

## Slide 10
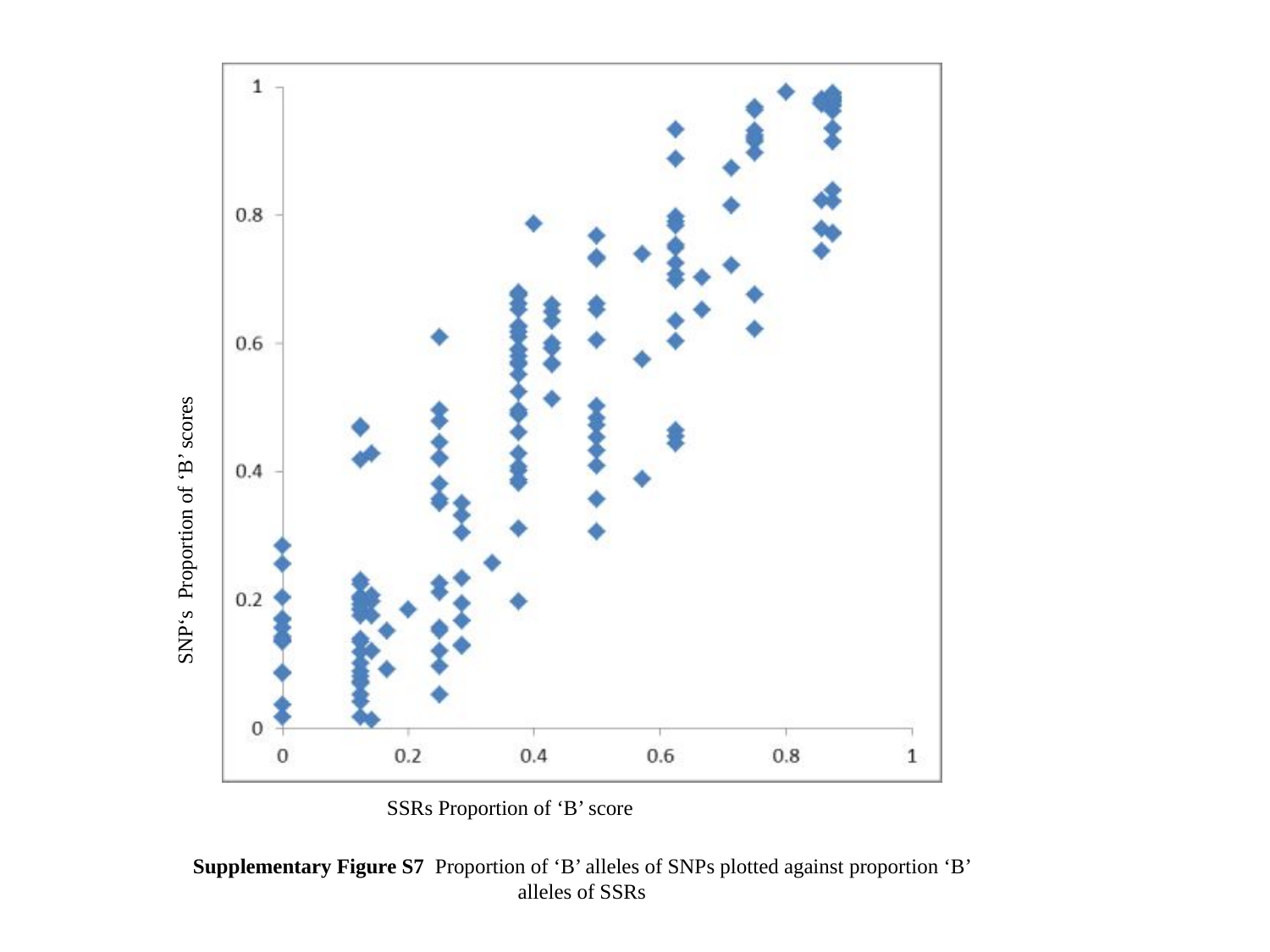

SNP‘s Proportion of ‘B’ scores
SSRs Proportion of ‘B’ score
Supplementary Figure S7 Proportion of ‘B’ alleles of SNPs plotted against proportion ‘B’ alleles of SSRs

## Slide 11
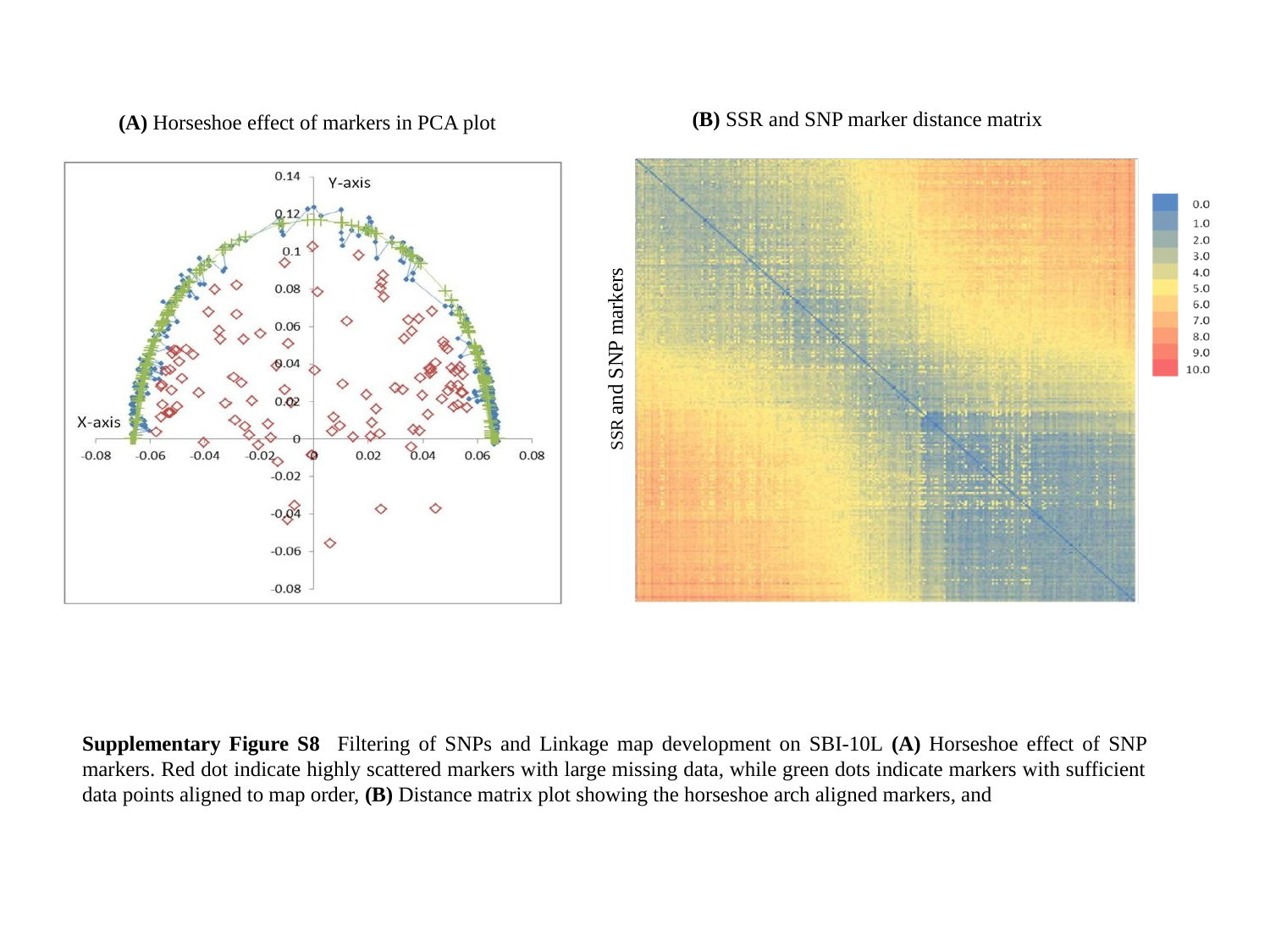

(A) Horseshoe effect of markers in PCA plot
(B) SSR and SNP marker distance matrix
SSR and SNP markers
Supplementary Figure S8 Filtering of SNPs and Linkage map development on SBI-10L (A) Horseshoe effect of SNP markers. Red dot indicate highly scattered markers with large missing data, while green dots indicate markers with sufficient data points aligned to map order, (B) Distance matrix plot showing the horseshoe arch aligned markers, and

## Slide 12
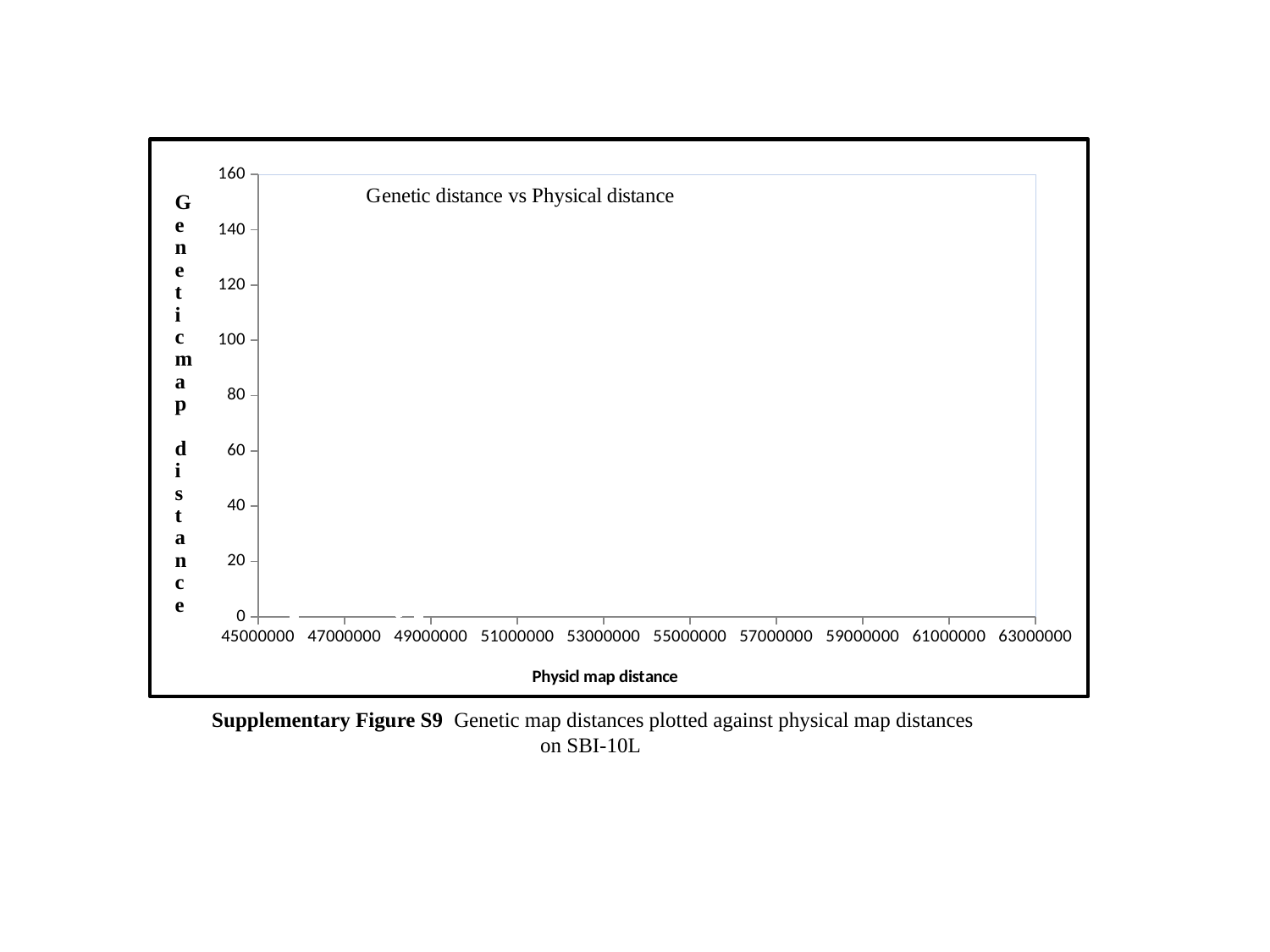

### Chart: Genetic distance vs Physical distance
| Category | |
|---|---|Supplementary Figure S9 Genetic map distances plotted against physical map distances on SBI-10L

## Slide 13
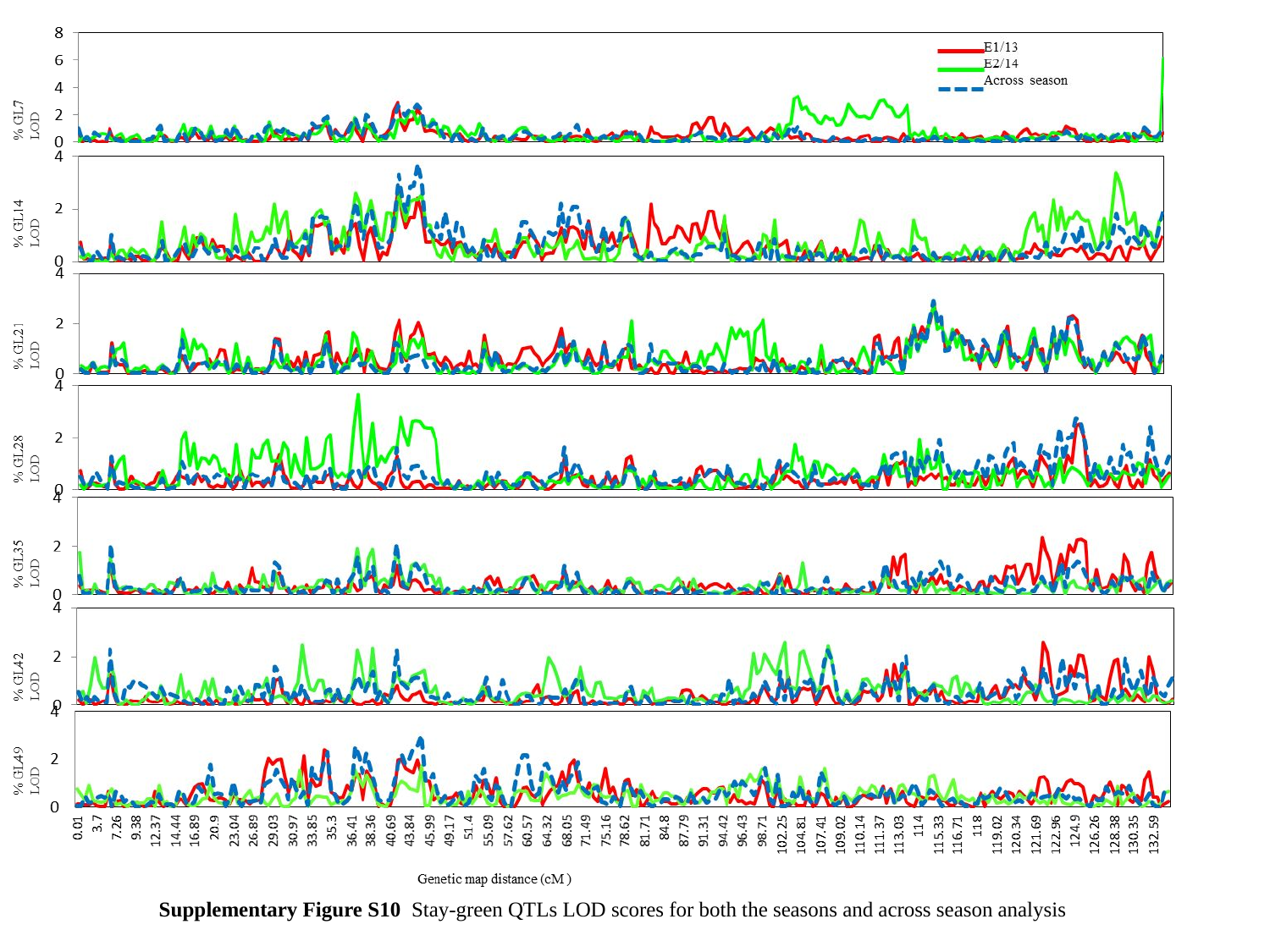

Supplementary Figure S10 Stay-green QTLs LOD scores for both the seasons and across season analysis
