# Supplementary material for: Fine-Mapping of Sorghum Stay-Green QTL on Chromosome10 Revealed Genes Associated with Delayed Senescence
Source: Genes (Basel). 2020 Sep 1;11(9):1026. doi: 10.3390/genes11091026 (PMC7565436; doi:10.3390/genes11091026)
Supplement: Supplementary file 1 [file genes-11-01026-s001.zip › STGFM_SUPPLETABLES_17082020.pdf]

Table S1: GBS SNP count chromosome wise

Sorghum Chromosome	SNP Count
1	5,121
2	3,880
3	3,891
4	3,081
5	2,046
6	2,738
7	1,990
8	1,928
9	2,032
**10**	**2,799**
Total	29,506

Table S2: Stay-green significant Marker trait associations

S No.	Trait	SNP	Allele	v3.1	Homolog Arabidopsis gene ids	Homolog rice gene ids	Functional annotation	P.value	maf	%R^2
1	%GL7_E1	S10_56428957	A/G	-	-	-	-	8.38E-03	0.50	25.87
2	%GL7_E1	S10_57663572	G/A	-	-	-	-	2.16E-03	0.31	27.35
3	%GL7_E1	S10_57714139	C/T	-	-	-	-	7.30E-03	0.47	26.02
4	%GL7_E1	S10_58758082	G/T	-	-	-	-	8.75E-03	0.44	25.83
5	%GL7_E1	S10_49536872	A/G	*Sobic.010G167500*	*AT5G25930.1*	*LOC_Os06g36270.1*	leucine-rich repeat family protein / protein kinase	7.25E-03	0.41	26.03
6	%GL7_E1	S10_53317190	C/T	*Sobic.010G191700*	*AT5G06900.1*	*LOC_Os06g41070.1*	cytochrome P450, putative, expressed	5.29E-03	0.34	26.37
7	%GL7_E1	S10_56428954	C/T	-	-	-	-	8.38E-03	0.50	25.87
8	%GL7_E1	S10_56475813	A/G	*Sobic.010G222600*	*AT3G06190.1*	*LOC_Os06g14060.2*	Zinc finger POZ domain protein	6.46E-03	0.48	26.15
9	%GL7_E1	S10_56914835	A/G	*Sobic.010G226400*	-	*LOC_Os02g12480.2*	putative unchracterised protein	7.69E-03	0.42	25.97
10	%GL7_E1	S10_57404251	G/A	*Sobic.010G231100*	*AT1G05200.1*	*LOC_Os06g46670.2*	PBP1_GABAb_receptor	8.14E-03	0.49	25.91
11	%GL7_E1	S10_57510942	A/G	-	-	-	40S ribosomal protein S14-1	3.00E-03	0.49	26.99
12	%GL7_E2	S10_56508509	A/G	-	-	-	-	5.51E-03	0.47	15.09
13	%GL7_E2	S10_56508557	A/T	-	-	-	-	5.51E-03	0.47	15.09
14	%GL7_E2	S10_56784622	T/G	-	-	-	-	2.23E-03	0.48	16.23
15	%GL7_E2	S10_58028206	C/T	-	-	-	-	1.90E-03	0.47	16.43
16	%GL7_E2	S10_58599753	A/G	-	-	-	-	5.94E-03	0.44	15.00
17	%GL7_E2	S10_54938193	T/G	*Sobic.010G206300*	*AT4G10320.1*	*LOC_Os06g43760.1*	isoleucyl-tRNA synthetase	7.58E-03	0.47	14.69
18	%GL7_E2	S10_54938226	A/T	*Sobic.010G206300*	*AT4G10320.1*	*LOC_Os06g43760.1*	isoleucyl-tRNA synthetase	7.58E-03	0.47	14.69
19	%GL7_E2	S10_54938227	T/A	*Sobic.010G206300*	*AT4G10320.1*	*LOC_Os06g43760.1*	isoleucyl-tRNA synthetase	7.58E-03	0.47	14.69
20	%GL7_E2	S10_54938228	C/T	*Sobic.010G206300*	*AT4G10320.1*	*LOC_Os06g43760.1*	isoleucyl-tRNA synthetase	7.58E-03	0.47	14.69
21	%GL7_E2	S10_55505116	T/C	*Sobic.010G212100*	*AT1G17890.1*	*LOC_Os06g44260.1*	Putative GDP-L-fucose synthase 2	1.12E-03	0.48	17.12
22	%GL7_E2	S10_58276305	C/A	*Sobic.010G241100*	*AT1G73620.1*	*LOC_Os06g47600.1*	Putative thaumatin-protein	2.27E-03	0.46	16.21
23	%GL7_E2	S10_58288752	A/G	*Sobic.010G241200*	*AT1G44350.1*	*LOC_Os06g47620.1*	AA-amino acid hydrolase	5.19E-03	0.46	15.17
24	%GL7_E2	S10_59292088	C/G	*Sobic.010G254100*	*AT2G07180.2*	*LOC_Os06g48980.1*	Catalytic domain of Protein Kinases	8.30E-03	0.47	14.58
25	%GL7_E2	S10_59292089	G/T	*Sobic.010G254100*	*AT2G07180.2*	*LOC_Os06g48980.1*	Catalytic domain of Protein Kinases	8.30E-03	0.47	14.58
26	%GL7_Across	S10_56428954	C/T	-	-	-	-	6.74E-03	0.50	15.06
27	%GL7_Across	S10_56428957	A/G	-	-	-	-	6.74E-03	0.50	15.06
28	%GL7_Across	S10_57663572	G/A	-	-	-	-	3.85E-03	0.31	15.69
29	%GL7_Across	S10_54897091	A/G	*Sobic.010G205900*	*AT4G04940.1*	*LOC_Os06g43690.1*	Transducin family protein / WD-40 repeat family	4.36E-03	0.48	15.55
30	%GL7_Across	S10_54919953	C/T	*Sobic.010G206200*	*AT3G52580.1*	*LOC_Os02g06700.2*	40S ribosomal protein S14 (RPS14C)	7.83E-03	0.35	14.89
31	%GL7_Across	S10_54938193	T/G	*Sobic.010G206300*	*AT4G10320.1*	*LOC_Os06g43760.1*	isoleucyl-tRNA synthetase	9.45E-03	0.47	14.68
32	%GL7_Across	S10_54938226	A/T	*Sobic.010G206300*	*AT4G10320.1*	*LOC_Os06g43760.1*	isoleucyl-tRNA synthetase	9.45E-03	0.47	14.68
33	%GL7_Across	S10_54938227	T/A	*Sobic.010G206300*	*AT4G10320.1*	*LOC_Os06g43760.1*	isoleucyl-tRNA synthetase	9.45E-03	0.47	14.68
34	%GL7_Across	S10_54938228	C/T	*Sobic.010G206300*	*AT4G10320.1*	*LOC_Os06g43760.1*	isoleucyl-tRNA synthetase	9.45E-03	0.47	14.68
35	%GL7_Across	S10_58288752	A/G	*Sobic.010G241200*	*AT1G44350.1*	*LOC_Os06g47620.1*	AA-amino acid hydrolase	9.63E-03	0.46	14.66
36	%GL7_Across	S10_60231203	C/T	*Sobic.010G267600*	*AT3G51250.1*	*LOC_Os06g50330.1*	Putative senescence-associated protein	7.03E-03	0.47	15.01
37	%GL7_Across	S10_60231243	T/G	*Sobic.010G267600*	*AT3G51250.1*	*LOC_Os06g50330.1*	Putative senescence-associated protein	7.03E-03	0.47	15.01
38	%GL14_E1	S10_56428954	C/T	-	-	-	-	3.35E-03	0.50	29.36
39	%GL14_E1	S10_56428957	A/G	-	-	-	-	3.35E-03	0.50	29.36
40	%GL14_E1	S10_57663572	G/A	-	-	-	-	9.64E-04	0.31	30.70
41	%GL14_E1	S10_58028206	C/T	-	-	-	-	6.25E-03	0.47	28.71
42	%GL14_E1	S10_49536872	A/G	*Sobic.010G167500*	*AT5G25930.1*	*LOC_Os06g36270.1*	leucine-rich repeat family protein / protein kinase	3.74E-03	0.41	29.24
43	%GL14_E1	S10_53317190	C/T	*Sobic.010G191700*	*AT5G06900.1*	*LOC_Os06g41070.1*	cytochrome P450, putative, expressed	3.64E-03	0.34	29.27
44	%GL14_E1	S10_56475813	A/G	*Sobic.010G222600*	*AT3G06190.1*	*LOC_Os06g14060.2*	Zinc finger POZ domain protein	3.29E-03	0.48	29.38
45	%GL14_E1	S10_57510942	A/G	-	-	-	40S ribosomal protein S14-1	3.01E-03	0.49	29.47
46	%GL14_E1	S10_58566780	G/C	*Sobic.010G245800*	*AT2G18980.1*	*LOC_Os06g48030.1*	Peroxidase 16 protein	5.45E-03	0.45	28.85
47	%GL14_E1	S10_59113364	G/A	*Sobic.010G251800*	*AT2G37880.1*	*LOC_Os05g20030.1*	DUF617; Protein of unknown function	6.38E-03	0.44	28.69
48	%GL14_E1	S10_59113369	G/C	*Sobic.010G251800*	*AT2G37880.1*	*LOC_Os05g20030.1*	DUF617; Protein of unknown function	6.38E-03	0.44	28.69
49	%GL14_E1	S10_59113372	G/T	*Sobic.010G251800*	*AT2G37880.1*	*LOC_Os05g20030.1*	DUF617; Protein of unknown function	6.38E-03	0.44	28.69
50	%GL14_E1	S10_59113374	C/T	*Sobic.010G251800*	*AT2G37880.1*	*LOC_Os05g20030.1*	DUF617; Protein of unknown function	6.38E-03	0.44	28.69
51	%GL14_E1	S10_59113412	A/T	*Sobic.010G251800*	*AT2G37880.1*	*LOC_Os05g20030.1*	DUF617; Protein of unknown function	6.38E-03	0.44	28.69
52	%GL14_E2	S10_50405318	A/G	-	-	-	-	1.14E-03	0.20	23.38
53	%GL14_E2	S10_50725358	G/A	*Sobic.010G172700*	*AT5G25900.1*	*LOC_Os06g37364.2*	GA3 (GA REQUIRING 3); ent-kaurene oxidase/	3.26E-03	0.33	22.16
54	%GL14_E2	S10_50865236	T/G	*Sobic.010G173400*	*AT4G26150.1*	*LOC_Os06g37450.1*	CGA1 (CYTOKININ-RESPONSIVE GATA	2.69E-03	0.26	22.38
55	%GL14_E2	S10_50945046	G/T	*Sobic.010G174100*	*AT4G20190.1*	*LOC_Os06g37600.1*	unknown protein	4.74E-03	0.43	21.73
56	%GL14_E2	S10_52971581	G/C	*Sobic.010G189200*	*AT1G03540.1*	*LOC_Os06g40860.1*	pentatricopeptide (PPR) repeat-containing	3.70E-03	0.40	22.01
57	%GL14_E2	S10_52971607	T/C	*Sobic.010G189200*	*AT1G03540.1*	*LOC_Os06g40860.1*	pentatricopeptide (PPR) repeat-containing	3.70E-03	0.40	22.01
58	%GL14_E2	S10_52971592	C/G	*Sobic.010G189200*	*AT1G03540.1*	*LOC_Os06g40860.1*	pentatricopeptide (PPR) repeat-containing	3.70E-03	0.40	22.01
59	%GL14_E2	S10_54841288	A/G	-	-	-	NBS-LRR disease resistance protein	3.27E-03	0.38	22.15
60	%GL14_E2	S10_54841304	C/T	-	-	-	NBS-LRR disease resistance protein	3.27E-03	0.38	22.15
61	%GL14_E2	S10_54841321	T/G	-	-	-	NBS-LRR disease resistance protein	3.27E-03	0.38	22.15
62	%GL14_Across	S10_45765686	G/C	-	-	-	-	7.66E-03	0.42	23.86
63	%GL14_Across	S10_48868738	G/A	-	-	-	-	5.10E-03	0.32	24.27
64	%GL14_Across	S10_56784622	T/G	-	-	-	-	3.48E-03	0.48	24.66
65	%GL14_Across	S10_57663572	G/A	-	-	-	-	8.46E-03	0.31	23.76
66	%GL14_Across	S10_49536872	A/G	*Sobic.010G167500*	*AT5G25930.1*	*LOC_Os06g36270.1*	leucine-rich repeat family protein / protein kinase	4.08E-04	0.41	26.92
67	%GL14_Across	S10_53333596	T/G	-	-	-	Putative uncharacterized protein	8.94E-03	0.38	23.71
68	%GL14_Across	S10_53333597	C/A	-	-	-	Putative uncharacterized protein	8.94E-03	0.38	23.71
69	%GL14_Across	S10_53333598	C/G	-	-	-	Putative uncharacterized protein	8.94E-03	0.38	23.71
70	%GL14_Across	S10_53333607	T/A	-	-	-	Putative uncharacterized protein	8.94E-03	0.38	23.71
71	%GL14_Across	S10_54388058	C/A	*Sobic.010G201100*	-	*LOC_Os06g43060.1*	zinc-binding family protein	9.50E-03	0.40	23.65
72	%GL14_Across	S10_54919953	C/T	*Sobic.010G206200*	*AT3G52580.1*	*LOC_Os02g06700.2*	40S ribosomal protein S14 (RPS14C)	1.70E-03	0.35	25.41
73	%GL21_E1	S10_56428954	C/T	-	-	-	-	3.83E-03	0.50	32.89
74	%GL21_E1	S10_56428957	A/G	-	-	-	-	3.83E-03	0.50	32.89
75	%GL21_E1	S10_56475813	A/G	*Sobic.010G222600*	*AT3G06190.1*	*LOC_Os06g14060.2*	Zinc finger POZ domain protein	3.43E-03	0.48	33.00
76	%GL21_E1	S10_59113364	G/A	*Sobic.010G251800*	*AT2G37880.1*	*LOC_Os05g20030.1*	DUF617; Protein of unknown function	1.15E-02	0.44	31.81

77	%GL21_E1	S10_59113369	G/C	*Sobic.010G251800*	*AT2G37880.1*	*LOC_Os05g20030.1*	DUF617; Protein of unknown function	1.15E-02	0.44	31.81
78	%GL21_E1	S10_59113372	G/T	*Sobic.010G251800*	*AT2G37880.1*	*LOC_Os05g20030.1*	DUF617; Protein of unknown function	1.15E-02	0.44	31.81
79	%GL21_E1	S10_59113374	C/T	*Sobic.010G251800*	*AT2G37880.1*	*LOC_Os05g20030.1*	DUF617; Protein of unknown function	1.15E-02	0.44	31.81
80	%GL21_E1	S10_59113412	A/T	*Sobic.010G251800*	*AT2G37880.1*	*LOC_Os05g20030.1*	DUF617; Protein of unknown function	1.15E-02	0.44	31.81
81	%GL21_E1	S10_60973291	A/G	*Sobic.010G276600*	*AT1G48410.1*	*LOC_Os06g51310.2*	Putative AGO1 homologous protein	4.83E-03	0.50	32.66
82	%GL21_E2	S10_48905718	G/A	-	-	-	-	1.33E-03	0.37	23.20
83	%GL21_E2	S10_50405318	A/G	-	-	-	-	1.14E-03	0.20	23.38
84	%GL21_E2	S10_50725358	G/A	*Sobic.010G172700*	*AT5G25900.1*	*LOC_Os06g37364.2*	GA3 (GA REQUIRING 3); ent-kaurene oxidase/	3.26E-03	0.33	22.16
85	%GL21_E2	S10_50865236	T/G	*Sobic.010G173400*	*AT4G26150.1*	*LOC_Os06g37450.1*	CGA1 (CYTOKININ-RESPONSIVE GATA	2.69E-03	0.26	22.38
86	%GL21_E2	S10_50945046	G/T	*Sobic.010G174100*	*AT4G20190.1*	*LOC_Os06g37600.1*	unknown protein	4.74E-03	0.43	21.73
87	%GL21_E2	S10_52971581	G/C	*Sobic.010G189200*	*AT1G03540.1*	*LOC_Os06g40860.1*	pentatricopeptide (PPR) repeat-containing	3.70E-03	0.40	22.01
88	%GL21_E2	S10_52971607	T/C	*Sobic.010G189200*	*AT1G03540.1*	*LOC_Os06g40860.1*	pentatricopeptide (PPR) repeat-containing	3.70E-03	0.40	22.01
89	%GL21_E2	S10_52971592	C/G	*Sobic.010G189200*	*AT1G03540.1*	*LOC_Os06g40860.1*	pentatricopeptide (PPR) repeat-containing	3.70E-03	0.40	22.01
90	%GL21_E2	S10_54841288	A/G	-	-	-	NBS-LRR disease resistance protein	3.27E-03	0.38	22.15
91	%GL21_E2	S10_54841304	C/T	-	-	-	NBS-LRR disease resistance protein	3.27E-03	0.38	22.15
92	%GL21_E2	S10_54841321	T/G	-	-	-	NBS-LRR disease resistance protein	3.27E-03	0.38	22.15
93	%GL21_E2	S10_59298151	A/G	*Sobic.010G254200*	*AT5G43270.2*	*LOC_Os06g49010.2*	Squamosa promoter-binding-like protein 12	6.67E-03	0.49	21.34
94	%GL21_Across	S10_56428954	C/T	-	-	-	-	7.16E-03	0.50	26.53
95	%GL21_Across	S10_56428957	A/G	-	-	-	-	7.16E-03	0.50	26.53
96	%GL21_Across	S10_49536872	A/G	*Sobic.010G167500*	*AT5G25930.1*	*LOC_Os06g36270.1*	leucine-rich repeat family protein / protein kinase	5.61E-03	0.41	26.77
97	%GL21_Across	S10_54897091	A/G	*Sobic.010G205900*	*AT4G04940.1*	*LOC_Os06g43690.1*	transducin family protein / WD-40 repeat family	6.13E-03	0.48	26.68
98	%GL21_Across	S10_60973291	A/G	*Sobic.010G276600*	*AT1G48410.1*	*LOC_Os06g51310.2*	Putative AGO1 homologous protein	7.78E-03	0.50	26.45
99	%GL28_E1	S10_56428954	C/T	-	-	-	-	4.46E-03	0.50	30.58
100	%GL28_E1	S10_56428957	A/G	-	-	-	-	4.46E-03	0.50	30.58
101	%GL28_E1	S10_56475813	A/G	*Sobic.010G222600*	*AT3G06190.1*	*LOC_Os06g14060.2*	Zinc finger POZ domain protein	8.90E-03	0.48	29.89
102	%GL28_E1	S10_60973291	A/G	*Sobic.010G276600*	*AT1G48410.1*	*LOC_Os06g51310.2*	Putative AGO1 homologous protein	4.03E-03	0.50	30.69
103	%GL28_E2	S10_50405318	A/G	-	-	-	-	4.35E-03	0.20	15.78
104	%GL28_E2	S10_54915154	G/A	-	-	-	-	2.35E-03	0.33	16.54
105	%GL28_E2	S10_54915161	G/C	-	-	-	-	2.35E-03	0.33	16.54
106	%GL28_E2	S10_54915163	G/A	-	-	-	-	2.35E-03	0.33	16.54
107	%GL28_E2	S10_46573203	A/T	*Sobic.010G158400*	*AT4G40070.1*	*LOC_Os06g34530.1*	zinc finger a RING-type-/ E3 ubiquitin ligase	5.23E-03	0.41	15.55
108	%GL28_E2	S10_50945046	G/T	*Sobic.010G174100*	*AT4G20190.1*	*LOC_Os06g37600.1*	unknown protein	5.42E-03	0.43	15.51
109	%GL28_Across	S10_49536872	A/G	*Sobic.010G167500*	*AT5G25930.1*	*LOC_Os06g36270.1*	leucine-rich repeat family protein / protein kinase	3.68E-03	0.41	23.80
110	%GL28_Across	S10_54897091	A/G	*Sobic.010G205900*	*AT4G04940.1*	*LOC_Os06g43690.1*	transducin family protein / WD-40 repeat family	9.38E-03	0.48	22.85
111	%GL28_Across	S10_54919953	C/T	*Sobic.010G206200*	*AT3G52580.1*	*LOC_Os02g06700.2*	40S ribosomal protein S14 (RPS14C)	9.49E-03	0.35	22.84
112	%GL28_Across	S10_60973291	A/G	*Sobic.010G276600*	*AT1G48410.1*	*LOC_Os06g51310.2*	Putative AGO1 homologous protein	1.00E-02	0.50	22.79
113	%GL35_E1	S10_53690458	A/T	-	-	-	-	7.16E-03	0.41	23.45
114	%GL35_E1	S10_56428954	C/T	-	-	-	-	7.79E-03	0.50	23.36
115	%GL35_E1	S10_56428957	A/G	-	-	-	-	7.79E-03	0.50	23.36
116	%GL35_E1	S10_49536872	A/G	*Sobic.010G167500*	*AT5G25930.1*	*LOC_Os06g36270.1*	leucine-rich repeat family protein / protein kinase	6.85E-03	0.41	23.50
117	%GL35_E1	S10_53972403	A/G	-	-	-	zinc-binding family protein	8.05E-03	0.35	23.32
118	%GL35_E1	S10_56475813	A/G	*Sobic.010G222600*	*AT3G06190.1*	*LOC_Os06g14060.2*	Zinc finger POZ domain protein	8.36E-03	0.48	23.28
119	%GL35_E1	S10_60973291	A/G	*Sobic.010G276600*	*AT1G48410.1*	*LOC_Os06g51310.2*	Putative AGO1 homologous protein	2.48E-03	0.50	24.62
120	%GL35_E2	S10_48905718	G/A	-	-	-	-	3.20E-03	0.37	21.98
121	%GL35_E2	S10_54897091	A/G	*Sobic.010G205900*	*AT4G04940.1*	*LOC_Os06g43690.1*	transducin family protein / WD-40 repeat family	9.99E-03	0.48	20.65
122	%GL35_Across	S10_45765686	G/C	-	-	-	-	8.35E-03	0.42	17.14
123	%GL35_Across	S10_54897091	A/G	*Sobic.010G205900*	*AT4G04940.1*	*LOC_Os06g43690.1*	transducin family protein / WD-40 repeat family	1.11E-03	0.48	19.39
124	%GL42_E1	S10_53690458	A/T	-	-	-	-	2.70E-03	0.41	19.59
125	%GL42_E1	S10_53972403	A/G	-	-	-	zinc-binding family protein	2.66E-03	0.35	19.61
126	%GL42_E1	S10_56475813	A/G	*Sobic.010G222600*	*AT3G06190.1*	*LOC_Os06g14060.2*	Zinc finger POZ domain protein	6.06E-03	0.48	18.64
127	%GL42_E1	S10_60654069	C/T	-	-	-	PLN03210; Resistant to P. syringae 6;	6.00E-03	0.38	18.65
128	%GL42_E1	S10_60973291	A/G	*Sobic.010G276600*	*AT1G48410.1*	*LOC_Os06g51310.2*	Putative AGO1 homologous protein	2.06E-03	0.50	19.92
129	%GL42_E2	S10_48512575	A/G	-	-	-	-	3.00E-04	0.35	16.38
130	%GL42_E2	S10_48512624	C/T	-	-	-	-	8.12E-04	0.34	15.01
131	%GL42_E2	S10_57512927	C/A	*Sobic.010G231900*	*AT3G20570.1*	*LOC_Os06g46740.1*	OsENODL1_like; Early nodulin-like protein	7.24E-03	0.49	12.12
132	%GL42_E2	S10_60058846	G/A	*Sobic.010G264400*	*AT2G46700.1*	*LOC_Os06g50030.1*	Calcium/calmodulin-dependent protein kinase	9.53E-03	0.43	11.77
133	%GL42_E2	S10_60058874	G/A	*Sobic.010G264400*	*AT2G46700.1*	*LOC_Os06g50030.1*	Calcium/calmodulin-dependent protein kinase	9.53E-03	0.43	11.77
134	%GL42_E2	S10_60058876	T/A	*Sobic.010G264400*	*AT2G46700.1*	*LOC_Os06g50030.1*	Calcium/calmodulin-dependent protein kinase	9.53E-03	0.43	11.77
135	%GL42_Across	S10_53400859	T/C	-	-	-	-	7.16E-03	0.41	10.28
136	%GL42_Across	S10_53400870	C/G	-	-	-	-	7.16E-03	0.41	10.28
137	%GL42_Across	S10_56987389	C/G	-	-	-	-	8.78E-03	0.50	10.04
138	%GL42_Across	S10_54897091	A/G	*Sobic.010G205900*	*AT4G04940.1*	*LOC_Os06g43690.1*	transducin family protein / WD-40 repeat family	5.10E-03	0.48	10.68
139	%GL42_Across	S10_57512927	C/A	*Sobic.010G231900*	*AT3G20570.1*	*LOC_Os06g46740.1*	OsENODL1_like; Early nodulin-like protein	7.38E-03	0.49	10.24
140	%GL49_E1	S10_49892187	A/G	-	-	-	-	7.98E-03	0.28	27.24
141	%GL49_E1	S10_49892188	G/A	-	-	-	-	7.98E-03	0.28	27.24
142	%GL49_E1	S10_49536872	A/G	*Sobic.010G167500*	*AT5G25930.1*	*LOC_Os06g36270.1*	leucine-rich repeat family protein / protein kinase	3.78E-04	0.41	30.52
143	%GL49_E1	S10_54841484	C/A	-	-	-	NBS-LRR disease resistance protein	5.95E-03	0.44	27.54
144	%GL49_E1	S10_54841489	A/C	-	-	-	NBS-LRR disease resistance protein	5.95E-03	0.44	27.54
145	%GL49_E1	S10_55953518	C/T	*Sobic.010G216200*	*AT5G20500.1*	*LOC_Os06g44910.1*	Glutaredoxin-C8 precursor	3.22E-03	0.48	28.19
146	%GL49_E1	S10_55953554	A/T	*Sobic.010G216200*	*AT5G20500.1*	*LOC_Os06g44910.1*	Glutaredoxin-C8 precursor	3.22E-03	0.48	28.19
147	%GL49_E1	S10_56475813	A/G	*Sobic.010G222600*	*AT3G06190.1*	*LOC_Os06g14060.2*	Zinc finger POZ domain protein	7.80E-03	0.48	27.26
148	%GL49_E2	S10_60091545	C/A	-	-	-	-	8.28E-03	0.42	11.97
149	%GL49_E2	S10_60091546	T/A	-	-	-	-	8.28E-03	0.42	11.97
150	%GL49_E2	S10_60091550	A/C	-	-	-	-	8.28E-03	0.42	11.97
151	%GL49_E2	S10_60091555	C/A	-	-	-	-	8.28E-03	0.42	11.97
152	%GL49_E2	S10_46606416	C/T	*Sobic.010G158400*	*AT4G40070.1*	*LOC_Os06g34530.1*	zinc finger a RING-type-/ E3 ubiquitin ligase	3.13E-03	0.47	13.22
153	%GL49_E2	S10_48198669	C/G	*Sobic.010G163600*	*AT4G18800.1*	*LOC_Os06g35814.1*	GTP-binding protein (endocytosis)	9.54E-03	0.47	11.79
154	%GL49_E2	S10_55505116	T/C	*Sobic.010G212100*	*AT1G17890.1*	*LOC_Os06g44260.1*	Putative GDP-L-fucose synthase 2	4.73E-03	0.48	12.68

155	%GL49_E2	S10_60058846	G/A	*Sobic.010G264400*	*AT2G46700.1*	*LOC_Os06g50030.1*	Calcium/calmodulin-dependent protein kinase	4.25E-03	0.43	12.82
156	%GL49_E2	S10_60058874	G/A	*Sobic.010G264400*	*AT2G46700.1*	*LOC_Os06g50030.1*	Calcium/calmodulin-dependent protein kinase	4.25E-03	0.43	12.82
157	%GL49_E2	S10_60058876	T/A	*Sobic.010G264400*	*AT2G46700.1*	*LOC_Os06g50030.1*	Calcium/calmodulin-dependent protein kinase	4.25E-03	0.43	12.82
158	%GL49_Across	S10_51738778	T/C	-	-	-	-	9.14E-03	0.32	11.99
159	%GL49_Across	S10_49536872	A/G	*Sobic.010G167500*	*AT5G25930.1*	*LOC_Os06g36270.1*	leucine-rich repeat family protein / protein kinase	4.28E-03	0.41	12.87
160	%GL49_Across	S10_50505397	C/T	-	-	-	Putative uncharacterized protein	8.70E-03	0.39	12.05
161	%GL49_Across	S10_50505399	T/G	-	-	-	Putative uncharacterized protein	8.70E-03	0.39	12.05
162	%GL49_Across	S10_50505400	T/C	-	-	-	Putative uncharacterized protein	8.70E-03	0.39	12.05
163	%GL49_Across	S10_54841484	C/A	-	-	-	NBS-LRR disease resistance protein	3.39E-03	0.44	13.14
164	%GL49_Across	S10_54841489	A/C	-	-	-	NBS-LRR disease resistance protein	3.39E-03	0.44	13.14
165	%GL49_Across	S10_54919953	C/T	*Sobic.010G206200*	*AT3G52580.1*	*LOC_Os02g06700.2*	40S ribosomal protein S14 (RPS14C)	8.20E-03	0.35	12.11
166	%GL49_Across	S10_56475813	A/G	*Sobic.010G222600*	*AT3G06190.1*	*LOC_Os06g14060.2*	Zinc finger POZ domain protein	9.86E-03	0.48	11.90
167	%GL49_Across	S10_60058846	G/A	*Sobic.010G264400*	*AT2G46700.1*	*LOC_Os06g50030.1*	Calcium/calmodulin-dependent protein kinase	6.17E-03	0.43	12.44
168	%GL49_Across	S10_60058874	G/A	*Sobic.010G264400*	*AT2G46700.1*	*LOC_Os06g50030.1*	Calcium/calmodulin-dependent protein kinase	6.17E-03	0.43	12.44
169	%GL49_Across	S10_60058876	T/A	*Sobic.010G264400*	*AT2G46700.1*	*LOC_Os06g50030.1*	Calcium/calmodulin-dependent protein kinase	6.17E-03	0.43	12.44

Table S3: Candidate genes in the target QTL regions of stay-green on sorghum chromosome SBI-10L

stg QTL	SNP	Allele	name V3.1	Functional role	Arabidopsis gene	ids
**%GL7**	S10_58565687	G/C	*Sobic.010G245800*	similar to Peroxidase 16 protein	*AT2G18980.1*	*LOC_Os06g48030.1*
**%GL7**	S10_58610981	A/C	*Sobic.010G246400*	similar to Putative uncharacterized protein	*AT5G57560.1*	*LOC_Os06g48160.1*
**%GL7**	S10_58901865	G/C	-	similar to Mitogen-activated protein kinase 3	-	*LOC_Os06g48590.1*
**%GL7**	S10_58920175	T/G	*Sobic.010G249800*	similar to Os03g0824600 protein; UDP glycosyl transferase	*AT3G55700.1*	*LOC_Os03g60960.1*
**%GL7**	S10_58950644	C/G	*Sobic.010G250400*	similar to Putative meiotic serine proteinase	*AT4G30020.1*	*LOC_Os06g48650.1*
**%GL7**	S10_59113364	G/A	*Sobic.010G251800*	similar to Putative uncharacterized protein	*AT2G37880.1*	*LOC_Os05g20030.1*
**%GL7**	S10_59294116	G/C	*Sobic.010G254100*	similar to Putative uncharacterized protein	*AT2G07180.2*	*LOC_Os06g48980.1*
**%GL7**	S10_59298151	A/G	*Sobic.010G254200*	similar to Squamosa promoter-binding-like protein 12	*AT5G43270.2*	*LOC_Os06g49010.1*
**%GL7**	S10_59336236	G/T	-	similar to hAT dimerisation domain-containing protein-like	-	*LOC_Os06g49050.1*
**%GL7**	S10_59342868	C/G	-	similar to translation initiation factor IF-2	-	
**%GL7**	S10_59422845	C/T	*Sobic.010G255700*	similar to Os02g0137100 protein	*AT3G10250.2*	*LOC_Os06g49180.1*
**%GL7**	S10_59593663	C/T	*Sobic.010G258600*	similar to Pentatricopeptide (PPR) repeat-containing protein-like	*AT2G36730.1*	*LOC_Os09g24640.1*
**%GL7**	S10_59697086	G/A	*Sobic.010G260300*	similar to Putative uncharacterized protein	-	*LOC_Os06g49700.1*
**%GL7**	S10_59837691	C/T	*Sobic.010G261800*	similar to MADS box transcription factor	*AT3G54340.1*	*LOC_Os06g49840.1*
**%GL7**	S10_60117639	A/T	*Sobic.010G265500*	weakly similar to Putative uncharacterized protein	*AT4G00895.1*	*LOC_Os06g50070.1*
**%GL7**	S10_60243256	A/C	*Sobic.010G267700*	similar to Putative receptor protein kinase	*AT1G75820.1*	*LOC_Os06g50340.1*
**%GL7**	S10_60308400	G/C	*Sobic.010G268400*	similar to Aspartic proteinase nepenthesin II-like	*AT4G35880.1*	*LOC_Os06g50390.1*
**%GL14**	S10_53235767	A/G	-	Helix-loop-helix DNA-binding	-	
**%GL14**	S10_53870950	G/T	*Sobic.010G195600*	NADP binding domain	*AT2G33590.1*	
**%GL14**	S10_54388058	C/A	*Sobic.010G201100*	weakly similar to Putative uncharacterized protein	-	*LOC_Os06g43060.1*
**%GL14**	S10_54491782	C/A	*Sobic.010G202100*	similar to Putative uncharacterized protein	*AT4G37250.1*	*LOC_Os01g33090.1*
**%GL14**	S10_54838977	C/T	-	NBS-LRR disease resistance protein	-	
**%GL14**	S10_54891180	A/T	*Sobic.010G205800*	similar to Ankyrin repeat-containing protein-like	*AT5G20350.1*	*LOC_Os06g43680.1*
**%GL14**	S10_54899228	A/T	*Sobic.010G205900*	WD40 repeat family protein (transducin protein)	*AT4G04940.1*	*LOC_Os06g43690.1*
**%GL14**	S10_55181668	G/A	-	Putative prolylcarboxypeptidase isoform 1	-	
**%GL14**	S10_60231203	C/T	*Sobic.010G267600*	similar to Putative senescence-associated protein	*AT3G51250.1*	*LOC_Os06g50330.1*
**%GL14**	S10_60468748	A/C	*Sobic.010G270300*	similar to Senescence-associated protein	*AT4G35770.1*	*LOC_Os06g50930.1*
**%GL14**	S10_60557352	C/T	-	Exo70 exocyst complex subunit (autophagycytosis/programmed	-	*LOC_Os09g17810.1*
**%GL14**	S10_60566724	G/C	*Sobic.010G271700*	Predicted protein	*AT1G07725.1*	*LOC_Os05g30660.1*
**%GL14**	S10_60577527	G/T	-	Predicted protein;cullin protein	-	
**%GL14**	S10_60602919	C/A	-	Predicted protein (autophagy related transport to vacuole)	-	*LOC_Os05g30640.1*
**%GL14**	S10_60619195	T/G	*Sobic.010G272800*	similar to Cell division protease ftsH homolog, chloroplast precu	*AT5G42270.1*	*LOC_Os06g51029.1*
**%GL14**	S10_60684319	A/T	*Sobic.010G273700*	No apical meristem (NAM) protein	*AT2G17040.1*	
**%GL14**	S10_60695074	G/A	*Sobic.010G273800*	Starch branching enzyme I precursor	*AT5G03650.1*	
**%GL14**	S10_60973291	A/G	*Sobic.010G276600*	similar to Putative AGO1 homologous protein	*AT1G48410.1*	*LOC_Os06g51310.1*
**%GL21**	S10_59491003	C/A	-	MATE efflux family protein	-	
**%GL21**	S10_59620312	A/C	*Sobic.010G259200*	similar to Putative uncharacterized protein P0655A07.24 LEA2	*AT5G45320.1*	*LOC_Os06g49650.1*
**%GL21**	S10_59690880	G/A	-	anthocyanin1	-	
**%GL21**	S10_59749988	A/G	*Sobic.010G261200*	similar to Putative uncharacterized protein	*AT1G62790.1*	*LOC_Os06g49770.1*
**%GL21**	S10_59848048	G/A	*Sobic.010G261900*	similar to Putative uncharacterized protein	*AT3G51070.1*	*LOC_Os06g49860.1*
**%GL21**	S10_60120147	T/A	*Sobic.010G265600*	similar to Os06g0714800 protein	*AT2G44670.1*	*LOC_Os06g50080.1*
**%GL28**	S10_49621607	T/C	*Sobic.010G167900*	similar to Chromosome chr9 scaffold_7, whole genome shotgun	*AT4G32850.5*	*LOC_Os06g36360.1*
**%GL28**	S10_52972082	G/A	*Sobic.010G189200*	similar to Pentatricopeptide (PPR) repeat-containing protein-like	*AT1G03540.1*	*LOC_Os06g40860.1*
**%GL28**	S10_53353127	G/A	-	Signal transduction receptor-regulatr domain	-	
**%GL28**	S10_53454779	T/C	-	similar to Putative leucine zipper	-	*LOC_Os06g41100.1*
**%GL28**	S10_54118313	A/G	*Sobic.010G198000*	similar to Chromosome chr14 scaffold_9, whole genome shotgu	*AT5G38530.1*	*LOC_Os06g42560.1*
**%GL28**	S10_54575547	C/T	*Sobic.010G202700*	similar to Glossy15/Apetal2	*AT2G28550.3*	*LOC_Os06g43220.1*
**%GL28**	S10_60551643	C/T	*Sobic.010G271400*	similar to Putative uncharacterized protein P0548E04.19	*AT1G76240.1*	*LOC_Os06g51010.1*
**%GL35**	S10_60059042	C/A	*Sobic.010G264400*	Ca/calmodulin dependant protein kinase		
**%GL35**	S10_60173717	A/C	-	rod shape-determining protein MreC	-	
**%GL35**	S10_60214866	C/A	*Sobic.010G267300*	similar to Putative uncharacterized protein	*AT4G36010.1*	*LOC_Os06g50240.1*
**%GL35**	S10_60613735	G/C	*Sobic.010G272700*	Predicted protein	*AT5G50380.1*	*LOC_Os08g13570.1*
**%GL35**	S10_61153461	A/G	*Sobic.010G279100*	similar to Os06g0731700 protein	-	*LOC_Os06g51500.1*
**%GL42**	S10_53077439	G/T	*Sobic.010G189600*	similar to Seven transmembrane protein Mlo7	*AT4G24250.1*	*LOC_Os02g10350.1*
**%GL42**	S10_54005372	A/G	*Sobic.010G196900*	similar to Os01g0609200 protein	*AT1G15520.1*	*LOC_Os01g42380.1*
**%GL42**	S10_55904321	T/G	*Sobic.010G215700*	similar to SBP-domain protein 4	*AT1G02065.1*	*LOC_Os06g44860.1*
**%GL42**	S10_57507647	C/T	-	similar to Putative uncharacterized protein	-	*LOC_Os06g46700.1*
**%GL42**	S10_57660753	C/A	*Sobic.010G234000*	similar to EF-hand Ca2+-binding protein CCD1	*AT2G46600.1*	*LOC_Os06g46950.1*
**%GL42**	S10_57692900	G/C	*Sobic.010G234400*	weakly similar to O-methyltransferase ZRP4	*AT4G35160.1*	
**%GL42**	S10_57783305	T/G	*Sobic.010G235500*	Predicted protein	*AT5G18780.2*	*LOC_Os02g06520.1*
**%GL42**	S10_57809876	C/G	-	F-box/LRR	-	
**%GL42**	S10_58276305	C/A	*Sobic.010G241100*	similar to Putative thaumatin-protein	*AT1G73620.1*	*LOC_Os06g47600.1*
**%GL42**	S10_58908285	A/C	*Sobic.010G249600*	similar to Putative uncharacterized protein	*AT5G57170.1*	*LOC_Os06g48600.1*
**%GL42**	S10_60059042	C/A	*Sobic.010G264400*	Ca/calmodulin dependant protein kinase		
**%GL49**	S10_54899228	A/T	*Sobic.010G205900*	WD40 repeat family protein (transducin protein)	*AT4G04940.1*	*LOC_Os06g43690.1*
**%GL49**	S10_51163559	T/C	-	-	-	-
**%GL49**	S10_54575547	C/T	*Sobic.010G202700*	similar to Glossy15/Apetal2	*AT2G28550.3*	*LOC_Os06g43220.1*
**%GL49**	S10_54841288	A/G	-	NBS-LRR disease resistance protein	-	-

Table S4 Climate variation for E1 & E2

Month		Rain (mm)		Evap		Max Temp°C		Rel Humidity1		Wind Velocity	
**E1 (13)**	**E2 (14)**	**E1_Rain**	**E2_Rain**	**E1_Evap**	**E2_Evap**	**E1_Temp**	**E2_Temp**	**E1**	**E2**	**E1**	**E2**
**Jan-13**	**Nov-13**	1	20.69	135.9	104.2	30.6	28.42	91.93	92.26	5.65	3.77
**Feb-13**	**Dec-13**	10.09	0	168.69	107.7	31.06	27.77	85.53	94.19	7.01	4.17
**Mar-13**	**Jan-14**	0	0	277	126.39	35.66	28.38	72.16	91.19	7.23	6.45
**Apr-13**	**Feb-14**	60.39	0	276.59	160.69	37.47	30.49	73.33	82.6	7.49	7.06
**May-13**	**Mar-14**	3.39	12.8	365.19	206.9	40.23	33.14	60.67	77.38	9.27	7.29
	**Mean**	14.9	6.6	244.6	141.1	**35**	**29.6**	76.7	87.5	7.3	5.7

SNP	Chromosome	Position	P.value	Trait
S1_12781217	1	12781217	7.62E-06	GL28_ac
S1_12781217	1	12781217	7.72E-06	GL14_14/E2
S1_12781217	1	12781217	9.96E-06	GL28_14/E2
S1_12781217	1	12781217	1.33E-05	GL21_ac
S1_12781217	1	12781217	1.53E-05	GL14_ac
S1_12156535	1	12156535	1.75E-05	GL28_13/E1
S1_12156535	1	12156535	2.61E-05	GL35_13/E1
S1_12781217	1	12781217	3.12E-05	GL35_ac
S1_12156535	1	12156535	3.57E-05	GL21_13/E1
S1_16647171	1	16647171	7.65E-05	GL49_13/E1
S1_18315751	1	18315751	7.80E-05	GL14_ac
S1_18315763	1	18315763	7.80E-05	GL14_ac
S1_18315761	1	18315761	7.80E-05	GL14_ac
S1_18315778	1	18315778	7.80E-05	GL14_ac
S1_57665002	1	57665002	7.99E-05	GL21_13/E1
S1_12564526	1	12564526	8.63E-05	GL21_ac
S1_12564526	1	12564526	9.75E-05	GL14_14/E2
S1_12781217	1	12781217	9.78E-05	GL21_14/E2
S1_80482739	1	80482739	9.94E-05	GL21_14/E2
S1_80482740	1	80482740	9.94E-05	GL21_14/E2
S1_80482739	1	80482739	1.03E-04	GL28_14/E2
S1_80482740	1	80482740	1.03E-04	GL28_14/E2
S1_12106664	1	12106664	1.09E-04	GL21_13/E1
S1_18315751	1	18315751	1.14E-04	GL7_13/E1
S1_18315761	1	18315761	1.14E-04	GL7_13/E1
S1_18315763	1	18315763	1.14E-04	GL7_13/E1
S1_18315778	1	18315778	1.14E-04	GL7_13/E1
S1_12106664	1	12106664	1.26E-04	GL21_ac
S1_12564526	1	12564526	1.28E-04	GL14_ac
S1_12106664	1	12106664	1.37E-04	GL28_13/E1
S1_75427740	1	75427740	1.46E-04	GL49_ac
S1_12156535	1	12156535	1.55E-04	GL21_ac
S1_75427740	1	75427740	1.57E-04	GL28_13/E1
S1_12564526	1	12564526	1.59E-04	GL21_13/E1
S1_12106664	1	12106664	1.77E-04	GL35_13/E1
S1_12106664	1	12106664	1.79E-04	GL14_ac
S1_14690005	1	14690005	1.82E-04	GL21_14/E2
S1_57665002	1	57665002	1.89E-04	GL7_13/E1
S1_12564526	1	12564526	1.92E-04	GL28_ac
S1_75427740	1	75427740	1.97E-04	GL21_13/E1
S1_12106664	1	12106664	2.20E-04	GL35_ac
S1_12781217	1	12781217	2.27E-04	GL21_13/E1
S1_12122950	1	12122950	2.38E-04	GL14_ac
S1_12106664	1	12106664	2.51E-04	GL7_13/E1
S1_12781217	1	12781217	2.52E-04	GL35_13/E1
S1_12564742	1	12564742	2.54E-04	GL14_14/E2
S1_57665002	1	57665002	2.54E-04	GL14_13/E1
S1_75427740	1	75427740	2.59E-04	GL21_ac
S1_8931365	1	8931365	2.68E-04	GL35_14/E2
S1_18315751	1	18315751	2.79E-04	GL14_13/E1
S1_18315761	1	18315761	2.79E-04	GL14_13/E1

SNP	Chromosome	Position	P.value	Trait
S1_18315763	1	18315763	2.79E-04	GL14_13/E1
S1_18315778	1	18315778	2.79E-04	GL14_13/E1
S1_63226451	1	63226451	2.79E-04	GL21_14/E2
S1_63226451	1	63226451	2.79E-04	GL21_14/E2
S1_12156535	1	12156535	2.82E-04	GL14_ac
S1_12106664	1	12106664	2.83E-04	GL28_ac
S1_12156535	1	12156535	2.94E-04	GL14_13/E1
S1_12156535	1	12156535	3.04E-04	GL35_ac
S1_12156535	1	12156535	3.10E-04	GL28_ac
S1_12627796	1	12627796	3.16E-04	GL7_ac
S1_77764974	1	77764974	3.36E-04	GL28_14/E2
S1_13095746	1	13095746	3.39E-04	GL21_13/E1
S1_13095759	1	13095759	3.39E-04	GL21_13/E1
S1_12106664	1	12106664	3.58E-04	GL42_13/E1
S1_75427740	1	75427740	3.62E-04	GL35_ac
S1_12564526	1	12564526	3.62E-04	GL28_14/E2
S1_12627796	1	12627796	3.78E-04	GL21_13/E1
S1_12627779	1	12627779	3.89E-04	GL21_13/E1
S1_78485839	1	78485839	3.90E-04	GL49_14/E2
S1_7388314	1	7388314	3.91E-04	GL42_13/E1
S1_78752784	1	78752784	4.04E-04	GL49_14/E2
S1_12156535	1	12156535	4.10E-04	GL49_13/E1
S1_12106664	1	12106664	4.21E-04	GL14_13/E1
S1_12627796	1	12627796	4.35E-04	GL14_ac
S1_14869710	1	14869710	4.37E-04	GL28_13/E1
S1_12106664	1	12106664	4.40E-04	GL49_13/E1
S1_80482739	1	80482739	4.44E-04	GL14_14/E2
S1_80482740	1	80482740	4.44E-04	GL14_14/E2
S1_18315751	1	18315751	4.49E-04	GL28_ac
S1_18315763	1	18315763	4.49E-04	GL28_ac
S1_18315761	1	18315761	4.49E-04	GL28_ac
S1_18315778	1	18315778	4.49E-04	GL28_ac
S1_15084897	1	15084897	4.50E-04	GL35_14/E2
S1_12758577	1	12758577	4.51E-04	GL14_ac
S1_12627779	1	12627779	4.62E-04	GL14_ac
S1_12627779	1	12627779	4.69E-04	GL7_ac
S1_14690005	1	14690005	4.78E-04	GL14_14/E2
S1_12627796	1	12627796	4.99E-04	GL49_13/E1
S1_57111668	1	57111668	5.14E-04	GL35_ac
S1_17818500	1	17818500	5.58E-04	GL7_14/E2
S1_12156535	1	12156535	5.60E-04	GL7_13/E1
S1_12156535	1	12156535	5.61E-04	GL42_13/E1
S1_12122950	1	12122950	5.71E-04	GL7_13/E1
S1_56549114	1	56549114	5.90E-04	GL35_13/E1
S1_56549114	1	56549114	5.90E-04	GL35_13/E1
S1_12122950	1	12122950	5.99E-04	GL14_13/E1
S1_14690013	1	14690013	6.03E-04	GL21_14/E2
S1_14689984	1	14689984	6.03E-04	GL21_14/E2
S1_56225727	1	56225727	6.23E-04	GL21_14/E2
S1_12967667	1	12967667	6.42E-04	GL28_13/E1
S1_12967675	1	12967675	6.42E-04	GL28_13/E1

SNP	Chromosome	Position	P.value	Trait
S1_12967696	1	12967696	6.42E-04	GL28_13/E1
S1_12627796	1	12627796	6.47E-04	GL28_13/E1
S1_16647171	1	16647171	6.51E-04	GL28_13/E1
S1_67392250	1	67392250	6.51E-04	GL42_ac
S1_18315751	1	18315751	6.53E-04	GL21_ac
S1_18315763	1	18315763	6.53E-04	GL21_ac
S1_18315761	1	18315761	6.53E-04	GL21_ac
S1_18315778	1	18315778	6.53E-04	GL21_ac
S1_72205632	1	72205632	6.57E-04	GL14_ac
S1_12758577	1	12758577	6.57E-04	GL14_14/E2
S1_13968190	1	13968190	6.57E-04	GL7_ac
S1_12781217	1	12781217	6.60E-04	GL35_14/E2
S1_7388314	1	7388314	6.66E-04	GL49_13/E1
S1_12781217	1	12781217	6.71E-04	GL28_13/E1
S1_78753233	1	78753233	6.80E-04	GL49_14/E2
S1_78752790	1	78752790	6.80E-04	GL49_14/E2
S1_75427740	1	75427740	6.89E-04	GL28_ac
S1_12106664	1	12106664	7.03E-04	GL7_ac
S1_12967667	1	12967667	7.40E-04	GL7_ac
S1_12967675	1	12967675	7.40E-04	GL7_ac
S1_12967696	1	12967696	7.40E-04	GL7_ac
S1_77764974	1	77764974	7.42E-04	GL21_14/E2
S1_75427740	1	75427740	7.52E-04	GL42_ac
S1_12564526	1	12564526	7.59E-04	GL28_13/E1
S1_15084897	1	15084897	7.84E-04	GL35_ac
S1_11003165	1	11003165	7.96E-04	GL28_14/E2
S1_72205632	1	72205632	8.09E-04	GL21_ac
S1_72940285	1	72940285	8.22E-04	GL42_ac
S1_72940286	1	72940286	8.22E-04	GL42_ac
S1_72940300	1	72940300	8.22E-04	GL42_ac
S1_72940335	1	72940335	8.22E-04	GL42_ac
S1_12627779	1	12627779	8.23E-04	GL49_13/E1
S1_12415971	1	12415971	8.29E-04	GL21_13/E1
S1_56906702	1	56906702	8.31E-04	GL35_13/E1
S1_65668476	1	65668476	8.33E-04	GL35_13/E1
S1_65668486	1	65668486	8.33E-04	GL35_13/E1
S1_56225727	1	56225727	8.39E-04	GL14_14/E2
S1_7388314	1	7388314	8.43E-04	GL35_13/E1
S1_12967667	1	12967667	8.72E-04	GL21_13/E1
S1_12967675	1	12967675	8.72E-04	GL21_13/E1
S1_12967696	1	12967696	8.72E-04	GL21_13/E1
S1_12922040	1	12922040	8.80E-04	GL35_13/E1
S1_57665002	1	57665002	8.83E-04	GL28_13/E1
S1_13095746	1	13095746	9.10E-04	GL28_13/E1
S1_13095759	1	13095759	9.10E-04	GL28_13/E1
S1_63226451	1	63226451	9.22E-04	GL35_ac
S1_63226451	1	63226451	9.22E-04	GL35_ac
S1_13300990	1	13300990	9.37E-04	GL35_ac
S1_12415971	1	12415971	9.47E-04	GL35_13/E1
S1_56906702	1	56906702	9.49E-04	GL49_13/E1
S1_12997445	1	12997445	9.59E-04	GL21_13/E1

SNP	Chromosome	Position	P.value	Trait
S1_12997479	1	12997479	9.59E-04	GL21_13/E1
S1_12627779	1	12627779	9.64E-04	GL28_13/E1
S1_13427114	1	13427114	9.72E-04	GL35_14/E2
S1_31474198	1	31474198	9.82E-04	GL21_14/E2
S1_79545879	1	79545879	9.85E-04	GL28_14/E2
S1_18315751	1	18315751	1.00E-03	GL21_13/E1
S1_18315761	1	18315761	1.00E-03	GL21_13/E1
S1_18315763	1	18315763	1.00E-03	GL21_13/E1
S1_18315778	1	18315778	1.00E-03	GL21_13/E1
S1_12627779	1	12627779	1.02E-03	GL7_13/E1
S1_12758577	1	12758577	1.02E-03	GL21_13/E1
S1_12563731	1	12563731	1.03E-03	GL21_13/E1
S1_12563732	1	12563732	1.03E-03	GL21_13/E1
S1_12563733	1	12563733	1.03E-03	GL21_13/E1
S1_12563734	1	12563734	1.03E-03	GL21_13/E1
S1_12563735	1	12563735	1.03E-03	GL21_13/E1
S1_12563736	1	12563736	1.03E-03	GL21_13/E1
S1_12563738	1	12563738	1.03E-03	GL21_13/E1
S1_12563739	1	12563739	1.03E-03	GL21_13/E1
S1_12563723	1	12563723	1.03E-03	GL21_13/E1
S1_65649103	1	65649103	1.03E-03	GL14_14/E2
S1_18315751	1	18315751	1.03E-03	GL7_ac
S1_18315763	1	18315763	1.03E-03	GL7_ac
S1_18315761	1	18315761	1.03E-03	GL7_ac
S1_18315778	1	18315778	1.03E-03	GL7_ac
S1_12758577	1	12758577	1.05E-03	GL21_ac
S1_57665002	1	57665002	1.06E-03	GL21_ac
S1_18315751	1	18315751	1.06E-03	GL28_13/E1
S1_18315761	1	18315761	1.06E-03	GL28_13/E1
S1_18315763	1	18315763	1.06E-03	GL28_13/E1
S1_18315778	1	18315778	1.06E-03	GL28_13/E1
S1_12564526	1	12564526	1.08E-03	GL21_14/E2
S1_12967667	1	12967667	1.08E-03	GL49_13/E1
S1_12967675	1	12967675	1.08E-03	GL49_13/E1
S1_12967696	1	12967696	1.08E-03	GL49_13/E1
S1_75624748	1	75624748	1.09E-03	GL35_14/E2
S1_75624752	1	75624752	1.09E-03	GL35_14/E2
S1_28195074	1	28195074	1.11E-03	GL35_14/E2
S1_74323695	1	74323695	1.13E-03	GL35_14/E2
S1_74323697	1	74323697	1.13E-03	GL35_14/E2
S1_74323691	1	74323691	1.13E-03	GL35_14/E2
S1_74323696	1	74323696	1.13E-03	GL35_14/E2
S1_12967667	1	12967667	1.13E-03	GL14_13/E1
S1_12967675	1	12967675	1.13E-03	GL14_13/E1
S1_12967696	1	12967696	1.13E-03	GL14_13/E1
S1_19349521	1	19349521	1.14E-03	GL42_14/E2
S1_19349545	1	19349545	1.14E-03	GL42_14/E2
S1_19349551	1	19349551	1.14E-03	GL42_14/E2
S1_79619171	1	79619171	1.14E-03	GL35_14/E2
S1_31474198	1	31474198	1.16E-03	GL14_14/E2
S1_57665002	1	57665002	1.20E-03	GL7_ac

SNP	Chromosome	Position	P.value	Trait
S1_75427740	1	75427740	1.21E-03	GL49_14/E2
S1_13300990	1	13300990	1.22E-03	GL28_ac
S1_12627796	1	12627796	1.24E-03	GL7_13/E1
S1_12968168	1	12968168	1.26E-03	GL21_13/E1
S1_12968211	1	12968211	1.26E-03	GL21_13/E1
S1_71360475	1	71360475	1.27E-03	GL7_ac
S1_12627796	1	12627796	1.27E-03	GL14_13/E1
S1_75427740	1	75427740	1.27E-03	GL35_13/E1
S1_12997445	1	12997445	1.28E-03	GL28_13/E1
S1_12997479	1	12997479	1.28E-03	GL28_13/E1
S1_7388314	1	7388314	1.28E-03	GL28_13/E1
S1_22035471	1	22035471	1.29E-03	GL14_14/E2
S1_72205632	1	72205632	1.31E-03	GL28_ac
S1_12627796	1	12627796	1.31E-03	GL21_ac
S1_74323695	1	74323695	1.32E-03	GL14_ac
S1_74323697	1	74323697	1.32E-03	GL14_ac
S1_74323691	1	74323691	1.32E-03	GL14_ac
S1_74323696	1	74323696	1.32E-03	GL14_ac
S1_12627779	1	12627779	1.34E-03	GL14_13/E1
S1_75427740	1	75427740	1.36E-03	GL14_ac
S1_7388314	1	7388314	1.36E-03	GL21_13/E1
S1_79195214	1	79195214	1.36E-03	GL28_14/E2
S1_12595162	1	12595162	1.37E-03	GL28_13/E1
S1_75427740	1	75427740	1.37E-03	GL49_13/E1
S1_14285571	1	14285571	1.38E-03	GL49_13/E1
S1_14285572	1	14285572	1.38E-03	GL49_13/E1
S1_14285586	1	14285586	1.38E-03	GL49_13/E1
S1_14285587	1	14285587	1.38E-03	GL49_13/E1
S1_12959162	1	12959162	1.38E-03	GL21_ac
S1_12997445	1	12997445	1.38E-03	GL21_ac
S1_12997479	1	12997479	1.38E-03	GL21_ac
S1_12627779	1	12627779	1.38E-03	GL21_ac
S1_12415971	1	12415971	1.40E-03	GL28_13/E1
S1_12564526	1	12564526	1.40E-03	GL35_ac
S1_13968190	1	13968190	1.41E-03	GL14_ac
S1_12596401	1	12596401	1.42E-03	GL21_13/E1
S1_12627796	1	12627796	1.43E-03	GL7_14/E2
S1_79576377	1	79576377	1.43E-03	GL28_14/E2
S1_3547245	1	3547245	1.43E-03	GL49_13/E1
S1_71360475	1	71360475	1.44E-03	GL7_14/E2
S1_10087988	1	10087988	1.44E-03	GL28_13/E1
S1_14876291	1	14876291	1.44E-03	GL7_14/E2
S1_14876303	1	14876303	1.44E-03	GL7_14/E2
S1_14876334	1	14876334	1.44E-03	GL7_14/E2
S1_14869710	1	14869710	1.45E-03	GL7_ac
S1_53772305	1	53772305	1.46E-03	GL35_14/E2
S1_53772311	1	53772311	1.46E-03	GL35_14/E2
S1_16647171	1	16647171	1.48E-03	GL14_13/E1
S1_7222657	1	7222657	1.49E-03	GL7_13/E1
S1_7222659	1	7222659	1.49E-03	GL7_13/E1
S1_7222675	1	7222675	1.49E-03	GL7_13/E1

SNP	Chromosome	Position	P.value	Trait
S1_7222692	1	7222692	1.49E-03	GL7_13/E1
S1_7222713	1	7222713	1.49E-03	GL7_13/E1
S1_13095746	1	13095746	1.50E-03	GL7_13/E1
S1_13095759	1	13095759	1.50E-03	GL7_13/E1
S1_12595162	1	12595162	1.51E-03	GL21_13/E1
S1_13095746	1	13095746	1.51E-03	GL14_13/E1
S1_13095759	1	13095759	1.51E-03	GL14_13/E1
S1_12563731	1	12563731	1.54E-03	GL28_13/E1
S1_12563732	1	12563732	1.54E-03	GL28_13/E1
S1_12563733	1	12563733	1.54E-03	GL28_13/E1
S1_12563734	1	12563734	1.54E-03	GL28_13/E1
S1_12563735	1	12563735	1.54E-03	GL28_13/E1
S1_12563736	1	12563736	1.54E-03	GL28_13/E1
S1_12563738	1	12563738	1.54E-03	GL28_13/E1
S1_12563739	1	12563739	1.54E-03	GL28_13/E1
S1_12563723	1	12563723	1.54E-03	GL28_13/E1
S1_12564742	1	12564742	1.56E-03	GL14_ac
S1_12627739	1	12627739	1.58E-03	GL28_13/E1
S1_14285571	1	14285571	1.60E-03	GL28_13/E1
S1_14285572	1	14285572	1.60E-03	GL28_13/E1
S1_14285586	1	14285586	1.60E-03	GL28_13/E1
S1_14285587	1	14285587	1.60E-03	GL28_13/E1
S1_13968190	1	13968190	1.60E-03	GL7_14/E2
S1_63389970	1	63389970	1.61E-03	GL21_14/E2
S1_2715229	1	2715229	1.62E-03	GL35_13/E1
S1_12781217	1	12781217	1.63E-03	GL42_13/E1
S1_1897884	1	1897884	1.64E-03	GL35_13/E1
S1_12415971	1	12415971	1.65E-03	GL42_13/E1
S1_12627796	1	12627796	1.66E-03	GL35_13/E1
S1_71360475	1	71360475	1.71E-03	GL28_14/E2
S1_12758577	1	12758577	1.73E-03	GL28_13/E1
S1_14876291	1	14876291	1.74E-03	GL14_14/E2
S1_14876303	1	14876303	1.74E-03	GL14_14/E2
S1_14876334	1	14876334	1.74E-03	GL14_14/E2
S1_71936174	1	71936174	1.74E-03	GL42_ac
S1_68697259	1	68697259	1.75E-03	GL42_13/E1
S1_11764852	1	11764852	1.75E-03	GL35_13/E1
S1_10087988	1	10087988	1.76E-03	GL21_13/E1
S1_12627796	1	12627796	1.78E-03	GL35_ac
S1_13300990	1	13300990	1.78E-03	GL21_ac
S1_65649103	1	65649103	1.78E-03	GL7_14/E2
S1_26888112	1	26888112	1.79E-03	GL35_13/E1
S1_12959162	1	12959162	1.80E-03	GL14_ac
S1_26893025	1	26893025	1.80E-03	GL14_14/E2
S1_14285571	1	14285571	1.80E-03	GL21_13/E1
S1_14285572	1	14285572	1.80E-03	GL21_13/E1
S1_14285586	1	14285586	1.80E-03	GL21_13/E1
S1_14285587	1	14285587	1.80E-03	GL21_13/E1
S1_12922040	1	12922040	1.82E-03	GL35_ac
S1_12122950	1	12122950	1.82E-03	GL7_ac
S1_56567618	1	56567618	1.83E-03	GL35_14/E2

SNP	Chromosome	Position	P.value	Trait
S1_72567734	1	72567734	1.83E-03	GL7_ac
S1_72567739	1	72567739	1.83E-03	GL7_ac
S1_72567698	1	72567698	1.83E-03	GL7_ac
S1_72567740	1	72567740	1.83E-03	GL7_ac
S1_28195074	1	28195074	1.85E-03	GL21_14/E2
S1_78753233	1	78753233	1.86E-03	GL49_ac
S1_56074371	1	56074371	1.87E-03	GL49_13/E1
S1_56074384	1	56074384	1.87E-03	GL49_13/E1
S1_14672796	1	14672796	1.90E-03	GL7_14/E2
S1_14285586	1	14285586	1.91E-03	GL35_ac
S1_14285587	1	14285587	1.91E-03	GL35_ac
S1_14285571	1	14285571	1.91E-03	GL35_ac
S1_14285572	1	14285572	1.91E-03	GL35_ac
S1_57676176	1	57676176	1.93E-03	GL14_ac
S1_57676228	1	57676228	1.93E-03	GL14_ac
S1_67567570	1	67567570	1.93E-03	GL42_13/E1
S1_72567734	1	72567734	1.93E-03	GL14_ac
S1_72567739	1	72567739	1.93E-03	GL14_ac
S1_72567698	1	72567698	1.93E-03	GL14_ac
S1_72567740	1	72567740	1.93E-03	GL14_ac
S1_65668476	1	65668476	1.94E-03	GL28_13/E1
S1_65668486	1	65668486	1.94E-03	GL28_13/E1
S1_28195074	1	28195074	1.95E-03	GL14_14/E2
S1_56527271	1	56527271	1.95E-03	GL42_14/E2
S1_13427114	1	13427114	1.95E-03	GL7_14/E2
S1_16390928	1	16390928	1.98E-03	GL7_ac
S1_16390978	1	16390978	1.98E-03	GL7_ac
S1_73866786	1	73866786	1.98E-03	GL35_ac
S1_73866784	1	73866784	1.98E-03	GL35_ac
S1_28195074	1	28195074	1.98E-03	GL28_14/E2
S1_78752784	1	78752784	2.01E-03	GL42_14/E2
S1_27278097	1	27278097	2.02E-03	GL35_ac
S1_57676176	1	57676176	2.02E-03	GL7_14/E2
S1_57676228	1	57676228	2.02E-03	GL7_14/E2
S1_75427740	1	75427740	2.03E-03	GL7_ac
S1_56225727	1	56225727	2.05E-03	GL42_ac
S1_13095759	1	13095759	2.05E-03	GL14_ac
S1_13095746	1	13095746	2.05E-03	GL14_ac
S1_12959333	1	12959333	2.08E-03	GL21_13/E1
S1_12921087	1	12921087	2.08E-03	GL14_14/E2
S1_8931365	1	8931365	2.10E-03	GL42_14/E2
S1_14285586	1	14285586	2.11E-03	GL21_ac
S1_14285587	1	14285587	2.11E-03	GL21_ac
S1_14285571	1	14285571	2.11E-03	GL21_ac
S1_14285572	1	14285572	2.11E-03	GL21_ac
S1_12627796	1	12627796	2.12E-03	GL28_ac
S1_78036426	1	78036426	2.12E-03	GL28_14/E2
S1_50451128	1	50451128	2.12E-03	GL14_13/E1
S1_12997445	1	12997445	2.13E-03	GL14_ac
S1_12997479	1	12997479	2.13E-03	GL14_ac
S1_31474198	1	31474198	2.14E-03	GL28_14/E2

SNP	Chromosome	Position	P.value	Trait
S1_12627779	1	12627779	2.14E-03	GL35_ac
S1_11686529	1	11686529	2.16E-03	GL35_13/E1
S1_49256106	1	49256106	2.18E-03	GL35_14/E2
S1_55062451	1	55062451	2.20E-03	GL42_ac
S1_75427740	1	75427740	2.21E-03	GL42_13/E1
S1_29334826	1	29334826	2.21E-03	GL7_ac
S1_15084897	1	15084897	2.23E-03	GL7_ac
S1_17757376	1	17757376	2.24E-03	GL42_13/E1
S1_12165733	1	12165733	2.26E-03	GL49_13/E1
S1_7222657	1	7222657	2.27E-03	GL14_13/E1
S1_7222659	1	7222659	2.27E-03	GL14_13/E1
S1_7222675	1	7222675	2.27E-03	GL14_13/E1
S1_7222692	1	7222692	2.27E-03	GL14_13/E1
S1_7222713	1	7222713	2.27E-03	GL14_13/E1
S1_75427740	1	75427740	2.27E-03	GL21_14/E2
S1_12596401	1	12596401	2.27E-03	GL28_13/E1
S1_14512256	1	14512256	2.28E-03	GL35_14/E2
S1_12921087	1	12921087	2.29E-03	GL28_14/E2
S1_12415971	1	12415971	2.29E-03	GL14_13/E1
S1_12627739	1	12627739	2.30E-03	GL35_13/E1
S1_14162189	1	14162189	2.30E-03	GL49_13/E1
S1_11686529	1	11686529	2.31E-03	GL42_13/E1
S1_12968198	1	12968198	2.32E-03	GL21_13/E1
S1_56549114	1	56549114	2.32E-03	GL42_13/E1
S1_56549114	1	56549114	2.32E-03	GL42_13/E1
S1_12967667	1	12967667	2.33E-03	GL28_ac
S1_12967675	1	12967675	2.33E-03	GL28_ac
S1_12967696	1	12967696	2.33E-03	GL28_ac
S1_76661673	1	76661673	2.35E-03	GL42_14/E2
S1_76661685	1	76661685	2.35E-03	GL42_14/E2
S1_12627796	1	12627796	2.38E-03	GL14_14/E2
S1_14672796	1	14672796	2.38E-03	GL7_ac
S1_57665002	1	57665002	2.38E-03	GL28_ac
S1_12122950	1	12122950	2.39E-03	GL21_13/E1
S1_63226451	1	63226451	2.41E-03	GL14_14/E2
S1_63226451	1	63226451	2.41E-03	GL14_14/E2
S1_26888112	1	26888112	2.41E-03	GL35_ac
S1_15084897	1	15084897	2.42E-03	GL28_13/E1
S1_57111668	1	57111668	2.43E-03	GL42_ac
S1_14690013	1	14690013	2.43E-03	GL14_14/E2
S1_14689984	1	14689984	2.43E-03	GL14_14/E2
S1_12758577	1	12758577	2.43E-03	GL28_ac
S1_12627779	1	12627779	2.45E-03	GL7_14/E2
S1_14285586	1	14285586	2.46E-03	GL28_ac
S1_14285587	1	14285587	2.46E-03	GL28_ac
S1_14285571	1	14285571	2.46E-03	GL28_ac
S1_14285572	1	14285572	2.46E-03	GL28_ac
S1_67567570	1	67567570	2.47E-03	GL35_13/E1
S1_16647171	1	16647171	2.48E-03	GL21_13/E1
S1_72205632	1	72205632	2.49E-03	GL21_13/E1
S1_72205632	1	72205632	2.49E-03	GL49_13/E1

SNP	Chromosome	Position	P.value	Trait
S1_72205632	1	72205632	2.50E-03	GL14_13/E1
S1_3464574	1	3464574	2.51E-03	GL21_14/E2
S1_3464575	1	3464575	2.51E-03	GL21_14/E2
S1_3464576	1	3464576	2.51E-03	GL21_14/E2
S1_3464577	1	3464577	2.51E-03	GL21_14/E2
S1_48835914	1	48835914	2.53E-03	GL49_13/E1
S1_48835921	1	48835921	2.53E-03	GL49_13/E1
S1_48835922	1	48835922	2.53E-03	GL49_13/E1
S1_67950145	1	67950145	2.53E-03	GL28_ac
S1_76661673	1	76661673	2.54E-03	GL49_14/E2
S1_76661685	1	76661685	2.54E-03	GL49_14/E2
S1_15084897	1	15084897	2.56E-03	GL28_ac
S1_12415971	1	12415971	2.59E-03	GL7_13/E1
S1_49415027	1	49415027	2.59E-03	GL21_14/E2
S1_12921087	1	12921087	2.61E-03	GL14_ac
S1_78715461	1	78715461	2.62E-03	GL42_14/E2
S1_12781217	1	12781217	2.65E-03	GL7_ac
S1_2715229	1	2715229	2.65E-03	GL35_ac
S1_14870012	1	14870012	2.67E-03	GL14_ac
S1_7576799	1	7576799	2.67E-03	GL14_14/E2
S1_56906702	1	56906702	2.68E-03	GL35_ac
S1_12627796	1	12627796	2.69E-03	GL42_13/E1
S1_71606027	1	71606027	2.69E-03	GL49_ac
S1_71606019	1	71606019	2.69E-03	GL49_ac
S1_12122950	1	12122950	2.69E-03	GL28_13/E1
S1_12781217	1	12781217	2.72E-03	GL42_ac
S1_72938515	1	72938515	2.73E-03	GL42_ac
S1_72938517	1	72938517	2.73E-03	GL42_ac
S1_12627739	1	12627739	2.73E-03	GL28_ac
S1_28470926	1	28470926	2.73E-03	GL7_14/E2
S1_12968168	1	12968168	2.73E-03	GL21_ac
S1_12968211	1	12968211	2.73E-03	GL21_ac
S1_12564526	1	12564526	2.73E-03	GL7_13/E1
S1_12564526	1	12564526	2.75E-03	GL14_13/E1
S1_71731573	1	71731573	2.76E-03	GL14_14/E2
S1_57874569	1	57874569	2.76E-03	GL7_14/E2
S1_10462682	1	10462682	2.77E-03	GL14_14/E2
S1_11151142	1	11151142	2.77E-03	GL49_14/E2
S1_11151144	1	11151144	2.77E-03	GL49_14/E2
S1_15084897	1	15084897	2.78E-03	GL21_13/E1
S1_71601826	1	71601826	2.78E-03	GL21_14/E2
S1_12227113	1	12227113	2.78E-03	GL28_13/E1
S1_12227115	1	12227115	2.78E-03	GL28_13/E1
S1_66652233	1	66652233	2.79E-03	GL42_ac
S1_12106664	1	12106664	2.82E-03	GL42_ac
S1_56735617	1	56735617	2.84E-03	GL42_ac
S1_56735641	1	56735641	2.84E-03	GL42_ac
S1_12627779	1	12627779	2.85E-03	GL35_13/E1
S1_14876291	1	14876291	2.85E-03	GL7_ac
S1_14876303	1	14876303	2.85E-03	GL7_ac
S1_14876334	1	14876334	2.85E-03	GL7_ac

SNP	Chromosome	Position	P.value	Trait
S1_12030980	1	12030980	2.86E-03	GL7_ac
S1_75427740	1	75427740	2.87E-03	GL7_13/E1
S1_50451128	1	50451128	2.88E-03	GL7_13/E1
S1_65649103	1	65649103	2.90E-03	GL28_14/E2
S1_13096036	1	13096036	2.91E-03	GL14_14/E2
S1_66786951	1	66786951	2.92E-03	GL28_14/E2
S1_12968168	1	12968168	2.92E-03	GL28_13/E1
S1_12968211	1	12968211	2.92E-03	GL28_13/E1
S1_14876291	1	14876291	2.92E-03	GL28_14/E2
S1_14876303	1	14876303	2.92E-03	GL28_14/E2
S1_14876334	1	14876334	2.92E-03	GL28_14/E2
S1_16647171	1	16647171	2.94E-03	GL42_13/E1
S1_12967667	1	12967667	2.96E-03	GL14_ac
S1_12967675	1	12967675	2.96E-03	GL14_ac
S1_12967696	1	12967696	2.96E-03	GL14_ac
S1_15084897	1	15084897	2.96E-03	GL21_ac
S1_12968198	1	12968198	2.98E-03	GL21_ac
S1_12627739	1	12627739	2.99E-03	GL21_13/E1
S1_13300990	1	13300990	3.00E-03	GL7_ac
S1_14854881	1	14854881	3.00E-03	GL35_14/E2
S1_14448619	1	14448619	3.00E-03	GL49_ac
S1_14448621	1	14448621	3.00E-03	GL49_ac
S1_14448625	1	14448625	3.00E-03	GL49_ac
S1_14448626	1	14448626	3.00E-03	GL49_ac
S1_14448627	1	14448627	3.00E-03	GL49_ac
S1_14448628	1	14448628	3.00E-03	GL49_ac
S1_14448631	1	14448631	3.00E-03	GL49_ac
S1_14448632	1	14448632	3.00E-03	GL49_ac
S1_14448634	1	14448634	3.00E-03	GL49_ac
S1_68578501	1	68578501	3.01E-03	GL28_14/E2
S1_79545879	1	79545879	3.02E-03	GL21_14/E2
S1_14869710	1	14869710	3.02E-03	GL21_13/E1
S1_65668476	1	65668476	3.03E-03	GL42_13/E1
S1_65668486	1	65668486	3.03E-03	GL42_13/E1
S1_74876549	1	74876549	3.04E-03	GL35_ac
S1_56906702	1	56906702	3.06E-03	GL42_13/E1
S1_75427740	1	75427740	3.07E-03	GL14_13/E1
S1_11764852	1	11764852	3.07E-03	GL35_ac
S1_12596401	1	12596401	3.07E-03	GL21_ac
S1_12122950	1	12122950	3.07E-03	GL21_ac
S1_16647171	1	16647171	3.07E-03	GL14_ac
S1_71614848	1	71614848	3.10E-03	GL42_ac
S1_12415971	1	12415971	3.12E-03	GL21_ac
S1_14162189	1	14162189	3.12E-03	GL35_13/E1
S1_12781217	1	12781217	3.13E-03	GL49_13/E1
S1_18315751	1	18315751	3.13E-03	GL35_13/E1
S1_18315761	1	18315761	3.13E-03	GL35_13/E1
S1_18315763	1	18315763	3.13E-03	GL35_13/E1
S1_18315778	1	18315778	3.13E-03	GL35_13/E1
S1_63543649	1	63543649	3.14E-03	GL28_14/E2
S1_13300990	1	13300990	3.15E-03	GL21_13/E1

SNP	Chromosome	Position	P.value	Trait
S1_13095759	1	13095759	3.16E-03	GL21_ac
S1_13095746	1	13095746	3.16E-03	GL21_ac
S1_13427114	1	13427114	3.17E-03	GL28_14/E2
S1_13384592	1	13384592	3.18E-03	GL28_14/E2
S1_12997445	1	12997445	3.20E-03	GL35_ac
S1_12997479	1	12997479	3.20E-03	GL35_ac
S1_65649103	1	65649103	3.20E-03	GL49_ac
S1_66004503	1	66004503	3.21E-03	GL21_14/E2
S1_12627779	1	12627779	3.21E-03	GL14_14/E2
S1_78585090	1	78585090	3.22E-03	GL35_14/E2
S1_13188261	1	13188261	3.23E-03	GL28_ac
S1_12922040	1	12922040	3.25E-03	GL28_13/E1
S1_7782167	1	7782167	3.29E-03	GL14_14/E2
S1_11625328	1	11625328	3.29E-03	GL35_ac
S1_49256106	1	49256106	3.30E-03	GL42_14/E2
S1_12106664	1	12106664	3.30E-03	GL14_14/E2
S1_14869710	1	14869710	3.30E-03	GL14_ac
S1_67950145	1	67950145	3.32E-03	GL42_ac
S1_7552102	1	7552102	3.34E-03	GL35_13/E1
S1_14869710	1	14869710	3.35E-03	GL28_ac
S1_14854881	1	14854881	3.35E-03	GL14_14/E2
S1_73644317	1	73644317	3.36E-03	GL49_14/E2
S1_73644284	1	73644284	3.36E-03	GL49_14/E2
S1_73644308	1	73644308	3.36E-03	GL49_14/E2
S1_14462377	1	14462377	3.37E-03	GL35_14/E2
S1_14462383	1	14462383	3.37E-03	GL35_14/E2
S1_77343688	1	77343688	3.37E-03	GL49_14/E2
S1_77343694	1	77343694	3.37E-03	GL49_14/E2
S1_77343698	1	77343698	3.37E-03	GL49_14/E2
S1_77343689	1	77343689	3.37E-03	GL49_14/E2
S1_77343690	1	77343690	3.37E-03	GL49_14/E2
S1_77343693	1	77343693	3.37E-03	GL49_14/E2
S1_77343695	1	77343695	3.37E-03	GL49_14/E2
S1_77343696	1	77343696	3.37E-03	GL49_14/E2
S1_77343699	1	77343699	3.37E-03	GL49_14/E2
S1_57676176	1	57676176	3.38E-03	GL21_14/E2
S1_57676228	1	57676228	3.38E-03	GL21_14/E2
S1_12781217	1	12781217	3.39E-03	GL14_13/E1
S1_13095746	1	13095746	3.40E-03	GL49_13/E1
S1_13095759	1	13095759	3.40E-03	GL49_13/E1
S1_17106086	1	17106086	3.40E-03	GL7_ac
S1_12959162	1	12959162	3.40E-03	GL21_13/E1
S1_71601826	1	71601826	3.41E-03	GL14_14/E2
S1_57596308	1	57596308	3.42E-03	GL35_13/E1
S1_12165733	1	12165733	3.43E-03	GL28_13/E1
S1_60523410	1	60523410	3.44E-03	GL42_ac
S1_70551787	1	70551787	3.45E-03	GL7_14/E2
S1_12627739	1	12627739	3.47E-03	GL21_ac
S1_55923588	1	55923588	3.47E-03	GL28_14/E2
S1_63672052	1	63672052	3.49E-03	GL21_14/E2
S1_19184682	1	19184682	3.49E-03	GL14_14/E2

SNP	Chromosome	Position	P.value	Trait
S1_19184686	1	19184686	3.49E-03	GL14_14/E2
S1_19184687	1	19184687	3.49E-03	GL14_14/E2
S1_55062451	1	55062451	3.49E-03	GL49_ac
S1_71614848	1	71614848	3.50E-03	GL42_14/E2
S1_12921087	1	12921087	3.52E-03	GL7_14/E2
S1_65668476	1	65668476	3.52E-03	GL35_ac
S1_65668486	1	65668486	3.52E-03	GL35_ac
S1_56225727	1	56225727	3.53E-03	GL35_14/E2
S1_12997445	1	12997445	3.53E-03	GL28_ac
S1_12997479	1	12997479	3.53E-03	GL28_ac
S1_16647171	1	16647171	3.53E-03	GL7_13/E1
S1_10739071	1	10739071	3.53E-03	GL49_ac
S1_14420980	1	14420980	3.54E-03	GL35_14/E2
S1_14420995	1	14420995	3.54E-03	GL35_14/E2
S1_12967667	1	12967667	3.54E-03	GL35_13/E1
S1_12967675	1	12967675	3.54E-03	GL35_13/E1
S1_12967696	1	12967696	3.54E-03	GL35_13/E1
S1_15084897	1	15084897	3.54E-03	GL7_14/E2
S1_57596308	1	57596308	3.55E-03	GL21_13/E1
S1_17757376	1	17757376	3.55E-03	GL35_13/E1
S1_12106664	1	12106664	3.56E-03	GL35_14/E2
S1_71936174	1	71936174	3.56E-03	GL35_ac
S1_12627779	1	12627779	3.60E-03	GL28_ac
S1_14285586	1	14285586	3.60E-03	GL14_ac
S1_14285587	1	14285587	3.60E-03	GL14_ac
S1_14285571	1	14285571	3.60E-03	GL14_ac
S1_14285572	1	14285572	3.60E-03	GL14_ac
S1_11786106	1	11786106	3.61E-03	GL14_14/E2
S1_12165733	1	12165733	3.62E-03	GL14_13/E1
S1_70551787	1	70551787	3.63E-03	GL7_ac
S1_12967667	1	12967667	3.64E-03	GL7_13/E1
S1_12967675	1	12967675	3.64E-03	GL7_13/E1
S1_12967696	1	12967696	3.64E-03	GL7_13/E1
S1_14869710	1	14869710	3.67E-03	GL35_ac
S1_66991835	1	66991835	3.68E-03	GL21_14/E2
S1_17106117	1	17106117	3.69E-03	GL7_ac
S1_71731573	1	71731573	3.69E-03	GL21_ac
S1_67392250	1	67392250	3.70E-03	GL35_ac
S1_12968216	1	12968216	3.73E-03	GL21_13/E1
S1_59982178	1	59982178	3.74E-03	GL7_14/E2
S1_59985155	1	59985155	3.74E-03	GL42_14/E2
S1_14869710	1	14869710	3.75E-03	GL21_ac
S1_71174651	1	71174651	3.76E-03	GL7_14/E2
S1_12030980	1	12030980	3.77E-03	GL7_13/E1
S1_51240322	1	51240322	3.77E-03	GL7_13/E1
S1_71731573	1	71731573	3.77E-03	GL14_ac
S1_12967667	1	12967667	3.80E-03	GL21_ac
S1_12967675	1	12967675	3.80E-03	GL21_ac
S1_12967696	1	12967696	3.80E-03	GL21_ac
S1_75624748	1	75624748	3.80E-03	GL14_14/E2
S1_75624752	1	75624752	3.80E-03	GL14_14/E2

SNP	Chromosome	Position	P.value	Trait
S1_13188261	1	13188261	3.80E-03	GL21_ac
S1_12338566	1	12338566	3.80E-03	GL28_ac
S1_12156535	1	12156535	3.81E-03	GL42_ac
S1_12122950	1	12122950	3.82E-03	GL28_ac
S1_7222753	1	7222753	3.83E-03	GL14_14/E2
S1_61353815	1	61353815	3.83E-03	GL14_14/E2
S1_13095746	1	13095746	3.84E-03	GL35_13/E1
S1_13095759	1	13095759	3.84E-03	GL35_13/E1
S1_57202401	1	57202401	3.84E-03	GL35_13/E1
S1_79582017	1	79582017	3.87E-03	GL42_14/E2
S1_12122950	1	12122950	3.88E-03	GL49_13/E1
S1_50894412	1	50894412	3.88E-03	GL14_14/E2
S1_11625328	1	11625328	3.89E-03	GL28_13/E1
S1_11003138	1	11003138	3.90E-03	GL28_14/E2
S1_16647171	1	16647171	3.91E-03	GL35_13/E1
S1_56906702	1	56906702	3.91E-03	GL28_13/E1
S1_79619171	1	79619171	3.92E-03	GL28_14/E2
S1_65649103	1	65649103	3.94E-03	GL42_ac
S1_56225727	1	56225727	3.94E-03	GL28_14/E2
S1_10565425	1	10565425	3.95E-03	GL28_14/E2
S1_12564742	1	12564742	3.95E-03	GL35_14/E2
S1_57740678	1	57740678	3.96E-03	GL7_13/E1
S1_2717923	1	2717923	3.96E-03	GL35_13/E1
S1_55062453	1	55062453	3.98E-03	GL49_ac
S1_10087988	1	10087988	3.98E-03	GL42_13/E1
S1_56225727	1	56225727	3.98E-03	GL35_ac
S1_11625328	1	11625328	3.99E-03	GL21_13/E1
S1_55831986	1	55831986	4.00E-03	GL49_14/E2
S1_12415971	1	12415971	4.01E-03	GL14_ac
S1_14869710	1	14869710	4.01E-03	GL14_13/E1
S1_49256106	1	49256106	4.02E-03	GL14_14/E2
S1_74876553	1	74876553	4.02E-03	GL35_ac
S1_74876556	1	74876556	4.02E-03	GL35_ac
S1_72205632	1	72205632	4.04E-03	GL28_13/E1
S1_67950145	1	67950145	4.06E-03	GL21_13/E1
S1_80482739	1	80482739	4.07E-03	GL42_14/E2
S1_80482740	1	80482740	4.07E-03	GL42_14/E2
S1_63389994	1	63389994	4.07E-03	GL42_ac
S1_5666314	1	5666314	4.09E-03	GL42_13/E1
S1_13095759	1	13095759	4.09E-03	GL28_ac
S1_13095746	1	13095746	4.09E-03	GL28_ac
S1_7388314	1	7388314	4.10E-03	GL35_ac
S1_78752790	1	78752790	4.11E-03	GL42_14/E2
S1_71511929	1	71511929	4.11E-03	GL7_13/E1
S1_2715229	1	2715229	4.14E-03	GL28_13/E1
S1_67950145	1	67950145	4.16E-03	GL28_13/E1
S1_72940285	1	72940285	4.18E-03	GL28_ac
S1_72940286	1	72940286	4.18E-03	GL28_ac
S1_72940300	1	72940300	4.18E-03	GL28_ac
S1_72940335	1	72940335	4.18E-03	GL28_ac
S1_79545879	1	79545879	4.18E-03	GL7_14/E2

SNP	Chromosome	Position	P.value	Trait
S1_14864964	1	14864964	4.18E-03	GL21_13/E1
S1_14864972	1	14864972	4.18E-03	GL21_13/E1
S1_12095456	1	12095456	4.18E-03	GL28_14/E2
S1_19180066	1	19180066	4.19E-03	GL7_ac
S1_67485484	1	67485484	4.20E-03	GL7_14/E2
S1_67485490	1	67485490	4.20E-03	GL7_14/E2
S1_67485491	1	67485491	4.20E-03	GL7_14/E2
S1_17532285	1	17532285	4.21E-03	GL28_13/E1
S1_57596308	1	57596308	4.21E-03	GL28_14/E2
S1_75378360	1	75378360	4.24E-03	GL49_14/E2
S1_72205632	1	72205632	4.25E-03	GL7_13/E1
S1_72940285	1	72940285	4.26E-03	GL35_ac
S1_72940286	1	72940286	4.26E-03	GL35_ac
S1_72940300	1	72940300	4.26E-03	GL35_ac
S1_72940335	1	72940335	4.26E-03	GL35_ac
S1_67607594	1	67607594	4.26E-03	GL14_14/E2
S1_15908614	1	15908614	4.27E-03	GL35_14/E2
S1_12165733	1	12165733	4.30E-03	GL21_13/E1
S1_4063045	1	4063045	4.30E-03	GL35_14/E2
S1_59985148	1	59985148	4.30E-03	GL49_14/E2
S1_59985195	1	59985195	4.30E-03	GL49_14/E2
S1_15015362	1	15015362	4.30E-03	GL7_ac
S1_15015363	1	15015363	4.30E-03	GL7_ac
S1_15015351	1	15015351	4.30E-03	GL7_ac
S1_27278097	1	27278097	4.33E-03	GL35_13/E1
S1_7552102	1	7552102	4.37E-03	GL42_13/E1
S1_14876291	1	14876291	4.38E-03	GL14_ac
S1_14876303	1	14876303	4.38E-03	GL14_ac
S1_14876334	1	14876334	4.38E-03	GL14_ac
S1_12563731	1	12563731	4.38E-03	GL7_13/E1
S1_12563732	1	12563732	4.38E-03	GL7_13/E1
S1_12563733	1	12563733	4.38E-03	GL7_13/E1
S1_12563734	1	12563734	4.38E-03	GL7_13/E1
S1_12563735	1	12563735	4.38E-03	GL7_13/E1
S1_12563736	1	12563736	4.38E-03	GL7_13/E1
S1_12563738	1	12563738	4.38E-03	GL7_13/E1
S1_12563739	1	12563739	4.38E-03	GL7_13/E1
S1_12563723	1	12563723	4.38E-03	GL7_13/E1
S1_14285586	1	14285586	4.39E-03	GL35_14/E2
S1_14285587	1	14285587	4.39E-03	GL35_14/E2
S1_14285571	1	14285571	4.39E-03	GL35_14/E2
S1_14285572	1	14285572	4.39E-03	GL35_14/E2
S1_27278097	1	27278097	4.39E-03	GL28_ac
S1_18315751	1	18315751	4.40E-03	GL49_13/E1
S1_18315761	1	18315761	4.40E-03	GL49_13/E1
S1_18315763	1	18315763	4.40E-03	GL49_13/E1
S1_18315778	1	18315778	4.40E-03	GL49_13/E1
S1_9629161	1	9629161	4.40E-03	GL7_14/E2
S1_12415971	1	12415971	4.40E-03	GL7_ac
S1_15880752	1	15880752	4.42E-03	GL28_14/E2
S1_15880765	1	15880765	4.42E-03	GL28_14/E2

SNP	Chromosome	Position	P.value	Trait
S1_15880754	1	15880754	4.42E-03	GL28_14/E2
S1_15880763	1	15880763	4.42E-03	GL28_14/E2
S1_15880766	1	15880766	4.42E-03	GL28_14/E2
S1_14420980	1	14420980	4.45E-03	GL28_13/E1
S1_14420995	1	14420995	4.45E-03	GL28_13/E1
S1_80482739	1	80482739	4.45E-03	GL35_ac
S1_80482740	1	80482740	4.45E-03	GL35_ac
S1_72171208	1	72171208	4.46E-03	GL28_13/E1
S1_72171210	1	72171210	4.46E-03	GL28_13/E1
S1_56906702	1	56906702	4.46E-03	GL14_13/E1
S1_20258213	1	20258213	4.46E-03	GL35_14/E2
S1_72430279	1	72430279	4.47E-03	GL42_ac
S1_49924192	1	49924192	4.48E-03	GL28_14/E2
S1_13300990	1	13300990	4.48E-03	GL28_13/E1
S1_12932509	1	12932509	4.48E-03	GL49_13/E1
S1_12563731	1	12563731	4.52E-03	GL42_13/E1
S1_12563732	1	12563732	4.52E-03	GL42_13/E1
S1_12563733	1	12563733	4.52E-03	GL42_13/E1
S1_12563734	1	12563734	4.52E-03	GL42_13/E1
S1_12563735	1	12563735	4.52E-03	GL42_13/E1
S1_12563736	1	12563736	4.52E-03	GL42_13/E1
S1_12563738	1	12563738	4.52E-03	GL42_13/E1
S1_12563739	1	12563739	4.52E-03	GL42_13/E1
S1_12563723	1	12563723	4.52E-03	GL42_13/E1
S1_56549114	1	56549114	4.54E-03	GL49_13/E1
S1_56549114	1	56549114	4.54E-03	GL49_13/E1
S1_73866784	1	73866784	4.54E-03	GL35_13/E1
S1_73866786	1	73866786	4.54E-03	GL35_13/E1
S1_57419605	1	57419605	4.55E-03	GL49_13/E1
S1_11686529	1	11686529	4.57E-03	GL7_13/E1
S1_55062451	1	55062451	4.57E-03	GL42_14/E2
S1_10087988	1	10087988	4.58E-03	GL49_13/E1
S1_59985155	1	59985155	4.60E-03	GL49_14/E2
S1_12338566	1	12338566	4.61E-03	GL21_ac
S1_19184682	1	19184682	4.62E-03	GL21_14/E2
S1_19184686	1	19184686	4.62E-03	GL21_14/E2
S1_19184687	1	19184687	4.62E-03	GL21_14/E2
S1_80482739	1	80482739	4.63E-03	GL35_14/E2
S1_80482740	1	80482740	4.63E-03	GL35_14/E2
S1_12596401	1	12596401	4.64E-03	GL28_ac
S1_79638265	1	79638265	4.64E-03	GL42_13/E1
S1_67567570	1	67567570	4.65E-03	GL28_13/E1
S1_5666314	1	5666314	4.67E-03	GL28_13/E1
S1_1215475	1	1215475	4.69E-03	GL49_13/E1
S1_12922040	1	12922040	4.70E-03	GL42_13/E1
S1_16390928	1	16390928	4.73E-03	GL7_14/E2
S1_16390978	1	16390978	4.73E-03	GL7_14/E2
S1_2717923	1	2717923	4.74E-03	GL28_13/E1
S1_79540230	1	79540230	4.75E-03	GL28_14/E2
S1_12249706	1	12249706	4.76E-03	GL35_13/E1
S1_7769551	1	7769551	4.77E-03	GL21_14/E2

SNP	Chromosome	Position	P.value	Trait
S1_57665002	1	57665002	4.79E-03	GL14_ac
S1_12122950	1	12122950	4.79E-03	GL35_ac
S1_78161599	1	78161599	4.80E-03	GL49_14/E2
S1_74323695	1	74323695	4.81E-03	GL14_14/E2
S1_74323697	1	74323697	4.81E-03	GL14_14/E2
S1_74323691	1	74323691	4.81E-03	GL14_14/E2
S1_74323696	1	74323696	4.81E-03	GL14_14/E2
S1_8931365	1	8931365	4.84E-03	GL28_14/E2
S1_18971221	1	18971221	4.84E-03	GL7_14/E2
S1_68772784	1	68772784	4.84E-03	GL42_ac
S1_68772784	1	68772784	4.84E-03	GL42_ac
S1_68772784	1	68772784	4.84E-03	GL42_ac
S1_79576377	1	79576377	4.85E-03	GL21_14/E2
S1_50830279	1	50830279	4.85E-03	GL21_13/E1
S1_63965855	1	63965855	4.85E-03	GL21_14/E2
S1_12165733	1	12165733	4.86E-03	GL21_ac
S1_12160253	1	12160253	4.87E-03	GL35_ac
S1_12160257	1	12160257	4.87E-03	GL35_ac
S1_12165733	1	12165733	4.87E-03	GL7_13/E1
S1_63354974	1	63354974	4.87E-03	GL35_ac
S1_12627739	1	12627739	4.87E-03	GL42_13/E1
S1_13300990	1	13300990	4.89E-03	GL35_13/E1
S1_71360475	1	71360475	4.89E-03	GL7_13/E1
S1_73413882	1	73413882	4.92E-03	GL42_ac
S1_13376221	1	13376221	4.92E-03	GL7_14/E2
S1_12249706	1	12249706	4.92E-03	GL42_13/E1
S1_50830279	1	50830279	4.93E-03	GL14_13/E1
S1_57665002	1	57665002	4.94E-03	GL35_13/E1
S1_12967786	1	12967786	4.94E-03	GL21_ac
S1_56906702	1	56906702	4.95E-03	GL21_13/E1
S1_80576324	1	80576324	4.96E-03	GL28_14/E2
S1_10793791	1	10793791	4.97E-03	GL42_13/E1
S1_26596842	1	26596842	4.98E-03	GL35_14/E2
S1_79545879	1	79545879	4.98E-03	GL42_14/E2
S1_78180778	1	78180778	5.00E-03	GL49_14/E2
S1_78180781	1	78180781	5.00E-03	GL49_14/E2
S1_78180782	1	78180782	5.00E-03	GL49_14/E2
S1_71511929	1	71511929	5.02E-03	GL7_ac
S1_13300990	1	13300990	5.02E-03	GL14_ac
S1_12564742	1	12564742	5.03E-03	GL21_ac
S1_14162189	1	14162189	5.04E-03	GL28_13/E1
S1_12959333	1	12959333	5.04E-03	GL14_ac
S1_8998674	1	8998674	5.05E-03	GL28_ac
S1_8998667	1	8998667	5.05E-03	GL28_ac
S1_79306363	1	79306363	5.05E-03	GL14_14/E2
S1_12564742	1	12564742	5.07E-03	GL35_ac
S1_67950145	1	67950145	5.07E-03	GL35_13/E1
S1_71511878	1	71511878	5.08E-03	GL7_ac
S1_12106664	1	12106664	5.09E-03	GL21_14/E2
S1_59558610	1	59558610	5.11E-03	GL21_13/E1
S1_57202401	1	57202401	5.11E-03	GL42_ac

SNP	Chromosome	Position	P.value	Trait
S1_12959333	1	12959333	5.11E-03	GL28_13/E1
S1_74323695	1	74323695	5.13E-03	GL35_ac
S1_74323697	1	74323697	5.13E-03	GL35_ac
S1_74323691	1	74323691	5.13E-03	GL35_ac
S1_74323696	1	74323696	5.13E-03	GL35_ac
S1_13102046	1	13102046	5.14E-03	GL21_13/E1
S1_56567618	1	56567618	5.14E-03	GL42_14/E2
S1_19180066	1	19180066	5.14E-03	GL14_13/E1
S1_12757772	1	12757772	5.15E-03	GL14_ac
S1_12227113	1	12227113	5.15E-03	GL14_13/E1
S1_12227115	1	12227115	5.15E-03	GL14_13/E1
S1_79149961	1	79149961	5.17E-03	GL49_14/E2
S1_12959333	1	12959333	5.19E-03	GL21_ac
S1_63672014	1	63672014	5.20E-03	GL21_14/E2
S1_67332284	1	67332284	5.22E-03	GL49_14/E2
S1_79149958	1	79149958	5.22E-03	GL49_14/E2
S1_71553878	1	71553878	5.23E-03	GL42_ac
S1_12997445	1	12997445	5.24E-03	GL35_14/E2
S1_12997479	1	12997479	5.24E-03	GL35_14/E2
S1_79638265	1	79638265	5.25E-03	GL35_13/E1
S1_72205632	1	72205632	5.25E-03	GL42_13/E1
S1_12758577	1	12758577	5.26E-03	GL7_13/E1
S1_12968216	1	12968216	5.26E-03	GL14_ac
S1_13376646	1	13376646	5.28E-03	GL21_14/E2
S1_56549114	1	56549114	5.28E-03	GL28_13/E1
S1_56549114	1	56549114	5.28E-03	GL28_13/E1
S1_14672796	1	14672796	5.29E-03	GL14_14/E2
S1_12030980	1	12030980	5.30E-03	GL35_ac
S1_12781217	1	12781217	5.30E-03	GL49_ac
S1_12968198	1	12968198	5.32E-03	GL28_13/E1
S1_12564526	1	12564526	5.33E-03	GL35_13/E1
S1_17757376	1	17757376	5.33E-03	GL28_13/E1
S1_56327742	1	56327742	5.35E-03	GL35_14/E2
S1_56327744	1	56327744	5.35E-03	GL35_14/E2
S1_13300990	1	13300990	5.39E-03	GL7_14/E2
S1_60519774	1	60519774	5.40E-03	GL28_14/E2
S1_79149949	1	79149949	5.40E-03	GL49_14/E2
S1_57111668	1	57111668	5.41E-03	GL42_14/E2
S1_12227113	1	12227113	5.41E-03	GL21_13/E1
S1_12227115	1	12227115	5.41E-03	GL21_13/E1
S1_12249706	1	12249706	5.43E-03	GL28_13/E1
S1_50390544	1	50390544	5.43E-03	GL49_14/E2
S1_12921087	1	12921087	5.44E-03	GL21_ac
S1_14869710	1	14869710	5.44E-03	GL7_13/E1
S1_74359940	1	74359940	5.47E-03	GL49_13/E1
S1_72940285	1	72940285	5.49E-03	GL21_13/E1
S1_72940286	1	72940286	5.49E-03	GL21_13/E1
S1_72940300	1	72940300	5.49E-03	GL21_13/E1
S1_72940335	1	72940335	5.49E-03	GL21_13/E1
S1_57383718	1	57383718	5.50E-03	GL7_13/E1
S1_68772784	1	68772784	5.51E-03	GL42_14/E2

SNP	Chromosome	Position	P.value	Trait
S1_68772784	1	68772784	5.51E-03	GL42_14/E2
S1_68772784	1	68772784	5.51E-03	GL42_14/E2
S1_9898134	1	9898134	5.52E-03	GL42_ac
S1_9142676	1	9142676	5.52E-03	GL35_14/E2
S1_13968190	1	13968190	5.52E-03	GL14_13/E1
S1_80482739	1	80482739	5.53E-03	GL28_ac
S1_80482740	1	80482740	5.53E-03	GL28_ac
S1_14870012	1	14870012	5.55E-03	GL14_14/E2
S1_12415971	1	12415971	5.56E-03	GL28_ac
S1_14876291	1	14876291	5.57E-03	GL49_ac
S1_14876303	1	14876303	5.57E-03	GL49_ac
S1_14876334	1	14876334	5.57E-03	GL49_ac
S1_56225727	1	56225727	5.59E-03	GL42_14/E2
S1_70529622	1	70529622	5.60E-03	GL21_14/E2
S1_76479677	1	76479677	5.63E-03	GL49_13/E1
S1_12967786	1	12967786	5.65E-03	GL28_ac
S1_12596401	1	12596401	5.65E-03	GL35_ac
S1_2717923	1	2717923	5.65E-03	GL42_13/E1
S1_3547245	1	3547245	5.66E-03	GL21_13/E1
S1_63965794	1	63965794	5.67E-03	GL21_14/E2
S1_57596308	1	57596308	5.67E-03	GL14_13/E1
S1_61353815	1	61353815	5.68E-03	GL21_14/E2
S1_16745318	1	16745318	5.69E-03	GL35_14/E2
S1_13725150	1	13725150	5.71E-03	GL7_14/E2
S1_3329758	1	3329758	5.72E-03	GL21_ac
S1_12997445	1	12997445	5.73E-03	GL7_13/E1
S1_12997479	1	12997479	5.73E-03	GL7_13/E1
S1_14448619	1	14448619	5.73E-03	GL49_14/E2
S1_14448621	1	14448621	5.73E-03	GL49_14/E2
S1_14448625	1	14448625	5.73E-03	GL49_14/E2
S1_14448626	1	14448626	5.73E-03	GL49_14/E2
S1_14448627	1	14448627	5.73E-03	GL49_14/E2
S1_14448628	1	14448628	5.73E-03	GL49_14/E2
S1_14448631	1	14448631	5.73E-03	GL49_14/E2
S1_14448632	1	14448632	5.73E-03	GL49_14/E2
S1_14448634	1	14448634	5.73E-03	GL49_14/E2
S1_12249706	1	12249706	5.73E-03	GL28_ac
S1_30564915	1	30564915	5.74E-03	GL7_13/E1
S1_7388314	1	7388314	5.74E-03	GL28_ac
S1_14864964	1	14864964	5.74E-03	GL7_ac
S1_14864972	1	14864972	5.74E-03	GL7_ac
S1_72567698	1	72567698	5.75E-03	GL14_13/E1
S1_72567734	1	72567734	5.75E-03	GL14_13/E1
S1_72567739	1	72567739	5.75E-03	GL14_13/E1
S1_72567740	1	72567740	5.75E-03	GL14_13/E1
S1_78510453	1	78510453	5.75E-03	GL49_14/E2
S1_9629161	1	9629161	5.75E-03	GL49_14/E2
S1_71174651	1	71174651	5.76E-03	GL7_ac
S1_49415027	1	49415027	5.77E-03	GL28_14/E2
S1_78036426	1	78036426	5.78E-03	GL28_ac
S1_13384592	1	13384592	5.79E-03	GL14_14/E2

SNP	Chromosome	Position	P.value	Trait
S1_79402147	1	79402147	5.79E-03	GL42_14/E2
S1_12338566	1	12338566	5.81E-03	GL14_ac
S1_12596401	1	12596401	5.82E-03	GL14_ac
S1_72302128	1	72302128	5.83E-03	GL42_ac
S1_72302139	1	72302139	5.83E-03	GL42_ac
S1_12564526	1	12564526	5.84E-03	GL7_ac
S1_12564742	1	12564742	5.86E-03	GL21_14/E2
S1_5666314	1	5666314	5.88E-03	GL35_13/E1
S1_8931365	1	8931365	5.89E-03	GL35_ac
S1_14420980	1	14420980	5.92E-03	GL21_13/E1
S1_14420995	1	14420995	5.92E-03	GL21_13/E1
S1_11791521	1	11791521	5.93E-03	GL28_13/E1
S1_11791541	1	11791541	5.93E-03	GL28_13/E1
S1_78715461	1	78715461	5.96E-03	GL42_ac
S1_11764852	1	11764852	5.96E-03	GL28_13/E1
S1_3329758	1	3329758	5.96E-03	GL21_13/E1
S1_78234141	1	78234141	5.96E-03	GL49_14/E2
S1_6785913	1	6785913	5.97E-03	GL7_ac
S1_56906702	1	56906702	5.99E-03	GL42_ac
S1_11003138	1	11003138	6.04E-03	GL35_ac
S1_72611603	1	72611603	6.04E-03	GL35_ac
S1_72611646	1	72611646	6.04E-03	GL35_ac
S1_72611660	1	72611660	6.04E-03	GL35_ac
S1_1902640	1	1902640	6.05E-03	GL42_13/E1
S1_1902651	1	1902651	6.05E-03	GL42_13/E1
S1_72692082	1	72692082	6.05E-03	GL7_14/E2
S1_72692088	1	72692088	6.05E-03	GL7_14/E2
S1_12968168	1	12968168	6.05E-03	GL14_ac
S1_12968211	1	12968211	6.05E-03	GL14_ac
S1_12563723	1	12563723	6.06E-03	GL21_13/E1
S1_13102046	1	13102046	6.07E-03	GL28_13/E1
S1_71511878	1	71511878	6.07E-03	GL7_13/E1
S1_14869710	1	14869710	6.08E-03	GL42_ac
S1_16387827	1	16387827	6.08E-03	GL35_ac
S1_16387829	1	16387829	6.08E-03	GL35_ac
S1_16387838	1	16387838	6.08E-03	GL35_ac
S1_16387850	1	16387850	6.08E-03	GL35_ac
S1_69035433	1	69035433	6.09E-03	GL49_14/E2
S1_19180066	1	19180066	6.09E-03	GL21_13/E1
S1_12758577	1	12758577	6.09E-03	GL28_14/E2
S1_12338566	1	12338566	6.11E-03	GL35_13/E1
S1_11686529	1	11686529	6.11E-03	GL28_13/E1
S1_75394912	1	75394912	6.11E-03	GL49_13/E1
S1_75394958	1	75394958	6.11E-03	GL49_13/E1
S1_75394963	1	75394963	6.11E-03	GL49_13/E1
S1_75394964	1	75394964	6.11E-03	GL49_13/E1
S1_68578501	1	68578501	6.12E-03	GL7_14/E2
S1_13376221	1	13376221	6.12E-03	GL35_14/E2
S1_12758577	1	12758577	6.13E-03	GL14_13/E1
S1_12160253	1	12160253	6.15E-03	GL28_ac
S1_12160257	1	12160257	6.15E-03	GL28_ac

SNP	Chromosome	Position	P.value	Trait
S1_73866786	1	73866786	6.18E-03	GL42_ac
S1_73866784	1	73866784	6.18E-03	GL42_ac
S1_12968168	1	12968168	6.18E-03	GL28_ac
S1_12968211	1	12968211	6.18E-03	GL28_ac
S1_14162189	1	14162189	6.19E-03	GL42_13/E1
S1_12945016	1	12945016	6.21E-03	GL49_13/E1
S1_12945018	1	12945018	6.21E-03	GL49_13/E1
S1_12563731	1	12563731	6.22E-03	GL14_13/E1
S1_12563732	1	12563732	6.22E-03	GL14_13/E1
S1_12563733	1	12563733	6.22E-03	GL14_13/E1
S1_12563734	1	12563734	6.22E-03	GL14_13/E1
S1_12563735	1	12563735	6.22E-03	GL14_13/E1
S1_12563736	1	12563736	6.22E-03	GL14_13/E1
S1_12563738	1	12563738	6.22E-03	GL14_13/E1
S1_12563739	1	12563739	6.22E-03	GL14_13/E1
S1_12563723	1	12563723	6.22E-03	GL14_13/E1
S1_12156535	1	12156535	6.22E-03	GL7_ac
S1_19180066	1	19180066	6.23E-03	GL7_13/E1
S1_57111668	1	57111668	6.23E-03	GL28_13/E1
S1_12968216	1	12968216	6.24E-03	GL21_ac
S1_50894412	1	50894412	6.24E-03	GL35_14/E2
S1_26893025	1	26893025	6.25E-03	GL21_14/E2
S1_72611603	1	72611603	6.27E-03	GL28_ac
S1_72611646	1	72611646	6.27E-03	GL28_ac
S1_72611660	1	72611660	6.27E-03	GL28_ac
S1_73866784	1	73866784	6.27E-03	GL28_13/E1
S1_73866786	1	73866786	6.27E-03	GL28_13/E1
S1_73866784	1	73866784	6.28E-03	GL7_13/E1
S1_73866786	1	73866786	6.28E-03	GL7_13/E1
S1_12095456	1	12095456	6.29E-03	GL28_ac
S1_13668564	1	13668564	6.31E-03	GL14_ac
S1_79619171	1	79619171	6.32E-03	GL7_14/E2
S1_16387827	1	16387827	6.33E-03	GL28_13/E1
S1_16387829	1	16387829	6.33E-03	GL28_13/E1
S1_16387838	1	16387838	6.33E-03	GL28_13/E1
S1_16387850	1	16387850	6.33E-03	GL28_13/E1
S1_18315751	1	18315751	6.37E-03	GL49_ac
S1_18315763	1	18315763	6.37E-03	GL49_ac
S1_18315761	1	18315761	6.37E-03	GL49_ac
S1_18315778	1	18315778	6.37E-03	GL49_ac
S1_79545879	1	79545879	6.38E-03	GL14_14/E2
S1_13300990	1	13300990	6.39E-03	GL28_14/E2
S1_78753233	1	78753233	6.39E-03	GL42_14/E2
S1_63965855	1	63965855	6.41E-03	GL35_14/E2
S1_3329755	1	3329755	6.42E-03	GL21_13/E1
S1_79582017	1	79582017	6.42E-03	GL42_ac
S1_49256106	1	49256106	6.42E-03	GL28_14/E2
S1_57202401	1	57202401	6.44E-03	GL28_13/E1
S1_79195214	1	79195214	6.45E-03	GL7_14/E2
S1_17757376	1	17757376	6.47E-03	GL21_ac
S1_15084897	1	15084897	6.47E-03	GL28_14/E2

SNP	Chromosome	Position	P.value	Trait
S1_71371449	1	71371449	6.47E-03	GL49_ac
S1_15907793	1	15907793	6.48E-03	GL7_13/E1
S1_15907794	1	15907794	6.48E-03	GL7_13/E1
S1_15907796	1	15907796	6.48E-03	GL7_13/E1
S1_79195214	1	79195214	6.49E-03	GL21_14/E2
S1_19184676	1	19184676	6.50E-03	GL21_14/E2
S1_19184677	1	19184677	6.50E-03	GL21_14/E2
S1_15880752	1	15880752	6.50E-03	GL21_14/E2
S1_15880765	1	15880765	6.50E-03	GL21_14/E2
S1_15880754	1	15880754	6.50E-03	GL21_14/E2
S1_15880763	1	15880763	6.50E-03	GL21_14/E2
S1_15880766	1	15880766	6.50E-03	GL21_14/E2
S1_65668476	1	65668476	6.54E-03	GL21_13/E1
S1_65668486	1	65668486	6.54E-03	GL21_13/E1
S1_2715229	1	2715229	6.54E-03	GL21_13/E1
S1_12921087	1	12921087	6.54E-03	GL7_ac
S1_10087988	1	10087988	6.54E-03	GL21_ac
S1_18079294	1	18079294	6.56E-03	GL28_14/E2
S1_12249706	1	12249706	6.57E-03	GL21_13/E1
S1_12968216	1	12968216	6.59E-03	GL14_14/E2
S1_20696637	1	20696637	6.60E-03	GL49_14/E2
S1_78395767	1	78395767	6.62E-03	GL49_14/E2
S1_13157764	1	13157764	6.63E-03	GL28_13/E1
S1_12968216	1	12968216	6.64E-03	GL28_13/E1
S1_11625328	1	11625328	6.64E-03	GL35_13/E1
S1_75039461	1	75039461	6.64E-03	GL7_14/E2
S1_57490044	1	57490044	6.65E-03	GL21_14/E2
S1_12959162	1	12959162	6.66E-03	GL14_13/E1
S1_65668476	1	65668476	6.66E-03	GL7_ac
S1_65668486	1	65668486	6.66E-03	GL7_ac
S1_57202401	1	57202401	6.67E-03	GL35_ac
S1_1842653	1	1842653	6.69E-03	GL21_14/E2
S1_10087988	1	10087988	6.69E-03	GL7_13/E1
S1_10739071	1	10739071	6.69E-03	GL49_14/E2
S1_72611603	1	72611603	6.70E-03	GL21_ac
S1_72611646	1	72611646	6.70E-03	GL21_ac
S1_72611660	1	72611660	6.70E-03	GL21_ac
S1_72171208	1	72171208	6.71E-03	GL35_13/E1
S1_72171210	1	72171210	6.71E-03	GL35_13/E1
S1_13832006	1	13832006	6.71E-03	GL28_14/E2
S1_13300990	1	13300990	6.71E-03	GL42_ac
S1_14285586	1	14285586	6.73E-03	GL7_ac
S1_14285587	1	14285587	6.73E-03	GL7_ac
S1_14285571	1	14285571	6.73E-03	GL7_ac
S1_14285572	1	14285572	6.73E-03	GL7_ac
S1_67950145	1	67950145	6.74E-03	GL21_ac
S1_11625328	1	11625328	6.76E-03	GL21_ac
S1_12122950	1	12122950	6.76E-03	GL35_13/E1
S1_12338566	1	12338566	6.78E-03	GL28_13/E1
S1_12932509	1	12932509	6.78E-03	GL14_ac
S1_79638265	1	79638265	6.78E-03	GL28_13/E1

SNP	Chromosome	Position	P.value	Trait
S1_78752784	1	78752784	6.78E-03	GL49_ac
S1_14864964	1	14864964	6.78E-03	GL28_13/E1
S1_14864972	1	14864972	6.78E-03	GL28_13/E1
S1_6234373	1	6234373	6.79E-03	GL35_13/E1
S1_6234392	1	6234392	6.79E-03	GL35_13/E1
S1_72940285	1	72940285	6.79E-03	GL42_13/E1
S1_72940286	1	72940286	6.79E-03	GL42_13/E1
S1_72940300	1	72940300	6.79E-03	GL42_13/E1
S1_72940335	1	72940335	6.79E-03	GL42_13/E1
S1_50787048	1	50787048	6.80E-03	GL14_13/E1
S1_50787051	1	50787051	6.80E-03	GL14_13/E1
S1_14448620	1	14448620	6.80E-03	GL49_ac
S1_14869710	1	14869710	6.82E-03	GL35_13/E1
S1_57596308	1	57596308	6.82E-03	GL28_ac
S1_10087988	1	10087988	6.83E-03	GL35_13/E1
S1_13892084	1	13892084	6.83E-03	GL14_14/E2
S1_13892096	1	13892096	6.83E-03	GL14_14/E2
S1_64086235	1	64086235	6.84E-03	GL14_14/E2
S1_64086236	1	64086236	6.84E-03	GL14_14/E2
S1_79638265	1	79638265	6.86E-03	GL21_ac
S1_11686529	1	11686529	6.86E-03	GL49_13/E1
S1_72567698	1	72567698	6.87E-03	GL7_13/E1
S1_72567734	1	72567734	6.87E-03	GL7_13/E1
S1_72567739	1	72567739	6.87E-03	GL7_13/E1
S1_72567740	1	72567740	6.87E-03	GL7_13/E1
S1_72940285	1	72940285	6.89E-03	GL28_13/E1
S1_72940286	1	72940286	6.89E-03	GL28_13/E1
S1_72940300	1	72940300	6.89E-03	GL28_13/E1
S1_72940335	1	72940335	6.89E-03	GL28_13/E1
S1_17757376	1	17757376	6.90E-03	GL21_13/E1
S1_12030980	1	12030980	6.91E-03	GL35_14/E2
S1_31874208	1	31874208	6.92E-03	GL7_14/E2
S1_57202401	1	57202401	6.95E-03	GL49_ac
S1_57202401	1	57202401	6.95E-03	GL21_13/E1
S1_21469663	1	21469663	6.96E-03	GL14_14/E2
S1_12758577	1	12758577	6.98E-03	GL35_ac
S1_13300990	1	13300990	6.99E-03	GL14_14/E2
S1_1795296	1	1795296	6.99E-03	GL49_13/E1
S1_12564526	1	12564526	7.00E-03	GL42_ac
S1_16390928	1	16390928	7.00E-03	GL49_ac
S1_16390978	1	16390978	7.00E-03	GL49_ac
S1_65257377	1	65257377	7.00E-03	GL7_ac
S1_65257392	1	65257392	7.00E-03	GL7_ac
S1_65257422	1	65257422	7.00E-03	GL7_ac
S1_19017797	1	19017797	7.01E-03	GL42_ac
S1_67950145	1	67950145	7.04E-03	GL35_ac
S1_63389994	1	63389994	7.05E-03	GL21_14/E2
S1_65649103	1	65649103	7.05E-03	GL21_14/E2
S1_12785104	1	12785104	7.06E-03	GL42_13/E1
S1_12785120	1	12785120	7.06E-03	GL42_13/E1
S1_14672796	1	14672796	7.09E-03	GL35_14/E2

SNP	Chromosome	Position	P.value	Trait
S1_78748478	1	78748478	7.10E-03	GL49_14/E2
S1_12781217	1	12781217	7.11E-03	GL7_13/E1
S1_72430279	1	72430279	7.12E-03	GL49_ac
S1_54333136	1	54333136	7.13E-03	GL28_14/E2
S1_6625660	1	6625660	7.13E-03	GL42_ac
S1_66991835	1	66991835	7.14E-03	GL35_14/E2
S1_13892084	1	13892084	7.15E-03	GL14_ac
S1_13892096	1	13892096	7.15E-03	GL14_ac
S1_9898134	1	9898134	7.18E-03	GL42_14/E2
S1_28470926	1	28470926	7.19E-03	GL21_13/E1
S1_18901381	1	18901381	7.20E-03	GL42_14/E2
S1_62825493	1	62825493	7.21E-03	GL42_14/E2
S1_17285880	1	17285880	7.21E-03	GL35_14/E2
S1_14330617	1	14330617	7.21E-03	GL7_13/E1
S1_14330622	1	14330622	7.21E-03	GL7_13/E1
S1_12968198	1	12968198	7.26E-03	GL14_ac
S1_79990582	1	79990582	7.26E-03	GL21_ac
S1_19184676	1	19184676	7.27E-03	GL14_14/E2
S1_19184677	1	19184677	7.27E-03	GL14_14/E2
S1_13427114	1	13427114	7.29E-03	GL35_ac
S1_11431224	1	11431224	7.29E-03	GL7_ac
S1_9142676	1	9142676	7.30E-03	GL28_14/E2
S1_74323691	1	74323691	7.32E-03	GL14_13/E1
S1_74323695	1	74323695	7.32E-03	GL14_13/E1
S1_74323696	1	74323696	7.32E-03	GL14_13/E1
S1_74323697	1	74323697	7.32E-03	GL14_13/E1
S1_12997445	1	12997445	7.32E-03	GL14_13/E1
S1_12997479	1	12997479	7.32E-03	GL14_13/E1
S1_18315751	1	18315751	7.32E-03	GL14_14/E2
S1_18315763	1	18315763	7.32E-03	GL14_14/E2
S1_18315761	1	18315761	7.32E-03	GL14_14/E2
S1_18315778	1	18315778	7.32E-03	GL14_14/E2
S1_1842653	1	1842653	7.34E-03	GL14_14/E2
S1_12160253	1	12160253	7.34E-03	GL28_13/E1
S1_12160257	1	12160257	7.34E-03	GL28_13/E1
S1_15084897	1	15084897	7.35E-03	GL14_ac
S1_75394912	1	75394912	7.35E-03	GL21_13/E1
S1_75394958	1	75394958	7.35E-03	GL21_13/E1
S1_75394963	1	75394963	7.35E-03	GL21_13/E1
S1_75394964	1	75394964	7.35E-03	GL21_13/E1
S1_12338566	1	12338566	7.36E-03	GL21_13/E1
S1_15084897	1	15084897	7.37E-03	GL21_14/E2
S1_78711570	1	78711570	7.37E-03	GL49_14/E2
S1_57111668	1	57111668	7.39E-03	GL21_13/E1
S1_15907793	1	15907793	7.39E-03	GL14_ac
S1_15907794	1	15907794	7.39E-03	GL14_ac
S1_15907796	1	15907796	7.39E-03	GL14_ac
S1_14285586	1	14285586	7.40E-03	GL49_ac
S1_14285587	1	14285587	7.40E-03	GL49_ac
S1_14285571	1	14285571	7.40E-03	GL49_ac
S1_14285572	1	14285572	7.40E-03	GL49_ac

SNP	Chromosome	Position	P.value	Trait
S1_57676176	1	57676176	7.42E-03	GL49_ac
S1_57676228	1	57676228	7.42E-03	GL49_ac
S1_57202401	1	57202401	7.43E-03	GL42_13/E1
S1_72772586	1	72772586	7.43E-03	GL28_13/E1
S1_26888112	1	26888112	7.43E-03	GL28_ac
S1_7248001	1	7248001	7.44E-03	GL49_14/E2
S1_12921087	1	12921087	7.46E-03	GL35_14/E2
S1_9898152	1	9898152	7.47E-03	GL35_14/E2
S1_26893025	1	26893025	7.47E-03	GL35_14/E2
S1_63226451	1	63226451	7.48E-03	GL21_ac
S1_63226451	1	63226451	7.48E-03	GL21_ac
S1_16432036	1	16432036	7.48E-03	GL21_14/E2
S1_50830084	1	50830084	7.49E-03	GL42_13/E1
S1_50830102	1	50830102	7.49E-03	GL42_13/E1
S1_16484731	1	16484731	7.50E-03	GL7_14/E2
S1_12921087	1	12921087	7.51E-03	GL21_14/E2
S1_65223559	1	65223559	7.52E-03	GL7_14/E2
S1_65223574	1	65223574	7.52E-03	GL7_14/E2
S1_55831986	1	55831986	7.53E-03	GL35_14/E2
S1_1897884	1	1897884	7.55E-03	GL42_13/E1
S1_603	1	603	7.56E-03	GL21_13/E1
S1_12959333	1	12959333	7.56E-03	GL28_ac
S1_65668476	1	65668476	7.57E-03	GL28_ac
S1_65668486	1	65668486	7.57E-03	GL28_ac
S1_12758577	1	12758577	7.61E-03	GL35_13/E1
S1_74876549	1	74876549	7.61E-03	GL21_ac
S1_16390928	1	16390928	7.61E-03	GL14_ac
S1_16390978	1	16390978	7.61E-03	GL14_ac
S1_17532285	1	17532285	7.61E-03	GL42_13/E1
S1_72171208	1	72171208	7.64E-03	GL42_13/E1
S1_72171210	1	72171210	7.64E-03	GL42_13/E1
S1_12030980	1	12030980	7.66E-03	GL14_13/E1
S1_10087988	1	10087988	7.66E-03	GL28_ac
S1_9142676	1	9142676	7.68E-03	GL49_14/E2
S1_75378360	1	75378360	7.70E-03	GL28_14/E2
S1_78036426	1	78036426	7.71E-03	GL35_ac
S1_11764852	1	11764852	7.72E-03	GL49_13/E1
S1_67065341	1	67065341	7.72E-03	GL7_14/E2
S1_11625328	1	11625328	7.72E-03	GL14_ac
S1_7576799	1	7576799	7.73E-03	GL21_14/E2
S1_80576324	1	80576324	7.74E-03	GL21_14/E2
S1_12564526	1	12564526	7.74E-03	GL7_14/E2
S1_12627779	1	12627779	7.75E-03	GL42_13/E1
S1_16432036	1	16432036	7.78E-03	GL21_ac
S1_79619171	1	79619171	7.79E-03	GL7_ac
S1_57596308	1	57596308	7.79E-03	GL28_13/E1
S1_3547245	1	3547245	7.79E-03	GL42_13/E1
S1_9898134	1	9898134	7.80E-03	GL35_14/E2
S1_56906702	1	56906702	7.81E-03	GL14_ac
S1_70223610	1	70223610	7.82E-03	GL42_ac
S1_72611603	1	72611603	7.83E-03	GL21_14/E2

SNP	Chromosome	Position	P.value	Trait
S1_72611646	1	72611646	7.83E-03	GL21_14/E2
S1_72611660	1	72611660	7.83E-03	GL21_14/E2
S1_6239640	1	6239640	7.83E-03	GL14_14/E2
S1_12627604	1	12627604	7.83E-03	GL14_ac
S1_79195214	1	79195214	7.83E-03	GL14_14/E2
S1_12563723	1	12563723	7.85E-03	GL28_13/E1
S1_3329755	1	3329755	7.86E-03	GL21_ac
S1_22035471	1	22035471	7.87E-03	GL21_14/E2
S1_72205632	1	72205632	7.88E-03	GL7_ac
S1_3547245	1	3547245	7.89E-03	GL28_13/E1
S1_13095759	1	13095759	7.90E-03	GL35_ac
S1_13095746	1	13095746	7.90E-03	GL35_ac
S1_55062451	1	55062451	7.91E-03	GL35_ac
S1_12997445	1	12997445	7.91E-03	GL35_13/E1
S1_12997479	1	12997479	7.91E-03	GL35_13/E1
S1_71553878	1	71553878	7.91E-03	GL49_ac
S1_8180759	1	8180759	7.91E-03	GL42_14/E2
S1_19104755	1	19104755	7.93E-03	GL42_14/E2
S1_27385834	1	27385834	7.95E-03	GL21_ac
S1_73776874	1	73776874	7.95E-03	GL7_13/E1
S1_71606027	1	71606027	7.96E-03	GL42_ac
S1_71606019	1	71606019	7.96E-03	GL42_ac
S1_12627739	1	12627739	7.98E-03	GL35_ac
S1_56549114	1	56549114	7.98E-03	GL21_13/E1
S1_56549114	1	56549114	7.98E-03	GL21_13/E1
S1_74359940	1	74359940	8.01E-03	GL42_13/E1
S1_16387827	1	16387827	8.01E-03	GL21_13/E1
S1_16387829	1	16387829	8.01E-03	GL21_13/E1
S1_16387838	1	16387838	8.01E-03	GL21_13/E1
S1_16387850	1	16387850	8.01E-03	GL21_13/E1
S1_13469184	1	13469184	8.02E-03	GL35_ac
S1_13469196	1	13469196	8.02E-03	GL35_ac
S1_14512256	1	14512256	8.06E-03	GL7_14/E2
S1_71553878	1	71553878	8.06E-03	GL21_ac
S1_12959162	1	12959162	8.06E-03	GL28_13/E1
S1_61353815	1	61353815	8.06E-03	GL28_14/E2
S1_78200697	1	78200697	8.08E-03	GL42_14/E2
S1_59558610	1	59558610	8.08E-03	GL7_ac
S1_56906702	1	56906702	8.09E-03	GL7_ac
S1_55062451	1	55062451	8.09E-03	GL49_14/E2
S1_12227113	1	12227113	8.09E-03	GL7_13/E1
S1_12227115	1	12227115	8.09E-03	GL7_13/E1
S1_12165733	1	12165733	8.10E-03	GL14_ac
S1_58937781	1	58937781	8.10E-03	GL42_ac
S1_58937838	1	58937838	8.10E-03	GL42_ac
S1_79582017	1	79582017	8.11E-03	GL21_14/E2
S1_19244209	1	19244209	8.11E-03	GL28_13/E1
S1_62723977	1	62723977	8.12E-03	GL21_13/E1
S1_3329764	1	3329764	8.12E-03	GL21_13/E1
S1_74876553	1	74876553	8.14E-03	GL21_ac
S1_74876556	1	74876556	8.14E-03	GL21_ac

SNP	Chromosome	Position	P.value	Trait
S1_17757376	1	17757376	8.15E-03	GL28_ac
S1_12596401	1	12596401	8.18E-03	GL7_ac
S1_65649103	1	65649103	8.18E-03	GL35_ac
S1_65668476	1	65668476	8.19E-03	GL21_ac
S1_65668486	1	65668486	8.19E-03	GL21_ac
S1_2715229	1	2715229	8.19E-03	GL42_ac
S1_50787048	1	50787048	8.20E-03	GL42_13/E1
S1_50787051	1	50787051	8.20E-03	GL42_13/E1
S1_66310194	1	66310194	8.20E-03	GL21_14/E2
S1_56906702	1	56906702	8.21E-03	GL21_ac
S1_13312936	1	13312936	8.22E-03	GL7_ac
S1_3329760	1	3329760	8.23E-03	GL21_13/E1
S1_49256106	1	49256106	8.24E-03	GL21_14/E2
S1_12249706	1	12249706	8.25E-03	GL21_ac
S1_3329758	1	3329758	8.25E-03	GL28_ac
S1_16390928	1	16390928	8.26E-03	GL35_ac
S1_16390978	1	16390978	8.26E-03	GL35_ac
S1_71174651	1	71174651	8.30E-03	GL14_14/E2
S1_72611603	1	72611603	8.31E-03	GL28_13/E1
S1_72611646	1	72611646	8.31E-03	GL28_13/E1
S1_72611660	1	72611660	8.31E-03	GL28_13/E1
S1_11686529	1	11686529	8.31E-03	GL21_13/E1
S1_16390928	1	16390928	8.32E-03	GL28_ac
S1_16390978	1	16390978	8.32E-03	GL28_ac
S1_12030980	1	12030980	8.32E-03	GL42_ac
S1_16731242	1	16731242	8.33E-03	GL14_ac
S1_16731268	1	16731268	8.33E-03	GL14_ac
S1_16731272	1	16731272	8.33E-03	GL14_ac
S1_27385834	1	27385834	8.33E-03	GL21_13/E1
S1_64086235	1	64086235	8.35E-03	GL21_14/E2
S1_64086236	1	64086236	8.35E-03	GL21_14/E2
S1_12160253	1	12160253	8.35E-03	GL14_14/E2
S1_12160257	1	12160257	8.35E-03	GL14_14/E2
S1_78036426	1	78036426	8.35E-03	GL35_14/E2
S1_12095456	1	12095456	8.39E-03	GL7_ac
S1_76661673	1	76661673	8.39E-03	GL49_ac
S1_76661685	1	76661685	8.39E-03	GL49_ac
S1_56568091	1	56568091	8.41E-03	GL21_14/E2
S1_3329760	1	3329760	8.42E-03	GL21_ac
S1_67190094	1	67190094	8.44E-03	GL14_13/E1
S1_77343688	1	77343688	8.45E-03	GL35_14/E2
S1_77343694	1	77343694	8.45E-03	GL35_14/E2
S1_77343698	1	77343698	8.45E-03	GL35_14/E2
S1_77343689	1	77343689	8.45E-03	GL35_14/E2
S1_77343690	1	77343690	8.45E-03	GL35_14/E2
S1_77343693	1	77343693	8.45E-03	GL35_14/E2
S1_77343695	1	77343695	8.45E-03	GL35_14/E2
S1_77343696	1	77343696	8.45E-03	GL35_14/E2
S1_77343699	1	77343699	8.45E-03	GL35_14/E2
S1_12338566	1	12338566	8.46E-03	GL35_ac
S1_59985148	1	59985148	8.47E-03	GL42_14/E2

SNP	Chromosome	Position	P.value	Trait
S1_59985195	1	59985195	8.47E-03	GL42_14/E2
S1_72927335	1	72927335	8.47E-03	GL7_14/E2
S1_45942285	1	45942285	8.50E-03	GL49_13/E1
S1_12758238	1	12758238	8.51E-03	GL21_13/E1
S1_55923588	1	55923588	8.51E-03	GL35_14/E2
S1_15907793	1	15907793	8.52E-03	GL14_13/E1
S1_15907794	1	15907794	8.52E-03	GL14_13/E1
S1_15907796	1	15907796	8.52E-03	GL14_13/E1
S1_2458035	1	2458035	8.54E-03	GL35_13/E1
S1_14869710	1	14869710	8.54E-03	GL35_14/E2
S1_65324937	1	65324937	8.54E-03	GL7_13/E1
S1_12758577	1	12758577	8.55E-03	GL21_14/E2
S1_77252113	1	77252113	8.55E-03	GL7_13/E1
S1_14285571	1	14285571	8.55E-03	GL14_13/E1
S1_14285572	1	14285572	8.55E-03	GL14_13/E1
S1_14285586	1	14285586	8.55E-03	GL14_13/E1
S1_14285587	1	14285587	8.55E-03	GL14_13/E1
S1_2715229	1	2715229	8.56E-03	GL42_13/E1
S1_16731242	1	16731242	8.56E-03	GL28_13/E1
S1_16731268	1	16731268	8.56E-03	GL28_13/E1
S1_16731272	1	16731272	8.56E-03	GL28_13/E1
S1_16647171	1	16647171	8.58E-03	GL21_ac
S1_73866786	1	73866786	8.59E-03	GL28_ac
S1_73866784	1	73866784	8.59E-03	GL28_ac
S1_7576799	1	7576799	8.60E-03	GL14_ac
S1_65740609	1	65740609	8.60E-03	GL14_14/E2
S1_65740630	1	65740630	8.60E-03	GL14_14/E2
S1_12758238	1	12758238	8.60E-03	GL35_14/E2
S1_19718165	1	19718165	8.61E-03	GL42_14/E2
S1_72692099	1	72692099	8.61E-03	GL35_ac
S1_20338764	1	20338764	8.61E-03	GL7_14/E2
S1_72766670	1	72766670	8.62E-03	GL7_14/E2
S1_16390928	1	16390928	8.62E-03	GL21_ac
S1_16390978	1	16390978	8.62E-03	GL21_ac
S1_12030980	1	12030980	8.64E-03	GL14_ac
S1_12249706	1	12249706	8.66E-03	GL49_13/E1
S1_16387827	1	16387827	8.67E-03	GL28_14/E2
S1_16387829	1	16387829	8.67E-03	GL28_14/E2
S1_16387838	1	16387838	8.67E-03	GL28_14/E2
S1_16387850	1	16387850	8.67E-03	GL28_14/E2
S1_15315301	1	15315301	8.67E-03	GL7_14/E2
S1_16484731	1	16484731	8.68E-03	GL21_14/E2
S1_10739071	1	10739071	8.69E-03	GL42_ac
S1_12945016	1	12945016	8.70E-03	GL28_13/E1
S1_12945018	1	12945018	8.70E-03	GL28_13/E1
S1_50787048	1	50787048	8.71E-03	GL7_13/E1
S1_50787051	1	50787051	8.71E-03	GL7_13/E1
S1_14462372	1	14462372	8.71E-03	GL35_14/E2
S1_12968198	1	12968198	8.72E-03	GL28_ac
S1_14854881	1	14854881	8.74E-03	GL35_ac
S1_56567618	1	56567618	8.75E-03	GL42_ac

SNP	Chromosome	Position	P.value	Trait
S1_50894412	1	50894412	8.75E-03	GL21_14/E2
S1_12563731	1	12563731	8.75E-03	GL35_13/E1
S1_12563732	1	12563732	8.75E-03	GL35_13/E1
S1_12563733	1	12563733	8.75E-03	GL35_13/E1
S1_12563734	1	12563734	8.75E-03	GL35_13/E1
S1_12563735	1	12563735	8.75E-03	GL35_13/E1
S1_12563736	1	12563736	8.75E-03	GL35_13/E1
S1_12563738	1	12563738	8.75E-03	GL35_13/E1
S1_12563739	1	12563739	8.75E-03	GL35_13/E1
S1_12563723	1	12563723	8.75E-03	GL35_13/E1
S1_28200982	1	28200982	8.77E-03	GL35_13/E1
S1_14869710	1	14869710	8.77E-03	GL42_14/E2
S1_8998667	1	8998667	8.78E-03	GL42_13/E1
S1_8998674	1	8998674	8.78E-03	GL42_13/E1
S1_13300990	1	13300990	8.78E-03	GL7_13/E1
S1_71707259	1	71707259	8.79E-03	GL14_14/E2
S1_71707261	1	71707261	8.79E-03	GL14_14/E2
S1_80344898	1	80344898	8.79E-03	GL21_14/E2
S1_79990582	1	79990582	8.80E-03	GL21_13/E1
S1_14448620	1	14448620	8.80E-03	GL35_ac
S1_57202401	1	57202401	8.81E-03	GL28_ac
S1_12967786	1	12967786	8.81E-03	GL14_ac
S1_3351629	1	3351629	8.84E-03	GL14_14/E2
S1_79990582	1	79990582	8.84E-03	GL14_ac
S1_14876291	1	14876291	8.84E-03	GL21_14/E2
S1_14876303	1	14876303	8.84E-03	GL21_14/E2
S1_14876334	1	14876334	8.84E-03	GL21_14/E2
S1_74359940	1	74359940	8.84E-03	GL21_13/E1
S1_13188261	1	13188261	8.85E-03	GL21_13/E1
S1_12757772	1	12757772	8.87E-03	GL14_13/E1
S1_12959333	1	12959333	8.89E-03	GL7_13/E1
S1_78753233	1	78753233	8.89E-03	GL28_ac
S1_79016620	1	79016620	8.89E-03	GL42_ac
S1_72567734	1	72567734	8.89E-03	GL49_ac
S1_72567739	1	72567739	8.89E-03	GL49_ac
S1_72567698	1	72567698	8.89E-03	GL49_ac
S1_72567740	1	72567740	8.89E-03	GL49_ac
S1_67950145	1	67950145	8.90E-03	GL49_ac
S1_14285571	1	14285571	8.91E-03	GL35_13/E1
S1_14285572	1	14285572	8.91E-03	GL35_13/E1
S1_14285586	1	14285586	8.91E-03	GL35_13/E1
S1_14285587	1	14285587	8.91E-03	GL35_13/E1
S1_12922040	1	12922040	8.93E-03	GL28_ac
S1_68883084	1	68883084	8.93E-03	GL42_ac
S1_68883119	1	68883119	8.93E-03	GL42_ac
S1_68883121	1	68883121	8.93E-03	GL42_ac
S1_68883124	1	68883124	8.93E-03	GL42_ac
S1_68883135	1	68883135	8.93E-03	GL42_ac
S1_68883113	1	68883113	8.93E-03	GL42_ac
S1_68883141	1	68883141	8.93E-03	GL42_ac
S1_73776874	1	73776874	8.95E-03	GL14_13/E1

SNP	Chromosome	Position	P.value	Trait
S1_51240322	1	51240322	8.95E-03	GL14_13/E1
S1_17532282	1	17532282	8.96E-03	GL28_13/E1
S1_17532284	1	17532284	8.96E-03	GL28_13/E1
S1_17532290	1	17532290	8.96E-03	GL28_13/E1
S1_75427740	1	75427740	8.97E-03	GL14_14/E2
S1_14869710	1	14869710	8.97E-03	GL42_13/E1
S1_72691333	1	72691333	8.98E-03	GL49_14/E2
S1_72691345	1	72691345	8.98E-03	GL49_14/E2
S1_72691349	1	72691349	8.98E-03	GL49_14/E2
S1_72691344	1	72691344	8.98E-03	GL49_14/E2
S1_75624748	1	75624748	9.01E-03	GL49_14/E2
S1_75624752	1	75624752	9.01E-03	GL49_14/E2
S1_79990584	1	79990584	9.01E-03	GL21_13/E1
S1_79990589	1	79990589	9.01E-03	GL21_13/E1
S1_79990590	1	79990590	9.01E-03	GL21_13/E1
S1_67950145	1	67950145	9.02E-03	GL42_14/E2
S1_62723977	1	62723977	9.03E-03	GL28_13/E1
S1_11431224	1	11431224	9.04E-03	GL14_ac
S1_63226451	1	63226451	9.05E-03	GL28_14/E2
S1_63226451	1	63226451	9.05E-03	GL28_14/E2
S1_14690005	1	14690005	9.05E-03	GL35_14/E2
S1_3329758	1	3329758	9.06E-03	GL28_13/E1
S1_2717923	1	2717923	9.06E-03	GL49_13/E1
S1_11003165	1	11003165	9.07E-03	GL21_14/E2
S1_12959162	1	12959162	9.08E-03	GL7_13/E1
S1_64365057	1	64365057	9.09E-03	GL42_ac
S1_61603639	1	61603639	9.09E-03	GL42_13/E1
S1_78485839	1	78485839	9.10E-03	GL42_14/E2
S1_74359940	1	74359940	9.10E-03	GL21_ac
S1_11125364	1	11125364	9.11E-03	GL35_13/E1
S1_57740678	1	57740678	9.11E-03	GL14_13/E1
S1_65668476	1	65668476	9.12E-03	GL7_13/E1
S1_65668486	1	65668486	9.12E-03	GL7_13/E1
S1_29271525	1	29271525	9.13E-03	GL21_14/E2
S1_22746381	1	22746381	9.13E-03	GL42_13/E1
S1_57596308	1	57596308	9.16E-03	GL7_13/E1
S1_9664234	1	9664234	9.16E-03	GL49_14/E2
S1_56033556	1	56033556	9.17E-03	GL35_ac
S1_56033561	1	56033561	9.17E-03	GL35_ac
S1_56033562	1	56033562	9.17E-03	GL35_ac
S1_56033564	1	56033564	9.17E-03	GL35_ac
S1_56033554	1	56033554	9.17E-03	GL35_ac
S1_56033565	1	56033565	9.17E-03	GL35_ac
S1_72171208	1	72171208	9.17E-03	GL49_13/E1
S1_72171210	1	72171210	9.17E-03	GL49_13/E1
S1_56033559	1	56033559	9.18E-03	GL35_ac
S1_13725150	1	13725150	9.20E-03	GL28_14/E2
S1_19180066	1	19180066	9.20E-03	GL14_ac
S1_79967071	1	79967071	9.23E-03	GL42_14/E2
S1_78106488	1	78106488	9.26E-03	GL7_14/E2
S1_12959162	1	12959162	9.26E-03	GL28_ac

SNP	Chromosome	Position	P.value	Trait
S1_64993130	1	64993130	9.27E-03	GL7_14/E2
S1_71731573	1	71731573	9.27E-03	GL35_ac
S1_72205632	1	72205632	9.29E-03	GL35_ac
S1_16647171	1	16647171	9.29E-03	GL7_ac
S1_16387827	1	16387827	9.31E-03	GL42_13/E1
S1_16387829	1	16387829	9.31E-03	GL42_13/E1
S1_16387838	1	16387838	9.31E-03	GL42_13/E1
S1_16387850	1	16387850	9.31E-03	GL42_13/E1
S1_77214030	1	77214030	9.32E-03	GL21_14/E2
S1_3105950	1	3105950	9.36E-03	GL28_13/E1
S1_16387827	1	16387827	9.37E-03	GL7_13/E1
S1_16387829	1	16387829	9.37E-03	GL7_13/E1
S1_16387838	1	16387838	9.37E-03	GL7_13/E1
S1_16387850	1	16387850	9.37E-03	GL7_13/E1
S1_63226451	1	63226451	9.40E-03	GL28_ac
S1_63226451	1	63226451	9.40E-03	GL28_ac
S1_19180066	1	19180066	9.42E-03	GL28_13/E1
S1_4581926	1	4581926	9.43E-03	GL28_14/E2
S1_72171208	1	72171208	9.43E-03	GL7_13/E1
S1_72171210	1	72171210	9.43E-03	GL7_13/E1
S1_3329764	1	3329764	9.45E-03	GL21_ac
S1_71861477	1	71861477	9.46E-03	GL14_14/E2
S1_72166164	1	72166164	9.46E-03	GL21_14/E2
S1_71731573	1	71731573	9.47E-03	GL28_ac
S1_68578501	1	68578501	9.47E-03	GL21_14/E2
S1_63389994	1	63389994	9.47E-03	GL35_ac
S1_68069848	1	68069848	9.47E-03	GL14_ac
S1_1880194	1	1880194	9.47E-03	GL49_ac
S1_7576799	1	7576799	9.47E-03	GL35_ac
S1_78752784	1	78752784	9.50E-03	GL7_14/E2
S1_14870012	1	14870012	9.50E-03	GL21_ac
S1_79529450	1	79529450	9.50E-03	GL21_14/E2
S1_6059478	1	6059478	9.53E-03	GL7_14/E2
S1_68777771	1	68777771	9.53E-03	GL7_14/E2
S1_12596401	1	12596401	9.53E-03	GL14_14/E2
S1_73644317	1	73644317	9.55E-03	GL42_14/E2
S1_73644284	1	73644284	9.55E-03	GL42_14/E2
S1_73644308	1	73644308	9.55E-03	GL42_14/E2
S1_12758577	1	12758577	9.57E-03	GL35_14/E2
S1_12968216	1	12968216	9.59E-03	GL28_ac
S1_67801983	1	67801983	9.59E-03	GL28_14/E2
S1_9898152	1	9898152	9.61E-03	GL42_ac
S1_71363969	1	71363969	9.62E-03	GL14_14/E2
S1_12945016	1	12945016	9.63E-03	GL42_13/E1
S1_12945018	1	12945018	9.63E-03	GL42_13/E1
S1_3329754	1	3329754	9.63E-03	GL21_13/E1
S1_3329757	1	3329757	9.63E-03	GL21_13/E1
S1_3329763	1	3329763	9.63E-03	GL21_13/E1
S1_3329765	1	3329765	9.63E-03	GL21_13/E1
S1_28195074	1	28195074	9.63E-03	GL7_14/E2
S1_12627739	1	12627739	9.64E-03	GL14_ac

SNP	Chromosome	Position	P.value	Trait
S1_12160253	1	12160253	9.64E-03	GL21_ac
S1_12160257	1	12160257	9.64E-03	GL21_ac
S1_78510453	1	78510453	9.64E-03	GL42_14/E2
S1_72949911	1	72949911	9.65E-03	GL35_14/E2
S1_72949963	1	72949963	9.65E-03	GL35_14/E2
S1_72949908	1	72949908	9.65E-03	GL35_14/E2
S1_72949913	1	72949913	9.65E-03	GL35_14/E2
S1_72949915	1	72949915	9.65E-03	GL35_14/E2
S1_72949918	1	72949918	9.65E-03	GL35_14/E2
S1_72949921	1	72949921	9.65E-03	GL35_14/E2
S1_72949964	1	72949964	9.65E-03	GL35_14/E2
S1_71731573	1	71731573	9.66E-03	GL7_ac
S1_72940285	1	72940285	9.67E-03	GL49_ac
S1_72940286	1	72940286	9.67E-03	GL49_ac
S1_72940300	1	72940300	9.67E-03	GL49_ac
S1_72940335	1	72940335	9.67E-03	GL49_ac
S1_67567570	1	67567570	9.67E-03	GL21_13/E1
S1_13892084	1	13892084	9.69E-03	GL21_ac
S1_13892096	1	13892096	9.69E-03	GL21_ac
S1_53772305	1	53772305	9.70E-03	GL14_13/E1
S1_53772311	1	53772311	9.70E-03	GL14_13/E1
S1_14512256	1	14512256	9.71E-03	GL28_13/E1
S1_12922040	1	12922040	9.72E-03	GL21_13/E1
S1_7222657	1	7222657	9.74E-03	GL14_ac
S1_7222659	1	7222659	9.74E-03	GL14_ac
S1_7222675	1	7222675	9.74E-03	GL14_ac
S1_7222692	1	7222692	9.74E-03	GL14_ac
S1_7222713	1	7222713	9.74E-03	GL14_ac
S1_13832006	1	13832006	9.76E-03	GL21_14/E2
S1_14672796	1	14672796	9.76E-03	GL14_ac
S1_9705879	1	9705879	9.76E-03	GL28_13/E1
S1_75765686	1	75765686	9.78E-03	GL42_14/E2
S1_75765696	1	75765696	9.78E-03	GL42_14/E2
S1_12595162	1	12595162	9.78E-03	GL21_ac
S1_59558610	1	59558610	9.79E-03	GL28_13/E1
S1_72938515	1	72938515	9.79E-03	GL42_13/E1
S1_72938517	1	72938517	9.79E-03	GL42_13/E1
S1_72205632	1	72205632	9.83E-03	GL35_13/E1
S1_7769551	1	7769551	9.85E-03	GL28_14/E2
S1_60816164	1	60816164	9.87E-03	GL49_13/E1
S1_73866784	1	73866784	9.88E-03	GL42_13/E1
S1_73866786	1	73866786	9.88E-03	GL42_13/E1
S1_50830279	1	50830279	9.89E-03	GL7_14/E2
S1_67294739	1	67294739	9.92E-03	GL7_ac
S1_67190094	1	67190094	9.93E-03	GL7_13/E1
S1_22055615	1	22055615	9.93E-03	GL49_14/E2
S1_11764852	1	11764852	9.94E-03	GL21_ac
S1_57111668	1	57111668	9.94E-03	GL35_13/E1
S1_27278097	1	27278097	9.94E-03	GL28_14/E2
S1_12143895	1	12143895	9.97E-03	GL28_13/E1
S1_7222657	1	7222657	9.98E-03	GL7_ac

SNP	Chromosome	Position	P.value	Trait
S1_7222659	1	7222659	9.98E-03	GL7_ac
S1_7222675	1	7222675	9.98E-03	GL7_ac
S1_7222692	1	7222692	9.98E-03	GL7_ac
S1_7222713	1	7222713	9.98E-03	GL7_ac
S1_12627739	1	12627739	9.99E-03	GL49_13/E1
S1_75394912	1	75394912	9.99E-03	GL28_13/E1
S1_75394958	1	75394958	9.99E-03	GL28_13/E1
S1_75394963	1	75394963	9.99E-03	GL28_13/E1
S1_75394964	1	75394964	9.99E-03	GL28_13/E1
S2_68505778	2	68505778	6.35E-05	GL42_14/E2
S2_60856616	2	60856616	8.30E-05	GL21_14/E2
S2_63692993	2	63692993	2.21E-04	GL7_14/E2
S2_63693006	2	63693006	2.21E-04	GL7_14/E2
S2_12894427	2	12894427	2.52E-04	GL49_ac
S2_12894456	2	12894456	2.52E-04	GL49_ac
S2_63803728	2	63803728	4.83E-04	GL7_14/E2
S2_63803722	2	63803722	5.14E-04	GL7_14/E2
S2_63803726	2	63803726	5.14E-04	GL7_14/E2
S2_63803729	2	63803729	5.14E-04	GL7_14/E2
S2_45863094	2	45863094	5.45E-04	GL7_13/E1
S2_5786269	2	5786269	5.56E-04	GL42_13/E1
S2_67899865	2	67899865	5.56E-04	GL49_13/E1
S2_67463962	2	67463962	5.85E-04	GL35_14/E2
S2_8661367	2	8661367	6.10E-04	GL35_14/E2
S2_60856616	2	60856616	6.56E-04	GL7_14/E2
S2_199703	2	199703	6.79E-04	GL42_14/E2
S2_63057180	2	63057180	6.97E-04	GL35_ac
S2_57042267	2	57042267	7.21E-04	GL21_14/E2
S2_18307240	2	18307240	7.32E-04	GL21_14/E2
S2_18307256	2	18307256	7.32E-04	GL21_14/E2
S2_18307270	2	18307270	7.32E-04	GL21_14/E2
S2_64550312	2	64550312	7.83E-04	GL7_14/E2
S2_12894427	2	12894427	8.43E-04	GL7_ac
S2_12894456	2	12894456	8.43E-04	GL7_ac
S2_12190474	2	12190474	8.54E-04	GL21_14/E2
S2_63290511	2	63290511	8.74E-04	GL42_ac
S2_63290512	2	63290512	8.74E-04	GL42_ac
S2_63290513	2	63290513	8.74E-04	GL42_ac
S2_63290514	2	63290514	8.74E-04	GL42_ac
S2_63290516	2	63290516	8.74E-04	GL42_ac
S2_63290517	2	63290517	8.74E-04	GL42_ac
S2_63290520	2	63290520	8.74E-04	GL42_ac
S2_63290523	2	63290523	8.74E-04	GL42_ac
S2_63290526	2	63290526	8.74E-04	GL42_ac
S2_63290527	2	63290527	8.74E-04	GL42_ac
S2_63290528	2	63290528	8.74E-04	GL42_ac
S2_63290533	2	63290533	8.74E-04	GL42_ac
S2_63290537	2	63290537	8.74E-04	GL42_ac
S2_65718904	2	65718904	9.23E-04	GL49_ac
S2_63182511	2	63182511	9.32E-04	GL14_14/E2
S2_13459053	2	13459053	9.61E-04	GL14_14/E2

SNP	Chromosome	Position	P.value	Trait
S2_5786269	2	5786269	9.99E-04	GL35_13/E1
S2_59249439	2	59249439	1.08E-03	GL35_14/E2
S2_59249432	2	59249432	1.08E-03	GL35_14/E2
S2_67463962	2	67463962	1.08E-03	GL21_13/E1
S2_16814404	2	16814404	1.08E-03	GL35_ac
S2_16814409	2	16814409	1.08E-03	GL35_ac
S2_62121657	2	62121657	1.19E-03	GL42_14/E2
S2_6277727	2	6277727	1.24E-03	GL28_13/E1
S2_6277742	2	6277742	1.24E-03	GL28_13/E1
S2_67977203	2	67977203	1.25E-03	GL7_14/E2
S2_65686094	2	65686094	1.29E-03	GL21_13/E1
S2_40623466	2	40623466	1.31E-03	GL14_ac
S2_6277727	2	6277727	1.32E-03	GL21_13/E1
S2_6277742	2	6277742	1.32E-03	GL21_13/E1
S2_6277727	2	6277727	1.33E-03	GL14_13/E1
S2_6277742	2	6277742	1.33E-03	GL14_13/E1
S2_67463962	2	67463962	1.33E-03	GL14_14/E2
S2_61686194	2	61686194	1.34E-03	GL42_14/E2
S2_61686229	2	61686229	1.34E-03	GL42_14/E2
S2_61686233	2	61686233	1.34E-03	GL42_14/E2
S2_6277727	2	6277727	1.35E-03	GL7_13/E1
S2_6277742	2	6277742	1.35E-03	GL7_13/E1
S2_58239206	2	58239206	1.37E-03	GL35_14/E2
S2_58239285	2	58239285	1.37E-03	GL35_14/E2
S2_65686094	2	65686094	1.47E-03	GL28_13/E1
S2_12346371	2	12346371	1.56E-03	GL7_ac
S2_63450777	2	63450777	1.59E-03	GL42_ac
S2_63450759	2	63450759	1.59E-03	GL42_ac
S2_63450765	2	63450765	1.59E-03	GL42_ac
S2_12346371	2	12346371	1.60E-03	GL7_14/E2
S2_53473251	2	53473251	1.63E-03	GL35_ac
S2_53473255	2	53473255	1.63E-03	GL35_ac
S2_8878734	2	8878734	1.66E-03	GL7_13/E1
S2_67463962	2	67463962	1.70E-03	GL28_13/E1
S2_8823410	2	8823410	1.71E-03	GL42_14/E2
S2_65686094	2	65686094	1.72E-03	GL14_13/E1
S2_199703	2	199703	1.73E-03	GL42_ac
S2_67206475	2	67206475	1.80E-03	GL49_14/E2
S2_47590004	2	47590004	1.81E-03	GL42_ac
S2_61586246	2	61586246	1.81E-03	GL35_14/E2
S2_61586256	2	61586256	1.81E-03	GL35_14/E2
S2_61586244	2	61586244	1.81E-03	GL35_14/E2
S2_57042267	2	57042267	1.82E-03	GL49_13/E1
S2_5786269	2	5786269	1.88E-03	GL28_13/E1
S2_53473251	2	53473251	1.92E-03	GL35_13/E1
S2_53473255	2	53473255	1.92E-03	GL35_13/E1
S2_14406452	2	14406452	1.92E-03	GL42_13/E1
S2_40623466	2	40623466	1.97E-03	GL21_ac
S2_6009191	2	6009191	1.98E-03	GL35_13/E1
S2_63692439	2	63692439	2.09E-03	GL7_14/E2
S2_61233514	2	61233514	2.10E-03	GL7_13/E1

SNP	Chromosome	Position	P.value	Trait
S2_61233514	2	61233514	2.10E-03	GL7_13/E1
S2_61233514	2	61233514	2.10E-03	GL7_13/E1
S2_61233514	2	61233514	2.10E-03	GL7_13/E1
S2_65686094	2	65686094	2.11E-03	GL28_ac
S2_63057180	2	63057180	2.12E-03	GL14_ac
S2_6245630	2	6245630	2.12E-03	GL49_13/E1
S2_66316757	2	66316757	2.12E-03	GL35_13/E1
S2_60856616	2	60856616	2.15E-03	GL28_14/E2
S2_55292148	2	55292148	2.17E-03	GL14_ac
S2_55292150	2	55292150	2.17E-03	GL14_ac
S2_55292147	2	55292147	2.17E-03	GL14_ac
S2_55292149	2	55292149	2.17E-03	GL14_ac
S2_13459053	2	13459053	2.18E-03	GL49_ac
S2_60724171	2	60724171	2.19E-03	GL7_13/E1
S2_12190474	2	12190474	2.20E-03	GL14_14/E2
S2_58239206	2	58239206	2.21E-03	GL21_14/E2
S2_58239285	2	58239285	2.21E-03	GL21_14/E2
S2_61733987	2	61733987	2.24E-03	GL42_14/E2
S2_4554748	2	4554748	2.26E-03	GL42_14/E2
S2_4554749	2	4554749	2.26E-03	GL42_14/E2
S2_65095903	2	65095903	2.26E-03	GL28_14/E2
S2_60724171	2	60724171	2.26E-03	GL21_13/E1
S2_53473251	2	53473251	2.28E-03	GL49_13/E1
S2_53473255	2	53473255	2.28E-03	GL49_13/E1
S2_58008723	2	58008723	2.29E-03	GL21_ac
S2_58614106	2	58614106	2.32E-03	GL42_ac
S2_58614110	2	58614110	2.32E-03	GL42_ac
S2_6523542	2	6523542	2.34E-03	GL21_14/E2
S2_6523564	2	6523564	2.34E-03	GL21_14/E2
S2_60856616	2	60856616	2.37E-03	GL14_14/E2
S2_40623466	2	40623466	2.42E-03	GL35_ac
S2_12894427	2	12894427	2.46E-03	GL49_14/E2
S2_12894456	2	12894456	2.46E-03	GL49_14/E2
S2_59249439	2	59249439	2.50E-03	GL21_14/E2
S2_59249432	2	59249432	2.50E-03	GL21_14/E2
S2_69200414	2	69200414	2.51E-03	GL35_14/E2
S2_73374818	2	73374818	2.52E-03	GL21_13/E1
S2_7342926	2	7342926	2.57E-03	GL42_ac
S2_6277727	2	6277727	2.59E-03	GL35_13/E1
S2_6277742	2	6277742	2.59E-03	GL35_13/E1
S2_64335513	2	64335513	2.60E-03	GL49_14/E2
S2_64335542	2	64335542	2.60E-03	GL49_14/E2
S2_16814404	2	16814404	2.61E-03	GL7_13/E1
S2_16814409	2	16814409	2.61E-03	GL7_13/E1
S2_6218390	2	6218390	2.61E-03	GL7_ac
S2_67463962	2	67463962	2.63E-03	GL7_13/E1
S2_72273733	2	72273733	2.63E-03	GL28_13/E1
S2_2679997	2	2679997	2.64E-03	GL28_14/E2
S2_61733987	2	61733987	2.67E-03	GL42_ac
S2_59655524	2	59655524	2.68E-03	GL49_14/E2
S2_59655550	2	59655550	2.68E-03	GL49_14/E2

SNP	Chromosome	Position	P.value	Trait
S2_68505778	2	68505778	2.69E-03	GL49_14/E2
S2_64335513	2	64335513	2.69E-03	GL42_14/E2
S2_64335542	2	64335542	2.69E-03	GL42_14/E2
S2_6218390	2	6218390	2.72E-03	GL28_14/E2
S2_60724171	2	60724171	2.72E-03	GL28_13/E1
S2_45863094	2	45863094	2.74E-03	GL14_13/E1
S2_66958051	2	66958051	2.75E-03	GL21_14/E2
S2_66958078	2	66958078	2.75E-03	GL21_14/E2
S2_66958081	2	66958081	2.75E-03	GL21_14/E2
S2_18307240	2	18307240	2.78E-03	GL14_14/E2
S2_18307256	2	18307256	2.78E-03	GL14_14/E2
S2_18307270	2	18307270	2.78E-03	GL14_14/E2
S2_71486250	2	71486250	2.79E-03	GL35_13/E1
S2_12894427	2	12894427	2.82E-03	GL7_14/E2
S2_12894456	2	12894456	2.82E-03	GL7_14/E2
S2_65359876	2	65359876	2.86E-03	GL49_14/E2
S2_65359832	2	65359832	2.86E-03	GL49_14/E2
S2_58239206	2	58239206	2.86E-03	GL14_14/E2
S2_58239285	2	58239285	2.86E-03	GL14_14/E2
S2_73374818	2	73374818	2.88E-03	GL35_13/E1
S2_4820725	2	4820725	2.92E-03	GL42_13/E1
S2_41611737	2	41611737	2.97E-03	GL14_ac
S2_76430887	2	76430887	2.98E-03	GL28_14/E2
S2_76430894	2	76430894	2.98E-03	GL28_14/E2
S2_76430889	2	76430889	2.98E-03	GL28_14/E2
S2_76430891	2	76430891	2.98E-03	GL28_14/E2
S2_76430893	2	76430893	2.98E-03	GL28_14/E2
S2_76430895	2	76430895	2.98E-03	GL28_14/E2
S2_61686194	2	61686194	2.99E-03	GL35_14/E2
S2_61686229	2	61686229	2.99E-03	GL35_14/E2
S2_61686233	2	61686233	2.99E-03	GL35_14/E2
S2_73374792	2	73374792	3.00E-03	GL35_13/E1
S2_69124081	2	69124081	3.08E-03	GL7_13/E1
S2_27054650	2	27054650	3.08E-03	GL42_13/E1
S2_12894427	2	12894427	3.12E-03	GL28_ac
S2_12894456	2	12894456	3.12E-03	GL28_ac
S2_63182511	2	63182511	3.15E-03	GL21_14/E2
S2_75594134	2	75594134	3.18E-03	GL42_13/E1
S2_75594135	2	75594135	3.18E-03	GL42_13/E1
S2_60709420	2	60709420	3.20E-03	GL49_ac
S2_12686859	2	12686859	3.21E-03	GL42_13/E1
S2_12686875	2	12686875	3.21E-03	GL42_13/E1
S2_12686896	2	12686896	3.21E-03	GL42_13/E1
S2_61873327	2	61873327	3.22E-03	GL42_14/E2
S2_16814404	2	16814404	3.23E-03	GL28_ac
S2_16814409	2	16814409	3.23E-03	GL28_ac
S2_67463962	2	67463962	3.23E-03	GL49_13/E1
S2_63357026	2	63357026	3.23E-03	GL49_13/E1
S2_10001930	2	10001930	3.23E-03	GL7_ac
S2_15077590	2	15077590	3.23E-03	GL35_ac
S2_15077622	2	15077622	3.23E-03	GL35_ac

SNP	Chromosome	Position	P.value	Trait
S2_5786269	2	5786269	3.27E-03	GL49_13/E1
S2_60709429	2	60709429	3.28E-03	GL49_ac
S2_73813174	2	73813174	3.28E-03	GL42_ac
S2_50985085	2	50985085	3.28E-03	GL35_14/E2
S2_36306432	2	36306432	3.29E-03	GL7_13/E1
S2_65686094	2	65686094	3.31E-03	GL21_ac
S2_7531999	2	7531999	3.32E-03	GL35_13/E1
S2_61505405	2	61505405	3.32E-03	GL35_14/E2
S2_56904254	2	56904254	3.33E-03	GL35_14/E2
S2_56904274	2	56904274	3.33E-03	GL35_14/E2
S2_56904295	2	56904295	3.33E-03	GL35_14/E2
S2_67206475	2	67206475	3.34E-03	GL28_14/E2
S2_67952247	2	67952247	3.37E-03	GL28_14/E2
S2_54461667	2	54461667	3.37E-03	GL42_ac
S2_12894427	2	12894427	3.37E-03	GL35_ac
S2_12894456	2	12894456	3.37E-03	GL35_ac
S2_10001930	2	10001930	3.37E-03	GL7_13/E1
S2_57449109	2	57449109	3.37E-03	GL7_13/E1
S2_75792059	2	75792059	3.37E-03	GL14_14/E2
S2_75792053	2	75792053	3.37E-03	GL14_14/E2
S2_58008723	2	58008723	3.39E-03	GL35_13/E1
S2_55524388	2	55524388	3.40E-03	GL28_14/E2
S2_73374818	2	73374818	3.49E-03	GL28_13/E1
S2_16814404	2	16814404	3.50E-03	GL49_13/E1
S2_16814409	2	16814409	3.50E-03	GL49_13/E1
S2_63803728	2	63803728	3.51E-03	GL21_14/E2
S2_73374792	2	73374792	3.52E-03	GL21_13/E1
S2_4555276	2	4555276	3.54E-03	GL42_13/E1
S2_4555285	2	4555285	3.54E-03	GL42_13/E1
S2_62117918	2	62117918	3.54E-03	GL14_ac
S2_67463962	2	67463962	3.55E-03	GL14_13/E1
S2_69124081	2	69124081	3.55E-03	GL49_14/E2
S2_13459053	2	13459053	3.57E-03	GL28_14/E2
S2_12894427	2	12894427	3.61E-03	GL28_14/E2
S2_12894456	2	12894456	3.61E-03	GL28_14/E2
S2_12190474	2	12190474	3.63E-03	GL28_14/E2
S2_69124081	2	69124081	3.65E-03	GL7_14/E2
S2_62117918	2	62117918	3.66E-03	GL7_ac
S2_67463962	2	67463962	3.68E-03	GL7_14/E2
S2_62117918	2	62117918	3.69E-03	GL21_ac
S2_5685669	2	5685669	3.70E-03	GL42_13/E1
S2_64368069	2	64368069	3.71E-03	GL7_14/E2
S2_16814404	2	16814404	3.73E-03	GL14_ac
S2_16814409	2	16814409	3.73E-03	GL14_ac
S2_16041893	2	16041893	3.78E-03	GL21_14/E2
S2_57558184	2	57558184	3.79E-03	GL21_13/E1
S2_9780495	2	9780495	3.80E-03	GL49_13/E1
S2_60724171	2	60724171	3.85E-03	GL14_13/E1
S2_6612036	2	6612036	3.85E-03	GL21_ac
S2_65357280	2	65357280	3.88E-03	GL35_14/E2
S2_3807228	2	3807228	3.90E-03	GL21_ac

SNP	Chromosome	Position	P.value	Trait
S2_45863094	2	45863094	3.92E-03	GL7_ac
S2_58182637	2	58182637	3.93E-03	GL7_14/E2
S2_16041893	2	16041893	3.94E-03	GL28_14/E2
S2_30234091	2	30234091	3.95E-03	GL14_14/E2
S2_30234092	2	30234092	3.95E-03	GL14_14/E2
S2_30234097	2	30234097	3.95E-03	GL14_14/E2
S2_30234114	2	30234114	3.95E-03	GL14_14/E2
S2_61686194	2	61686194	3.98E-03	GL42_ac
S2_61686229	2	61686229	3.98E-03	GL42_ac
S2_61686233	2	61686233	3.98E-03	GL42_ac
S2_54461667	2	54461667	4.00E-03	GL35_ac
S2_16041893	2	16041893	4.04E-03	GL14_14/E2
S2_65686094	2	65686094	4.11E-03	GL7_13/E1
S2_13809628	2	13809628	4.12E-03	GL42_13/E1
S2_13809634	2	13809634	4.12E-03	GL42_13/E1
S2_63290511	2	63290511	4.14E-03	GL42_14/E2
S2_63290512	2	63290512	4.14E-03	GL42_14/E2
S2_63290513	2	63290513	4.14E-03	GL42_14/E2
S2_63290514	2	63290514	4.14E-03	GL42_14/E2
S2_63290516	2	63290516	4.14E-03	GL42_14/E2
S2_63290517	2	63290517	4.14E-03	GL42_14/E2
S2_63290520	2	63290520	4.14E-03	GL42_14/E2
S2_63290523	2	63290523	4.14E-03	GL42_14/E2
S2_63290526	2	63290526	4.14E-03	GL42_14/E2
S2_63290527	2	63290527	4.14E-03	GL42_14/E2
S2_63290528	2	63290528	4.14E-03	GL42_14/E2
S2_63290533	2	63290533	4.14E-03	GL42_14/E2
S2_63290537	2	63290537	4.14E-03	GL42_14/E2
S2_16814404	2	16814404	4.15E-03	GL14_13/E1
S2_16814409	2	16814409	4.15E-03	GL14_13/E1
S2_73740888	2	73740888	4.16E-03	GL42_ac
S2_57264320	2	57264320	4.17E-03	GL49_13/E1
S2_57264322	2	57264322	4.17E-03	GL49_13/E1
S2_47590004	2	47590004	4.19E-03	GL28_13/E1
S2_62095251	2	62095251	4.23E-03	GL14_14/E2
S2_12190474	2	12190474	4.25E-03	GL35_ac
S2_72273733	2	72273733	4.25E-03	GL35_ac
S2_56904254	2	56904254	4.30E-03	GL14_14/E2
S2_56904274	2	56904274	4.30E-03	GL14_14/E2
S2_56904295	2	56904295	4.30E-03	GL14_14/E2
S2_8878734	2	8878734	4.30E-03	GL14_13/E1
S2_47204245	2	47204245	4.31E-03	GL42_14/E2
S2_61233514	2	61233514	4.34E-03	GL7_ac
S2_61233514	2	61233514	4.34E-03	GL7_ac
S2_61233514	2	61233514	4.34E-03	GL7_ac
S2_61233514	2	61233514	4.34E-03	GL7_ac
S2_76430887	2	76430887	4.34E-03	GL7_13/E1
S2_76430889	2	76430889	4.34E-03	GL7_13/E1
S2_76430891	2	76430891	4.34E-03	GL7_13/E1
S2_76430893	2	76430893	4.34E-03	GL7_13/E1
S2_76430894	2	76430894	4.34E-03	GL7_13/E1

SNP	Chromosome	Position	P.value	Trait
S2_76430895	2	76430895	4.34E-03	GL7_13/E1
S2_58875658	2	58875658	4.34E-03	GL49_ac
S2_64464930	2	64464930	4.35E-03	GL49_14/E2
S2_64464938	2	64464938	4.35E-03	GL49_14/E2
S2_60709420	2	60709420	4.36E-03	GL35_ac
S2_52993647	2	52993647	4.37E-03	GL7_14/E2
S2_60724171	2	60724171	4.39E-03	GL35_13/E1
S2_63803728	2	63803728	4.39E-03	GL49_14/E2
S2_6009191	2	6009191	4.43E-03	GL42_13/E1
S2_40623466	2	40623466	4.46E-03	GL35_13/E1
S2_69572060	2	69572060	4.53E-03	GL49_14/E2
S2_35154563	2	35154563	4.54E-03	GL35_14/E2
S2_63803726	2	63803726	4.55E-03	GL49_14/E2
S2_63803729	2	63803729	4.55E-03	GL49_14/E2
S2_63803722	2	63803722	4.55E-03	GL49_14/E2
S2_16814404	2	16814404	4.60E-03	GL21_ac
S2_16814409	2	16814409	4.60E-03	GL21_ac
S2_60856616	2	60856616	4.62E-03	GL49_ac
S2_13505785	2	13505785	4.65E-03	GL21_14/E2
S2_67620075	2	67620075	4.68E-03	GL28_13/E1
S2_61233402	2	61233402	4.72E-03	GL35_14/E2
S2_63057180	2	63057180	4.73E-03	GL35_13/E1
S2_49669484	2	49669484	4.73E-03	GL35_14/E2
S2_49669489	2	49669489	4.73E-03	GL35_14/E2
S2_69130242	2	69130242	4.75E-03	GL21_14/E2
S2_5786269	2	5786269	4.76E-03	GL21_13/E1
S2_18309037	2	18309037	4.78E-03	GL28_ac
S2_8937547	2	8937547	4.80E-03	GL49_14/E2
S2_61233514	2	61233514	4.81E-03	GL14_13/E1
S2_61233514	2	61233514	4.81E-03	GL14_13/E1
S2_61233514	2	61233514	4.81E-03	GL14_13/E1
S2_61233514	2	61233514	4.81E-03	GL14_13/E1
S2_66956243	2	66956243	4.81E-03	GL14_13/E1
S2_61733986	2	61733986	4.81E-03	GL42_ac
S2_61733993	2	61733993	4.81E-03	GL42_ac
S2_58008723	2	58008723	4.83E-03	GL42_13/E1
S2_67463962	2	67463962	4.86E-03	GL35_ac
S2_35154563	2	35154563	4.87E-03	GL42_14/E2
S2_36306432	2	36306432	4.87E-03	GL14_13/E1
S2_3807228	2	3807228	4.90E-03	GL21_14/E2
S2_66035257	2	66035257	4.91E-03	GL7_14/E2
S2_61266191	2	61266191	4.93E-03	GL14_14/E2
S2_61266192	2	61266192	4.93E-03	GL14_14/E2
S2_8154762	2	8154762	4.93E-03	GL42_14/E2
S2_6277727	2	6277727	4.94E-03	GL42_13/E1
S2_6277742	2	6277742	4.94E-03	GL42_13/E1
S2_6009191	2	6009191	4.96E-03	GL28_13/E1
S2_63057180	2	63057180	4.96E-03	GL49_13/E1
S2_75331009	2	75331009	4.97E-03	GL35_14/E2
S2_27054650	2	27054650	5.00E-03	GL28_13/E1
S2_72260776	2	72260776	5.01E-03	GL42_ac

SNP	Chromosome	Position	P.value	Trait
S2_72260818	2	72260818	5.01E-03	GL42_ac
S2_69237155	2	69237155	5.02E-03	GL21_14/E2
S2_60709420	2	60709420	5.02E-03	GL14_14/E2
S2_10906987	2	10906987	5.04E-03	GL7_ac
S2_66830583	2	66830583	5.06E-03	GL21_14/E2
S2_62095158	2	62095158	5.07E-03	GL35_ac
S2_62095147	2	62095147	5.07E-03	GL35_ac
S2_67463962	2	67463962	5.08E-03	GL49_ac
S2_57834823	2	57834823	5.09E-03	GL28_13/E1
S2_73374792	2	73374792	5.12E-03	GL28_13/E1
S2_66074828	2	66074828	5.13E-03	GL49_13/E1
S2_65686094	2	65686094	5.13E-03	GL49_13/E1
S2_14406452	2	14406452	5.13E-03	GL35_13/E1
S2_6245630	2	6245630	5.13E-03	GL42_13/E1
S2_63195176	2	63195176	5.14E-03	GL28_14/E2
S2_65877733	2	65877733	5.15E-03	GL28_14/E2
S2_68505778	2	68505778	5.16E-03	GL42_ac
S2_61841943	2	61841943	5.19E-03	GL28_ac
S2_60856616	2	60856616	5.21E-03	GL21_13/E1
S2_18307240	2	18307240	5.22E-03	GL21_ac
S2_18307256	2	18307256	5.22E-03	GL21_ac
S2_18307270	2	18307270	5.22E-03	GL21_ac
S2_67463962	2	67463962	5.26E-03	GL42_13/E1
S2_67206475	2	67206475	5.26E-03	GL35_ac
S2_25873582	2	25873582	5.26E-03	GL35_ac
S2_25873657	2	25873657	5.26E-03	GL35_ac
S2_25873671	2	25873671	5.26E-03	GL35_ac
S2_69593580	2	69593580	5.26E-03	GL7_14/E2
S2_59655484	2	59655484	5.30E-03	GL14_ac
S2_61686625	2	61686625	5.32E-03	GL35_ac
S2_16009255	2	16009255	5.32E-03	GL28_14/E2
S2_61686194	2	61686194	5.32E-03	GL35_ac
S2_61686229	2	61686229	5.32E-03	GL35_ac
S2_61686233	2	61686233	5.32E-03	GL35_ac
S2_55292148	2	55292148	5.34E-03	GL14_14/E2
S2_55292150	2	55292150	5.34E-03	GL14_14/E2
S2_55292147	2	55292147	5.34E-03	GL14_14/E2
S2_55292149	2	55292149	5.34E-03	GL14_14/E2
S2_61841943	2	61841943	5.35E-03	GL21_13/E1
S2_63078122	2	63078122	5.36E-03	GL35_ac
S2_69124081	2	69124081	5.38E-03	GL21_13/E1
S2_63057180	2	63057180	5.39E-03	GL21_ac
S2_13459050	2	13459050	5.39E-03	GL14_ac
S2_2679997	2	2679997	5.41E-03	GL21_14/E2
S2_56196270	2	56196270	5.44E-03	GL35_14/E2
S2_66316757	2	66316757	5.51E-03	GL28_13/E1
S2_59655484	2	59655484	5.51E-03	GL7_ac
S2_16041893	2	16041893	5.54E-03	GL7_14/E2
S2_69124081	2	69124081	5.56E-03	GL14_13/E1
S2_38294461	2	38294461	5.60E-03	GL49_ac
S2_63078122	2	63078122	5.60E-03	GL49_ac

SNP	Chromosome	Position	P.value	Trait
S2_74969570	2	74969570	5.62E-03	GL42_13/E1
S2_57042267	2	57042267	5.62E-03	GL14_14/E2
S2_69237155	2	69237155	5.64E-03	GL28_14/E2
S2_67620075	2	67620075	5.67E-03	GL7_13/E1
S2_36306432	2	36306432	5.68E-03	GL28_ac
S2_4863239	2	4863239	5.71E-03	GL49_13/E1
S2_63057180	2	63057180	5.73E-03	GL28_ac
S2_69130242	2	69130242	5.73E-03	GL14_14/E2
S2_61733986	2	61733986	5.73E-03	GL42_14/E2
S2_61733993	2	61733993	5.73E-03	GL42_14/E2
S2_61841943	2	61841943	5.74E-03	GL21_ac
S2_29767393	2	29767393	5.76E-03	GL7_13/E1
S2_29767401	2	29767401	5.76E-03	GL7_13/E1
S2_50985085	2	50985085	5.82E-03	GL35_ac
S2_6009191	2	6009191	5.84E-03	GL35_ac
S2_425915	2	425915	5.85E-03	GL42_14/E2
S2_65784827	2	65784827	5.86E-03	GL21_14/E2
S2_65784818	2	65784818	5.86E-03	GL21_14/E2
S2_65784823	2	65784823	5.86E-03	GL21_14/E2
S2_56439200	2	56439200	5.89E-03	GL7_13/E1
S2_73374792	2	73374792	5.90E-03	GL42_13/E1
S2_63290511	2	63290511	5.91E-03	GL49_ac
S2_63290512	2	63290512	5.91E-03	GL49_ac
S2_63290513	2	63290513	5.91E-03	GL49_ac
S2_63290514	2	63290514	5.91E-03	GL49_ac
S2_63290516	2	63290516	5.91E-03	GL49_ac
S2_63290517	2	63290517	5.91E-03	GL49_ac
S2_63290520	2	63290520	5.91E-03	GL49_ac
S2_63290523	2	63290523	5.91E-03	GL49_ac
S2_63290526	2	63290526	5.91E-03	GL49_ac
S2_63290527	2	63290527	5.91E-03	GL49_ac
S2_63290528	2	63290528	5.91E-03	GL49_ac
S2_63290533	2	63290533	5.91E-03	GL49_ac
S2_63290537	2	63290537	5.91E-03	GL49_ac
S2_65784827	2	65784827	5.93E-03	GL28_14/E2
S2_65784818	2	65784818	5.93E-03	GL28_14/E2
S2_65784823	2	65784823	5.93E-03	GL28_14/E2
S2_38860433	2	38860433	5.94E-03	GL35_14/E2
S2_57258389	2	57258389	5.96E-03	GL14_14/E2
S2_57258426	2	57258426	5.96E-03	GL14_14/E2
S2_57258429	2	57258429	5.96E-03	GL14_14/E2
S2_67977203	2	67977203	5.98E-03	GL28_14/E2
S2_61091712	2	61091712	6.00E-03	GL7_ac
S2_66074828	2	66074828	6.00E-03	GL21_13/E1
S2_67620075	2	67620075	6.01E-03	GL35_13/E1
S2_13459059	2	13459059	6.03E-03	GL14_ac
S2_62121657	2	62121657	6.04E-03	GL49_14/E2
S2_64391996	2	64391996	6.05E-03	GL28_13/E1
S2_60406949	2	60406949	6.06E-03	GL7_14/E2
S2_16814404	2	16814404	6.06E-03	GL7_ac
S2_16814409	2	16814409	6.06E-03	GL7_ac

SNP	Chromosome	Position	P.value	Trait
S2_55524388	2	55524388	6.13E-03	GL21_14/E2
S2_58008723	2	58008723	6.17E-03	GL28_ac
S2_18307240	2	18307240	6.19E-03	GL21_13/E1
S2_18307256	2	18307256	6.19E-03	GL21_13/E1
S2_18307270	2	18307270	6.19E-03	GL21_13/E1
S2_12190474	2	12190474	6.23E-03	GL28_ac
S2_13459053	2	13459053	6.25E-03	GL14_ac
S2_60709429	2	60709429	6.25E-03	GL35_ac
S2_6008867	2	6008867	6.29E-03	GL42_13/E1
S2_61686194	2	61686194	6.30E-03	GL28_14/E2
S2_61686229	2	61686229	6.30E-03	GL28_14/E2
S2_61686233	2	61686233	6.30E-03	GL28_14/E2
S2_67463962	2	67463962	6.31E-03	GL42_ac
S2_67620075	2	67620075	6.33E-03	GL21_13/E1
S2_25873582	2	25873582	6.35E-03	GL7_14/E2
S2_25873657	2	25873657	6.35E-03	GL7_14/E2
S2_25873671	2	25873671	6.35E-03	GL7_14/E2
S2_72273733	2	72273733	6.35E-03	GL28_ac
S2_18921102	2	18921102	6.36E-03	GL42_13/E1
S2_75333122	2	75333122	6.37E-03	GL42_ac
S2_61537476	2	61537476	6.37E-03	GL35_ac
S2_67620075	2	67620075	6.39E-03	GL49_13/E1
S2_72260776	2	72260776	6.42E-03	GL42_13/E1
S2_72260818	2	72260818	6.42E-03	GL42_13/E1
S2_41611737	2	41611737	6.42E-03	GL14_13/E1
S2_56904254	2	56904254	6.55E-03	GL21_14/E2
S2_56904274	2	56904274	6.55E-03	GL21_14/E2
S2_56904295	2	56904295	6.55E-03	GL21_14/E2
S2_6245657	2	6245657	6.59E-03	GL42_14/E2
S2_6245664	2	6245664	6.59E-03	GL42_14/E2
S2_6245665	2	6245665	6.59E-03	GL42_14/E2
S2_75333122	2	75333122	6.59E-03	GL35_ac
S2_58500920	2	58500920	6.61E-03	GL42_13/E1
S2_54461667	2	54461667	6.63E-03	GL14_ac
S2_59645184	2	59645184	6.63E-03	GL42_ac
S2_61105192	2	61105192	6.64E-03	GL7_14/E2
S2_6612036	2	6612036	6.65E-03	GL14_ac
S2_69173961	2	69173961	6.66E-03	GL7_14/E2
S2_10906987	2	10906987	6.74E-03	GL28_14/E2
S2_6612036	2	6612036	6.74E-03	GL28_ac
S2_63357026	2	63357026	6.77E-03	GL42_13/E1
S2_62139142	2	62139142	6.78E-03	GL35_13/E1
S2_9370623	2	9370623	6.78E-03	GL7_14/E2
S2_62484730	2	62484730	6.79E-03	GL49_14/E2
S2_60533933	2	60533933	6.79E-03	GL42_13/E1
S2_60533933	2	60533933	6.79E-03	GL42_13/E1
S2_60533933	2	60533933	6.79E-03	GL42_13/E1
S2_58286606	2	58286606	6.79E-03	GL14_13/E1
S2_67977203	2	67977203	6.80E-03	GL35_14/E2
S2_6042431	2	6042431	6.84E-03	GL42_13/E1
S2_66316757	2	66316757	6.84E-03	GL21_13/E1

SNP	Chromosome	Position	P.value	Trait
S2_14746117	2	14746117	6.85E-03	GL21_14/E2
S2_36306432	2	36306432	6.87E-03	GL21_13/E1
S2_3807228	2	3807228	6.91E-03	GL28_ac
S2_67977203	2	67977203	6.95E-03	GL35_ac
S2_75792059	2	75792059	6.96E-03	GL49_ac
S2_75792053	2	75792053	6.96E-03	GL49_ac
S2_72260776	2	72260776	6.96E-03	GL35_13/E1
S2_72260818	2	72260818	6.96E-03	GL35_13/E1
S2_3216766	2	3216766	6.97E-03	GL42_14/E2
S2_63450777	2	63450777	6.98E-03	GL35_ac
S2_63450759	2	63450759	6.98E-03	GL35_ac
S2_63450765	2	63450765	6.98E-03	GL35_ac
S2_67463962	2	67463962	6.98E-03	GL14_ac
S2_62703717	2	62703717	7.01E-03	GL35_ac
S2_12894427	2	12894427	7.03E-03	GL42_ac
S2_12894456	2	12894456	7.03E-03	GL42_ac
S2_58428674	2	58428674	7.03E-03	GL28_13/E1
S2_58875658	2	58875658	7.06E-03	GL49_14/E2
S2_53473251	2	53473251	7.07E-03	GL28_13/E1
S2_53473255	2	53473255	7.07E-03	GL28_13/E1
S2_73412146	2	73412146	7.07E-03	GL49_ac
S2_76908521	2	76908521	7.10E-03	GL49_ac
S2_76908526	2	76908526	7.10E-03	GL49_ac
S2_76908527	2	76908527	7.10E-03	GL49_ac
S2_65650443	2	65650443	7.10E-03	GL49_14/E2
S2_57558184	2	57558184	7.11E-03	GL49_13/E1
S2_60406949	2	60406949	7.15E-03	GL35_14/E2
S2_18309037	2	18309037	7.16E-03	GL42_ac
S2_73888705	2	73888705	7.18E-03	GL14_ac
S2_73813174	2	73813174	7.21E-03	GL35_13/E1
S2_49637033	2	49637033	7.21E-03	GL49_13/E1
S2_61091712	2	61091712	7.22E-03	GL21_ac
S2_66316757	2	66316757	7.24E-03	GL42_13/E1
S2_57848126	2	57848126	7.24E-03	GL35_13/E1
S2_30234091	2	30234091	7.24E-03	GL42_14/E2
S2_30234092	2	30234092	7.24E-03	GL42_14/E2
S2_30234097	2	30234097	7.24E-03	GL42_14/E2
S2_30234114	2	30234114	7.24E-03	GL42_14/E2
S2_60709420	2	60709420	7.27E-03	GL14_ac
S2_18309037	2	18309037	7.31E-03	GL7_ac
S2_14789653	2	14789653	7.38E-03	GL21_ac
S2_65686094	2	65686094	7.38E-03	GL35_13/E1
S2_67656785	2	67656785	7.38E-03	GL49_13/E1
S2_73813174	2	73813174	7.40E-03	GL42_13/E1
S2_67206475	2	67206475	7.44E-03	GL35_14/E2
S2_59722587	2	59722587	7.45E-03	GL28_14/E2
S2_73739659	2	73739659	7.46E-03	GL14_13/E1
S2_73739698	2	73739698	7.46E-03	GL14_13/E1
S2_62631187	2	62631187	7.46E-03	GL49_14/E2
S2_14406452	2	14406452	7.47E-03	GL28_13/E1
S2_16848234	2	16848234	7.47E-03	GL35_13/E1

SNP	Chromosome	Position	P.value	Trait
S2_4334298	2	4334298	7.53E-03	GL14_ac
S2_6612036	2	6612036	7.53E-03	GL35_13/E1
S2_36306432	2	36306432	7.56E-03	GL21_ac
S2_7531999	2	7531999	7.59E-03	GL28_13/E1
S2_61505405	2	61505405	7.59E-03	GL21_14/E2
S2_47590004	2	47590004	7.61E-03	GL21_13/E1
S2_18569274	2	18569274	7.63E-03	GL35_13/E1
S2_40623466	2	40623466	7.63E-03	GL21_13/E1
S2_62631187	2	62631187	7.64E-03	GL28_14/E2
S2_73740884	2	73740884	7.65E-03	GL42_ac
S2_73740883	2	73740883	7.65E-03	GL42_ac
S2_64531149	2	64531149	7.67E-03	GL49_14/E2
S2_67368464	2	67368464	7.69E-03	GL49_13/E1
S2_9529733	2	9529733	7.75E-03	GL21_ac
S2_6236103	2	6236103	7.78E-03	GL35_14/E2
S2_59249439	2	59249439	7.78E-03	GL35_ac
S2_59249432	2	59249432	7.78E-03	GL35_ac
S2_62631187	2	62631187	7.78E-03	GL42_14/E2
S2_49662976	2	49662976	7.80E-03	GL21_14/E2
S2_40623466	2	40623466	7.80E-03	GL42_13/E1
S2_6008867	2	6008867	7.80E-03	GL35_13/E1
S2_27054650	2	27054650	7.84E-03	GL35_13/E1
S2_30114663	2	30114663	7.87E-03	GL35_14/E2
S2_14406452	2	14406452	7.88E-03	GL49_13/E1
S2_18309037	2	18309037	7.88E-03	GL21_ac
S2_73374818	2	73374818	7.88E-03	GL42_13/E1
S2_61927663	2	61927663	7.91E-03	GL42_14/E2
S2_61686194	2	61686194	7.95E-03	GL14_14/E2
S2_61686229	2	61686229	7.95E-03	GL14_14/E2
S2_61686233	2	61686233	7.95E-03	GL14_14/E2
S2_61505405	2	61505405	7.95E-03	GL14_14/E2
S2_71254462	2	71254462	7.96E-03	GL21_14/E2
S2_57558184	2	57558184	7.96E-03	GL28_13/E1
S2_12190474	2	12190474	7.96E-03	GL35_13/E1
S2_58614106	2	58614106	7.98E-03	GL49_ac
S2_58614110	2	58614110	7.98E-03	GL49_ac
S2_76430887	2	76430887	7.98E-03	GL7_ac
S2_76430894	2	76430894	7.98E-03	GL7_ac
S2_76430889	2	76430889	7.98E-03	GL7_ac
S2_76430891	2	76430891	7.98E-03	GL7_ac
S2_76430893	2	76430893	7.98E-03	GL7_ac
S2_76430895	2	76430895	7.98E-03	GL7_ac
S2_61233514	2	61233514	8.02E-03	GL35_13/E1
S2_61233514	2	61233514	8.02E-03	GL35_13/E1
S2_61233514	2	61233514	8.02E-03	GL35_13/E1
S2_61233514	2	61233514	8.02E-03	GL35_13/E1
S2_56439200	2	56439200	8.02E-03	GL49_ac
S2_6277727	2	6277727	8.03E-03	GL21_ac
S2_6277742	2	6277742	8.03E-03	GL21_ac
S2_62095158	2	62095158	8.03E-03	GL28_ac
S2_62095147	2	62095147	8.03E-03	GL28_ac

SNP	Chromosome	Position	P.value	Trait
S2_54461667	2	54461667	8.04E-03	GL49_13/E1
S2_62095158	2	62095158	8.07E-03	GL35_14/E2
S2_62095147	2	62095147	8.07E-03	GL35_14/E2
S2_74389504	2	74389504	8.07E-03	GL7_14/E2
S2_61246561	2	61246561	8.07E-03	GL42_13/E1
S2_52997657	2	52997657	8.08E-03	GL35_ac
S2_65854375	2	65854375	8.12E-03	GL42_14/E2
S2_18309037	2	18309037	8.12E-03	GL35_13/E1
S2_5685669	2	5685669	8.13E-03	GL35_13/E1
S2_75220306	2	75220306	8.15E-03	GL35_13/E1
S2_60432744	2	60432744	8.21E-03	GL42_ac
S2_4334291	2	4334291	8.21E-03	GL14_ac
S2_6245630	2	6245630	8.23E-03	GL28_13/E1
S2_66035257	2	66035257	8.24E-03	GL35_14/E2
S2_5786269	2	5786269	8.25E-03	GL21_ac
S2_65386069	2	65386069	8.25E-03	GL7_14/E2
S2_63803726	2	63803726	8.26E-03	GL21_14/E2
S2_63803729	2	63803729	8.26E-03	GL21_14/E2
S2_63803722	2	63803722	8.26E-03	GL21_14/E2
S2_61708630	2	61708630	8.26E-03	GL49_13/E1
S2_72273733	2	72273733	8.28E-03	GL21_13/E1
S2_61548986	2	61548986	8.31E-03	GL42_ac
S2_61548987	2	61548987	8.31E-03	GL42_ac
S2_66078977	2	66078977	8.34E-03	GL7_14/E2
S2_68505778	2	68505778	8.38E-03	GL35_14/E2
S2_10906987	2	10906987	8.39E-03	GL14_ac
S2_58614106	2	58614106	8.41E-03	GL7_ac
S2_58614110	2	58614110	8.41E-03	GL7_ac
S2_10906987	2	10906987	8.41E-03	GL28_ac
S2_71490218	2	71490218	8.42E-03	GL14_14/E2
S2_61923353	2	61923353	8.46E-03	GL35_ac
S2_56515124	2	56515124	8.47E-03	GL49_ac
S2_7531999	2	7531999	8.49E-03	GL21_13/E1
S2_57728708	2	57728708	8.50E-03	GL35_ac
S2_14406452	2	14406452	8.54E-03	GL21_13/E1
S2_5685669	2	5685669	8.57E-03	GL28_13/E1
S2_40623466	2	40623466	8.58E-03	GL28_ac
S2_61505405	2	61505405	8.59E-03	GL35_ac
S2_6009965	2	6009965	8.59E-03	GL42_13/E1
S2_41150065	2	41150065	8.60E-03	GL7_ac
S2_25873582	2	25873582	8.61E-03	GL35_14/E2
S2_25873657	2	25873657	8.61E-03	GL35_14/E2
S2_25873671	2	25873671	8.61E-03	GL35_14/E2
S2_77444031	2	77444031	8.63E-03	GL49_13/E1
S2_56904254	2	56904254	8.64E-03	GL28_14/E2
S2_56904274	2	56904274	8.64E-03	GL28_14/E2
S2_56904295	2	56904295	8.64E-03	GL28_14/E2
S2_65877733	2	65877733	8.64E-03	GL28_13/E1
S2_65686094	2	65686094	8.66E-03	GL28_14/E2
S2_61091712	2	61091712	8.67E-03	GL7_13/E1
S2_69593580	2	69593580	8.69E-03	GL35_14/E2

SNP	Chromosome	Position	P.value	Trait
S2_67205749	2	67205749	8.69E-03	GL21_14/E2
S2_67205751	2	67205751	8.69E-03	GL21_14/E2
S2_67205757	2	67205757	8.69E-03	GL21_14/E2
S2_57834823	2	57834823	8.70E-03	GL49_13/E1
S2_65686094	2	65686094	8.72E-03	GL14_ac
S2_4334291	2	4334291	8.73E-03	GL7_14/E2
S2_60856616	2	60856616	8.74E-03	GL49_14/E2
S2_62128772	2	62128772	8.78E-03	GL35_ac
S2_9512992	2	9512992	8.83E-03	GL42_14/E2
S2_18307240	2	18307240	8.84E-03	GL42_13/E1
S2_18307256	2	18307256	8.84E-03	GL42_13/E1
S2_18307270	2	18307270	8.84E-03	GL42_13/E1
S2_13459070	2	13459070	8.85E-03	GL14_ac
S2_14746117	2	14746117	8.87E-03	GL35_ac
S2_14406452	2	14406452	8.87E-03	GL28_ac
S2_71490218	2	71490218	8.89E-03	GL42_ac
S2_69593580	2	69593580	8.91E-03	GL42_14/E2
S2_61105192	2	61105192	8.91E-03	GL14_14/E2
S2_49867897	2	49867897	8.92E-03	GL14_ac
S2_16814404	2	16814404	8.93E-03	GL28_13/E1
S2_16814409	2	16814409	8.93E-03	GL28_13/E1
S2_61686625	2	61686625	8.96E-03	GL42_ac
S2_16814404	2	16814404	8.98E-03	GL35_13/E1
S2_16814409	2	16814409	8.98E-03	GL35_13/E1
S2_47590004	2	47590004	9.00E-03	GL42_13/E1
S2_40321184	2	40321184	9.01E-03	GL28_13/E1
S2_7828106	2	7828106	9.05E-03	GL42_14/E2
S2_6277727	2	6277727	9.06E-03	GL28_ac
S2_6277742	2	6277742	9.06E-03	GL28_ac
S2_61923353	2	61923353	9.06E-03	GL35_14/E2
S2_58428674	2	58428674	9.06E-03	GL21_ac
S2_63290511	2	63290511	9.08E-03	GL35_ac
S2_63290512	2	63290512	9.08E-03	GL35_ac
S2_63290513	2	63290513	9.08E-03	GL35_ac
S2_63290514	2	63290514	9.08E-03	GL35_ac
S2_63290516	2	63290516	9.08E-03	GL35_ac
S2_63290517	2	63290517	9.08E-03	GL35_ac
S2_63290520	2	63290520	9.08E-03	GL35_ac
S2_63290523	2	63290523	9.08E-03	GL35_ac
S2_63290526	2	63290526	9.08E-03	GL35_ac
S2_63290527	2	63290527	9.08E-03	GL35_ac
S2_63290528	2	63290528	9.08E-03	GL35_ac
S2_63290533	2	63290533	9.08E-03	GL35_ac
S2_63290537	2	63290537	9.08E-03	GL35_ac
S2_69715021	2	69715021	9.09E-03	GL35_14/E2
S2_71486250	2	71486250	9.09E-03	GL42_13/E1
S2_75220306	2	75220306	9.10E-03	GL42_13/E1
S2_59751793	2	59751793	9.11E-03	GL35_13/E1
S2_59751803	2	59751803	9.11E-03	GL35_13/E1
S2_75342750	2	75342750	9.11E-03	GL42_14/E2
S2_10821401	2	10821401	9.15E-03	GL14_14/E2

SNP	Chromosome	Position	P.value	Trait
S2_10821418	2	10821418	9.15E-03	GL14_14/E2
S2_55211584	2	55211584	9.16E-03	GL7_13/E1
S2_55211626	2	55211626	9.16E-03	GL7_13/E1
S2_61841943	2	61841943	9.16E-03	GL7_ac
S2_58873435	2	58873435	9.19E-03	GL21_14/E2
S2_62726505	2	62726505	9.20E-03	GL42_14/E2
S2_62484730	2	62484730	9.24E-03	GL21_14/E2
S2_66492060	2	66492060	9.27E-03	GL35_14/E2
S2_66492108	2	66492108	9.27E-03	GL35_14/E2
S2_66074828	2	66074828	9.27E-03	GL28_13/E1
S2_65095903	2	65095903	9.27E-03	GL21_14/E2
S2_199703	2	199703	9.33E-03	GL35_ac
S2_68505778	2	68505778	9.34E-03	GL21_14/E2
S2_18309037	2	18309037	9.39E-03	GL7_13/E1
S2_40321184	2	40321184	9.41E-03	GL28_ac
S2_73037539	2	73037539	9.42E-03	GL7_13/E1
S2_14775620	2	14775620	9.42E-03	GL35_ac
S2_126519	2	126519	9.43E-03	GL14_ac
S2_60709420	2	60709420	9.43E-03	GL35_14/E2
S2_63803728	2	63803728	9.44E-03	GL28_14/E2
S2_8887713	2	8887713	9.44E-03	GL42_14/E2
S2_37434253	2	37434253	9.45E-03	GL7_14/E2
S2_56985131	2	56985131	9.47E-03	GL21_14/E2
S2_61233402	2	61233402	9.47E-03	GL42_14/E2
S2_66916115	2	66916115	9.48E-03	GL7_13/E1
S2_66916116	2	66916116	9.48E-03	GL7_13/E1
S2_67656785	2	67656785	9.48E-03	GL35_13/E1
S2_67620075	2	67620075	9.49E-03	GL14_13/E1
S2_38294461	2	38294461	9.50E-03	GL42_ac
S2_60709429	2	60709429	9.50E-03	GL42_ac
S2_6410085	2	6410085	9.53E-03	GL28_13/E1
S2_6410129	2	6410129	9.53E-03	GL28_13/E1
S2_3807228	2	3807228	9.53E-03	GL28_14/E2
S2_12905653	2	12905653	9.55E-03	GL49_ac
S2_12905708	2	12905708	9.55E-03	GL49_ac
S2_5786269	2	5786269	9.56E-03	GL14_13/E1
S2_63803728	2	63803728	9.57E-03	GL35_14/E2
S2_58239206	2	58239206	9.58E-03	GL28_14/E2
S2_58239285	2	58239285	9.58E-03	GL28_14/E2
S2_66412792	2	66412792	9.59E-03	GL42_14/E2
S2_66412795	2	66412795	9.59E-03	GL42_14/E2
S2_66412806	2	66412806	9.59E-03	GL42_14/E2
S2_66412842	2	66412842	9.59E-03	GL42_14/E2
S2_66412846	2	66412846	9.59E-03	GL42_14/E2
S2_66412848	2	66412848	9.59E-03	GL42_14/E2
S2_49867897	2	49867897	9.60E-03	GL7_ac
S2_4820725	2	4820725	9.60E-03	GL35_13/E1
S2_61233514	2	61233514	9.61E-03	GL28_ac
S2_61233514	2	61233514	9.61E-03	GL28_ac
S2_61233514	2	61233514	9.61E-03	GL28_ac
S2_61233514	2	61233514	9.61E-03	GL28_ac

SNP	Chromosome	Position	P.value	Trait
S2_60709420	2	60709420	9.63E-03	GL42_ac
S2_54461667	2	54461667	9.63E-03	GL21_ac
S2_62484730	2	62484730	9.66E-03	GL28_14/E2
S2_6888062	2	6888062	9.66E-03	GL28_14/E2
S2_6888065	2	6888065	9.66E-03	GL28_14/E2
S2_6888067	2	6888067	9.66E-03	GL28_14/E2
S2_6888069	2	6888069	9.66E-03	GL28_14/E2
S2_67330792	2	67330792	9.69E-03	GL49_ac
S2_29767393	2	29767393	9.71E-03	GL21_13/E1
S2_29767401	2	29767401	9.71E-03	GL21_13/E1
S2_65877733	2	65877733	9.71E-03	GL21_13/E1
S2_61233514	2	61233514	9.72E-03	GL21_ac
S2_61233514	2	61233514	9.72E-03	GL21_ac
S2_61233514	2	61233514	9.72E-03	GL21_ac
S2_61233514	2	61233514	9.72E-03	GL21_ac
S2_60856616	2	60856616	9.73E-03	GL21_ac
S2_7531999	2	7531999	9.75E-03	GL21_ac
S2_40651428	2	40651428	9.76E-03	GL7_14/E2
S2_47590004	2	47590004	9.78E-03	GL35_13/E1
S2_68556738	2	68556738	9.83E-03	GL42_14/E2
S2_73813174	2	73813174	9.84E-03	GL28_ac
S2_13459050	2	13459050	9.84E-03	GL7_ac
S2_76908521	2	76908521	9.84E-03	GL14_ac
S2_76908526	2	76908526	9.84E-03	GL14_ac
S2_76908527	2	76908527	9.84E-03	GL14_ac
S2_75792059	2	75792059	9.88E-03	GL49_14/E2
S2_75792053	2	75792053	9.88E-03	GL49_14/E2
S2_13459059	2	13459059	9.89E-03	GL35_ac
S2_64368069	2	64368069	9.94E-03	GL49_14/E2
S2_69572060	2	69572060	9.95E-03	GL35_14/E2
S2_14406452	2	14406452	9.96E-03	GL21_ac
S2_61548989	2	61548989	9.96E-03	GL42_ac
S2_61548991	2	61548991	9.96E-03	GL42_ac
S2_61549008	2	61549008	9.96E-03	GL42_ac
S2_61549009	2	61549009	9.96E-03	GL42_ac
S2_61549010	2	61549010	9.96E-03	GL42_ac
S2_61549011	2	61549011	9.96E-03	GL42_ac
S2_61549003	2	61549003	9.96E-03	GL42_ac
S2_61549004	2	61549004	9.96E-03	GL42_ac
S2_61549005	2	61549005	9.96E-03	GL42_ac
S2_61549007	2	61549007	9.96E-03	GL42_ac
S2_58286606	2	58286606	9.99E-03	GL7_13/E1
S2_66916116	2	66916116	9.99E-03	GL28_14/E2
S2_66916115	2	66916115	9.99E-03	GL28_14/E2
S3_73759500	3	73759500	1.43E-05	GL7_14/E2
S3_73181134	3	73181134	8.18E-05	GL7_14/E2
S3_73181134	3	73181134	1.50E-04	GL28_14/E2
S3_73609149	3	73609149	2.23E-04	GL7_14/E2
S3_59573293	3	59573293	2.48E-04	GL14_13/E1
S3_59573293	3	59573293	2.60E-04	GL28_13/E1
S3_59573293	3	59573293	2.75E-04	GL49_13/E1

SNP	Chromosome	Position	P.value	Trait
S3_68055529	3	68055529	2.89E-04	GL21_14/E2
S3_20069954	3	20069954	3.21E-04	GL35_14/E2
S3_68055528	3	68055528	3.26E-04	GL21_14/E2
S3_73907824	3	73907824	3.60E-04	GL7_14/E2
S3_59573293	3	59573293	4.06E-04	GL21_ac
S3_72849712	3	72849712	4.39E-04	GL28_14/E2
S3_72849706	3	72849706	4.39E-04	GL28_14/E2
S3_72849707	3	72849707	4.39E-04	GL28_14/E2
S3_61040495	3	61040495	4.67E-04	GL35_14/E2
S3_61040495	3	61040495	5.93E-04	GL49_ac
S3_59573293	3	59573293	6.02E-04	GL35_13/E1
S3_70976276	3	70976276	6.24E-04	GL7_13/E1
S3_70976292	3	70976292	6.24E-04	GL7_13/E1
S3_70976301	3	70976301	6.24E-04	GL7_13/E1
S3_70976306	3	70976306	6.24E-04	GL7_13/E1
S3_59573293	3	59573293	6.84E-04	GL21_13/E1
S3_70976276	3	70976276	7.06E-04	GL14_13/E1
S3_70976292	3	70976292	7.06E-04	GL14_13/E1
S3_70976301	3	70976301	7.06E-04	GL14_13/E1
S3_70976306	3	70976306	7.06E-04	GL14_13/E1
S3_53260490	3	53260490	7.37E-04	GL7_ac
S3_71874658	3	71874658	7.52E-04	GL7_14/E2
S3_22472367	3	22472367	8.16E-04	GL35_14/E2
S3_74310717	3	74310717	8.18E-04	GL35_14/E2
S3_74310760	3	74310760	8.18E-04	GL35_14/E2
S3_59573293	3	59573293	8.43E-04	GL14_ac
S3_3622825	3	3622825	8.81E-04	GL7_13/E1
S3_3622828	3	3622828	8.81E-04	GL7_13/E1
S3_3622832	3	3622832	8.81E-04	GL7_13/E1
S3_3622836	3	3622836	8.81E-04	GL7_13/E1
S3_3622850	3	3622850	8.81E-04	GL7_13/E1
S3_61040495	3	61040495	9.11E-04	GL35_ac
S3_57954741	3	57954741	9.59E-04	GL42_ac
S3_73027184	3	73027184	1.04E-03	GL7_14/E2
S3_72504817	3	72504817	1.06E-03	GL21_14/E2
S3_15620595	3	15620595	1.06E-03	GL14_14/E2
S3_72849689	3	72849689	1.06E-03	GL7_14/E2
S3_72087844	3	72087844	1.09E-03	GL7_14/E2
S3_59573293	3	59573293	1.12E-03	GL7_13/E1
S3_61040495	3	61040495	1.24E-03	GL42_14/E2
S3_61040495	3	61040495	1.30E-03	GL42_ac
S3_61393178	3	61393178	1.33E-03	GL28_ac
S3_73181134	3	73181134	1.35E-03	GL35_14/E2
S3_69123818	3	69123818	1.40E-03	GL42_13/E1
S3_72311013	3	72311013	1.49E-03	GL7_14/E2
S3_70976292	3	70976292	1.62E-03	GL7_ac
S3_70976301	3	70976301	1.62E-03	GL7_ac
S3_70976276	3	70976276	1.62E-03	GL7_ac
S3_70976306	3	70976306	1.62E-03	GL7_ac
S3_55908669	3	55908669	1.67E-03	GL35_14/E2
S3_69207670	3	69207670	1.69E-03	GL21_14/E2

SNP	Chromosome	Position	P.value	Trait
S3_73231302	3	73231302	1.70E-03	GL7_14/E2
S3_16332253	3	16332253	1.71E-03	GL35_ac
S3_73609149	3	73609149	1.74E-03	GL49_ac
S3_72071455	3	72071455	1.80E-03	GL7_14/E2
S3_72071460	3	72071460	1.85E-03	GL7_14/E2
S3_57954741	3	57954741	1.90E-03	GL42_14/E2
S3_53260490	3	53260490	1.93E-03	GL7_13/E1
S3_6605420	3	6605420	1.96E-03	GL21_14/E2
S3_73181134	3	73181134	1.99E-03	GL14_14/E2
S3_52182585	3	52182585	2.16E-03	GL35_14/E2
S3_61393178	3	61393178	2.16E-03	GL28_13/E1
S3_53260490	3	53260490	2.19E-03	GL14_13/E1
S3_59573293	3	59573293	2.24E-03	GL35_ac
S3_48669583	3	48669583	2.26E-03	GL7_14/E2
S3_3622825	3	3622825	2.29E-03	GL28_13/E1
S3_3622828	3	3622828	2.29E-03	GL28_13/E1
S3_3622832	3	3622832	2.29E-03	GL28_13/E1
S3_3622836	3	3622836	2.29E-03	GL28_13/E1
S3_3622850	3	3622850	2.29E-03	GL28_13/E1
S3_73149171	3	73149171	2.36E-03	GL7_14/E2
S3_1945637	3	1945637	2.41E-03	GL7_14/E2
S3_1945649	3	1945649	2.41E-03	GL7_14/E2
S3_1945682	3	1945682	2.41E-03	GL7_14/E2
S3_73181134	3	73181134	2.44E-03	GL21_14/E2
S3_20069954	3	20069954	2.45E-03	GL49_14/E2
S3_61393178	3	61393178	2.47E-03	GL35_13/E1
S3_69688959	3	69688959	2.47E-03	GL21_14/E2
S3_59573293	3	59573293	2.48E-03	GL28_ac
S3_63388746	3	63388746	2.52E-03	GL35_ac
S3_5146437	3	5146437	2.54E-03	GL7_14/E2
S3_5146440	3	5146440	2.54E-03	GL7_14/E2
S3_5146441	3	5146441	2.54E-03	GL7_14/E2
S3_5146459	3	5146459	2.54E-03	GL7_14/E2
S3_16332253	3	16332253	2.54E-03	GL28_ac
S3_72087844	3	72087844	2.56E-03	GL49_13/E1
S3_61393178	3	61393178	2.58E-03	GL21_13/E1
S3_70976276	3	70976276	2.63E-03	GL28_13/E1
S3_70976292	3	70976292	2.63E-03	GL28_13/E1
S3_70976301	3	70976301	2.63E-03	GL28_13/E1
S3_70976306	3	70976306	2.63E-03	GL28_13/E1
S3_70200633	3	70200633	2.70E-03	GL14_13/E1
S3_69967110	3	69967110	2.79E-03	GL14_ac
S3_5626292	3	5626292	2.81E-03	GL28_13/E1
S3_5626293	3	5626293	2.81E-03	GL28_13/E1
S3_19317784	3	19317784	2.86E-03	GL14_14/E2
S3_57954741	3	57954741	2.89E-03	GL49_ac
S3_16332253	3	16332253	2.91E-03	GL28_13/E1
S3_56070788	3	56070788	2.93E-03	GL35_14/E2
S3_73145496	3	73145496	2.96E-03	GL7_14/E2
S3_52182545	3	52182545	2.96E-03	GL35_14/E2
S3_52182575	3	52182575	2.96E-03	GL35_14/E2

SNP	Chromosome	Position	P.value	Trait
S3_52182580	3	52182580	2.96E-03	GL35_14/E2
S3_73217103	3	73217103	3.00E-03	GL7_14/E2
S3_72849706	3	72849706	3.01E-03	GL7_14/E2
S3_72849707	3	72849707	3.01E-03	GL7_14/E2
S3_72849712	3	72849712	3.01E-03	GL7_14/E2
S3_72321370	3	72321370	3.01E-03	GL14_14/E2
S3_15620595	3	15620595	3.03E-03	GL35_14/E2
S3_59573293	3	59573293	3.05E-03	GL49_ac
S3_73149171	3	73149171	3.12E-03	GL28_14/E2
S3_5146437	3	5146437	3.12E-03	GL7_ac
S3_5146440	3	5146440	3.12E-03	GL7_ac
S3_5146441	3	5146441	3.12E-03	GL7_ac
S3_5146459	3	5146459	3.12E-03	GL7_ac
S3_51536454	3	51536454	3.15E-03	GL14_ac
S3_3997275	3	3997275	3.16E-03	GL7_14/E2
S3_56160181	3	56160181	3.17E-03	GL35_14/E2
S3_56160181	3	56160181	3.17E-03	GL35_14/E2
S3_56160257	3	56160257	3.17E-03	GL35_14/E2
S3_56160181	3	56160181	3.17E-03	GL35_14/E2
S3_56160265	3	56160265	3.17E-03	GL35_14/E2
S3_16332253	3	16332253	3.18E-03	GL28_14/E2
S3_57759730	3	57759730	3.18E-03	GL35_13/E1
S3_72149527	3	72149527	3.25E-03	GL7_14/E2
S3_57759730	3	57759730	3.27E-03	GL49_13/E1
S3_68055530	3	68055530	3.28E-03	GL21_14/E2
S3_3997275	3	3997275	3.28E-03	GL21_14/E2
S3_73702647	3	73702647	3.28E-03	GL14_ac
S3_73702640	3	73702640	3.28E-03	GL14_ac
S3_3622825	3	3622825	3.28E-03	GL21_13/E1
S3_3622828	3	3622828	3.28E-03	GL21_13/E1
S3_3622832	3	3622832	3.28E-03	GL21_13/E1
S3_3622836	3	3622836	3.28E-03	GL21_13/E1
S3_3622850	3	3622850	3.28E-03	GL21_13/E1
S3_70253127	3	70253127	3.34E-03	GL49_ac
S3_63388746	3	63388746	3.39E-03	GL35_14/E2
S3_47886098	3	47886098	3.43E-03	GL28_13/E1
S3_5417977	3	5417977	3.50E-03	GL14_13/E1
S3_59492624	3	59492624	3.50E-03	GL49_13/E1
S3_68055529	3	68055529	3.50E-03	GL14_14/E2
S3_73907824	3	73907824	3.55E-03	GL28_14/E2
S3_53260490	3	53260490	3.57E-03	GL21_13/E1
S3_15620595	3	15620595	3.59E-03	GL21_14/E2
S3_54714984	3	54714984	3.66E-03	GL14_14/E2
S3_54714971	3	54714971	3.66E-03	GL14_14/E2
S3_73485829	3	73485829	3.70E-03	GL7_14/E2
S3_70976292	3	70976292	3.71E-03	GL14_ac
S3_70976301	3	70976301	3.71E-03	GL14_ac
S3_70976276	3	70976276	3.71E-03	GL14_ac
S3_70976306	3	70976306	3.71E-03	GL14_ac
S3_72440333	3	72440333	3.76E-03	GL7_14/E2
S3_56160263	3	56160263	3.77E-03	GL7_ac

SNP	Chromosome	Position	P.value	Trait
S3_56160266	3	56160266	3.77E-03	GL7_ac
S3_51536454	3	51536454	3.79E-03	GL7_ac
S3_56160181	3	56160181	3.79E-03	GL35_14/E2
S3_73609149	3	73609149	3.79E-03	GL28_14/E2
S3_56656490	3	56656490	3.79E-03	GL35_14/E2
S3_5137603	3	5137603	3.80E-03	GL7_14/E2
S3_70200633	3	70200633	3.84E-03	GL21_13/E1
S3_22472367	3	22472367	3.88E-03	GL28_14/E2
S3_59880946	3	59880946	3.89E-03	GL35_14/E2
S3_61393178	3	61393178	3.90E-03	GL35_ac
S3_73509002	3	73509002	3.91E-03	GL7_14/E2
S3_70486044	3	70486044	3.92E-03	GL42_14/E2
S3_61393178	3	61393178	3.92E-03	GL7_13/E1
S3_54714984	3	54714984	3.92E-03	GL21_14/E2
S3_54714971	3	54714971	3.92E-03	GL21_14/E2
S3_5041711	3	5041711	4.00E-03	GL28_ac
S3_70200633	3	70200633	4.05E-03	GL7_13/E1
S3_56160263	3	56160263	4.09E-03	GL35_14/E2
S3_56160266	3	56160266	4.09E-03	GL35_14/E2
S3_6605420	3	6605420	4.09E-03	GL28_14/E2
S3_74152972	3	74152972	4.17E-03	GL7_14/E2
S3_70392133	3	70392133	4.20E-03	GL28_13/E1
S3_16332253	3	16332253	4.21E-03	GL35_14/E2
S3_55801557	3	55801557	4.22E-03	GL42_ac
S3_55801558	3	55801558	4.22E-03	GL42_ac
S3_62087338	3	62087338	4.24E-03	GL14_14/E2
S3_61393178	3	61393178	4.24E-03	GL35_14/E2
S3_72001410	3	72001410	4.30E-03	GL21_14/E2
S3_71850604	3	71850604	4.33E-03	GL7_14/E2
S3_51536454	3	51536454	4.39E-03	GL7_13/E1
S3_62531572	3	62531572	4.42E-03	GL49_13/E1
S3_62531574	3	62531574	4.42E-03	GL49_13/E1
S3_62531577	3	62531577	4.42E-03	GL49_13/E1
S3_57369757	3	57369757	4.43E-03	GL49_ac
S3_62196944	3	62196944	4.46E-03	GL42_ac
S3_53520780	3	53520780	4.52E-03	GL35_14/E2
S3_51536454	3	51536454	4.52E-03	GL14_13/E1
S3_45987130	3	45987130	4.59E-03	GL49_14/E2
S3_15946969	3	15946969	4.60E-03	GL21_14/E2
S3_73087592	3	73087592	4.64E-03	GL7_14/E2
S3_73337352	3	73337352	4.64E-03	GL7_14/E2
S3_73634524	3	73634524	4.64E-03	GL7_14/E2
S3_6605420	3	6605420	4.64E-03	GL21_ac
S3_21774427	3	21774427	4.68E-03	GL35_14/E2
S3_73217029	3	73217029	4.70E-03	GL7_14/E2
S3_61393178	3	61393178	4.76E-03	GL21_ac
S3_52557257	3	52557257	4.76E-03	GL28_14/E2
S3_56070783	3	56070783	4.77E-03	GL35_14/E2
S3_72849689	3	72849689	4.82E-03	GL28_14/E2
S3_53260490	3	53260490	4.83E-03	GL28_13/E1
S3_71689566	3	71689566	4.86E-03	GL42_ac

SNP	Chromosome	Position	P.value	Trait
S3_57759730	3	57759730	4.90E-03	GL14_13/E1
S3_73702640	3	73702640	4.91E-03	GL14_13/E1
S3_73702647	3	73702647	4.91E-03	GL14_13/E1
S3_4610275	3	4610275	4.95E-03	GL7_14/E2
S3_59746637	3	59746637	4.97E-03	GL7_13/E1
S3_59746638	3	59746638	4.97E-03	GL7_13/E1
S3_59746639	3	59746639	4.97E-03	GL7_13/E1
S3_59746640	3	59746640	4.97E-03	GL7_13/E1
S3_59746642	3	59746642	4.97E-03	GL7_13/E1
S3_5640669	3	5640669	4.98E-03	GL7_14/E2
S3_69552210	3	69552210	4.99E-03	GL49_13/E1
S3_73087561	3	73087561	5.02E-03	GL7_14/E2
S3_6605420	3	6605420	5.07E-03	GL28_ac
S3_68055528	3	68055528	5.10E-03	GL14_14/E2
S3_15946969	3	15946969	5.14E-03	GL35_14/E2
S3_72807274	3	72807274	5.16E-03	GL14_13/E1
S3_63388746	3	63388746	5.16E-03	GL28_14/E2
S3_3997275	3	3997275	5.17E-03	GL14_14/E2
S3_4690521	3	4690521	5.19E-03	GL21_ac
S3_61040495	3	61040495	5.35E-03	GL14_ac
S3_5137603	3	5137603	5.37E-03	GL7_ac
S3_74099131	3	74099131	5.39E-03	GL7_14/E2
S3_16332253	3	16332253	5.42E-03	GL21_ac
S3_72800609	3	72800609	5.48E-03	GL21_14/E2
S3_52007842	3	52007842	5.53E-03	GL35_14/E2
S3_72504817	3	72504817	5.55E-03	GL28_14/E2
S3_72149527	3	72149527	5.61E-03	GL14_14/E2
S3_4690521	3	4690521	5.69E-03	GL49_ac
S3_72913128	3	72913128	5.70E-03	GL7_14/E2
S3_69637559	3	69637559	5.72E-03	GL14_ac
S3_70253127	3	70253127	5.73E-03	GL49_14/E2
S3_72917312	3	72917312	5.75E-03	GL7_14/E2
S3_16332253	3	16332253	5.76E-03	GL21_13/E1
S3_69637559	3	69637559	5.79E-03	GL21_ac
S3_72268827	3	72268827	5.80E-03	GL28_14/E2
S3_51536454	3	51536454	5.82E-03	GL35_ac
S3_5417977	3	5417977	5.83E-03	GL7_13/E1
S3_70547272	3	70547272	5.85E-03	GL7_14/E2
S3_70547280	3	70547280	5.85E-03	GL7_14/E2
S3_72140613	3	72140613	5.92E-03	GL7_14/E2
S3_72140617	3	72140617	5.92E-03	GL7_14/E2
S3_63388746	3	63388746	5.96E-03	GL14_14/E2
S3_74068840	3	74068840	5.96E-03	GL35_14/E2
S3_72125617	3	72125617	5.98E-03	GL42_13/E1
S3_48669557	3	48669557	5.99E-03	GL7_14/E2
S3_74178521	3	74178521	6.01E-03	GL7_14/E2
S3_19589570	3	19589570	6.05E-03	GL28_13/E1
S3_22472367	3	22472367	6.06E-03	GL7_14/E2
S3_3776153	3	3776153	6.07E-03	GL7_14/E2
S3_54878830	3	54878830	6.09E-03	GL28_14/E2
S3_72403315	3	72403315	6.11E-03	GL7_14/E2

SNP	Chromosome	Position	P.value	Trait
S3_7416112	3	7416112	6.18E-03	GL49_13/E1
S3_56150261	3	56150261	6.25E-03	GL7_ac
S3_56150267	3	56150267	6.25E-03	GL7_ac
S3_56150258	3	56150258	6.25E-03	GL7_ac
S3_56150264	3	56150264	6.25E-03	GL7_ac
S3_73861181	3	73861181	6.25E-03	GL7_14/E2
S3_63388746	3	63388746	6.26E-03	GL28_ac
S3_73040860	3	73040860	6.28E-03	GL21_13/E1
S3_72800609	3	72800609	6.32E-03	GL7_14/E2
S3_58878048	3	58878048	6.33E-03	GL7_14/E2
S3_4690521	3	4690521	6.33E-03	GL14_ac
S3_60767342	3	60767342	6.35E-03	GL7_13/E1
S3_60767367	3	60767367	6.35E-03	GL7_13/E1
S3_59503600	3	59503600	6.35E-03	GL49_13/E1
S3_59503603	3	59503603	6.35E-03	GL49_13/E1
S3_59503604	3	59503604	6.35E-03	GL49_13/E1
S3_59503608	3	59503608	6.35E-03	GL49_13/E1
S3_47886098	3	47886098	6.37E-03	GL35_13/E1
S3_73861174	3	73861174	6.39E-03	GL7_14/E2
S3_73861180	3	73861180	6.39E-03	GL7_14/E2
S3_73861183	3	73861183	6.39E-03	GL7_14/E2
S3_61040495	3	61040495	6.41E-03	GL49_14/E2
S3_72849712	3	72849712	6.43E-03	GL28_ac
S3_72849706	3	72849706	6.43E-03	GL28_ac
S3_72849707	3	72849707	6.43E-03	GL28_ac
S3_3622832	3	3622832	6.46E-03	GL28_ac
S3_3622836	3	3622836	6.46E-03	GL28_ac
S3_3622850	3	3622850	6.46E-03	GL28_ac
S3_3622825	3	3622825	6.46E-03	GL28_ac
S3_3622828	3	3622828	6.46E-03	GL28_ac
S3_59573293	3	59573293	6.48E-03	GL42_ac
S3_57759730	3	57759730	6.49E-03	GL14_ac
S3_72440333	3	72440333	6.54E-03	GL28_14/E2
S3_74310717	3	74310717	6.54E-03	GL14_14/E2
S3_74310760	3	74310760	6.54E-03	GL14_14/E2
S3_4690521	3	4690521	6.56E-03	GL14_14/E2
S3_72154612	3	72154612	6.57E-03	GL7_14/E2
S3_56656490	3	56656490	6.59E-03	GL42_ac
S3_73055176	3	73055176	6.70E-03	GL7_14/E2
S3_57759730	3	57759730	6.73E-03	GL28_13/E1
S3_72504817	3	72504817	6.79E-03	GL7_14/E2
S3_55805059	3	55805059	6.83E-03	GL14_14/E2
S3_72512315	3	72512315	6.83E-03	GL7_14/E2
S3_73702640	3	73702640	6.83E-03	GL7_13/E1
S3_73702647	3	73702647	6.83E-03	GL7_13/E1
S3_16332253	3	16332253	6.84E-03	GL7_ac
S3_50563646	3	50563646	6.86E-03	GL49_14/E2
S3_4459081	3	4459081	6.86E-03	GL42_14/E2
S3_57759730	3	57759730	6.88E-03	GL35_ac
S3_72125617	3	72125617	6.91E-03	GL21_13/E1
S3_69688959	3	69688959	6.91E-03	GL14_14/E2

SNP	Chromosome	Position	P.value	Trait
S3_72125617	3	72125617	6.91E-03	GL35_13/E1
S3_61393178	3	61393178	6.92E-03	GL14_ac
S3_56160263	3	56160263	6.92E-03	GL49_14/E2
S3_56160266	3	56160266	6.92E-03	GL49_14/E2
S3_69637559	3	69637559	7.04E-03	GL42_ac
S3_35819697	3	35819697	7.04E-03	GL14_13/E1
S3_73362984	3	73362984	7.04E-03	GL28_14/E2
S3_56160181	3	56160181	7.11E-03	GL7_ac
S3_56160181	3	56160181	7.11E-03	GL7_ac
S3_56160257	3	56160257	7.11E-03	GL7_ac
S3_56160181	3	56160181	7.11E-03	GL7_ac
S3_56160265	3	56160265	7.11E-03	GL7_ac
S3_5626292	3	5626292	7.13E-03	GL21_13/E1
S3_5626293	3	5626293	7.13E-03	GL21_13/E1
S3_72807274	3	72807274	7.14E-03	GL7_13/E1
S3_4760405	3	4760405	7.16E-03	GL7_14/E2
S3_71876675	3	71876675	7.16E-03	GL7_13/E1
S3_55801557	3	55801557	7.19E-03	GL42_14/E2
S3_55801558	3	55801558	7.19E-03	GL42_14/E2
S3_73329458	3	73329458	7.19E-03	GL7_14/E2
S3_59503609	3	59503609	7.22E-03	GL49_13/E1
S3_73609149	3	73609149	7.25E-03	GL49_14/E2
S3_22472367	3	22472367	7.27E-03	GL49_14/E2
S3_64840196	3	64840196	7.28E-03	GL49_14/E2
S3_72149527	3	72149527	7.33E-03	GL35_14/E2
S3_56160181	3	56160181	7.45E-03	GL49_14/E2
S3_56160181	3	56160181	7.45E-03	GL49_14/E2
S3_56160257	3	56160257	7.45E-03	GL49_14/E2
S3_56160181	3	56160181	7.45E-03	GL49_14/E2
S3_56160265	3	56160265	7.45E-03	GL49_14/E2
S3_74068840	3	74068840	7.46E-03	GL42_14/E2
S3_74099129	3	74099129	7.47E-03	GL7_14/E2
S3_70200633	3	70200633	7.50E-03	GL28_13/E1
S3_57321183	3	57321183	7.53E-03	GL28_13/E1
S3_2191124	3	2191124	7.59E-03	GL7_14/E2
S3_73425627	3	73425627	7.60E-03	GL7_14/E2
S3_68600838	3	68600838	7.63E-03	GL35_14/E2
S3_71054443	3	71054443	7.65E-03	GL49_14/E2
S3_73215479	3	73215479	7.67E-03	GL21_13/E1
S3_72125617	3	72125617	7.68E-03	GL28_13/E1
S3_59308318	3	59308318	7.71E-03	GL49_13/E1
S3_72123621	3	72123621	7.71E-03	GL7_ac
S3_55801294	3	55801294	7.72E-03	GL35_14/E2
S3_55801307	3	55801307	7.72E-03	GL35_14/E2
S3_70392133	3	70392133	7.72E-03	GL35_13/E1
S3_74310717	3	74310717	7.77E-03	GL35_ac
S3_74310760	3	74310760	7.77E-03	GL35_ac
S3_61393178	3	61393178	7.82E-03	GL14_13/E1
S3_13494206	3	13494206	7.82E-03	GL42_14/E2
S3_52182585	3	52182585	7.84E-03	GL7_ac
S3_66676550	3	66676550	7.85E-03	GL21_14/E2

SNP	Chromosome	Position	P.value	Trait
S3_66631231	3	66631231	7.89E-03	GL14_14/E2
S3_66631238	3	66631238	7.89E-03	GL14_14/E2
S3_72125617	3	72125617	7.90E-03	GL49_13/E1
S3_3622825	3	3622825	7.90E-03	GL35_13/E1
S3_3622828	3	3622828	7.90E-03	GL35_13/E1
S3_3622832	3	3622832	7.90E-03	GL35_13/E1
S3_3622836	3	3622836	7.90E-03	GL35_13/E1
S3_3622850	3	3622850	7.90E-03	GL35_13/E1
S3_59501629	3	59501629	7.92E-03	GL49_13/E1
S3_56160181	3	56160181	7.92E-03	GL7_ac
S3_56276031	3	56276031	7.95E-03	GL21_14/E2
S3_52182545	3	52182545	7.98E-03	GL7_ac
S3_52182575	3	52182575	7.98E-03	GL7_ac
S3_52182580	3	52182580	7.98E-03	GL7_ac
S3_72149527	3	72149527	8.03E-03	GL28_14/E2
S3_70976276	3	70976276	8.04E-03	GL49_13/E1
S3_70976292	3	70976292	8.04E-03	GL49_13/E1
S3_70976301	3	70976301	8.04E-03	GL49_13/E1
S3_70976306	3	70976306	8.04E-03	GL49_13/E1
S3_3622832	3	3622832	8.05E-03	GL21_ac
S3_3622836	3	3622836	8.05E-03	GL21_ac
S3_3622850	3	3622850	8.05E-03	GL21_ac
S3_3622825	3	3622825	8.05E-03	GL21_ac
S3_3622828	3	3622828	8.05E-03	GL21_ac
S3_70392133	3	70392133	8.07E-03	GL21_13/E1
S3_70976276	3	70976276	8.20E-03	GL21_13/E1
S3_70976292	3	70976292	8.20E-03	GL21_13/E1
S3_70976301	3	70976301	8.20E-03	GL21_13/E1
S3_70976306	3	70976306	8.20E-03	GL21_13/E1
S3_5417977	3	5417977	8.21E-03	GL21_13/E1
S3_57369759	3	57369759	8.23E-03	GL49_ac
S3_61378959	3	61378959	8.24E-03	GL42_14/E2
S3_70547280	3	70547280	8.24E-03	GL28_14/E2
S3_70547272	3	70547272	8.24E-03	GL28_14/E2
S3_71411263	3	71411263	8.27E-03	GL7_14/E2
S3_70688041	3	70688041	8.34E-03	GL42_14/E2
S3_72087844	3	72087844	8.35E-03	GL14_13/E1
S3_2047847	3	2047847	8.37E-03	GL49_14/E2
S3_73149171	3	73149171	8.37E-03	GL21_14/E2
S3_14383038	3	14383038	8.38E-03	GL35_13/E1
S3_59693387	3	59693387	8.39E-03	GL49_13/E1
S3_59693399	3	59693399	8.39E-03	GL49_13/E1
S3_16332253	3	16332253	8.44E-03	GL14_ac
S3_72505764	3	72505764	8.45E-03	GL28_14/E2
S3_71876675	3	71876675	8.48E-03	GL21_13/E1
S3_16168612	3	16168612	8.51E-03	GL14_14/E2
S3_66660389	3	66660389	8.53E-03	GL42_14/E2
S3_70860889	3	70860889	8.53E-03	GL28_14/E2
S3_52417141	3	52417141	8.54E-03	GL35_14/E2
S3_60581793	3	60581793	8.61E-03	GL49_ac
S3_60581801	3	60581801	8.61E-03	GL49_ac

SNP	Chromosome	Position	P.value	Trait
S3_60581809	3	60581809	8.61E-03	GL49_ac
S3_59503608	3	59503608	8.62E-03	GL42_ac
S3_59503600	3	59503600	8.62E-03	GL42_ac
S3_59503603	3	59503603	8.62E-03	GL42_ac
S3_59503604	3	59503604	8.62E-03	GL42_ac
S3_53324822	3	53324822	8.62E-03	GL35_14/E2
S3_53324852	3	53324852	8.62E-03	GL35_14/E2
S3_71876675	3	71876675	8.64E-03	GL14_13/E1
S3_66676550	3	66676550	8.67E-03	GL14_14/E2
S3_6759948	3	6759948	8.69E-03	GL42_13/E1
S3_54967398	3	54967398	8.70E-03	GL7_ac
S3_63388746	3	63388746	8.79E-03	GL49_ac
S3_57759730	3	57759730	8.86E-03	GL21_13/E1
S3_72321370	3	72321370	8.89E-03	GL7_14/E2
S3_74099136	3	74099136	8.89E-03	GL7_14/E2
S3_74099137	3	74099137	8.89E-03	GL7_14/E2
S3_57481362	3	57481362	8.90E-03	GL49_13/E1
S3_73014267	3	73014267	8.91E-03	GL21_14/E2
S3_4690521	3	4690521	8.95E-03	GL28_ac
S3_63388746	3	63388746	8.95E-03	GL49_14/E2
S3_72800609	3	72800609	8.97E-03	GL14_14/E2
S3_72071460	3	72071460	9.02E-03	GL21_14/E2
S3_53324796	3	53324796	9.07E-03	GL35_14/E2
S3_70976292	3	70976292	9.09E-03	GL42_ac
S3_70976301	3	70976301	9.09E-03	GL42_ac
S3_70976276	3	70976276	9.09E-03	GL42_ac
S3_70976306	3	70976306	9.09E-03	GL42_ac
S3_68205650	3	68205650	9.12E-03	GL42_ac
S3_73217103	3	73217103	9.12E-03	GL35_14/E2
S3_56160181	3	56160181	9.14E-03	GL49_14/E2
S3_64907616	3	64907616	9.16E-03	GL42_14/E2
S3_69967110	3	69967110	9.21E-03	GL14_13/E1
S3_71429118	3	71429118	9.22E-03	GL7_14/E2
S3_74099133	3	74099133	9.24E-03	GL7_14/E2
S3_69967110	3	69967110	9.25E-03	GL7_13/E1
S3_6754729	3	6754729	9.26E-03	GL14_ac
S3_6754738	3	6754738	9.26E-03	GL14_ac
S3_54823475	3	54823475	9.30E-03	GL7_ac
S3_51536454	3	51536454	9.30E-03	GL28_ac
S3_59573293	3	59573293	9.34E-03	GL7_ac
S3_59501629	3	59501629	9.39E-03	GL35_13/E1
S3_62196944	3	62196944	9.41E-03	GL28_14/E2
S3_5626292	3	5626292	9.41E-03	GL7_13/E1
S3_5626293	3	5626293	9.41E-03	GL7_13/E1
S3_61393178	3	61393178	9.43E-03	GL42_13/E1
S3_17	3	17	9.45E-03	GL35_14/E2
S3_14	3	14	9.45E-03	GL35_14/E2
S3_72989113	3	72989113	9.49E-03	GL7_14/E2
S3_70856879	3	70856879	9.52E-03	GL7_14/E2
S3_59573293	3	59573293	9.52E-03	GL42_13/E1
S3_72087844	3	72087844	9.54E-03	GL7_ac

SNP	Chromosome	Position	P.value	Trait
S3_73217103	3	73217103	9.55E-03	GL49_14/E2
S3_66699298	3	66699298	9.55E-03	GL42_13/E1
S3_15959211	3	15959211	9.60E-03	GL28_14/E2
S3_5626292	3	5626292	9.67E-03	GL28_ac
S3_5626293	3	5626293	9.67E-03	GL28_ac
S3_5626292	3	5626292	9.70E-03	GL14_13/E1
S3_5626293	3	5626293	9.70E-03	GL14_13/E1
S3_66606594	3	66606594	9.71E-03	GL7_14/E2
S3_61040495	3	61040495	9.77E-03	GL7_ac
S3_53972530	3	53972530	9.81E-03	GL7_ac
S3_59503609	3	59503609	9.85E-03	GL42_ac
S3_69552210	3	69552210	9.86E-03	GL42_13/E1
S3_73881401	3	73881401	9.93E-03	GL7_14/E2
S3_53551432	3	53551432	9.96E-03	GL35_14/E2
S3_56276013	3	56276013	9.98E-03	GL21_14/E2
S4_36263190	4	36263190	2.17E-04	GL21_ac
S4_63170220	4	63170220	2.43E-04	GL35_13/E1
S4_36263190	4	36263190	2.59E-04	GL28_ac
S4_36263190	4	36263190	2.67E-04	GL7_ac
S4_55547289	4	55547289	2.81E-04	GL14_14/E2
S4_43199868	4	43199868	2.86E-04	GL14_14/E2
S4_63170220	4	63170220	3.07E-04	GL42_ac
S4_7982383	4	7982383	3.31E-04	GL21_14/E2
S4_63170220	4	63170220	3.68E-04	GL28_ac
S4_41454633	4	41454633	3.73E-04	GL42_14/E2
S4_56611095	4	56611095	3.92E-04	GL49_ac
S4_36263190	4	36263190	4.05E-04	GL21_13/E1
S4_62880530	4	62880530	4.28E-04	GL7_ac
S4_39654579	4	39654579	4.67E-04	GL28_ac
S4_63170220	4	63170220	4.76E-04	GL35_ac
S4_53162965	4	53162965	5.15E-04	GL14_ac
S4_54055164	4	54055164	5.41E-04	GL35_13/E1
S4_54055170	4	54055170	5.41E-04	GL35_13/E1
S4_52118628	4	52118628	5.57E-04	GL7_ac
S4_52118633	4	52118633	5.57E-04	GL7_ac
S4_52118630	4	52118630	5.57E-04	GL7_ac
S4_52118634	4	52118634	5.57E-04	GL7_ac
S4_7468105	4	7468105	5.64E-04	GL28_14/E2
S4_7468111	4	7468111	5.64E-04	GL28_14/E2
S4_7468114	4	7468114	5.64E-04	GL28_14/E2
S4_7468129	4	7468129	5.64E-04	GL28_14/E2
S4_36263190	4	36263190	5.94E-04	GL7_13/E1
S4_39654579	4	39654579	6.18E-04	GL35_13/E1
S4_53162965	4	53162965	6.23E-04	GL49_ac
S4_10427993	4	10427993	6.75E-04	GL49_14/E2
S4_19288511	4	19288511	7.55E-04	GL49_ac
S4_36263190	4	36263190	7.65E-04	GL14_ac
S4_8916481	4	8916481	7.94E-04	GL28_ac
S4_8916473	4	8916473	7.94E-04	GL28_ac
S4_63170220	4	63170220	8.29E-04	GL21_ac
S4_39654579	4	39654579	8.46E-04	GL28_13/E1

SNP	Chromosome	Position	P.value	Trait
S4_25292491	4	25292491	8.66E-04	GL49_ac
S4_53162965	4	53162965	9.56E-04	GL14_14/E2
S4_1337768	4	1337768	9.87E-04	GL35_13/E1
S4_1337768	4	1337768	9.90E-04	GL28_ac
S4_51957270	4	51957270	1.02E-03	GL35_ac
S4_55552100	4	55552100	1.02E-03	GL49_ac
S4_10427993	4	10427993	1.03E-03	GL49_ac
S4_41454633	4	41454633	1.08E-03	GL42_ac
S4_52118628	4	52118628	1.08E-03	GL28_13/E1
S4_52118630	4	52118630	1.08E-03	GL28_13/E1
S4_52118633	4	52118633	1.08E-03	GL28_13/E1
S4_52118634	4	52118634	1.08E-03	GL28_13/E1
S4_52118628	4	52118628	1.08E-03	GL28_ac
S4_52118633	4	52118633	1.08E-03	GL28_ac
S4_52118630	4	52118630	1.08E-03	GL28_ac
S4_52118634	4	52118634	1.08E-03	GL28_ac
S4_52118628	4	52118628	1.10E-03	GL21_13/E1
S4_52118630	4	52118630	1.10E-03	GL21_13/E1
S4_52118633	4	52118633	1.10E-03	GL21_13/E1
S4_52118634	4	52118634	1.10E-03	GL21_13/E1
S4_19288511	4	19288511	1.11E-03	GL7_ac
S4_63170220	4	63170220	1.23E-03	GL42_13/E1
S4_52118628	4	52118628	1.23E-03	GL21_ac
S4_52118633	4	52118633	1.23E-03	GL21_ac
S4_52118630	4	52118630	1.23E-03	GL21_ac
S4_52118634	4	52118634	1.23E-03	GL21_ac
S4_41454633	4	41454633	1.24E-03	GL49_ac
S4_1337768	4	1337768	1.28E-03	GL35_ac
S4_52118628	4	52118628	1.28E-03	GL35_13/E1
S4_52118630	4	52118630	1.28E-03	GL35_13/E1
S4_52118633	4	52118633	1.28E-03	GL35_13/E1
S4_52118634	4	52118634	1.28E-03	GL35_13/E1
S4_36263190	4	36263190	1.30E-03	GL28_13/E1
S4_56427895	4	56427895	1.32E-03	GL42_14/E2
S4_56427923	4	56427923	1.32E-03	GL42_14/E2
S4_56427893	4	56427893	1.32E-03	GL42_14/E2
S4_56427894	4	56427894	1.32E-03	GL42_14/E2
S4_56427898	4	56427898	1.32E-03	GL42_14/E2
S4_36263190	4	36263190	1.35E-03	GL14_13/E1
S4_54055164	4	54055164	1.38E-03	GL42_13/E1
S4_54055170	4	54055170	1.38E-03	GL42_13/E1
S4_66125410	4	66125410	1.39E-03	GL7_14/E2
S4_2234744	4	2234744	1.39E-03	GL49_ac
S4_41454633	4	41454633	1.41E-03	GL49_14/E2
S4_38905545	4	38905545	1.44E-03	GL49_14/E2
S4_56427895	4	56427895	1.45E-03	GL7_ac
S4_56427923	4	56427923	1.45E-03	GL7_ac
S4_56427893	4	56427893	1.45E-03	GL7_ac
S4_56427894	4	56427894	1.45E-03	GL7_ac
S4_56427898	4	56427898	1.45E-03	GL7_ac
S4_7982383	4	7982383	1.48E-03	GL14_14/E2

SNP	Chromosome	Position	P.value	Trait
S4_39654579	4	39654579	1.50E-03	GL21_ac
S4_1337768	4	1337768	1.51E-03	GL28_13/E1
S4_13143419	4	13143419	1.55E-03	GL7_14/E2
S4_13143449	4	13143449	1.55E-03	GL7_14/E2
S4_13143461	4	13143461	1.55E-03	GL7_14/E2
S4_13143466	4	13143466	1.55E-03	GL7_14/E2
S4_13143467	4	13143467	1.55E-03	GL7_14/E2
S4_13143468	4	13143468	1.55E-03	GL7_14/E2
S4_13143469	4	13143469	1.55E-03	GL7_14/E2
S4_8916481	4	8916481	1.56E-03	GL21_ac
S4_8916473	4	8916473	1.56E-03	GL21_ac
S4_38905545	4	38905545	1.56E-03	GL49_ac
S4_56570801	4	56570801	1.58E-03	GL14_14/E2
S4_55552100	4	55552100	1.60E-03	GL49_14/E2
S4_56654900	4	56654900	1.61E-03	GL21_14/E2
S4_67119244	4	67119244	1.66E-03	GL14_14/E2
S4_56570801	4	56570801	1.74E-03	GL21_14/E2
S4_56611095	4	56611095	1.75E-03	GL49_14/E2
S4_5107303	4	5107303	1.75E-03	GL28_13/E1
S4_54055164	4	54055164	1.76E-03	GL28_13/E1
S4_54055170	4	54055170	1.76E-03	GL28_13/E1
S4_1337768	4	1337768	1.79E-03	GL21_ac
S4_50646022	4	50646022	1.84E-03	GL35_14/E2
S4_1216332	4	1216332	1.88E-03	GL14_ac
S4_15095692	4	15095692	1.90E-03	GL14_ac
S4_56654691	4	56654691	1.90E-03	GL28_14/E2
S4_52118628	4	52118628	1.92E-03	GL7_13/E1
S4_52118630	4	52118630	1.92E-03	GL7_13/E1
S4_52118633	4	52118633	1.92E-03	GL7_13/E1
S4_52118634	4	52118634	1.92E-03	GL7_13/E1
S4_9328122	4	9328122	1.93E-03	GL21_14/E2
S4_8592446	4	8592446	1.95E-03	GL42_ac
S4_39654579	4	39654579	1.96E-03	GL35_ac
S4_56611095	4	56611095	2.00E-03	GL7_ac
S4_67288874	4	67288874	2.02E-03	GL7_14/E2
S4_50646022	4	50646022	2.02E-03	GL14_14/E2
S4_68098051	4	68098051	2.05E-03	GL28_14/E2
S4_68098036	4	68098036	2.05E-03	GL28_14/E2
S4_68098045	4	68098045	2.05E-03	GL28_14/E2
S4_68098053	4	68098053	2.05E-03	GL28_14/E2
S4_53162965	4	53162965	2.08E-03	GL28_ac
S4_52118628	4	52118628	2.09E-03	GL49_13/E1
S4_52118630	4	52118630	2.09E-03	GL49_13/E1
S4_52118633	4	52118633	2.09E-03	GL49_13/E1
S4_52118634	4	52118634	2.09E-03	GL49_13/E1
S4_56522702	4	56522702	2.11E-03	GL7_ac
S4_53162965	4	53162965	2.12E-03	GL7_ac
S4_51460491	4	51460491	2.14E-03	GL35_ac
S4_13036200	4	13036200	2.17E-03	GL28_14/E2
S4_38905545	4	38905545	2.20E-03	GL42_14/E2
S4_13036200	4	13036200	2.22E-03	GL7_14/E2

SNP	Chromosome	Position	P.value	Trait
S4_16066479	4	16066479	2.33E-03	GL35_ac
S4_56654900	4	56654900	2.33E-03	GL49_14/E2
S4_8916481	4	8916481	2.40E-03	GL28_13/E1
S4_8916473	4	8916473	2.40E-03	GL28_13/E1
S4_42822700	4	42822700	2.40E-03	GL49_13/E1
S4_5107303	4	5107303	2.40E-03	GL35_13/E1
S4_52118628	4	52118628	2.46E-03	GL42_13/E1
S4_52118630	4	52118630	2.46E-03	GL42_13/E1
S4_52118633	4	52118633	2.46E-03	GL42_13/E1
S4_52118634	4	52118634	2.46E-03	GL42_13/E1
S4_53811522	4	53811522	2.46E-03	GL14_14/E2
S4_19288511	4	19288511	2.49E-03	GL14_ac
S4_55547289	4	55547289	2.53E-03	GL14_ac
S4_53162965	4	53162965	2.56E-03	GL21_ac
S4_41454633	4	41454633	2.57E-03	GL35_14/E2
S4_55546064	4	55546064	2.58E-03	GL14_14/E2
S4_55546078	4	55546078	2.58E-03	GL14_14/E2
S4_9587212	4	9587212	2.59E-03	GL14_14/E2
S4_16066479	4	16066479	2.61E-03	GL28_ac
S4_50646022	4	50646022	2.62E-03	GL28_14/E2
S4_43199868	4	43199868	2.64E-03	GL28_14/E2
S4_16066479	4	16066479	2.69E-03	GL42_ac
S4_19288511	4	19288511	2.75E-03	GL42_ac
S4_67214412	4	67214412	2.76E-03	GL49_14/E2
S4_36263190	4	36263190	2.80E-03	GL35_ac
S4_51957270	4	51957270	2.83E-03	GL42_ac
S4_50348958	4	50348958	2.87E-03	GL28_ac
S4_63170220	4	63170220	2.91E-03	GL28_13/E1
S4_56654900	4	56654900	3.00E-03	GL28_14/E2
S4_56615273	4	56615273	3.10E-03	GL7_ac
S4_60584591	4	60584591	3.14E-03	GL21_14/E2
S4_6805481	4	6805481	3.15E-03	GL42_14/E2
S4_23663550	4	23663550	3.15E-03	GL49_ac
S4_23663550	4	23663550	3.18E-03	GL49_14/E2
S4_51957270	4	51957270	3.19E-03	GL28_ac
S4_46239103	4	46239103	3.20E-03	GL14_ac
S4_1659391	4	1659391	3.24E-03	GL7_ac
S4_51957270	4	51957270	3.27E-03	GL35_13/E1
S4_8916481	4	8916481	3.29E-03	GL7_ac
S4_8916473	4	8916473	3.29E-03	GL7_ac
S4_55552100	4	55552100	3.32E-03	GL42_14/E2
S4_66254474	4	66254474	3.33E-03	GL7_ac
S4_43825975	4	43825975	3.34E-03	GL42_14/E2
S4_43199868	4	43199868	3.41E-03	GL49_ac
S4_36263190	4	36263190	3.42E-03	GL49_13/E1
S4_2645020	4	2645020	3.44E-03	GL49_ac
S4_62936586	4	62936586	3.49E-03	GL7_ac
S4_7468105	4	7468105	3.50E-03	GL21_14/E2
S4_7468111	4	7468111	3.50E-03	GL21_14/E2
S4_7468114	4	7468114	3.50E-03	GL21_14/E2
S4_7468129	4	7468129	3.50E-03	GL21_14/E2

SNP	Chromosome	Position	P.value	Trait
S4_6805481	4	6805481	3.56E-03	GL35_14/E2
S4_52118628	4	52118628	3.57E-03	GL14_13/E1
S4_52118630	4	52118630	3.57E-03	GL14_13/E1
S4_52118633	4	52118633	3.57E-03	GL14_13/E1
S4_52118634	4	52118634	3.57E-03	GL14_13/E1
S4_67712021	4	67712021	3.57E-03	GL35_ac
S4_56557333	4	56557333	3.64E-03	GL7_ac
S4_16066479	4	16066479	3.67E-03	GL42_13/E1
S4_58459316	4	58459316	3.68E-03	GL35_14/E2
S4_19288511	4	19288511	3.73E-03	GL35_ac
S4_53866167	4	53866167	3.78E-03	GL14_14/E2
S4_27569225	4	27569225	3.82E-03	GL35_ac
S4_27569230	4	27569230	3.82E-03	GL35_ac
S4_50348958	4	50348958	3.84E-03	GL7_ac
S4_56427899	4	56427899	3.87E-03	GL7_ac
S4_1337768	4	1337768	3.89E-03	GL42_ac
S4_13036200	4	13036200	3.91E-03	GL49_14/E2
S4_56570801	4	56570801	3.92E-03	GL28_14/E2
S4_2645020	4	2645020	4.05E-03	GL7_ac
S4_43199868	4	43199868	4.07E-03	GL21_14/E2
S4_51771351	4	51771351	4.08E-03	GL49_13/E1
S4_63395529	4	63395529	4.09E-03	GL42_13/E1
S4_63395536	4	63395536	4.09E-03	GL42_13/E1
S4_63395561	4	63395561	4.09E-03	GL42_13/E1
S4_51460491	4	51460491	4.11E-03	GL49_ac
S4_67214412	4	67214412	4.12E-03	GL49_14/E2
S4_67214412	4	67214412	4.12E-03	GL49_14/E2
S4_67214412	4	67214412	4.12E-03	GL49_14/E2
S4_67214412	4	67214412	4.12E-03	GL49_14/E2
S4_67214412	4	67214412	4.12E-03	GL49_14/E2
S4_56654900	4	56654900	4.12E-03	GL14_14/E2
S4_7277550	4	7277550	4.13E-03	GL28_13/E1
S4_27569225	4	27569225	4.15E-03	GL28_13/E1
S4_27569230	4	27569230	4.15E-03	GL28_13/E1
S4_24546731	4	24546731	4.15E-03	GL49_ac
S4_52118628	4	52118628	4.17E-03	GL35_ac
S4_52118633	4	52118633	4.17E-03	GL35_ac
S4_52118630	4	52118630	4.17E-03	GL35_ac
S4_52118634	4	52118634	4.17E-03	GL35_ac
S4_7982383	4	7982383	4.28E-03	GL28_14/E2
S4_51839386	4	51839386	4.41E-03	GL49_14/E2
S4_41454633	4	41454633	4.43E-03	GL35_ac
S4_36263190	4	36263190	4.45E-03	GL49_ac
S4_7277550	4	7277550	4.46E-03	GL35_13/E1
S4_6901834	4	6901834	4.46E-03	GL14_ac
S4_51957270	4	51957270	4.48E-03	GL42_13/E1
S4_51277611	4	51277611	4.51E-03	GL35_14/E2
S4_51277634	4	51277634	4.51E-03	GL35_14/E2
S4_51277651	4	51277651	4.51E-03	GL35_14/E2
S4_58174391	4	58174391	4.60E-03	GL42_14/E2
S4_62936396	4	62936396	4.60E-03	GL7_ac

SNP	Chromosome	Position	P.value	Trait
S4_9443882	4	9443882	4.63E-03	GL49_14/E2
S4_5107303	4	5107303	4.64E-03	GL42_13/E1
S4_56570801	4	56570801	4.70E-03	GL28_ac
S4_25292491	4	25292491	4.75E-03	GL49_14/E2
S4_1216332	4	1216332	4.89E-03	GL7_ac
S4_1337768	4	1337768	4.91E-03	GL42_13/E1
S4_56427899	4	56427899	4.92E-03	GL14_14/E2
S4_20114058	4	20114058	4.92E-03	GL35_ac
S4_63170220	4	63170220	4.99E-03	GL21_13/E1
S4_62936063	4	62936063	5.01E-03	GL35_14/E2
S4_62936040	4	62936040	5.01E-03	GL35_14/E2
S4_62936061	4	62936061	5.01E-03	GL35_14/E2
S4_63170220	4	63170220	5.02E-03	GL49_13/E1
S4_56611095	4	56611095	5.07E-03	GL42_14/E2
S4_40071868	4	40071868	5.08E-03	GL35_13/E1
S4_43523383	4	43523383	5.09E-03	GL28_ac
S4_6415429	4	6415429	5.13E-03	GL42_13/E1
S4_51199590	4	51199590	5.17E-03	GL49_13/E1
S4_51199593	4	51199593	5.17E-03	GL49_13/E1
S4_52118628	4	52118628	5.22E-03	GL28_14/E2
S4_52118633	4	52118633	5.22E-03	GL28_14/E2
S4_52118630	4	52118630	5.22E-03	GL28_14/E2
S4_52118634	4	52118634	5.22E-03	GL28_14/E2
S4_6901834	4	6901834	5.24E-03	GL21_ac
S4_55553811	4	55553811	5.35E-03	GL14_14/E2
S4_55553820	4	55553820	5.35E-03	GL14_14/E2
S4_1337768	4	1337768	5.37E-03	GL21_13/E1
S4_5561087	4	5561087	5.39E-03	GL21_ac
S4_5107303	4	5107303	5.49E-03	GL21_13/E1
S4_20114058	4	20114058	5.54E-03	GL28_13/E1
S4_58459316	4	58459316	5.62E-03	GL42_14/E2
S4_50348958	4	50348958	5.63E-03	GL21_ac
S4_50348958	4	50348958	5.64E-03	GL49_13/E1
S4_56610968	4	56610968	5.65E-03	GL21_14/E2
S4_63395529	4	63395529	5.66E-03	GL35_13/E1
S4_63395536	4	63395536	5.66E-03	GL35_13/E1
S4_63395561	4	63395561	5.66E-03	GL35_13/E1
S4_53866167	4	53866167	5.72E-03	GL42_14/E2
S4_6901834	4	6901834	5.78E-03	GL49_ac
S4_49546333	4	49546333	5.81E-03	GL49_ac
S4_55547289	4	55547289	5.81E-03	GL7_ac
S4_56611095	4	56611095	5.83E-03	GL14_ac
S4_62936583	4	62936583	5.84E-03	GL7_ac
S4_62936585	4	62936585	5.84E-03	GL7_ac
S4_62936588	4	62936588	5.84E-03	GL7_ac
S4_62936589	4	62936589	5.84E-03	GL7_ac
S4_62936596	4	62936596	5.84E-03	GL7_ac
S4_62936581	4	62936581	5.84E-03	GL7_ac
S4_62936592	4	62936592	5.84E-03	GL7_ac
S4_62936599	4	62936599	5.84E-03	GL7_ac
S4_8916481	4	8916481	5.86E-03	GL21_13/E1

SNP	Chromosome	Position	P.value	Trait
S4_8916473	4	8916473	5.86E-03	GL21_13/E1
S4_56610968	4	56610968	5.89E-03	GL7_ac
S4_53752969	4	53752969	5.90E-03	GL14_14/E2
S4_67712021	4	67712021	5.90E-03	GL14_ac
S4_52118628	4	52118628	5.91E-03	GL14_ac
S4_52118633	4	52118633	5.91E-03	GL14_ac
S4_52118630	4	52118630	5.91E-03	GL14_ac
S4_52118634	4	52118634	5.91E-03	GL14_ac
S4_15095692	4	15095692	5.92E-03	GL7_13/E1
S4_36166378	4	36166378	5.99E-03	GL35_14/E2
S4_36166392	4	36166392	5.99E-03	GL35_14/E2
S4_9443882	4	9443882	6.01E-03	GL42_14/E2
S4_39654579	4	39654579	6.01E-03	GL21_13/E1
S4_56570801	4	56570801	6.03E-03	GL7_ac
S4_43825975	4	43825975	6.09E-03	GL49_ac
S4_51106787	4	51106787	6.18E-03	GL7_ac
S4_2234744	4	2234744	6.19E-03	GL49_14/E2
S4_55584592	4	55584592	6.20E-03	GL21_14/E2
S4_22476336	4	22476336	6.21E-03	GL7_ac
S4_14247808	4	14247808	6.24E-03	GL49_13/E1
S4_53842243	4	53842243	6.27E-03	GL21_14/E2
S4_10427993	4	10427993	6.29E-03	GL42_ac
S4_16066479	4	16066479	6.30E-03	GL28_13/E1
S4_54055164	4	54055164	6.36E-03	GL21_13/E1
S4_54055170	4	54055170	6.36E-03	GL21_13/E1
S4_1645507	4	1645507	6.37E-03	GL28_13/E1
S4_16066479	4	16066479	6.38E-03	GL21_ac
S4_40351832	4	40351832	6.39E-03	GL49_13/E1
S4_36263190	4	36263190	6.44E-03	GL35_13/E1
S4_50348958	4	50348958	6.44E-03	GL28_14/E2
S4_51460491	4	51460491	6.45E-03	GL28_ac
S4_62988514	4	62988514	6.47E-03	GL7_ac
S4_55547289	4	55547289	6.47E-03	GL35_ac
S4_50348958	4	50348958	6.51E-03	GL35_ac
S4_63410059	4	63410059	6.67E-03	GL7_ac
S4_63410079	4	63410079	6.67E-03	GL7_ac
S4_58259891	4	58259891	6.74E-03	GL42_ac
S4_5561087	4	5561087	6.75E-03	GL7_ac
S4_34662222	4	34662222	6.77E-03	GL28_13/E1
S4_43752393	4	43752393	6.82E-03	GL49_ac
S4_39654579	4	39654579	6.85E-03	GL14_ac
S4_50348958	4	50348958	6.86E-03	GL28_13/E1
S4_56654691	4	56654691	6.87E-03	GL14_14/E2
S4_55547370	4	55547370	6.87E-03	GL49_ac
S4_6463380	4	6463380	6.92E-03	GL7_13/E1
S4_51774534	4	51774534	6.94E-03	GL42_14/E2
S4_51774538	4	51774538	6.94E-03	GL42_14/E2
S4_51774544	4	51774544	6.94E-03	GL42_14/E2
S4_51774564	4	51774564	6.94E-03	GL42_14/E2
S4_5561087	4	5561087	7.00E-03	GL28_ac
S4_55546064	4	55546064	7.00E-03	GL28_14/E2

SNP	Chromosome	Position	P.value	Trait
S4_55546078	4	55546078	7.00E-03	GL28_14/E2
S4_54055164	4	54055164	7.03E-03	GL28_ac
S4_54055170	4	54055170	7.03E-03	GL28_ac
S4_56427895	4	56427895	7.05E-03	GL42_ac
S4_56427923	4	56427923	7.05E-03	GL42_ac
S4_56427893	4	56427893	7.05E-03	GL42_ac
S4_56427894	4	56427894	7.05E-03	GL42_ac
S4_56427898	4	56427898	7.05E-03	GL42_ac
S4_63170220	4	63170220	7.07E-03	GL14_ac
S4_1373052	4	1373052	7.08E-03	GL28_ac
S4_62988514	4	62988514	7.14E-03	GL7_13/E1
S4_13036200	4	13036200	7.16E-03	GL49_ac
S4_56654900	4	56654900	7.16E-03	GL42_14/E2
S4_56611095	4	56611095	7.18E-03	GL28_14/E2
S4_55552100	4	55552100	7.18E-03	GL42_ac
S4_19288511	4	19288511	7.21E-03	GL21_ac
S4_62936063	4	62936063	7.26E-03	GL42_14/E2
S4_62936040	4	62936040	7.26E-03	GL42_14/E2
S4_62936061	4	62936061	7.26E-03	GL42_14/E2
S4_55547289	4	55547289	7.31E-03	GL21_14/E2
S4_34662222	4	34662222	7.31E-03	GL7_ac
S4_62880530	4	62880530	7.35E-03	GL7_13/E1
S4_55584592	4	55584592	7.38E-03	GL14_14/E2
S4_16050848	4	16050848	7.41E-03	GL49_14/E2
S4_50646022	4	50646022	7.44E-03	GL21_14/E2
S4_42822700	4	42822700	7.49E-03	GL42_ac
S4_46972443	4	46972443	7.49E-03	GL49_ac
S4_46972445	4	46972445	7.49E-03	GL49_ac
S4_46972428	4	46972428	7.49E-03	GL49_ac
S4_51460491	4	51460491	7.57E-03	GL21_ac
S4_55553811	4	55553811	7.59E-03	GL49_ac
S4_55553820	4	55553820	7.59E-03	GL49_ac
S4_53162965	4	53162965	7.61E-03	GL21_14/E2
S4_6463380	4	6463380	7.74E-03	GL14_13/E1
S4_46239103	4	46239103	7.75E-03	GL7_ac
S4_51199590	4	51199590	7.75E-03	GL28_13/E1
S4_51199593	4	51199593	7.75E-03	GL28_13/E1
S4_56428568	4	56428568	7.78E-03	GL14_14/E2
S4_24546731	4	24546731	7.80E-03	GL28_14/E2
S4_54055164	4	54055164	7.80E-03	GL21_ac
S4_54055170	4	54055170	7.80E-03	GL21_ac
S4_25714460	4	25714460	7.83E-03	GL49_ac
S4_63170220	4	63170220	7.86E-03	GL7_13/E1
S4_53708540	4	53708540	7.86E-03	GL14_14/E2
S4_1216332	4	1216332	7.86E-03	GL7_13/E1
S4_10709836	4	10709836	7.89E-03	GL14_13/E1
S4_56611095	4	56611095	7.90E-03	GL42_ac
S4_6463380	4	6463380	8.00E-03	GL7_ac
S4_51957270	4	51957270	8.01E-03	GL21_ac
S4_50348958	4	50348958	8.02E-03	GL21_13/E1
S4_60584591	4	60584591	8.02E-03	GL35_14/E2

SNP	Chromosome	Position	P.value	Trait
S4_5561087	4	5561087	8.06E-03	GL21_13/E1
S4_16066479	4	16066479	8.07E-03	GL35_13/E1
S4_7277550	4	7277550	8.15E-03	GL21_13/E1
S4_34662222	4	34662222	8.18E-03	GL49_13/E1
S4_22432524	4	22432524	8.19E-03	GL49_ac
S4_51199590	4	51199590	8.28E-03	GL35_13/E1
S4_51199593	4	51199593	8.28E-03	GL35_13/E1
S4_67092863	4	67092863	8.34E-03	GL14_ac
S4_54055164	4	54055164	8.34E-03	GL7_13/E1
S4_54055170	4	54055170	8.34E-03	GL7_13/E1
S4_51106775	4	51106775	8.35E-03	GL7_ac
S4_52408267	4	52408267	8.35E-03	GL7_ac
S4_56610968	4	56610968	8.36E-03	GL28_14/E2
S4_3715066	4	3715066	8.47E-03	GL21_14/E2
S4_3715095	4	3715095	8.47E-03	GL21_14/E2
S4_3715085	4	3715085	8.47E-03	GL21_14/E2
S4_56654691	4	56654691	8.49E-03	GL35_14/E2
S4_54055164	4	54055164	8.49E-03	GL35_ac
S4_54055170	4	54055170	8.49E-03	GL35_ac
S4_50669857	4	50669857	8.50E-03	GL49_ac
S4_50669859	4	50669859	8.50E-03	GL49_ac
S4_50669861	4	50669861	8.50E-03	GL49_ac
S4_46239103	4	46239103	8.53E-03	GL7_13/E1
S4_1216332	4	1216332	8.53E-03	GL14_13/E1
S4_60584591	4	60584591	8.56E-03	GL14_14/E2
S4_62935692	4	62935692	8.58E-03	GL7_ac
S4_62935693	4	62935693	8.58E-03	GL7_ac
S4_62935703	4	62935703	8.58E-03	GL7_ac
S4_62935704	4	62935704	8.58E-03	GL7_ac
S4_62935747	4	62935747	8.58E-03	GL7_ac
S4_1216332	4	1216332	8.59E-03	GL21_ac
S4_19288511	4	19288511	8.60E-03	GL49_13/E1
S4_36263190	4	36263190	8.61E-03	GL28_14/E2
S4_51839386	4	51839386	8.61E-03	GL49_ac
S4_4553845	4	4553845	8.69E-03	GL7_ac
S4_62935694	4	62935694	8.78E-03	GL7_ac
S4_40351832	4	40351832	8.85E-03	GL42_13/E1
S4_5561087	4	5561087	8.92E-03	GL28_13/E1
S4_41454636	4	41454636	8.95E-03	GL35_14/E2
S4_41454638	4	41454638	8.95E-03	GL35_14/E2
S4_41454640	4	41454640	8.95E-03	GL35_14/E2
S4_41454637	4	41454637	8.95E-03	GL35_14/E2
S4_5015976	4	5015976	8.96E-03	GL7_ac
S4_39246023	4	39246023	9.00E-03	GL21_13/E1
S4_6775390	4	6775390	9.03E-03	GL35_13/E1
S4_6775390	4	6775390	9.03E-03	GL35_13/E1
S4_58490586	4	58490586	9.03E-03	GL42_14/E2
S4_46239103	4	46239103	9.07E-03	GL21_ac
S4_51460491	4	51460491	9.08E-03	GL42_ac
S4_1238210	4	1238210	9.09E-03	GL49_14/E2
S4_40071868	4	40071868	9.10E-03	GL28_13/E1

SNP	Chromosome	Position	P.value	Trait
S4_43523383	4	43523383	9.21E-03	GL28_13/E1
S4_12722232	4	12722232	9.31E-03	GL21_14/E2
S4_56557333	4	56557333	9.32E-03	GL14_ac
S4_24739885	4	24739885	9.33E-03	GL14_13/E1
S4_21752328	4	21752328	9.36E-03	GL14_14/E2
S4_55546064	4	55546064	9.42E-03	GL7_ac
S4_55546078	4	55546078	9.42E-03	GL7_ac
S4_43825975	4	43825975	9.42E-03	GL49_14/E2
S4_67712021	4	67712021	9.44E-03	GL35_14/E2
S4_19288511	4	19288511	9.47E-03	GL7_14/E2
S4_8916481	4	8916481	9.47E-03	GL42_13/E1
S4_8916473	4	8916473	9.47E-03	GL42_13/E1
S4_43064226	4	43064226	9.50E-03	GL28_13/E1
S4_40071868	4	40071868	9.51E-03	GL42_13/E1
S4_51199590	4	51199590	9.53E-03	GL21_ac
S4_51199593	4	51199593	9.53E-03	GL21_ac
S4_48092800	4	48092800	9.55E-03	GL21_ac
S4_55454594	4	55454594	9.57E-03	GL14_14/E2
S4_55454617	4	55454617	9.57E-03	GL14_14/E2
S4_56654691	4	56654691	9.60E-03	GL42_14/E2
S4_51460491	4	51460491	9.61E-03	GL35_13/E1
S4_56427895	4	56427895	9.62E-03	GL14_14/E2
S4_56427923	4	56427923	9.62E-03	GL14_14/E2
S4_56427893	4	56427893	9.62E-03	GL14_14/E2
S4_56427894	4	56427894	9.62E-03	GL14_14/E2
S4_56427898	4	56427898	9.62E-03	GL14_14/E2
S4_55546064	4	55546064	9.64E-03	GL14_ac
S4_55546078	4	55546078	9.64E-03	GL14_ac
S4_45490523	4	45490523	9.64E-03	GL14_14/E2
S4_9770382	4	9770382	9.66E-03	GL49_ac
S4_9770406	4	9770406	9.66E-03	GL49_ac
S4_9770407	4	9770407	9.66E-03	GL49_ac
S4_9770365	4	9770365	9.66E-03	GL49_ac
S4_16256981	4	16256981	9.66E-03	GL42_13/E1
S4_8916481	4	8916481	9.68E-03	GL35_13/E1
S4_8916473	4	8916473	9.68E-03	GL35_13/E1
S4_39876042	4	39876042	9.68E-03	GL42_14/E2
S4_20672814	4	20672814	9.74E-03	GL28_13/E1
S4_68098051	4	68098051	9.75E-03	GL14_14/E2
S4_68098036	4	68098036	9.75E-03	GL14_14/E2
S4_68098045	4	68098045	9.75E-03	GL14_14/E2
S4_68098053	4	68098053	9.75E-03	GL14_14/E2
S4_60584591	4	60584591	9.77E-03	GL28_14/E2
S4_39654579	4	39654579	9.80E-03	GL49_13/E1
S4_6756630	4	6756630	9.86E-03	GL14_14/E2
S4_51199590	4	51199590	9.86E-03	GL42_13/E1
S4_51199593	4	51199593	9.86E-03	GL42_13/E1
S4_34662222	4	34662222	9.89E-03	GL21_ac
S4_7277550	4	7277550	9.90E-03	GL42_13/E1
S4_56097128	4	56097128	9.94E-03	GL7_ac
S4_41454639	4	41454639	9.95E-03	GL35_14/E2

SNP	Chromosome	Position	P.value	Trait
S4_56522702	4	56522702	9.96E-03	GL7_13/E1
S5_2047156	5	2047156	1.78E-05	GL42_13/E1
S5_2047156	5	2047156	1.14E-04	GL35_13/E1
S5_9181075	5	9181075	1.24E-04	GL7_14/E2
S5_9181082	5	9181082	1.24E-04	GL7_14/E2
S5_9181090	5	9181090	1.24E-04	GL7_14/E2
S5_8604813	5	8604813	1.37E-04	GL28_ac
S5_5262919	5	5262919	1.90E-04	GL28_14/E2
S5_62686361	5	62686361	1.92E-04	GL42_14/E2
S5_4866177	5	4866177	2.12E-04	GL21_14/E2
S5_3193004	5	3193004	2.81E-04	GL42_13/E1
S5_8604813	5	8604813	2.98E-04	GL21_ac
S5_8604813	5	8604813	3.04E-04	GL35_13/E1
S5_3134227	5	3134227	3.32E-04	GL49_13/E1
S5_7018899	5	7018899	3.62E-04	GL21_13/E1
S5_7018926	5	7018926	3.62E-04	GL21_13/E1
S5_8994776	5	8994776	4.01E-04	GL7_14/E2
S5_3193004	5	3193004	4.62E-04	GL28_13/E1
S5_7018899	5	7018899	5.36E-04	GL35_13/E1
S5_7018926	5	7018926	5.36E-04	GL35_13/E1
S5_8604813	5	8604813	5.56E-04	GL28_13/E1
S5_46702355	5	46702355	5.72E-04	GL49_14/E2
S5_12855792	5	12855792	5.89E-04	GL49_14/E2
S5_2047156	5	2047156	5.95E-04	GL28_13/E1
S5_4866177	5	4866177	6.04E-04	GL28_14/E2
S5_3193004	5	3193004	6.22E-04	GL49_13/E1
S5_3038898	5	3038898	6.33E-04	GL42_13/E1
S5_8604813	5	8604813	6.50E-04	GL21_13/E1
S5_9550350	5	9550350	6.60E-04	GL49_14/E2
S5_7018899	5	7018899	6.61E-04	GL28_13/E1
S5_7018926	5	7018926	6.61E-04	GL28_13/E1
S5_4866177	5	4866177	7.12E-04	GL14_14/E2
S5_8604896	5	8604896	7.80E-04	GL7_14/E2
S5_8604908	5	8604908	7.95E-04	GL7_14/E2
S5_62499915	5	62499915	7.97E-04	GL35_13/E1
S5_62499919	5	62499919	7.97E-04	GL35_13/E1
S5_2047156	5	2047156	8.03E-04	GL28_ac
S5_12536061	5	12536061	8.53E-04	GL35_14/E2
S5_12536085	5	12536085	8.53E-04	GL35_14/E2
S5_12536087	5	12536087	8.53E-04	GL35_14/E2
S5_12536072	5	12536072	8.53E-04	GL35_14/E2
S5_3134227	5	3134227	9.78E-04	GL42_13/E1
S5_11055457	5	11055457	1.01E-03	GL7_13/E1
S5_3193004	5	3193004	1.05E-03	GL21_13/E1
S5_57276203	5	57276203	1.08E-03	GL49_13/E1
S5_4866177	5	4866177	1.09E-03	GL35_14/E2
S5_10420146	5	10420146	1.15E-03	GL21_14/E2
S5_2692983	5	2692983	1.24E-03	GL42_13/E1
S5_6991342	5	6991342	1.29E-03	GL28_14/E2
S5_5262919	5	5262919	1.37E-03	GL49_13/E1
S5_11055457	5	11055457	1.37E-03	GL14_13/E1

SNP	Chromosome	Position	P.value	Trait
S5_4866177	5	4866177	1.41E-03	GL35_ac
S5_8604813	5	8604813	1.42E-03	GL35_ac
S5_35518301	5	35518301	1.47E-03	GL35_14/E2
S5_4866177	5	4866177	1.48E-03	GL21_ac
S5_62492944	5	62492944	1.49E-03	GL7_14/E2
S5_62492964	5	62492964	1.49E-03	GL7_14/E2
S5_7018899	5	7018899	1.51E-03	GL21_ac
S5_7018926	5	7018926	1.51E-03	GL21_ac
S5_38827168	5	38827168	1.55E-03	GL7_14/E2
S5_4866177	5	4866177	1.62E-03	GL49_ac
S5_7018899	5	7018899	1.63E-03	GL42_13/E1
S5_7018926	5	7018926	1.63E-03	GL42_13/E1
S5_3865103	5	3865103	1.71E-03	GL49_13/E1
S5_62499915	5	62499915	1.76E-03	GL49_13/E1
S5_62499919	5	62499919	1.76E-03	GL49_13/E1
S5_62729427	5	62729427	1.82E-03	GL28_14/E2
S5_7018899	5	7018899	1.83E-03	GL7_13/E1
S5_7018926	5	7018926	1.83E-03	GL7_13/E1
S5_8735555	5	8735555	1.85E-03	GL14_ac
S5_4509065	5	4509065	1.85E-03	GL42_13/E1
S5_4509074	5	4509074	1.85E-03	GL42_13/E1
S5_8695103	5	8695103	1.85E-03	GL7_14/E2
S5_2692983	5	2692983	1.88E-03	GL49_13/E1
S5_7018899	5	7018899	1.89E-03	GL14_13/E1
S5_7018926	5	7018926	1.89E-03	GL14_13/E1
S5_1932849	5	1932849	1.93E-03	GL35_ac
S5_1932855	5	1932855	1.93E-03	GL35_ac
S5_62499915	5	62499915	1.93E-03	GL42_13/E1
S5_62499919	5	62499919	1.93E-03	GL42_13/E1
S5_57276203	5	57276203	1.96E-03	GL28_13/E1
S5_12365060	5	12365060	2.00E-03	GL42_13/E1
S5_2047156	5	2047156	2.01E-03	GL21_ac
S5_11472805	5	11472805	2.06E-03	GL49_14/E2
S5_8735555	5	8735555	2.10E-03	GL7_13/E1
S5_61921236	5	61921236	2.13E-03	GL42_13/E1
S5_61921241	5	61921241	2.13E-03	GL42_13/E1
S5_6765184	5	6765184	2.24E-03	GL42_14/E2
S5_4866177	5	4866177	2.28E-03	GL14_ac
S5_6972135	5	6972135	2.36E-03	GL28_13/E1
S5_12308132	5	12308132	2.39E-03	GL7_14/E2
S5_4866177	5	4866177	2.42E-03	GL28_ac
S5_57276203	5	57276203	2.44E-03	GL35_13/E1
S5_12715925	5	12715925	2.45E-03	GL7_14/E2
S5_8735555	5	8735555	2.45E-03	GL21_14/E2
S5_4000601	5	4000601	2.45E-03	GL49_13/E1
S5_6781664	5	6781664	2.61E-03	GL28_14/E2
S5_9053125	5	9053125	2.64E-03	GL7_14/E2
S5_7242271	5	7242271	2.77E-03	GL14_13/E1
S5_5262919	5	5262919	2.78E-03	GL7_14/E2
S5_9053124	5	9053124	2.86E-03	GL7_14/E2
S5_9181075	5	9181075	2.88E-03	GL28_14/E2

SNP	Chromosome	Position	P.value	Trait
S5_9181082	5	9181082	2.88E-03	GL28_14/E2
S5_9181090	5	9181090	2.88E-03	GL28_14/E2
S5_7242271	5	7242271	2.93E-03	GL7_13/E1
S5_68758354	5	68758354	3.01E-03	GL49_13/E1
S5_46702355	5	46702355	3.01E-03	GL7_14/E2
S5_4560293	5	4560293	3.05E-03	GL28_14/E2
S5_3193004	5	3193004	3.06E-03	GL35_13/E1
S5_3193004	5	3193004	3.06E-03	GL7_13/E1
S5_9550350	5	9550350	3.15E-03	GL7_14/E2
S5_38471416	5	38471416	3.16E-03	GL49_14/E2
S5_2692983	5	2692983	3.17E-03	GL28_13/E1
S5_3193004	5	3193004	3.17E-03	GL21_ac
S5_11055457	5	11055457	3.21E-03	GL7_ac
S5_1932849	5	1932849	3.24E-03	GL14_ac
S5_1932855	5	1932855	3.24E-03	GL14_ac
S5_5262919	5	5262919	3.35E-03	GL21_14/E2
S5_8604813	5	8604813	3.37E-03	GL14_ac
S5_8735555	5	8735555	3.37E-03	GL28_14/E2
S5_4509065	5	4509065	3.38E-03	GL35_13/E1
S5_4509074	5	4509074	3.38E-03	GL35_13/E1
S5_57276203	5	57276203	3.39E-03	GL42_13/E1
S5_8695135	5	8695135	3.39E-03	GL7_14/E2
S5_9053126	5	9053126	3.39E-03	GL7_14/E2
S5_9053127	5	9053127	3.39E-03	GL7_14/E2
S5_9053128	5	9053128	3.39E-03	GL7_14/E2
S5_9053129	5	9053129	3.39E-03	GL7_14/E2
S5_9053130	5	9053130	3.39E-03	GL7_14/E2
S5_63844658	5	63844658	3.42E-03	GL49_14/E2
S5_3613434	5	3613434	3.45E-03	GL49_13/E1
S5_8735555	5	8735555	3.48E-03	GL7_ac
S5_62173070	5	62173070	3.51E-03	GL42_13/E1
S5_8735555	5	8735555	3.55E-03	GL28_ac
S5_8735555	5	8735555	3.56E-03	GL14_13/E1
S5_1932849	5	1932849	3.56E-03	GL21_ac
S5_1932855	5	1932855	3.56E-03	GL21_ac
S5_2047156	5	2047156	3.56E-03	GL21_13/E1
S5_57276203	5	57276203	3.64E-03	GL7_13/E1
S5_7721956	5	7721956	3.65E-03	GL21_14/E2
S5_8696398	5	8696398	3.74E-03	GL49_ac
S5_62509203	5	62509203	3.75E-03	GL28_14/E2
S5_4866177	5	4866177	3.75E-03	GL28_13/E1
S5_57276203	5	57276203	3.80E-03	GL21_13/E1
S5_62499915	5	62499915	3.82E-03	GL42_ac
S5_62499919	5	62499919	3.82E-03	GL42_ac
S5_4866177	5	4866177	3.84E-03	GL21_13/E1
S5_9537136	5	9537136	3.85E-03	GL14_14/E2
S5_9537142	5	9537142	3.85E-03	GL14_14/E2
S5_2968842	5	2968842	3.88E-03	GL42_13/E1
S5_9537136	5	9537136	3.88E-03	GL35_14/E2
S5_9537142	5	9537142	3.88E-03	GL35_14/E2
S5_68185691	5	68185691	3.92E-03	GL14_14/E2

SNP	Chromosome	Position	P.value	Trait
S5_66909528	5	66909528	3.92E-03	GL42_14/E2
S5_66487485	5	66487485	4.00E-03	GL49_13/E1
S5_66487518	5	66487518	4.00E-03	GL49_13/E1
S5_6972135	5	6972135	4.01E-03	GL21_13/E1
S5_63272413	5	63272413	4.08E-03	GL14_13/E1
S5_614663	5	614663	4.08E-03	GL42_13/E1
S5_614669	5	614669	4.08E-03	GL42_13/E1
S5_4509059	5	4509059	4.12E-03	GL28_14/E2
S5_14745074	5	14745074	4.12E-03	GL28_13/E1
S5_4866177	5	4866177	4.12E-03	GL7_ac
S5_8735555	5	8735555	4.15E-03	GL21_ac
S5_7283752	5	7283752	4.18E-03	GL7_ac
S5_7283810	5	7283810	4.18E-03	GL7_ac
S5_12308132	5	12308132	4.19E-03	GL49_14/E2
S5_10759631	5	10759631	4.21E-03	GL28_14/E2
S5_5262919	5	5262919	4.30E-03	GL28_ac
S5_7721956	5	7721956	4.40E-03	GL14_14/E2
S5_8604813	5	8604813	4.40E-03	GL14_13/E1
S5_8735555	5	8735555	4.42E-03	GL7_14/E2
S5_2047156	5	2047156	4.47E-03	GL49_13/E1
S5_6972135	5	6972135	4.53E-03	GL7_13/E1
S5_68185691	5	68185691	4.54E-03	GL21_14/E2
S5_6630988	5	6630988	4.63E-03	GL28_14/E2
S5_8994776	5	8994776	4.64E-03	GL7_13/E1
S5_4923530	5	4923530	4.64E-03	GL49_13/E1
S5_62492964	5	62492964	4.67E-03	GL49_ac
S5_62492944	5	62492944	4.67E-03	GL49_ac
S5_11347038	5	11347038	4.67E-03	GL35_14/E2
S5_63844658	5	63844658	4.67E-03	GL7_14/E2
S5_3193004	5	3193004	4.69E-03	GL28_ac
S5_614663	5	614663	4.69E-03	GL21_13/E1
S5_614669	5	614669	4.69E-03	GL21_13/E1
S5_614663	5	614663	4.72E-03	GL28_13/E1
S5_614669	5	614669	4.72E-03	GL28_13/E1
S5_15311867	5	15311867	4.82E-03	GL7_14/E2
S5_67767099	5	67767099	4.83E-03	GL42_13/E1
S5_8604813	5	8604813	4.83E-03	GL42_13/E1
S5_11891529	5	11891529	4.85E-03	GL7_14/E2
S5_57276203	5	57276203	4.86E-03	GL14_13/E1
S5_8702688	5	8702688	4.96E-03	GL7_14/E2
S5_58692184	5	58692184	4.97E-03	GL7_ac
S5_3016226	5	3016226	5.00E-03	GL49_13/E1
S5_8668415	5	8668415	5.01E-03	GL42_14/E2
S5_8668419	5	8668419	5.01E-03	GL42_14/E2
S5_8668429	5	8668429	5.01E-03	GL42_14/E2
S5_3282948	5	3282948	5.04E-03	GL42_13/E1
S5_11779471	5	11779471	5.07E-03	GL7_14/E2
S5_11779477	5	11779477	5.07E-03	GL7_14/E2
S5_11055457	5	11055457	5.07E-03	GL21_13/E1
S5_35518301	5	35518301	5.12E-03	GL49_14/E2
S5_66430109	5	66430109	5.22E-03	GL49_14/E2

SNP	Chromosome	Position	P.value	Trait
S5_66430112	5	66430112	5.22E-03	GL49_14/E2
S5_6765184	5	6765184	5.26E-03	GL49_14/E2
S5_12855792	5	12855792	5.28E-03	GL35_14/E2
S5_40360109	5	40360109	5.29E-03	GL49_14/E2
S5_18528466	5	18528466	5.36E-03	GL49_14/E2
S5_61929439	5	61929439	5.42E-03	GL35_14/E2
S5_61929441	5	61929441	5.42E-03	GL35_14/E2
S5_61929454	5	61929454	5.42E-03	GL35_14/E2
S5_39054517	5	39054517	5.51E-03	GL35_14/E2
S5_1932849	5	1932849	5.54E-03	GL28_ac
S5_1932855	5	1932855	5.54E-03	GL28_ac
S5_68758354	5	68758354	5.55E-03	GL42_13/E1
S5_15248013	5	15248013	5.58E-03	GL28_14/E2
S5_67718081	5	67718081	5.60E-03	GL7_14/E2
S5_50504935	5	50504935	5.61E-03	GL7_14/E2
S5_61681577	5	61681577	5.61E-03	GL42_14/E2
S5_4866177	5	4866177	5.63E-03	GL42_14/E2
S5_18528466	5	18528466	5.64E-03	GL35_14/E2
S5_2669059	5	2669059	5.66E-03	GL28_13/E1
S5_2571570	5	2571570	5.72E-03	GL7_14/E2
S5_8668415	5	8668415	5.74E-03	GL49_14/E2
S5_8668419	5	8668419	5.74E-03	GL49_14/E2
S5_8668429	5	8668429	5.74E-03	GL49_14/E2
S5_7018899	5	7018899	5.75E-03	GL28_ac
S5_7018926	5	7018926	5.75E-03	GL28_ac
S5_8994776	5	8994776	5.78E-03	GL14_13/E1
S5_68963917	5	68963917	5.81E-03	GL42_13/E1
S5_62686361	5	62686361	5.81E-03	GL49_14/E2
S5_6972135	5	6972135	5.83E-03	GL14_13/E1
S5_68185691	5	68185691	5.84E-03	GL42_14/E2
S5_3016226	5	3016226	5.85E-03	GL49_ac
S5_3193004	5	3193004	5.86E-03	GL14_13/E1
S5_7641620	5	7641620	5.90E-03	GL7_14/E2
S5_7018899	5	7018899	5.95E-03	GL49_13/E1
S5_7018926	5	7018926	5.95E-03	GL49_13/E1
S5_20196453	5	20196453	6.00E-03	GL35_13/E1
S5_8696398	5	8696398	6.01E-03	GL7_ac
S5_7018899	5	7018899	6.07E-03	GL14_ac
S5_7018926	5	7018926	6.07E-03	GL14_ac
S5_63272413	5	63272413	6.07E-03	GL21_13/E1
S5_3282948	5	3282948	6.11E-03	GL49_13/E1
S5_8702688	5	8702688	6.18E-03	GL28_14/E2
S5_7840232	5	7840232	6.19E-03	GL7_14/E2
S5_17390774	5	17390774	6.22E-03	GL7_ac
S5_68031054	5	68031054	6.24E-03	GL49_14/E2
S5_11779425	5	11779425	6.28E-03	GL7_14/E2
S5_11779444	5	11779444	6.28E-03	GL7_14/E2
S5_9550350	5	9550350	6.41E-03	GL7_ac
S5_9307319	5	9307319	6.43E-03	GL7_13/E1
S5_1152721	5	1152721	6.44E-03	GL21_13/E1
S5_6024239	5	6024239	6.45E-03	GL49_13/E1

SNP	Chromosome	Position	P.value	Trait
S5_6024243	5	6024243	6.45E-03	GL49_13/E1
S5_7295411	5	7295411	6.46E-03	GL49_14/E2
S5_3876828	5	3876828	6.48E-03	GL49_13/E1
S5_7351468	5	7351468	6.50E-03	GL7_13/E1
S5_8604813	5	8604813	6.51E-03	GL42_ac
S5_1795586	5	1795586	6.52E-03	GL35_14/E2
S5_1795590	5	1795590	6.52E-03	GL35_14/E2
S5_8695893	5	8695893	6.60E-03	GL7_14/E2
S5_10493196	5	10493196	6.63E-03	GL14_14/E2
S5_10493199	5	10493199	6.63E-03	GL14_14/E2
S5_10493200	5	10493200	6.63E-03	GL14_14/E2
S5_10493195	5	10493195	6.63E-03	GL14_14/E2
S5_10493197	5	10493197	6.63E-03	GL14_14/E2
S5_10493201	5	10493201	6.63E-03	GL14_14/E2
S5_6866425	5	6866425	6.66E-03	GL42_14/E2
S5_614663	5	614663	6.66E-03	GL35_13/E1
S5_614669	5	614669	6.66E-03	GL35_13/E1
S5_62499915	5	62499915	6.66E-03	GL28_13/E1
S5_62499919	5	62499919	6.66E-03	GL28_13/E1
S5_63844658	5	63844658	6.66E-03	GL49_ac
S5_3134227	5	3134227	6.70E-03	GL35_13/E1
S5_55298105	5	55298105	6.71E-03	GL49_ac
S5_10354509	5	10354509	6.81E-03	GL7_14/E2
S5_12715925	5	12715925	6.82E-03	GL7_ac
S5_12536061	5	12536061	6.83E-03	GL7_ac
S5_12536085	5	12536085	6.83E-03	GL7_ac
S5_12536087	5	12536087	6.83E-03	GL7_ac
S5_12536072	5	12536072	6.83E-03	GL7_ac
S5_8696398	5	8696398	6.84E-03	GL49_14/E2
S5_4000601	5	4000601	6.89E-03	GL42_13/E1
S5_63844658	5	63844658	6.92E-03	GL7_ac
S5_10493196	5	10493196	6.97E-03	GL28_14/E2
S5_10493199	5	10493199	6.97E-03	GL28_14/E2
S5_10493200	5	10493200	6.97E-03	GL28_14/E2
S5_10493195	5	10493195	6.97E-03	GL28_14/E2
S5_10493197	5	10493197	6.97E-03	GL28_14/E2
S5_10493201	5	10493201	6.97E-03	GL28_14/E2
S5_8695893	5	8695893	7.00E-03	GL7_13/E1
S5_62686361	5	62686361	7.01E-03	GL42_ac
S5_3187334	5	3187334	7.02E-03	GL42_13/E1
S5_7006744	5	7006744	7.03E-03	GL49_13/E1
S5_3626674	5	3626674	7.31E-03	GL49_13/E1
S5_62492964	5	62492964	7.33E-03	GL49_14/E2
S5_62492944	5	62492944	7.33E-03	GL49_14/E2
S5_67529207	5	67529207	7.35E-03	GL42_ac
S5_10759631	5	10759631	7.36E-03	GL21_14/E2
S5_67670581	5	67670581	7.38E-03	GL49_14/E2
S5_67670582	5	67670582	7.38E-03	GL49_14/E2
S5_67670584	5	67670584	7.38E-03	GL49_14/E2
S5_67670586	5	67670586	7.38E-03	GL49_14/E2
S5_2845565	5	2845565	7.40E-03	GL35_13/E1

SNP	Chromosome	Position	P.value	Trait
S5_6972135	5	6972135	7.41E-03	GL35_13/E1
S5_4499186	5	4499186	7.54E-03	GL28_13/E1
S5_4499193	5	4499193	7.54E-03	GL28_13/E1
S5_4499195	5	4499195	7.54E-03	GL28_13/E1
S5_5262919	5	5262919	7.56E-03	GL21_13/E1
S5_46702355	5	46702355	7.58E-03	GL49_ac
S5_4000601	5	4000601	7.61E-03	GL28_13/E1
S5_61921236	5	61921236	7.66E-03	GL49_13/E1
S5_61921241	5	61921241	7.66E-03	GL49_13/E1
S5_14745074	5	14745074	7.67E-03	GL21_13/E1
S5_14745074	5	14745074	7.67E-03	GL14_13/E1
S5_2692983	5	2692983	7.68E-03	GL35_13/E1
S5_61921236	5	61921236	7.79E-03	GL35_13/E1
S5_61921241	5	61921241	7.79E-03	GL35_13/E1
S5_5262919	5	5262919	7.85E-03	GL7_ac
S5_12365060	5	12365060	7.90E-03	GL35_13/E1
S5_6630988	5	6630988	7.92E-03	GL42_14/E2
S5_10814315	5	10814315	8.03E-03	GL42_13/E1
S5_66965812	5	66965812	8.08E-03	GL49_14/E2
S5_68758354	5	68758354	8.11E-03	GL35_13/E1
S5_11472805	5	11472805	8.14E-03	GL49_ac
S5_4509065	5	4509065	8.19E-03	GL49_13/E1
S5_4509074	5	4509074	8.19E-03	GL49_13/E1
S5_4509059	5	4509059	8.21E-03	GL49_13/E1
S5_3620138	5	3620138	8.22E-03	GL42_14/E2
S5_1795586	5	1795586	8.24E-03	GL49_14/E2
S5_1795590	5	1795590	8.24E-03	GL49_14/E2
S5_437471	5	437471	8.24E-03	GL28_13/E1
S5_437471	5	437471	8.24E-03	GL28_13/E1
S5_437541	5	437541	8.24E-03	GL28_13/E1
S5_61931981	5	61931981	8.29E-03	GL49_14/E2
S5_61577399	5	61577399	8.32E-03	GL42_13/E1
S5_47972099	5	47972099	8.36E-03	GL49_14/E2
S5_10195816	5	10195816	8.37E-03	GL7_14/E2
S5_63307674	5	63307674	8.42E-03	GL28_14/E2
S5_3038898	5	3038898	8.46E-03	GL28_13/E1
S5_8604813	5	8604813	8.56E-03	GL49_13/E1
S5_46702355	5	46702355	8.63E-03	GL7_ac
S5_4130475	5	4130475	8.67E-03	GL49_13/E1
S5_8994776	5	8994776	8.69E-03	GL7_ac
S5_3876828	5	3876828	8.72E-03	GL28_13/E1
S5_3613434	5	3613434	8.73E-03	GL28_13/E1
S5_3829915	5	3829915	8.74E-03	GL42_13/E1
S5_68185691	5	68185691	8.76E-03	GL35_14/E2
S5_7192179	5	7192179	8.85E-03	GL21_14/E2
S5_2278268	5	2278268	8.87E-03	GL28_13/E1
S5_2278291	5	2278291	8.87E-03	GL28_13/E1
S5_6972135	5	6972135	8.90E-03	GL21_ac
S5_15208630	5	15208630	8.91E-03	GL42_13/E1
S5_2692983	5	2692983	8.95E-03	GL7_13/E1
S5_63272413	5	63272413	9.03E-03	GL28_13/E1

SNP	Chromosome	Position	P.value	Trait
S5_62492964	5	62492964	9.08E-03	GL14_14/E2
S5_62492944	5	62492944	9.08E-03	GL14_14/E2
S5_7267098	5	7267098	9.10E-03	GL21_14/E2
S5_2845565	5	2845565	9.19E-03	GL42_13/E1
S5_12857271	5	12857271	9.24E-03	GL42_13/E1
S5_7351468	5	7351468	9.24E-03	GL21_13/E1
S5_63272413	5	63272413	9.24E-03	GL21_ac
S5_10420146	5	10420146	9.33E-03	GL14_14/E2
S5_10818778	5	10818778	9.35E-03	GL7_14/E2
S5_3187334	5	3187334	9.39E-03	GL49_13/E1
S5_2219901	5	2219901	9.41E-03	GL28_13/E1
S5_10555635	5	10555635	9.43E-03	GL21_14/E2
S5_8604813	5	8604813	9.43E-03	GL7_ac
S5_2968842	5	2968842	9.51E-03	GL28_13/E1
S5_10818778	5	10818778	9.70E-03	GL21_14/E2
S5_3134227	5	3134227	9.70E-03	GL21_13/E1
S5_9550350	5	9550350	9.72E-03	GL49_ac
S5_10420146	5	10420146	9.75E-03	GL21_ac
S5_4866177	5	4866177	9.81E-03	GL7_13/E1
S5_62499915	5	62499915	9.85E-03	GL35_ac
S5_62499919	5	62499919	9.85E-03	GL35_ac
S5_9307319	5	9307319	9.92E-03	GL7_ac
S5_3876828	5	3876828	9.95E-03	GL42_13/E1
S5_1795586	5	1795586	9.96E-03	GL42_14/E2
S5_1795590	5	1795590	9.96E-03	GL42_14/E2
S5_12855792	5	12855792	9.98E-03	GL42_14/E2
S6_54448658	6	54448658	1.60E-04	GL28_ac
S6_809010	6	809010	3.19E-04	GL7_ac
S6_31754242	6	31754242	3.41E-04	GL28_ac
S6_44610491	6	44610491	3.78E-04	GL21_13/E1
S6_19400188	6	19400188	4.43E-04	GL49_14/E2
S6_31754242	6	31754242	4.90E-04	GL21_ac
S6_15331131	6	15331131	5.00E-04	GL28_14/E2
S6_15331130	6	15331130	5.00E-04	GL28_14/E2
S6_15331131	6	15331131	5.22E-04	GL21_ac
S6_15331130	6	15331130	5.22E-04	GL21_ac
S6_15331131	6	15331131	5.60E-04	GL28_ac
S6_15331130	6	15331130	5.60E-04	GL28_ac
S6_44610491	6	44610491	5.87E-04	GL35_13/E1
S6_15331131	6	15331131	6.55E-04	GL21_14/E2
S6_15331130	6	15331130	6.55E-04	GL21_14/E2
S6_304009	6	304009	8.00E-04	GL35_ac
S6_58035596	6	58035596	8.09E-04	GL14_ac
S6_54448658	6	54448658	8.19E-04	GL21_ac
S6_18915692	6	18915692	9.22E-04	GL42_ac
S6_44610491	6	44610491	9.40E-04	GL28_13/E1
S6_18915692	6	18915692	9.69E-04	GL42_14/E2
S6_44610491	6	44610491	9.80E-04	GL42_13/E1
S6_52087285	6	52087285	1.02E-03	GL7_14/E2
S6_52087290	6	52087290	1.02E-03	GL7_14/E2
S6_52087308	6	52087308	1.02E-03	GL7_14/E2

SNP	Chromosome	Position	P.value	Trait
S6_17356403	6	17356403	1.07E-03	GL49_ac
S6_17356407	6	17356407	1.07E-03	GL49_ac
S6_17356361	6	17356361	1.07E-03	GL49_ac
S6_38161717	6	38161717	1.08E-03	GL7_ac
S6_38161730	6	38161730	1.08E-03	GL7_ac
S6_51539086	6	51539086	1.10E-03	GL28_14/E2
S6_6458325	6	6458325	1.16E-03	GL49_13/E1
S6_19400188	6	19400188	1.16E-03	GL49_ac
S6_44610491	6	44610491	1.17E-03	GL7_13/E1
S6_334458	6	334458	1.24E-03	GL7_ac
S6_822538	6	822538	1.29E-03	GL7_ac
S6_822527	6	822527	1.29E-03	GL7_ac
S6_4090558	6	4090558	1.29E-03	GL49_ac
S6_4092964	6	4092964	1.29E-03	GL7_ac
S6_26788357	6	26788357	1.30E-03	GL7_ac
S6_26788359	6	26788359	1.30E-03	GL7_ac
S6_809010	6	809010	1.34E-03	GL49_13/E1
S6_36408670	6	36408670	1.43E-03	GL49_14/E2
S6_3643098	6	3643098	1.43E-03	GL49_ac
S6_48659560	6	48659560	1.45E-03	GL28_ac
S6_54448658	6	54448658	1.48E-03	GL14_ac
S6_2598827	6	2598827	1.50E-03	GL7_ac
S6_54257355	6	54257355	1.52E-03	GL42_ac
S6_54257380	6	54257380	1.52E-03	GL42_ac
S6_15331131	6	15331131	1.52E-03	GL14_ac
S6_15331130	6	15331130	1.52E-03	GL14_ac
S6_3643098	6	3643098	1.52E-03	GL14_14/E2
S6_41604283	6	41604283	1.64E-03	GL7_ac
S6_953196	6	953196	1.67E-03	GL7_ac
S6_48659560	6	48659560	1.68E-03	GL21_ac
S6_30816124	6	30816124	1.75E-03	GL7_ac
S6_53506008	6	53506008	1.80E-03	GL49_14/E2
S6_53506011	6	53506011	1.80E-03	GL49_14/E2
S6_53506012	6	53506012	1.80E-03	GL49_14/E2
S6_6738840	6	6738840	1.81E-03	GL42_ac
S6_822538	6	822538	1.82E-03	GL28_14/E2
S6_822527	6	822527	1.82E-03	GL28_14/E2
S6_19400188	6	19400188	1.83E-03	GL7_ac
S6_47626872	6	47626872	1.86E-03	GL7_ac
S6_47626871	6	47626871	1.86E-03	GL7_ac
S6_47626874	6	47626874	1.86E-03	GL7_ac
S6_6977745	6	6977745	1.87E-03	GL42_13/E1
S6_58035596	6	58035596	1.89E-03	GL21_ac
S6_31754242	6	31754242	1.91E-03	GL14_ac
S6_43554708	6	43554708	1.94E-03	GL49_13/E1
S6_39651119	6	39651119	1.96E-03	GL28_14/E2
S6_47700046	6	47700046	1.97E-03	GL14_ac
S6_1306514	6	1306514	1.98E-03	GL7_ac
S6_1306527	6	1306527	1.98E-03	GL7_ac
S6_47626872	6	47626872	2.01E-03	GL21_ac
S6_47626871	6	47626871	2.01E-03	GL21_ac

SNP	Chromosome	Position	P.value	Trait
S6_47626874	6	47626874	2.01E-03	GL21_ac
S6_31754242	6	31754242	2.06E-03	GL7_ac
S6_4092964	6	4092964	2.07E-03	GL49_14/E2
S6_6458325	6	6458325	2.09E-03	GL28_13/E1
S6_28730716	6	28730716	2.09E-03	GL49_14/E2
S6_53154736	6	53154736	2.11E-03	GL7_14/E2
S6_53641290	6	53641290	2.12E-03	GL28_13/E1
S6_1391724	6	1391724	2.13E-03	GL7_ac
S6_53641290	6	53641290	2.13E-03	GL42_13/E1
S6_36408670	6	36408670	2.19E-03	GL42_ac
S6_36408670	6	36408670	2.24E-03	GL42_14/E2
S6_809010	6	809010	2.27E-03	GL28_ac
S6_54448658	6	54448658	2.28E-03	GL35_ac
S6_38162288	6	38162288	2.28E-03	GL21_ac
S6_38162288	6	38162288	2.29E-03	GL14_ac
S6_45176024	6	45176024	2.29E-03	GL42_ac
S6_45176035	6	45176035	2.29E-03	GL42_ac
S6_29130191	6	29130191	2.32E-03	GL28_14/E2
S6_29130176	6	29130176	2.32E-03	GL28_14/E2
S6_17520983	6	17520983	2.35E-03	GL7_ac
S6_46569258	6	46569258	2.37E-03	GL49_14/E2
S6_53353798	6	53353798	2.37E-03	GL35_ac
S6_44610491	6	44610491	2.42E-03	GL14_13/E1
S6_6738840	6	6738840	2.45E-03	GL35_ac
S6_53714413	6	53714413	2.49E-03	GL35_13/E1
S6_53154736	6	53154736	2.50E-03	GL21_14/E2
S6_31747106	6	31747106	2.60E-03	GL7_ac
S6_48659560	6	48659560	2.63E-03	GL14_ac
S6_15331126	6	15331126	2.66E-03	GL21_14/E2
S6_15331153	6	15331153	2.66E-03	GL21_14/E2
S6_15331164	6	15331164	2.66E-03	GL21_14/E2
S6_8919203	6	8919203	2.68E-03	GL21_13/E1
S6_6657906	6	6657906	2.68E-03	GL7_ac
S6_6657907	6	6657907	2.68E-03	GL7_ac
S6_6657915	6	6657915	2.68E-03	GL7_ac
S6_52485875	6	52485875	2.68E-03	GL49_14/E2
S6_52485895	6	52485895	2.68E-03	GL49_14/E2
S6_52485898	6	52485898	2.68E-03	GL49_14/E2
S6_1734778	6	1734778	2.68E-03	GL7_ac
S6_31747106	6	31747106	2.73E-03	GL21_ac
S6_2547474	6	2547474	2.80E-03	GL7_ac
S6_2547476	6	2547476	2.80E-03	GL7_ac
S6_2547479	6	2547479	2.80E-03	GL7_ac
S6_2547468	6	2547468	2.80E-03	GL7_ac
S6_358918	6	358918	2.80E-03	GL28_14/E2
S6_17559082	6	17559082	2.82E-03	GL7_ac
S6_40434431	6	40434431	2.85E-03	GL49_13/E1
S6_13915681	6	13915681	2.86E-03	GL21_ac
S6_2547431	6	2547431	2.86E-03	GL7_ac
S6_31040630	6	31040630	2.92E-03	GL42_14/E2
S6_53273004	6	53273004	2.95E-03	GL49_14/E2

SNP	Chromosome	Position	P.value	Trait
S6_4092964	6	4092964	2.95E-03	GL49_ac
S6_54168529	6	54168529	2.95E-03	GL35_14/E2
S6_54168531	6	54168531	2.95E-03	GL35_14/E2
S6_41922310	6	41922310	2.96E-03	GL7_ac
S6_53641290	6	53641290	2.97E-03	GL35_13/E1
S6_808986	6	808986	3.00E-03	GL7_ac
S6_6458325	6	6458325	3.02E-03	GL7_ac
S6_47700046	6	47700046	3.08E-03	GL7_ac
S6_53187236	6	53187236	3.10E-03	GL21_13/E1
S6_6977745	6	6977745	3.15E-03	GL35_13/E1
S6_1306615	6	1306615	3.15E-03	GL49_13/E1
S6_1306617	6	1306617	3.15E-03	GL49_13/E1
S6_1454237	6	1454237	3.15E-03	GL42_13/E1
S6_1454288	6	1454288	3.15E-03	GL42_13/E1
S6_18806973	6	18806973	3.28E-03	GL42_ac
S6_57536070	6	57536070	3.31E-03	GL28_14/E2
S6_53064987	6	53064987	3.32E-03	GL49_ac
S6_304009	6	304009	3.33E-03	GL42_ac
S6_809010	6	809010	3.37E-03	GL14_13/E1
S6_53506025	6	53506025	3.48E-03	GL49_14/E2
S6_358918	6	358918	3.49E-03	GL35_ac
S6_18915692	6	18915692	3.50E-03	GL28_ac
S6_48659560	6	48659560	3.52E-03	GL42_13/E1
S6_4090558	6	4090558	3.54E-03	GL7_ac
S6_1391724	6	1391724	3.54E-03	GL21_ac
S6_14556770	6	14556770	3.55E-03	GL7_ac
S6_18731918	6	18731918	3.57E-03	GL7_ac
S6_6458325	6	6458325	3.57E-03	GL21_13/E1
S6_54400448	6	54400448	3.60E-03	GL28_14/E2
S6_6458325	6	6458325	3.60E-03	GL14_ac
S6_8919203	6	8919203	3.61E-03	GL7_ac
S6_36255557	6	36255557	3.61E-03	GL35_ac
S6_36255558	6	36255558	3.61E-03	GL35_ac
S6_54448658	6	54448658	3.63E-03	GL35_13/E1
S6_809010	6	809010	3.65E-03	GL14_ac
S6_18731918	6	18731918	3.65E-03	GL35_ac
S6_42084968	6	42084968	3.69E-03	GL7_ac
S6_53353798	6	53353798	3.74E-03	GL28_13/E1
S6_58117354	6	58117354	3.76E-03	GL49_ac
S6_38162288	6	38162288	3.76E-03	GL7_ac
S6_49441807	6	49441807	3.82E-03	GL7_ac
S6_49441808	6	49441808	3.82E-03	GL7_ac
S6_52092883	6	52092883	3.83E-03	GL7_13/E1
S6_52092901	6	52092901	3.83E-03	GL7_13/E1
S6_52092904	6	52092904	3.83E-03	GL7_13/E1
S6_15331131	6	15331131	3.84E-03	GL14_14/E2
S6_15331130	6	15331130	3.84E-03	GL14_14/E2
S6_52599267	6	52599267	3.87E-03	GL49_14/E2
S6_58035596	6	58035596	3.87E-03	GL35_ac
S6_53714413	6	53714413	3.94E-03	GL28_13/E1
S6_52092913	6	52092913	3.95E-03	GL7_13/E1

SNP	Chromosome	Position	P.value	Trait
S6_31747106	6	31747106	3.97E-03	GL14_ac
S6_4090558	6	4090558	3.98E-03	GL49_14/E2
S6_53641290	6	53641290	3.99E-03	GL21_13/E1
S6_6458325	6	6458325	4.02E-03	GL14_13/E1
S6_6458325	6	6458325	4.03E-03	GL21_ac
S6_18806973	6	18806973	4.05E-03	GL42_14/E2
S6_8919203	6	8919203	4.12E-03	GL21_ac
S6_54448658	6	54448658	4.12E-03	GL28_14/E2
S6_6458325	6	6458325	4.18E-03	GL42_ac
S6_41922332	6	41922332	4.22E-03	GL7_ac
S6_44560660	6	44560660	4.22E-03	GL42_13/E1
S6_48659560	6	48659560	4.24E-03	GL35_ac
S6_57536070	6	57536070	4.27E-03	GL21_14/E2
S6_2667402	6	2667402	4.28E-03	GL7_ac
S6_37639790	6	37639790	4.33E-03	GL7_ac
S6_58551686	6	58551686	4.37E-03	GL42_14/E2
S6_3643098	6	3643098	4.39E-03	GL7_ac
S6_26282267	6	26282267	4.41E-03	GL7_14/E2
S6_809010	6	809010	4.45E-03	GL7_13/E1
S6_53187236	6	53187236	4.46E-03	GL14_13/E1
S6_48659560	6	48659560	4.47E-03	GL7_ac
S6_47700046	6	47700046	4.49E-03	GL14_14/E2
S6_18786387	6	18786387	4.50E-03	GL7_ac
S6_44610491	6	44610491	4.51E-03	GL21_ac
S6_51539086	6	51539086	4.52E-03	GL7_13/E1
S6_26788357	6	26788357	4.52E-03	GL14_ac
S6_26788359	6	26788359	4.52E-03	GL14_ac
S6_47626872	6	47626872	4.55E-03	GL14_ac
S6_47626871	6	47626871	4.55E-03	GL14_ac
S6_47626874	6	47626874	4.55E-03	GL14_ac
S6_13915681	6	13915681	4.56E-03	GL42_ac
S6_18806973	6	18806973	4.57E-03	GL21_ac
S6_54448658	6	54448658	4.58E-03	GL49_13/E1
S6_8919203	6	8919203	4.60E-03	GL21_13/E1
S6_1758754	6	1758754	4.62E-03	GL28_ac
S6_52092913	6	52092913	4.62E-03	GL28_13/E1
S6_46791454	6	46791454	4.64E-03	GL7_ac
S6_45184532	6	45184532	4.64E-03	GL28_14/E2
S6_34041171	6	34041171	4.66E-03	GL7_14/E2
S6_2991268	6	2991268	4.66E-03	GL21_13/E1
S6_54221535	6	54221535	4.68E-03	GL28_14/E2
S6_2683804	6	2683804	4.69E-03	GL49_ac
S6_29476511	6	29476511	4.72E-03	GL28_14/E2
S6_3915055	6	3915055	4.72E-03	GL49_ac
S6_3915058	6	3915058	4.72E-03	GL49_ac
S6_1339919	6	1339919	4.72E-03	GL7_14/E2
S6_6458325	6	6458325	4.73E-03	GL42_13/E1
S6_44610491	6	44610491	4.74E-03	GL28_ac
S6_51800735	6	51800735	4.78E-03	GL35_13/E1
S6_51800742	6	51800742	4.78E-03	GL35_13/E1
S6_51307249	6	51307249	4.78E-03	GL49_14/E2

SNP	Chromosome	Position	P.value	Trait
S6_51307294	6	51307294	4.78E-03	GL49_14/E2
S6_13915681	6	13915681	4.79E-03	GL14_ac
S6_41600509	6	41600509	4.79E-03	GL7_ac
S6_13715430	6	13715430	4.86E-03	GL21_ac
S6_19400188	6	19400188	4.86E-03	GL28_ac
S6_48659560	6	48659560	4.88E-03	GL21_13/E1
S6_50726639	6	50726639	4.89E-03	GL28_ac
S6_52092883	6	52092883	4.97E-03	GL28_13/E1
S6_52092901	6	52092901	4.97E-03	GL28_13/E1
S6_52092904	6	52092904	4.97E-03	GL28_13/E1
S6_35745026	6	35745026	4.97E-03	GL42_14/E2
S6_51800735	6	51800735	4.98E-03	GL42_13/E1
S6_51800742	6	51800742	4.98E-03	GL42_13/E1
S6_31747114	6	31747114	4.98E-03	GL21_ac
S6_17520983	6	17520983	5.01E-03	GL7_14/E2
S6_4090558	6	4090558	5.02E-03	GL28_ac
S6_47700046	6	47700046	5.03E-03	GL35_ac
S6_46734869	6	46734869	5.05E-03	GL35_ac
S6_46734881	6	46734881	5.05E-03	GL35_ac
S6_2547432	6	2547432	5.07E-03	GL7_ac
S6_54257355	6	54257355	5.11E-03	GL35_ac
S6_54257380	6	54257380	5.11E-03	GL35_ac
S6_1460005	6	1460005	5.15E-03	GL21_ac
S6_1460052	6	1460052	5.15E-03	GL21_ac
S6_1460053	6	1460053	5.15E-03	GL21_ac
S6_1309867	6	1309867	5.17E-03	GL7_ac
S6_50726639	6	50726639	5.24E-03	GL21_ac
S6_50847381	6	50847381	5.24E-03	GL7_13/E1
S6_1454193	6	1454193	5.25E-03	GL7_ac
S6_1454142	6	1454142	5.25E-03	GL7_ac
S6_43554708	6	43554708	5.32E-03	GL21_13/E1
S6_54448658	6	54448658	5.36E-03	GL7_ac
S6_18806973	6	18806973	5.39E-03	GL28_ac
S6_359049	6	359049	5.45E-03	GL7_ac
S6_359052	6	359052	5.45E-03	GL7_ac
S6_34041171	6	34041171	5.45E-03	GL28_14/E2
S6_358918	6	358918	5.45E-03	GL28_ac
S6_53187236	6	53187236	5.47E-03	GL28_13/E1
S6_48659560	6	48659560	5.48E-03	GL35_13/E1
S6_1391724	6	1391724	5.53E-03	GL28_ac
S6_58117354	6	58117354	5.59E-03	GL7_14/E2
S6_48659560	6	48659560	5.59E-03	GL7_13/E1
S6_40434431	6	40434431	5.63E-03	GL35_13/E1
S6_26282267	6	26282267	5.63E-03	GL35_14/E2
S6_2991268	6	2991268	5.66E-03	GL21_ac
S6_3525948	6	3525948	5.66E-03	GL7_ac
S6_3525949	6	3525949	5.66E-03	GL7_ac
S6_30982372	6	30982372	5.67E-03	GL42_ac
S6_47927895	6	47927895	5.69E-03	GL28_14/E2
S6_40434431	6	40434431	5.75E-03	GL42_13/E1
S6_43554708	6	43554708	5.77E-03	GL7_13/E1

SNP	Chromosome	Position	P.value	Trait
S6_31754242	6	31754242	5.77E-03	GL28_13/E1
S6_47626871	6	47626871	5.82E-03	GL14_13/E1
S6_47626872	6	47626872	5.82E-03	GL14_13/E1
S6_47626874	6	47626874	5.82E-03	GL14_13/E1
S6_1758754	6	1758754	5.89E-03	GL49_13/E1
S6_31754242	6	31754242	5.90E-03	GL35_ac
S6_52008494	6	52008494	5.93E-03	GL14_14/E2
S6_48121146	6	48121146	5.96E-03	GL28_14/E2
S6_57536070	6	57536070	5.97E-03	GL14_14/E2
S6_1587039	6	1587039	5.98E-03	GL7_ac
S6_1047672	6	1047672	5.99E-03	GL7_ac
S6_54257355	6	54257355	6.01E-03	GL21_ac
S6_54257380	6	54257380	6.01E-03	GL21_ac
S6_1339921	6	1339921	6.02E-03	GL7_14/E2
S6_72442	6	72442	6.03E-03	GL7_ac
S6_18915692	6	18915692	6.04E-03	GL35_ac
S6_36255557	6	36255557	6.05E-03	GL7_ac
S6_36255558	6	36255558	6.05E-03	GL7_ac
S6_54257355	6	54257355	6.06E-03	GL28_ac
S6_54257380	6	54257380	6.06E-03	GL28_ac
S6_25666726	6	25666726	6.09E-03	GL42_14/E2
S6_47626871	6	47626871	6.09E-03	GL21_13/E1
S6_47626872	6	47626872	6.09E-03	GL21_13/E1
S6_47626874	6	47626874	6.09E-03	GL21_13/E1
S6_7029530	6	7029530	6.10E-03	GL7_ac
S6_51307249	6	51307249	6.11E-03	GL42_14/E2
S6_51307294	6	51307294	6.11E-03	GL42_14/E2
S6_3915055	6	3915055	6.19E-03	GL49_14/E2
S6_3915058	6	3915058	6.19E-03	GL49_14/E2
S6_1839684	6	1839684	6.22E-03	GL49_ac
S6_18323513	6	18323513	6.22E-03	GL49_ac
S6_1339921	6	1339921	6.22E-03	GL7_ac
S6_31747114	6	31747114	6.24E-03	GL7_ac
S6_15331130	6	15331130	6.25E-03	GL7_13/E1
S6_15331131	6	15331131	6.25E-03	GL7_13/E1
S6_15331131	6	15331131	6.26E-03	GL7_ac
S6_15331130	6	15331130	6.26E-03	GL7_ac
S6_41266904	6	41266904	6.27E-03	GL42_ac
S6_41266913	6	41266913	6.27E-03	GL42_ac
S6_31754242	6	31754242	6.29E-03	GL42_ac
S6_52682975	6	52682975	6.39E-03	GL21_13/E1
S6_49689735	6	49689735	6.41E-03	GL14_ac
S6_52884986	6	52884986	6.45E-03	GL28_14/E2
S6_19400188	6	19400188	6.46E-03	GL14_ac
S6_2193097	6	2193097	6.49E-03	GL49_ac
S6_8919203	6	8919203	6.51E-03	GL7_ac
S6_44560725	6	44560725	6.52E-03	GL35_14/E2
S6_2683804	6	2683804	6.57E-03	GL42_ac
S6_52294116	6	52294116	6.57E-03	GL49_13/E1
S6_54448658	6	54448658	6.60E-03	GL49_ac
S6_31747106	6	31747106	6.68E-03	GL35_ac

SNP	Chromosome	Position	P.value	Trait
S6_1332901	6	1332901	6.68E-03	GL7_ac
S6_1332870	6	1332870	6.68E-03	GL7_ac
S6_1332872	6	1332872	6.68E-03	GL7_ac
S6_304009	6	304009	6.76E-03	GL28_ac
S6_52092913	6	52092913	6.78E-03	GL21_13/E1
S6_18731918	6	18731918	6.79E-03	GL42_ac
S6_15331126	6	15331126	6.82E-03	GL28_14/E2
S6_15331153	6	15331153	6.82E-03	GL28_14/E2
S6_15331164	6	15331164	6.82E-03	GL28_14/E2
S6_52850900	6	52850900	6.83E-03	GL28_13/E1
S6_2991268	6	2991268	6.83E-03	GL28_13/E1
S6_19400188	6	19400188	6.85E-03	GL21_ac
S6_36408670	6	36408670	6.86E-03	GL49_ac
S6_1306615	6	1306615	6.86E-03	GL21_ac
S6_1306617	6	1306617	6.86E-03	GL21_ac
S6_15331130	6	15331130	6.94E-03	GL14_13/E1
S6_15331131	6	15331131	6.94E-03	GL14_13/E1
S6_8919203	6	8919203	6.94E-03	GL28_13/E1
S6_51800735	6	51800735	7.00E-03	GL35_ac
S6_51800742	6	51800742	7.00E-03	GL35_ac
S6_58035596	6	58035596	7.02E-03	GL28_ac
S6_58035596	6	58035596	7.02E-03	GL21_13/E1
S6_43554708	6	43554708	7.05E-03	GL28_13/E1
S6_52092883	6	52092883	7.07E-03	GL21_13/E1
S6_52092901	6	52092901	7.07E-03	GL21_13/E1
S6_52092904	6	52092904	7.07E-03	GL21_13/E1
S6_2683804	6	2683804	7.12E-03	GL49_14/E2
S6_38461551	6	38461551	7.16E-03	GL49_ac
S6_53592874	6	53592874	7.17E-03	GL42_13/E1
S6_48659560	6	48659560	7.18E-03	GL28_13/E1
S6_52682975	6	52682975	7.18E-03	GL28_13/E1
S6_6738840	6	6738840	7.18E-03	GL42_14/E2
S6_52891701	6	52891701	7.20E-03	GL14_13/E1
S6_48682774	6	48682774	7.22E-03	GL7_ac
S6_8919203	6	8919203	7.22E-03	GL7_ac
S6_49426749	6	49426749	7.23E-03	GL28_14/E2
S6_19400188	6	19400188	7.27E-03	GL7_14/E2
S6_31747106	6	31747106	7.27E-03	GL21_13/E1
S6_52092913	6	52092913	7.29E-03	GL35_13/E1
S6_50726639	6	50726639	7.30E-03	GL35_13/E1
S6_31754242	6	31754242	7.33E-03	GL14_13/E1
S6_26383084	6	26383084	7.33E-03	GL7_ac
S6_13915681	6	13915681	7.34E-03	GL49_13/E1
S6_58035596	6	58035596	7.37E-03	GL14_13/E1
S6_3643098	6	3643098	7.40E-03	GL14_ac
S6_15331130	6	15331130	7.41E-03	GL21_13/E1
S6_15331131	6	15331131	7.41E-03	GL21_13/E1
S6_6977745	6	6977745	7.42E-03	GL49_13/E1
S6_1339919	6	1339919	7.42E-03	GL7_ac
S6_16802520	6	16802520	7.48E-03	GL7_ac
S6_54257355	6	54257355	7.48E-03	GL21_14/E2

SNP	Chromosome	Position	P.value	Trait
S6_54257380	6	54257380	7.48E-03	GL21_14/E2
S6_304009	6	304009	7.48E-03	GL21_ac
S6_53064987	6	53064987	7.52E-03	GL42_ac
S6_48651000	6	48651000	7.54E-03	GL42_ac
S6_330054	6	330054	7.54E-03	GL7_ac
S6_330085	6	330085	7.54E-03	GL7_ac
S6_39673152	6	39673152	7.55E-03	GL42_14/E2
S6_1917432	6	1917432	7.55E-03	GL35_ac
S6_13715430	6	13715430	7.56E-03	GL21_13/E1
S6_51403993	6	51403993	7.58E-03	GL49_14/E2
S6_52342910	6	52342910	7.58E-03	GL49_14/E2
S6_40219350	6	40219350	7.60E-03	GL21_ac
S6_52485896	6	52485896	7.62E-03	GL49_14/E2
S6_6738840	6	6738840	7.64E-03	GL21_ac
S6_822538	6	822538	7.65E-03	GL28_ac
S6_822527	6	822527	7.65E-03	GL28_ac
S6_1454237	6	1454237	7.66E-03	GL35_13/E1
S6_1454288	6	1454288	7.66E-03	GL35_13/E1
S6_54400448	6	54400448	7.75E-03	GL21_14/E2
S6_37639735	6	37639735	7.75E-03	GL42_14/E2
S6_38113153	6	38113153	7.77E-03	GL7_ac
S6_4090558	6	4090558	7.77E-03	GL21_ac
S6_53273004	6	53273004	7.79E-03	GL42_14/E2
S6_8919203	6	8919203	7.81E-03	GL21_ac
S6_47626872	6	47626872	7.91E-03	GL28_ac
S6_47626871	6	47626871	7.91E-03	GL28_ac
S6_47626874	6	47626874	7.91E-03	GL28_ac
S6_39672724	6	39672724	7.92E-03	GL14_ac
S6_38461551	6	38461551	7.97E-03	GL42_14/E2
S6_43554708	6	43554708	8.01E-03	GL14_13/E1
S6_51990210	6	51990210	8.04E-03	GL14_13/E1
S6_40219350	6	40219350	8.09E-03	GL28_ac
S6_49740177	6	49740177	8.09E-03	GL7_ac
S6_358918	6	358918	8.11E-03	GL21_14/E2
S6_51148591	6	51148591	8.13E-03	GL21_ac
S6_13915681	6	13915681	8.15E-03	GL21_13/E1
S6_54221535	6	54221535	8.17E-03	GL28_ac
S6_47954090	6	47954090	8.21E-03	GL7_14/E2
S6_53353798	6	53353798	8.21E-03	GL42_ac
S6_41825703	6	41825703	8.21E-03	GL7_ac
S6_53187236	6	53187236	8.26E-03	GL35_13/E1
S6_52092883	6	52092883	8.27E-03	GL35_13/E1
S6_52092901	6	52092901	8.27E-03	GL35_13/E1
S6_52092904	6	52092904	8.27E-03	GL35_13/E1
S6_1003772	6	1003772	8.28E-03	GL28_14/E2
S6_6458325	6	6458325	8.30E-03	GL7_13/E1
S6_58762171	6	58762171	8.31E-03	GL42_14/E2
S6_31747114	6	31747114	8.38E-03	GL14_ac
S6_1415785	6	1415785	8.40E-03	GL7_ac
S6_50726639	6	50726639	8.41E-03	GL42_13/E1
S6_54257355	6	54257355	8.44E-03	GL35_13/E1

SNP	Chromosome	Position	P.value	Trait
S6_54257380	6	54257380	8.44E-03	GL35_13/E1
S6_52777810	6	52777810	8.45E-03	GL49_14/E2
S6_45543005	6	45543005	8.46E-03	GL7_ac
S6_45543014	6	45543014	8.46E-03	GL7_ac
S6_31754242	6	31754242	8.52E-03	GL21_13/E1
S6_39673152	6	39673152	8.52E-03	GL42_ac
S6_14806975	6	14806975	8.55E-03	GL42_ac
S6_31747106	6	31747106	8.58E-03	GL28_ac
S6_26788357	6	26788357	8.60E-03	GL49_ac
S6_26788359	6	26788359	8.60E-03	GL49_ac
S6_17986086	6	17986086	8.66E-03	GL21_14/E2
S6_47800571	6	47800571	8.66E-03	GL49_ac
S6_47952360	6	47952360	8.71E-03	GL42_13/E1
S6_48659560	6	48659560	8.71E-03	GL14_13/E1
S6_30816124	6	30816124	8.72E-03	GL14_ac
S6_6738840	6	6738840	8.76E-03	GL7_ac
S6_38162033	6	38162033	8.94E-03	GL49_ac
S6_1374319	6	1374319	8.94E-03	GL14_ac
S6_14537959	6	14537959	9.00E-03	GL7_ac
S6_1047745	6	1047745	9.02E-03	GL35_ac
S6_13715430	6	13715430	9.03E-03	GL28_13/E1
S6_3444769	6	3444769	9.03E-03	GL7_14/E2
S6_58767277	6	58767277	9.04E-03	GL28_13/E1
S6_52777810	6	52777810	9.07E-03	GL42_14/E2
S6_1306615	6	1306615	9.09E-03	GL42_13/E1
S6_1306617	6	1306617	9.09E-03	GL42_13/E1
S6_47700046	6	47700046	9.09E-03	GL35_14/E2
S6_809010	6	809010	9.12E-03	GL28_13/E1
S6_1306615	6	1306615	9.13E-03	GL14_ac
S6_1306617	6	1306617	9.13E-03	GL14_ac
S6_51539086	6	51539086	9.14E-03	GL14_13/E1
S6_13915681	6	13915681	9.16E-03	GL7_ac
S6_20765239	6	20765239	9.18E-03	GL7_ac
S6_54448658	6	54448658	9.18E-03	GL42_ac
S6_49461190	6	49461190	9.18E-03	GL7_ac
S6_2547431	6	2547431	9.19E-03	GL14_13/E1
S6_36408670	6	36408670	9.20E-03	GL35_ac
S6_18618207	6	18618207	9.21E-03	GL42_14/E2
S6_18618211	6	18618211	9.21E-03	GL42_14/E2
S6_53187236	6	53187236	9.30E-03	GL42_13/E1
S6_1047745	6	1047745	9.30E-03	GL7_ac
S6_55225385	6	55225385	9.35E-03	GL14_13/E1
S6_6293491	6	6293491	9.35E-03	GL49_14/E2
S6_6657963	6	6657963	9.39E-03	GL49_14/E2
S6_47626871	6	47626871	9.39E-03	GL28_13/E1
S6_47626872	6	47626872	9.39E-03	GL28_13/E1
S6_47626874	6	47626874	9.39E-03	GL28_13/E1
S6_37366196	6	37366196	9.39E-03	GL7_ac
S6_1783639	6	1783639	9.40E-03	GL7_ac
S6_1783595	6	1783595	9.40E-03	GL7_ac
S6_46380487	6	46380487	9.40E-03	GL7_ac

SNP	Chromosome	Position	P.value	Trait
S6_46380491	6	46380491	9.40E-03	GL7_ac
S6_46467404	6	46467404	9.41E-03	GL49_14/E2
S6_2991268	6	2991268	9.42E-03	GL7_13/E1
S6_18806973	6	18806973	9.45E-03	GL49_ac
S6_51539086	6	51539086	9.51E-03	GL7_14/E2
S6_58035596	6	58035596	9.57E-03	GL35_13/E1
S6_29130191	6	29130191	9.57E-03	GL28_ac
S6_29130176	6	29130176	9.57E-03	GL28_ac
S6_13915681	6	13915681	9.58E-03	GL28_ac
S6_6458325	6	6458325	9.58E-03	GL49_ac
S6_44610491	6	44610491	9.59E-03	GL49_13/E1
S6_1454237	6	1454237	9.62E-03	GL28_13/E1
S6_1454288	6	1454288	9.62E-03	GL28_13/E1
S6_36255557	6	36255557	9.63E-03	GL14_ac
S6_36255558	6	36255558	9.63E-03	GL14_ac
S6_52289454	6	52289454	9.65E-03	GL14_13/E1
S6_2683804	6	2683804	9.66E-03	GL42_14/E2
S6_304009	6	304009	9.66E-03	GL42_14/E2
S6_1520148	6	1520148	9.66E-03	GL49_ac
S6_1520155	6	1520155	9.66E-03	GL49_ac
S6_1520152	6	1520152	9.66E-03	GL49_ac
S6_53353798	6	53353798	9.66E-03	GL49_14/E2
S6_52707096	6	52707096	9.73E-03	GL49_13/E1
S6_37547014	6	37547014	9.75E-03	GL21_14/E2
S6_31747106	6	31747106	9.79E-03	GL28_13/E1
S6_53353798	6	53353798	9.81E-03	GL21_13/E1
S6_2547431	6	2547431	9.83E-03	GL7_13/E1
S6_15382059	6	15382059	9.88E-03	GL42_ac
S6_8919203	6	8919203	9.89E-03	GL14_13/E1
S6_809010	6	809010	9.92E-03	GL21_ac
S6_46819386	6	46819386	9.95E-03	GL7_14/E2
S6_46819431	6	46819431	9.95E-03	GL7_14/E2
S6_51403978	6	51403978	9.96E-03	GL49_14/E2
S7_6745922	7	6745922	4.86E-06	GL7_14/E2
S7_5154297	7	5154297	2.86E-04	GL49_ac
S7_61769757	7	61769757	3.47E-04	GL35_ac
S7_6745922	7	6745922	3.78E-04	GL7_ac
S7_61384137	7	61384137	4.29E-04	GL49_13/E1
S7_5154331	7	5154331	5.82E-04	GL49_ac
S7_62431927	7	62431927	7.96E-04	GL14_ac
S7_5980283	7	5980283	8.76E-04	GL35_14/E2
S7_5416531	7	5416531	9.16E-04	GL42_ac
S7_6745922	7	6745922	9.17E-04	GL14_ac
S7_8637676	7	8637676	9.24E-04	GL49_14/E2
S7_61769757	7	61769757	1.01E-03	GL21_ac
S7_17261664	7	17261664	1.02E-03	GL49_13/E1
S7_61769757	7	61769757	1.02E-03	GL14_14/E2
S7_7029074	7	7029074	1.10E-03	GL28_13/E1
S7_5154827	7	5154827	1.13E-03	GL21_14/E2
S7_6745922	7	6745922	1.21E-03	GL21_14/E2
S7_61769757	7	61769757	1.22E-03	GL14_ac

SNP	Chromosome	Position	P.value	Trait
S7_9515682	7	9515682	1.22E-03	GL49_13/E1
S7_61769757	7	61769757	1.30E-03	GL28_ac
S7_61769757	7	61769757	1.35E-03	GL21_14/E2
S7_5963581	7	5963581	1.35E-03	GL42_ac
S7_5963604	7	5963604	1.35E-03	GL42_ac
S7_5963607	7	5963607	1.35E-03	GL42_ac
S7_5963635	7	5963635	1.35E-03	GL42_ac
S7_5963637	7	5963637	1.35E-03	GL42_ac
S7_5963638	7	5963638	1.35E-03	GL42_ac
S7_5154827	7	5154827	1.43E-03	GL28_14/E2
S7_5154297	7	5154297	1.45E-03	GL35_ac
S7_6745922	7	6745922	1.49E-03	GL28_14/E2
S7_42752212	7	42752212	1.50E-03	GL14_13/E1
S7_6745922	7	6745922	1.60E-03	GL28_ac
S7_5154297	7	5154297	1.67E-03	GL49_14/E2
S7_57211993	7	57211993	1.68E-03	GL42_13/E1
S7_5171839	7	5171839	1.70E-03	GL28_14/E2
S7_5171848	7	5171848	1.70E-03	GL28_14/E2
S7_5171847	7	5171847	1.70E-03	GL28_14/E2
S7_7190488	7	7190488	1.75E-03	GL49_ac
S7_6745922	7	6745922	1.79E-03	GL21_ac
S7_6745922	7	6745922	1.80E-03	GL7_13/E1
S7_60264001	7	60264001	1.86E-03	GL7_ac
S7_60263992	7	60263992	1.86E-03	GL7_ac
S7_60263995	7	60263995	1.86E-03	GL7_ac
S7_5154297	7	5154297	1.90E-03	GL28_14/E2
S7_7588548	7	7588548	1.93E-03	GL42_13/E1
S7_62069222	7	62069222	1.94E-03	GL7_ac
S7_5154297	7	5154297	1.97E-03	GL42_ac
S7_5154331	7	5154331	1.98E-03	GL49_14/E2
S7_7029074	7	7029074	2.00E-03	GL35_13/E1
S7_42752212	7	42752212	2.06E-03	GL21_13/E1
S7_4559643	7	4559643	2.11E-03	GL49_14/E2
S7_7029074	7	7029074	2.12E-03	GL42_13/E1
S7_56425997	7	56425997	2.25E-03	GL28_13/E1
S7_61149944	7	61149944	2.28E-03	GL7_ac
S7_61149948	7	61149948	2.28E-03	GL7_ac
S7_61149950	7	61149950	2.28E-03	GL7_ac
S7_61149952	7	61149952	2.28E-03	GL7_ac
S7_61149953	7	61149953	2.28E-03	GL7_ac
S7_61149960	7	61149960	2.28E-03	GL7_ac
S7_61769757	7	61769757	2.31E-03	GL42_ac
S7_7029074	7	7029074	2.39E-03	GL21_13/E1
S7_9515682	7	9515682	2.41E-03	GL35_13/E1
S7_4526800	7	4526800	2.44E-03	GL49_14/E2
S7_4526808	7	4526808	2.44E-03	GL49_14/E2
S7_5154297	7	5154297	2.50E-03	GL28_ac
S7_6745922	7	6745922	2.53E-03	GL14_13/E1
S7_57211993	7	57211993	2.56E-03	GL35_13/E1
S7_64079510	7	64079510	2.57E-03	GL49_13/E1
S7_58845835	7	58845835	2.58E-03	GL28_14/E2

SNP	Chromosome	Position	P.value	Trait
S7_57211993	7	57211993	2.59E-03	GL28_13/E1
S7_5416531	7	5416531	2.64E-03	GL49_ac
S7_4785414	7	4785414	2.65E-03	GL21_14/E2
S7_4785415	7	4785415	2.65E-03	GL21_14/E2
S7_4438987	7	4438987	2.69E-03	GL49_14/E2
S7_5154297	7	5154297	2.72E-03	GL7_14/E2
S7_9515682	7	9515682	2.73E-03	GL42_13/E1
S7_6974581	7	6974581	2.74E-03	GL21_14/E2
S7_5383474	7	5383474	2.78E-03	GL49_13/E1
S7_5154331	7	5154331	2.81E-03	GL7_14/E2
S7_7588548	7	7588548	2.84E-03	GL35_13/E1
S7_61769757	7	61769757	2.87E-03	GL49_ac
S7_7588548	7	7588548	2.87E-03	GL28_13/E1
S7_63084000	7	63084000	2.95E-03	GL28_14/E2
S7_63084011	7	63084011	2.95E-03	GL28_14/E2
S7_57848081	7	57848081	3.02E-03	GL49_13/E1
S7_5417560	7	5417560	3.03E-03	GL21_14/E2
S7_58845835	7	58845835	3.10E-03	GL21_14/E2
S7_5154331	7	5154331	3.29E-03	GL35_ac
S7_4559643	7	4559643	3.34E-03	GL49_ac
S7_5154331	7	5154331	3.39E-03	GL21_13/E1
S7_56425997	7	56425997	3.43E-03	GL14_13/E1
S7_61384137	7	61384137	3.45E-03	GL42_13/E1
S7_56425997	7	56425997	3.51E-03	GL21_13/E1
S7_59417478	7	59417478	3.52E-03	GL49_14/E2
S7_59417500	7	59417500	3.52E-03	GL49_14/E2
S7_59417502	7	59417502	3.52E-03	GL49_14/E2
S7_5154297	7	5154297	3.52E-03	GL21_13/E1
S7_58845835	7	58845835	3.54E-03	GL14_ac
S7_60263992	7	60263992	3.55E-03	GL14_13/E1
S7_60263995	7	60263995	3.55E-03	GL14_13/E1
S7_60264001	7	60264001	3.55E-03	GL14_13/E1
S7_9515682	7	9515682	3.58E-03	GL28_13/E1
S7_62431927	7	62431927	3.59E-03	GL21_ac
S7_60698183	7	60698183	3.63E-03	GL7_ac
S7_61769757	7	61769757	3.65E-03	GL28_14/E2
S7_11042618	7	11042618	3.67E-03	GL21_14/E2
S7_12260910	7	12260910	3.76E-03	GL7_ac
S7_61769757	7	61769757	3.81E-03	GL7_ac
S7_6745922	7	6745922	3.82E-03	GL21_13/E1
S7_2083276	7	2083276	3.86E-03	GL7_14/E2
S7_64079510	7	64079510	3.91E-03	GL7_13/E1
S7_42752212	7	42752212	4.11E-03	GL7_13/E1
S7_57819725	7	57819725	4.12E-03	GL49_13/E1
S7_17261664	7	17261664	4.40E-03	GL35_13/E1
S7_5154331	7	5154331	4.49E-03	GL42_ac
S7_1522170	7	1522170	4.50E-03	GL7_13/E1
S7_1522174	7	1522174	4.50E-03	GL7_13/E1
S7_1522177	7	1522177	4.50E-03	GL7_13/E1
S7_1522180	7	1522180	4.50E-03	GL7_13/E1
S7_1522182	7	1522182	4.50E-03	GL7_13/E1

SNP	Chromosome	Position	P.value	Trait
S7_1522183	7	1522183	4.50E-03	GL7_13/E1
S7_6745922	7	6745922	4.51E-03	GL35_ac
S7_2112786	7	2112786	4.53E-03	GL7_14/E2
S7_7872315	7	7872315	4.53E-03	GL14_13/E1
S7_538528	7	538528	4.55E-03	GL14_14/E2
S7_151554	7	151554	4.55E-03	GL49_14/E2
S7_8637676	7	8637676	4.57E-03	GL42_14/E2
S7_12790226	7	12790226	4.59E-03	GL14_13/E1
S7_2112747	7	2112747	4.60E-03	GL49_14/E2
S7_60709314	7	60709314	4.82E-03	GL7_ac
S7_17261664	7	17261664	4.85E-03	GL28_13/E1
S7_2112747	7	2112747	4.92E-03	GL7_14/E2
S7_6974678	7	6974678	4.93E-03	GL49_ac
S7_6975329	7	6975329	4.94E-03	GL49_13/E1
S7_7619279	7	7619279	5.03E-03	GL21_14/E2
S7_12071810	7	12071810	5.04E-03	GL49_14/E2
S7_7588548	7	7588548	5.09E-03	GL42_ac
S7_5207452	7	5207452	5.10E-03	GL49_ac
S7_6976847	7	6976847	5.10E-03	GL7_ac
S7_5154331	7	5154331	5.13E-03	GL28_ac
S7_64024491	7	64024491	5.13E-03	GL49_14/E2
S7_64079510	7	64079510	5.20E-03	GL28_13/E1
S7_58845835	7	58845835	5.22E-03	GL35_ac
S7_4438946	7	4438946	5.26E-03	GL49_14/E2
S7_4438947	7	4438947	5.26E-03	GL49_14/E2
S7_42752212	7	42752212	5.35E-03	GL14_ac
S7_4438987	7	4438987	5.43E-03	GL49_ac
S7_5416531	7	5416531	5.45E-03	GL28_14/E2
S7_60709314	7	60709314	5.48E-03	GL14_13/E1
S7_807355	7	807355	5.51E-03	GL42_ac
S7_5416531	7	5416531	5.51E-03	GL35_ac
S7_8909175	7	8909175	5.53E-03	GL28_13/E1
S7_151554	7	151554	5.54E-03	GL42_14/E2
S7_64742874	7	64742874	5.54E-03	GL7_14/E2
S7_59404484	7	59404484	5.56E-03	GL42_14/E2
S7_56160907	7	56160907	5.56E-03	GL35_13/E1
S7_56160908	7	56160908	5.56E-03	GL35_13/E1
S7_56160938	7	56160938	5.56E-03	GL35_13/E1
S7_3623922	7	3623922	5.59E-03	GL7_14/E2
S7_3623940	7	3623940	5.59E-03	GL7_14/E2
S7_57372955	7	57372955	5.66E-03	GL28_13/E1
S7_151554	7	151554	5.72E-03	GL42_ac
S7_59389616	7	59389616	5.77E-03	GL49_13/E1
S7_5416531	7	5416531	5.78E-03	GL21_14/E2
S7_62069222	7	62069222	5.80E-03	GL14_13/E1
S7_6974581	7	6974581	5.82E-03	GL28_ac
S7_57892844	7	57892844	5.85E-03	GL42_13/E1
S7_57892845	7	57892845	5.85E-03	GL42_13/E1
S7_57892847	7	57892847	5.85E-03	GL42_13/E1
S7_62467421	7	62467421	5.88E-03	GL7_13/E1
S7_56425997	7	56425997	5.91E-03	GL35_13/E1

SNP	Chromosome	Position	P.value	Trait
S7_2083276	7	2083276	5.99E-03	GL28_14/E2
S7_60263992	7	60263992	6.00E-03	GL7_13/E1
S7_60263995	7	60263995	6.00E-03	GL7_13/E1
S7_60264001	7	60264001	6.00E-03	GL7_13/E1
S7_5154331	7	5154331	6.10E-03	GL28_14/E2
S7_17261664	7	17261664	6.14E-03	GL42_13/E1
S7_4438946	7	4438946	6.23E-03	GL49_ac
S7_4438947	7	4438947	6.23E-03	GL49_ac
S7_60407976	7	60407976	6.31E-03	GL49_13/E1
S7_6974678	7	6974678	6.31E-03	GL35_ac
S7_59830113	7	59830113	6.32E-03	GL49_13/E1
S7_38780634	7	38780634	6.36E-03	GL14_13/E1
S7_38780634	7	38780634	6.36E-03	GL49_13/E1
S7_65242089	7	65242089	6.37E-03	GL14_14/E2
S7_64079510	7	64079510	6.46E-03	GL14_13/E1
S7_410940	7	410940	6.50E-03	GL7_ac
S7_930915	7	930915	6.55E-03	GL14_14/E2
S7_6745922	7	6745922	6.57E-03	GL14_14/E2
S7_6976847	7	6976847	6.58E-03	GL7_14/E2
S7_5171839	7	5171839	6.58E-03	GL7_14/E2
S7_5171847	7	5171847	6.58E-03	GL7_14/E2
S7_5171848	7	5171848	6.58E-03	GL7_14/E2
S7_5207452	7	5207452	6.64E-03	GL21_14/E2
S7_1522170	7	1522170	6.69E-03	GL21_13/E1
S7_1522174	7	1522174	6.69E-03	GL21_13/E1
S7_1522177	7	1522177	6.69E-03	GL21_13/E1
S7_1522180	7	1522180	6.69E-03	GL21_13/E1
S7_1522182	7	1522182	6.69E-03	GL21_13/E1
S7_1522183	7	1522183	6.69E-03	GL21_13/E1
S7_4559643	7	4559643	6.71E-03	GL42_ac
S7_6974581	7	6974581	6.72E-03	GL35_ac
S7_410878	7	410878	6.73E-03	GL7_ac
S7_56054547	7	56054547	6.74E-03	GL28_14/E2
S7_4438987	7	4438987	6.75E-03	GL42_ac
S7_5383474	7	5383474	6.80E-03	GL49_ac
S7_62118605	7	62118605	6.80E-03	GL14_14/E2
S7_62431927	7	62431927	6.81E-03	GL35_ac
S7_1639886	7	1639886	6.88E-03	GL7_13/E1
S7_1639911	7	1639911	6.88E-03	GL7_13/E1
S7_62390907	7	62390907	6.90E-03	GL35_14/E2
S7_4785414	7	4785414	6.95E-03	GL49_ac
S7_4785415	7	4785415	6.95E-03	GL49_ac
S7_61414539	7	61414539	6.96E-03	GL7_ac
S7_6974581	7	6974581	7.00E-03	GL21_13/E1
S7_65085167	7	65085167	7.04E-03	GL42_13/E1
S7_59307230	7	59307230	7.05E-03	GL21_14/E2
S7_6761736	7	6761736	7.08E-03	GL7_13/E1
S7_42752212	7	42752212	7.08E-03	GL28_13/E1
S7_6745922	7	6745922	7.12E-03	GL35_14/E2
S7_5301931	7	5301931	7.15E-03	GL49_ac
S7_5154331	7	5154331	7.17E-03	GL28_13/E1

SNP	Chromosome	Position	P.value	Trait
S7_5572054	7	5572054	7.17E-03	GL28_13/E1
S7_5154297	7	5154297	7.18E-03	GL28_13/E1
S7_7872315	7	7872315	7.27E-03	GL21_13/E1
S7_5207452	7	5207452	7.30E-03	GL28_14/E2
S7_63134774	7	63134774	7.35E-03	GL42_ac
S7_42752212	7	42752212	7.37E-03	GL49_14/E2
S7_538372	7	538372	7.41E-03	GL28_14/E2
S7_5872814	7	5872814	7.54E-03	GL35_13/E1
S7_8909175	7	8909175	7.56E-03	GL42_13/E1
S7_5154827	7	5154827	7.59E-03	GL35_ac
S7_14482605	7	14482605	7.59E-03	GL14_ac
S7_7190488	7	7190488	7.63E-03	GL42_ac
S7_63563543	7	63563543	7.64E-03	GL42_13/E1
S7_62869363	7	62869363	7.64E-03	GL35_13/E1
S7_8105662	7	8105662	7.69E-03	GL28_14/E2
S7_62431927	7	62431927	7.87E-03	GL28_ac
S7_5171839	7	5171839	7.97E-03	GL42_ac
S7_5171848	7	5171848	7.97E-03	GL42_ac
S7_5171847	7	5171847	7.97E-03	GL42_ac
S7_5963581	7	5963581	7.97E-03	GL42_13/E1
S7_5963604	7	5963604	7.97E-03	GL42_13/E1
S7_5963607	7	5963607	7.97E-03	GL42_13/E1
S7_5963635	7	5963635	7.97E-03	GL42_13/E1
S7_5963637	7	5963637	7.97E-03	GL42_13/E1
S7_5963638	7	5963638	7.97E-03	GL42_13/E1
S7_53872401	7	53872401	8.02E-03	GL7_ac
S7_61414567	7	61414567	8.05E-03	GL7_ac
S7_55979632	7	55979632	8.09E-03	GL21_13/E1
S7_4785414	7	4785414	8.15E-03	GL42_ac
S7_4785415	7	4785415	8.15E-03	GL42_ac
S7_4438987	7	4438987	8.18E-03	GL21_14/E2
S7_58845835	7	58845835	8.22E-03	GL7_ac
S7_7029074	7	7029074	8.32E-03	GL49_13/E1
S7_7190488	7	7190488	8.33E-03	GL28_14/E2
S7_5417560	7	5417560	8.33E-03	GL42_ac
S7_42752212	7	42752212	8.35E-03	GL49_ac
S7_63623326	7	63623326	8.37E-03	GL49_13/E1
S7_63623330	7	63623330	8.37E-03	GL49_13/E1
S7_5207452	7	5207452	8.41E-03	GL35_ac
S7_8994958	7	8994958	8.47E-03	GL21_14/E2
S7_64024491	7	64024491	8.49E-03	GL42_14/E2
S7_5154297	7	5154297	8.52E-03	GL35_13/E1
S7_7029074	7	7029074	8.53E-03	GL28_ac
S7_7619279	7	7619279	8.59E-03	GL14_14/E2
S7_64079510	7	64079510	8.60E-03	GL35_13/E1
S7_58668167	7	58668167	8.64E-03	GL35_13/E1
S7_61149944	7	61149944	8.73E-03	GL7_13/E1
S7_61149948	7	61149948	8.73E-03	GL7_13/E1
S7_61149950	7	61149950	8.73E-03	GL7_13/E1
S7_61149952	7	61149952	8.73E-03	GL7_13/E1
S7_61149953	7	61149953	8.73E-03	GL7_13/E1

SNP	Chromosome	Position	P.value	Trait
S7_61149960	7	61149960	8.73E-03	GL7_13/E1
S7_60260045	7	60260045	8.78E-03	GL21_14/E2
S7_60260059	7	60260059	8.78E-03	GL21_14/E2
S7_29646785	7	29646785	8.80E-03	GL42_14/E2
S7_2083276	7	2083276	8.83E-03	GL49_14/E2
S7_5207452	7	5207452	8.89E-03	GL42_ac
S7_8397336	7	8397336	8.92E-03	GL35_14/E2
S7_8397340	7	8397340	8.92E-03	GL35_14/E2
S7_8397385	7	8397385	8.92E-03	GL35_14/E2
S7_8397393	7	8397393	8.92E-03	GL35_14/E2
S7_8397394	7	8397394	8.92E-03	GL35_14/E2
S7_5980283	7	5980283	8.98E-03	GL14_14/E2
S7_5183992	7	5183992	8.99E-03	GL21_14/E2
S7_570062	7	570062	9.00E-03	GL14_14/E2
S7_5154827	7	5154827	9.00E-03	GL42_ac
S7_58845835	7	58845835	9.02E-03	GL28_ac
S7_62467421	7	62467421	9.02E-03	GL14_13/E1
S7_7588548	7	7588548	9.02E-03	GL49_13/E1
S7_5417560	7	5417560	9.02E-03	GL28_14/E2
S7_60468035	7	60468035	9.04E-03	GL14_ac
S7_592795	7	592795	9.07E-03	GL7_13/E1
S7_59417478	7	59417478	9.09E-03	GL35_14/E2
S7_59417500	7	59417500	9.09E-03	GL35_14/E2
S7_59417502	7	59417502	9.09E-03	GL35_14/E2
S7_7588548	7	7588548	9.10E-03	GL21_13/E1
S7_61271950	7	61271950	9.13E-03	GL7_ac
S7_8905054	7	8905054	9.14E-03	GL42_14/E2
S7_8905055	7	8905055	9.14E-03	GL42_14/E2
S7_8905066	7	8905066	9.14E-03	GL42_14/E2
S7_4526800	7	4526800	9.17E-03	GL49_ac
S7_4526808	7	4526808	9.17E-03	GL49_ac
S7_5154827	7	5154827	9.17E-03	GL14_14/E2
S7_64079510	7	64079510	9.18E-03	GL14_ac
S7_6974581	7	6974581	9.19E-03	GL35_13/E1
S7_5154297	7	5154297	9.20E-03	GL21_ac
S7_6761736	7	6761736	9.21E-03	GL14_13/E1
S7_60468035	7	60468035	9.27E-03	GL49_13/E1
S7_54789016	7	54789016	9.27E-03	GL7_14/E2
S7_65081304	7	65081304	9.30E-03	GL42_13/E1
S7_61414557	7	61414557	9.33E-03	GL7_ac
S7_6974581	7	6974581	9.34E-03	GL21_ac
S7_63067948	7	63067948	9.42E-03	GL35_14/E2
S7_5383474	7	5383474	9.44E-03	GL7_14/E2
S7_62467421	7	62467421	9.45E-03	GL21_13/E1
S7_57803343	7	57803343	9.55E-03	GL49_13/E1
S7_8909175	7	8909175	9.56E-03	GL35_13/E1
S7_63563527	7	63563527	9.57E-03	GL42_13/E1
S7_6974678	7	6974678	9.58E-03	GL21_14/E2
S7_6974806	7	6974806	9.62E-03	GL49_13/E1
S7_1356501	7	1356501	9.63E-03	GL49_ac
S7_1356530	7	1356530	9.63E-03	GL49_ac

SNP	Chromosome	Position	P.value	Trait
S7_60709314	7	60709314	9.71E-03	GL7_13/E1
S7_57745824	7	57745824	9.71E-03	GL28_13/E1
S7_57745836	7	57745836	9.71E-03	GL28_13/E1
S7_2939252	7	2939252	9.76E-03	GL21_14/E2
S7_61149944	7	61149944	9.76E-03	GL28_ac
S7_61149948	7	61149948	9.76E-03	GL28_ac
S7_61149950	7	61149950	9.76E-03	GL28_ac
S7_61149952	7	61149952	9.76E-03	GL28_ac
S7_61149953	7	61149953	9.76E-03	GL28_ac
S7_61149960	7	61149960	9.76E-03	GL28_ac
S7_57417001	7	57417001	9.86E-03	GL14_14/E2
S7_57417016	7	57417016	9.86E-03	GL14_14/E2
S7_5572054	7	5572054	9.91E-03	GL35_13/E1
S7_3391760	7	3391760	9.97E-03	GL28_13/E1
S7_61414539	7	61414539	9.98E-03	GL21_ac
S8_1325687	8	1325687	2.75E-04	GL49_14/E2
S8_1325690	8	1325690	2.75E-04	GL49_14/E2
S8_1325745	8	1325745	2.75E-04	GL49_14/E2
S8_43465153	8	43465153	3.70E-04	GL49_14/E2
S8_61581004	8	61581004	4.04E-04	GL7_13/E1
S8_4481064	8	4481064	1.01E-03	GL35_ac
S8_4481064	8	4481064	1.05E-03	GL35_14/E2
S8_5441996	8	5441996	1.11E-03	GL7_13/E1
S8_1993448	8	1993448	1.15E-03	GL49_14/E2
S8_61581004	8	61581004	1.43E-03	GL14_13/E1
S8_60246781	8	60246781	1.64E-03	GL7_14/E2
S8_44669846	8	44669846	1.69E-03	GL35_14/E2
S8_33210795	8	33210795	1.76E-03	GL21_14/E2
S8_48988557	8	48988557	1.81E-03	GL7_14/E2
S8_5441996	8	5441996	1.83E-03	GL14_13/E1
S8_2374641	8	2374641	1.84E-03	GL49_14/E2
S8_33210795	8	33210795	1.91E-03	GL14_14/E2
S8_61581004	8	61581004	2.09E-03	GL7_ac
S8_61590665	8	61590665	2.18E-03	GL28_14/E2
S8_1558734	8	1558734	2.21E-03	GL49_14/E2
S8_1558740	8	1558740	2.21E-03	GL49_14/E2
S8_60349308	8	60349308	2.26E-03	GL49_14/E2
S8_1325687	8	1325687	2.63E-03	GL42_14/E2
S8_1325690	8	1325690	2.63E-03	GL42_14/E2
S8_1325745	8	1325745	2.63E-03	GL42_14/E2
S8_61581004	8	61581004	2.65E-03	GL28_13/E1
S8_504185	8	504185	2.67E-03	GL35_14/E2
S8_504221	8	504221	2.67E-03	GL35_14/E2
S8_60943170	8	60943170	2.78E-03	GL42_13/E1
S8_60943171	8	60943171	2.78E-03	GL42_13/E1
S8_60943177	8	60943177	2.78E-03	GL42_13/E1
S8_339001	8	339001	2.79E-03	GL28_14/E2
S8_61581004	8	61581004	2.81E-03	GL21_ac
S8_1197740	8	1197740	2.84E-03	GL42_14/E2
S8_15299963	8	15299963	2.89E-03	GL42_13/E1
S8_15299927	8	15299927	2.89E-03	GL42_13/E1

SNP	Chromosome	Position	P.value	Trait
S8_4289984	8	4289984	2.95E-03	GL42_ac
S8_2063869	8	2063869	3.00E-03	GL49_14/E2
S8_60349308	8	60349308	3.11E-03	GL49_ac
S8_4481064	8	4481064	3.21E-03	GL42_ac
S8_1066065	8	1066065	3.25E-03	GL42_14/E2
S8_1066073	8	1066073	3.25E-03	GL42_14/E2
S8_1066079	8	1066079	3.25E-03	GL42_14/E2
S8_1066080	8	1066080	3.25E-03	GL42_14/E2
S8_1066081	8	1066081	3.25E-03	GL42_14/E2
S8_1066082	8	1066082	3.25E-03	GL42_14/E2
S8_1066083	8	1066083	3.25E-03	GL42_14/E2
S8_61581004	8	61581004	3.31E-03	GL14_ac
S8_51575493	8	51575493	3.31E-03	GL21_14/E2
S8_51575507	8	51575507	3.31E-03	GL21_14/E2
S8_60349308	8	60349308	3.43E-03	GL7_14/E2
S8_60140244	8	60140244	3.43E-03	GL21_ac
S8_1642168	8	1642168	3.47E-03	GL49_14/E2
S8_1642171	8	1642171	3.47E-03	GL49_14/E2
S8_15337904	8	15337904	3.49E-03	GL42_ac
S8_53829072	8	53829072	3.70E-03	GL49_14/E2
S8_53829081	8	53829081	3.70E-03	GL49_14/E2
S8_61581004	8	61581004	3.75E-03	GL21_13/E1
S8_53594816	8	53594816	3.76E-03	GL42_13/E1
S8_2063869	8	2063869	3.84E-03	GL42_14/E2
S8_51315455	8	51315455	3.89E-03	GL21_14/E2
S8_50475201	8	50475201	3.91E-03	GL42_ac
S8_504185	8	504185	3.93E-03	GL28_14/E2
S8_504221	8	504221	3.93E-03	GL28_14/E2
S8_60349308	8	60349308	3.93E-03	GL14_14/E2
S8_51922096	8	51922096	3.96E-03	GL42_13/E1
S8_6158575	8	6158575	4.10E-03	GL49_13/E1
S8_6158577	8	6158577	4.10E-03	GL49_13/E1
S8_16865003	8	16865003	4.15E-03	GL21_14/E2
S8_53829072	8	53829072	4.21E-03	GL35_14/E2
S8_53829081	8	53829081	4.21E-03	GL35_14/E2
S8_53594816	8	53594816	4.26E-03	GL28_13/E1
S8_32755312	8	32755312	4.26E-03	GL28_14/E2
S8_51294410	8	51294410	4.35E-03	GL42_13/E1
S8_1252165	8	1252165	4.43E-03	GL49_14/E2
S8_32829500	8	32829500	4.44E-03	GL35_14/E2
S8_53508627	8	53508627	4.46E-03	GL35_13/E1
S8_53508627	8	53508627	4.49E-03	GL28_13/E1
S8_5441996	8	5441996	4.61E-03	GL14_ac
S8_5379958	8	5379958	4.62E-03	GL7_13/E1
S8_1839524	8	1839524	4.68E-03	GL42_14/E2
S8_1839524	8	1839524	4.68E-03	GL42_14/E2
S8_55846613	8	55846613	4.76E-03	GL42_13/E1
S8_1123093	8	1123093	5.03E-03	GL42_14/E2
S8_5441996	8	5441996	5.07E-03	GL21_13/E1
S8_37668684	8	37668684	5.09E-03	GL35_ac
S8_60349308	8	60349308	5.18E-03	GL42_14/E2

SNP	Chromosome	Position	P.value	Trait
S8_1216720	8	1216720	5.21E-03	GL28_14/E2
S8_2615029	8	2615029	5.22E-03	GL49_14/E2
S8_2615030	8	2615030	5.22E-03	GL49_14/E2
S8_2615032	8	2615032	5.22E-03	GL49_14/E2
S8_2615037	8	2615037	5.22E-03	GL49_14/E2
S8_61612185	8	61612185	5.24E-03	GL7_14/E2
S8_49087038	8	49087038	5.28E-03	GL49_13/E1
S8_60943169	8	60943169	5.32E-03	GL42_13/E1
S8_61590665	8	61590665	5.35E-03	GL21_14/E2
S8_1699232	8	1699232	5.39E-03	GL35_14/E2
S8_1577433	8	1577433	5.44E-03	GL35_14/E2
S8_59748647	8	59748647	5.44E-03	GL35_ac
S8_59748618	8	59748618	5.44E-03	GL35_ac
S8_1642168	8	1642168	5.60E-03	GL42_14/E2
S8_1642171	8	1642171	5.60E-03	GL42_14/E2
S8_1501432	8	1501432	5.69E-03	GL49_14/E2
S8_5441996	8	5441996	5.71E-03	GL28_13/E1
S8_2063869	8	2063869	5.75E-03	GL35_14/E2
S8_1577433	8	1577433	5.95E-03	GL14_14/E2
S8_37668684	8	37668684	6.01E-03	GL42_14/E2
S8_49194390	8	49194390	6.03E-03	GL49_ac
S8_60349308	8	60349308	6.15E-03	GL21_14/E2
S8_2051830	8	2051830	6.16E-03	GL35_14/E2
S8_9637993	8	9637993	6.22E-03	GL28_14/E2
S8_44669846	8	44669846	6.23E-03	GL14_14/E2
S8_1197740	8	1197740	6.26E-03	GL42_ac
S8_61581004	8	61581004	6.28E-03	GL49_13/E1
S8_53508627	8	53508627	6.30E-03	GL42_13/E1
S8_5441996	8	5441996	6.38E-03	GL49_13/E1
S8_43465153	8	43465153	6.48E-03	GL42_14/E2
S8_15299963	8	15299963	6.57E-03	GL49_13/E1
S8_15299927	8	15299927	6.57E-03	GL49_13/E1
S8_37668684	8	37668684	6.58E-03	GL42_ac
S8_46347251	8	46347251	6.60E-03	GL49_14/E2
S8_61581004	8	61581004	6.61E-03	GL28_ac
S8_56113697	8	56113697	6.64E-03	GL28_14/E2
S8_56113718	8	56113718	6.64E-03	GL28_14/E2
S8_56113728	8	56113728	6.64E-03	GL28_14/E2
S8_1252126	8	1252126	6.64E-03	GL21_14/E2
S8_5477029	8	5477029	6.71E-03	GL21_14/E2
S8_5477050	8	5477050	6.71E-03	GL21_14/E2
S8_5477049	8	5477049	6.71E-03	GL21_14/E2
S8_51922096	8	51922096	6.73E-03	GL28_13/E1
S8_60944459	8	60944459	6.97E-03	GL28_13/E1
S8_60944465	8	60944465	6.97E-03	GL28_13/E1
S8_60944466	8	60944466	6.97E-03	GL28_13/E1
S8_60140244	8	60140244	6.98E-03	GL14_ac
S8_33210795	8	33210795	7.02E-03	GL35_14/E2
S8_60349308	8	60349308	7.07E-03	GL42_ac
S8_40730391	8	40730391	7.13E-03	GL49_13/E1
S8_40730445	8	40730445	7.13E-03	GL49_13/E1

SNP	Chromosome	Position	P.value	Trait
S8_51315455	8	51315455	7.21E-03	GL42_ac
S8_45793219	8	45793219	7.30E-03	GL21_14/E2
S8_55846613	8	55846613	7.30E-03	GL49_13/E1
S8_16865003	8	16865003	7.31E-03	GL14_14/E2
S8_50617053	8	50617053	7.38E-03	GL7_ac
S8_56539807	8	56539807	7.40E-03	GL49_ac
S8_42774320	8	42774320	7.46E-03	GL14_ac
S8_504185	8	504185	7.47E-03	GL28_ac
S8_504221	8	504221	7.47E-03	GL28_ac
S8_4209912	8	4209912	7.50E-03	GL28_ac
S8_6185103	8	6185103	7.52E-03	GL42_ac
S8_513629	8	513629	7.54E-03	GL28_ac
S8_1839095	8	1839095	7.59E-03	GL49_14/E2
S8_5379958	8	5379958	7.75E-03	GL35_13/E1
S8_16196648	8	16196648	7.82E-03	GL7_14/E2
S8_15337904	8	15337904	7.85E-03	GL28_ac
S8_49087038	8	49087038	7.92E-03	GL14_14/E2
S8_50475201	8	50475201	7.95E-03	GL35_ac
S8_1123093	8	1123093	7.97E-03	GL49_14/E2
S8_16865003	8	16865003	8.12E-03	GL28_14/E2
S8_1993659	8	1993659	8.12E-03	GL35_13/E1
S8_53508627	8	53508627	8.14E-03	GL21_13/E1
S8_46362742	8	46362742	8.21E-03	GL14_14/E2
S8_51762013	8	51762013	8.24E-03	GL42_ac
S8_51761985	8	51761985	8.24E-03	GL42_ac
S8_1991830	8	1991830	8.32E-03	GL14_14/E2
S8_1642168	8	1642168	8.37E-03	GL35_14/E2
S8_1642171	8	1642171	8.37E-03	GL35_14/E2
S8_4209912	8	4209912	8.40E-03	GL21_13/E1
S8_15337904	8	15337904	8.45E-03	GL14_14/E2
S8_1172689	8	1172689	8.50E-03	GL35_14/E2
S8_1172690	8	1172690	8.50E-03	GL35_14/E2
S8_1577433	8	1577433	8.55E-03	GL21_14/E2
S8_1356088	8	1356088	8.65E-03	GL28_14/E2
S8_38329974	8	38329974	8.68E-03	GL35_ac
S8_1699232	8	1699232	8.69E-03	GL42_14/E2
S8_15337904	8	15337904	8.74E-03	GL21_ac
S8_51922096	8	51922096	8.80E-03	GL35_13/E1
S8_1515469	8	1515469	8.94E-03	GL35_14/E2
S8_3510658	8	3510658	8.97E-03	GL42_13/E1
S8_40730391	8	40730391	9.08E-03	GL21_13/E1
S8_40730445	8	40730445	9.08E-03	GL21_13/E1
S8_1501432	8	1501432	9.16E-03	GL35_ac
S8_38329974	8	38329974	9.18E-03	GL35_14/E2
S8_15337904	8	15337904	9.18E-03	GL7_ac
S8_49770634	8	49770634	9.20E-03	GL7_14/E2
S8_49770652	8	49770652	9.20E-03	GL7_14/E2
S8_1839095	8	1839095	9.20E-03	GL35_14/E2
S8_46320095	8	46320095	9.22E-03	GL7_14/E2
S8_15337904	8	15337904	9.29E-03	GL14_ac
S8_53860662	8	53860662	9.29E-03	GL49_14/E2

SNP	Chromosome	Position	P.value	Trait
S8_4209912	8	4209912	9.35E-03	GL21_ac
S8_45095054	8	45095054	9.36E-03	GL7_14/E2
S8_61612185	8	61612185	9.41E-03	GL7_ac
S8_40730391	8	40730391	9.49E-03	GL21_ac
S8_40730445	8	40730445	9.49E-03	GL21_ac
S8_50617053	8	50617053	9.53E-03	GL14_14/E2
S8_62009189	8	62009189	9.54E-03	GL7_14/E2
S8_60246781	8	60246781	9.72E-03	GL7_ac
S8_51761985	8	51761985	9.74E-03	GL42_13/E1
S8_51762013	8	51762013	9.74E-03	GL42_13/E1
S8_4209912	8	4209912	9.75E-03	GL35_13/E1
S8_60944459	8	60944459	9.82E-03	GL21_13/E1
S8_60944465	8	60944465	9.82E-03	GL21_13/E1
S8_60944466	8	60944466	9.82E-03	GL21_13/E1
S8_45862085	8	45862085	9.97E-03	GL42_14/E2
S8_53626886	8	53626886	9.97E-03	GL21_14/E2
S8_53626899	8	53626899	9.97E-03	GL21_14/E2
S9_58295230	9	58295230	5.45E-06	GL14_ac
S9_58757394	9	58757394	9.54E-06	GL42_ac
S9_58757394	9	58757394	2.82E-05	GL35_ac
S9_58295230	9	58295230	3.38E-05	GL14_14/E2
S9_58295230	9	58295230	5.38E-05	GL7_13/E1
S9_58295230	9	58295230	9.75E-05	GL7_ac
S9_57545053	9	57545053	9.92E-05	GL14_14/E2
S9_58757394	9	58757394	1.10E-04	GL21_14/E2
S9_57947937	9	57947937	1.14E-04	GL7_ac
S9_58757394	9	58757394	1.37E-04	GL28_ac
S9_57947937	9	57947937	1.38E-04	GL21_13/E1
S9_58757394	9	58757394	1.39E-04	GL14_14/E2
S9_57875319	9	57875319	1.44E-04	GL28_13/E1
S9_57947937	9	57947937	1.46E-04	GL28_13/E1
S9_58757394	9	58757394	1.50E-04	GL28_14/E2
S9_58295230	9	58295230	1.53E-04	GL14_13/E1
S9_58504227	9	58504227	1.75E-04	GL21_14/E2
S9_58684354	9	58684354	2.01E-04	GL49_13/E1
S9_58504227	9	58504227	2.02E-04	GL28_ac
S9_58504227	9	58504227	2.16E-04	GL21_ac
S9_3933616	9	3933616	2.34E-04	GL35_ac
S9_58295230	9	58295230	2.41E-04	GL21_ac
S9_58504227	9	58504227	2.56E-04	GL14_ac
S9_58504227	9	58504227	2.78E-04	GL28_14/E2
S9_2233856	9	2233856	2.85E-04	GL42_ac
S9_58757394	9	58757394	2.97E-04	GL21_ac
S9_57905432	9	57905432	3.03E-04	GL28_14/E2
S9_57545053	9	57545053	3.17E-04	GL35_ac
S9_55566804	9	55566804	3.22E-04	GL49_13/E1
S9_57670282	9	57670282	3.68E-04	GL21_13/E1
S9_52908858	9	52908858	3.73E-04	GL35_13/E1
S9_58757394	9	58757394	3.99E-04	GL42_14/E2
S9_58114502	9	58114502	4.02E-04	GL21_13/E1
S9_58684354	9	58684354	4.04E-04	GL42_ac

SNP	Chromosome	Position	P.value	Trait
S9_58761955	9	58761955	4.09E-04	GL28_13/E1
S9_57666338	9	57666338	4.27E-04	GL28_13/E1
S9_58114502	9	58114502	4.37E-04	GL28_13/E1
S9_45915919	9	45915919	4.46E-04	GL7_ac
S9_2233856	9	2233856	4.48E-04	GL35_ac
S9_55869446	9	55869446	4.49E-04	GL28_13/E1
S9_2233856	9	2233856	4.50E-04	GL7_ac
S9_47241922	9	47241922	4.60E-04	GL21_13/E1
S9_47241963	9	47241963	4.60E-04	GL21_13/E1
S9_51434058	9	51434058	4.71E-04	GL14_13/E1
S9_57541114	9	57541114	4.85E-04	GL42_ac
S9_2233856	9	2233856	5.06E-04	GL21_14/E2
S9_57666338	9	57666338	5.33E-04	GL42_13/E1
S9_57811319	9	57811319	5.36E-04	GL21_13/E1
S9_55566804	9	55566804	5.37E-04	GL35_13/E1
S9_55869446	9	55869446	5.55E-04	GL21_13/E1
S9_2233856	9	2233856	5.68E-04	GL35_14/E2
S9_57947937	9	57947937	5.78E-04	GL7_13/E1
S9_58295230	9	58295230	5.79E-04	GL28_ac
S9_54080354	9	54080354	5.83E-04	GL21_ac
S9_54080354	9	54080354	6.17E-04	GL49_13/E1
S9_57781398	9	57781398	6.31E-04	GL21_13/E1
S9_52908858	9	52908858	6.45E-04	GL49_13/E1
S9_3933616	9	3933616	6.50E-04	GL28_ac
S9_57947937	9	57947937	6.51E-04	GL21_ac
S9_57905432	9	57905432	6.51E-04	GL21_13/E1
S9_57545053	9	57545053	6.58E-04	GL28_14/E2
S9_58757504	9	58757504	6.60E-04	GL28_13/E1
S9_59371922	9	59371922	6.74E-04	GL35_ac
S9_45915919	9	45915919	6.90E-04	GL14_ac
S9_52908858	9	52908858	6.91E-04	GL49_ac
S9_51434058	9	51434058	6.98E-04	GL7_13/E1
S9_57904462	9	57904462	7.09E-04	GL28_13/E1
S9_58761955	9	58761955	7.10E-04	GL21_13/E1
S9_43155530	9	43155530	7.15E-04	GL21_14/E2
S9_2233856	9	2233856	7.30E-04	GL14_ac
S9_58684354	9	58684354	7.32E-04	GL49_13/E1
S9_58684354	9	58684354	7.42E-04	GL42_13/E1
S9_153348	9	153348	7.56E-04	GL42_ac
S9_153350	9	153350	7.56E-04	GL42_ac
S9_153356	9	153356	7.56E-04	GL42_ac
S9_58757394	9	58757394	7.56E-04	GL49_ac
S9_57875319	9	57875319	7.59E-04	GL21_13/E1
S9_57905432	9	57905432	7.62E-04	GL28_13/E1
S9_58295230	9	58295230	7.65E-04	GL21_13/E1
S9_58684354	9	58684354	7.65E-04	GL42_ac
S9_57947937	9	57947937	7.76E-04	GL14_13/E1
S9_57904462	9	57904462	7.87E-04	GL21_13/E1
S9_58757394	9	58757394	7.87E-04	GL35_13/E1
S9_57811310	9	57811310	8.13E-04	GL21_13/E1
S9_54080354	9	54080354	8.17E-04	GL14_ac

SNP	Chromosome	Position	P.value	Trait
S9_54857339	9	54857339	8.27E-04	GL49_14/E2
S9_55566804	9	55566804	8.28E-04	GL28_13/E1
S9_58541198	9	58541198	8.52E-04	GL14_ac
S9_58295230	9	58295230	8.56E-04	GL35_ac
S9_57947937	9	57947937	8.59E-04	GL14_ac
S9_52908858	9	52908858	8.64E-04	GL42_13/E1
S9_47241922	9	47241922	9.02E-04	GL14_13/E1
S9_47241963	9	47241963	9.02E-04	GL14_13/E1
S9_51434058	9	51434058	9.09E-04	GL21_13/E1
S9_58684354	9	58684354	9.14E-04	GL35_ac
S9_57657251	9	57657251	9.18E-04	GL49_14/E2
S9_45915919	9	45915919	9.21E-04	GL14_14/E2
S9_57545053	9	57545053	9.40E-04	GL49_14/E2
S9_58504227	9	58504227	9.44E-04	GL35_ac
S9_57811310	9	57811310	9.46E-04	GL28_ac
S9_57905432	9	57905432	9.50E-04	GL28_ac
S9_58757394	9	58757394	9.53E-04	GL28_13/E1
S9_59371922	9	59371922	9.59E-04	GL14_14/E2
S9_54080354	9	54080354	9.63E-04	GL28_ac
S9_57780832	9	57780832	9.93E-04	GL28_13/E1
S9_57780833	9	57780833	9.93E-04	GL28_13/E1
S9_57780853	9	57780853	9.93E-04	GL28_13/E1
S9_55566804	9	55566804	9.96E-04	GL35_ac
S9_58541198	9	58541198	1.01E-03	GL28_13/E1
S9_57541114	9	57541114	1.01E-03	GL35_ac
S9_57811319	9	57811319	1.06E-03	GL28_13/E1
S9_56572848	9	56572848	1.06E-03	GL35_ac
S9_57545053	9	57545053	1.07E-03	GL21_13/E1
S9_58748351	9	58748351	1.07E-03	GL7_ac
S9_58748316	9	58748316	1.07E-03	GL7_ac
S9_58748317	9	58748317	1.07E-03	GL7_ac
S9_58748322	9	58748322	1.07E-03	GL7_ac
S9_57804067	9	57804067	1.08E-03	GL28_13/E1
S9_58504227	9	58504227	1.08E-03	GL14_14/E2
S9_6802694	9	6802694	1.10E-03	GL42_13/E1
S9_6802694	9	6802694	1.10E-03	GL42_13/E1
S9_6802694	9	6802694	1.10E-03	GL42_13/E1
S9_6802788	9	6802788	1.10E-03	GL42_13/E1
S9_6802789	9	6802789	1.10E-03	GL42_13/E1
S9_6802790	9	6802790	1.10E-03	GL42_13/E1
S9_57804067	9	57804067	1.10E-03	GL21_13/E1
S9_54857339	9	54857339	1.11E-03	GL42_14/E2
S9_57947937	9	57947937	1.12E-03	GL28_ac
S9_52908858	9	52908858	1.13E-03	GL28_13/E1
S9_52908858	9	52908858	1.14E-03	GL42_ac
S9_57811319	9	57811319	1.16E-03	GL28_ac
S9_53867527	9	53867527	1.18E-03	GL21_ac
S9_53867526	9	53867526	1.18E-03	GL21_ac
S9_53867528	9	53867528	1.18E-03	GL21_ac
S9_53867529	9	53867529	1.18E-03	GL21_ac
S9_47006103	9	47006103	1.18E-03	GL49_ac

SNP	Chromosome	Position	P.value	Trait
S9_47006126	9	47006126	1.18E-03	GL49_ac
S9_47006102	9	47006102	1.18E-03	GL49_ac
S9_2233856	9	2233856	1.18E-03	GL14_14/E2
S9_57670282	9	57670282	1.20E-03	GL28_13/E1
S9_2233856	9	2233856	1.21E-03	GL49_ac
S9_57811310	9	57811310	1.22E-03	GL28_13/E1
S9_54080354	9	54080354	1.23E-03	GL7_ac
S9_58291473	9	58291473	1.26E-03	GL21_13/E1
S9_56943758	9	56943758	1.27E-03	GL28_13/E1
S9_58748326	9	58748326	1.27E-03	GL7_ac
S9_56943758	9	56943758	1.27E-03	GL21_13/E1
S9_56572848	9	56572848	1.28E-03	GL21_13/E1
S9_59318053	9	59318053	1.29E-03	GL28_13/E1
S9_6802694	9	6802694	1.30E-03	GL35_13/E1
S9_6802694	9	6802694	1.30E-03	GL35_13/E1
S9_6802694	9	6802694	1.30E-03	GL35_13/E1
S9_6802788	9	6802788	1.30E-03	GL35_13/E1
S9_6802789	9	6802789	1.30E-03	GL35_13/E1
S9_6802790	9	6802790	1.30E-03	GL35_13/E1
S9_58757394	9	58757394	1.31E-03	GL21_13/E1
S9_41678371	9	41678371	1.32E-03	GL49_ac
S9_41678381	9	41678381	1.32E-03	GL49_ac
S9_41678382	9	41678382	1.32E-03	GL49_ac
S9_58177614	9	58177614	1.32E-03	GL28_13/E1
S9_57776578	9	57776578	1.32E-03	GL49_14/E2
S9_57875319	9	57875319	1.33E-03	GL42_13/E1
S9_57545053	9	57545053	1.33E-03	GL21_14/E2
S9_58748351	9	58748351	1.33E-03	GL14_ac
S9_58748316	9	58748316	1.33E-03	GL14_ac
S9_58748317	9	58748317	1.33E-03	GL14_ac
S9_58748322	9	58748322	1.33E-03	GL14_ac
S9_57780832	9	57780832	1.34E-03	GL21_13/E1
S9_57780833	9	57780833	1.34E-03	GL21_13/E1
S9_57780853	9	57780853	1.34E-03	GL21_13/E1
S9_58761955	9	58761955	1.38E-03	GL7_ac
S9_57776578	9	57776578	1.38E-03	GL28_13/E1
S9_58748326	9	58748326	1.39E-03	GL14_ac
S9_57545053	9	57545053	1.39E-03	GL7_ac
S9_40815469	9	40815469	1.40E-03	GL42_ac
S9_58761955	9	58761955	1.40E-03	GL21_ac
S9_59371922	9	59371922	1.40E-03	GL42_ac
S9_57811319	9	57811319	1.41E-03	GL28_14/E2
S9_57811319	9	57811319	1.42E-03	GL49_14/E2
S9_58541198	9	58541198	1.43E-03	GL14_14/E2
S9_3055421	9	3055421	1.43E-03	GL7_13/E1
S9_55566804	9	55566804	1.45E-03	GL21_13/E1
S9_54080354	9	54080354	1.45E-03	GL49_ac
S9_54080354	9	54080354	1.45E-03	GL7_13/E1
S9_58295230	9	58295230	1.46E-03	GL21_14/E2
S9_47241922	9	47241922	1.46E-03	GL35_13/E1
S9_47241963	9	47241963	1.46E-03	GL35_13/E1

SNP	Chromosome	Position	P.value	Trait
S9_56943756	9	56943756	1.47E-03	GL21_13/E1
S9_56943761	9	56943761	1.47E-03	GL21_13/E1
S9_56943762	9	56943762	1.47E-03	GL21_13/E1
S9_56943763	9	56943763	1.47E-03	GL21_13/E1
S9_56943773	9	56943773	1.47E-03	GL21_13/E1
S9_56943774	9	56943774	1.47E-03	GL21_13/E1
S9_56943776	9	56943776	1.47E-03	GL21_13/E1
S9_56943782	9	56943782	1.47E-03	GL21_13/E1
S9_55566804	9	55566804	1.47E-03	GL21_ac
S9_57811310	9	57811310	1.48E-03	GL21_ac
S9_53867527	9	53867527	1.49E-03	GL28_ac
S9_53867526	9	53867526	1.49E-03	GL28_ac
S9_53867528	9	53867528	1.49E-03	GL28_ac
S9_53867529	9	53867529	1.49E-03	GL28_ac
S9_54080354	9	54080354	1.50E-03	GL14_13/E1
S9_56594405	9	56594405	1.51E-03	GL42_13/E1
S9_56594417	9	56594417	1.51E-03	GL42_13/E1
S9_57911513	9	57911513	1.53E-03	GL21_13/E1
S9_57911514	9	57911514	1.53E-03	GL21_13/E1
S9_57911519	9	57911519	1.53E-03	GL21_13/E1
S9_57911522	9	57911522	1.53E-03	GL21_13/E1
S9_57811268	9	57811268	1.54E-03	GL28_13/E1
S9_57781398	9	57781398	1.54E-03	GL7_13/E1
S9_55969226	9	55969226	1.54E-03	GL49_13/E1
S9_58541198	9	58541198	1.55E-03	GL21_13/E1
S9_58295230	9	58295230	1.57E-03	GL28_14/E2
S9_3933616	9	3933616	1.57E-03	GL35_14/E2
S9_57529022	9	57529022	1.57E-03	GL28_14/E2
S9_55566804	9	55566804	1.58E-03	GL42_13/E1
S9_57787432	9	57787432	1.58E-03	GL28_13/E1
S9_58839540	9	58839540	1.60E-03	GL7_14/E2
S9_58761955	9	58761955	1.61E-03	GL35_13/E1
S9_59311585	9	59311585	1.61E-03	GL7_ac
S9_58114502	9	58114502	1.62E-03	GL7_ac
S9_58757394	9	58757394	1.63E-03	GL42_13/E1
S9_163434	9	163434	1.64E-03	GL14_ac
S9_58114502	9	58114502	1.65E-03	GL28_ac
S9_57776578	9	57776578	1.67E-03	GL21_13/E1
S9_56943756	9	56943756	1.67E-03	GL28_13/E1
S9_56943761	9	56943761	1.67E-03	GL28_13/E1
S9_56943762	9	56943762	1.67E-03	GL28_13/E1
S9_56943763	9	56943763	1.67E-03	GL28_13/E1
S9_56943773	9	56943773	1.67E-03	GL28_13/E1
S9_56943774	9	56943774	1.67E-03	GL28_13/E1
S9_56943776	9	56943776	1.67E-03	GL28_13/E1
S9_56943782	9	56943782	1.67E-03	GL28_13/E1
S9_59311585	9	59311585	1.68E-03	GL7_13/E1
S9_57911513	9	57911513	1.69E-03	GL7_ac
S9_57911514	9	57911514	1.69E-03	GL7_ac
S9_57911519	9	57911519	1.69E-03	GL7_ac
S9_57911522	9	57911522	1.69E-03	GL7_ac

SNP	Chromosome	Position	P.value	Trait
S9_57545053	9	57545053	1.69E-03	GL28_ac
S9_3055421	9	3055421	1.70E-03	GL14_13/E1
S9_57670282	9	57670282	1.71E-03	GL21_ac
S9_57811319	9	57811319	1.73E-03	GL21_ac
S9_54908173	9	54908173	1.73E-03	GL49_13/E1
S9_54908187	9	54908187	1.73E-03	GL49_13/E1
S9_54908190	9	54908190	1.73E-03	GL49_13/E1
S9_54908191	9	54908191	1.73E-03	GL49_13/E1
S9_54908192	9	54908192	1.73E-03	GL49_13/E1
S9_54908193	9	54908193	1.73E-03	GL49_13/E1
S9_57776578	9	57776578	1.74E-03	GL14_14/E2
S9_53238030	9	53238030	1.74E-03	GL42_13/E1
S9_58841852	9	58841852	1.76E-03	GL28_14/E2
S9_59322668	9	59322668	1.77E-03	GL7_13/E1
S9_57911513	9	57911513	1.77E-03	GL28_13/E1
S9_57911514	9	57911514	1.77E-03	GL28_13/E1
S9_57911519	9	57911519	1.77E-03	GL28_13/E1
S9_57911522	9	57911522	1.77E-03	GL28_13/E1
S9_57911518	9	57911518	1.78E-03	GL7_ac
S9_58114502	9	58114502	1.78E-03	GL21_ac
S9_58114502	9	58114502	1.79E-03	GL28_14/E2
S9_59371922	9	59371922	1.84E-03	GL21_14/E2
S9_52908858	9	52908858	1.84E-03	GL28_ac
S9_45915919	9	45915919	1.85E-03	GL35_14/E2
S9_53238030	9	53238030	1.85E-03	GL35_13/E1
S9_56572848	9	56572848	1.86E-03	GL21_ac
S9_57911518	9	57911518	1.86E-03	GL21_13/E1
S9_57947937	9	57947937	1.87E-03	GL35_13/E1
S9_58761955	9	58761955	1.89E-03	GL14_ac
S9_163434	9	163434	1.90E-03	GL49_ac
S9_2233856	9	2233856	1.91E-03	GL21_ac
S9_2238350	9	2238350	1.91E-03	GL35_ac
S9_47241922	9	47241922	1.92E-03	GL28_13/E1
S9_47241963	9	47241963	1.92E-03	GL28_13/E1
S9_59322668	9	59322668	1.92E-03	GL14_13/E1
S9_59207864	9	59207864	1.92E-03	GL21_13/E1
S9_57545053	9	57545053	1.93E-03	GL14_ac
S9_57065277	9	57065277	1.94E-03	GL14_14/E2
S9_58757504	9	58757504	1.97E-03	GL21_13/E1
S9_57811319	9	57811319	1.99E-03	GL7_ac
S9_59207864	9	59207864	2.00E-03	GL7_ac
S9_58757504	9	58757504	2.00E-03	GL35_13/E1
S9_57781398	9	57781398	2.01E-03	GL14_13/E1
S9_57065264	9	57065264	2.01E-03	GL21_14/E2
S9_58634599	9	58634599	2.02E-03	GL14_ac
S9_58634609	9	58634609	2.02E-03	GL14_ac
S9_45915919	9	45915919	2.04E-03	GL7_13/E1
S9_57914531	9	57914531	2.06E-03	GL42_ac
S9_2238350	9	2238350	2.06E-03	GL49_13/E1
S9_47241922	9	47241922	2.07E-03	GL7_13/E1
S9_47241963	9	47241963	2.07E-03	GL7_13/E1

SNP	Chromosome	Position	P.value	Trait
S9_58541198	9	58541198	2.08E-03	GL21_ac
S9_2546489	9	2546489	2.09E-03	GL14_14/E2
S9_59322668	9	59322668	2.10E-03	GL28_13/E1
S9_57670282	9	57670282	2.10E-03	GL28_ac
S9_57541114	9	57541114	2.11E-03	GL42_13/E1
S9_58757394	9	58757394	2.11E-03	GL14_ac
S9_56572848	9	56572848	2.12E-03	GL28_ac
S9_58177614	9	58177614	2.12E-03	GL21_13/E1
S9_55492025	9	55492025	2.14E-03	GL21_14/E2
S9_55492019	9	55492019	2.14E-03	GL21_14/E2
S9_55492020	9	55492020	2.14E-03	GL21_14/E2
S9_57781398	9	57781398	2.15E-03	GL28_13/E1
S9_58761955	9	58761955	2.17E-03	GL7_13/E1
S9_58114502	9	58114502	2.18E-03	GL7_14/E2
S9_58748326	9	58748326	2.20E-03	GL14_13/E1
S9_58748326	9	58748326	2.20E-03	GL7_13/E1
S9_57065277	9	57065277	2.21E-03	GL21_13/E1
S9_58748316	9	58748316	2.21E-03	GL7_13/E1
S9_58748317	9	58748317	2.21E-03	GL7_13/E1
S9_58748322	9	58748322	2.21E-03	GL7_13/E1
S9_58748351	9	58748351	2.21E-03	GL7_13/E1
S9_53172825	9	53172825	2.22E-03	GL21_14/E2
S9_53172828	9	53172828	2.22E-03	GL21_14/E2
S9_3933616	9	3933616	2.22E-03	GL14_ac
S9_57947937	9	57947937	2.24E-03	GL49_13/E1
S9_58761955	9	58761955	2.25E-03	GL14_13/E1
S9_47241922	9	47241922	2.25E-03	GL21_ac
S9_47241963	9	47241963	2.25E-03	GL21_ac
S9_59322668	9	59322668	2.27E-03	GL21_13/E1
S9_55566804	9	55566804	2.27E-03	GL28_ac
S9_58684354	9	58684354	2.28E-03	GL35_13/E1
S9_58295230	9	58295230	2.29E-03	GL28_13/E1
S9_58757504	9	58757504	2.32E-03	GL35_ac
S9_58748316	9	58748316	2.33E-03	GL14_13/E1
S9_58748317	9	58748317	2.33E-03	GL14_13/E1
S9_58748322	9	58748322	2.33E-03	GL14_13/E1
S9_58748351	9	58748351	2.33E-03	GL14_13/E1
S9_58295230	9	58295230	2.34E-03	GL35_13/E1
S9_57529022	9	57529022	2.34E-03	GL28_ac
S9_57911518	9	57911518	2.34E-03	GL28_13/E1
S9_58684354	9	58684354	2.34E-03	GL42_13/E1
S9_58541198	9	58541198	2.35E-03	GL28_ac
S9_58761955	9	58761955	2.35E-03	GL28_ac
S9_56101938	9	56101938	2.36E-03	GL35_ac
S9_57666338	9	57666338	2.36E-03	GL35_13/E1
S9_55067209	9	55067209	2.40E-03	GL49_ac
S9_55067231	9	55067231	2.40E-03	GL49_ac
S9_55067234	9	55067234	2.40E-03	GL49_ac
S9_59371922	9	59371922	2.42E-03	GL14_ac
S9_58761955	9	58761955	2.42E-03	GL35_ac
S9_59322668	9	59322668	2.45E-03	GL7_ac

SNP	Chromosome	Position	P.value	Trait
S9_58684354	9	58684354	2.46E-03	GL28_13/E1
S9_3933616	9	3933616	2.47E-03	GL21_ac
S9_57541114	9	57541114	2.47E-03	GL35_13/E1
S9_47006102	9	47006102	2.48E-03	GL7_14/E2
S9_47006103	9	47006103	2.48E-03	GL7_14/E2
S9_47006126	9	47006126	2.48E-03	GL7_14/E2
S9_57065264	9	57065264	2.48E-03	GL28_14/E2
S9_55004994	9	55004994	2.49E-03	GL42_14/E2
S9_56594405	9	56594405	2.50E-03	GL28_13/E1
S9_56594417	9	56594417	2.50E-03	GL28_13/E1
S9_57545053	9	57545053	2.50E-03	GL21_ac
S9_41577016	9	41577016	2.50E-03	GL49_14/E2
S9_58841852	9	58841852	2.52E-03	GL28_13/E1
S9_2233856	9	2233856	2.52E-03	GL28_14/E2
S9_58841852	9	58841852	2.52E-03	GL21_13/E1
S9_2754294	9	2754294	2.53E-03	GL21_ac
S9_59318053	9	59318053	2.54E-03	GL21_13/E1
S9_47241922	9	47241922	2.55E-03	GL28_ac
S9_47241963	9	47241963	2.55E-03	GL28_ac
S9_47006103	9	47006103	2.55E-03	GL49_14/E2
S9_47006126	9	47006126	2.55E-03	GL49_14/E2
S9_47006102	9	47006102	2.55E-03	GL49_14/E2
S9_57666338	9	57666338	2.55E-03	GL49_13/E1
S9_56572848	9	56572848	2.56E-03	GL28_13/E1
S9_57811310	9	57811310	2.56E-03	GL28_14/E2
S9_57065264	9	57065264	2.57E-03	GL14_14/E2
S9_59291140	9	59291140	2.57E-03	GL35_ac
S9_57811319	9	57811319	2.58E-03	GL7_13/E1
S9_54973417	9	54973417	2.60E-03	GL28_14/E2
S9_2233856	9	2233856	2.60E-03	GL28_ac
S9_58634599	9	58634599	2.62E-03	GL7_13/E1
S9_58634609	9	58634609	2.62E-03	GL7_13/E1
S9_58973752	9	58973752	2.62E-03	GL49_14/E2
S9_58973755	9	58973755	2.62E-03	GL49_14/E2
S9_58973756	9	58973756	2.62E-03	GL49_14/E2
S9_374484	9	374484	2.63E-03	GL49_ac
S9_374483	9	374483	2.63E-03	GL49_ac
S9_51434058	9	51434058	2.66E-03	GL28_13/E1
S9_56101938	9	56101938	2.67E-03	GL21_14/E2
S9_58541198	9	58541198	2.69E-03	GL35_13/E1
S9_58504227	9	58504227	2.69E-03	GL7_ac
S9_41430950	9	41430950	2.70E-03	GL35_14/E2
S9_8117469	9	8117469	2.72E-03	GL35_14/E2
S9_58114502	9	58114502	2.72E-03	GL35_ac
S9_58748326	9	58748326	2.73E-03	GL28_13/E1
S9_56520725	9	56520725	2.76E-03	GL14_14/E2
S9_3933616	9	3933616	2.78E-03	GL42_ac
S9_56535023	9	56535023	2.79E-03	GL21_13/E1
S9_56535046	9	56535046	2.79E-03	GL21_13/E1
S9_6802694	9	6802694	2.80E-03	GL28_13/E1
S9_6802694	9	6802694	2.80E-03	GL28_13/E1

SNP	Chromosome	Position	P.value	Trait
S9_6802694	9	6802694	2.80E-03	GL28_13/E1
S9_6802788	9	6802788	2.80E-03	GL28_13/E1
S9_6802789	9	6802789	2.80E-03	GL28_13/E1
S9_6802790	9	6802790	2.80E-03	GL28_13/E1
S9_57875319	9	57875319	2.82E-03	GL35_13/E1
S9_44414073	9	44414073	2.83E-03	GL42_ac
S9_55067209	9	55067209	2.84E-03	GL49_13/E1
S9_55067231	9	55067231	2.84E-03	GL49_13/E1
S9_55067234	9	55067234	2.84E-03	GL49_13/E1
S9_52908858	9	52908858	2.85E-03	GL21_13/E1
S9_57811310	9	57811310	2.86E-03	GL7_ac
S9_4652966	9	4652966	2.86E-03	GL35_14/E2
S9_59311585	9	59311585	2.86E-03	GL49_13/E1
S9_10261847	9	10261847	2.87E-03	GL49_14/E2
S9_12066581	9	12066581	2.88E-03	GL7_ac
S9_58684354	9	58684354	2.89E-03	GL35_ac
S9_57670282	9	57670282	2.89E-03	GL7_ac
S9_58757504	9	58757504	2.89E-03	GL14_14/E2
S9_42808999	9	42808999	2.91E-03	GL42_ac
S9_57545053	9	57545053	2.91E-03	GL35_14/E2
S9_57811268	9	57811268	2.93E-03	GL21_13/E1
S9_53565698	9	53565698	2.94E-03	GL49_13/E1
S9_57541114	9	57541114	2.96E-03	GL28_13/E1
S9_55566804	9	55566804	3.00E-03	GL42_ac
S9_58748316	9	58748316	3.01E-03	GL28_13/E1
S9_58748317	9	58748317	3.01E-03	GL28_13/E1
S9_58748322	9	58748322	3.01E-03	GL28_13/E1
S9_58748351	9	58748351	3.01E-03	GL28_13/E1
S9_58684354	9	58684354	3.02E-03	GL28_ac
S9_58757394	9	58757394	3.02E-03	GL35_14/E2
S9_56535023	9	56535023	3.03E-03	GL35_ac
S9_56535046	9	56535046	3.03E-03	GL35_ac
S9_2546489	9	2546489	3.03E-03	GL42_ac
S9_57811310	9	57811310	3.06E-03	GL7_13/E1
S9_59371922	9	59371922	3.09E-03	GL35_13/E1
S9_58684354	9	58684354	3.09E-03	GL14_ac
S9_53867526	9	53867526	3.11E-03	GL21_13/E1
S9_53867527	9	53867527	3.11E-03	GL21_13/E1
S9_53867528	9	53867528	3.11E-03	GL21_13/E1
S9_53867529	9	53867529	3.11E-03	GL21_13/E1
S9_56594405	9	56594405	3.11E-03	GL49_13/E1
S9_56594417	9	56594417	3.11E-03	GL49_13/E1
S9_57804067	9	57804067	3.12E-03	GL35_13/E1
S9_53818841	9	53818841	3.12E-03	GL42_ac
S9_57123473	9	57123473	3.15E-03	GL28_13/E1
S9_56572848	9	56572848	3.15E-03	GL14_14/E2
S9_57657884	9	57657884	3.19E-03	GL21_14/E2
S9_56945635	9	56945635	3.20E-03	GL42_13/E1
S9_58508533	9	58508533	3.20E-03	GL7_ac
S9_54080354	9	54080354	3.20E-03	GL35_ac
S9_57905432	9	57905432	3.21E-03	GL7_13/E1

SNP	Chromosome	Position	P.value	Trait
S9_54080354	9	54080354	3.22E-03	GL21_13/E1
S9_58748326	9	58748326	3.23E-03	GL21_13/E1
S9_57776578	9	57776578	3.23E-03	GL49_ac
S9_56943756	9	56943756	3.23E-03	GL14_13/E1
S9_56943761	9	56943761	3.23E-03	GL14_13/E1
S9_56943762	9	56943762	3.23E-03	GL14_13/E1
S9_56943763	9	56943763	3.23E-03	GL14_13/E1
S9_56943773	9	56943773	3.23E-03	GL14_13/E1
S9_56943774	9	56943774	3.23E-03	GL14_13/E1
S9_56943776	9	56943776	3.23E-03	GL14_13/E1
S9_56943782	9	56943782	3.23E-03	GL14_13/E1
S9_5767929	9	5767929	3.24E-03	GL49_14/E2
S9_2754294	9	2754294	3.25E-03	GL28_ac
S9_52908858	9	52908858	3.26E-03	GL7_ac
S9_57776578	9	57776578	3.26E-03	GL28_14/E2
S9_59311585	9	59311585	3.27E-03	GL14_13/E1
S9_57139943	9	57139943	3.27E-03	GL21_13/E1
S9_55969226	9	55969226	3.27E-03	GL35_13/E1
S9_52908858	9	52908858	3.28E-03	GL7_13/E1
S9_56535023	9	56535023	3.28E-03	GL42_ac
S9_56535046	9	56535046	3.28E-03	GL42_ac
S9_55492025	9	55492025	3.30E-03	GL21_ac
S9_55492019	9	55492019	3.30E-03	GL21_ac
S9_55492020	9	55492020	3.30E-03	GL21_ac
S9_59156843	9	59156843	3.30E-03	GL35_ac
S9_57065277	9	57065277	3.31E-03	GL28_14/E2
S9_57925883	9	57925883	3.32E-03	GL49_13/E1
S9_374484	9	374484	3.34E-03	GL42_ac
S9_374483	9	374483	3.34E-03	GL42_ac
S9_57905432	9	57905432	3.37E-03	GL14_14/E2
S9_47006103	9	47006103	3.38E-03	GL7_ac
S9_47006126	9	47006126	3.38E-03	GL7_ac
S9_47006102	9	47006102	3.38E-03	GL7_ac
S9_57065277	9	57065277	3.38E-03	GL21_14/E2
S9_55969226	9	55969226	3.39E-03	GL42_13/E1
S9_57904462	9	57904462	3.39E-03	GL28_14/E2
S9_57904462	9	57904462	3.40E-03	GL7_13/E1
S9_57776578	9	57776578	3.40E-03	GL35_13/E1
S9_52908858	9	52908858	3.40E-03	GL14_13/E1
S9_58541198	9	58541198	3.40E-03	GL14_13/E1
S9_2754294	9	2754294	3.41E-03	GL7_ac
S9_58541198	9	58541198	3.42E-03	GL35_ac
S9_52923067	9	52923067	3.42E-03	GL49_13/E1
S9_42808999	9	42808999	3.43E-03	GL49_14/E2
S9_56101938	9	56101938	3.43E-03	GL49_ac
S9_54973417	9	54973417	3.44E-03	GL49_14/E2
S9_59371922	9	59371922	3.47E-03	GL21_ac
S9_55969226	9	55969226	3.48E-03	GL42_ac
S9_56943758	9	56943758	3.50E-03	GL14_13/E1
S9_58684354	9	58684354	3.50E-03	GL49_ac
S9_3055421	9	3055421	3.52E-03	GL21_13/E1

SNP	Chromosome	Position	P.value	Trait
S9_58748351	9	58748351	3.54E-03	GL14_14/E2
S9_58748316	9	58748316	3.54E-03	GL14_14/E2
S9_58748317	9	58748317	3.54E-03	GL14_14/E2
S9_58748322	9	58748322	3.54E-03	GL14_14/E2
S9_58748316	9	58748316	3.54E-03	GL21_13/E1
S9_58748317	9	58748317	3.54E-03	GL21_13/E1
S9_58748322	9	58748322	3.54E-03	GL21_13/E1
S9_58748351	9	58748351	3.54E-03	GL21_13/E1
S9_57905432	9	57905432	3.56E-03	GL21_ac
S9_58541198	9	58541198	3.58E-03	GL7_13/E1
S9_2754294	9	2754294	3.59E-03	GL21_13/E1
S9_54908173	9	54908173	3.59E-03	GL42_13/E1
S9_54908187	9	54908187	3.59E-03	GL42_13/E1
S9_54908190	9	54908190	3.59E-03	GL42_13/E1
S9_54908191	9	54908191	3.59E-03	GL42_13/E1
S9_54908192	9	54908192	3.59E-03	GL42_13/E1
S9_54908193	9	54908193	3.59E-03	GL42_13/E1
S9_54330774	9	54330774	3.62E-03	GL42_ac
S9_56101938	9	56101938	3.62E-03	GL49_14/E2
S9_58634599	9	58634599	3.62E-03	GL14_13/E1
S9_58634609	9	58634609	3.62E-03	GL14_13/E1
S9_519082	9	519082	3.63E-03	GL7_ac
S9_519098	9	519098	3.63E-03	GL7_ac
S9_57904462	9	57904462	3.63E-03	GL49_13/E1
S9_56535023	9	56535023	3.65E-03	GL49_ac
S9_56535046	9	56535046	3.65E-03	GL49_ac
S9_59311585	9	59311585	3.65E-03	GL21_13/E1
S9_42808999	9	42808999	3.65E-03	GL49_ac
S9_57666338	9	57666338	3.65E-03	GL21_13/E1
S9_59371922	9	59371922	3.66E-03	GL28_ac
S9_54330774	9	54330774	3.66E-03	GL42_14/E2
S9_55869446	9	55869446	3.66E-03	GL14_13/E1
S9_49338003	9	49338003	3.68E-03	GL14_14/E2
S9_58291473	9	58291473	3.68E-03	GL28_13/E1
S9_57529022	9	57529022	3.70E-03	GL21_ac
S9_57545053	9	57545053	3.70E-03	GL49_ac
S9_58291473	9	58291473	3.73E-03	GL14_13/E1
S9_57833859	9	57833859	3.74E-03	GL28_13/E1
S9_57833860	9	57833860	3.74E-03	GL28_13/E1
S9_56542015	9	56542015	3.75E-03	GL28_13/E1
S9_56101938	9	56101938	3.75E-03	GL28_ac
S9_2754294	9	2754294	3.77E-03	GL28_13/E1
S9_56101938	9	56101938	3.77E-03	GL28_14/E2
S9_55869446	9	55869446	3.78E-03	GL35_13/E1
S9_57776578	9	57776578	3.79E-03	GL42_13/E1
S9_56549681	9	56549681	3.79E-03	GL28_13/E1
S9_57781398	9	57781398	3.80E-03	GL7_ac
S9_57057648	9	57057648	3.82E-03	GL21_13/E1
S9_57545053	9	57545053	3.82E-03	GL35_13/E1
S9_41678371	9	41678371	3.85E-03	GL7_13/E1
S9_41678381	9	41678381	3.85E-03	GL7_13/E1

SNP	Chromosome	Position	P.value	Trait
S9_41678382	9	41678382	3.85E-03	GL7_13/E1
S9_55566804	9	55566804	3.85E-03	GL49_ac
S9_55095671	9	55095671	3.87E-03	GL42_ac
S9_47241922	9	47241922	3.89E-03	GL49_ac
S9_47241963	9	47241963	3.89E-03	GL49_ac
S9_57792165	9	57792165	3.89E-03	GL7_ac
S9_57792168	9	57792168	3.89E-03	GL7_ac
S9_57792171	9	57792171	3.89E-03	GL7_ac
S9_57670230	9	57670230	3.89E-03	GL21_13/E1
S9_57670262	9	57670262	3.89E-03	GL21_13/E1
S9_57780833	9	57780833	3.92E-03	GL21_14/E2
S9_57780853	9	57780853	3.92E-03	GL21_14/E2
S9_57780832	9	57780832	3.92E-03	GL21_14/E2
S9_59312050	9	59312050	3.93E-03	GL14_14/E2
S9_2233856	9	2233856	3.94E-03	GL7_14/E2
S9_58295230	9	58295230	3.97E-03	GL35_14/E2
S9_58867145	9	58867145	3.98E-03	GL28_13/E1
S9_57123473	9	57123473	4.00E-03	GL21_13/E1
S9_52923075	9	52923075	4.00E-03	GL42_13/E1
S9_58841852	9	58841852	4.02E-03	GL28_ac
S9_58841852	9	58841852	4.03E-03	GL14_14/E2
S9_53238030	9	53238030	4.03E-03	GL21_ac
S9_58748326	9	58748326	4.04E-03	GL14_14/E2
S9_58531766	9	58531766	4.05E-03	GL14_14/E2
S9_55869446	9	55869446	4.09E-03	GL7_13/E1
S9_59311585	9	59311585	4.10E-03	GL49_ac
S9_2238350	9	2238350	4.10E-03	GL28_ac
S9_6802694	9	6802694	4.13E-03	GL49_13/E1
S9_6802694	9	6802694	4.13E-03	GL49_13/E1
S9_6802694	9	6802694	4.13E-03	GL49_13/E1
S9_6802788	9	6802788	4.13E-03	GL49_13/E1
S9_6802789	9	6802789	4.13E-03	GL49_13/E1
S9_6802790	9	6802790	4.13E-03	GL49_13/E1
S9_54093550	9	54093550	4.13E-03	GL49_13/E1
S9_58332115	9	58332115	4.14E-03	GL28_14/E2
S9_59207864	9	59207864	4.16E-03	GL28_13/E1
S9_53172825	9	53172825	4.17E-03	GL42_14/E2
S9_53172828	9	53172828	4.17E-03	GL42_14/E2
S9_44637848	9	44637848	4.18E-03	GL14_14/E2
S9_57947937	9	57947937	4.19E-03	GL49_ac
S9_57947937	9	57947937	4.20E-03	GL42_ac
S9_54080354	9	54080354	4.21E-03	GL28_13/E1
S9_59207864	9	59207864	4.22E-03	GL21_ac
S9_12066581	9	12066581	4.23E-03	GL49_13/E1
S9_58291473	9	58291473	4.24E-03	GL7_13/E1
S9_58757504	9	58757504	4.24E-03	GL28_ac
S9_57545053	9	57545053	4.25E-03	GL28_13/E1
S9_58757394	9	58757394	4.26E-03	GL49_14/E2
S9_57780832	9	57780832	4.27E-03	GL14_13/E1
S9_57780833	9	57780833	4.27E-03	GL14_13/E1
S9_57780853	9	57780853	4.27E-03	GL14_13/E1

SNP	Chromosome	Position	P.value	Trait
S9_58634599	9	58634599	4.27E-03	GL7_ac
S9_58634609	9	58634609	4.27E-03	GL7_ac
S9_56101938	9	56101938	4.27E-03	GL21_13/E1
S9_5512888	9	5512888	4.28E-03	GL21_14/E2
S9_56535023	9	56535023	4.29E-03	GL42_14/E2
S9_56535046	9	56535046	4.29E-03	GL42_14/E2
S9_56943758	9	56943758	4.30E-03	GL35_13/E1
S9_10386294	9	10386294	4.33E-03	GL14_14/E2
S9_52923065	9	52923065	4.37E-03	GL49_13/E1
S9_52923066	9	52923066	4.37E-03	GL49_13/E1
S9_52923069	9	52923069	4.37E-03	GL49_13/E1
S9_52923070	9	52923070	4.37E-03	GL49_13/E1
S9_52923072	9	52923072	4.37E-03	GL49_13/E1
S9_3933616	9	3933616	4.39E-03	GL28_14/E2
S9_57065277	9	57065277	4.39E-03	GL28_13/E1
S9_58508533	9	58508533	4.40E-03	GL21_ac
S9_55095671	9	55095671	4.41E-03	GL21_14/E2
S9_45915919	9	45915919	4.43E-03	GL35_ac
S9_59371922	9	59371922	4.44E-03	GL28_13/E1
S9_56101938	9	56101938	4.46E-03	GL21_ac
S9_58757504	9	58757504	4.50E-03	GL21_ac
S9_57947937	9	57947937	4.51E-03	GL35_ac
S9_57657830	9	57657830	4.52E-03	GL21_14/E2
S9_59311585	9	59311585	4.53E-03	GL14_ac
S9_57065277	9	57065277	4.53E-03	GL35_ac
S9_51434058	9	51434058	4.53E-03	GL14_ac
S9_1258977	9	1258977	4.57E-03	GL35_14/E2
S9_6615435	9	6615435	4.57E-03	GL7_14/E2
S9_57811319	9	57811319	4.57E-03	GL49_ac
S9_10386294	9	10386294	4.58E-03	GL21_14/E2
S9_56943758	9	56943758	4.58E-03	GL21_ac
S9_58684354	9	58684354	4.61E-03	GL49_ac
S9_56101938	9	56101938	4.62E-03	GL28_13/E1
S9_58841852	9	58841852	4.62E-03	GL21_ac
S9_52908858	9	52908858	4.64E-03	GL21_ac
S9_59173027	9	59173027	4.65E-03	GL14_14/E2
S9_57069131	9	57069131	4.69E-03	GL35_ac
S9_57069088	9	57069088	4.69E-03	GL35_ac
S9_57069097	9	57069097	4.69E-03	GL35_ac
S9_57069125	9	57069125	4.69E-03	GL35_ac
S9_57670282	9	57670282	4.69E-03	GL7_13/E1
S9_57811310	9	57811310	4.70E-03	GL49_14/E2
S9_52923067	9	52923067	4.70E-03	GL42_13/E1
S9_3055421	9	3055421	4.70E-03	GL14_ac
S9_41430950	9	41430950	4.70E-03	GL42_14/E2
S9_59291140	9	59291140	4.71E-03	GL14_14/E2
S9_55492025	9	55492025	4.73E-03	GL49_ac
S9_55492019	9	55492019	4.73E-03	GL49_ac
S9_55492020	9	55492020	4.73E-03	GL49_ac
S9_56943756	9	56943756	4.74E-03	GL21_ac
S9_56943761	9	56943761	4.74E-03	GL21_ac

SNP	Chromosome	Position	P.value	Trait
S9_56943762	9	56943762	4.74E-03	GL21_ac
S9_56943776	9	56943776	4.74E-03	GL21_ac
S9_56943763	9	56943763	4.74E-03	GL21_ac
S9_56943773	9	56943773	4.74E-03	GL21_ac
S9_56943774	9	56943774	4.74E-03	GL21_ac
S9_56943782	9	56943782	4.74E-03	GL21_ac
S9_57545053	9	57545053	4.78E-03	GL7_13/E1
S9_44414073	9	44414073	4.78E-03	GL49_14/E2
S9_55626875	9	55626875	4.86E-03	GL42_13/E1
S9_55626876	9	55626876	4.86E-03	GL42_13/E1
S9_55492025	9	55492025	4.87E-03	GL49_14/E2
S9_55492019	9	55492019	4.87E-03	GL49_14/E2
S9_55492020	9	55492020	4.87E-03	GL49_14/E2
S9_52923065	9	52923065	4.89E-03	GL42_13/E1
S9_52923066	9	52923066	4.89E-03	GL42_13/E1
S9_52923069	9	52923069	4.89E-03	GL42_13/E1
S9_52923070	9	52923070	4.89E-03	GL42_13/E1
S9_52923072	9	52923072	4.89E-03	GL42_13/E1
S9_153348	9	153348	4.93E-03	GL49_ac
S9_153350	9	153350	4.93E-03	GL49_ac
S9_153356	9	153356	4.93E-03	GL49_ac
S9_57947937	9	57947937	4.94E-03	GL14_14/E2
S9_44414073	9	44414073	4.97E-03	GL49_ac
S9_57811319	9	57811319	4.99E-03	GL14_13/E1
S9_51830298	9	51830298	5.00E-03	GL14_ac
S9_51830251	9	51830251	5.00E-03	GL14_ac
S9_2546489	9	2546489	5.01E-03	GL21_14/E2
S9_54917701	9	54917701	5.01E-03	GL28_14/E2
S9_51434058	9	51434058	5.04E-03	GL49_13/E1
S9_55492025	9	55492025	5.06E-03	GL14_14/E2
S9_55492019	9	55492019	5.06E-03	GL14_14/E2
S9_55492020	9	55492020	5.06E-03	GL14_14/E2
S9_57904462	9	57904462	5.07E-03	GL35_13/E1
S9_41678371	9	41678371	5.07E-03	GL14_13/E1
S9_41678381	9	41678381	5.07E-03	GL14_13/E1
S9_41678382	9	41678382	5.07E-03	GL14_13/E1
S9_55883070	9	55883070	5.08E-03	GL21_13/E1
S9_57811319	9	57811319	5.08E-03	GL35_ac
S9_59173027	9	59173027	5.09E-03	GL28_14/E2
S9_56549681	9	56549681	5.10E-03	GL21_13/E1
S9_57139944	9	57139944	5.11E-03	GL49_14/E2
S9_57216854	9	57216854	5.12E-03	GL7_ac
S9_59311770	9	59311770	5.13E-03	GL14_14/E2
S9_54917701	9	54917701	5.14E-03	GL21_14/E2
S9_57792165	9	57792165	5.15E-03	GL21_13/E1
S9_57792168	9	57792168	5.15E-03	GL21_13/E1
S9_57792171	9	57792171	5.15E-03	GL21_13/E1
S9_44637848	9	44637848	5.16E-03	GL21_14/E2
S9_57225685	9	57225685	5.16E-03	GL7_ac
S9_2233856	9	2233856	5.17E-03	GL42_14/E2
S9_57780832	9	57780832	5.18E-03	GL35_13/E1

SNP	Chromosome	Position	P.value	Trait
S9_57780833	9	57780833	5.18E-03	GL35_13/E1
S9_57780853	9	57780853	5.18E-03	GL35_13/E1
S9_57811310	9	57811310	5.19E-03	GL14_ac
S9_56535023	9	56535023	5.23E-03	GL28_13/E1
S9_56535046	9	56535046	5.23E-03	GL28_13/E1
S9_58127631	9	58127631	5.25E-03	GL28_13/E1
S9_57776578	9	57776578	5.25E-03	GL42_ac
S9_57804067	9	57804067	5.25E-03	GL28_ac
S9_56535023	9	56535023	5.26E-03	GL21_ac
S9_56535046	9	56535046	5.26E-03	GL21_ac
S9_56594405	9	56594405	5.30E-03	GL35_13/E1
S9_56594417	9	56594417	5.30E-03	GL35_13/E1
S9_163434	9	163434	5.33E-03	GL7_ac
S9_56943756	9	56943756	5.34E-03	GL35_13/E1
S9_56943761	9	56943761	5.34E-03	GL35_13/E1
S9_56943762	9	56943762	5.34E-03	GL35_13/E1
S9_56943763	9	56943763	5.34E-03	GL35_13/E1
S9_56943773	9	56943773	5.34E-03	GL35_13/E1
S9_56943774	9	56943774	5.34E-03	GL35_13/E1
S9_56943776	9	56943776	5.34E-03	GL35_13/E1
S9_56943782	9	56943782	5.34E-03	GL35_13/E1
S9_58177614	9	58177614	5.35E-03	GL21_ac
S9_45915919	9	45915919	5.37E-03	GL14_13/E1
S9_57811310	9	57811310	5.38E-03	GL14_13/E1
S9_54080354	9	54080354	5.38E-03	GL28_14/E2
S9_44446459	9	44446459	5.39E-03	GL42_ac
S9_59312050	9	59312050	5.41E-03	GL28_14/E2
S9_59207864	9	59207864	5.42E-03	GL35_ac
S9_58472760	9	58472760	5.44E-03	GL14_13/E1
S9_2754294	9	2754294	5.45E-03	GL7_13/E1
S9_3933616	9	3933616	5.46E-03	GL14_14/E2
S9_57947937	9	57947937	5.49E-03	GL7_14/E2
S9_57119962	9	57119962	5.50E-03	GL21_13/E1
S9_57119965	9	57119965	5.50E-03	GL21_13/E1
S9_57065264	9	57065264	5.50E-03	GL21_13/E1
S9_55004994	9	55004994	5.50E-03	GL42_ac
S9_58295230	9	58295230	5.51E-03	GL42_ac
S9_57641803	9	57641803	5.52E-03	GL28_13/E1
S9_57781398	9	57781398	5.52E-03	GL21_ac
S9_57811268	9	57811268	5.55E-03	GL35_13/E1
S9_3933616	9	3933616	5.56E-03	GL21_14/E2
S9_55969226	9	55969226	5.57E-03	GL28_13/E1
S9_56101938	9	56101938	5.59E-03	GL14_ac
S9_57119962	9	57119962	5.59E-03	GL35_ac
S9_57119965	9	57119965	5.59E-03	GL35_ac
S9_58114502	9	58114502	5.59E-03	GL14_13/E1
S9_52923075	9	52923075	5.61E-03	GL49_13/E1
S9_54917701	9	54917701	5.62E-03	GL49_14/E2
S9_6802694	9	6802694	5.63E-03	GL21_13/E1
S9_6802694	9	6802694	5.63E-03	GL21_13/E1
S9_6802694	9	6802694	5.63E-03	GL21_13/E1

SNP	Chromosome	Position	P.value	Trait
S9_6802788	9	6802788	5.63E-03	GL21_13/E1
S9_6802789	9	6802789	5.63E-03	GL21_13/E1
S9_6802790	9	6802790	5.63E-03	GL21_13/E1
S9_54884966	9	54884966	5.63E-03	GL21_14/E2
S9_52908858	9	52908858	5.64E-03	GL14_ac
S9_53172825	9	53172825	5.64E-03	GL14_14/E2
S9_53172828	9	53172828	5.64E-03	GL14_14/E2
S9_54583679	9	54583679	5.66E-03	GL49_ac
S9_57925883	9	57925883	5.71E-03	GL35_13/E1
S9_519082	9	519082	5.71E-03	GL49_ac
S9_519098	9	519098	5.71E-03	GL49_ac
S9_56507227	9	56507227	5.72E-03	GL21_13/E1
S9_57670282	9	57670282	5.74E-03	GL35_13/E1
S9_53238030	9	53238030	5.75E-03	GL49_13/E1
S9_57545053	9	57545053	5.75E-03	GL7_14/E2
S9_58472760	9	58472760	5.76E-03	GL7_ac
S9_57905432	9	57905432	5.77E-03	GL14_ac
S9_58177614	9	58177614	5.80E-03	GL21_14/E2
S9_57681486	9	57681486	5.80E-03	GL21_13/E1
S9_57780832	9	57780832	5.82E-03	GL7_13/E1
S9_57780833	9	57780833	5.82E-03	GL7_13/E1
S9_57780853	9	57780853	5.82E-03	GL7_13/E1
S9_54080354	9	54080354	5.82E-03	GL21_14/E2
S9_43336956	9	43336956	5.83E-03	GL49_ac
S9_53867526	9	53867526	5.83E-03	GL42_13/E1
S9_53867527	9	53867527	5.83E-03	GL42_13/E1
S9_53867528	9	53867528	5.83E-03	GL42_13/E1
S9_53867529	9	53867529	5.83E-03	GL42_13/E1
S9_59311585	9	59311585	5.85E-03	GL28_13/E1
S9_57925883	9	57925883	5.86E-03	GL28_13/E1
S9_57069088	9	57069088	5.86E-03	GL21_13/E1
S9_57069097	9	57069097	5.86E-03	GL21_13/E1
S9_57069125	9	57069125	5.86E-03	GL21_13/E1
S9_57069131	9	57069131	5.86E-03	GL21_13/E1
S9_56535023	9	56535023	5.89E-03	GL49_14/E2
S9_56535046	9	56535046	5.89E-03	GL49_14/E2
S9_58504227	9	58504227	5.91E-03	GL14_13/E1
S9_56594405	9	56594405	5.92E-03	GL21_13/E1
S9_56594417	9	56594417	5.92E-03	GL21_13/E1
S9_59318053	9	59318053	5.93E-03	GL14_ac
S9_56542015	9	56542015	5.94E-03	GL21_13/E1
S9_56101938	9	56101938	5.94E-03	GL14_14/E2
S9_55883070	9	55883070	6.00E-03	GL7_13/E1
S9_57914531	9	57914531	6.01E-03	GL42_14/E2
S9_56943756	9	56943756	6.01E-03	GL14_ac
S9_56943761	9	56943761	6.01E-03	GL14_ac
S9_56943762	9	56943762	6.01E-03	GL14_ac
S9_56943776	9	56943776	6.01E-03	GL14_ac
S9_56943763	9	56943763	6.01E-03	GL14_ac
S9_56943773	9	56943773	6.01E-03	GL14_ac
S9_56943774	9	56943774	6.01E-03	GL14_ac

SNP	Chromosome	Position	P.value	Trait
S9_56943782	9	56943782	6.01E-03	GL14_ac
S9_57904462	9	57904462	6.02E-03	GL28_ac
S9_58531766	9	58531766	6.02E-03	GL28_14/E2
S9_57811319	9	57811319	6.02E-03	GL14_ac
S9_52908858	9	52908858	6.03E-03	GL35_ac
S9_57139943	9	57139943	6.04E-03	GL28_13/E1
S9_3933616	9	3933616	6.04E-03	GL35_13/E1
S9_56542015	9	56542015	6.07E-03	GL7_ac
S9_57787432	9	57787432	6.09E-03	GL21_13/E1
S9_57139943	9	57139943	6.10E-03	GL49_14/E2
S9_57925883	9	57925883	6.10E-03	GL21_13/E1
S9_54325003	9	54325003	6.12E-03	GL21_13/E1
S9_47241922	9	47241922	6.12E-03	GL14_ac
S9_47241963	9	47241963	6.12E-03	GL14_ac
S9_57812258	9	57812258	6.13E-03	GL28_13/E1
S9_41678371	9	41678371	6.14E-03	GL21_13/E1
S9_41678381	9	41678381	6.14E-03	GL21_13/E1
S9_41678382	9	41678382	6.14E-03	GL21_13/E1
S9_59311770	9	59311770	6.15E-03	GL14_ac
S9_41678371	9	41678371	6.15E-03	GL49_13/E1
S9_41678381	9	41678381	6.15E-03	GL49_13/E1
S9_41678382	9	41678382	6.15E-03	GL49_13/E1
S9_58114502	9	58114502	6.19E-03	GL7_13/E1
S9_57745849	9	57745849	6.20E-03	GL28_13/E1
S9_58177614	9	58177614	6.23E-03	GL35_13/E1
S9_42663390	9	42663390	6.24E-03	GL28_13/E1
S9_58504227	9	58504227	6.26E-03	GL7_13/E1
S9_57911513	9	57911513	6.27E-03	GL49_13/E1
S9_57911514	9	57911514	6.27E-03	GL49_13/E1
S9_57911519	9	57911519	6.27E-03	GL49_13/E1
S9_57911522	9	57911522	6.27E-03	GL49_13/E1
S9_58757504	9	58757504	6.27E-03	GL42_13/E1
S9_57119962	9	57119962	6.28E-03	GL28_14/E2
S9_57119965	9	57119965	6.28E-03	GL28_14/E2
S9_53238030	9	53238030	6.29E-03	GL42_ac
S9_57540793	9	57540793	6.31E-03	GL21_13/E1
S9_57540804	9	57540804	6.31E-03	GL21_13/E1
S9_57545053	9	57545053	6.37E-03	GL14_13/E1
S9_55969226	9	55969226	6.38E-03	GL21_ac
S9_165205	9	165205	6.38E-03	GL7_ac
S9_12066581	9	12066581	6.39E-03	GL14_13/E1
S9_57776578	9	57776578	6.39E-03	GL28_ac
S9_153348	9	153348	6.40E-03	GL28_14/E2
S9_153350	9	153350	6.40E-03	GL28_14/E2
S9_153356	9	153356	6.40E-03	GL28_14/E2
S9_55883070	9	55883070	6.40E-03	GL28_13/E1
S9_57225685	9	57225685	6.44E-03	GL28_13/E1
S9_57670282	9	57670282	6.45E-03	GL28_14/E2
S9_56572848	9	56572848	6.45E-03	GL35_13/E1
S9_56507227	9	56507227	6.46E-03	GL28_13/E1
S9_57780833	9	57780833	6.46E-03	GL21_ac

SNP	Chromosome	Position	P.value	Trait
S9_57780853	9	57780853	6.46E-03	GL21_ac
S9_57780832	9	57780832	6.46E-03	GL21_ac
S9_41678371	9	41678371	6.47E-03	GL28_13/E1
S9_41678381	9	41678381	6.47E-03	GL28_13/E1
S9_41678382	9	41678382	6.47E-03	GL28_13/E1
S9_57811310	9	57811310	6.48E-03	GL35_13/E1
S9_2251160	9	2251160	6.48E-03	GL14_ac
S9_51434058	9	51434058	6.49E-03	GL7_ac
S9_57065277	9	57065277	6.50E-03	GL28_ac
S9_59371922	9	59371922	6.50E-03	GL21_13/E1
S9_53867526	9	53867526	6.52E-03	GL35_13/E1
S9_53867527	9	53867527	6.52E-03	GL35_13/E1
S9_53867528	9	53867528	6.52E-03	GL35_13/E1
S9_53867529	9	53867529	6.52E-03	GL35_13/E1
S9_56524133	9	56524133	6.53E-03	GL42_14/E2
S9_58775824	9	58775824	6.53E-03	GL7_ac
S9_56572848	9	56572848	6.54E-03	GL21_14/E2
S9_58761955	9	58761955	6.54E-03	GL42_13/E1
S9_56943756	9	56943756	6.55E-03	GL7_13/E1
S9_56943761	9	56943761	6.55E-03	GL7_13/E1
S9_56943762	9	56943762	6.55E-03	GL7_13/E1
S9_56943763	9	56943763	6.55E-03	GL7_13/E1
S9_56943773	9	56943773	6.55E-03	GL7_13/E1
S9_56943774	9	56943774	6.55E-03	GL7_13/E1
S9_56943776	9	56943776	6.55E-03	GL7_13/E1
S9_56943782	9	56943782	6.55E-03	GL7_13/E1
S9_58867145	9	58867145	6.57E-03	GL21_13/E1
S9_57529022	9	57529022	6.58E-03	GL14_ac
S9_58541198	9	58541198	6.58E-03	GL7_ac
S9_58114502	9	58114502	6.61E-03	GL49_ac
S9_57065277	9	57065277	6.62E-03	GL21_ac
S9_58114502	9	58114502	6.62E-03	GL42_ac
S9_57541114	9	57541114	6.63E-03	GL21_13/E1
S9_57119962	9	57119962	6.66E-03	GL49_14/E2
S9_57119965	9	57119965	6.66E-03	GL49_14/E2
S9_57811310	9	57811310	6.67E-03	GL35_ac
S9_58177614	9	58177614	6.67E-03	GL49_13/E1
S9_54583679	9	54583679	6.68E-03	GL49_14/E2
S9_513571	9	513571	6.69E-03	GL7_ac
S9_56943758	9	56943758	6.71E-03	GL14_ac
S9_56943756	9	56943756	6.72E-03	GL7_ac
S9_56943761	9	56943761	6.72E-03	GL7_ac
S9_56943762	9	56943762	6.72E-03	GL7_ac
S9_56943776	9	56943776	6.72E-03	GL7_ac
S9_56943763	9	56943763	6.72E-03	GL7_ac
S9_56943773	9	56943773	6.72E-03	GL7_ac
S9_56943774	9	56943774	6.72E-03	GL7_ac
S9_56943782	9	56943782	6.72E-03	GL7_ac
S9_3055421	9	3055421	6.73E-03	GL7_ac
S9_54917701	9	54917701	6.75E-03	GL49_ac
S9_57780833	9	57780833	6.75E-03	GL7_ac

SNP	Chromosome	Position	P.value	Trait
S9_57780853	9	57780853	6.75E-03	GL7_ac
S9_57780832	9	57780832	6.75E-03	GL7_ac
S9_45915919	9	45915919	6.76E-03	GL28_ac
S9_57925883	9	57925883	6.78E-03	GL42_13/E1
S9_55067227	9	55067227	6.78E-03	GL49_ac
S9_58684354	9	58684354	6.80E-03	GL7_ac
S9_57904462	9	57904462	6.81E-03	GL42_13/E1
S9_57681486	9	57681486	6.82E-03	GL28_13/E1
S9_2251160	9	2251160	6.82E-03	GL21_ac
S9_59299818	9	59299818	6.83E-03	GL14_14/E2
S9_57833859	9	57833859	6.84E-03	GL35_13/E1
S9_57833860	9	57833860	6.84E-03	GL35_13/E1
S9_58114502	9	58114502	6.87E-03	GL21_14/E2
S9_45915919	9	45915919	6.87E-03	GL21_14/E2
S9_1203414	9	1203414	6.88E-03	GL35_14/E2
S9_57529022	9	57529022	6.90E-03	GL21_14/E2
S9_57780833	9	57780833	6.91E-03	GL35_ac
S9_57780853	9	57780853	6.91E-03	GL35_ac
S9_57780832	9	57780832	6.91E-03	GL35_ac
S9_59207864	9	59207864	6.92E-03	GL14_ac
S9_4373590	9	4373590	6.95E-03	GL7_13/E1
S9_52923075	9	52923075	6.95E-03	GL35_13/E1
S9_2251160	9	2251160	6.95E-03	GL28_ac
S9_2238350	9	2238350	6.97E-03	GL14_ac
S9_44446459	9	44446459	6.97E-03	GL42_13/E1
S9_57529022	9	57529022	6.98E-03	GL21_13/E1
S9_58757504	9	58757504	6.99E-03	GL14_ac
S9_56945723	9	56945723	7.03E-03	GL28_13/E1
S9_56945733	9	56945733	7.03E-03	GL28_13/E1
S9_57787432	9	57787432	7.03E-03	GL7_13/E1
S9_56943758	9	56943758	7.03E-03	GL7_ac
S9_4652966	9	4652966	7.05E-03	GL14_14/E2
S9_53164772	9	53164772	7.07E-03	GL49_13/E1
S9_56115202	9	56115202	7.08E-03	GL21_13/E1
S9_57065277	9	57065277	7.09E-03	GL35_13/E1
S9_57911518	9	57911518	7.11E-03	GL49_13/E1
S9_56943758	9	56943758	7.11E-03	GL7_13/E1
S9_58472760	9	58472760	7.11E-03	GL14_ac
S9_56535023	9	56535023	7.13E-03	GL14_14/E2
S9_56535046	9	56535046	7.13E-03	GL14_14/E2
S9_58177614	9	58177614	7.15E-03	GL14_14/E2
S9_59312050	9	59312050	7.15E-03	GL35_ac
S9_58291473	9	58291473	7.15E-03	GL14_ac
S9_57541114	9	57541114	7.17E-03	GL42_14/E2
S9_2233856	9	2233856	7.18E-03	GL7_13/E1
S9_57905432	9	57905432	7.19E-03	GL35_13/E1
S9_53897971	9	53897971	7.19E-03	GL14_13/E1
S9_54370459	9	54370459	7.20E-03	GL42_ac
S9_58332115	9	58332115	7.24E-03	GL28_ac
S9_57065264	9	57065264	7.25E-03	GL35_ac
S9_58291473	9	58291473	7.25E-03	GL21_ac

SNP	Chromosome	Position	P.value	Trait
S9_57875319	9	57875319	7.26E-03	GL28_ac
S9_59371922	9	59371922	7.26E-03	GL42_14/E2
S9_53238030	9	53238030	7.29E-03	GL21_13/E1
S9_57529022	9	57529022	7.30E-03	GL14_14/E2
S9_58702178	9	58702178	7.31E-03	GL7_ac
S9_57139943	9	57139943	7.33E-03	GL35_13/E1
S9_58761955	9	58761955	7.33E-03	GL49_13/E1
S9_375417	9	375417	7.34E-03	GL7_ac
S9_375423	9	375423	7.34E-03	GL7_ac
S9_375406	9	375406	7.34E-03	GL7_ac
S9_58684354	9	58684354	7.34E-03	GL28_ac
S9_57657251	9	57657251	7.35E-03	GL21_14/E2
S9_2238350	9	2238350	7.35E-03	GL7_ac
S9_47241922	9	47241922	7.36E-03	GL35_ac
S9_47241963	9	47241963	7.36E-03	GL35_ac
S9_519082	9	519082	7.36E-03	GL14_ac
S9_519098	9	519098	7.36E-03	GL14_ac
S9_55492025	9	55492025	7.41E-03	GL7_ac
S9_55492019	9	55492019	7.41E-03	GL7_ac
S9_55492020	9	55492020	7.41E-03	GL7_ac
S9_58702178	9	58702178	7.41E-03	GL14_14/E2
S9_52923067	9	52923067	7.42E-03	GL35_13/E1
S9_59291140	9	59291140	7.42E-03	GL28_14/E2
S9_59371922	9	59371922	7.42E-03	GL42_13/E1
S9_58114502	9	58114502	7.43E-03	GL49_14/E2
S9_2941419	9	2941419	7.47E-03	GL49_13/E1
S9_58114502	9	58114502	7.47E-03	GL14_ac
S9_53527986	9	53527986	7.48E-03	GL21_14/E2
S9_56101938	9	56101938	7.49E-03	GL42_ac
S9_57947937	9	57947937	7.54E-03	GL21_14/E2
S9_57540793	9	57540793	7.55E-03	GL49_14/E2
S9_57540804	9	57540804	7.55E-03	GL49_14/E2
S9_57905432	9	57905432	7.56E-03	GL35_ac
S9_58634599	9	58634599	7.56E-03	GL21_ac
S9_58634609	9	58634609	7.56E-03	GL21_ac
S9_45915919	9	45915919	7.57E-03	GL28_14/E2
S9_47145952	9	47145952	7.60E-03	GL42_ac
S9_45915919	9	45915919	7.62E-03	GL21_ac
S9_58332115	9	58332115	7.62E-03	GL21_ac
S9_54080354	9	54080354	7.63E-03	GL42_ac
S9_59159043	9	59159043	7.65E-03	GL21_14/E2
S9_58634599	9	58634599	7.65E-03	GL28_13/E1
S9_58634609	9	58634609	7.65E-03	GL28_13/E1
S9_59318053	9	59318053	7.66E-03	GL7_13/E1
S9_55067209	9	55067209	7.67E-03	GL35_ac
S9_55067231	9	55067231	7.67E-03	GL35_ac
S9_55067234	9	55067234	7.67E-03	GL35_ac
S9_57914426	9	57914426	7.67E-03	GL28_13/E1
S9_44414073	9	44414073	7.69E-03	GL21_14/E2
S9_53818841	9	53818841	7.71E-03	GL42_14/E2
S9_57123473	9	57123473	7.72E-03	GL49_13/E1

SNP	Chromosome	Position	P.value	Trait
S9_57119962	9	57119962	7.72E-03	GL21_14/E2
S9_57119965	9	57119965	7.72E-03	GL21_14/E2
S9_57811319	9	57811319	7.73E-03	GL35_13/E1
S9_57812258	9	57812258	7.73E-03	GL21_13/E1
S9_2546489	9	2546489	7.73E-03	GL35_ac
S9_58684354	9	58684354	7.73E-03	GL21_13/E1
S9_58508533	9	58508533	7.75E-03	GL28_ac
S9_40815469	9	40815469	7.76E-03	GL49_14/E2
S9_42663390	9	42663390	7.78E-03	GL21_13/E1
S9_58541198	9	58541198	7.81E-03	GL42_13/E1
S9_57792165	9	57792165	7.82E-03	GL28_13/E1
S9_57792168	9	57792168	7.82E-03	GL28_13/E1
S9_57792171	9	57792171	7.82E-03	GL28_13/E1
S9_59291140	9	59291140	7.85E-03	GL7_14/E2
S9_58504227	9	58504227	7.86E-03	GL49_13/E1
S9_59291140	9	59291140	7.86E-03	GL7_ac
S9_58127631	9	58127631	7.88E-03	GL21_13/E1
S9_57875319	9	57875319	7.88E-03	GL21_ac
S9_58504227	9	58504227	7.88E-03	GL42_ac
S9_54857339	9	54857339	7.89E-03	GL42_ac
S9_56572848	9	56572848	7.89E-03	GL49_14/E2
S9_58775824	9	58775824	7.89E-03	GL7_13/E1
S9_57051565	9	57051565	7.91E-03	GL21_13/E1
S9_54917701	9	54917701	7.92E-03	GL42_ac
S9_58634599	9	58634599	7.92E-03	GL21_13/E1
S9_58634609	9	58634609	7.92E-03	GL21_13/E1
S9_53527889	9	53527889	7.95E-03	GL28_14/E2
S9_57925883	9	57925883	7.95E-03	GL49_ac
S9_51830251	9	51830251	7.98E-03	GL14_13/E1
S9_51830298	9	51830298	7.98E-03	GL14_13/E1
S9_57904462	9	57904462	7.99E-03	GL14_13/E1
S9_55969226	9	55969226	8.01E-03	GL28_ac
S9_3055421	9	3055421	8.03E-03	GL21_ac
S9_2754294	9	2754294	8.03E-03	GL14_13/E1
S9_57780833	9	57780833	8.04E-03	GL42_ac
S9_57780853	9	57780853	8.04E-03	GL42_ac
S9_57780832	9	57780832	8.04E-03	GL42_ac
S9_57225685	9	57225685	8.04E-03	GL21_13/E1
S9_57216854	9	57216854	8.07E-03	GL28_14/E2
S9_57069120	9	57069120	8.08E-03	GL21_13/E1
S9_59207864	9	59207864	8.08E-03	GL35_13/E1
S9_57925883	9	57925883	8.13E-03	GL21_ac
S9_58531766	9	58531766	8.14E-03	GL14_ac
S9_55969226	9	55969226	8.17E-03	GL21_13/E1
S9_47050016	9	47050016	8.21E-03	GL42_ac
S9_153373	9	153373	8.21E-03	GL49_ac
S9_57051565	9	57051565	8.25E-03	GL28_13/E1
S9_58949348	9	58949348	8.25E-03	GL49_13/E1
S9_58949348	9	58949348	8.25E-03	GL49_13/E1
S9_56101938	9	56101938	8.25E-03	GL35_14/E2
S9_58775824	9	58775824	8.26E-03	GL14_ac

SNP	Chromosome	Position	P.value	Trait
S9_56594405	9	56594405	8.27E-03	GL14_14/E2
S9_56594417	9	56594417	8.27E-03	GL14_14/E2
S9_40815469	9	40815469	8.28E-03	GL14_14/E2
S9_57540793	9	57540793	8.29E-03	GL28_13/E1
S9_57540804	9	57540804	8.29E-03	GL28_13/E1
S9_59311585	9	59311585	8.31E-03	GL7_14/E2
S9_55031132	9	55031132	8.31E-03	GL49_ac
S9_59322668	9	59322668	8.32E-03	GL14_ac
S9_55121776	9	55121776	8.34E-03	GL42_14/E2
S9_10729664	9	10729664	8.35E-03	GL42_14/E2
S9_57904462	9	57904462	8.35E-03	GL7_ac
S9_59311770	9	59311770	8.38E-03	GL35_ac
S9_59311770	9	59311770	8.38E-03	GL7_ac
S9_56943756	9	56943756	8.40E-03	GL28_ac
S9_56943761	9	56943761	8.40E-03	GL28_ac
S9_56943762	9	56943762	8.40E-03	GL28_ac
S9_56943776	9	56943776	8.40E-03	GL28_ac
S9_56943763	9	56943763	8.40E-03	GL28_ac
S9_56943773	9	56943773	8.40E-03	GL28_ac
S9_56943774	9	56943774	8.40E-03	GL28_ac
S9_56943782	9	56943782	8.40E-03	GL28_ac
S9_57752751	9	57752751	8.44E-03	GL28_14/E2
S9_57752787	9	57752787	8.44E-03	GL28_14/E2
S9_55067203	9	55067203	8.44E-03	GL49_ac
S9_56530403	9	56530403	8.44E-03	GL21_13/E1
S9_57529022	9	57529022	8.47E-03	GL7_ac
S9_57247235	9	57247235	8.48E-03	GL14_14/E2
S9_57804067	9	57804067	8.48E-03	GL35_ac
S9_236344	9	236344	8.48E-03	GL49_ac
S9_53899689	9	53899689	8.49E-03	GL42_14/E2
S9_58332115	9	58332115	8.52E-03	GL42_ac
S9_57947937	9	57947937	8.54E-03	GL49_14/E2
S9_58508533	9	58508533	8.55E-03	GL28_13/E1
S9_56520725	9	56520725	8.56E-03	GL49_ac
S9_55551966	9	55551966	8.57E-03	GL21_13/E1
S9_57925883	9	57925883	8.58E-03	GL28_ac
S9_40815469	9	40815469	8.58E-03	GL49_ac
S9_57545053	9	57545053	8.59E-03	GL42_ac
S9_53529462	9	53529462	8.63E-03	GL42_ac
S9_54973417	9	54973417	8.64E-03	GL49_ac
S9_57776578	9	57776578	8.65E-03	GL35_ac
S9_52939504	9	52939504	8.66E-03	GL42_14/E2
S9_57811310	9	57811310	8.68E-03	GL49_ac
S9_57670672	9	57670672	8.69E-03	GL7_14/E2
S9_57051565	9	57051565	8.71E-03	GL28_14/E2
S9_40815469	9	40815469	8.71E-03	GL42_14/E2
S9_57875319	9	57875319	8.72E-03	GL49_13/E1
S9_58684354	9	58684354	8.74E-03	GL28_13/E1
S9_56943758	9	56943758	8.74E-03	GL28_ac
S9_41678371	9	41678371	8.75E-03	GL42_ac
S9_41678381	9	41678381	8.75E-03	GL42_ac

SNP	Chromosome	Position	P.value	Trait
S9_41678382	9	41678382	8.75E-03	GL42_ac
S9_55004994	9	55004994	8.76E-03	GL21_14/E2
S9_43445226	9	43445226	8.77E-03	GL7_13/E1
S9_58670296	9	58670296	8.78E-03	GL28_14/E2
S9_10724823	9	10724823	8.80E-03	GL14_14/E2
S9_47241922	9	47241922	8.84E-03	GL42_13/E1
S9_47241963	9	47241963	8.84E-03	GL42_13/E1
S9_55869446	9	55869446	8.84E-03	GL42_13/E1
S9_57069088	9	57069088	8.87E-03	GL28_13/E1
S9_57069097	9	57069097	8.87E-03	GL28_13/E1
S9_57069125	9	57069125	8.87E-03	GL28_13/E1
S9_57069131	9	57069131	8.87E-03	GL28_13/E1
S9_57756001	9	57756001	8.87E-03	GL28_14/E2
S9_57756001	9	57756001	8.87E-03	GL28_14/E2
S9_58684354	9	58684354	8.88E-03	GL35_13/E1
S9_57069120	9	57069120	8.89E-03	GL28_13/E1
S9_58177614	9	58177614	8.89E-03	GL28_ac
S9_53867527	9	53867527	8.90E-03	GL14_ac
S9_53867526	9	53867526	8.90E-03	GL14_ac
S9_53867528	9	53867528	8.90E-03	GL14_ac
S9_53867529	9	53867529	8.90E-03	GL14_ac
S9_57123473	9	57123473	8.91E-03	GL42_13/E1
S9_58702178	9	58702178	8.93E-03	GL21_14/E2
S9_57905432	9	57905432	8.94E-03	GL42_13/E1
S9_56535023	9	56535023	8.96E-03	GL14_ac
S9_56535046	9	56535046	8.96E-03	GL14_ac
S9_58775824	9	58775824	8.96E-03	GL28_13/E1
S9_58684354	9	58684354	8.97E-03	GL21_ac
S9_44414073	9	44414073	8.97E-03	GL14_14/E2
S9_57657884	9	57657884	8.97E-03	GL28_14/E2
S9_56945723	9	56945723	8.99E-03	GL7_ac
S9_56945733	9	56945733	8.99E-03	GL7_ac
S9_57792165	9	57792165	9.01E-03	GL14_ac
S9_57792168	9	57792168	9.01E-03	GL14_ac
S9_57792171	9	57792171	9.01E-03	GL14_ac
S9_59312050	9	59312050	9.02E-03	GL42_ac
S9_57051565	9	57051565	9.02E-03	GL42_13/E1
S9_55566640	9	55566640	9.04E-03	GL42_13/E1
S9_58508533	9	58508533	9.04E-03	GL14_ac
S9_58177614	9	58177614	9.05E-03	GL35_ac
S9_54370459	9	54370459	9.05E-03	GL49_13/E1
S9_41430950	9	41430950	9.05E-03	GL49_14/E2
S9_51434058	9	51434058	9.08E-03	GL35_13/E1
S9_54908173	9	54908173	9.11E-03	GL35_13/E1
S9_54908187	9	54908187	9.11E-03	GL35_13/E1
S9_54908190	9	54908190	9.11E-03	GL35_13/E1
S9_54908191	9	54908191	9.11E-03	GL35_13/E1
S9_54908192	9	54908192	9.11E-03	GL35_13/E1
S9_54908193	9	54908193	9.11E-03	GL35_13/E1
S9_57905432	9	57905432	9.12E-03	GL42_ac
S9_57925883	9	57925883	9.13E-03	GL14_ac

SNP	Chromosome	Position	P.value	Trait
S9_57139944	9	57139944	9.15E-03	GL21_13/E1
S9_41543597	9	41543597	9.21E-03	GL42_14/E2
S9_57119962	9	57119962	9.22E-03	GL28_13/E1
S9_57119965	9	57119965	9.22E-03	GL28_13/E1
S9_57905432	9	57905432	9.22E-03	GL7_ac
S9_58727975	9	58727975	9.23E-03	GL28_13/E1
S9_58727998	9	58727998	9.23E-03	GL28_13/E1
S9_58728008	9	58728008	9.23E-03	GL28_13/E1
S9_58841852	9	58841852	9.30E-03	GL14_ac
S9_56535023	9	56535023	9.33E-03	GL28_ac
S9_56535046	9	56535046	9.33E-03	GL28_ac
S9_54857339	9	54857339	9.33E-03	GL21_14/E2
S9_56524133	9	56524133	9.33E-03	GL14_ac
S9_51434058	9	51434058	9.35E-03	GL42_13/E1
S9_59207864	9	59207864	9.37E-03	GL14_13/E1
S9_59207864	9	59207864	9.39E-03	GL7_13/E1
S9_41678371	9	41678371	9.39E-03	GL7_ac
S9_41678381	9	41678381	9.39E-03	GL7_ac
S9_41678382	9	41678382	9.39E-03	GL7_ac
S9_59312050	9	59312050	9.41E-03	GL21_14/E2
S9_55004994	9	55004994	9.41E-03	GL21_ac
S9_57804067	9	57804067	9.43E-03	GL7_13/E1
S9_56520725	9	56520725	9.44E-03	GL49_14/E2
S9_58841852	9	58841852	9.44E-03	GL21_14/E2
S9_51434058	9	51434058	9.45E-03	GL21_ac
S9_6802694	9	6802694	9.45E-03	GL35_ac
S9_6802790	9	6802790	9.45E-03	GL35_ac
S9_6802694	9	6802694	9.45E-03	GL35_ac
S9_6802694	9	6802694	9.45E-03	GL35_ac
S9_6802788	9	6802788	9.45E-03	GL35_ac
S9_6802789	9	6802789	9.45E-03	GL35_ac
S9_58775824	9	58775824	9.48E-03	GL14_13/E1
S9_2886888	9	2886888	9.48E-03	GL42_14/E2
S9_2886913	9	2886913	9.48E-03	GL42_14/E2
S9_57781398	9	57781398	9.49E-03	GL14_ac
S9_59299818	9	59299818	9.51E-03	GL35_ac
S9_52923065	9	52923065	9.51E-03	GL35_13/E1
S9_52923066	9	52923066	9.51E-03	GL35_13/E1
S9_52923069	9	52923069	9.51E-03	GL35_13/E1
S9_52923070	9	52923070	9.51E-03	GL35_13/E1
S9_52923072	9	52923072	9.51E-03	GL35_13/E1
S9_59159043	9	59159043	9.53E-03	GL14_14/E2
S9_2233856	9	2233856	9.53E-03	GL49_14/E2
S9_57216854	9	57216854	9.53E-03	GL21_13/E1
S9_57787432	9	57787432	9.55E-03	GL35_13/E1
S9_519136	9	519136	9.55E-03	GL28_ac
S9_58702178	9	58702178	9.57E-03	GL14_ac
S9_3616579	9	3616579	9.57E-03	GL42_14/E2
S9_59318053	9	59318053	9.58E-03	GL14_13/E1
S9_2239828	9	2239828	9.59E-03	GL7_ac
S9_57911513	9	57911513	9.60E-03	GL28_ac

SNP	Chromosome	Position	P.value	Trait
S9_57911514	9	57911514	9.60E-03	GL28_ac
S9_57911519	9	57911519	9.60E-03	GL28_ac
S9_57911522	9	57911522	9.60E-03	GL28_ac
S9_57914426	9	57914426	9.60E-03	GL14_14/E2
S9_53238030	9	53238030	9.62E-03	GL28_ac
S9_57681486	9	57681486	9.63E-03	GL21_14/E2
S9_54583637	9	54583637	9.63E-03	GL49_14/E2
S9_57947937	9	57947937	9.64E-03	GL42_13/E1
S9_2546489	9	2546489	9.67E-03	GL21_ac
S9_55004994	9	55004994	9.69E-03	GL35_ac
S9_3933616	9	3933616	9.71E-03	GL7_ac
S9_58634599	9	58634599	9.72E-03	GL28_ac
S9_58634609	9	58634609	9.72E-03	GL28_ac
S9_57804067	9	57804067	9.73E-03	GL21_ac
S9_58748326	9	58748326	9.74E-03	GL28_ac
S9_58748351	9	58748351	9.77E-03	GL21_ac
S9_58748316	9	58748316	9.77E-03	GL21_ac
S9_58748317	9	58748317	9.77E-03	GL21_ac
S9_58748322	9	58748322	9.77E-03	GL21_ac
S9_54370459	9	54370459	9.77E-03	GL35_ac
S9_57780833	9	57780833	9.83E-03	GL14_ac
S9_57780853	9	57780853	9.83E-03	GL14_ac
S9_57780832	9	57780832	9.83E-03	GL14_ac
S9_58748351	9	58748351	9.84E-03	GL28_ac
S9_58748316	9	58748316	9.84E-03	GL28_ac
S9_58748317	9	58748317	9.84E-03	GL28_ac
S9_58748322	9	58748322	9.84E-03	GL28_ac
S9_6802694	9	6802694	9.85E-03	GL14_13/E1
S9_6802694	9	6802694	9.85E-03	GL14_13/E1
S9_6802694	9	6802694	9.85E-03	GL14_13/E1
S9_6802788	9	6802788	9.85E-03	GL14_13/E1
S9_6802789	9	6802789	9.85E-03	GL14_13/E1
S9_6802790	9	6802790	9.85E-03	GL14_13/E1
S9_51890203	9	51890203	9.85E-03	GL14_13/E1
S9_2239826	9	2239826	9.86E-03	GL7_ac
S9_2239848	9	2239848	9.86E-03	GL7_ac
S9_2239829	9	2239829	9.86E-03	GL7_ac
S9_53930959	9	53930959	9.88E-03	GL42_13/E1
S9_53930960	9	53930960	9.88E-03	GL42_13/E1
S9_53930966	9	53930966	9.88E-03	GL42_13/E1
S9_56594405	9	56594405	9.91E-03	GL35_ac
S9_56594417	9	56594417	9.91E-03	GL35_ac
S9_41614962	9	41614962	9.97E-03	GL21_13/E1
S9_57273252	9	57273252	9.99E-03	GL49_14/E2
S9_54325003	9	54325003	9.99E-03	GL42_14/E2
S9_519082	9	519082	9.99E-03	GL28_ac
S9_519098	9	519098	9.99E-03	GL28_ac
S10_48512575	10	48512575	3.00E-04	GL42_14/E2
S10_8873222	10	8873222	3.65E-04	GL14_14/E2
S10_8873243	10	8873243	3.65E-04	GL14_14/E2
S10_49536872	10	49536872	3.78E-04	GL49_13/E1

SNP	Chromosome	Position	P.value	Trait
S10_5558734	10	5558734	3.84E-04	GL14_ac
S10_49536872	10	49536872	4.08E-04	GL14_ac
S10_6049695	10	6049695	4.45E-04	GL14_ac
S10_50865236	10	50865236	5.02E-04	GL14_14/E2
S10_8873222	10	8873222	5.41E-04	GL21_14/E2
S10_8873243	10	8873243	5.41E-04	GL21_14/E2
S10_5558734	10	5558734	5.90E-04	GL21_ac
S10_6132423	10	6132423	6.66E-04	GL42_ac
S10_50865236	10	50865236	7.08E-04	GL28_14/E2
S10_8873501	10	8873501	7.87E-04	GL35_14/E2
S10_7917302	10	7917302	7.93E-04	GL21_ac
S10_48512624	10	48512624	8.12E-04	GL42_14/E2
S10_6132423	10	6132423	8.54E-04	GL49_ac
S10_5558734	10	5558734	9.38E-04	GL28_13/E1
S10_6132413	10	6132413	9.42E-04	GL42_ac
S10_57663572	10	57663572	9.64E-04	GL14_13/E1
S10_6132413	10	6132413	1.04E-03	GL49_ac
S10_54897091	10	54897091	1.11E-03	GL35_ac
S10_9617926	10	9617926	1.12E-03	GL28_14/E2
S10_55505116	10	55505116	1.12E-03	GL7_14/E2
S10_574319	10	574319	1.13E-03	GL49_ac
S10_50405318	10	50405318	1.14E-03	GL21_14/E2
S10_52971581	10	52971581	1.16E-03	GL14_14/E2
S10_52971607	10	52971607	1.16E-03	GL14_14/E2
S10_52971592	10	52971592	1.16E-03	GL14_14/E2
S10_5558734	10	5558734	1.18E-03	GL28_ac
S10_5558734	10	5558734	1.20E-03	GL21_13/E1
S10_44910447	10	44910447	1.24E-03	GL49_14/E2
S10_48905718	10	48905718	1.33E-03	GL21_14/E2
S10_6808270	10	6808270	1.36E-03	GL35_14/E2
S10_2716110	10	2716110	1.39E-03	GL7_14/E2
S10_48929913	10	48929913	1.39E-03	GL14_14/E2
S10_6049695	10	6049695	1.41E-03	GL21_ac
S10_4310973	10	4310973	1.41E-03	GL28_14/E2
S10_48905718	10	48905718	1.42E-03	GL14_14/E2
S10_8873222	10	8873222	1.42E-03	GL28_14/E2
S10_8873243	10	8873243	1.42E-03	GL28_14/E2
S10_6132423	10	6132423	1.60E-03	GL21_ac
S10_9617926	10	9617926	1.63E-03	GL21_14/E2
S10_54919953	10	54919953	1.70E-03	GL14_ac
S10_42917852	10	42917852	1.77E-03	GL14_ac
S10_574319	10	574319	1.85E-03	GL49_14/E2
S10_13677145	10	13677145	1.88E-03	GL14_14/E2
S10_5558734	10	5558734	1.88E-03	GL14_13/E1
S10_58028206	10	58028206	1.90E-03	GL7_14/E2
S10_5558734	10	5558734	1.92E-03	GL7_13/E1
S10_6132423	10	6132423	1.99E-03	GL14_ac
S10_7917302	10	7917302	2.04E-03	GL28_ac
S10_60973291	10	60973291	2.06E-03	GL42_13/E1
S10_57663572	10	57663572	2.16E-03	GL7_13/E1
S10_4233975	10	4233975	2.18E-03	GL14_ac

SNP	Chromosome	Position	P.value	Trait
S10_56784622	10	56784622	2.23E-03	GL7_14/E2
S10_574319	10	574319	2.26E-03	GL42_ac
S10_58276305	10	58276305	2.27E-03	GL7_14/E2
S10_6132423	10	6132423	2.28E-03	GL28_ac
S10_54915154	10	54915154	2.35E-03	GL28_14/E2
S10_54915161	10	54915161	2.35E-03	GL28_14/E2
S10_54915163	10	54915163	2.35E-03	GL28_14/E2
S10_60973291	10	60973291	2.48E-03	GL35_13/E1
S10_13483803	10	13483803	2.52E-03	GL42_14/E2
S10_16865408	10	16865408	2.57E-03	GL49_13/E1
S10_16865449	10	16865449	2.57E-03	GL49_13/E1
S10_43137265	10	43137265	2.66E-03	GL42_ac
S10_53972403	10	53972403	2.66E-03	GL42_13/E1
S10_4233975	10	4233975	2.67E-03	GL7_ac
S10_50865236	10	50865236	2.69E-03	GL21_14/E2
S10_53690458	10	53690458	2.70E-03	GL42_13/E1
S10_4231550	10	4231550	2.88E-03	GL35_ac
S10_57510942	10	57510942	3.00E-03	GL7_13/E1
S10_8873501	10	8873501	3.00E-03	GL7_ac
S10_57510942	10	57510942	3.01E-03	GL14_13/E1
S10_5558734	10	5558734	3.02E-03	GL7_ac
S10_44899734	10	44899734	3.08E-03	GL49_13/E1
S10_5210528	10	5210528	3.09E-03	GL7_13/E1
S10_46606416	10	46606416	3.13E-03	GL49_14/E2
S10_48905718	10	48905718	3.20E-03	GL35_14/E2
S10_6808270	10	6808270	3.21E-03	GL49_14/E2
S10_55953518	10	55953518	3.22E-03	GL49_13/E1
S10_55953554	10	55953554	3.22E-03	GL49_13/E1
S10_41032913	10	41032913	3.22E-03	GL49_ac
S10_4233975	10	4233975	3.23E-03	GL21_ac
S10_50725358	10	50725358	3.26E-03	GL21_14/E2
S10_54841288	10	54841288	3.27E-03	GL21_14/E2
S10_54841304	10	54841304	3.27E-03	GL21_14/E2
S10_54841321	10	54841321	3.27E-03	GL21_14/E2
S10_56475813	10	56475813	3.29E-03	GL14_13/E1
S10_9572293	10	9572293	3.29E-03	GL35_14/E2
S10_9572249	10	9572249	3.29E-03	GL35_14/E2
S10_8873501	10	8873501	3.33E-03	GL49_14/E2
S10_16865408	10	16865408	3.34E-03	GL7_13/E1
S10_16865449	10	16865449	3.34E-03	GL7_13/E1
S10_5210528	10	5210528	3.35E-03	GL14_13/E1
S10_54841288	10	54841288	3.35E-03	GL14_14/E2
S10_54841304	10	54841304	3.35E-03	GL14_14/E2
S10_54841321	10	54841321	3.35E-03	GL14_14/E2
S10_56428954	10	56428954	3.35E-03	GL14_13/E1
S10_56428957	10	56428957	3.35E-03	GL14_13/E1
S10_7917302	10	7917302	3.36E-03	GL35_ac
S10_15185284	10	15185284	3.38E-03	GL14_14/E2
S10_54841484	10	54841484	3.39E-03	GL49_ac
S10_54841489	10	54841489	3.39E-03	GL49_ac
S10_56475813	10	56475813	3.43E-03	GL21_13/E1

SNP	Chromosome	Position	P.value	Trait
S10_56784622	10	56784622	3.48E-03	GL14_ac
S10_6132413	10	6132413	3.53E-03	GL21_ac
S10_43586473	10	43586473	3.60E-03	GL49_14/E2
S10_7917302	10	7917302	3.63E-03	GL21_13/E1
S10_53317190	10	53317190	3.64E-03	GL14_13/E1
S10_49536872	10	49536872	3.68E-03	GL28_ac
S10_9577350	10	9577350	3.68E-03	GL28_14/E2
S10_52971581	10	52971581	3.70E-03	GL21_14/E2
S10_52971607	10	52971607	3.70E-03	GL21_14/E2
S10_52971592	10	52971592	3.70E-03	GL21_14/E2
S10_8753539	10	8753539	3.72E-03	GL14_14/E2
S10_49536872	10	49536872	3.74E-03	GL14_13/E1
S10_56428954	10	56428954	3.83E-03	GL21_13/E1
S10_56428957	10	56428957	3.83E-03	GL21_13/E1
S10_57663572	10	57663572	3.85E-03	GL7_ac
S10_9577350	10	9577350	3.90E-03	GL28_14/E2
S10_6132423	10	6132423	4.00E-03	GL35_ac
S10_60973291	10	60973291	4.03E-03	GL28_13/E1
S10_7697840	10	7697840	4.06E-03	GL28_13/E1
S10_7697850	10	7697850	4.06E-03	GL28_13/E1
S10_7697851	10	7697851	4.06E-03	GL28_13/E1
S10_7697852	10	7697852	4.06E-03	GL28_13/E1
S10_7697853	10	7697853	4.06E-03	GL28_13/E1
S10_7697856	10	7697856	4.06E-03	GL28_13/E1
S10_7697858	10	7697858	4.06E-03	GL28_13/E1
S10_6132413	10	6132413	4.11E-03	GL14_ac
S10_42944613	10	42944613	4.19E-03	GL28_13/E1
S10_60058846	10	60058846	4.25E-03	GL49_14/E2
S10_60058874	10	60058874	4.25E-03	GL49_14/E2
S10_60058876	10	60058876	4.25E-03	GL49_14/E2
S10_49536872	10	49536872	4.28E-03	GL49_ac
S10_6132413	10	6132413	4.32E-03	GL28_ac
S10_18003749	10	18003749	4.32E-03	GL14_14/E2
S10_18003751	10	18003751	4.32E-03	GL14_14/E2
S10_50405318	10	50405318	4.35E-03	GL28_14/E2
S10_54897091	10	54897091	4.36E-03	GL7_ac
S10_5210528	10	5210528	4.36E-03	GL21_13/E1
S10_56428954	10	56428954	4.46E-03	GL28_13/E1
S10_56428957	10	56428957	4.46E-03	GL28_13/E1
S10_7138802	10	7138802	4.48E-03	GL35_14/E2
S10_17330914	10	17330914	4.49E-03	GL35_14/E2
S10_38695561	10	38695561	4.53E-03	GL35_ac
S10_55505116	10	55505116	4.73E-03	GL49_14/E2
S10_50945046	10	50945046	4.74E-03	GL21_14/E2
S10_10215201	10	10215201	4.78E-03	GL7_14/E2
S10_7893655	10	7893655	4.80E-03	GL35_14/E2
S10_60973291	10	60973291	4.83E-03	GL21_13/E1
S10_44910447	10	44910447	4.86E-03	GL49_ac
S10_4309617	10	4309617	4.90E-03	GL14_ac
S10_4309625	10	4309625	4.90E-03	GL14_ac
S10_4310973	10	4310973	4.95E-03	GL21_14/E2

SNP	Chromosome	Position	P.value	Trait
S10_4231550	10	4231550	4.98E-03	GL35_13/E1
S10_6100258	10	6100258	5.04E-03	GL7_13/E1
S10_574319	10	574319	5.06E-03	GL42_14/E2
S10_7934181	10	7934181	5.08E-03	GL49_ac
S10_48868738	10	48868738	5.10E-03	GL14_ac
S10_54897091	10	54897091	5.10E-03	GL42_ac
S10_13677145	10	13677145	5.12E-03	GL35_14/E2
S10_6808270	10	6808270	5.12E-03	GL7_ac
S10_58288752	10	58288752	5.19E-03	GL7_14/E2
S10_7434473	10	7434473	5.21E-03	GL49_ac
S10_46573203	10	46573203	5.23E-03	GL28_14/E2
S10_53317190	10	53317190	5.29E-03	GL7_13/E1
S10_50945046	10	50945046	5.42E-03	GL28_14/E2
S10_17330914	10	17330914	5.44E-03	GL42_14/E2
S10_42917852	10	42917852	5.44E-03	GL21_ac
S10_58566780	10	58566780	5.45E-03	GL14_13/E1
S10_7917302	10	7917302	5.49E-03	GL35_13/E1
S10_56508509	10	56508509	5.51E-03	GL7_14/E2
S10_56508557	10	56508557	5.51E-03	GL7_14/E2
S10_4233975	10	4233975	5.54E-03	GL35_ac
S10_56784622	10	56784622	5.55E-03	GL14_14/E2
S10_6049695	10	6049695	5.59E-03	GL14_13/E1
S10_49536872	10	49536872	5.61E-03	GL21_ac
S10_5217988	10	5217988	5.86E-03	GL21_14/E2
S10_41032913	10	41032913	5.87E-03	GL28_ac
S10_58599753	10	58599753	5.94E-03	GL7_14/E2
S10_54841484	10	54841484	5.95E-03	GL49_13/E1
S10_54841489	10	54841489	5.95E-03	GL49_13/E1
S10_6132413	10	6132413	5.99E-03	GL35_ac
S10_42944613	10	42944613	5.99E-03	GL35_13/E1
S10_60654069	10	60654069	6.00E-03	GL42_13/E1
S10_13178326	10	13178326	6.01E-03	GL21_14/E2
S10_56475813	10	56475813	6.06E-03	GL42_13/E1
S10_4309617	10	4309617	6.08E-03	GL21_ac
S10_4309625	10	4309625	6.08E-03	GL21_ac
S10_54897091	10	54897091	6.13E-03	GL21_ac
S10_60058846	10	60058846	6.17E-03	GL49_ac
S10_60058874	10	60058874	6.17E-03	GL49_ac
S10_60058876	10	60058876	6.17E-03	GL49_ac
S10_4233975	10	4233975	6.21E-03	GL28_13/E1
S10_4183532	10	4183532	6.23E-03	GL28_14/E2
S10_58028206	10	58028206	6.25E-03	GL14_13/E1
S10_6808270	10	6808270	6.27E-03	GL28_14/E2
S10_634811	10	634811	6.35E-03	GL21_14/E2
S10_59113364	10	59113364	6.38E-03	GL14_13/E1
S10_59113369	10	59113369	6.38E-03	GL14_13/E1
S10_59113372	10	59113372	6.38E-03	GL14_13/E1
S10_59113374	10	59113374	6.38E-03	GL14_13/E1
S10_59113412	10	59113412	6.38E-03	GL14_13/E1
S10_38695561	10	38695561	6.41E-03	GL35_14/E2
S10_56475813	10	56475813	6.46E-03	GL7_13/E1

SNP	Chromosome	Position	P.value	Trait
S10_7697840	10	7697840	6.48E-03	GL14_13/E1
S10_7697850	10	7697850	6.48E-03	GL14_13/E1
S10_7697851	10	7697851	6.48E-03	GL14_13/E1
S10_7697852	10	7697852	6.48E-03	GL14_13/E1
S10_7697853	10	7697853	6.48E-03	GL14_13/E1
S10_7697856	10	7697856	6.48E-03	GL14_13/E1
S10_7697858	10	7697858	6.48E-03	GL14_13/E1
S10_16865408	10	16865408	6.51E-03	GL14_13/E1
S10_16865449	10	16865449	6.51E-03	GL14_13/E1
S10_56914835	10	56914835	6.51E-03	GL14_13/E1
S10_41032913	10	41032913	6.63E-03	GL14_ac
S10_59298151	10	59298151	6.67E-03	GL21_14/E2
S10_4309617	10	4309617	6.70E-03	GL14_13/E1
S10_4309625	10	4309625	6.70E-03	GL14_13/E1
S10_16612854	10	16612854	6.71E-03	GL14_14/E2
S10_41032913	10	41032913	6.73E-03	GL21_ac
S10_4117657	10	4117657	6.73E-03	GL28_ac
S10_56428954	10	56428954	6.74E-03	GL7_ac
S10_56428957	10	56428957	6.74E-03	GL7_ac
S10_8595239	10	8595239	6.74E-03	GL42_13/E1
S10_8595240	10	8595240	6.74E-03	GL42_13/E1
S10_8595251	10	8595251	6.74E-03	GL42_13/E1
S10_5558734	10	5558734	6.77E-03	GL35_13/E1
S10_58758082	10	58758082	6.80E-03	GL14_13/E1
S10_7790256	10	7790256	6.83E-03	GL35_13/E1
S10_49536872	10	49536872	6.85E-03	GL35_13/E1
S10_7205331	10	7205331	6.85E-03	GL49_13/E1
S10_8750817	10	8750817	6.90E-03	GL14_14/E2
S10_8750813	10	8750813	6.90E-03	GL14_14/E2
S10_6132423	10	6132423	6.93E-03	GL21_14/E2
S10_4233975	10	4233975	6.97E-03	GL49_ac
S10_9709174	10	9709174	7.00E-03	GL14_13/E1
S10_16612854	10	16612854	7.00E-03	GL28_14/E2
S10_60231203	10	60231203	7.03E-03	GL7_ac
S10_60231243	10	60231243	7.03E-03	GL7_ac
S10_56508509	10	56508509	7.03E-03	GL14_13/E1
S10_56508557	10	56508557	7.03E-03	GL14_13/E1
S10_7205329	10	7205329	7.11E-03	GL49_13/E1
S10_56428954	10	56428954	7.16E-03	GL21_ac
S10_56428957	10	56428957	7.16E-03	GL21_ac
S10_53690458	10	53690458	7.16E-03	GL35_13/E1
S10_53400859	10	53400859	7.16E-03	GL42_ac
S10_53400870	10	53400870	7.16E-03	GL42_ac
S10_6132423	10	6132423	7.18E-03	GL21_13/E1
S10_8698707	10	8698707	7.19E-03	GL49_14/E2
S10_13483803	10	13483803	7.20E-03	GL35_14/E2
S10_5688557	10	5688557	7.20E-03	GL28_14/E2
S10_57512927	10	57512927	7.24E-03	GL42_14/E2
S10_49536872	10	49536872	7.25E-03	GL7_13/E1
S10_4309617	10	4309617	7.29E-03	GL7_ac
S10_4309625	10	4309625	7.29E-03	GL7_ac

SNP	Chromosome	Position	P.value	Trait
S10_57714139	10	57714139	7.30E-03	GL7_13/E1
S10_5558734	10	5558734	7.31E-03	GL49_13/E1
S10_55831009	10	55831009	7.32E-03	GL42_13/E1
S10_5210528	10	5210528	7.32E-03	GL14_ac
S10_4233975	10	4233975	7.32E-03	GL21_13/E1
S10_16612854	10	16612854	7.35E-03	GL21_14/E2
S10_57512927	10	57512927	7.38E-03	GL42_ac
S10_7697840	10	7697840	7.40E-03	GL21_13/E1
S10_7697850	10	7697850	7.40E-03	GL21_13/E1
S10_7697851	10	7697851	7.40E-03	GL21_13/E1
S10_7697852	10	7697852	7.40E-03	GL21_13/E1
S10_7697853	10	7697853	7.40E-03	GL21_13/E1
S10_7697856	10	7697856	7.40E-03	GL21_13/E1
S10_7697858	10	7697858	7.40E-03	GL21_13/E1
S10_54838977	10	54838977	7.44E-03	GL14_14/E2
S10_7091500	10	7091500	7.45E-03	GL14_ac
S10_52214688	10	52214688	7.46E-03	GL21_14/E2
S10_5521872	10	5521872	7.48E-03	GL42_14/E2
S10_41510797	10	41510797	7.53E-03	GL49_ac
S10_5831753	10	5831753	7.55E-03	GL21_14/E2
S10_54938193	10	54938193	7.58E-03	GL7_14/E2
S10_54938226	10	54938226	7.58E-03	GL7_14/E2
S10_54938227	10	54938227	7.58E-03	GL7_14/E2
S10_54938228	10	54938228	7.58E-03	GL7_14/E2
S10_12932699	10	12932699	7.65E-03	GL14_14/E2
S10_45765686	10	45765686	7.66E-03	GL14_ac
S10_56914835	10	56914835	7.69E-03	GL7_13/E1
S10_60973291	10	60973291	7.78E-03	GL21_ac
S10_56428954	10	56428954	7.79E-03	GL35_13/E1
S10_56428957	10	56428957	7.79E-03	GL35_13/E1
S10_56475813	10	56475813	7.80E-03	GL49_13/E1
S10_54919953	10	54919953	7.83E-03	GL7_ac
S10_49892187	10	49892187	7.98E-03	GL49_13/E1
S10_49892188	10	49892188	7.98E-03	GL49_13/E1
S10_7917302	10	7917302	7.98E-03	GL28_13/E1
S10_4309617	10	4309617	7.99E-03	GL28_ac
S10_4309625	10	4309625	7.99E-03	GL28_ac
S10_13178326	10	13178326	8.03E-03	GL21_ac
S10_42944613	10	42944613	8.04E-03	GL42_13/E1
S10_53972403	10	53972403	8.05E-03	GL35_13/E1
S10_12932699	10	12932699	8.09E-03	GL35_14/E2
S10_7091415	10	7091415	8.14E-03	GL28_14/E2
S10_57404251	10	57404251	8.14E-03	GL7_13/E1
S10_57804222	10	57804222	8.15E-03	GL14_13/E1
S10_54897091	10	54897091	8.17E-03	GL21_14/E2
S10_6049695	10	6049695	8.20E-03	GL7_13/E1
S10_54919953	10	54919953	8.20E-03	GL49_ac
S10_8873501	10	8873501	8.23E-03	GL49_ac
S10_4612082	10	4612082	8.25E-03	GL7_14/E2
S10_4612104	10	4612104	8.25E-03	GL7_14/E2
S10_10690088	10	10690088	8.26E-03	GL14_13/E1

SNP	Chromosome	Position	P.value	Trait
S10_60091545	10	60091545	8.28E-03	GL49_14/E2
S10_60091546	10	60091546	8.28E-03	GL49_14/E2
S10_60091550	10	60091550	8.28E-03	GL49_14/E2
S10_60091555	10	60091555	8.28E-03	GL49_14/E2
S10_59292088	10	59292088	8.30E-03	GL7_14/E2
S10_59292089	10	59292089	8.30E-03	GL7_14/E2
S10_45765686	10	45765686	8.35E-03	GL35_ac
S10_56475813	10	56475813	8.36E-03	GL35_13/E1
S10_56428954	10	56428954	8.38E-03	GL7_13/E1
S10_56428957	10	56428957	8.38E-03	GL7_13/E1
S10_58499266	10	58499266	8.43E-03	GL14_13/E1
S10_57663572	10	57663572	8.46E-03	GL14_ac
S10_44016129	10	44016129	8.50E-03	GL49_13/E1
S10_44016131	10	44016131	8.50E-03	GL49_13/E1
S10_44016135	10	44016135	8.50E-03	GL49_13/E1
S10_44016174	10	44016174	8.50E-03	GL49_13/E1
S10_7697840	10	7697840	8.55E-03	GL7_13/E1
S10_7697850	10	7697850	8.55E-03	GL7_13/E1
S10_7697851	10	7697851	8.55E-03	GL7_13/E1
S10_7697852	10	7697852	8.55E-03	GL7_13/E1
S10_7697853	10	7697853	8.55E-03	GL7_13/E1
S10_7697856	10	7697856	8.55E-03	GL7_13/E1
S10_7697858	10	7697858	8.55E-03	GL7_13/E1
S10_8873501	10	8873501	8.57E-03	GL14_14/E2
S10_43137265	10	43137265	8.57E-03	GL42_14/E2
S10_4058699	10	4058699	8.58E-03	GL28_ac
S10_4058702	10	4058702	8.58E-03	GL28_ac
S10_4058708	10	4058708	8.58E-03	GL28_ac
S10_6100255	10	6100255	8.69E-03	GL7_13/E1
S10_50505397	10	50505397	8.70E-03	GL49_ac
S10_50505399	10	50505399	8.70E-03	GL49_ac
S10_50505400	10	50505400	8.70E-03	GL49_ac
S10_10690088	10	10690088	8.71E-03	GL7_13/E1
S10_8521536	10	8521536	8.74E-03	GL42_13/E1
S10_58758082	10	58758082	8.75E-03	GL7_13/E1
S10_56987389	10	56987389	8.78E-03	GL42_ac
S10_58276305	10	58276305	8.80E-03	GL28_14/E2
S10_7536572	10	7536572	8.88E-03	GL35_13/E1
S10_8873222	10	8873222	8.88E-03	GL35_14/E2
S10_8873243	10	8873243	8.88E-03	GL35_14/E2
S10_6132423	10	6132423	8.88E-03	GL14_14/E2
S10_56475813	10	56475813	8.90E-03	GL28_13/E1
S10_15185284	10	15185284	8.90E-03	GL21_14/E2
S10_53333596	10	53333596	8.94E-03	GL14_ac
S10_53333597	10	53333597	8.94E-03	GL14_ac
S10_53333598	10	53333598	8.94E-03	GL14_ac
S10_53333607	10	53333607	8.94E-03	GL14_ac
S10_4231550	10	4231550	8.94E-03	GL28_ac
S10_4233975	10	4233975	8.99E-03	GL28_ac
S10_16865408	10	16865408	9.09E-03	GL21_13/E1
S10_16865449	10	16865449	9.09E-03	GL21_13/E1

SNP	Chromosome	Position	P.value	Trait
S10_7917302	10	7917302	9.10E-03	GL42_13/E1
S10_6132413	10	6132413	9.12E-03	GL21_14/E2
S10_41510797	10	41510797	9.12E-03	GL28_13/E1
S10_51738778	10	51738778	9.14E-03	GL49_ac
S10_43137265	10	43137265	9.15E-03	GL49_ac
S10_57404251	10	57404251	9.26E-03	GL14_13/E1
S10_5187557	10	5187557	9.27E-03	GL21_14/E2
S10_9680166	10	9680166	9.31E-03	GL21_14/E2
S10_5212475	10	5212475	9.33E-03	GL28_13/E1
S10_4078224	10	4078224	9.35E-03	GL14_14/E2
S10_4078254	10	4078254	9.35E-03	GL14_14/E2
S10_54897091	10	54897091	9.38E-03	GL28_ac
S10_9614039	10	9614039	9.39E-03	GL14_ac
S10_54919953	10	54919953	9.42E-03	GL28_14/E2
S10_6355417	10	6355417	9.42E-03	GL14_ac
S10_54938226	10	54938226	9.45E-03	GL7_ac
S10_54938228	10	54938228	9.45E-03	GL7_ac
S10_54938193	10	54938193	9.45E-03	GL7_ac
S10_54938227	10	54938227	9.45E-03	GL7_ac
S10_57805609	10	57805609	9.47E-03	GL14_13/E1
S10_54919953	10	54919953	9.49E-03	GL28_ac
S10_54388058	10	54388058	9.50E-03	GL14_ac
S10_5558734	10	5558734	9.51E-03	GL21_14/E2
S10_60058846	10	60058846	9.53E-03	GL42_14/E2
S10_60058874	10	60058874	9.53E-03	GL42_14/E2
S10_60058876	10	60058876	9.53E-03	GL42_14/E2
S10_58333653	10	58333653	9.53E-03	GL14_13/E1
S10_8263604	10	8263604	9.53E-03	GL35_14/E2
S10_48198669	10	48198669	9.54E-03	GL49_14/E2
S10_13178326	10	13178326	9.55E-03	GL7_ac
S10_965211	10	965211	9.56E-03	GL14_14/E2
S10_965186	10	965186	9.56E-03	GL14_14/E2
S10_3299123	10	3299123	9.58E-03	GL35_13/E1
S10_2596717	10	2596717	9.59E-03	GL42_14/E2
S10_58288752	10	58288752	9.63E-03	GL7_ac
S10_4117657	10	4117657	9.67E-03	GL35_ac
S10_6049695	10	6049695	9.67E-03	GL28_ac
S10_16865408	10	16865408	9.70E-03	GL14_ac
S10_16865449	10	16865449	9.70E-03	GL14_ac
S10_4309617	10	4309617	9.80E-03	GL7_13/E1
S10_4309625	10	4309625	9.80E-03	GL7_13/E1
S10_4117657	10	4117657	9.83E-03	GL35_13/E1
S10_58217210	10	58217210	9.84E-03	GL14_13/E1
S10_56635633	10	56635633	9.84E-03	GL14_13/E1
S10_44899734	10	44899734	9.85E-03	GL42_ac
S10_56475813	10	56475813	9.86E-03	GL49_ac
S10_4068596	10	4068596	9.91E-03	GL28_ac
S10_6355417	10	6355417	9.91E-03	GL14_14/E2
S10_7697850	10	7697850	9.92E-03	GL21_ac
S10_7697851	10	7697851	9.92E-03	GL21_ac
S10_7697852	10	7697852	9.92E-03	GL21_ac

SNP	Chromosome	Position	P.value	Trait
S10_7697853	10	7697853	9.92E-03	GL21_ac
S10_7697856	10	7697856	9.92E-03	GL21_ac
S10_7697858	10	7697858	9.92E-03	GL21_ac
S10_7697840	10	7697840	9.92E-03	GL21_ac
S10_8813653	10	8813653	9.93E-03	GL21_14/E2
S10_8832996	10	8832996	9.94E-03	GL35_13/E1
S10_7091500	10	7091500	9.95E-03	GL14_13/E1
S10_44016129	10	44016129	9.96E-03	GL42_13/E1
S10_44016131	10	44016131	9.96E-03	GL42_13/E1
S10_44016135	10	44016135	9.96E-03	GL42_13/E1
S10_44016174	10	44016174	9.96E-03	GL42_13/E1
S10_54897091	10	54897091	9.99E-03	GL35_14/E2
S10_60973291	10	60973291	9.99E-03	GL28_ac
S10_6100258	10	6100258	9.99E-03	GL7_ac

Catogoery	Total	SNPs
GWAS signif SNP J2614/RSG04008 SBI-10 SNP SBI-10L(45-60Mb)	96	S10_51738778
		S10_57510942
		S10_54841288
		S10_59113364
		S10_57805609
		S10_53333607
		S10_58333653
		S10_56987389
		S10_54919953
		S10_46606416
		S10_53317190
		S10_60231243
		S10_53972403
		S10_59298151
		S10_56914835
		S10_55831009
		S10_58288752
		S10_52971607
		S10_60654069
		S10_53400870
		S10_50945046
		S10_50405318
		S10_49892188
		S10_54841484
		S10_59113374
		S10_54915163
		S10_58599753
		S10_54841321
		S10_58566780
		S10_52971581
		S10_59292089
		S10_56428954
		S10_54938193
		S10_59113372
		S10_56635633
		S10_53333598
		S10_57804222
		S10_48512624
		S10_49892187
		S10_54841304
		S10_48198669
		S10_56428957
		S10_54841489
		S10_46573203
		S10_53690458
		S10_58217210
		S10_56508509
		S10_53333597
		S10_54915154
		S10_52971592
		S10_58276305
		S10_60091555
		S10_54938226
		S10_60058874
		S10_54915161
		S10_60091545
		S10_53400859
		S10_48929913
		S10_60231203
		S10_54897091
		S10_59113369
		S10_50865236
		S10_57714139
		S10_53333596
		S10_60091546

Catogoery	Total	SNPs
		S10_57663572
		S10_50725358
		S10_60058876
		S10_55953518
		S10_54938228
		S10_59113412
		S10_48868738
		S10_55505116
		S10_50505399
		S10_48905718
		S10_54388058
		S10_56475813
		S10_58758082
		S10_45765686
		S10_60973291
		S10_49536872
		S10_50505400
		S10_54938227
		S10_57512927
		S10_60058846
		S10_59292088
		S10_60091550
		S10_55953554
		S10_56508557
		S10_50505397
		S10_58028206
		S10_57404251
		S10_54838977
		S10_56784622
		S10_48512575
		S10_52214688
J2614/RSG04008 SBI-10 SNP SBI-10L(45-60Mb)	1418	S10_54493047
		S10_51128543
		S10_55164775
		S10_55850047
		S10_53353127
		S10_54501308
		S10_53197912
		S10_53963237
		S10_60135253
		S10_58920166
		S10_52212145
		S10_57295957
		S10_50701747
		S10_59799901
		S10_56803856
		S10_46045728
		S10_53557780
		S10_58515854
		S10_60231108
		S10_56417482
		S10_52212161
		S10_55948696
		S10_60593638
		S10_56914920
		S10_55765421
		S10_54491575
		S10_52815169
		S10_49639791
		S10_56299294
		S10_53975344
		S10_55278625
		S10_58929730
		S10_56847317

Catogoery	Total	SNPs
		S10_48876558
		S10_59696189
		S10_54491612
		S10_58302476
		S10_52451129
		S10_54952947
		S10_51087166
		S10_54492779
		S10_46016738
		S10_56918504
		S10_51457148
		S10_50273432
		S10_54490326
		S10_56608572
		S10_50867370
		S10_55679720
		S10_49563208
		S10_53962999
		S10_53375455
		S10_59878644
		S10_54663080
		S10_56746132
		S10_59594781
		S10_50029310
		S10_60593660
		S10_51805234
		S10_52520637
		S10_60551646
		S10_55676149
		S10_53839087
		S10_58565687
		S10_48237953
		S10_53839115
		S10_58417141
		S10_59187850
		S10_54490533
		S10_53818291
		S10_56352496
		S10_60545564
		S10_51082222
		S10_59854876
		S10_59513463
		S10_58566782
		S10_60902159
		S10_45814238
		S10_53975349
		S10_51923306
		S10_57745002
		S10_51497655
		S10_50915580
		S10_46110218
		S10_60572089
		S10_50029750
		S10_52324138
		S10_61153460
		S10_52971994
		S10_45829059
		S10_55998859
		S10_54037106
		S10_51464950
		S10_56303321
		S10_59593663
		S10_56475108
		S10_55527635

Catogoery	Total	SNPs
		S10_58704538
		S10_53108340
		S10_54834121
		S10_53509963
		S10_53975341
		S10_53317408
		S10_59620312
		S10_58728876
		S10_51083132
		S10_58599849
		S10_48702096
		S10_55606379
		S10_53962031
		S10_55679761
		S10_56641589
		S10_55679719
		S10_59892977
		S10_55608528
		S10_57566449
		S10_56508618
		S10_58042627
		S10_49332738
		S10_58564174
		S10_50706002
		S10_55632302
		S10_51082267
		S10_48114827
		S10_48226443
		S10_54491305
		S10_52025707
		S10_54952945
		S10_52025694
		S10_60602919
		S10_58805470
		S10_55999093
		S10_57511037
		S10_60571994
		S10_52520575
		S10_58920175
		S10_51706472
		S10_60513646
		S10_50705999
		S10_52991179
		S10_60872627
		S10_48114860
		S10_59750378
		S10_60231220
		S10_59355959
		S10_48208865
		S10_56508631
		S10_50169630
		S10_56987374
		S10_60513644
		S10_55345431
		S10_52969717
		S10_60566724
		S10_45354184
		S10_54499693
		S10_55999632
		S10_57512997
		S10_58065961
		S10_55298896
		S10_59355948
		S10_60593609
		S10_55277339

Catogoery	Total	SNPs
		S10_55761173
		S10_60593601
		S10_57197754
		S10_52969624
		S10_55413702
		S10_46358525
		S10_52025693
		S10_56789221
		S10_47766555
		S10_57762743
		S10_58565700
		S10_57809876
		S10_53616305
		S10_48208881
		S10_52681154
		S10_60120147
		S10_47253849
		S10_56195617
		S10_50706047
		S10_54375780
		S10_55676131
		S10_57709483
		S10_52353473
		S10_57534223
		S10_59352605
		S10_55973739
		S10_56749623
		S10_51082216
		S10_49810375
		S10_54961246
		S10_59491003
		S10_56632413
		S10_57295224
		S10_55674363
		S10_54891105
		S10_60117831
		S10_58188386
		S10_59113218
		S10_56467139
		S10_56987464
		S10_53108339
		S10_51792131
		S10_52508184
		S10_56110888
		S10_55973848
		S10_51464944
		S10_59837691
		S10_49884951
		S10_53818752
		S10_55655606
		S10_56006312
		S10_52969580
		S10_55904321
		S10_56507025
		S10_52283379
		S10_56006574
		S10_55277344
		S10_59851188
		S10_54491611
		S10_60034872
		S10_58292977
		S10_50706040
		S10_56708894
		S10_51893835
		S10_56885802

Catogoery	Total	SNPs
		S10_60545556
		S10_60572192
		S10_56411378
		S10_61190725
		S10_57507647
		S10_60614161
		S10_56508724
		S10_56989401
		S10_50702058
		S10_58950644
		S10_50918727
		S10_59696267
		S10_48401859
		S10_55164757
		S10_59139829
		S10_60612242
		S10_46110057
		S10_57295224
		S10_55586987
		S10_56847351
		S10_54890557
		S10_55679791
		S10_57660753
		S10_51339006
		S10_54148706
		S10_46016748
		S10_50029743
		S10_56523490
		S10_60160203
		S10_53197886
		S10_53197909
		S10_59499391
		S10_54809401
		S10_55998507
		S10_45742241
		S10_53944270
		S10_56046667
		S10_55375263
		S10_56770478
		S10_59697086
		S10_56471264
		S10_59303885
		S10_52972135
		S10_53491085
		S10_60468754
		S10_56087512
		S10_57663473
		S10_55680567
		S10_54890109
		S10_46041365
		S10_55880871
		S10_59034786
		S10_52025659
		S10_53709640
		S10_47768340
		S10_54918531
		S10_55164765
		S10_53080452
		S10_54313035
		S10_55481309
		S10_54037100
		S10_53944322
		S10_57197754
		S10_60872509
		S10_57295224

Catogoery	Total	SNPs
		S10_55678367
		S10_51531086
		S10_50169716
		S10_55277341
		S10_55850047
		S10_60841479
		S10_50706000
		S10_46110103
		S10_55598473
		S10_55679725
		S10_50427488
		S10_56466498
		S10_60468749
		S10_50867359
		S10_48930598
		S10_58920059
		S10_54139188
		S10_54915018
		S10_50029747
		S10_59844168
		S10_51537075
		S10_53973176
		S10_58920182
		S10_54952045
		S10_60231299
		S10_46000219
		S10_60146528
		S10_58089765
		S10_60613755
		S10_60117639
		S10_58565704
		S10_54493025
		S10_57511036
		S10_50402880
		S10_58031253
		S10_60864633
		S10_52972007
		S10_56466519
		S10_55655838
		S10_57295224
		S10_57093649
		S10_60468746
		S10_51767683
		S10_48208212
		S10_53944318
		S10_52212157
		S10_56466518
		S10_55618490
		S10_55680563
		S10_59476916
		S10_54553482
		S10_53391063
		S10_59474781
		S10_51051970
		S10_60572071
		S10_52450168
		S10_57158599
		S10_57530992
		S10_55761214
		S10_56761722
		S10_46573604
		S10_55604233
		S10_48237949
		S10_56769950
		S10_60545563

Catogoery	Total	SNPs
		S10_46675672
		S10_60971005
		S10_48930598
		S10_52407732
		S10_58276722
		S10_60135251
		S10_55602787
		S10_51531075
		S10_49151184
		S10_51464945
		S10_54499690
		S10_58018474
		S10_60034894
		S10_59265767
		S10_48215508
		S10_59750331
		S10_55277352
		S10_53556850
		S10_54938287
		S10_54841474
		S10_60255230
		S10_57265445
		S10_56132414
		S10_57663473
		S10_57750536
		S10_55375160
		S10_49346370
		S10_59498752
		S10_55504343
		S10_55270382
		S10_53963098
		S10_54952949
		S10_58065962
		S10_54375507
		S10_54493420
		S10_47253360
		S10_55783068
		S10_50793744
		S10_51605417
		S10_58087992
		S10_58276434
		S10_55993145
		S10_53333981
		S10_49810362
		S10_57683836
		S10_60822245
		S10_54575547
		S10_54005450
		S10_56466500
		S10_56391607
		S10_55839533
		S10_52969654
		S10_51934289
		S10_51866536
		S10_57032509
		S10_60623608
		S10_59757527
		S10_60612176
		S10_56746165
		S10_58704599
		S10_52419817
		S10_53502240
		S10_56847350
		S10_51531079
		S10_57202665

Catogoery	Total	SNPs
		S10_48116045
		S10_60922122
		S10_54148705
		S10_52924958
		S10_61153378
		S10_54098027
		S10_50742820
		S10_58908285
		S10_59696189
		S10_58901865
		S10_60695074
		S10_57217358
		S10_55164764
		S10_55164773
		S10_54375504
		S10_55680576
		S10_60173717
		S10_50867365
		S10_53966922
		S10_52213950
		S10_54841598
		S10_56749818
		S10_60633020
		S10_53197914
		S10_57215437
		S10_59637804
		S10_60322316
		S10_60117648
		S10_49621607
		S10_55655487
		S10_59696268
		S10_56030236
		S10_57295919
		S10_59342868
		S10_51706456
		S10_55691249
		S10_54499704
		S10_52486479
		S10_58553403
		S10_57181402
		S10_60572000
		S10_51517151
		S10_52973075
		S10_54890508
		S10_59850807
		S10_46045563
		S10_51082261
		S10_54890514
		S10_58276705
		S10_58920143
		S10_53357061
		S10_59708582
		S10_46110060
		S10_46016765
		S10_59854935
		S10_57688301
		S10_54148715
		S10_56467046
		S10_56989399
		S10_58920181
		S10_56006319
		S10_56236894
		S10_54491653
		S10_52213952
		S10_50508494

Catogoery	Total	SNPs
		S10_57512996
		S10_54841618
		S10_53391059
		S10_49362481
		S10_46573616
		S10_56417244
		S10_56458521
		S10_51934278
		S10_55655846
		S10_53455342
		S10_55976082
		S10_56744993
		S10_51523147
		S10_60971004
		S10_50705989
		S10_58805470
		S10_55999622
		S10_54037099
		S10_59750332
		S10_56803858
		S10_60767637
		S10_54493045
		S10_50867361
		S10_60174136
		S10_59034726
		S10_55680523
		S10_57534189
		S10_53394524
		S10_50706016
		S10_56830947
		S10_60513711
		S10_58304209
		S10_57881382
		S10_53225095
		S10_53197915
		S10_60925330
		S10_58565703
		S10_51792164
		S10_47253248
		S10_54663078
		S10_51939336
		S10_52025948
		S10_59849054
		S10_50918673
		S10_54841360
		S10_60231118
		S10_53486317
		S10_55164777
		S10_58302410
		S10_58704538
		S10_55164772
		S10_60602924
		S10_48918084
		S10_58293030
		S10_56352304
		S10_58602694
		S10_57714076
		S10_55819374
		S10_52782615
		S10_47766559
		S10_54663072
		S10_56918503
		S10_56986177
		S10_61153461
		S10_49621608

Catogoery	Total	SNPs
		S10_55181620
		S10_58191126
		S10_53197911
		S10_59228735
		S10_53319120
		S10_55164754
		S10_60612272
		S10_54407969
		S10_50706008
		S10_56855696
		S10_60091616
		S10_55527634
		S10_57653551
		S10_49463259
		S10_51163559
		S10_56641518
		S10_56575280
		S10_53966845
		S10_53375456
		S10_54499711
		S10_54952056
		S10_48226286
		S10_49463058
		S10_58563474
		S10_61085454
		S10_59034801
		S10_50706054
		S10_59841565
		S10_56885748
		S10_60110925
		S10_57663473
		S10_48092146
		S10_56283783
		S10_48237824
		S10_58118819
		S10_58805470
		S10_60053791
		S10_60706294
		S10_58088036
		S10_51337149
		S10_58276433
		S10_56803853
		S10_54944453
		S10_45831812
		S10_49822373
		S10_57281857
		S10_46110099
		S10_56854442
		S10_54692912
		S10_55675394
		S10_53375457
		S10_49144697
		S10_53961062
		S10_51971962
		S10_54493035
		S10_54961342
		S10_58057692
		S10_58191038
		S10_59849058
		S10_61027454
		S10_53197916
		S10_60557352
		S10_60034990
		S10_58598738
		S10_52972009

Catogoery	Total	SNPs
		S10_49463271
		S10_54839763
		S10_55808453
		S10_50202583
		S10_54891182
		S10_49099586
		S10_59700935
		S10_60231311
		S10_50918674
		S10_54915058
		S10_54493421
		S10_56299271
		S10_58758633
		S10_56417385
		S10_52025663
		S10_59852562
		S10_57306758
		S10_54663069
		S10_60551643
		S10_58704538
		S10_54392616
		S10_50365959
		S10_49100577
		S10_54491440
		S10_60619195
		S10_51464947
		S10_58931054
		S10_58610981
		S10_50273445
		S10_61040218
		S10_60603118
		S10_52281274
		S10_56466516
		S10_59294286
		S10_53403530
		S10_50867351
		S10_56708893
		S10_54493453
		S10_59372267
		S10_53189230
		S10_55270383
		S10_56937658
		S10_50168382
		S10_56466509
		S10_49563225
		S10_53108642
		S10_54890148
		S10_50169723
		S10_56769948
		S10_52995996
		S10_58694071
		S10_56361883
		S10_50742640
		S10_60572048
		S10_54148714
		S10_60135449
		S10_51934288
		S10_51087131
		S10_55819330
		S10_57157909
		S10_54491533
		S10_54841421
		S10_56632417
		S10_60633029
		S10_55181668

Catogoery	Total	SNPs
		S10_59831050
		S10_47766572
		S10_50915583
		S10_60593639
		S10_52995997
		S10_55998504
		S10_57128986
		S10_52199633
		S10_60602929
		S10_58563469
		S10_56468091
		S10_60144111
		S10_49332205
		S10_55277348
		S10_55676176
		S10_56458646
		S10_56854454
		S10_60518102
		S10_46045708
		S10_49924924
		S10_60117668
		S10_58187114
		S10_53557784
		S10_56352583
		S10_56110887
		S10_47248652
		S10_46110058
		S10_59791228
		S10_52212141
		S10_55345435
		S10_58293029
		S10_47253395
		S10_56918510
		S10_56465522
		S10_59784952
		S10_60513647
		S10_57663473
		S10_54491782
		S10_52508157
		S10_56476051
		S10_55208611
		S10_56415044
		S10_53197885
		S10_46045590
		S10_58293031
		S10_60231331
		S10_55261261
		S10_56374942
		S10_53706468
		S10_48402220
		S10_52971370
		S10_57566464
		S10_56770477
		S10_57663626
		S10_55503979
		S10_51939403
		S10_58921104
		S10_50413373
		S10_52969605
		S10_46680940
		S10_52408290
		S10_55761246
		S10_58565705
		S10_54490299

Catogoery	Total	SNPs
		S10_61153371
		S10_49922700
		S10_51082196
		S10_60864636
		S10_53197917
		S10_54138767
		S10_50413380
		S10_60038637
		S10_60308474
		S10_58087999
		S10_54493385
		S10_56298255
		S10_55905323
		S10_60148190
		S10_50029742
		S10_54393393
		S10_56006554
		S10_45134206
		S10_59848048
		S10_50169626
		S10_50427465
		S10_57745005
		S10_53394489
		S10_59422845
		S10_50365960
		S10_59498757
		S10_50706009
		S10_55680559
		S10_52520638
		S10_52961064
		S10_49639838
		S10_60614111
		S10_54118313
		S10_59620328
		S10_60231304
		S10_49922765
		S10_51987143
		S10_54841789
		S10_55080504
		S10_58610539
		S10_49922733
		S10_55679788
		S10_50394264
		S10_54663067
		S10_56636288
		S10_48237938
		S10_55905116
		S10_60231407
		S10_55332354
		S10_57535908
		S10_47735777
		S10_57291725
		S10_53944323
		S10_56352502
		S10_60231364
		S10_55679724
		S10_51809997
		S10_46110121
		S10_54920078
		S10_55828810
		S10_49955060
		S10_59624350
		S10_52281513
		S10_60468750
		S10_58293034

Catogoery	Total	SNPs
		S10_56414677
		S10_56769949
		S10_49810340
		S10_58933441
		S10_55679837
		S10_52995982
		S10_50169644
		S10_57600325
		S10_53512408
		S10_59974908
		S10_56847296
		S10_56770479
		S10_54841750
		S10_58598658
		S10_47766571
		S10_58598733
		S10_57663473
		S10_55850047
		S10_58563475
		S10_51464966
		S10_54663070
		S10_55164766
		S10_60505066
		S10_60613735
		S10_54491608
		S10_58333198
		S10_52224927
		S10_52025658
		S10_53944274
		S10_59690880
		S10_56302132
		S10_56374818
		S10_60767684
		S10_57663473
		S10_56466517
		S10_60322310
		S10_57021183
		S10_51730311
		S10_59696667
		S10_50918671
		S10_58767339
		S10_54493396
		S10_54493457
		S10_53148305
		S10_45814078
		S10_51501680
		S10_54529963
		S10_59104863
		S10_55277334
		S10_49715312
		S10_51339002
		S10_59773433
		S10_52451215
		S10_58920183
		S10_55181794
		S10_50029746
		S10_59422842
		S10_56994002
		S10_54499700
		S10_53972308
		S10_55836631
		S10_55315074
		S10_57815884
		S10_61153456
		S10_60333222

Catogoery	Total	SNPs
		S10_59304423
		S10_59874512
		S10_59559274
		S10_56458532
		S10_60308400
		S10_49342560
		S10_57181179
		S10_54914723
		S10_51819111
		S10_50706043
		S10_53186854
		S10_55345438
		S10_60577532
		S10_57196793
		S10_59850129
		S10_59841690
		S10_60333223
		S10_58563472
		S10_51805264
		S10_59034803
		S10_59441633
		S10_53246175
		S10_60322315
		S10_52199666
		S10_57806877
		S10_53313191
		S10_50793992
		S10_54952946
		S10_48095185
		S10_54899228
		S10_49100560
		S10_54491617
		S10_51779766
		S10_52212144
		S10_49924859
		S10_52025703
		S10_54444615
		S10_54492811
		S10_56769947
		S10_55164776
		S10_57260050
		S10_56803851
		S10_60767638
		S10_50277442
		S10_59773490
		S10_56337814
		S10_55211783
		S10_56646511
		S10_49822376
		S10_55164753
		S10_60231305
		S10_52995995
		S10_57590703
		S10_50867355
		S10_57426890
		S10_51504877
		S10_55850047
		S10_56466511
		S10_60929771
		S10_57298754
		S10_57295224
		S10_54491347
		S10_51744542
		S10_50402839
		S10_53337622

Catogoery	Total	SNPs
		S10_57804193
		S10_54891180
		S10_60649673
		S10_59613549
		S10_57590596
		S10_58088607
		S10_55164762
		S10_59892311
		S10_59849056
		S10_56641607
		S10_51087099
		S10_57191924
		S10_56340570
		S10_53963256
		S10_55277335
		S10_49099541
		S10_57529752
		S10_52451349
		S10_52972082
		S10_53966921
		S10_54838782
		S10_56464599
		S10_54491644
		S10_59849060
		S10_56914823
		S10_57744999
		S10_51504822
		S10_49719876
		S10_51933207
		S10_57590618
		S10_51006848
		S10_58610366
		S10_56641590
		S10_59482408
		S10_57182476
		S10_53246183
		S10_55724994
		S10_52025708
		S10_60612486
		S10_58323275
		S10_56361881
		S10_54952934
		S10_49615255
		S10_58610983
		S10_58531246
		S10_60593652
		S10_54392589
		S10_49639804
		S10_54492768
		S10_59850128
		S10_59336236
		S10_58805470
		S10_49810361
		S10_60106142
		S10_58417139
		S10_54839800
		S10_58565708
		S10_55277346
		S10_52646279
		S10_53975340
		S10_56830985
		S10_56692703
		S10_60148188
		S10_52323643
		S10_51339001

Catogoery	Total	SNPs
		S10_60603152
		S10_56937377
		S10_51789941
		S10_58634498
		S10_53319122
		S10_60228405
		S10_60117646
		S10_57381554
		S10_58695241
		S10_47766566
		S10_54952038
		S10_59228749
		S10_55261319
		S10_59187846
		S10_56989405
		S10_56417317
		S10_59090835
		S10_61153372
		S10_48813436
		S10_60214866
		S10_60612267
		S10_50926165
		S10_53333957
		S10_53454779
		S10_54663077
		S10_53197929
		S10_58694164
		S10_54663071
		S10_54491310
		S10_53512501
		S10_58565690
		S10_56653607
		S10_55604323
		S10_58707073
		S10_58297650
		S10_52244563
		S10_55906018
		S10_57306778
		S10_60738788
		S10_53469309
		S10_60322286
		S10_59228736
		S10_60633706
		S10_61063564
		S10_55375159
		S10_48074234
		S10_57295952
		S10_46045715
		S10_59104911
		S10_49332286
		S10_55761184
		S10_51729940
		S10_58563466
		S10_53241691
		S10_57653534
		S10_58721909
		S10_58921117
		S10_58619039
		S10_56445036
		S10_55277336
		S10_50029744
		S10_58704597
		S10_60971009
		S10_59187852
		S10_59696269

Catogoery	Total	SNPs
		S10_51712776
		S10_60551647
		S10_56700087
		S10_56389957
		S10_55355408
		S10_59696265
		S10_53077439
		S10_60612481
		S10_58897087
		S10_49678427
		S10_55691261
		S10_54491220
		S10_57714034
		S10_56415056
		S10_54915046
		S10_60593657
		S10_54491446
		S10_60243256
		S10_58408926
		S10_55973890
		S10_57511034
		S10_50918670
		S10_53197875
		S10_59749988
		S10_57128992
		S10_45951468
		S10_53342865
		S10_46110152
		S10_52286440
		S10_58598712
		S10_50013115
		S10_60545562
		S10_51809985
		S10_54841833
		S10_59879873
		S10_54915053
		S10_53496511
		S10_54444616
		S10_54499694
		S10_51464943
		S10_55320722
		S10_48918085
		S10_47766560
		S10_56653608
		S10_52322143
		S10_52025946
		S10_57745004
		S10_54110936
		S10_51805184
		S10_54491422
		S10_48215457
		S10_59874482
		S10_58596839
		S10_60552099
		S10_60920070
		S10_56803854
		S10_48114855
		S10_57347175
		S10_54491775
		S10_56855613
		S10_52508155
		S10_57692900
		S10_52025730
		S10_58087972

Catogoery	Total	SNPs
		S10_59892362
		S10_52396296
		S10_55679722
		S10_52213936
		S10_53972297
		S10_59844015
		S10_57809613
		S10_51345407
		S10_57206595
		S10_53108341
		S10_46110145
		S10_51131121
		S10_57513049
		S10_55345437
		S10_60059042
		S10_54037107
		S10_53818722
		S10_46043449
		S10_58565707
		S10_53386896
		S10_59294116
		S10_52212146
		S10_53502252
		S10_56352463
		S10_58931054
		S10_48931198
		S10_60619196
		S10_48237948
		S10_52520575
		S10_57021181
		S10_55678368
		S10_48402238
		S10_56195632
		S10_53975348
		S10_57714017
		S10_57562475
		S10_51339000
		S10_49822386
		S10_54499686
		S10_59294324
		S10_55678348
		S10_56997186
		S10_59034742
		S10_56641522
		S10_56467077
		S10_50365958
		S10_55345388
		S10_55819212
		S10_56006316
		S10_58596541
		S10_51706523
		S10_59791137
		S10_51464925
		S10_51523141
		S10_52287479
		S10_56466497
		S10_58800380
		S10_48215488
		S10_50029748
		S10_60150914
		S10_53518613
		S10_55306987
		S10_58707072
		S10_54891183
		S10_56417260

Catogoery	Total	SNPs
		S10_56761493
		S10_57215489
		S10_59104832
		S10_57049562
		S10_53108331
		S10_58945562
		S10_59799283
		S10_59234324
		S10_52025646
		S10_58563470
		S10_50868319
		S10_59760818
		S10_58276719
		S10_55481308
		S10_55990186
		S10_55680562
		S10_54841727
		S10_58596845
		S10_56847252
		S10_60468748
		S10_56201321
		S10_52969741
		S10_57362981
		S10_54148718
		S10_52287191
		S10_48798807
		S10_58920523
		S10_53944317
		S10_46405565
		S10_51975446
		S10_58704603
		S10_60215727
		S10_52243430
		S10_61153373
		S10_55850047
		S10_59874513
		S10_52353511
		S10_49621651
		S10_46043459
		S10_55608501
		S10_52286804
		S10_53973163
		S10_54491183
		S10_50311563
		S10_50273436
		S10_47361452
		S10_49884947
		S10_57290169
		S10_57205091
		S10_60602928
		S10_53317330
		S10_60330354
		S10_54005372
		S10_55999796
		S10_45951367
		S10_56195582
		S10_58933428
		S10_59696189
		S10_56847264
		S10_54952044
		S10_54490914
		S10_52450139
		S10_60104607
		S10_50706046
		S10_56987390

Catogoery	Total	SNPs
		S10_50360545
		S10_50402836
		S10_48512737
		S10_55679717
		S10_60577527
		S10_56110892
		S10_54499713
		S10_56352500
		S10_58417069
		S10_54491227
		S10_55618485
		S10_51986914
		S10_52024555
		S10_55765420
		S10_54952948
		S10_57530991
		S10_55836629
		S10_52520636
		S10_51482143
		S10_48237952
		S10_48154038
		S10_58004094
		S10_53962916
		S10_50357907
		S10_54687999
		S10_48226499
		S10_55972410
		S10_51345335
		S10_54491578
		S10_59513462
		S10_51767686
		S10_56467067
		S10_49810363
		S10_56348980
		S10_48237937
		S10_52027306
		S10_56311879
		S10_60577616
		S10_60612495
		S10_53235767
		S10_50427459
		S10_53394522
		S10_51789935
		S10_55998501
		S10_56997188
		S10_55655486
		S10_52969662
		S10_57742930
		S10_59850134
		S10_60971008
		S10_60322317
		S10_52025710
		S10_60148189
		S10_53464745
		S10_51464942
		S10_55973956
		S10_55973891
		S10_50382635
		S10_52212137
		S10_50413384
		S10_50702543
		S10_57783305
		S10_50413390
		S10_56046738
		S10_59879960

Catogoery	Total	SNPs
		S10_53502301
		S10_56466508
		S10_54444617
		S10_51308062
		S10_58191224
		S10_53975346
		S10_57283437
		S10_54444619
		S10_59034730
		S10_54500202
		S10_60552533
		S10_59593345
		S10_46045589
		S10_52212162
		S10_59187849
		S10_51939329
		S10_51245140
		S10_56937655
		S10_58565702
		S10_55948790
		S10_58681032
		S10_50705966
		S10_56303383
		S10_50706012
		S10_49527091
		S10_53975350
		S10_59854936
		S10_60497043
		S10_60151035
		S10_53963007
		S10_54005394
		S10_55426930
		S10_60486819
		S10_60117590
		S10_55345165
		S10_57157948
		S10_54491659
		S10_60684319
		S10_59866374
		S10_51805235
		S10_57663473
		S10_58598727
		S10_53403684
		S10_54493042
		S10_52316579
		S10_55680561
		S10_50427498
		S10_45931754
		S10_60623625
		S10_56918509
		S10_54952049
		S10_56466524
		S10_54896832
		S10_54139341
		S10_54499692
		S10_53391094
		S10_55778193
		S10_58293033
		S10_46110061
		S10_56511507
		S10_55921709
		S10_56997187
		S10_57021184
		S10_60770004
		S10_50705998

Catogoery	Total	SNPs
		S10_53961047
		S10_52212134
		S10_58805470
		S10_53944310
		S10_53391088
		S10_55836452
		S10_55655606
		S10_48208864
		S10_51345410
		S10_52476293
		S10_60322312
		S10_54492813
		S10_56508615
		S10_50445172
		S10_58920539
		S10_49615295
		S10_56135787
		S10_53870950
		S10_57283396
GWAS signif SNP J2614/RSG04008 SBI-10 SNP	103	S10_7205329
		S10_13178326
		S10_15185284
		S10_9572293
		S10_8750817
		S10_2716110
		S10_9617926
		S10_5521872
		S10_8595239
		S10_965186
		S10_8521536
		S10_7697851
		S10_41510797
		S10_965211
		S10_13483803
		S10_8595240
		S10_2596717
		S10_10690088
		S10_4310973
		S10_4058702
		S10_9577350
		S10_7934181
		S10_7917302
		S10_7893655
		S10_4309625
		S10_7205331
		S10_5210528
		S10_5217988
		S10_16612854
		S10_4183532
		S10_7091415
		S10_9680166
		S10_4117657
		S10_8873501
		S10_4058699
		S10_8813653
		S10_42917852
		S10_4058708
		S10_4078224
		S10_6100255
		S10_7536572
		S10_44016131
		S10_7697840
		S10_5187557
		S10_5212475
		S10_7697850

Catogoery	Total	SNPs
		S10_18003751
		S10_44910447
		S10_9709174
		S10_10215201
		S10_6049695
		S10_9614039
		S10_7697858
		S10_9572249
		S10_16865449
		S10_8263604
		S10_8753539
		S10_5831753
		S10_44899734
		S10_7697853
		S10_3299123
		S10_7091500
		S10_44016129
		S10_4068596
		S10_5688557
		S10_6132413
		S10_4231550
		S10_6355417
		S10_6132423
		S10_43137265
		S10_43586473
		S10_574319
		S10_7697852
		S10_4309617
		S10_4612104
		S10_6100258
		S10_18003749
		S10_41032913
		S10_5558734
		S10_8698707
		S10_42944613
		S10_8873243
		S10_8873222
		S10_7697856
		S10_44016135
		S10_8832996
		S10_6808270
		S10_38695561
		S10_7434473
		S10_16865408
		S10_9577350
		S10_13677145
		S10_17330914
		S10_44016174
		S10_4612082
		S10_4078254
		S10_8750813
		S10_634811
		S10_4233975
		S10_7138802
		S10_8595251
		S10_7790256
		S10_12932699
J2614/RSG04008 SBI-10 SNP	1773	S10_5528585
		S10_8578211
		S10_1954776
		S10_8300125
		S10_4058774
		S10_7299698
		S10_11711944
		S10_4323523

Catogoery	Total	SNPs
		S10_32985943
		S10_4231584
		S10_7821412
		S10_1328445
		S10_2943407
		S10_3942573
		S10_40203994
		S10_12712881
		S10_1724616
		S10_7481171
		S10_3547628
		S10_7692703
		S10_12834853
		S10_8754028
		S10_11065086
		S10_8975967
		S10_386597
		S10_7107955
		S10_38405073
		S10_15981977
		S10_4488923
		S10_7821544
		S10_38113346
		S10_750235
		S10_2395657
		S10_4652380
		S10_44759545
		S10_15873288
		S10_15823588
		S10_7603526
		S10_6338727
		S10_16614656
		S10_1132431
		S10_6017059
		S10_1928141
		S10_6472258
		S10_4084526
		S10_18366510
		S10_17207263
		S10_1803178
		S10_5517812
		S10_15441151
		S10_7801686
		S10_40620394
		S10_7469101
		S10_9051908
		S10_1162835
		S10_2710942
		S10_7820121
		S10_25002527
		S10_13825620
		S10_2719918
		S10_6359491
		S10_1172886
		S10_9880720
		S10_4053918
		S10_3306523
		S10_2366076
		S10_16911567
		S10_11204660
		S10_41838785
		S10_4445626
		S10_9976755
		S10_2025094
		S10_7170715

Catogoery	Total	SNPs
		S10_976945
		S10_750358
		S10_10751753
		S10_1162703
		S10_5628553
		S10_750356
		S10_7157502
		S10_38918698
		S10_1827776
		S10_15311058
		S10_9441588
		S10_6016325
		S10_13479641
		S10_4237490
		S10_8750574
		S10_7790105
		S10_7201676
		S10_7559346
		S10_2257426
		S10_7878486
		S10_3927586
		S10_14645730
		S10_1830658
		S10_1733962
		S10_5528526
		S10_4226108
		S10_40201117
		S10_762593
		S10_10901110
		S10_2411545
		S10_5255608
		S10_750307
		S10_7043784
		S10_7103305
		S10_4109725
		S10_41123594
		S10_4216350
		S10_4602566
		S10_8206048
		S10_9809697
		S10_10995729
		S10_1938029
		S10_11452006
		S10_621919
		S10_3948337
		S10_2358749
		S10_3751531
		S10_16967955
		S10_8750564
		S10_7170613
		S10_9342246
		S10_4611804
		S10_11711908
		S10_13825617
		S10_3741097
		S10_10692549
		S10_4236897
		S10_1938063
		S10_7092800
		S10_8861886
		S10_15752277
		S10_3942574
		S10_2624211
		S10_84927
		S10_7352930

Catogoery	Total	SNPs
		S10_4313355
		S10_7934462
		S10_4141530
		S10_38695533
		S10_7512859
		S10_5608969
		S10_6533233
		S10_5688780
		S10_3006777
		S10_7170723
		S10_4124396
		S10_6375236
		S10_1093361
		S10_8533711
		S10_5557969
		S10_770243
		S10_6100256
		S10_40837591
		S10_5469560
		S10_7820439
		S10_7821419
		S10_3308516
		S10_7725762
		S10_7524945
		S10_4252692
		S10_13705205
		S10_4323515
		S10_11830539
		S10_7877102
		S10_4233934
		S10_5200239
		S10_979222
		S10_4611634
		S10_312224
		S10_3320764
		S10_11079621
		S10_4460432
		S10_4232829
		S10_524337
		S10_954209
		S10_1529444
		S10_7791923
		S10_4053921
		S10_4030750
		S10_4652723
		S10_2395611
		S10_4293834
		S10_7166512
		S10_16001506
		S10_4231670
		S10_6044473
		S10_4236917
		S10_10887543
		S10_4231920
		S10_9706943
		S10_5957765
		S10_12005282
		S10_3536844
		S10_5787713
		S10_6057510
		S10_3569834
		S10_574525
		S10_34085103
		S10_41054991
		S10_5881762

Catogoery	Total	SNPs
		S10_6017066
		S10_8292069
		S10_8873387
		S10_34622114
		S10_7469055
		S10_2362249
		S10_10888992
		S10_555857
		S10_11210509
		S10_2186456
		S10_43628079
		S10_3265785
		S10_15924551
		S10_11830573
		S10_10690279
		S10_2960225
		S10_1856073
		S10_1172500
		S10_4232901
		S10_35903825
		S10_17951364
		S10_1589165
		S10_4488980
		S10_44897635
		S10_4395071
		S10_7934094
		S10_11064990
		S10_4603326
		S10_7487333
		S10_2308558
		S10_7692713
		S10_13624073
		S10_1297013
		S10_6164270
		S10_6641672
		S10_4339703
		S10_11452008
		S10_6358347
		S10_544504
		S10_2025111
		S10_9048655
		S10_689159
		S10_13039182
		S10_1954754
		S10_8616678
		S10_4214542
		S10_3753630
		S10_1724624
		S10_9447483
		S10_1270047
		S10_1733966
		S10_1954772
		S10_8753995
		S10_4484624
		S10_7481175
		S10_1543171
		S10_7044477
		S10_5175157
		S10_38436425
		S10_1778053
		S10_8686380
		S10_7522447
		S10_555098
		S10_1860949
		S10_8813664

Catogoery	Total	SNPs
		S10_9052219
		S10_6999171
		S10_38125298
		S10_1280555
		S10_10889199
		S10_266106
		S10_5633705
		S10_8750566
		S10_10161242
		S10_44555205
		S10_6421401
		S10_10690326
		S10_9598047
		S10_43389880
		S10_1827791
		S10_9808834
		S10_3006786
		S10_10889103
		S10_2143366
		S10_3547624
		S10_12835325
		S10_4193987
		S10_8617201
		S10_5198848
		S10_4262864
		S10_16042521
		S10_7166546
		S10_4354584
		S10_5517949
		S10_7821415
		S10_38095360
		S10_5211154
		S10_7021748
		S10_17330546
		S10_1954770
		S10_10689999
		S10_979126
		S10_13716848
		S10_19383824
		S10_1780976
		S10_5171595
		S10_11452004
		S10_13705111
		S10_6355305
		S10_3942540
		S10_2507537
		S10_10752102
		S10_8619131
		S10_6655760
		S10_423662
		S10_7819808
		S10_1687538
		S10_17609974
		S10_2359902
		S10_4268880
		S10_17827977
		S10_11980570
		S10_17537883
		S10_41900917
		S10_10030835
		S10_43586440
		S10_12391613
		S10_7506695
		S10_9515890
		S10_3753631

Catogoery	Total	SNPs
		S10_1720296
		S10_4764563
		S10_43218042
		S10_16001505
		S10_5674283
		S10_2025106
		S10_11129942
		S10_3699364
		S10_13677821
		S10_582081
		S10_8988814
		S10_1172485
		S10_766283
		S10_3948318
		S10_40620345
		S10_40641673
		S10_7877112
		S10_18111828
		S10_4171524
		S10_1938412
		S10_4301979
		S10_8299802
		S10_4230512
		S10_2772851
		S10_4458579
		S10_9571982
		S10_2025078
		S10_9660702
		S10_2025116
		S10_9750276
		S10_6056858
		S10_11979689
		S10_10167349
		S10_761735
		S10_9130459
		S10_7170835
		S10_4124378
		S10_8842309
		S10_3124140
		S10_13677846
		S10_13735037
		S10_4471055
		S10_7801724
		S10_8861959
		S10_9750216
		S10_4059507
		S10_69649
		S10_6741172
		S10_386596
		S10_38965818
		S10_1156484
		S10_17077111
		S10_1135668
		S10_16460968
		S10_1937262
		S10_8657123
		S10_1488421
		S10_10881111
		S10_6808070
		S10_4109599
		S10_16649679
		S10_16174521
		S10_16969786
		S10_22524993
		S10_4237515

Catogoery	Total	SNPs
		S10_12824807
		S10_39546853
		S10_10901089
		S10_7111936
		S10_39911267
		S10_13705208
		S10_34188986
		S10_5214345
		S10_14679526
		S10_589321
		S10_7273508
		S10_2361402
		S10_38004364
		S10_91407
		S10_7416726
		S10_822618
		S10_1583371
		S10_16916414
		S10_252468
		S10_9880741
		S10_6706254
		S10_3011624
		S10_2383581
		S10_1162777
		S10_44897630
		S10_2624166
		S10_318783
		S10_9709425
		S10_6027583
		S10_8861919
		S10_7604402
		S10_7273514
		S10_1172508
		S10_12022231
		S10_1938520
		S10_282478
		S10_9515793
		S10_7157159
		S10_5517814
		S10_9864544
		S10_5054575
		S10_5720147
		S10_12649479
		S10_43600538
		S10_7460411
		S10_4698634
		S10_5550715
		S10_38484570
		S10_1162808
		S10_7454437
		S10_2435084
		S10_13595571
		S10_9986876
		S10_7273522
		S10_4602510
		S10_12356652
		S10_17051105
		S10_17848841
		S10_9130456
		S10_5501855
		S10_17173307
		S10_412775
		S10_4460409
		S10_4233778
		S10_19077200

Catogoery	Total	SNPs
		S10_4458482
		S10_4326856
		S10_4232828
		S10_4084449
		S10_7157484
		S10_9239469
		S10_1733985
		S10_10132759
		S10_1685829
		S10_8976564
		S10_22062679
		S10_8738374
		S10_3948343
		S10_2624164
		S10_4233938
		S10_11003030
		S10_17077129
		S10_44923073
		S10_6620349
		S10_4696160
		S10_4745787
		S10_7877072
		S10_7820187
		S10_9460898
		S10_7559302
		S10_768372
		S10_970831
		S10_3094765
		S10_6926859
		S10_684563
		S10_5974027
		S10_11197125
		S10_9349313
		S10_3806879
		S10_11979542
		S10_7093419
		S10_970310
		S10_7456456
		S10_43624959
		S10_412745
		S10_6669421
		S10_132819
		S10_1954759
		S10_5055020
		S10_5199326
		S10_7092806
		S10_1542436
		S10_3547625
		S10_15910093
		S10_40905634
		S10_2643329
		S10_5198887
		S10_719026
		S10_8533530
		S10_11222588
		S10_9022177
		S10_8533443
		S10_7680604
		S10_2025104
		S10_4764428
		S10_761756
		S10_684429
		S10_9559509
		S10_42060960
		S10_5787668

Catogoery	Total	SNPs
		S10_8977080
		S10_8818725
		S10_28784910
		S10_8742672
		S10_13922527
		S10_8873521
		S10_5787664
		S10_9978238
		S10_822577
		S10_449993
		S10_8976510
		S10_4293931
		S10_4158436
		S10_3455503
		S10_40204014
		S10_43007546
		S10_10024829
		S10_2570772
		S10_16612551
		S10_413150
		S10_4216387
		S10_4233823
		S10_38436437
		S10_7918051
		S10_5158639
		S10_41667828
		S10_3011389
		S10_42259282
		S10_4179157
		S10_5957733
		S10_16171256
		S10_2611944
		S10_15705930
		S10_2623015
		S10_7433088
		S10_4323756
		S10_1937223
		S10_462606
		S10_1542550
		S10_8053718
		S10_177180
		S10_5490590
		S10_7524892
		S10_6056836
		S10_6606346
		S10_9354817
		S10_38735183
		S10_3927553
		S10_18065108
		S10_5201410
		S10_34188990
		S10_2730762
		S10_761806
		S10_12963644
		S10_4495900
		S10_2025096
		S10_7509341
		S10_8896960
		S10_38750143
		S10_2426710
		S10_4233816
		S10_6056859
		S10_91767
		S10_4458544

Catogoery	Total	SNPs
		S10_5728473
		S10_9686962
		S10_7460376
		S10_6464136
		S10_6797940
		S10_8480028
		S10_5558149
		S10_11129877
		S10_3002676
		S10_21908863
		S10_1803402
		S10_689172
		S10_7750752
		S10_962305
		S10_5054609
		S10_5675838
		S10_1685957
		S10_13903186
		S10_5269782
		S10_3950582
		S10_1803774
		S10_11452012
		S10_6056865
		S10_2457056
		S10_1954778
		S10_7791944
		S10_2395620
		S10_15908969
		S10_7692708
		S10_1422265
		S10_7352922
		S10_6901859
		S10_12839145
		S10_9650899
		S10_4323627
		S10_2025108
		S10_5608263
		S10_2308596
		S10_6818386
		S10_5066549
		S10_10169416
		S10_6133413
		S10_4236894
		S10_11829928
		S10_34310928
		S10_2411546
		S10_4231040
		S10_9048174
		S10_4053913
		S10_17015293
		S10_113148
		S10_423663
		S10_13039211
		S10_38854868
		S10_1816013
		S10_970309
		S10_9516063
		S10_9049673
		S10_6555005
		S10_964442
		S10_6620386
		S10_2411530
		S10_10258692
		S10_6100133
		S10_12019727

Catogoery	Total	SNPs
		S10_15917635
		S10_38735184
		S10_4495947
		S10_1135669
		S10_9559492
		S10_6808068
		S10_11003045
		S10_473906
		S10_4651274
		S10_7750817
		S10_40152202
		S10_7869437
		S10_16866422
		S10_16614658
		S10_6028168
		S10_9709409
		S10_818208
		S10_5906917
		S10_2366085
		S10_12644976
		S10_15908953
		S10_44555195
		S10_5787681
		S10_4654533
		S10_1928131
		S10_3292842
		S10_42592251
		S10_6056856
		S10_2904399
		S10_15050752
		S10_7044060
		S10_8655310
		S10_5699663
		S10_5927658
		S10_5054612
		S10_1792090
		S10_9750349
		S10_5201417
		S10_1799198
		S10_14679525
		S10_10167351
		S10_4080071
		S10_44035977
		S10_4216383
		S10_7352923
		S10_11830541
		S10_7678887
		S10_41123436
		S10_16969854
		S10_91251
		S10_4345873
		S10_9627221
		S10_5957749
		S10_15311040
		S10_15777600
		S10_7821429
		S10_15824446
		S10_4606334
		S10_10151865
		S10_17172570
		S10_750327
		S10_7934076
		S10_5214342
		S10_967084

Catogoery	Total	SNPs
		S10_7917709
		S10_7522672
		S10_38937330
		S10_4053911
		S10_3942576
		S10_942011
		S10_11838062
		S10_42060733
		S10_8964428
		S10_4178316
		S10_9049686
		S10_40201013
		S10_5257023
		S10_41754515
		S10_767969
		S10_2868370
		S10_4218952
		S10_9648539
		S10_4764562
		S10_75753
		S10_7878053
		S10_6077901
		S10_9559447
		S10_7692720
		S10_7522676
		S10_40502377
		S10_34622112
		S10_2366077
		S10_10258677
		S10_16533637
		S10_5801094
		S10_7878447
		S10_17106015
		S10_7917721
		S10_7877251
		S10_7877072
		S10_761743
		S10_13922695
		S10_6998940
		S10_2258821
		S10_7103107
		S10_2366082
		S10_16609766
		S10_9352423
		S10_1162831
		S10_18226947
		S10_1300281
		S10_17077130
		S10_41667577
		S10_1954775
		S10_7201714
		S10_8645340
		S10_1674004
		S10_17173627
		S10_9880714
		S10_8297472
		S10_13461505
		S10_719031
		S10_4458532
		S10_386604
		S10_6069870
		S10_7522671
		S10_4232896
		S10_1792190

Catogoery	Total	SNPs
		S10_17172565
		S10_4484607
		S10_12231600
		S10_9280235
		S10_1542549
		S10_2194407
		S10_10819549
		S10_3927808
		S10_1419151
		S10_621913
		S10_13735105
		S10_5178385
		S10_10167347
		S10_682024
		S10_8078568
		S10_4183307
		S10_98374
		S10_7750771
		S10_1189585
		S10_9598061
		S10_2025120
		S10_4078155
		S10_9690324
		S10_6219290
		S10_4240820
		S10_15181823
		S10_5957748
		S10_42178569
		S10_42249695
		S10_7604398
		S10_8632677
		S10_13046019
		S10_5055017
		S10_13922760
		S10_1814101
		S10_4092049
		S10_318788
		S10_3236439
		S10_8976533
		S10_2383583
		S10_10891365
		S10_1767829
		S10_1699468
		S10_386600
		S10_1937221
		S10_4079984
		S10_3959556
		S10_3124024
		S10_719025
		S10_14679524
		S10_318784
		S10_16627264
		S10_9048869
		S10_750372
		S10_5906944
		S10_10861424
		S10_1688416
		S10_386599
		S10_1132316
		S10_12711560
		S10_8742675
		S10_8200367
		S10_11774820
		S10_13389681
		S10_2383575

Catogoery	Total	SNPs
		S10_15647
		S10_4395061
		S10_2359929
		S10_554335
		S10_9130442
		S10_8896934
		S10_674381
		S10_4252731
		S10_43318509
		S10_4496021
		S10_3188708
		S10_4233912
		S10_2622974
		S10_1685946
		S10_9566182
		S10_6533512
		S10_7788816
		S10_414812
		S10_6741123
		S10_11711274
		S10_4058773
		S10_4470866
		S10_7059185
		S10_38125322
		S10_5469538
		S10_9441610
		S10_5518554
		S10_43625919
		S10_2623230
		S10_9559450
		S10_75758
		S10_7604394
		S10_1830665
		S10_5200237
		S10_582099
		S10_9880699
		S10_7873111
		S10_18062698
		S10_4178285
		S10_2774135
		S10_5964872
		S10_970833
		S10_5720157
		S10_761776
		S10_3575834
		S10_8200390
		S10_4458529
		S10_9048637
		S10_4460456
		S10_39940227
		S10_1529333
		S10_38387510
		S10_3530217
		S10_2900240
		S10_5201348
		S10_6840829
		S10_5039029
		S10_13628643
		S10_6375246
		S10_13480918
		S10_4258703
		S10_4262802
		S10_9044529
		S10_9680969
		S10_2624378

Catogoery	Total	SNPs
		S10_2383588
		S10_5210241
		S10_4311113
		S10_16362444
		S10_36782809
		S10_8964429
		S10_1938055
		S10_3753629
		S10_4216380
		S10_34622113
		S10_4293810
		S10_7107912
		S10_12315437
		S10_11774817
		S10_5957741
		S10_818266
		S10_725937
		S10_4167381
		S10_1803405
		S10_1792190
		S10_15439996
		S10_1928081
		S10_1488017
		S10_7103228
		S10_8655296
		S10_4232854
		S10_1954757
		S10_9519898
		S10_4395080
		S10_7433083
		S10_1135671
		S10_11808719
		S10_16043627
		S10_7201580
		S10_7171680
		S10_4346597
		S10_1799457
		S10_1171621
		S10_38750226
		S10_3455457
		S10_2366034
		S10_5158563
		S10_40093049
		S10_8977082
		S10_2383574
		S10_5054489
		S10_3956707
		S10_40003644
		S10_2772815
		S10_2534916
		S10_9660755
		S10_5212466
		S10_845898
		S10_3073245
		S10_5210352
		S10_2074894
		S10_7043786
		S10_3566747
		S10_8818725
		S10_9280235
		S10_4470865
		S10_4124382
		S10_12329578
		S10_4326976
		S10_7112607

Catogoery	Total	SNPs
		S10_40092962
		S10_43097427
		S10_10483289
		S10_9353281
		S10_924410
		S10_5834083
		S10_39791146
		S10_5960705
		S10_9680913
		S10_7604407
		S10_13825789
		S10_2455677
		S10_970972
		S10_2308547
		S10_7529174
		S10_3948319
		S10_4183518
		S10_3741187
		S10_7799762
		S10_9969608
		S10_1816120
		S10_1269995
		S10_2897403
		S10_7201587
		S10_11197145
		S10_5965717
		S10_10614507
		S10_40641672
		S10_719269
		S10_9352596
		S10_41305944
		S10_7112713
		S10_13705100
		S10_6933677
		S10_16173948
		S10_17540673
		S10_3245734
		S10_8200386
		S10_16969739
		S10_11829900
		S10_3686338
		S10_2518942
		S10_39982011
		S10_16177155
		S10_1491254
		S10_970133
		S10_4652526
		S10_8206070
		S10_1744527
		S10_8544408
		S10_9841677
		S10_34872958
		S10_5787645
		S10_9441609
		S10_7213889
		S10_312219
		S10_16916561
		S10_9294850
		S10_574530
		S10_6031304
		S10_44899763
		S10_11221833
		S10_7513433

Catogoery	Total	SNPs
		S10_965978
		S10_7460381
		S10_7934078
		S10_9660639
		S10_16969243
		S10_2661361
		S10_9048156
		S10_2624165
		S10_4185812
		S10_44897637
		S10_38500972
		S10_9052035
		S10_761736
		S10_1724631
		S10_7522670
		S10_7170702
		S10_2623036
		S10_7821427
		S10_10167350
		S10_3566867
		S10_11711933
		S10_7801764
		S10_4395060
		S10_3575323
		S10_5157835
		S10_41349263
		S10_12045182
		S10_39553345
		S10_560123
		S10_5957758
		S10_1767110
		S10_6668687
		S10_5957747
		S10_43585376
		S10_970969
		S10_15181822
		S10_44660182
		S10_3566868
		S10_689074
		S10_22062678
		S10_38965819
		S10_4335867
		S10_2889490
		S10_3951507
		S10_635100
		S10_17460563
		S10_10614501
		S10_3569818
		S10_1827794
		S10_6060153
		S10_8617192
		S10_7819801
		S10_38095375
		S10_15872865
		S10_761699
		S10_11830084
		S10_17958992
		S10_11713821
		S10_2784327
		S10_10222859
		S10_14169220
		S10_9571579
		S10_9864531
		S10_18003830

Catogoery	Total	SNPs
		S10_12045190
		S10_970402
		S10_157992
		S10_11003062
		S10_5210360
		S10_1132306
		S10_7604389
		S10_7201706
		S10_5551078
		S10_7112729
		S10_3168941
		S10_19641092
		S10_44897629
		S10_8750642
		S10_1777963
		S10_41314247
		S10_3948302
		S10_16406063
		S10_16914480
		S10_43625918
		S10_14679060
		S10_8750575
		S10_2025102
		S10_7791943
		S10_3686756
		S10_2383580
		S10_3569841
		S10_12824497
		S10_1266298
		S10_5903111
		S10_7692704
		S10_17848820
		S10_41656433
		S10_7201703
		S10_12647085
		S10_39791194
		S10_9048153
		S10_11374006
		S10_781023
		S10_5957742
		S10_7044474
		S10_5558516
		S10_10692538
		S10_36850914
		S10_181144
		S10_689097
		S10_1265945
		S10_23514414
		S10_761731
		S10_13707168
		S10_7171689
		S10_1724625
		S10_9048645
		S10_10167345
		S10_981915
		S10_1921714
		S10_4460408
		S10_10541708
		S10_16649648
		S10_14680349
		S10_678028
		S10_10051990
		S10_5175152
		S10_979141
		S10_4124038

Catogoery	Total	SNPs
		S10_8616239
		S10_10258610
		S10_9515761
		S10_10563873
		S10_7509330
		S10_11838285
		S10_7505775
		S10_15190882
		S10_6464134
		S10_8813696
		S10_1144481
		S10_6991533
		S10_6045765
		S10_8976514
		S10_9049688
		S10_7273533
		S10_7865165
		S10_12045189
		S10_2784327
		S10_9711442
		S10_3006783
		S10_10051971
		S10_1636698
		S10_7839731
		S10_15001167
		S10_7859272
		S10_18065124
		S10_4593013
		S10_8101054
		S10_7878445
		S10_35904324
		S10_3538355
		S10_9566942
		S10_1688449
		S10_970995
		S10_4746076
		S10_252318
		S10_7770213
		S10_5175156
		S10_386581
		S10_3927587
		S10_8818725
		S10_680636
		S10_40092967
		S10_962424
		S10_7860454
		S10_555074
		S10_4232897
		S10_6927299
		S10_5054561
		S10_5250313
		S10_6533239
		S10_21039299
		S10_10614688
		S10_312214
		S10_34339151
		S10_7918156
		S10_1803369
		S10_13046017
		S10_8813692
		S10_7692712
		S10_3097536
		S10_5171553
		S10_5210241
		S10_2680435

Catogoery	Total	SNPs
		S10_25333465
		S10_7692707
		S10_6164381
		S10_10692550
		S10_3927805
		S10_9519870
		S10_8206059
		S10_5157856
		S10_3547626
		S10_9254606
		S10_4226266
		S10_7604392
		S10_11830546
		S10_17092352
		S10_5212468
		S10_6818456
		S10_2025107
		S10_39982001
		S10_7166559
		S10_17515615
		S10_7519271
		S10_4053922
		S10_2025118
		S10_12648237
		S10_4053912
		S10_7352933
		S10_2772847
		S10_7043773
		S10_7158491
		S10_4470864
		S10_386174
		S10_25456301
		S10_16406102
		S10_4458466
		S10_4185170
		S10_9690430
		S10_2772827
		S10_8741695
		S10_18100057
		S10_12089692
		S10_291758
		S10_7559368
		S10_4549479
		S10_6107766
		S10_8206062
		S10_11064492
		S10_4460548
		S10_7201588
		S10_41353125
		S10_6808069
		S10_5158416
		S10_7454457
		S10_132816
		S10_9680923
		S10_6556491
		S10_13479637
		S10_13705147
		S10_4116988
		S10_17173333
		S10_6358208
		S10_4185840
		S10_4345370
		S10_10161075
		S10_1933123
		S10_9281388

Catogoery	Total	SNPs
		S10_10830725
		S10_16878952
		S10_318786
		S10_2626158
		S10_18068645
		S10_10132760
		S10_11852848
		S10_7877100
		S10_39791861
		S10_3932328
		S10_7708842
		S10_2362227
		S10_4764549
		S10_634957
		S10_1607519
		S10_11731514
		S10_970399
		S10_7821423
		S10_6639089
		S10_2397978
		S10_11113367
		S10_9044522
		S10_2485956
		S10_750376
		S10_8544397
		S10_2838398
		S10_13922748
		S10_8750578
		S10_7244524
		S10_634907
		S10_7819825
		S10_14798210
		S10_767974
		S10_17173927
		S10_2514618
		S10_6998929
		S10_6669400
		S10_41292034
		S10_970838
		S10_2366074
		S10_6375216
		S10_2804858
		S10_5777967
		S10_2960224
		S10_621916
		S10_4339941
		S10_8861926
		S10_9889661
		S10_4309213
		S10_9519907
		S10_2943351
		S10_2308585
		S10_17746660
		S10_1132530
		S10_4259012
		S10_13574161
		S10_40511895
		S10_1536217
		S10_12108998
		S10_2640803
		S10_7875372
		S10_7917724
		S10_412752
		S10_11713774
		S10_2025083

Catogoery	Total	SNPs
		S10_252467
		S10_7107913
		S10_4453827
		S10_4293938
		S10_45100746
		S10_689184
		S10_2507258
		S10_7093277
		S10_7769691
		S10_233998
		S10_13716848
		S10_13677798
		S10_10798980
		S10_12045188
		S10_2411560
		S10_4069165
		S10_7477945
		S10_18072488
		S10_7727170
		S10_38860683
		S10_9049687
		S10_2025097
		S10_6850812
		S10_6338776
		S10_8263508
		S10_2306859
		S10_767957
		S10_11065088
		S10_721321
		S10_7519775
		S10_2451466
		S10_15823167
		S10_7481141
		S10_10258678
		S10_2019887
		S10_4304188
		S10_7692719
		S10_970837
		S10_4745794
		S10_970999
		S10_4327008
		S10_9880713
		S10_4185809
		S10_5782343
		S10_439084
		S10_9577491
		S10_4604354
		S10_8696061
		S10_1129748
		S10_1799399
		S10_2623023
		S10_9809693
		S10_8264143
		S10_5951073
		S10_9441607
		S10_7488379
		S10_4692314
		S10_17172572
		S10_9236744
		S10_1587181
		S10_15181824
		S10_7750769
		S10_4058775
		S10_5212024
		S10_11387994

Catogoery	Total	SNPs
		S10_1709756
		S10_8111185
		S10_959253
		S10_15044559
		S10_41759244
		S10_18065098
		S10_13922794
		S10_33566271
		S10_721232
		S10_2506311
		S10_900892
		S10_2025088
		S10_86467
		S10_9880716
		S10_8977083
		S10_10690011
		S10_7522668
		S10_621914
		S10_86447
		S10_1720259
		S10_7158488
		S10_7918147
		S10_8575442
		S10_9375459
		S10_41838020
		S10_9880722
		S10_6028164
		S10_9460846
		S10_9044595
		S10_15908935
		S10_9811253
		S10_40502370
		S10_674364
		S10_1938487
		S10_4254921
		S10_5518576
		S10_40204351
		S10_7157504
		S10_6376671
		S10_23878270
		S10_10830715
		S10_6043145
		S10_5962678
		S10_765551
		S10_719073
		S10_40512576
		S10_9598041
		S10_11064621
		S10_8200389
		S10_2624300
		S10_6219295
		S10_6164142
		S10_7103225
		S10_1132440
		S10_842655
		S10_12089717
		S10_4323521
		S10_8279385
		S10_13483901
		S10_4252730
		S10_17848862
		S10_4230721
		S10_318791
		S10_2025119
		S10_970311

Catogoery	Total	SNPs
		S10_7112601
		S10_4199098
		S10_4080099
		S10_11197136
		S10_4262802
		S10_10223614
		S10_7352936
		S10_15050761
		S10_6130674
		S10_1733968
		S10_761775
		S10_10483314
		S10_43401572
		S10_2383576
		S10_40130153
		S10_2306880
		S10_725962
		S10_2661334
		S10_1733901
		S10_7944302
		S10_7460429
		S10_9660713
		S10_7166591
		S10_4339702
		S10_4109647
		S10_11006900
		S10_10798979
		S10_34188960
		S10_8750741
		S10_2025080
		S10_4602585
		S10_10317472
		S10_43586475
		S10_9559441
		S10_2431293
		S10_980970
		S10_6471640
		S10_4460547
		S10_1162694
		S10_41067642
		S10_370677
		S10_4395062
		S10_4109746
		S10_11003036
		S10_1132359
		S10_6818474
		S10_7750807
		S10_3827528
		S10_36445544
		S10_7752702
		S10_4602512
		S10_5048286
		S10_4053923
		S10_9302870
		S10_4453828
		S10_7820127
		S10_4460434
		S10_312298
		S10_3169867
		S10_8754421
		S10_5201426
		S10_4602570
		S10_9864543
		S10_1560190
		S10_7352934

Catogoery	Total	SNPs
		S10_4621209
		S10_13032464
		S10_44533575
		S10_18070432
		S10_2451191
		S10_4216436
		S10_9987016
		S10_7545206
		S10_2383587
		S10_12320075
		S10_13574163
		S10_1170329
		S10_13831254
		S10_6998934
		S10_4060137
		S10_43023628
		S10_16969799
		S10_2359780
		S10_13046016
		S10_6620384
		S10_11211307
		S10_2266403
		S10_4231916
		S10_5973850
		S10_4232937
		S10_3927802
		S10_7112817
		S10_8977079
		S10_7678856
		S10_7166590
		S10_3575417
		S10_7157163
		S10_15910988
		S10_3188699
		S10_9880721
		S10_4080102
		S10_1687509
		S10_9711446
		S10_6219281
		S10_16413766
		S10_15001175
		S10_4116992
		S10_8921555
		S10_4654315
		S10_44119386
		S10_10797567
		S10_9049701
		S10_9282679
		S10_318792
		S10_3265766
		S10_11452003
		S10_9515797
		S10_7519280
		S10_40691947
		S10_1769613
		S10_3932380
		S10_9750273
		S10_16916112
		S10_1280564
		S10_1724621
		S10_9167322
		S10_1964819
		S10_7844288
		S10_10030814
		S10_40641720

Catogoery	Total	SNPs
		S10_2108172
		S10_7877111
		S10_38965801
		S10_5210309
		S10_2855653
		S10_15908934
		S10_1954760
		S10_7619738
		S10_4606294
		S10_11221362
		S10_39911237
		S10_11829941
		S10_7877058
		S10_44304202
		S10_11829851
		S10_1189954
		S10_7240054
		S10_11004993
		S10_12457200
		S10_7201705
		S10_2025105
		S10_252444
		S10_43401562
		S10_4328072
		S10_2570795
		S10_7524898
		S10_16047535
		S10_7821422
		S10_8754253
		S10_19733465
		S10_2114661
		S10_4612271
		S10_768130
		S10_43586474
		S10_6464160
		S10_1675358
		S10_6001944
		S10_1921429
		S10_828159
		S10_589327
		S10_4293849
		S10_2025095
		S10_8975952
		S10_12045122
		S10_9598097
		S10_21140574
		S10_459703
		S10_3948316
		S10_2178888
		S10_44897539
		S10_3089002
		S10_5777989
		S10_4233977
		S10_1170733
		S10_386594
		S10_5210241
		S10_7604423
		S10_4233803
		S10_2411561
		S10_16969807
		S10_5158560
		S10_725931
		S10_589231
		S10_16174523
		S10_41577330

Catogoery	Total	SNPs
		S10_7433086
		S10_7604408
		S10_6359497
		S10_13577892
		S10_10830477
		S10_4233830
		S10_1830657
		S10_40837633
		S10_2514619
		S10_7352941
		S10_7201584
		S10_4460433
		S10_5198881
		S10_1170332
		S10_5818167
		S10_5720148
		S10_13923379
		S10_9660844
		S10_970993
		S10_7171647
		S10_1529450
		S10_4298654
		S10_13703716
		S10_11452002
		S10_12645954
		S10_4706287
		S10_6086862
		S10_1954755
		S10_1560220
		S10_2466098
		S10_43097988
		S10_2960227
		S10_7692716
		S10_20392358
		S10_6056866
		S10_15183774
		S10_7945558
		S10_1767111
		S10_11064963
		S10_670684
		S10_874160
		S10_38726588
		S10_9686971
		S10_8551202
		S10_15908929
		S10_9516020
		S10_4453709
		S10_4193964
		S10_7273525
		S10_1855104
		S10_10161164
		S10_3569838
		S10_4185190
		S10_9051895
		S10_4226288
		S10_43320860
		S10_684433
		S10_7201543
		S10_13706862
		S10_3291339
		S10_2359881
		S10_9750535
		S10_6851055
		S10_4604779
		S10_17173318

Catogoery	Total	SNPs
		S10_11197144
		S10_4118102
		S10_5674135
		S10_423831
		S10_1170298
		S10_2575944
		S10_457905
		S10_7503738
		S10_970380
		S10_10540267
GWAS signif SNP J2614/RSG04008	2296	S5_4923530
		S3_57321183
		S2_61586246
		S4_62936589
		S5_68758354
		S2_8937547
		S2_73739698
		S1_12563734
		S1_77343696
		S2_75342750
		S7_5963581
		S4_5561087
		S2_58875658
		S6_359052
		S5_63844658
		S6_52008494
		S1_79990589
		S6_53714413
		S1_3329758
		S3_72800609
		S1_17532284
		S6_25666726
		S6_52092904
		S7_61384137
		S5_6972135
		S6_52092913
		S6_51403978
		S6_1047672
		S3_72071460
		S1_14448627
		S4_3715066
		S1_6785913
		S3_15620595
		S7_1522183
		S3_3622828
		S6_17986086
		S7_12071810
		S3_73702647
		S9_51434058
		S5_11055457
		S7_56160907
		S7_64742874
		S2_59655484
		S1_78752790
		S6_36255558
		S3_70860889
		S3_73217029
		S7_8397336
		S6_52485896
		S6_53506008
		S5_57276203
		S2_57449109
		S4_16066479
		S1_56033556
		S2_65784818

Catogoery	Total	SNPs
		S2_59751793
		S1_14330617
		S1_18971221
		S1_67294739
		S6_2547431
		S7_61149950
		S3_72123621
		S2_55211584
		S2_6008867
		S1_13095759
		S2_63290523
		S4_36263190
		S1_12249706
		S3_3997275
		S7_61149952
		S3_56160266
		S9_54330774
		S5_8735555
		S3_74099136
		S6_54221535
		S9_57225685
		S5_7840232
		S9_57247235
		S1_9898134
		S6_1520148
		S4_62936585
		S8_51762013
		S2_73888705
		S3_16168612
		S8_45793219
		S2_63290511
		S5_12365060
		S1_77343689
		S6_51307294
		S1_71861477
		S8_51294410
		S1_56549114
		S1_77214030
		S6_47800571
		S8_40730445
		S1_71707259
		S6_6977745
		S9_8117469
		S6_7029530
		S9_58504227
		S6_38162033
		S2_65359832
		S9_41543597
		S5_62499919
		S2_66830583
		S4_50348958
		S4_3715085
		S1_29271525
		S2_10906987
		S1_16484731
		S2_52993647
		S9_57069088
		S5_9537142
		S9_59322668
		S4_10709836
		S7_63084011
		S1_10462682
		S5_1795586
		S4_58490586
		S3_16332253

Catogoery	Total	SNPs
		S8_1356088
		S1_19349551
		S1_66310194
		S1_11764852
		S3_73055176
		S6_31747114
		S7_63563527
		S1_603
		S4_51106787
		S6_2667402
		S1_71936174
		S9_57119965
		S1_3329754
		S1_56568091
		S2_61841943
		S7_5963638
		S7_7190488
		S4_55546064
		S5_62686361
		S2_66492060
		S2_13505785
		S2_65718904
		S2_69200414
		S3_55801294
		S6_1454193
		S3_74099137
		S4_63410059
		S1_10565425
		S9_58684354
		S5_9053126
		S4_53752969
		S1_11791521
		S8_1216720
		S2_61686625
		S2_61586256
		S8_2063869
		S5_4000601
		S8_32755312
		S1_14854881
		S7_59417478
		S3_66699298
		S1_73413882
		S9_41577016
		S4_7277550
		S3_59746640
		S5_67670582
		S8_4209912
		S8_60944466
		S1_71371449
		S1_17532290
		S5_4509059
		S9_57780853
		S5_5262919
		S2_75594134
		S3_73087592
		S9_58949348
		S2_4554749
		S7_8905066
		S9_6802789
		S1_56033554
		S2_72260776
		S6_1454237
		S1_13427114
		S2_63290520
		S5_614663

Catogoery	Total	SNPs
		S3_73861174
		S7_57819725
		S9_58295230
		S1_78715461
		S4_52118628
		S2_12894456
		S1_72567739
		S7_6974806
		S2_30234114
		S9_54884966
		S1_29334826
		S1_72692082
		S9_6802790
		S8_2051830
		S3_6605420
		S1_12563739
		S9_54857339
		S4_67214412
		S1_70529622
		S1_68883113
		S5_11779477
		S8_46362742
		S3_59503609
		S6_17520983
		S7_1522180
		S2_8878734
		S4_63395561
		S9_57752751
		S9_47006102
		S1_68883124
		S2_6245657
		S2_66412795
		S5_11472805
		S1_4581926
		S5_10493197
		S1_56549114
		S2_41150065
		S5_8696398
		S3_57759730
		S1_11003138
		S2_59249432
		S9_57666338
		S2_58428674
		S3_70392133
		S5_62492964
		S9_58748351
		S9_52939504
		S9_53164772
		S1_13188261
		S7_5171847
		S1_14462372
		S1_72611646
		S1_56033565
		S1_12563732
		S4_13036200
		S1_12945016
		S1_57202401
		S4_7982383
		S9_53867528
		S4_67214412
		S9_57911514
		S1_12627604
		S1_79529450
		S6_37639790
		S2_425915

Catogoery	Total	SNPs
		S9_375423
		S6_1758754
		S2_66074828
		S3_55801558
		S1_12160253
		S8_6185103
		S4_62936040
		S1_12563723
		S1_68883141
		S6_47626871
		S3_71874658
		S1_74876556
		S1_56033561
		S1_12781217
		S9_57119962
		S1_74876553
		S7_3391760
		S8_9637993
		S7_57745824
		S6_52707096
		S4_43825975
		S3_54714971
		S1_12227115
		S7_2112786
		S2_58614110
		S3_70976301
		S1_72166164
		S6_1309867
		S9_55067203
		S5_9053130
		S4_55553820
		S9_163434
		S7_5417560
		S3_13494206
		S3_73907824
		S9_52923065
		S6_18915692
		S9_55004994
		S9_47006126
		S9_58757394
		S9_153356
		S5_7192179
		S9_41678382
		S3_72505764
		S6_54168531
		S4_62936596
		S6_37366196
		S4_55547370
		S2_69572060
		S1_17106086
		S1_71511929
		S2_63290514
		S2_69173961
		S4_60584591
		S7_592795
		S2_53473251
		S1_11686529
		S4_58259891
		S9_55551966
		S7_63563543
		S3_59880946
		S5_9053125
		S2_60432744
		S4_62988514
		S2_62095147

Catogoery	Total	SNPs
		S8_62009189
		S1_79016620
		S1_73776874
		S7_64079510
		S8_6158577
		S9_56572848
		S7_5572054
		S3_72512315
		S7_5872814
		S4_51277634
		S6_3915058
		S1_11125364
		S1_50390544
		S9_4373590
		S6_1003772
		S2_35154563
		S7_2939252
		S9_53899689
		S1_27385834
		S5_20196453
		S7_3623922
		S1_12932509
		S5_3193004
		S9_57911513
		S9_57657251
		S1_79402147
		S6_3643098
		S6_52092883
		S4_51277651
		S4_53866167
		S4_7468114
		S2_66412806
		S6_45543014
		S2_67330792
		S2_3807228
		S6_50847381
		S3_3622850
		S6_52599267
		S5_3876828
		S1_78106488
		S2_60533933
		S4_53708540
		S7_57892847
		S2_18921102
		S4_55454617
		S1_19349545
		S2_75331009
		S1_56327742
		S5_8695135
		S2_63693006
		S2_59249439
		S1_67485491
		S6_47626874
		S2_57258429
		S1_6234392
		S7_1356501
		S7_59404484
		S5_6781664
		S2_63803728
		S3_48669583
		S2_61923353
		S2_72260818
		S5_8604908
		S1_79619171
		S1_7248001

**Catogoery**	**Total**	**SNPs**
		S7_17261664
		S8_2374641
		S4_63410079
		S4_51774538
		S8_1172689
		S9_2238350
		S9_43155530
		S5_2692983
		S9_57780832
		S4_3715095
		S1_12627739
		S3_62531577
		S1_16387827
		S9_59159043
		S1_17818500
		S2_50985085
		S9_2251160
		S4_7468129
		S4_56611095
		S9_10724823
		S8_53594816
		S5_38827168
		S1_3351629
		S9_57123473
		S8_2615032
		S6_1783595
		S2_58239285
		S6_41604283
		S3_74310760
		S4_2234744
		S2_66412792
		S7_6761736
		S2_71490218
		S2_12346371
		S6_52294116
		S1_13968190
		S5_67718081
		S1_72772586
		S3_62531572
		S1_11151142
		S5_4499193
		S2_64550312
		S2_66316757
		S4_41454640
		S1_14869710
		S3_73145496
		S1_11791541
		S6_6657915
		S1_12030980
		S6_49689735
		S1_78711570
		S2_25873671
		S7_63134774
		S7_62118605
		S1_14448632
		S9_3616579
		S1_72940300
		S1_16432036
		S1_75765696
		S4_10427993
		S6_45176024
		S1_11431224
		S8_504185
		S4_67214412
		S6_39651119

Catogoery	Total	SNPs
		S1_72691344
		S9_56945733
		S2_63290537
		S6_1454142
		S3_6754738
		S2_6888062
		S4_50669859
		S3_53324796
		S1_45942285
		S9_56520725
		S3_59503604
		S3_68055530
		S7_62390907
		S1_71360475
		S3_57481362
		S6_1460053
		S1_64086236
		S3_72001410
		S4_62936586
		S4_66254474
		S6_53506011
		S1_72940335
		S2_12190474
		S3_73702640
		S4_19288511
		S1_79990590
		S4_5015976
		S3_72989113
		S5_46702355
		S7_5383474
		S4_46972445
		S9_57833860
		S2_73739659
		S9_57914426
		S5_3829915
		S6_3915055
		S1_56033564
		S5_8994776
		S8_51575507
		S1_16731242
		S6_49461190
		S2_74389504
		S6_58035596
		S1_26596842
		S9_55031132
		S7_61271950
		S4_41454639
		S1_67332284
		S2_61927663
		S1_72302128
		S4_51771351
		S9_519098
		S2_66956243
		S2_4334298
		S9_56524133
		S1_12596401
		S5_4499195
		S5_38471416
		S8_1066082
		S9_2239826
		S1_16745318
		S1_14672796
		S3_21774427
		S6_1306617
		S6_46819431

Catogoery	Total	SNPs
		S1_80482739
		S7_65081304
		S4_1238210
		S7_5171839
		S6_37639735
		S2_75594135
		S5_8604896
		S2_55292147
		S5_10195816
		S7_5963607
		S1_17532282
		S2_61233514
		S3_70976306
		S7_57417001
		S6_52289454
		S6_58117354
		S4_51199590
		S1_20258213
		S2_63290533
		S6_30816124
		S2_6277727
		S1_55923588
		S1_12338566
		S5_8668429
		S4_41454636
		S2_13459050
		S9_57812258
		S1_72949911
		S2_6523542
		S1_12967675
		S2_61686229
		S1_78510453
		S6_953196
		S2_6245664
		S7_63067948
		S9_59173027
		S2_36306432
		S2_59722587
		S6_822538
		S2_57848126
		S3_4690521
		S6_49441807
		S8_44669846
		S6_52485895
		S9_59291140
		S1_77343698
		S9_58684354
		S5_67529207
		S2_60709429
		S9_12066581
		S1_56527271
		S2_8661367
		S8_49770652
		S1_16387850
		S9_58332115
		S1_3105950
		S2_65877733
		S5_2278268
		S6_15382059
		S2_76430891
		S6_8919203
		S2_75792053
		S9_51830298
		S2_6236103
		S2_76430887

Catogoery	Total	SNPs
		S1_18079294
		S6_18731918
		S6_1587039
		S6_53506025
		S1_14330622
		S3_59746639
		S1_55062451
		S7_4785414
		S2_9512992
		S1_63389970
		S2_61246561
		S9_513571
		S1_78180781
		S3_56070788
		S6_16802520
		S6_47927895
		S6_4090558
		S5_6024239
		S4_67119244
		S8_53829081
		S4_67214412
		S9_56594405
		S4_8916481
		S7_61414539
		S8_56113697
		S1_12968211
		S5_3016226
		S3_56656490
		S4_58459316
		S5_2845565
		S3_7416112
		S2_61233402
		S1_7388314
		S1_59985155
		S3_57954741
		S2_15077622
		S1_1880194
		S6_42084968
		S2_64335542
		S6_17559082
		S1_17532285
		S4_51957270
		S3_66660389
		S1_15880765
		S9_55626876
		S1_11786106
		S1_14162189
		S5_437471
		S5_6866425
		S4_62936581
		S2_61549003
		S9_57911522
		S6_3525948
		S1_19184687
		S6_2547474
		S1_28470926
		S2_72273733
		S2_76908521
		S3_53260490
		S3_60767367
		S6_46569258
		S1_73644317
		S1_68772784
		S4_55547289
		S1_8998667

Catogoery	Total	SNPs
		S6_46734881
		S9_57051565
		S7_1356530
		S4_9770407
		S5_8695893
		S6_17356361
		S1_67190094
		S2_65854375
		S9_58508533
		S3_53324822
		S4_43064226
		S4_13143449
		S9_52923075
		S1_5666314
		S7_1522170
		S9_57925883
		S1_50830084
		S4_53162965
		S5_67767099
		S4_56427923
		S3_56160263
		S4_62936592
		S5_6630988
		S6_38113153
		S1_56225727
		S6_41266913
		S1_72692099
		S5_9053129
		S5_35518301
		S3_53520780
		S9_375417
		S6_52850900
		S9_57681486
		S4_1216332
		S6_51403993
		S9_57540793
		S1_26888112
		S1_9142676
		S3_69967110
		S2_61733987
		S1_12595162
		S2_61686194
		S1_19104755
		S2_57728708
		S2_12686896
		S8_1501432
		S5_66487518
		S1_1842653
		S4_34662222
		#N/A
		S2_38294461
		S2_18307240
		S9_56943782
		S8_56539807
		S9_59156843
		S9_57905432
		S8_60246781
		S4_67214412
		S1_3464574
		S4_56427893
		S4_1373052
		S8_48988557
		S9_52908858
		S1_51240322
		S2_75792059

Catogoery	Total	SNPs
		S2_67620075
		S4_13143466
		S3_69207670
		S1_72927335
		S3_62196944
		S7_4559643
		S2_18569274
		S2_62121657
		S3_2191124
		S9_57745849
		S1_71553878
		S7_570062
		S1_72611660
		S3_70976276
		S7_62467421
		S9_45915919
		S1_57676176
		S3_53388973
		S2_13459053
		S2_4555276
		S9_55566640
		S9_58973752
		S6_26788359
		S2_49662976
		S7_2112747
		S4_68098051
		S1_63672052
		S3_60581809
		S1_12967786
		S1_9664234
		S3_6759948
		S3_60767342
		S1_72938517
		S6_1734778
		S9_2886913
		S5_12536072
		S7_12790226
		S1_49256106
		S9_54908187
		S4_49546333
		S1_3329755
		S9_58728008
		S1_72949915
		S8_60349308
		S6_809010
		S4_43752393
		S6_1374319
		S1_12967667
		S3_2047847
		S9_3933616
		S9_57657830
		S3_56150261
		S5_4509065
		S1_68883084
		S9_54325003
		S9_47241963
		S2_61548986
		S8_38329974
		S3_52007842
		S8_53829072
		S9_56101938
		S8_40730391
		S1_14448631
		S1_13469184
		S9_153350

Catogoery	Total	SNPs
		S2_67206475
		S4_16050848
		S9_52923067
		S2_63803722
		S4_43199868
		S4_50646022
		S1_64365057
		S4_36166378
		S1_56033559
		S1_10739071
		S7_60263992
		S9_56535023
		S3_72913128
		S9_57792168
		S1_50830102
		S2_61686233
		S3_73087561
		S7_57211993
		S6_41600509
		S9_57641803
		S6_51800735
		S7_5154827
		S1_14462383
		S4_45490523
		S5_1932855
		S6_52485898
		S1_12106664
		S6_40434431
		S9_58114502
		S5_11779471
		S5_17390774
		S9_53238030
		S2_63078122
		S9_59371922
		S1_67392250
		S9_57811319
		S6_2193097
		S9_56945723
		S8_1066081
		S4_62936063
		S9_57670282
		S4_54055170
		S3_72849712
		S2_12686875
		S7_8909175
		S3_72917312
		S2_66412846
		S3_73217103
		S9_54080354
		S5_40360109
		S1_14448626
		S9_4652966
		S1_22746381
		S2_7342926
		S3_5146441
		S1_57676228
		S5_66430109
		S4_22432524
		S2_61549011
		S7_55979632
		S6_38161717
		S7_58668167
		S3_73040860
		S9_44446459
		S2_63450765

Catogoery	Total	SNPs
		S1_56074384
		S6_35745026
		S7_63623326
		S1_7769551
		S4_51199593
		S4_6805481
		S3_53388970
		S1_12563738
		S6_52092901
		S1_14285586
		S7_8637676
		S3_3622836
		S2_66078977
		S5_66909528
		S6_44560660
		S1_14690005
		S1_73644308
		S2_73740883
		S2_61233514
		S9_57911518
		S8_51315455
		S6_2598827
		S3_35819697
		S3_52182545
		S8_1577433
		S3_74178521
		S4_56654691
		S2_69130242
		S9_58177614
		S8_1993659
		S8_6158575
		S5_61681577
		S2_60406949
		S9_53565698
		S1_17285880
		S1_57665002
		S3_57369757
		S5_18528466
		S7_930915
		S2_61548989
		S4_62936588
		S7_2083276
		S8_2615037
		S3_5626292
		S1_74323696
		S7_5154331
		S3_15946969
		S9_58757504
		S2_58239206
		S1_67485484
		S2_65686094
		S6_15331126
		S3_73425627
		S1_10793791
		S9_59312050
		S2_7531999
		S1_13157764
		S5_12536085
		S7_57745836
		S2_57258426
		S9_53818841
		S4_67214412
		S1_50894412
		S1_2458035
		S8_1325690

Catogoery	Total	SNPs
		S2_6612036
		S1_17757376
		S6_53273004
		S2_66958081
		S5_10493200
		S9_2239829
		S5_7242271
		S1_66004503
		S2_6410129
		S4_50669857
		S6_36408670
		S1_26893025
		S9_55626875
		S3_72849707
		S7_60709314
		S9_57139944
		S5_15248013
		S8_49770634
		S6_41825703
		S8_1699232
		S7_4438947
		S1_14285587
		S4_56654900
		S1_75624748
		S2_69237155
		S8_16196648
		S7_7588548
		S6_51990210
		S7_62069222
		S3_61040495
		S4_25292491
		S8_60943169
		S5_3134227
		S6_46819386
		S1_67065341
		S1_14690013
		S2_73037539
		S2_59655550
		S5_9537136
		S6_1332870
		S1_15084897
		S6_31040630
		S1_78180778
		S6_1306514
		S2_62095251
		S1_64086235
		S3_73609149
		S1_78234141
		S4_56557333
		S2_61549004
		S6_58762171
		S3_68055528
		S9_56530403
		S8_1642168
		S3_59503603
		S3_73014267
		S1_65668486
		S5_61929454
		S2_63290512
		S3_72440333
		S6_1047745
		S4_56522702
		S2_65386069
		S1_19184682
		S6_53592874

Catogoery	Total	SNPs
		S4_7468111
		S5_2219901
		S4_14247808
		S2_60856616
		S1_16387838
		S2_68505778
		S1_12227113
		S6_13715430
		S1_12922040
		S1_22035471
		S4_9443882
		S9_57069120
		S3_72504817
		S6_52884986
		S4_66125410
		S1_79540230
		S8_53508627
		S4_68098036
		S2_65357280
		S2_63692439
		S4_40071868
		S5_2968842
		S9_6615435
		S1_48835921
		S1_1795296
		S1_72171210
		S9_41678381
		S8_1252165
		S1_12415971
		S9_58867145
		S2_4555285
		S8_1066080
		S3_56160181
		S9_59318053
		S3_63388746
		S9_59311585
		S6_29130176
		S5_3626674
		S1_12997479
		S6_47626872
		S5_7006744
		S2_199703
		S5_68963917
		S9_55969226
		S7_6976847
		S1_63389994
		S3_56070783
		S9_375406
		S2_73412146
		S1_28195074
		S8_53626899
		S9_10729664
		S6_2547468
		S1_71707261
		S6_41922332
		S1_79195214
		S2_66916116
		S3_73337352
		S5_7295411
		S7_4438946
		S1_18901381
		S5_10759631
		S1_72691349
		S9_57216854
		S9_55067209

Catogoery	Total	SNPs
		S9_5767929
		S4_51460491
		S9_42663390
		S2_61873327
		S1_55062453
		S8_15299927
		S2_62095158
		S1_58937781
		S4_7468105
		S4_63395529
		S2_66958051
		S2_77444031
		S1_62723977
		S1_18315761
		S7_410940
		S1_63226451
		S3_64907616
		S4_6756630
		S8_1325687
		S6_51539086
		S1_10087988
		S9_53527986
		S7_61149944
		S1_7576799
		S4_56427895
		S2_63290516
		S1_12921087
		S7_5963635
		S7_1522174
		S2_63803729
		S9_57776578
		S6_15331164
		S6_2991268
		S1_11625328
		S3_73861180
		S2_4334291
		S1_57490044
		S9_57670230
		S3_70253127
		S1_4063045
		S3_19317784
		S3_54823475
		S6_45184532
		S9_58541198
		S2_4554748
		S2_62117918
		S5_7018899
		S1_71731573
		S3_71429118
		S5_61921241
		S9_52923066
		S9_57540804
		S4_52118634
		S8_1642171
		S6_41266904
		S9_57947937
		S9_47006103
		S9_57787432
		S2_65784827
		S1_65668476
		S3_72140617
		S1_2717923
		S1_12627779
		S1_62825493
		S2_64391996

Catogoery	Total	SNPs
		S8_43465153
		S8_15299963
		S7_807355
		S7_61769757
		S7_8905054
		S8_50617053
		S5_9550350
		S3_74099133
		S3_4760405
		S6_304009
		S2_62631187
		S1_3464576
		S1_14870012
		S9_54583637
		S9_2886888
		S9_57670672
		S3_45987130
		S5_9181090
		S5_67670586
		S9_54908192
		S2_62703717
		S6_1306527
		S1_57383718
		S1_3329763
		S1_12122950
		S9_44637848
		S2_61549005
		S6_48651000
		S9_44414073
		S3_5640669
		S4_9770406
		S2_61266191
		S2_66958078
		S9_57792171
		S2_67205757
		S8_60944465
		S1_78180782
		S2_54461667
		S8_33210795
		S8_42774320
		S9_57811310
		S1_72205632
		S1_65257392
		S1_72567734
		S1_12095456
		S5_62173070
		S1_1902640
		S3_72071455
		S1_12959162
		S2_67899865
		S3_69123818
		S2_63290526
		S2_67977203
		S1_3329764
		S4_13143461
		S1_12967696
		S5_63307674
		S1_20338764
		S4_46972443
		S1_73866786
		S1_71606027
		S1_72949963
		S9_58949348
		S4_2645020
		S9_53172825

Catogoery	Total	SNPs
		S4_20672814
		S1_68777771
		S1_77343699
		S1_78200697
		S4_36166392
		S1_15880754
		S8_61590665
		S4_68098045
		S3_59492624
		S1_14462377
		S6_15331153
		S6_6657907
		S9_2233856
		S5_4509074
		S6_52891701
		S3_56160265
		S6_19400188
		S5_67670581
		S6_49441808
		S3_54967398
		S4_9328122
		S3_59573293
		S3_72849706
		S4_56610968
		S1_6234373
		S1_78395767
		S9_58670296
		S3_5137603
		S2_4863239
		S7_7029074
		S5_4560293
		S2_63692993
		S7_5183992
		S3_55801557
		S3_1945637
		S7_4438987
		S2_29767393
		S1_75394963
		S2_76908527
		S1_74323697
		S4_55546078
		S5_8695103
		S2_29767401
		S1_14448628
		S1_58937838
		S6_58767277
		S2_55292148
		S4_56615273
		S2_6277742
		S2_9370623
		S5_12536061
		S2_66492108
		S7_60264001
		S9_57065264
		S3_58878048
		S1_53772305
		S2_10001930
		S9_57529022
		S9_55883070
		S9_41678371
		S3_69637559
		S4_55454594
		S9_57780833
		S5_11779444
		S1_19244209

Catogoery	Total	SNPs
		S2_61586244
		S6_46734869
		S4_56427898
		S1_12160257
		S4_39654579
		S2_37434253
		S6_53064987
		S7_6745922
		S4_13143468
		S1_31874208
		#N/A
		S5_6024243
		S2_126519
		S1_14876334
		S1_65257377
		S1_76661685
		S9_58291473
		S4_62935704
		S6_17356403
		S7_60260045
		S1_18315751
		S1_72692088
		S9_519082
		S1_14448619
		S6_14556770
		S9_57904462
		S2_61233514
		S5_62729427
		S3_66631238
		S4_56428568
		S5_10493201
		S8_60140244
		S1_19017797
		S2_47204245
		S9_58634609
		S8_5477050
		S3_61378959
		S2_63195176
		S4_15095692
		S5_614669
		S9_58702178
		S6_334458
		S3_74152972
		S1_50451128
		S8_61581004
		S1_13096036
		S7_14482605
		#N/A
		S2_57264322
		S7_5963604
		S8_1993448
		S3_73759500
		S8_55846613
		S1_63965794
		S6_2547476
		S5_7351468
		S1_68883119
		S1_65740630
		S1_14876291
		S9_53930960
		S4_4553845
		S3_56160181
		S1_65257422
		S3_72087844
		S5_68031054

Catogoery	Total	SNPs
		S1_7222753
		S6_2547479
		S4_39876042
		S4_62936583
		S6_2683804
		S6_53154736
		S5_62492944
		S4_1337768
		S2_6888067
		S2_14775620
		S3_70976292
		S9_55067234
		S1_80344898
		S2_66916115
		S1_63543649
		S5_7267098
		S1_76661673
		S9_57069125
		S3_73181134
		S9_57914531
		S8_15337904
		S1_8931365
		S9_54908193
		S3_5041711
		S6_26282267
		S9_58472760
		S5_3865103
		S1_79149961
		S2_6009965
		S9_56943758
		S4_9770365
		S3_48669557
		S7_65085167
		S1_48835914
		S5_10814315
		S6_14537959
		S5_7641620
		S8_1197740
		S3_1945682
		S5_10354509
		S9_57752787
		S6_358918
		S2_73740888
		S6_40219350
		S2_61549010
		S5_8668419
		S6_72442
		S6_6458325
		S1_13376646
		S7_4526800
		S1_64993130
		S4_41454637
		S3_52182580
		S1_74359940
		S3_56160257
		S1_19180066
		S9_43336956
		S2_13809634
		S2_49669484
		S1_75039461
		S3_72140613
		S4_62936061
		S5_7283810
		S1_16731272
		S9_55067227

Catogoery	Total	SNPs
		S2_63290527
		S1_65740609
		S2_76430889
		S6_31747106
		S9_57804067
		S3_52417141
		S4_51774534
		S1_72691345
		S3_56160181
		S8_1066079
		S3_74099129
		S7_5416531
		S8_59748647
		S2_12894427
		S1_75765686
		S6_6293491
		S5_3038898
		S9_47050016
		S6_48659560
		S3_4459081
		S9_56594417
		S9_55869446
		S3_73362984
		S4_67092863
		S7_59417500
		S9_58748322
		S2_47590004
		S6_26788357
		S4_63395536
		S6_28730716
		S8_1066083
		S1_11151144
		S4_24739885
		S9_52923070
		S4_53811522
		S3_72149527
		S9_6802694
		S3_52557257
		S2_30234092
		S1_72938515
		S1_28200982
		S1_68772784
		S1_80576324
		S5_15311867
		S7_42752212
		S3_56150264
		S1_13725150
		S6_1520152
		S1_79990584
		S2_76430893
		S9_55492019
		S7_61414557
		S2_6042431
		S6_52777810
		S3_73329458
		S9_57545053
		S7_5980283
		S6_18806973
		S1_72949913
		S2_25873657
		S7_5207452
		S1_80482740
		S7_6974678
		S9_56549681
		S3_52182585

Catogoery	Total	SNPs
		S8_16865003
		S4_1659391
		S3_73861183
		S5_62509203
		S9_41614962
		S3_59501629
		S1_8998674
		S5_62499915
		S1_57111668
		S5_7721956
		S5_1795590
		S2_57834823
		S9_374484
		S3_66606594
		S1_15907796
		S9_10261847
		S9_53930966
		S4_54055164
		S3_47886098
		S6_1460052
		S6_38461551
		S1_13384592
		S5_7018926
		S7_1522177
		S9_56943776
		S8_513629
		S5_12857271
		S2_59751803
		S7_3623940
		S1_12564742
		S9_57781398
		S1_78161599
		S8_1839524
		S2_55524388
		S7_7872315
		S9_2941419
		S4_51839386
		S7_64024491
		S8_60943171
		S6_53187236
		S1_7222675
		S8_51761985
		S7_57803343
		S1_12997445
		S1_13312936
		S7_12260910
		S7_57848081
		S8_1839524
		S1_14689984
		S1_12627796
		S6_46791454
		S2_57258389
		S1_79576377
		S2_18309037
		S1_56074371
		S1_68578501
		S9_56507227
		S8_60943177
		S1_53772311
		S6_38161730
		S9_58841852
		S4_13143469
		S8_1252126
		S3_73861181
		S4_39246023

Catogoery	Total	SNPs
		S9_58727998
		S2_9780495
		S3_50563646
		S1_77343690
		S4_56427899
		S4_62935694
		S5_66965812
		S8_37668684
		S2_63450759
		S9_56943756
		S1_12968168
		S2_58182637
		S9_47241922
		S6_8919203
		S3_72321370
		S2_58008723
		S3_71411263
		S3_70200633
		S3_59693387
		S2_63450777
		S2_63182511
		S9_54908190
		S4_53842243
		S9_6802694
		S1_17106117
		S2_64464930
		S2_14406452
		S4_6901834
		S6_52087290
		S2_8887713
		S9_153348
		S6_43554708
		S2_6523564
		S6_49740177
		S6_48682774
		S1_75394964
		S6_47954090
		S7_538528
		S1_56735641
		S2_61548987
		S1_74876549
		S2_40623466
		S3_71850604
		S1_75427740
		S2_52997657
		S3_68205650
		S5_437471
		S5_11891529
		S1_19184686
		S1_12143895
		S2_57558184
		S2_66412842
		S6_2547432
		S3_73881401
		S8_1558734
		S1_68772784
		S4_6415429
		S9_57756001
		S4_55584592
		S1_15880763
		S6_29130191
		S5_2278291
		S1_19718165
		S1_66991835
		S1_54333136

Catogoery	Total	SNPs
		S2_58614106
		S6_39672724
		S1_3329757
		S3_5146437
		S2_16009255
		S2_64335513
		S2_71486250
		S2_40651428
		S8_51575493
		S6_47952360
		S7_38780634
		S1_63226451
		S2_10821401
		S2_61537476
		S2_75220306
		S1_67950145
		S4_24546731
		S1_18315763
		S4_27569225
		S9_10386294
		S4_68098053
		S8_5441996
		S3_72268827
		S1_12563735
		S3_55908669
		S3_56276031
		S8_49194390
		S6_1339919
		S7_59417502
		S7_63623330
		S9_40815469
		S3_66676550
		S6_34041171
		S1_50787051
		S1_20696637
		S4_62935703
		S9_55492020
		S9_6802788
		S4_51774564
		S1_21469663
		S4_67712021
		S6_48121146
		S3_60581801
		S7_8397340
		S1_71363969
		S1_12564526
		S1_7222657
		S1_67485490
		S1_13892084
		S3_5417977
		S4_42822700
		S5_2669059
		S9_54908173
		S1_16647171
		S6_51307249
		S9_56943773
		S2_75333122
		S5_50504935
		S9_1258977
		S9_59207864
		S9_374483
		S8_1172690
		S4_23663550
		S5_58692184
		S2_45863094

Catogoery	Total	SNPs
		S3_72154612
		S6_53353798
		S7_8397393
		S6_15331130
		S6_3525949
		S1_14285571
		S5_12715925
		S1_3329765
		S7_57372955
		S2_73740884
		S4_16256981
		S1_14864964
		S3_55805059
		S1_72949964
		S1_11003165
		S7_4526808
		S6_1415785
		S6_41922310
		S1_72691333
		S2_58873435
		S1_49924192
		S9_51890203
		S2_16814409
		S8_56113718
		S5_3613434
		S8_1839095
		S2_8154762
		S1_15907793
		S1_12968216
		S1_14420995
		S1_22055615
		S2_61505405
		S4_13143419
		S1_67567570
		S1_65649103
		S1_12785104
		S8_4481064
		S2_65095903
		S2_67368464
		S7_1639886
		S1_57874569
		S4_6775390
		S5_10555635
		S6_52087285
		S2_61549007
		S3_70856879
		S3_56276013
		S6_20765239
		S7_8397394
		S7_5154297
		S7_53872401
		S5_3187334
		S2_60533933
		S7_5963637
		S8_49087038
		S1_72940286
		S1_49415027
		S6_330085
		S9_2754294
		S2_62139142
		S1_72949921
		S8_32829500
		S2_16848234
		S2_64464938
		S1_67801983

Catogoery	Total	SNPs
		S9_54917701
		S5_6991342
		S3_3622832
		S2_56904254
		S1_6625660
		S4_51277611
		S4_20114058
		S2_67656785
		S4_5107303
		S3_22472367
		S1_2715229
		S2_64368069
		S1_72766670
		S1_71601826
		S3_61393178
		S2_73813174
		S6_58551686
		S2_6009191
		S1_79638265
		S1_14420980
		S4_62936599
		S1_77343693
		S6_44560725
		S3_72849689
		S8_46347251
		S7_61414567
		S7_5171848
		S2_56904274
		S5_9181082
		S6_52485875
		S7_56160938
		S1_18315778
		S5_2571570
		S1_15315301
		S2_27054650
		S6_13915681
		S6_359049
		S1_19184676
		S9_53867529
		S2_56439200
		S8_1991830
		S9_3055421
		S8_504221
		S6_1839684
		S1_57419605
		S3_5626293
		S7_56160908
		S4_62936396
		S1_50787048
		S3_53972530
		S6_14806975
		S6_808986
		S9_56943762
		S3_19589570
		S2_63290513
		S8_45095054
		S1_1902651
		S4_38905545
		S5_10420146
		S1_74323695
		S2_38860433
		S9_57756001
		S2_6218390
		S2_3216766
		S1_72430279

Catogoery	Total	SNPs
		S9_58973756
		S3_72807274
		S9_58727975
		S1_65223559
		S5_61577399
		S1_12758238
		S3_73634524
		S1_79149958
		S1_72567698
		S1_63354974
		S9_57057648
		S2_61708630
		S9_57139943
		S7_9515682
		S1_14512256
		S9_56945635
		S8_1325745
		S2_61233514
		S2_67463962
		S2_55211626
		S4_51774544
		S1_73866784
		S3_72403315
		S6_1332872
		S9_53867526
		S1_31474198
		S9_2239828
		S4_43523383
		S1_72611603
		S9_51830251
		S5_39054517
		S9_57065277
		S1_14448621
		S1_15015362
		S9_5512888
		S2_12686859
		S3_20069954
		S2_41611737
		S2_60724171
		S3_60581793
		S2_60709420
		S5_9053124
		S3_74068840
		S1_59985148
		S7_29646785
		S3_55801307
		S9_53527889
		S7_8105662
		S5_12308132
		S9_165205
		S2_76908526
		S3_59746638
		S1_50830279
		S4_8592446
		S5_9181075
		S1_59558610
		S1_59982178
		S9_153373
		S1_12968198
		S9_43445226
		S3_54878830
		S9_55121776
		S1_12563736
		S3_14383038
		S7_6974581

Catogoery	Total	SNPs
		S6_8919203
		S2_30234091
		S5_67670584
		S1_14448634
		S8_1123093
		S1_56735617
		S3_64840196
		S2_6410085
		S4_46972428
		S1_27278097
		S7_63084000
		S2_13459059
		S9_57670262
		S6_45543005
		S6_51800742
		S9_54370459
		S7_60468035
		S2_73374818
		S1_77343688
		S9_57911519
		S1_77343694
		S4_63170220
		S1_60816164
		S6_1332901
		S1_19184677
		S1_79545879
		S4_56097128
		S9_57792165
		S3_68600838
		S1_7222692
		S1_67607594
		S3_66631231
		S4_52118630
		S6_52682975
		S9_58748317
		S1_68883121
		S2_9529733
		S1_65223574
		S3_56160181
		S6_18323513
		S3_71689566
		S4_62935693
		S7_59830113
		S8_60943170
		S9_57069097
		S6_54400448
		S5_61929439
		S3_56150258
		S4_55552100
		S4_27569230
		S4_40351832
		S1_74323691
		S5_10493199
		S7_57892844
		S1_61353815
		S7_6975329
		S1_68069848
		S7_61149948
		S4_41454633
		S1_6239640
		S7_58845835
		S1_13668564
		S4_46239103
		S1_13469196
		S3_53324852

Catogoery	Total	SNPs
		S2_76430895
		S2_64531149
		S9_57273252
		S7_59307230
		S2_13809628
		S1_79149949
		S1_19349521
		S6_1460005
		S9_52923072
		S1_14285572
		S5_63272413
		S7_410878
		S9_2546489
		S9_58775824
		S1_75378360
		S6_49426749
		S9_58839540
		S3_74099131
		S3_54714984
		S1_79306363
		S2_62726505
		S3_73027184
		S4_8916473
		S3_73231302
		S1_66652233
		S1_16387829
		S9_55492025
		S1_12959333
		S5_14745074
		S5_55298105
		S6_53641290
		S6_1917432
		S4_56427894
		S7_62869363
		S7_57417016
		S5_61921236
		S1_77252113
		S9_59299818
		S2_5786269
		S1_70223610
		S9_53897971
		S9_57541114
		S6_44610491
		S2_53473255
		S5_68185691
		S6_38162288
		S2_55292150
		S2_30234097
		S5_61931981
		S1_8180759
		S1_60519774
		S9_6802694
		S1_15908614
		S3_53551432
		S3_71054443
		S6_57536070
		S3_72311013
		S5_1932849
		S2_71254462
		S1_56906702
		S2_59645184
		S5_12855792
		S2_12905708
		S5_66430112
		S6_15331131

Catogoery	Total	SNPs
		S8_1515469
		S3_68055529
		S6_29476511
		S1_13892096
		S1_15015363
		S1_65324937
		S9_53529462
		S4_9587212
		S3_1945649
		S3_70486044
		S9_54973417
		S9_54908191
		S1_3464577
		S6_45176035
		S6_46467404
		S1_7782167
		S9_59311770
		S1_71606019
		S2_56196270
		S2_67205749
		S4_6463380
		S4_52118633
		S8_60944459
		S1_12758577
		S2_2679997
		S2_12905653
		S3_6754729
		S1_57596308
		S1_61603639
		S3_57369759
		S5_11347038
		S1_16731268
		S6_36255557
		S2_61266192
		S1_78585090
		S3_59746642
		S6_54168529
		S3_59746637
		S3_59503600
		S3_74310717
		S2_57264320
		S4_62935692
		S1_15880752
		S7_8905055
		S4_1645507
		S7_61149953
		S8_50475201
		S1_75624752
		S2_65650443
		S2_56985131
		S4_12722232
		S9_57811268
		S1_76479677
		S3_73215479
		S2_59655524
		S2_74969570
		S9_58748326
		S9_49338003
		S6_52087308
		S4_22476336
		S1_72940285
		S2_61549008
		S7_60698183
		S9_55095671
		S7_5301931

Catogoery	Total	SNPs
		S8_1066073
		S1_77764974
		S1_9629161
		S1_79990582
		S1_14876303
		S5_8604813
		S1_71614848
		S9_42808999
		S6_37547014
		S5_10818778
		S1_55831986
		S1_12156535
		S8_339001
		S1_78485839
		S7_61149960
		S7_1522182
		S2_6245665
		S6_39673152
		S2_14746117
		S2_63803726
		S2_55292149
		S1_12757772
		S6_26383084
		S1_15880766
		S2_61733986
		S6_1306615
		S6_31754242
		#N/A
		S2_49669489
		S3_5146459
		S1_12785120
		S2_61105192
		S5_3620138
		S2_4820725
		S6_3444769
		S6_4092964
		S2_67205751
		S1_3464575
		S8_3510658
		S1_16390928
		S2_16041893
		S7_60260059
		S1_9898152
		S1_12563733
		S9_52923069
		S2_69124081
		S9_57069131
		S9_1203414
		S1_72171208
		S9_56115202
		S5_10493195
		S4_41454638
		S1_30564915
		S6_1783639
		S3_59308318
		S2_6888065
		S9_2239848
		S6_30982372
		S2_15077590
		S8_1066065
		S2_63057180
		S6_1391724
		S2_76430894
		S8_56113728
		S5_6765184

Catogoery	Total	SNPs
		S5_8702688
		S7_4785415
		S6_6657906
		S8_5477029
		S9_55067231
		S9_57657884
		S2_61548991
		S6_18618211
		S3_59693399
		S3_70547272
		S1_14448620
		S7_7619279
		S7_56054547
		S1_16390978
		S1_12165733
		S2_63290517
		S2_65784823
		S1_15907794
		S1_15015351
		S2_68556738
		S9_58973755
		S9_53867527
		S2_69593580
		S3_73149171
		S2_62128772
		S6_55225385
		S6_53506012
		S6_6738840
		S1_72567740
		S1_13102046
		S4_58174391
		S2_40321184
		S2_65359876
		S3_73509002
		S8_2615030
		S2_18307256
		S1_3329760
		S2_6245630
		S5_12536087
		S5_437541
		S8_53626886
		S1_63672014
		S9_55566804
		S8_5477049
		S9_56535046
		S5_1152721
		S1_79967071
		S3_15959211
		S6_54257380
		S1_13300990
		S4_13143467
		S1_59985195
		S9_56943763
		S1_72302139
		S9_53930959
		S2_58500920
		S1_70551787
		S9_47145952
		S9_57833859
		S4_9770382
		S5_7283752
		S6_54257355
		S1_72949908
		S5_8668415
		S8_4289984

Catogoery	Total	SNPs
		S5_9053128
		S2_61091712
		S4_55553811
		S5_4130475
		S2_63357026
		S1_75394958
		S3_56150267
		S1_60523410
		S2_30114663
		S5_47972099
		S2_57042267
		S6_18618207
		S2_67952247
		S2_61549009
		S1_13376221
		S7_59389616
		S6_6657963
		S1_71511878
		S1_63965855
		S7_62431927
		S2_49637033
		S7_60407976
		S1_7222713
		S4_48092800
		S1_78752784
		S6_46380491
		S1_69035433
		S2_49867897
		S4_21752328
		S2_58286606
		S5_2047156
		S6_1520155
		S6_54448658
		S1_68883135
		S8_1558740
		S2_10821418
		S6_18786387
		S1_56567618
		S7_8397385
		S2_62484730
		S1_56033562
		S3_62531574
		S5_11779425
		S2_56904295
		S2_6888069
		S4_62880530
		S7_57892845
		S1_57740678
		S5_4866177
		S1_73644284
		S2_5685669
		S1_71174651
		S2_25873582
		S4_52408267
		S3_73485829
		S6_50726639
		S9_58761955
		S3_71876675
		S3_70547280
		S2_69715021
		S1_77343695
		S3_59503608
		S1_6059478
		S6_17356407
		S1_48835922

Catogoery	Total	SNPs
		S6_1339921
		S2_56515124
		S5_3282948
		S1_12563723
		S5_61929441
		S9_58634599
		S2_7828106
		S9_53172828
		S1_1215475
		S5_10493196
		S9_56943761
		S2_16814404
		S2_8823410
		S2_66035257
		S3_62087338
		S1_3547245
		S1_66786951
		S8_51922096
		S1_12945018
		S3_4610275
		S4_67288874
		S1_79582017
		S9_519136
		S3_51536454
		S8_5379958
		S1_13832006
		S8_53860662
		S4_56570801
		S9_236344
		S5_66487485
		S6_47700046
		S1_78753233
		S9_58127631
		S2_14789653
		S9_54583679
		S3_52182575
		S9_58531766
		S7_54789016
		S7_60263995
		S1_78748478
		S9_54093550
		S2_73374792
		S7_151554
		S6_1454288
		S4_6775390
		S1_14864972
		S6_330054
		S1_14448625
		S1_13095746
		S6_822527
		S9_41430950
		S4_50669861
		S1_7222659
		S3_5146440
		S1_78036426
		S9_56542015
		S3_69552210
		S7_11042618
		S7_56425997
		S1_68697259
		S2_63290528
		S3_72125617
		S9_58748316
		S8_2615029
		S4_62935747

Catogoery	Total	SNPs
		S5_15208630
		S4_25714460
		S2_60533933
		S5_9053127
		S4_51106775
		S2_18307270
		S3_3622825
		S8_61612185
		S3_69688959
		S7_8994958
		S8_59748618
		S1_1897884
		S6_46380487
		S1_56327744
		S6_51148591
		S7_1639911
		S7_65242089
		S2_66412848
		S1_75394912
		S1_12563731
		S2_61733993
		S9_57875319
		S1_72949918
		S3_3776153
		S9_56943774
		S5_4499186
		S2_13459070
		S8_46320095
		S7_538372
		S3_70688041
		S1_9705879
J2614/RSG04008	27150	S9_52612564
		S1_71814479
		S6_41102175
		S8_5259909
		S5_1524851
		S3_52014248
		S9_4207693
		S6_50309420
		S6_13377664
		S1_2909539
		S6_1391768
		S2_61695393
		S4_2306073
		S5_11905628
		S4_788809
		S1_62745901
		S5_1836514
		S2_66837757
		S2_329580
		S2_71569857
		S9_57611406
		S2_3998316
		S4_7390351
		S5_517172
		S8_55282932
		S5_62729511
		S8_56112473
		S9_5862636
		S9_1749889
		S4_50527457
		S9_1188105
		S7_62499692
		S8_42795456
		S1_61604325

Catogoery	Total	SNPs
		S4_9633212
		S8_61769098
		S1_59434480
		S7_12351526
		S4_53748186
		S8_4414023
		S3_63314092
		S1_2657546
		S9_50995225
		S5_8225542
		S2_69327372
		S3_64354597
		S4_33590924
		S2_69849611
		S8_495760
		S6_77554
		S3_600682
		S3_73807474
		S2_5488874
		S1_55917763
		S4_56522569
		S6_47409004
		S8_44960151
		S4_14127682
		S4_3189434
		S8_4542267
		S4_3253589
		S2_3428365
		S2_487459
		S6_58759537
		S9_43444599
		S9_51117411
		S5_18513771
		S4_6185234
		S1_66571295
		S6_52742440
		S1_9062513
		#N/A
		S1_9504085
		S4_22774202
		S2_59718245
		S4_15902715
		S6_51803454
		S5_67362938
		S6_51320769
		S3_71412130
		S5_69718748
		S1_7451910
		S7_61598299
		S2_64750105
		S7_19083362
		S2_64827233
		S7_54506420
		S4_44730239
		S4_8833721
		S7_8018533
		S2_1475398
		S4_41484116
		S9_57678990
		S3_69704218
		S8_55190095
		S1_60005630
		S9_52823603
		S7_52306329
		S4_44730219

Catogoery	Total	SNPs
		S7_10021005
		S6_45400862
		S1_20249805
		S2_16667420
		S4_51082366
		S9_9960353
		S6_335681
		S7_7749180
		S7_2920294
		S8_59749197
		S5_50603263
		S3_58247021
		S2_67807398
		S9_57629295
		S5_9537226
		S4_5850165
		S9_52880046
		S1_66650944
		S8_5160875
		S1_27367102
		S2_75613715
		S8_49673676
		S2_61310432
		S7_15698330
		S5_67765629
		S4_12409946
		S2_10286032
		S8_61171146
		S4_1577191
		S3_51971390
		S2_61444886
		S2_62966166
		S1_22034788
		S2_14516334
		S5_66598168
		S6_53396938
		S5_2829052
		S8_58125298
		S3_4955926
		S7_52649497
		S2_66339831
		S4_48091206
		S1_54540069
		S8_56332090
		S7_59056669
		S3_5521101
		S7_60709291
		S2_6887652
		S2_71556781
		S2_63077910
		S8_53262187
		S2_61444894
		S2_67991002
		S2_59247261
		S6_60460609
		S6_44813963
		S8_49933711
		S6_45540264
		S2_69194608
		S2_11737333
		S5_5114439
		S5_36721531
		S4_38398506
		S4_7411067
		S3_16373959

Catogoery	Total	SNPs
		S2_58140422
		S7_40119244
		S5_68983172
		S3_16410910
		S3_16131831
		S3_58246397
		S2_12434690
		S2_55206357
		S2_76838228
		S1_24465875
		S9_51116196
		S4_51052355
		S8_46039601
		S4_2233582
		S6_44731833
		S1_14438478
		S6_54279906
		S5_15887140
		S2_61432135
		S8_60912392
		S6_46327584
		S5_58574452
		S3_55300418
		S2_8038717
		S4_52778246
		S1_24790341
		S2_76706690
		S2_49652766
		S6_55634037
		S1_66348185
		S2_75343602
		S7_52242708
		S1_9369797
		S4_43740319
		S5_6750570
		S7_19042402
		S1_10686110
		S3_14076810
		S8_61165329
		S9_57563232
		S1_59759837
		S5_70791594
		S6_53215586
		S4_17381066
		S2_12735203
		S3_4682320
		S1_18956176
		S8_1840386
		S3_52014117
		S7_58778466
		S5_15890680
		S1_79858404
		S1_78126251
		S8_58151618
		S6_53857077
		S1_7092711
		S4_49329084
		S1_10678099
		S9_8512781
		S4_10542285
		S3_14076818
		S5_15194110
		S8_58680013
		S4_50643835
		S6_42083924

Catogoery	Total	SNPs
		S2_13177260
		S2_68602616
		S3_713252
		S2_12452484
		S6_37637731
		S6_3980119
		S7_9690908
		S6_72128
		S8_17917601
		S3_73817556
		S9_8337208
		S3_54507831
		S7_6761329
		S9_1658622
		S9_3194729
		S3_2480017
		S3_73334246
		S8_60932759
		S2_59538584
		S7_17873660
		S2_2396226
		S8_55721684
		S5_46337817
		S2_64342440
		S7_62237249
		S1_18055105
		S4_5867035
		S5_2693011
		S2_14677868
		S1_53476309
		S4_55660090
		S6_45197529
		S6_1839010
		S2_69135586
		S1_52755859
		S6_53933673
		S1_56731747
		S4_52535323
		S3_54181138
		S6_5905914
		S3_47930515
		S4_53344053
		S2_73277338
		S5_2966605
		S9_2931140
		S9_51454659
		S1_52089011
		S9_7788417
		S5_23824055
		S6_46386754
		S2_10329066
		S4_34312537
		S1_7624636
		S5_11005138
		S5_15774685
		S2_14360140
		S9_8398529
		S1_12101199
		S8_3219403
		S4_34492363
		S9_51655220
		S2_6691795
		S1_53615827
		S5_69823127
		S2_66633686

Catogoery	Total	SNPs
		S2_54248442
		S2_63376906
		S3_51997027
		S1_17585363
		S8_37714719
		S2_69860066
		S2_3641569
		S2_64413829
		S8_53638830
		S4_5085179
		S1_68783476
		S1_2919335
		S1_12137838
		S5_10195776
		S4_16244958
		S2_66842234
		S1_12305241
		S8_4413919
		S2_16424372
		S2_64464764
		S1_55709375
		S4_6615702
		S1_51889030
		S3_69250267
		S5_11465085
		S2_69223415
		S2_16341022
		S5_18446050
		S6_47043887
		S4_11132930
		S4_26389669
		S2_19318607
		S7_61625503
		S6_41539439
		S4_55549159
		S6_49220117
		S2_75510167
		S2_62315582
		S5_6084910
		S8_5442004
		S1_72416137
		S3_52457548
		S1_9062423
		S1_4638860
		S6_48638205
		S4_49016773
		S1_80252636
		S9_2884151
		S2_6755754
		S1_65680763
		S2_11851278
		S4_2005822
		S4_13959489
		S4_44730221
		S5_68864724
		S1_7212592
		S1_74979878
		S8_50567777
		S4_53742052
		S2_57495669
		S1_2744757
		S1_63672151
		S1_15031545
		S2_76838356
		S2_72264295

Catogoery	Total	SNPs
		S9_42681648
		S2_47634491
		S3_60922446
		S7_8528392
		S6_49470591
		S1_75624803
		S1_9667298
		S3_60581860
		S8_44848384
		S5_3372033
		S2_57862875
		S2_56515277
		S1_10739041
		S2_74680825
		S6_1067412
		S7_12287712
		S8_59174542
		S1_5730775
		S3_66906456
		S5_6809730
		S3_4576521
		S4_66157084
		S7_6984845
		S9_58752297
		S4_23730689
		S5_12297388
		S3_58580606
		S8_57452613
		S2_61060347
		S3_799115
		S3_1869794
		S6_935156
		S4_38228965
		S3_55345675
		S2_75650869
		S8_61980225
		S3_2580918
		S4_39813395
		S8_53520103
		S3_68055682
		S5_66081361
		S2_10832263
		S6_50333927
		S8_3606797
		S1_68188577
		S1_66340330
		S2_9905518
		S1_10589646
		S5_11513643
		S6_41925297
		S2_74680820
		S1_79932547
		S1_17137926
		S4_9322342
		S3_465841
		S1_28670318
		S3_69195566
		S3_70121136
		S9_53299990
		S2_37442780
		S5_63500294
		S2_49470478
		S1_30750165
		S1_17426842
		S9_8182958

Catogoery	Total	SNPs
		S8_56411726
		S4_21046599
		S1_20207354
		S7_58157543
		S2_63081247
		S2_60441057
		S1_71174949
		S7_6984855
		S6_41274358
		S1_72409292
		S8_55589108
		S1_48535542
		S1_5707278
		S2_75653698
		S2_11037766
		S1_78288271
		S6_38608642
		S2_66925449
		S2_64458204
		S1_73871190
		S9_50020141
		S9_8256362
		S1_78762572
		S5_62499699
		S3_71936637
		S2_57252910
		S8_3846913
		S2_75672240
		S2_6045251
		S6_29670065
		S1_59984929
		S1_61609485
		S1_75620993
		S1_59562688
		S6_21805405
		S6_1994392
		S2_59600095
		S5_1835239
		S2_9638356
		S4_7889627
		S1_73462336
		S9_7311396
		S7_61479107
		S5_62646215
		S4_46623605
		S2_57728577
		S3_6541337
		S2_12858137
		S7_63155672
		S1_57110297
		S6_58805717
		S1_69087707
		S8_3173717
		S5_55344513
		S1_77544712
		S6_54247881
		S3_59597924
		S3_54263400
		S2_9725896
		S1_6644016
		S2_62493962
		S9_4383018
		S7_58333574
		S7_7198082
		S1_59457304

Catogoery	Total	SNPs
		S2_12434771
		S4_52350713
		S6_3980187
		S3_71751752
		S6_1588259
		S2_2758319
		S6_48594693
		S2_1030649
		S8_55930267
		S2_62393740
		S6_6447888
		S2_4566176
		S1_24469018
		S8_2116800
		S2_73738393
		S4_51774112
		S3_69744220
		S4_52473433
		S4_52765007
		S3_69490652
		S2_57433434
		S3_5751866
		S2_69601975
		S1_79510068
		S2_1613916
		S7_15591244
		S5_63590574
		S1_73188117
		S5_69847924
		S5_63990767
		S3_61813754
		S1_65180996
		S6_53299040
		S6_50934144
		S2_61742412
		S2_71701733
		S3_57039969
		S7_1790943
		S1_11192714
		S1_26240427
		S3_51997079
		S7_6412746
		S7_8646849
		S2_8890435
		S5_61208221
		S9_6119539
		S4_2090514
		S7_63639796
		S6_51851666
		S2_76768539
		S1_74961864
		S1_19554732
		S3_3518038
		S3_61017881
		S8_59750932
		S4_38552005
		S3_56758956
		S1_51135553
		S4_450143
		S1_77750964
		S1_60414047
		S8_17974199
		S2_76513428
		S1_12662702
		S6_60453530

Catogoery	Total	SNPs
		S4_66239239
		S2_58202076
		S3_5603393
		S7_64968862
		S2_46748187
		S1_24466077
		S4_51084816
		S4_46042283
		S3_51970957
		S6_29807837
		S2_75791835
		S8_36638831
		S1_55512406
		S3_56254513
		S7_42599198
		S6_2548322
		S2_8647671
		S2_40566644
		S5_65889146
		S5_11055591
		S5_1481040
		S1_11205350
		S7_2834420
		S6_45614567
		S9_51286222
		S1_78236598
		S1_18521549
		#N/A
		S1_59362300
		S1_55049915
		S1_14462459
		S1_62222672
		S1_25819260
		S8_46967479
		S2_5869926
		S9_41818150
		S7_53163254
		S3_71247757
		S7_65103442
		S9_53467000
		S2_62235600
		S7_63021148
		S5_1126502
		S4_51368436
		S2_12686988
		S1_75521120
		S8_2082247
		S9_4454947
		S6_60763283
		S3_2112358
		S4_45652551
		S1_11169754
		S8_58488099
		S7_60301310
		S4_8457465
		S1_59937211
		S4_5085061
		S7_58156316
		S2_66175937
		S7_537126
		S8_57781629
		S6_58812598
		S2_14172714
		S1_65250264
		S8_58455794

Catogoery	Total	SNPs
		S1_79576171
		S1_26313020
		S1_24784752
		S2_61561843
		S1_21254066
		S8_57589263
		S5_3055439
		S3_56069835
		S7_52328207
		S9_50239760
		S2_65235047
		S1_2584196
		S8_60170369
		S6_47901244
		S5_490632
		S2_10327293
		S1_7902159
		S6_6691595
		S5_5335551
		S1_60758038
		S9_54901645
		S5_10489203
		S7_54525587
		S5_16122859
		S6_3561121
		S9_41678324
		S2_17760932
		S1_49433064
		S4_67084402
		S4_2578131
		S5_69851727
		S2_18516155
		S9_59191354
		S1_68788043
		S8_9589213
		S4_7276899
		S4_8882520
		S7_63118121
		S1_14437149
		S3_69744184
		S2_10926671
		S9_58179025
		S2_56955040
		S2_3759416
		S1_52226913
		S4_2148008
		S1_57110284
		S2_57042201
		S2_19732958
		S8_5320759
		S5_66114368
		S9_7313853
		S4_4816316
		S2_58131660
		S5_1910462
		S3_4484813
		S4_3920180
		S6_38194567
		S6_47065230
		S4_41226306
		S5_472214
		S1_17276947
		S9_3218277
		S5_1326712
		S1_18854820

Catogoery	Total	SNPs
		S6_55277732
		S2_56105309
		S1_13102068
		S6_15877541
		S2_457202
		S1_29947528
		S5_10795284
		S2_4566149
		S1_17426836
		S1_6329283
		S2_10527927
		S8_1199536
		S1_13668566
		S3_1969740
		S2_61654773
		S1_63359107
		S1_21473677
		S4_66125824
		S2_3388907
		S2_46693754
		S7_63707683
		S2_63351279
		S1_51841679
		S5_62493114
		S1_63302172
		S1_10793333
		S9_49909556
		S4_66144690
		S4_45652539
		S8_60160194
		S6_51222059
		S4_48385710
		S2_3786789
		S2_12894611
		S1_79208796
		S5_58574518
		S5_67495267
		S3_57040978
		S6_48693490
		S1_9066123
		S9_4454893
		S4_7131966
		S4_2626990
		S2_30090149
		S2_12744921
		S6_52691101
		S1_79659904
		S1_77412252
		S1_7557043
		S2_12754169
		S2_5389769
		S9_50042633
		S2_12754154
		S3_5683108
		S1_12247789
		S8_58029218
		S2_61356435
		S9_5167781
		S3_20073221
		S6_48554200
		S2_1956620
		S1_3461892
		S8_55274497
		S1_55000256
		S5_490395

Catogoery	Total	SNPs
		S1_71640621
		S1_72009817
		S2_42429621
		S6_41350157
		S5_24244999
		S1_14862315
		S5_66098794
		S1_66821747
		S6_54583412
		S3_60935423
		S4_14830235
		S3_53524013
		S8_60214774
		S7_64061186
		S7_62029567
		S7_3930630
		S8_61541258
		S6_49691732
		S9_8996917
		S2_67840036
		S4_53281361
		S1_48989290
		S3_54705285
		S2_64350285
		S4_3789095
		S1_3564600
		S7_63079825
		S9_53689113
		S8_61980171
		S2_5665284
		S1_60005774
		S9_55626868
		S8_55707601
		S2_61587755
		S3_3757316
		S4_5336564
		S1_50829902
		S5_1796449
		S2_68072549
		S7_58747355
		S2_73607906
		S2_60965594
		S7_64067747
		S2_3432082
		S2_12466168
		S9_51890390
		S8_57781635
		S1_64159003
		S9_51705473
		S1_59566980
		S1_60849660
		S4_22272820
		S4_11424261
		S6_61068744
		S5_3388732
		S8_49625326
		S2_68095342
		S1_2584246
		S2_72265476
		S2_9463957
		S8_46715440
		S1_52226871
		S6_46064034
		S7_54775610
		S7_9528476

Catogoery	Total	SNPs
		S2_75672243
		S2_66290375
		S8_55494916
		S6_55698548
		S4_8110124
		S3_15689049
		S4_56526599
		S1_59791348
		S3_60922651
		S7_54776854
		S2_67753833
		S2_59878119
		S7_58297971
		S4_53748503
		S7_38852532
		S3_67154311
		S9_51033828
		S9_55646106
		S4_42907300
		S5_67017677
		S1_3363017
		S3_73925023
		S1_6532604
		S4_1309486
		S8_54820046
		S2_6888170
		S4_54084129
		S8_61590734
		S6_46381712
		S7_63118127
		S6_54303389
		S5_63235220
		S1_72975349
		S1_80075044
		S7_6452710
		S2_62313321
		S1_30175594
		S6_47614045
		S8_60246924
		S8_59749168
		S8_51139067
		S1_76432135
		S6_53396988
		S5_4355028
		S9_1064325
		S3_44538963
		S8_53579073
		S7_62068065
		S8_56144771
		S6_50587412
		S1_14162101
		S9_40884348
		S5_61243552
		S7_52328194
		S1_22102970
		S4_58313414
		S9_50196181
		S9_51033814
		S1_64704490
		S1_912029
		S2_41611779
		S4_5107566
		S2_42945131
		S2_62272873
		S8_1325569

Catogoery	Total	SNPs
		S3_66560822
		S6_921588
		S7_39247477
		S6_45545076
		S2_468917
		S2_5392244
		S2_10832296
		S1_18883994
		S1_77768873
		S5_2224513
		S7_1570472
		S3_57257556
		S1_79932605
		S1_12968342
		S2_66695466
		S7_63739631
		S1_74539187
		S6_51309649
		S4_7081461
		S3_73817553
		S1_66564553
		S1_19154314
		S4_7868625
		S1_58913440
		S8_61915181
		S2_7315663
		S1_71831810
		S7_8583688
		S2_61872515
		S4_42747288
		S7_58295561
		S2_12688136
		S7_10441502
		S2_51573091
		S1_58913452
		S9_58472449
		S4_1004374
		S8_61606853
		S5_18317977
		S1_2084534
		S1_72000492
		S1_67679857
		S2_854046
		S6_53558261
		S9_5438986
		S6_1375099
		S4_58270197
		S4_12075103
		S2_10269494
		S2_6277391
		S8_32720973
		S9_6695468
		S2_59738787
		S4_1437217
		S5_68989204
		S5_61305037
		S6_3327751
		S3_58281909
		S3_71015182
		S3_58732628
		S3_55451457
		S3_2227441
		S6_58759806
		S4_5065232
		S2_62051204

Catogoery	Total	SNPs
		S6_17651821
		S6_31398690
		S3_14045973
		S9_50475546
		S9_4067498
		S3_73487935
		S4_43141278
		S7_2796118
		S2_6045419
		S9_2251131
		S8_57485708
		S1_25633667
		S9_4743787
		S5_66226189
		S8_57541076
		S3_55386145
		S9_6344453
		S6_47901263
		S2_13956977
		S1_11107581
		S2_9417007
		S8_42894485
		S9_2658135
		S2_58682757
		S6_41457652
		S1_77768851
		S5_65524477
		S3_4872831
		S1_73467704
		S5_3621110
		S8_5009282
		S2_61923292
		S6_42355180
		S2_62051316
		S9_51890217
		S2_38861452
		S6_52515226
		S2_56877690
		S3_6755033
		S2_63261883
		S9_3913698
		S6_38175752
		S2_3711510
		S7_7611624
		S2_3427377
		S1_56567573
		S3_57237986
		S5_36544434
		S9_3100272
		S7_64000193
		S8_36784011
		S5_13332729
		S1_67311257
		S3_56161768
		S1_57944436
		S1_31011800
		S1_60912496
		S1_15031644
		S3_57925445
		S6_1747383
		S5_1625520
		S3_70844781
		S2_33743303
		S4_12408508
		S4_4413496

Catogoery	Total	SNPs
		S1_59824406
		S6_50706677
		S3_70242201
		S1_18905237
		S2_12467143
		S9_54080827
		S2_12758476
		S8_29266718
		S6_51865309
		S2_59737433
		S3_51259390
		S9_8945265
		S5_61864603
		S8_3186440
		S7_7936960
		S1_9038904
		S1_11127570
		S5_65523892
		S1_58636543
		S2_75958006
		S9_11675267
		S1_25897341
		S3_69268861
		S6_44775845
		S2_73494286
		S1_73735399
		S2_14724290
		S2_8867775
		S1_2297968
		S4_1301333
		S1_51525836
		S1_10949872
		S2_6045010
		S1_79165724
		S2_2263970
		S3_60530840
		S7_51533017
		S1_77343712
		S5_2693192
		S3_54694797
		S4_67204598
		S9_7788411
		S1_71640619
		S4_3721519
		S3_51322647
		S1_64546849
		S9_55998035
		S6_47178984
		S7_65382454
		S7_60488045
		S7_64417760
		S6_53727761
		S4_5330422
		S4_836919
		S1_80475677
		S6_38196835
		S1_80476164
		S9_51523641
		S1_72350729
		S3_54933775
		S2_6543574
		S3_4956341
		S2_9725902
		S3_60619635
		S3_62858486

Catogoery	Total	SNPs
		S2_66061612
		S5_1122110
		S4_24003670
		S6_52206353
		S1_10687516
		S7_62782538
		S8_3173808
		S2_56431573
		S7_5994874
		S8_53709187
		S4_19681219
		S1_12968129
		S9_57907058
		S5_62493195
		S5_5248819
		S5_3397178
		S4_7889603
		S7_14233720
		S1_9809065
		S1_64631738
		S4_58263196
		S2_60110605
		S9_8337208
		S2_262334
		S4_56422174
		S6_1586988
		S5_20234318
		S6_53150749
		S8_55153945
		S1_71754817
		S1_12305548
		S6_50394948
		S1_72593157
		S2_69829407
		S5_62355366
		S4_3814343
		S4_66158423
		S4_12331531
		S2_69148754
		S8_1839524
		S2_64866050
		S2_11055052
		S2_71244528
		S8_49264050
		S1_64391924
		S6_20485631
		S1_67332437
		S1_8782238
		S4_12147457
		S2_67807386
		S2_14486711
		S9_56945587
		S2_63472076
		S6_48327337
		S6_51096991
		S6_46349024
		S1_79775168
		S2_74867331
		S9_5167776
		S9_57575478
		S5_2245618
		S4_63000066
		S1_49780391
		S1_7367560
		S4_6938941

Catogoery	Total	SNPs
		S9_9190144
		S8_60924460
		S3_68014882
		S6_29669989
		S5_62719831
		S3_64455899
		S1_12945068
		S1_19158082
		S8_57441797
		S5_51325475
		S5_66485236
		S6_2578812
		S2_57448611
		S1_76495672
		S8_53982165
		S3_72789839
		S3_15625148
		S4_25789912
		S1_66564573
		S9_57657923
		S2_74073533
		S9_57657887
		S1_11686452
		S1_11702246
		S9_4790851
		S3_68841606
		S1_50828975
		S6_51080262
		S8_51033660
		S2_8892383
		S7_41904886
		S2_52329354
		S2_13133133
		S5_1931788
		S2_75838546
		S2_12351392
		S5_12297369
		S9_5829128
		S6_26232726
		S4_23549959
		S8_40488310
		S1_10407865
		S6_53638836
		S6_77584
		S1_18967251
		S8_44339997
		S4_38900395
		S2_56881342
		S6_51427971
		S2_68072545
		S2_62729849
		S6_48693491
		S8_4491977
		S5_56030650
		S6_53723545
		S9_1675486
		S8_60912401
		S1_66894544
		S2_72606469
		S6_30920325
		S4_700155
		S7_59393452
		S1_24771007
		S1_27403064
		S2_65345380

Catogoery	Total	SNPs
		S7_555189
		S1_75899411
		S9_41257229
		S2_73816089
		S6_49718549
		S3_69698530
		S2_66465051
		S8_61863438
		S8_60695648
		S4_5902113
		S2_63179741
		S2_8239214
		S5_11504258
		S1_71946561
		S9_4790705
		S8_56501380
		S6_44828820
		S1_22051845
		S8_48068446
		S1_18043455
		S1_58180207
		S4_7340749
		S1_71831799
		S6_48693470
		S1_71605987
		S9_4320288
		S3_56905805
		S1_51003640
		S2_76913337
		S1_75624659
		S8_S8_42372028
		S5_61748994
		S1_21473676
		S3_66743965
		S1_66564545
		S4_22166161
		S1_71592612
		S8_56765193
		S1_20207365
		S7_52170072
		S4_16413895
		S2_60965628
		S7_2927180
		S9_50867947
		S3_16222579
		S1_58516070
		S3_74068824
		S3_5136728
		S8_54855514
		S6_3534933
		S4_67087270
		S4_2004729
		S2_56986888
		S8_47521889
		S2_67339033
		S7_16431442
		S3_60936302
		S2_11765687
		S6_49476630
		S8_61603512
		S8_56501119
		S5_15911074
		S2_8382615
		S1_67926981
		S3_59723387

Catogoery	Total	SNPs
		S2_65679809
		S4_34740025
		S3_61148779
		S2_57083748
		S9_2871432
		S2_53110266
		S9_52551749
		S7_62611600
		S2_75217695
		S6_52777832
		S4_7889588
		S3_74143089
		S2_65965886
		S2_6652571
		S2_75893552
		S8_3219288
		S7_12916271
		S6_17792710
		S2_76812915
		S9_52824396
		S7_64061482
		S7_1163211
		S3_73878126
		S1_60670354
		S6_41925155
		S7_567490
		S7_60299058
		S1_50852318
		S2_61411755
		S4_5866922
		S2_46699698
		S2_53473305
		S1_2294719
		S2_10821484
		S1_1346279
		S8_4192200
		S1_60758140
		S5_62418994
		S5_62849841
		S8_11854041
		S4_66217385
		S7_62935592
		S9_57793482
		S2_11765724
		S7_62396711
		S6_493657
		S9_58114234
		S2_62393737
		S3_69587306
		S5_1812770
		#N/A
		S6_32048804
		S1_59477610
		S5_5045949
		S1_74198332
		S2_73138515
		S2_69189883
		S9_51647522
		S3_71421932
		S4_41169837
		S1_10408832
		S1_72242902
		S3_70135322
		S5_4803814
		S3_57369669

Catogoery	Total	SNPs
		S3_57460670
		S1_1559074
		S4_66125730
		S1_33167285
		S9_2935907
		S1_24623295
		S2_53068877
		S4_40429335
		S1_49427760
		S2_72264302
		S4_1510627
		S4_5085171
		S7_6949096
		S3_16063296
		S1_54540477
		S2_59246457
		S1_12968116
		S1_21035056
		S3_62858481
		S9_52777236
		S6_48213989
		S3_59282296
		S2_54248288
		S3_1935863
		S2_49863514
		S2_6423700
		S7_55982549
		S2_75991665
		S2_60110406
		S2_77028035
		S3_2576524
		S9_50017444
		S2_75739798
		S1_77765800
		S9_49962575
		S6_60728393
		S6_37400390
		S3_53300153
		S2_61859443
		S5_66045595
		S5_69710077
		S3_6109972
		S2_72615397
		S1_66339261
		S2_65096335
		S1_130558
		S4_2504876
		S5_1326983
		S1_77100622
		S9_52241401
		S1_7621109
		S8_18292361
		S1_79965945
		S4_12792063
		S2_18466501
		S1_30327412
		S1_15673370
		S4_66103990
		S1_52349847
		S6_34498437
		S2_73693967
		S2_8702836
		S1_5871126
		S7_887851
		S3_71236498

Catogoery	Total	SNPs
		S2_18574112
		S1_2899266
		S3_60582751
		S3_2360945
		S1_8644856
		S7_62494226
		S8_43504344
		S9_5332572
		S9_42208700
		S8_47531142
		S7_1163389
		S3_13769800
		S5_70773459
		S7_60709194
		S7_9563181
		S8_3457010
		S7_63156251
		S1_49964354
		S9_59159058
		S7_40119174
		S2_6045450
		S2_67338935
		S4_12384879
		S2_67282448
		S1_77764819
		S5_14747128
		S1_53469902
		S8_60835782
		S4_11072650
		S3_5106782
		S1_2313810
		S1_67332419
		S2_37938410
		S5_62950663
		S4_62878220
		S3_72071698
		S5_67495284
		S6_40987304
		S1_79194045
		S7_54216799
		S1_71822903
		S3_66606728
		S2_61695667
		S2_60441070
		S1_60677743
		S1_17468705
		S7_38852586
		S3_73700080
		S5_5249161
		S1_66528020
		S8_57771903
		S2_6396285
		S5_6065163
		S1_55284221
		S7_6507355
		S1_988
		S1_21720872
		S3_1983177
		S1_79424031
		S4_61260556
		S5_58548617
		S4_51278090
		S3_48915720
		S7_433006
		S1_6775722

Catogoery	Total	SNPs
		S4_48612765
		S3_1969733
		S2_70913202
		S1_66340733
		S1_72186040
		S4_1800570
		S1_72010106
		S1_65740881
		S9_51666246
		S7_62194026
		S3_68034010
		S7_62029527
		S8_11365063
		S5_651696
		S8_36888150
		S3_72441614
		S1_21226202
		S2_64144913
		S8_57571898
		S2_10603680
		S8_45793317
		S3_57466949
		S2_18573479
		S9_2825017
		S4_2893839
		S1_23907616
		S8_1753896
		S1_19376032
		S5_4890878
		S7_4559702
		S2_68574800
		S3_72625577
		S1_16670026
		S9_2615839
		S8_3415871
		S2_65028829
		S1_8649083
		S9_49992568
		S8_61842917
		S6_47045060
		S7_437396
		S2_16390694
		S9_55626945
		S9_9538460
		S9_6367058
		S9_51890415
		S5_3621087
		S9_55969250
		S3_1964491
		S1_71831815
		S4_7889912
		S4_1755093
		S8_54853057
		S3_63301483
		S5_1189497
		S1_14438397
		S7_62920632
		S8_57752797
		S3_3915208
		S1_19376018
		S1_58661357
		S1_10098628
		S9_51752556
		S8_4409268
		S4_57520671

Catogoery	Total	SNPs
		S7_41523770
		S7_54375899
		S2_60087691
		S6_16994085
		S3_73114605
		S5_62919957
		S1_14080861
		S5_69794425
		S2_69174874
		S1_2860454
		S3_71422065
		S4_19935243
		S9_3616994
		S6_47065237
		S3_2454949
		S8_55622493
		S3_51516359
		S2_59335671
		S1_27756838
		S5_2916960
		S9_58880242
		S3_38419862
		S3_70691356
		S1_12758272
		S6_31450146
		S1_1341704
		S5_10354468
		S1_14081067
		S9_59171496
		S1_75862720
		S4_137748
		S2_10976644
		S1_73879478
		S1_11554764
		S3_72514877
		S4_49133915
		S7_9515486
		S2_57258388
		S4_42486186
		S2_18761329
		S8_57752805
		S6_1316196
		S7_62476208
		S1_59699510
		S5_67567208
		S3_53520797
		S4_3797216
		S2_7055324
		S4_51515678
		S1_71525155
		S1_5805316
		S1_2923872
		S2_67732479
		S1_66986837
		S7_62832449
		S1_53572273
		S4_2628089
		S2_67873314
		S5_11706289
		S6_6466682
		S1_78032452
		S4_5107114
		S2_4566187
		S2_66706723
		S4_67316847

Catogoery	Total	SNPs
		S9_52331492
		S4_48943039
		S3_73218642
		S5_61166848
		S2_56290181
		S2_8149412
		S5_61921336
		S1_1912074
		S3_70346685
		S6_52136569
		S6_45640376
		S6_1734882
		S6_52635480
		S3_15437478
		S3_71247713
		S3_69810208
		S6_54390544
		S3_73925027
		S2_62483839
		S2_60855893
		S4_58178155
		S6_53638849
		S3_70711108
		S3_73887971
		S5_9502833
		S2_41395929
		S3_3776188
		S2_64342422
		S1_23566478
		S4_67087247
		S4_1193625
		S3_58196913
		S2_56332752
		S5_61610875
		S4_12105385
		S6_55689464
		S6_54504040
		S7_55982570
		S2_12466191
		S6_19445130
		S1_21951666
		S9_57563236
		S4_38297615
		S1_67303128
		S7_60297748
		S3_72668794
		S6_48595391
		S4_38126673
		S1_7953708
		S9_6683833
		S9_43747397
		S7_5171833
		S5_62875094
		S2_75991679
		S1_2030898
		S4_4197441
		S2_57591450
		S3_69744216
		S1_59976431
		S5_6904641
		S5_5988413
		S1_8818954
		S9_10597839
		S2_68059576
		S1_2955422

Catogoery	Total	SNPs
		S6_6109025
		S1_5878256
		S2_9484847
		S7_62716935
		S1_65256954
		S2_42945122
		S2_5389763
		S4_56314543
		S1_75662841
		S3_52245056
		S5_32136
		S4_1338830
		S9_58114230
		S3_72096063
		S2_68830015
		S8_47526118
		S1_8881277
		S9_514702
		S8_5259980
		S3_61246825
		S3_6748205
		S1_19425979
		S7_56022301
		S1_17864144
		S1_68754225
		S6_954348
		S9_4365940
		S2_23357822
		S3_4576628
		S1_73661591
		S1_56731829
		S9_58978063
		S6_912408
		S2_6036388
		S1_66983009
		S4_8673901
		S3_61719157
		S9_4419145
		S7_62813788
		S3_73046030
		S4_11460164
		S2_49667998
		S8_42471750
		S8_38228173
		S2_74783755
		S9_55849084
		S2_60810822
		S3_5130755
		S2_10846804
		S1_59941546
		S2_63357242
		S9_6428271
		S7_59956021
		S3_16057358
		S8_55614040
		S4_67087249
		S2_4267508
		S2_47145710
		S2_41116496
		S1_4144200
		S2_66280871
		S6_40264949
		S5_67633878
		S1_11197487
		S8_5320766

Catogoery	Total	SNPs
		S1_77346176
		S7_59404164
		S1_30239719
		S1_67073525
		S2_12363128
		S2_49433573
		S1_20255559
		S9_53748581
		S3_57340299
		S8_1840292
		S1_52349791
		S9_52077318
		S6_51171410
		S8_1612588
		S3_53551026
		S2_60289481
		S2_2323690
		S4_4535068
		S5_3255894
		S2_12358025
		S4_68114596
		S3_52457551
		S2_6100081
		S9_52908068
		S1_67688552
		S2_62052872
		S6_54236916
		S4_6008982
		S9_43564911
		S4_53512178
		S9_4249056
		S1_77110549
		S1_55830312
		S6_53187988
		S7_63701847
		S2_6203814
		S4_54127111
		S1_11686458
		S2_10054541
		S4_7201211
		S4_62878055
		S6_44862128
		S6_47822490
		S2_47292136
		S2_69834682
		S9_10737467
		S2_59642470
		S6_50364500
		S8_61165321
		S8_59250896
		S6_49221114
		S8_55707601
		S4_2762255
		S3_51172054
		S2_61047598
		S9_42398429
		S2_7703708
		S5_2518712
		S1_78327768
		S7_54707394
		S5_69050212
		S4_62936594
		S5_1030304
		S1_61768194
		S1_11453220

Catogoery	Total	SNPs
		S8_54754698
		S1_14512219
		S3_72307568
		S4_12910713
		S1_66650937
		S1_46549111
		S3_73575773
		S3_57040382
		S4_24429450
		S5_59372539
		S2_61927304
		S1_65736327
		S6_2016270
		S9_57093230
		S7_766143
		S5_10814629
		S2_77008766
		S1_4277391
		S1_57165915
		S4_66334391
		S4_4074449
		S5_15774692
		S3_41087925
		S2_77505413
		S2_52306758
		S2_68533930
		S3_69391852
		S4_54046986
		S1_65259535
		S6_50876031
		S2_61590342
		S4_56306388
		S3_2218240
		S2_3788604
		S8_2736478
		S1_56521196
		S6_47072611
		S6_51218496
		S1_7388967
		S1_11550744
		S3_57515827
		S3_5130726
		S7_53163264
		S2_51750760
		S3_61017890
		S3_14340604
		S7_60649532
		S1_57874465
		S5_490394
		S9_9099728
		S6_45144543
		S3_60570948
		S8_5010636
		S5_67722071
		S9_2228497
		S4_46534513
		S5_65930886
		S2_49126548
		S2_2753824
		S3_73319064
		S2_4333952
		S9_57791592
		S4_4649822
		S4_7081487
		S3_55300436

Catogoery	Total	SNPs
		S6_48594453
		S2_55385812
		S8_57948155
		S4_700068
		S4_13903741
		S1_72722117
		S7_8646846
		S9_52032959
		S1_66568100
		S2_58862655
		S2_10977922
		S8_61956241
		S6_49276151
		S6_32048582
		S3_6722675
		S5_9831080
		S1_8975378
		S3_66671217
		S4_53344048
		S2_76975271
		S1_72060297
		S5_62738399
		S7_2771672
		S1_57535874
		S4_12355257
		S1_8641418
		S1_78236263
		S1_67937581
		S6_57941770
		S3_68835516
		S7_10009060
		S1_29361388
		S7_62780901
		S5_1942590
		S1_68804946
		S2_75343556
		S5_15774692
		S1_10678103
		S8_5320773
		S2_67873577
		S9_49963697
		S3_63482751
		S3_620329
		S6_45006708
		S9_58579528
		S7_1958795
		S6_1587099
		S3_14145258
		S8_4310170
		S7_58295882
		S4_2762243
		S8_54372894
		S3_58281913
		S1_7230123
		S9_8377583
		S7_7631208
		S1_72600631
		S7_9515073
		S2_74867336
		S2_12434720
		S6_55684139
		S1_71767476
		S9_57657898
		S5_67404925
		S3_620711

Catogoery	Total	SNPs
		S3_57045753
		S2_73602297
		S9_43747363
		S9_7788427
		S5_10818674
		S1_68783473
		S2_61368170
		S2_74972187
		S6_53439529
		S6_51972137
		S7_64964276
		S7_64236359
		S9_51522118
		S1_78530447
		S8_59745102
		S8_53795129
		S7_56425950
		S1_61737869
		S1_61729961
		S8_55930245
		S1_67739361
		S5_6176970
		S1_68304525
		S4_1734546
		S2_17604467
		S2_74786160
		S1_73736181
		S2_66089988
		S6_975674
		S1_75685810
		S2_75155372
		S3_4450316
		S9_50178715
		S2_64382567
		S7_59390678
		S3_54139292
		S1_78642395
		S2_3428371
		S1_10689323
		S8_57560276
		S1_2914477
		S6_1520113
		S2_65075268
		S2_17980426
		S3_70939970
		S1_76000309
		S2_67656813
		S5_61748997
		S3_65685507
		S6_60460614
		S3_14145199
		S2_58140607
		S2_58421143
		S7_17094520
		S8_3379948
		S3_4223671
		S3_73160964
		S6_44861586
		S1_79195361
		S2_47000446
		S1_11197154
		S6_49221300
		S3_53583420
		S5_1587081
		S3_73887809

Catogoery	Total	SNPs
		S4_55592566
		S4_12708417
		S1_3546395
		S8_3457019
		S1_75372144
		S2_64382565
		S1_65251196
		S7_5171858
		S8_4131941
		S3_63065597
		S6_50507798
		S4_53791391
		S8_60479928
		S3_73934271
		S2_59576784
		S1_67933629
		S1_11205231
		S5_6769980
		S6_41794283
		S3_3757330
		S9_54094860
		S2_40566597
		S2_45247779
		S6_5980654
		S9_54974269
		S9_1147045
		S6_41248890
		S9_50751611
		S2_76885172
		S1_66047119
		S7_1639841
		S1_56295190
		S2_1583802
		S7_2564750
		S4_41917516
		S6_41462500
		S5_927108
		S3_69704028
		S2_67828697
		S6_17122941
		S5_11706211
		S2_65022214
		S2_45816749
		S2_63927936
		S2_12275309
		S1_71371622
		S1_31385254
		S4_6256856
		S8_53990556
		S2_2241184
		S4_51653333
		S7_64416993
		S5_66260507
		S3_65332789
		S4_55546182
		S4_61260646
		S4_19526292
		S6_47785239
		S2_54244771
		S4_15347363
		S6_51925805
		S4_68116896
		S1_73359922
		S7_140769
		S2_68830000

Catogoery	Total	SNPs
		S7_7590921
		S4_1243810
		S6_26214038
		S4_9610858
		S8_35621443
		S1_3013075
		S1_8030669
		S6_44709856
		S6_13948111
		S1_71718771
		S9_58735834
		S8_57953973
		S2_4566441
		S4_16518124
		S5_62418972
		S4_50670392
		S4_55505254
		S2_1030811
		S2_63220918
		S3_51032055
		S8_5009167
		S6_52379005
		S1_74524404
		S5_7341526
		S3_53583414
		S2_12746303
		S2_7133694
		S4_19714157
		S1_71718810
		S4_6500631
		S2_58996764
		S2_65031811
		S6_58217540
		S7_55762916
		S5_16806216
		S6_40930367
		S1_59457630
		S5_11514087
		S3_51516250
		S9_7788344
		S6_47039921
		S7_60226125
		S6_34179986
		S9_58114146
		S3_71698449
		S2_13509820
		S4_3919953
		S9_51739071
		S1_67381965
		S1_6506798
		S8_9742761
		S8_51920191
		S1_73581097
		S1_19596394
		S1_49920331
		S3_6277275
		S1_940277
		S9_58179019
		S4_5065299
		S9_52932649
		S5_2022114
		S6_25575148
		S2_59791352
		S4_48717401
		S6_40800192

Catogoery	Total	SNPs
		S2_6045258
		S1_2313862
		S8_53400299
		S9_8599347
		S5_50603299
		S6_50555266
		S3_57267468
		S2_73187714
		S4_772582
		S9_53396301
		S2_69152502
		S2_12675943
		S8_3613845
		S9_50596104
		S4_20915716
		S9_55969248
		S2_6652587
		S4_22572063
		S4_7538591
		S6_47860741
		S1_77988039
		S5_18346260
		S2_61856243
		S2_66627215
		S8_1747826
		S1_66304610
		S1_51888725
		S4_54119176
		S6_52704204
		S6_29096822
		S8_57571794
		S3_60919521
		S4_63406986
		S4_68098071
		S7_60259869
		S3_20539837
		S2_67765549
		S4_3862215
		S3_2488000
		S1_78776747
		S1_75861461
		S4_37887217
		S3_62392244
		S2_62405526
		S9_49612494
		S4_66042979
		S8_3380603
		S4_12075124
		S2_4554898
		S4_68169827
		S3_4956344
		S1_50787198
		S7_62217640
		S6_14155476
		S4_61883157
		S4_52370213
		S6_34179953
		S2_64750086
		S3_52457556
		S7_63029076
		S2_12754197
		S9_57575587
		S2_75343605
		S1_12932096
		S3_69730830

Catogoery	Total	SNPs
		S1_61604328
		S3_67001532
		S5_6088244
		S3_5477468
		S9_4050227
		S8_41375292
		S9_1097388
		S4_50594683
		S7_54375900
		S4_16187124
		S2_6526421
		S2_57518719
		S2_63408953
		S5_59401183
		S1_57215436
		S6_16684529
		S1_11197150
		S8_53214489
		S6_53335443
		S4_37635164
		S2_9631251
		S4_9881140
		S1_77054839
		S7_39882205
		S3_72144062
		S7_6594081
		S2_1587789
		S1_10687460
		S1_79961970
		S6_1964239
		S1_2860320
		S4_53742057
		S3_64446953
		S1_59457256
		S7_63826165
		S3_72062225
		S2_3602222
		S6_17016112
		S2_67732479
		S3_61517383
		S9_3894720
		S5_66441792
		S2_64144934
		S7_10063263
		S5_8830759
		S8_37963443
		S8_31534740
		S7_63117138
		S7_62271899
		S1_65739449
		S1_52231136
		S4_1193645
		S2_60165255
		S5_6674992
		S2_3883280
		S6_17787464
		S6_5775117
		S3_74120706
		S4_3886408
		S9_8407275
		S8_61921569
		S4_67979552
		S3_73217090
		S5_61061340
		S5_16125588

Catogoery	Total	SNPs
		S7_62029912
		S8_2306116
		S3_56758959
		S3_3902018
		S2_59467254
		S2_39949260
		S3_61024800
		S3_69734209
		S9_57434878
		S2_75343610
		S3_57210598
		S1_49277047
		S1_52089007
		S4_4107323
		S1_53167693
		S4_8586516
		S4_56306380
		S2_70909124
		S2_467562
		S2_62346690
		S2_1124529
		S7_61085967
		S6_53295857
		S5_15081786
		S5_4855591
		S2_12683233
		S9_51417446
		S7_54525583
		S3_69488683
		S2_68194511
		S9_55626942
		S6_54390548
		S7_64737987
		S3_60614845
		S6_5764470
		S3_70605528
		S2_74018535
		S8_56420059
		S2_56056542
		S2_40078266
		S2_69037701
		S3_57907564
		S1_49433065
		S3_52260829
		S6_42300878
		S8_2670965
		S2_65219123
		S6_49427112
		S2_61303394
		S3_15611131
		S2_61392141
		S2_12687932
		S1_62268355
		S8_2374770
		S1_20976780
		S1_3156773
		S2_3785445
		S1_7339832
		S1_75827995
		S2_29883409
		S1_58037275
		S1_54300854
		S3_69421188
		S6_58137478
		S5_12426884

Catogoery	Total	SNPs
		S7_6984828
		S1_22024194
		S8_48961352
		S4_11521363
		S8_39684241
		S2_12466185
		S3_66660321
		S4_7187107
		S4_62988581
		S8_53262099
		S2_49237213
		S4_10430920
		S3_58347393
		S8_53875662
		S2_6220642
		S4_5534738
		S3_60935417
		S3_58381858
		S2_12858134
		S7_59043277
		S2_25591913
		S9_51068874
		S1_2859329
		S4_23737059
		S1_74484811
		S2_58843011
		S7_63414786
		S7_62030204
		S2_1969947
		S2_742820
		S8_51715052
		S1_65353964
		S7_574223
		S1_10101212
		S9_9187150
		S5_67495274
		S1_65219520
		S9_6872845
		S1_60838086
		S2_56925000
		S1_71708051
		S8_56334713
		S1_74928650
		S2_62452419
		S3_73335151
		S2_8430566
		S3_70245265
		S7_7631217
		S6_50583827
		S3_3910493
		S6_52713830
		S9_58070674
		S1_16641396
		S7_64056248
		S8_3193968
		S1_11756712
		S2_67004380
		S1_70900645
		S3_63436360
		S2_68059564
		S6_47306773
		S7_57691468
		S2_75975463
		S2_6139983
		S7_6651628

Catogoery	Total	SNPs
		S1_68236778
		S2_47322000
		S4_52949050
		S3_73493984
		S7_492500
		S3_58846958
		S9_1204277
		S1_59360155
		S4_4541061
		S5_3022170
		S2_3979058
		S5_5251750
		S4_54085162
		S2_66010354
		S3_36253643
		S1_68781637
		S7_41341026
		S1_67679854
		S5_4372554
		S1_46167116
		S2_3602282
		S1_10801408
		S7_6412768
		S3_57223924
		S1_74291725
		S7_58283037
		S4_7468098
		S4_8815021
		S6_36293709
		S1_17468425
		S6_1435190
		S6_29935794
		S5_1443780
		S1_74305101
		S9_5506531
		S5_66608999
		S8_37963221
		S7_6344151
		S8_59643428
		S2_12744928
		S8_32642356
		S4_10174017
		S4_7273384
		S6_53023373
		S2_61029315
		S1_68318303
		S2_66428657
		S2_65877755
		S6_58739490
		S1_3440410
		S4_39898808
		S4_33327401
		S2_2901595
		S2_62132487
		S7_60672642
		S2_19222682
		S6_46768983
		S6_55690117
		S2_55522443
		S2_58285239
		S1_23892403
		S2_64458204
		S1_53481293
		S5_69770101
		S4_5336578

Catogoery	Total	SNPs
		S5_50603266
		S1_51066567
		S3_73425554
		S1_66109408
		S7_65418497
		S5_62499848
		S9_52612582
		S4_988762
		S2_63566632
		S5_32148
		S2_3332675
		S1_16670022
		S1_67198892
		S1_32486907
		S3_61949259
		S1_11562522
		S4_35894799
		S8_1689133
		S2_66338073
		S9_55142266
		S1_78035708
		S1_21692389
		S1_25582348
		S2_62277671
		S1_59795669
		S7_21158082
		S1_21180729
		S7_42860198
		S8_9147019
		S4_9886176
		S9_3881528
		S6_37400387
		S1_66651017
		S4_39854483
		S8_62110166
		S3_68752917
		S4_382357
		S4_15874384
		S1_74196457
		S8_420759
		S6_953226
		S9_52092833
		S2_76820814
		S9_44572780
		S2_69560265
		S8_9587621
		S9_50653954
		S2_32127386
		S4_4197520
		S3_880412
		S6_46718854
		S3_65869638
		S8_2291169
		S3_70879388
		S4_42969118
		S2_9516942
		S4_55553265
		S8_4409292
		S3_53266334
		S7_54787408
		S8_57426996
		S5_1347519
		S2_59492640
		S1_78784545
		S1_48532045

Catogoery	Total	SNPs
		S8_49628773
		S6_50684929
		S1_7525880
		S6_58599823
		S1_78037313
		S1_22638532
		S6_40434376
		S1_14462495
		S7_40823682
		S1_61875723
		S2_63059621
		S3_66023252
		S3_74080198
		S3_56656417
		S6_47112993
		S2_56916119
		S6_47700166
		S1_77161158
		S9_53867589
		S9_960375
		S6_14491376
		S9_4743784
		S1_51498759
		S6_47960203
		S3_6748205
		S6_56333519
		S6_46727136
		S2_59755612
		S4_45663477
		S8_16511395
		S4_50569448
		S9_44207205
		S3_69598988
		S2_5685721
		S8_58488029
		S2_75804321
		S1_77832869
		S4_62901593
		S3_57945423
		S2_55048638
		S8_61956237
		S2_61368328
		S1_50256223
		S7_63065229
		S3_15437589
		S1_64802892
		S9_54094900
		S1_51213627
		S1_79159377
		S3_68055674
		S8_62031065
		S1_73833869
		S4_4224412
		S9_33147164
		S7_37667456
		S5_68867180
		S4_7979118
		S8_61892351
		S1_68301237
		S9_54242667
		S5_61615960
		S6_54267789
		S2_9725899
		S8_50855536
		S5_61792620

Catogoery	Total	SNPs
		S7_65080595
		S3_4244929
		S9_58752953
		S3_73363032
		S1_25827522
		S2_64334143
		S6_32519923
		S1_66407041
		S5_2639673
		S2_65881908
		S8_3457015
		S2_75759051
		S2_9874448
		S1_78486138
		S3_62678362
		S7_2413993
		S3_73184549
		S3_57237991
		S4_32979134
		S2_36529789
		S1_77844819
		S1_53480475
		S7_10000519
		S1_21228526
		S6_46801672
		S2_70918183
		S5_3181331
		S2_2521486
		S6_51222036
		S1_52089017
		S9_57575475
		S6_2665923
		S1_78032500
		S7_58562728
		S2_61032847
		S4_41634203
		S2_65466447
		S2_63996160
		S9_53447757
		S7_52170360
		S3_4558165
		S7_59752053
		S3_56085474
		S1_5892115
		S9_8337133
		S1_7810913
		S1_12920860
		S4_67082962
		S1_57102006
		S7_59858337
		S3_70599147
		S4_10224977
		S7_62927192
		S4_50974937
		S4_16065076
		S6_54825270
		S8_48975690
		S7_433479
		S4_56424053
		S3_57760798
		S1_11198824
		S1_74119561
		#N/A
		S2_63081247
		S1_72911542

Catogoery	Total	SNPs
		S7_12391235
		S6_50734181
		S2_76901386
		S8_50831053
		S4_34487024
		S3_66006806
		S8_11365050
		S5_1761290
		S8_1583995
		S4_50759114
		S1_56295446
		S1_65181092
		S3_67154238
		S4_64648393
		S3_57377055
		S3_68941992
		S8_60139931
		S9_3551776
		S9_3163263
		S1_57328915
		S4_41377895
		S7_64061377
		S8_61547471
		S4_6806301
		S2_4668467
		S3_61706557
		S6_50798037
		S2_185239
		S8_57452757
		S4_20672541
		S3_47964966
		S3_57901123
		S9_50154047
		S9_4575815
		S1_8975296
		S6_48120160
		S3_60919671
		S1_64803275
		S7_17512360
		S6_6108702
		S2_56332752
		S3_70241118
		S5_69879660
		S3_53192667
		S2_58007109
		S9_52158006
		S8_56662129
		S4_1224402
		S4_1004444
		S4_700011
		S2_66090027
		S3_53583425
		S8_46705241
		S2_58213279
		S1_59709014
		S8_51921610
		S5_2915342
		S2_9663698
		S3_50661182
		S4_5894588
		S8_48633894
		S4_7889933
		S3_67023765
		S2_76832014
		S7_63028917

Catogoery	Total	SNPs
		S6_48554245
		S3_73325747
		S1_16203451
		S8_49318873
		S2_66469904
		S1_74907337
		S3_51970970
		S2_63927688
		S3_13769981
		S2_58880705
		S5_1966410
		S1_78818478
		S7_59023238
		S3_7399710
		S3_51424272
		S3_5106789
		S2_67174713
		S1_2088839
		S3_15689578
		S1_22664002
		S1_79364604
		S4_2222132
		S4_3253578
		S1_57883020
		S4_28382971
		S2_64562615
		S9_4314086
		S6_34692542
		S3_2379498
		S1_79793862
		S7_52328166
		S3_71689774
		S2_62393736
		S5_3088504
		S9_52064241
		S6_48594698
		S9_55043314
		S8_57039828
		S5_66114512
		S2_76835087
		S2_10269499
		S1_12730877
		S2_63508657
		S7_58155217
		S6_49377338
		S4_52417620
		S6_38609555
		S2_72258907
		S1_63952038
		S5_9824679
		S1_71756318
		S9_57708044
		S8_9503549
		S7_436462
		S8_5061448
		S4_3232394
		S1_67679451
		S1_60877356
		S3_55006011
		S2_62312354
		S2_58209205
		S1_5806901
		S1_74084270
		S6_50364499
		S6_52515123

Catogoery	Total	SNPs
		S3_6599039
		S1_66395142
		S1_73581128
		S5_62639571
		S5_36122862
		S2_12688121
		S3_73437377
		S1_77156203
		S5_15247772
		S2_56925062
		S1_9039856
		S3_71368790
		S2_67633476
		S1_3546236
		S1_58036752
		S7_58333599
		S3_13405318
		S1_19064371
		S7_8924531
		S2_73381341
		S1_59444130
		S5_69996995
		S3_55111886
		S3_2176137
		S6_31398671
		S1_57273950
		S4_66273496
		S3_56719247
		S5_69998956
		S9_215200
		S3_71236552
		S8_1885157
		S2_58906562
		S1_79161790
		S3_73831916
		S2_8144412
		S2_16391634
		S4_55504251
		S4_5867020
		S1_55049666
		S9_45754771
		S8_16197575
		S2_6207063
		S2_60732783
		S7_65081283
		S3_73631955
		S4_52087119
		S7_56589398
		S3_58400495
		S1_66790772
		S4_1114768
		S2_59734074
		S1_15798024
		S3_54098275
		S2_56966587
		S2_75721200
		S1_67562291
		S3_68599153
		S2_74073523
		S2_59755605
		S2_69137627
		S1_16641209
		S1_72303502
		S1_73462368
		S5_11706318

Catogoery	Total	SNPs
		S7_6373429
		S9_54094888
		S7_62611358
		S8_59997528
		S2_61586193
		S2_60577678
		S8_2630999
		S8_1438074
		S7_63018578
		S2_63584032
		S4_37053419
		S2_10299117
		S3_73887740
		S2_6042680
		S2_58201045
		S3_72064132
		S2_65369664
		S4_13913525
		S4_7273364
		S9_9947584
		S5_66114451
		S1_60816162
		S9_9099731
		S3_74135699
		S8_1234739
		S9_1188905
		S5_67633993
		S2_10517554
		S7_278835
		S8_51316744
		S5_6770030
		S1_9153558
		S1_57130602
		S2_8038719
		S1_8138425
		S1_8879735
		S8_4441306
		S1_59131474
		S3_5787681
		S6_58599840
		S9_58504171
		S5_69852443
		S2_60229534
		S2_71496345
		S3_57213898
		S4_52871177
		S6_72145
		S1_59360078
		S7_61486951
		S5_62418965
		S1_78288218
		S4_7951048
		S1_17685439
		S1_49363712
		S6_53298847
		S2_16612013
		S9_4994288
		S9_3222853
		S3_72851678
		S1_49363696
		S6_953256
		S6_30072244
		S9_1115498
		S5_68952228
		S6_41091889

Catogoery	Total	SNPs
		S5_11615567
		S5_11706304
		S3_51516267
		S5_8834719
		S2_3641625
		S3_52428116
		S5_65909591
		S7_8098766
		S1_71493835
		S8_3110884
		S3_6248659
		S4_7390362
		S9_215195
		S1_6406803
		S5_6065164
		S1_55470039
		S2_63585216
		S7_42744184
		S1_75861012
		S4_53611288
		S4_3745099
		S8_61955768
		S7_56240658
		S1_16553170
		S3_53295411
		S5_6809843
		S1_67781739
		S5_1752430
		S1_79751944
		S1_73675637
		S1_51003649
		S3_69587276
		S5_10052217
		S6_50396693
		S1_59791220
		S7_4554674
		S4_7780319
		S7_62309661
		S3_69847183
		S5_69848161
		S4_14183724
		S9_50738302
		S7_51847245
		S4_9434672
		S9_57068855
		S8_55282983
		S9_1258329
		S7_57573967
		S1_11550669
		S9_18359415
		S7_7904098
		S1_51665934
		S4_10430692
		S3_68941993
		S6_42354450
		S3_57794463
		S4_5107563
		S1_19566444
		S1_66229898
		S9_52207270
		S5_11930922
		S5_9535508
		S9_6372645
		S1_8313982
		S3_73792053

Catogoery	Total	SNPs
		S9_6880642
		S1_68577648
		S2_7133772
		S6_54759097
		S6_7199018
		S9_4693675
		S2_5815067
		S1_80001154
		S3_48751268
		S1_58174719
		S9_51418493
		S4_52473328
		S2_47464159
		S1_78036887
		S8_9687826
		S2_58863541
		S1_72563927
		S4_47808762
		S4_8603072
		S5_66441795
		S6_53005464
		S6_44712010
		S1_79858397
		S1_53970493
		S2_29956815
		S7_40119193
		S1_27982428
		S1_1835189
		S1_52708446
		S4_14926827
		S1_51841705
		S1_66524874
		S8_53370811
		S6_3383223
		S4_4696475
		S8_55930250
		S5_11792661
		S1_16415226
		S1_55563963
		S2_73112181
		S1_67487760
		S5_68429832
		S2_9874327
		S1_68188211
		S2_65799483
		S6_1379485
		S7_52290738
		S2_68945875
		S1_54337800
		S9_51150378
		S2_63195378
		S1_64156474
		S4_52343000
		S1_496319
		S2_75151459
		S4_52416226
		S9_5152006
		S2_61060362
		S6_2712228
		S1_75386751
		S3_73239509
		S9_217552
		S1_66650720
		S1_58096670
		S4_11112340

Catogoery	Total	SNPs
		S5_6101720
		S2_57044542
		S1_10686072
		S1_19378109
		S3_70691364
		S2_12744939
		S1_56381708
		S8_4268635
		S3_60614838
		S2_65031787
		S9_209231
		S6_50876029
		S5_61209427
		S3_4951086
		S5_66622806
		S1_78005543
		S7_64048267
		S1_54569268
		S4_53344036
		S8_3678042
		S8_60393335
		S2_67633094
		S3_73233383
		S1_14438469
		S9_53466969
		S7_12210577
		S8_57596358
		S4_11121795
		S1_30760764
		S3_63251864
		S8_49668151
		S3_66638429
		S3_53394169
		S1_56532044
		S9_42391372
		S8_2653844
		S1_60849636
		S9_2754201
		S3_51034586
		S6_1338156
		S8_31501644
		S3_73163918
		S4_21963926
		S1_11668917
		S4_1275250
		S6_44777400
		S9_10176229
		S2_56440766
		S9_17698171
		S3_2381776
		S3_70901589
		S3_56681254
		S1_7784920
		S1_18563300
		S5_62305080
		S2_39942991
		S1_72938475
		S5_62419489
		#N/A
		S8_47889831
		S2_12952863
		S1_77436175
		S5_2693239
		S2_4555004
		S9_59169357

Catogoery	Total	SNPs
		S4_50758896
		S9_50520593
		S2_57705156
		S9_53493412
		S6_49768157
		S7_254017
		S5_54661204
		S6_31579660
		S4_1130009
		S2_61786330
		S1_2030897
		S2_12688029
		S2_6785049
		S6_60688770
		S1_9894069
		S6_41859558
		S3_72443128
		S2_10832247
		S8_55521892
		S4_2151558
		S2_13166012
		S1_9068942
		S4_952935
		S1_3129029
		S1_7643982
		S2_57433551
		S6_46483037
		S5_9542640
		S5_9377366
		S7_62716936
		S1_2005644
		S9_56987016
		S6_48683434
		S4_665908
		S7_54698334
		S8_55615179
		S7_887735
		S2_75804348
		S8_57426990
		S1_58096655
		S8_50405721
		S2_63220922
		S1_55830611
		S6_53726946
		S2_12690962
		S9_57859057
		S5_11069403
		S6_6197449
		S9_52077452
		S1_62840639
		S4_9764501
		S8_3018100
		S6_51228996
		S4_66609612
		S6_49221210
		S2_12466192
		S1_75620809
		S8_49102785
		S1_62068223
		S1_80487122
		S2_14478064
		S5_1291887
		S2_3949749
		S2_4149589
		S4_45490388

Catogoery	Total	SNPs
		S2_12746319
		S6_47901257
		S3_72654495
		S3_20398726
		S3_69847121
		S3_34504575
		S4_53816456
		S3_68831857
		S9_5861343
		S4_5039097
		S2_55048650
		S4_52849425
		S2_73664874
		S6_53576445
		S2_56923090
		S4_33698441
		S2_61859440
		S1_74778961
		S9_51461369
		S6_3200956
		S2_6488540
		S2_6864353
		S5_9537232
		S6_52016778
		S1_63004838
		S5_2596611
		S6_5732392
		S9_6791210
		S2_76430595
		S2_16852931
		S9_40978338
		S1_30958992
		S3_67029963
		S1_2740106
		S9_3025632
		S9_44575474
		S5_9537454
		S8_60479910
		S9_2744838
		S5_12294673
		S5_62405522
		S3_61949275
		S1_74119555
		S7_500842
		S4_34291199
		S9_8394115
		S4_666407
		S6_5961514
		S6_41925281
		S1_7784910
		S8_3299823
		S8_47592537
		S2_60965841
		S3_53546949
		S2_64465203
		S8_61925204
		S4_54095714
		S1_65740377
		S6_47960267
		S3_51903121
		S1_8822062
		S9_44575324
		S9_52076697
		S2_69155197
		S1_7069590

Catogoery	Total	SNPs
		S8_1567179
		S2_73486856
		S6_45544908
		S5_21959528
		S4_52473557
		S3_7384971
		S5_61168140
		S8_4402278
		S5_225941
		S2_77032749
		S6_49221310
		S8_41088404
		S1_2866090
		S5_1161407
		S4_52871198
		S1_68301231
		S1_20372050
		S3_69917980
		S1_79701405
		S5_63507709
		S6_31076137
		S7_58690963
		S4_42830368
		S5_6894096
		S3_59704860
		S1_60657131
		S1_58612172
		S1_62222674
		S3_5164852
		S6_3082333
		S4_4189605
		S8_4310181
		S1_80077554
		S8_56686327
		S2_59619373
		S1_78970946
		S1_72474528
		S8_51198133
		S6_30331779
		S6_55684141
		S8_55930275
		S3_65348741
		S2_59791375
		S4_7889915
		S4_5025237
		S4_67970834
		S2_58025287
		S9_5365185
		S1_59362325
		S9_1258258
		S9_1260270
		S6_44752180
		S7_6593995
		S5_69823139
		S5_4355126
		S1_52349784
		S2_12357438
		S2_7736120
		S8_61646355
		S5_67362975
		S1_50830176
		S1_68236839
		S3_69256854
		S1_56295591
		S8_60337802

Catogoery	Total	SNPs
		S1_12394002
		S6_1374716
		S5_59372523
		S1_67927855
		S2_63520897
		S7_2562550
		S3_68367370
		S6_58759527
		S2_6139974
		S6_51851606
		S6_50881736
		S7_4745777
		S4_6221240
		S3_67492313
		S1_78509751
		S2_17507747
		S9_57563281
		S2_64144910
		S1_5730542
		S3_69704218
		S2_13868792
		S1_74305201
		S3_62858494
		S3_61246821
		S2_6045447
		S8_57051591
		S4_4991035
		S3_68919618
		S1_26894221
		S3_74068834
		S8_29984522
		S2_74972183
		S2_59198381
		S4_6902960
		S2_61926720
		S7_5301886
		S1_15714608
		S3_64937605
		S3_69704150
		S2_56332752
		S1_16203493
		S7_889865
		S6_53726922
		S6_38608639
		S9_44040235
		S5_67404867
		S1_31001812
		S1_79434627
		S2_73366263
		S8_14632576
		S2_10740947
		S6_47043776
		S6_25575256
		S5_62493274
		S6_49333645
		S3_20127194
		S7_25790415
		S3_68919620
		S5_2829049
		S3_62269076
		S2_61857097
		S7_54698250
		S1_987717
		S2_62272851
		S6_47355740

Catogoery	Total	SNPs
		S4_56427343
		S6_493728
		S3_2112866
		S2_64477632
		S4_56621745
		S3_2379478
		S1_79961961
		S5_69699553
		S4_52949059
		S8_4934941
		S7_63067967
		S4_53052862
		S6_52214940
		S2_54203874
		S1_59709012
		S8_37494775
		S8_53982172
		S6_51982100
		S3_58716013
		S8_1123441
		S7_2118500
		S5_10818673
		S4_66386098
		S8_1156260
		S5_20978344
		S1_46547356
		S6_25968205
		S1_59360156
		S4_55591398
		S1_12920909
		S1_18202911
		S1_8739667
		S2_56168323
		S4_1646250
		S2_72588688
		S1_19184960
		S9_50867922
		S3_65692238
		S2_60595346
		S1_18896240
		S7_64967149
		S1_8819523
		S9_1531043
		S2_76798911
		S3_73887803
		S3_65328171
		S3_70569149
		S4_48058379
		S3_54138336
		S5_12521062
		S5_13605647
		S1_17426841
		S8_5781774
		S2_4206120
		S3_73010555
		S9_1094796
		S2_56922502
		S2_8335669
		S8_2391906
		S6_50583836
		S7_65080585
		S2_64088650
		S1_6335784
		S5_5273705
		S1_11205154

Catogoery	Total	SNPs
		S9_8849586
		S8_4056524
		S3_3700827
		S6_47179468
		S4_42264600
		S3_63301745
		S8_49156149
		S6_8213053
		S8_56662148
		S8_15936480
		S3_69496545
		S7_1163214
		S8_5255537
		S3_52417071
		S3_60576085
		S8_32316976
		S5_67495278
		S9_59340345
		S2_57518716
		S8_3264485
		S4_34493699
		S3_69900012
		S1_71822175
		S3_68664313
		S4_49581038
		S9_53930964
		S8_61980175
		S7_492236
		S2_65497447
		S1_17685616
		S1_12967570
		S6_16376659
		S4_710634
		S3_51525606
		S7_3646965
		S7_8924529
		S8_4685737
		S2_66930674
		S1_8138444
		S9_52254308
		S6_72133
		S3_68326264
		S1_80077482
		S3_57937676
		S1_72558654
		S2_76938852
		S3_47800173
		S9_52344225
		S1_56993353
		S1_70242896
		S8_4542297
		S3_65421714
		S3_67534160
		S1_841
		S8_57441521
		S3_55801372
		S9_40978335
		S9_57766617
		S8_2614974
		S9_10666771
		S7_62113550
		S7_56997690
		S3_1969732
		S2_62729862
		S8_2874236

Catogoery	Total	SNPs
		S2_73522521
		S1_9921050
		S1_11205226
		S4_8601430
		S3_72659678
		S4_7468082
		S3_3904040
		S2_66579415
		S2_64391924
		S7_37667460
		S3_65348746
		S3_5565450
		S9_58070650
		S4_34490623
		S9_2931133
		S2_48170481
		S1_72488191
		S7_54684270
		S2_60137826
		S2_320417
		S5_67290266
		S3_4956347
		S3_71368362
		S5_6166266
		S2_59626180
		S8_59643431
		S6_47955698
		S3_67001535
		S1_9980225
		S6_44828808
		S3_3700934
		S7_60881335
		S8_59673167
		S5_62172945
		S5_6894112
		S8_54938234
		S5_62493118
		S1_57932775
		S9_14174740
		S1_78749832
		S3_74105530
		S1_71822744
		S5_2542191
		S1_30344494
		S1_16415246
		S8_16313415
		S5_67558280
		S4_37132641
		S2_6045449
		S1_2297974
		S2_53885636
		S1_60414877
		S2_11765741
		S1_9066117
		S7_63787152
		S8_16796096
		S2_18573319
		S2_40566653
		S1_8441795
		S4_2151792
		S7_63392103
		S2_50059603
		S9_50867944
		S4_6941989
		S6_48562418

Catogoery	Total	SNPs
		S7_5172187
		S3_70583922
		S5_10879180
		S8_59192344
		S3_60581839
		S2_73675538
		S2_62317801
		S3_54117553
		S1_73777110
		S1_77686315
		S2_10860276
		S9_5829139
		S9_6492734
		S7_6671221
		S4_1570073
		S7_3637487
		S5_12368789
		S4_43740245
		S9_141331
		S5_36836033
		S9_58179028
		S4_7247480
		S2_4147120
		S3_60919779
		S3_38729813
		S1_9066107
		S4_8815510
		S7_6984803
		S7_484179
		S3_65332742
		S5_10818617
		S1_6298584
		S1_66393754
		S3_47941671
		S5_18483189
		S8_4268961
		S6_1994510
		S3_73484597
		S3_53394322
		S1_17426954
		S1_55504266
		S1_64126490
		S2_51823660
		S7_1552880
		S7_2117998
		S2_63520660
		S1_79363831
		S9_8626262
		S8_61636703
		S4_12350801
		S6_47074798
		S5_66734798
		S2_75153893
		S8_49763479
		S2_6950114
		S7_12094595
		S7_57053027
		S8_2767760
		S7_62418795
		S3_54540937
		S8_34341425
		S9_49267005
		S5_8658696
		S8_19009770
		S6_30655842

Catogoery	Total	SNPs
		S1_71831816
		S3_5617812
		S1_59874393
		S3_74143085
		S1_73857468
		S8_44419050
		S1_12968368
		S9_190656
		S9_8192301
		S6_47901754
		S3_63577212
		S6_51865359
		S4_62988578
		S2_2235780
		S1_17468368
		S3_40343
		S8_56501491
		S7_58673774
		S7_2713929
		S4_7868624
		S2_3641324
		S9_54111752
		S1_72848004
		S1_72142848
		S1_11600176
		S1_6244087
		S8_58766382
		S5_6088244
		S2_3427353
		S2_10525743
		S3_70839423
		S6_41825606
		S1_17489158
		S1_25902450
		S4_4682798
		S2_63811597
		S2_61739772
		S6_53576446
		S6_47936520
		S3_72414673
		S1_66043531
		S3_69485307
		S5_6769983
		S3_47221421
		S2_19732776
		S1_56695772
		S5_6065162
		S4_1110345
		S5_63235230
		S4_2894283
		S2_64088625
		S2_71254426
		S1_60148052
		S8_51871871
		S3_38419881
		S7_854856
		S8_57593553
		S2_67732479
		S5_15116559
		S1_52333070
		S8_6158436
		S3_58901018
		S2_67810209
		S3_2117222
		S8_57577208

Catogoery	Total	SNPs
		S5_10231163
		S3_15611145
		S1_66368858
		S7_1570471
		S5_62770294
		S2_66339820
		S1_55049627
		S5_60649297
		S2_69135584
		S6_35584099
		S1_2271926
		S9_9099729
		S5_11310290
		S4_53289418
		S4_39854477
		S5_58383653
		S6_46811631
		S1_7530607
		S1_11197155
		S9_2661122
		S9_7350298
		S1_14080890
		S1_62967228
		S1_2741108
		S9_58540469
		S6_31120306
		S5_5041430
		S2_73877109
		S6_41098370
		S1_21254058
		S2_65921400
		S8_9590447
		S1_7474880
		S1_77829706
		S4_35858110
		S5_15793596
		S6_47643487
		S5_11328784
		S5_63471128
		S2_64144925
		S8_47639760
		S7_64554801
		S1_55817592
		S2_1030470
		S3_4756380
		S6_53240820
		S1_16399103
		S4_56306385
		S6_53579050
		S1_59567034
		S2_75975460
		S8_61606876
		S2_67282449
		S8_46431799
		S8_55498817
		S5_67071224
		S1_77423995
		S5_61208122
		S4_66386086
		S1_72976370
		S5_63070184
		S4_50569620
		S1_8820960
		S8_5076037
		S3_67871390

Catogoery	Total	SNPs
		S5_62379334
		S7_3706382
		S7_9788489
		S8_2767675
		S1_12305187
		S8_50405574
		S3_811885
		S2_67234915
		S4_27570683
		S8_49573273
		S8_58901767
		S4_15886344
		S6_47179561
		S2_62974138
		S3_59692572
		S9_4693937
		S9_5829136
		S8_5010636
		S1_71371606
		S1_72308374
		S7_9515669
		S2_72615446
		S2_69037712
		S3_70861809
		S9_53720737
		S4_13916462
		S6_51803555
		S1_15854593
		S1_67679851
		S2_72264310
		S1_9154882
		S9_56542539
		S8_57451738
		S5_8210115
		S6_35109620
		S1_21732436
		S7_52328072
		S3_63301473
		S5_13606294
		S8_5263004
		S3_69847064
		S7_2771644
		S8_39383364
		S7_40828058
		S6_58535356
		S2_62739827
		S2_54200588
		S2_72606470
		S5_5248847
		S7_7631214
		S6_1520115
		S2_61140431
		S7_15941780
		S1_53480696
		S8_3179512
		S3_69485300
		S3_66660290
		S4_67087257
		S7_4546295
		S2_71215831
		S4_62909177
		S2_18758075
		S6_46497366
		S2_53349071
		S6_49718550

Catogoery	Total	SNPs
		S2_61833910
		S2_73318009
		S2_54254793
		S3_57869826
		S6_38178260
		S6_47063076
		S2_23316259
		S2_32127462
		S9_58675219
		S1_54031576
		S1_15293886
		S1_78776002
		S4_52450682
		S1_14860749
		S7_1217889
		S5_11029930
		S5_10250557
		S1_80064206
		S1_56695765
		S4_66125717
		S5_61127889
		S2_65106963
		S7_64774489
		S1_2365181
		S9_55626970
		S1_11127531
		S6_40986639
		S2_56045408
		S3_69692709
		S2_12357474
		S1_72938612
		S1_72879165
		S2_4554917
		S8_60447299
		S9_1194196
		S3_67762395
		S2_59637003
		S7_40625847
		S7_63639779
		S8_3218213
		S6_50555254
		S3_58281918
		S9_50517893
		S9_58916306
		S2_10290141
		S2_64382573
		S2_68060278
		S2_63220920
		S1_59441687
		S2_56332752
		S2_10905145
		S3_69692642
		S1_59159855
		S1_58035752
		S1_59131760
		S8_60010367
		S7_60936898
		S1_22260273
		S1_72373901
		S2_39135899
		S2_6864351
		S6_55690119
		S1_7624963
		S1_11550686
		S9_52654359

Catogoery	Total	SNPs
		S6_50696779
		S3_70752188
		S5_68192304
		S7_24083037
		S9_1525043
		S6_47627014
		S9_217554
		S5_4752799
		S1_380254
		S6_47305919
		S1_11566640
		S6_13695603
		S6_47901264
		S8_9503620
		S6_1587010
		S4_58178133
		S8_3183163
		S7_7825622
		S1_59131453
		S6_51082745
		S4_33698443
		S9_1111022
		S3_69847330
		S3_61711104
		S5_11792455
		S2_64563631
		S9_214851
		S7_63739638
		S1_9809437
		S2_73430831
		S8_57452904
		S8_3231057
		S6_52991218
		S9_5159828
		S5_1327061
		S7_65029422
		S3_1905133
		S1_60005616
		S1_9066138
		S8_15800184
		S2_66457117
		S7_2922555
		S7_62396534
		S5_1068107
		S4_48848271
		S4_20672527
		S1_55509706
		S8_10179380
		S6_58081930
		S3_3480331
		S3_63107809
		S1_1971949
		S7_42680889
		S2_19732742
		S1_23805678
		S8_7197922
		S8_3103699
		S2_57255727
		S1_64103570
		S4_48345684
		S4_5536019
		S1_7343310
		S3_5659711
		S1_60148706
		S1_68725335

Catogoery	Total	SNPs
		S9_53229771
		S2_3388918
		S3_74147906
		S8_59750957
		S3_59791691
		S6_1126805
		S1_78787997
		S3_73887806
		S7_60723643
		S3_60581652
		S7_7878422
		S4_58273426
		S2_61535466
		S2_2800956
		S4_34150726
		S3_72514853
		S2_56924840
		S1_76494959
		S6_14453011
		S2_9371850
		S8_1239918
		S1_7857257
		S6_47179551
		S3_57213858
		S2_2488277
		S8_3217762
		S9_8407285
		S6_49934312
		S6_26232705
		S7_63598442
		S3_5585039
		S2_66280924
		S3_6755997
		S2_61798510
		S5_61168857
		S1_62856693
		S8_3729267
		S2_6036354
		S2_67837720
		S8_3529863
		S4_19165591
		S3_55265390
		S2_22132210
		S2_60560620
		S3_63046012
		S7_60260315
		S6_53857090
		S4_35601841
		S5_15774692
		S2_73835247
		S2_69068634
		S1_64272954
		S8_59195198
		S2_13167468
		S9_1096784
		S6_46718885
		S9_7886948
		S8_4934904
		S5_7022245
		S8_60970343
		S1_14090641
		S5_8068937
		S2_59913692
		S4_66042974
		S1_78725078

Catogoery	Total	SNPs
		S1_51003641
		S7_63093025
		S4_33300785
		S2_54200762
		S6_29284543
		S4_1814216
		S7_58678093
		S1_29777702
		S5_66972699
		S3_64354537
		S8_58901847
		S7_54310018
		S9_5184355
		S1_77100623
		S2_32799661
		S2_361228
		S3_70245419
		S3_812196
		S3_70135425
		S4_7470107
		S2_69829342
		S2_65330875
		S2_19609862
		S1_2871569
		S2_16850751
		S1_70466572
		S3_68031538
		S1_17106135
		S6_47045249
		S3_48020894
		S8_58676551
		S4_49329179
		S3_15211129
		S6_60594858
		S5_2889165
		S5_4616976
		S2_63426474
		S2_10269492
		S9_51740630
		S9_1260338
		S9_1252839
		S8_1839665
		S2_56191730
		S3_74146234
		S4_7959994
		S3_58246385
		S6_51404443
		S1_79195167
		S2_74682258
		S2_6611946
		S3_54545113
		S5_50490612
		S1_63706797
		S6_47959666
		S1_53481415
		S1_72691233
		S2_61085182
		S6_52883012
		S3_6754771
		S9_44061663
		S2_75753273
		S1_8644866
		S1_64560396
		S6_47291823
		S1_6462604

Catogoery	Total	SNPs
		S2_63508669
		S1_80684229
		S1_21125308
		S1_65672078
		S1_7621112
		S8_50142526
		S1_67679846
		S5_1326928
		S5_2693011
		S2_41600658
		S7_62782579
		S3_73084463
		S8_17136113
		S6_35569175
		S3_7387758
		S9_48859439
		S8_57948158
		S4_33376303
		S4_16584771
		S5_66441871
		S7_62127559
		S3_56681997
		S3_51506181
		S5_12084419
		S2_49673135
		S9_8702778
		S8_53538445
		S1_11708098
		S1_16415204
		S1_14875273
		S1_59759835
		S7_6679582
		S6_53583068
		S1_72372621
		S2_16390698
		S1_59328801
		S1_59893035
		S1_67659930
		S2_71570208
		S8_1880678
		S1_22260225
		S1_65223570
		S2_382130
		S7_64070175
		S3_51704461
		S8_3065486
		S3_5164857
		S1_69088600
		S1_57204829
		S1_12945067
		S6_50726474
		S9_49281191
		S4_56200680
		S9_41589297
		S8_53262259
		S7_64745931
		S2_55384043
		S6_2489978
		S5_10354370
		S7_1163192
		S1_18299354
		S5_6101733
		S9_4743783
		S5_3179748
		S4_9633204

Catogoery	Total	SNPs
		S1_63253474
		S3_69485298
		S4_836920
		S1_72605779
		S2_57448587
		S1_52255940
		S2_10266038
		S2_6759092
		S3_53588189
		S6_46494866
		S3_51904166
		S3_45948344
		S6_26090600
		S6_53638835
		S4_11121768
		S1_16543098
		S2_9516943
		S2_6239359
		S5_6809683
		S1_8806052
		S8_1880655
		S3_6763711
		S7_64669760
		S5_68319537
		S2_2781003
		S8_4401914
		S2_64495135
		S9_51890195
		S5_68877545
		S7_64100629
		S3_54098280
		S1_59383258
		S2_4554937
		S4_4116030
		S1_57127936
		S9_4637487
		S2_6139961
		S7_61598347
		S4_6756980
		S3_73537363
		S2_60678877
		S2_30234059
		S1_78036923
		S5_1608521
		S2_59945478
		S1_15897130
		S7_8521103
		S2_13133097
		S4_25327664
		S1_59362299
		S3_69885978
		S4_1608099
		S2_13459051
		S5_69848155
		S1_58193692
		S4_7169869
		S3_3757320
		S5_2227804
		S7_6972779
		S3_56069569
		S7_52290734
		S1_3608512
		S2_62309924
		S8_61921713
		S7_6984834

Catogoery	Total	SNPs
		S1_71632652
		S7_62716941
		S6_53639698
		S4_58333350
		S4_42859901
		S2_8670129
		S3_3900247
		S2_14001693
		S1_19554732
		S2_15077715
		S1_72586823
		S2_31842657
		S9_14324410
		S2_71569863
		S8_54956241
		S1_31205592
		S5_4702983
		S6_61250660
		S8_17245033
		S3_71067257
		S1_55518621
		S9_52343072
		S2_65877756
		S1_2466270
		S2_6741890
		S4_7875393
		S2_58681044
		S1_22396990
		S1_77726734
		S5_61286602
		S6_58878709
		S7_56269844
		S8_3366613
		S6_47539286
		S4_50974302
		S2_74972189
		S6_38162125
		S4_55526458
		S9_1698860
		S1_12267255
		S4_53832153
		S2_69347973
		S6_61156532
		S3_48915590
		S2_58682747
		S4_5894353
		S1_25946835
		S3_60933698
		S3_68980515
		S7_62909834
		S1_10734484
		S1_15031544
		S5_63070153
		S1_71755417
		S9_8337208
		S6_47955709
		S3_52457549
		S1_63011694
		S9_4266593
		S5_63240854
		S5_67059025
		S1_51841680
		S1_74880249
		S1_75801512
		S1_79438065

Catogoery	Total	SNPs
		S8_18049376
		S4_14926830
		S1_20564833
		S3_71245219
		S7_25861
		S7_12015926
		S2_3123786
		S7_889726
		S5_20868092
		S1_28340560
		S3_4872727
		S6_17497706
		S3_73759081
		S4_56568021
		S2_1007342
		S2_63693112
		S6_53254127
		S2_300726
		S2_60448536
		S2_17707490
		S2_17589977
		S1_57110296
		S1_7586189
		S1_8881256
		S9_52327199
		S8_57569318
		S1_8981829
		S1_59338088
		S7_64223415
		S1_59338088
		S2_67327852
		S8_1438146
		S8_43945875
		S9_51718677
		S2_57258219
		S4_26378371
		S6_50696773
		S9_48996380
		S8_61636548
		S2_75808029
		S5_63419007
		S6_1668325
		S5_43169525
		S2_59799830
		S1_78275181
		S9_4690131
		S2_6139976
		S4_5947124
		S1_58193600
		S6_45570673
		S6_41584364
		S1_1547288
		S6_47179645
		S3_13445849
		S1_55832084
		S2_64465350
		S4_12679976
		S4_1609545
		S3_63161772
		S2_75170660
		S1_71174950
		S1_54325428
		S1_51841721
		S1_9066135
		S8_44769137

Catogoery	Total	SNPs
		S6_49841101
		S1_8442054
		S2_75804503
		S2_10219318
		S4_10307478
		S4_54086429
		S3_72414667
		S2_77071379
		S6_41220195
		S2_56440837
		S2_7829278
		S1_7403614
		S4_7084384
		S3_73934704
		S5_1122078
		S6_50307628
		S9_58332250
		S9_56572742
		S1_7412731
		S1_26892939
		S8_57685256
		S4_9492754
		S1_7388959
		S1_79368928
		S1_9068994
		S8_60094696
		S3_69918068
		S5_4526569
		S4_66123788
		S1_17685401
		S1_79510155
		S3_73233444
		S7_60684361
		S4_54345986
		S4_7436050
		S7_62494210
		S1_11197484
		S8_60933904
		S2_66005455
		S1_52349789
		S4_35170142
		S7_7036460
		S1_30824364
		S9_5465348
		S9_1813476
		S2_73494298
		S2_9463897
		S9_49551667
		S9_55837108
		S2_73115393
		S4_7550089
		S8_59597271
		S5_65523842
		S4_61278102
		S4_67124704
		S2_56514518
		S3_54690243
		S6_40987298
		S6_54168304
		S8_2374789
		S2_65043032
		S1_71831747
		S9_6371717
		S2_65028947
		S4_21148360

Catogoery	Total	SNPs
		S1_15315270
		S1_76914117
		S4_11072586
		S1_71688735
		S9_4690054
		S1_18967250
		S5_67784070
		S4_7869888
		S2_12357931
		S4_525376
		S6_18079667
		S1_61605951
		S5_406934
		S5_62493111
		S4_40072099
		S3_74054142
		S5_3397179
		S8_53982118
		S9_51705472
		S9_971828
		S7_9515484
		S6_53240799
		S5_68111653
		S2_6759496
		S2_75655156
		S2_61211198
		S8_4310376
		S5_61127884
		S2_6612478
		S3_68058266
		S9_50448596
		S1_15854569
		S6_51320772
		S2_6182753
		S9_57247166
		S2_73366940
		S9_52415574
		S5_67596483
		S6_3980213
		S5_63235227
		S2_72650746
		S7_5538133
		S9_1260638
		S8_34726702
		S1_77100620
		S9_47251762
		S1_80077529
		S7_63734794
		S5_65524470
		S2_55357470
		#N/A
		S6_56756451
		S9_52582223
		S6_58863285
		S1_79775169
		S1_11127555
		S9_54605596
		S1_17746737
		S7_5171929
		S1_72373911
		S6_32519933
		S4_62906657
		S9_57684260
		S5_59271627
		S3_54129610

Catogoery	Total	SNPs
		S8_61954450
		S1_7856669
		S7_62806306
		S3_73216549
		S6_45160546
		S5_1649772
		S9_3351147
		S2_61060073
		S2_3641557
		S2_60986376
		S6_8758835
		S3_71519745
		S7_54307489
		S3_31348023
		S9_42208682
		S3_61710966
		S2_49314529
		S7_15739638
		S2_61427936
		S9_1663055
		S5_61929614
		S1_18890738
		S9_53925504
		S6_58812600
		S3_6242259
		S6_53942655
		S2_13177227
		S1_7069623
		S3_61711108
		S3_5659694
		S3_61931993
		S9_3184853
		S4_50759132
		S1_3351658
		S4_8158977
		S4_2004722
		S4_53791384
		S4_9371716
		S7_24732044
		S1_74100971
		S2_69715650
		S8_42232511
		S6_41827772
		S1_73899479
		S2_59238552
		S7_6451642
		S1_7225921
		S1_21732421
		S8_60362310
		S8_50405718
		S8_45639020
		S7_39535394
		S1_20426842
		S3_68981252
		S9_8407245
		S5_7341860
		S6_37906681
		S1_1828631
		S2_9657274
		S1_60837186
		S2_19502463
		S2_60659342
		S8_3230471
		S4_55504147
		S3_70711191

Catogoery	Total	SNPs
		S4_66306332
		S2_65588210
		S1_62125354
		S3_47933959
		S5_2693011
		S4_34662264
		S3_69102557
		S1_66651024
		S8_33925166
		S6_35784842
		S1_53974673
		S9_51705777
		S7_58486821
		S2_61561817
		S2_56869874
		S2_61926721
		S5_12827450
		S4_4154113
		S7_62123261
		S7_62194050
		S2_32082843
		S8_5051886
		S8_57571694
		S2_39376131
		S9_22629599
		S2_6628150
		S4_51772396
		S7_62217670
		S4_2626987
		S1_12797908
		S1_13095879
		S1_72722103
		S2_59921833
		S6_47278611
		S6_8342666
		S3_58370459
		S1_10593921
		S2_59706892
		S9_51705770
		S2_59972370
		S6_50309386
		S4_6255348
		S1_67713905
		S1_76020352
		S9_1071472
		S2_66426395
		S2_69678359
		S1_17816732
		S1_7936735
		S3_61423368
		S5_11014851
		S2_4554953
		S5_65842216
		S9_215205
		S1_52230633
		S9_53301383
		S6_50567817
		S1_72724628
		S8_46410418
		S3_72617039
		S5_61864532
		S6_453019
		S2_1030812
		S1_18973234
		S1_63011703

Catogoery	Total	SNPs
		S2_6207070
		S5_6101725
		S9_1176610
		S3_57816851
		S7_57643247
		S9_1646670
		S9_54567150
		S1_66322633
		S1_28396796
		S1_80320598
		S3_71652664
		S2_56332752
		S1_72772071
		S2_158578
		S6_49336636
		S3_16556762
		S2_6204333
		S1_51240469
		S4_6982855
		S4_15684165
		S4_2641217
		S2_8438
		S1_64361916
		S1_5800574
		S2_4581269
		S5_14646760
		S5_8160930
		S3_70256138
		S9_2824989
		S1_58037245
		S9_17702153
		S2_59655479
		S3_73239325
		S1_28959235
		S4_18493175
		S2_73908229
		S2_72264270
		S2_61444533
		S6_45509834
		S4_1008393
		S4_47438931
		S1_17824252
		S1_65195969
		S2_64471069
		S6_36795115
		S5_12278979
		S3_67966299
		S2_65218961
		S6_1379434
		S7_58333589
		S1_5806904
		S1_21849075
		S4_2302908
		S1_18967257
		S1_55709374
		S1_2554816
		S2_29536052
		S3_15435900
		S3_12695173
		S2_62925036
		S4_7390350
		S8_51921780
		S6_51972688
		S2_60970174
		S1_12785090

Catogoery	Total	SNPs
		S2_17210935
		S9_51911939
		S7_59390486
		S6_5980563
		S3_5644993
		S7_64000221
		S6_1587014
		S7_62460669
		S5_9181002
		S2_6607847
		S3_16399907
		S4_9370820
		S3_73446046
		S2_76901401
		S2_16294923
		S8_60139934
		S8_60912482
		S9_50596053
		S1_13996735
		S6_58575369
		S3_57339717
		S5_1249583
		S1_49363691
		S4_12085528
		S7_53150031
		S5_18481273
		S8_56542070
		S2_61589025
		S7_56556006
		S6_58759538
		S7_124710
		S6_53728237
		S4_19935220
		S4_67915661
		S3_61487705
		S1_68247462
		S3_67910564
		S2_37085048
		S9_3821786
		S7_60102977
		S8_55615031
		S6_40439949
		S5_61168142
		S3_13434182
		S2_5389760
		S1_10687798
		S2_805338
		S2_56986086
		S1_80151659
		S6_52730777
		S4_8520439
		S2_53430284
		S8_51715224
		S6_47179454
		S2_64350270
		S2_62350630
		S5_65563148
		S4_18229286
		S6_38175754
		S4_51951342
		S6_53515985
		S2_64072269
		S5_15253440
		S8_60087259
		S3_71370672

Catogoery	Total	SNPs
		S7_9082788
		S6_40512595
		S1_67674102
		S7_65129262
		S1_59131477
		S2_56332752
		S4_5069962
		S5_67494930
		S4_20672537
		S1_71818578
		S5_61168342
		S8_45238288
		S6_60905357
		S6_13760238
		S1_18905298
		S1_48971898
		S4_54204333
		S1_63359080
		S1_77153143
		S3_72171197
		S2_60965839
		S5_6809726
		S5_6992416
		S2_9854714
		S2_61856114
		S2_8885777
		S3_65628149
		S9_2970148
		S7_58607539
		S4_66307094
		S5_69067563
		S7_56240624
		S2_64988009
		S9_57785219
		S7_6507374
		S2_9492236
		S2_57862911
		S4_9016582
		S4_2002539
		S8_56721716
		S7_52893271
		S8_3579334
		S2_13907401
		S2_67800526
		S1_18956475
		S7_433477
		S7_63790845
		S3_64354506
		S7_2321474
		S8_37008608
		S4_58254273
		S3_57244166
		S5_65523628
		S9_57717096
		S9_58742743
		S4_39813404
		S8_3264568
		S2_1587786
		S8_51258974
		S3_68055681
		S6_51224256
		S1_67949913
		S2_9218563
		S6_50309385
		S1_7018408

Catogoery	Total	SNPs
		S7_61598318
		S6_1067403
		S2_74073526
		S1_17466772
		S1_19154384
		S1_78180847
		S2_76657364
		S2_6015106
		S2_59247244
		S6_60453534
		S1_59457301
		S3_5505072
		S4_12081871
		S1_2135417
		S6_51114435
		S4_7539067
		S1_18954680
		S6_54583336
		S7_59953998
		S7_2907661
		S5_67433963
		S8_1753892
		S7_62611647
		S6_54825316
		S1_11191687
		S9_55142131
		S7_8824238
		S4_66273495
		S2_67198139
		S6_47105020
		S2_69135671
		S4_39940887
		S4_26389668
		S1_22260272
		S6_40434379
		S8_29793075
		S4_20672539
		S2_10992434
		S6_1344827
		S9_57247180
		S1_76324710
		S4_46872683
		S5_51255908
		S3_73514275
		S1_74940022
		S1_55413946
		S5_10213584
		S3_62269048
		S3_975720
		S6_6493610
		S7_63828573
		S8_2271601
		S6_47306839
		S4_50857310
		S2_54245111
		S7_63572009
		S4_137746
		S3_73040875
		S2_65329633
		S5_11763348
		S2_77734402
		S1_12032054
		S1_66793773
		S1_27152731
		S3_34504561

Catogoery	Total	SNPs
		S1_7822996
		S5_69822715
		S1_69080916
		S3_5787681
		S7_61166619
		S3_48916582
		S2_56290224
		S7_61625490
		S1_13891948
		S1_32445592
		#N/A
		S1_75873794
		S3_72932776
		S2_56924271
		S1_64803280
		S3_69832111
		S8_18730633
		S7_3694754
		S4_24157749
		S3_15959207
		S2_8707821
		S6_42482914
		S2_43753594
		S2_63357101
		S1_9894079
		S7_2923800
		S8_61657732
		S6_58535373
		S7_53163825
		S8_47077540
		S4_7449601
		S1_68772990
		S2_11656286
		S3_61901457
		S2_74753056
		S6_32732003
		S7_65081305
		S8_60478492
		S2_60595324
		S2_9463477
		S9_15258389
		S3_59703267
		S4_9667057
		S1_79052107
		S7_3637415
		S2_71654434
		S2_61029330
		S4_53778210
		S4_52415845
		S8_1199391
		S1_74818662
		S2_3804962
		S3_72635949
		S6_50285828
		S8_6344371
		S3_71058357
		S4_8012646
		S2_63996163
		S1_55830723
		S9_1597145
		S6_47652171
		S4_7277283
		S4_1497830
		S2_50067230
		S3_71247679

Catogoery	Total	SNPs
		S3_19386059
		S6_46748444
		S2_64465317
		S7_54788902
		S8_55522701
		S1_14372563
		S8_2374779
		S1_79165409
		S6_47104007
		S1_52230757
		S8_4934915
		S8_1251946
		S8_61930945
		S4_3862008
		S8_1698997
		S6_493569
		S5_6623525
		S4_7781868
		S2_53068918
		S5_68080072
		S3_48311876
		S2_811796
		S4_46961010
		S9_53172709
		S7_7189921
		S2_59657172
		S3_66660319
		S4_6006457
		S5_3230241
		S5_66651128
		S1_10949934
		S8_45169114
		S4_66042973
		S9_5365186
		S8_1839695
		S3_50993707
		S9_52985956
		S7_6594046
		S7_63959951
		S4_45036075
		S6_31881926
		S4_68098075
		S6_54718400
		S6_50435868
		S1_12932195
		S9_57657914
		S6_47672827
		S2_13167900
		S9_42264495
		S2_11508818
		S1_7856674
		S3_69131623
		S1_66564564
		S2_56150280
		S2_73719521
		S2_2888141
		S7_6671224
		S3_20527107
		S4_4459943
		S9_52654146
		S2_76833039
		S2_61319728
		S5_61208201
		S4_66125731
		S2_8263818

Catogoery	Total	SNPs
		S4_40218796
		S9_51951356
		S7_27170367
		S4_789235
		S2_66293943
		S9_47049408
		S9_8569880
		S6_14158896
		S7_41316324
		S2_69604878
		S1_31441559
		S1_65353966
		S1_76020286
		S3_57750567
		S4_45036060
		S1_58096654
		S8_2067957
		S2_59198266
		S2_60888829
		S7_40022275
		S3_69744215
		S7_54307590
		S1_59699608
		S1_16203492
		S9_59378890
		S1_74788845
		S2_66605632
		S7_538947
		S5_62770249
		S6_41084115
		S1_64391935
		S3_62094772
		S6_49955693
		S9_5041236
		S2_59913881
		S7_58333568
		S5_12029402
		S1_18002166
		S1_74848736
		S1_1835175
		S2_41023684
		S5_68165112
		S6_31398668
		S3_70691294
		S3_6276850
		S8_3186440
		S3_55299567
		S9_7342729
		S6_46696269
		S4_2284037
		S4_47808761
		S9_57797471
		S9_7538580
		S3_70323660
		S8_59214639
		S4_2556983
		S3_70013744
		S5_15885735
		S3_5787681
		S2_58202073
		S3_714370
		S8_46923380
		S8_5103663
		S4_33504830
		S1_7020505

Catogoery	Total	SNPs
		S3_60718862
		S6_31579659
		S5_32151
		S2_5512684
		S3_70639687
		S4_10215783
		S8_56141615
		S9_52552184
		S6_53253980
		S2_62051206
		S1_1644309
		S8_2378243
		S1_78991715
		S3_4576574
		S2_60573526
		S9_50451610
		S4_5107108
		S7_1630101
		S1_77431837
		S9_59371925
		S4_56560692
		S1_18573249
		S4_1553927
		S8_1238603
		S1_77425692
		S9_54094853
		S1_66793390
		S5_12790515
		S4_50671827
		S4_1424081
		S4_4696509
		S4_56610938
		S7_54371465
		S8_14526028
		S8_4192197
		S5_62674264
		S3_64984144
		S9_58843515
		S9_214847
		S7_58718034
		S6_11328984
		S8_61547255
		S3_73290375
		S1_67745231
		S2_66967768
		S2_7876871
		S6_632992
		S8_5105300
		S2_56538556
		S8_5076028
		S2_64337658
		S7_64554805
		S1_9903451
		S2_12926294
		S9_7314111
		S7_60308754
		S7_52290928
		S6_15100893
		S5_62071952
		S1_61720672
		S9_35287639
		S4_4461253
		S9_52343145
		S2_45864760
		S9_57068854

Catogoery	Total	SNPs
		S2_72129120
		S2_6755759
		S9_41543595
		S1_63251915
		S1_19341841
		S5_62755996
		S6_6980388
		S1_20427270
		S6_47699482
		S3_69679841
		S1_24602894
		S9_57463336
		S3_66908741
		S8_53829093
		S1_62911515
		S5_1481107
		S1_72558649
		S9_2661231
		S1_2909513
		S9_4072342
		S7_9515015
		S8_61615356
		S1_27977620
		S3_53549462
		S2_73375995
		S1_79164986
		S3_1607538
		S2_41116489
		S5_61921364
		S8_5444817
		S1_63004826
		S4_51611492
		S2_56955041
		S7_39098972
		S4_3715850
		S2_5735164
		S7_409597
		S1_73890734
		S1_72772071
		S2_6027188
		S4_68098073
		S5_3865219
		S3_61566793
		S3_52457568
		S8_3671187
		S5_61161526
		S9_4346899
		S2_6426061
		S2_8867755
		S3_1964489
		S4_6504790
		S9_54939919
		S5_65909610
		S7_64061383
		S8_60933914
		S7_9425922
		S1_59709013
		S2_7709367
		S1_75746045
		S7_52729997
		S8_1238692
		S6_52484380
		S2_74682263
		S2_9484823
		S6_47606073

Catogoery	Total	SNPs
		S3_69262227
		S4_52778323
		S1_74032404
		S7_60741519
		S2_59734297
		S7_2922525
		S2_2758204
		S2_74965512
		S3_54139270
		S1_74327686
		S1_10082070
		S1_12730867
		S6_55689463
		S1_3351640
		S5_1796484
		S4_35594636
		S3_55732618
		S7_52880146
		S6_52713553
		S5_10195576
		S2_64543301
		S2_8409633
		S1_74489996
		S7_65081282
		S1_75662895
		S2_64350068
		S6_6109025
		S3_33671033
		S1_7499623
		S6_60774039
		S5_6808617
		S2_6139975
		S1_77424000
		S3_19996228
		S3_14342500
		S1_51841715
		S4_5866918
		S2_19376758
		S1_52802478
		S2_67765539
		S3_69587278
		S8_61563181
		S7_62354785
		S1_7390184
		S6_50932356
		S1_10689284
		S2_76812922
		S9_52824010
		S4_7889914
		S8_35260149
		S4_66103667
		S1_3546355
		S3_55948953
		S6_44752173
		S2_68827581
		S5_69847800
		S2_53439037
		S8_60548702
		S8_41501749
		S5_1444460
		S1_62080478
		S7_28567436
		S9_233777
		S5_58574740
		S1_72950013

Catogoery	Total	SNPs
		S4_8776939
		S1_74475248
		S3_73160925
		S7_59393504
		S8_58151666
		S3_61820400
		S2_11765751
		S5_7341795
		S2_62268880
		S8_1518512
		S5_1169528
		S1_20207349
		S2_76812917
		S4_1725066
		S9_7538587
		S8_53783859
		S1_57944966
		S8_60451667
		S2_16883651
		S5_37057049
		S9_49407178
		S2_5947079
		S9_47005941
		S9_8178052
		S9_58666217
		S1_58666937
		S9_1188115
		S3_62340077
		S1_11819553
		S1_72790614
		S1_64171965
		S8_1268567
		S4_8862674
		S8_56504618
		S6_3440970
		S1_2940379
		S9_3811751
		S4_52219836
		S8_4310382
		S7_10069262
		S2_49387021
		S2_66842279
		S1_78715996
		S3_20255105
		S1_11818072
		S5_69851981
		S9_2967317
		S1_16543099
		S1_6625912
		S2_58011446
		S8_57234675
		S8_49573365
		S1_76491669
		S2_49673136
		S8_57441521
		S3_59741331
		S4_2190300
		S9_50598275
		S5_62493121
		S1_60370249
		S8_53520158
		S1_52755340
		S4_51688898
		S3_64197927
		S7_52306289

Catogoery	Total	SNPs
		S2_67837145
		S7_11953657
		S8_59988339
		S5_66360813
		S9_54604707
		S1_61353202
		S9_55573838
		S3_61249047
		S4_63426489
		S8_57571479
		S6_41984853
		S7_9980841
		S2_6019792
		S8_57441911
		S1_50626375
		S6_58759528
		S4_56232947
		S1_26240530
		S5_616227
		S1_17106143
		S9_141044
		S7_6679582
		S5_1224721
		S2_68948540
		S2_63154476
		S2_56247970
		S6_41925154
		S4_66230017
		S4_13302502
		S1_71994937
		S1_20076264
		S5_67419542
		S8_46476058
		S2_8465303
		S1_53600998
		S6_48642153
		S5_65930881
		S6_6450331
		S1_52645299
		S9_52207267
		S1_59362345
		S4_56615484
		S8_31497040
		S2_63378530
		S4_41412962
		S1_63759405
		S3_62678363
		S4_580094
		S3_51465331
		S8_56542080
		S2_58604313
		S5_335676
		S4_56404754
		S6_2712195
		S2_12670173
		S7_1548361
		S6_18079708
		S8_56686249
		S6_1668338
		S2_66012623
		S3_3700983
		S8_1924407
		S3_6757312
		S4_25645552
		S4_34735132

Catogoery	Total	SNPs
		S4_42486850
		S8_5103679
		S6_5980645
		S1_59456536
		S7_10969493
		S7_54578835
		S7_63601250
		S2_73277286
		S2_8313688
		S2_6009188
		S1_1547257
		S2_12688000
		S9_53139314
		S6_44775834
		S3_54404359
		S5_1730230
		S3_3480278
		S3_14076775
		S6_54259842
		S7_54909107
		S9_3517529
		S6_35661850
		S8_55247663
		S8_1840333
		S7_64204040
		S8_1108540
		S4_7453624
		S1_58096650
		S3_57930202
		S1_4401944
		S7_40479931
		S3_5509796
		S4_54131968
		S1_54556031
		S4_950159
		S1_57470057
		S4_24560563
		S2_66276062
		S1_2914199
		S1_65250806
		S2_8451031
		S9_53172713
		S7_64827241
		S3_51904169
		S8_50357006
		S3_57925400
		S6_53848452
		S2_59576775
		S2_8450148
		S1_78818470
		S2_54216861
		S3_2067799
		S4_53381281
		S2_3432066
		S1_5865351
		S5_70008130
		S2_8263819
		S6_58758131
		S2_35639372
		S2_2688654
		S2_4257943
		S6_41768141
		S1_2901302
		S3_56320498
		S3_6089280

Catogoery	Total	SNPs
		S8_44849408
		S6_38162134
		S6_31113979
		S1_78620954
		S8_1137911
		S1_30401422
		S6_3377802
		S2_11408875
		S8_1691420
		S1_55237045
		S1_49218819
		S1_6439121
		S5_10490352
		S2_59045056
		S4_3728377
		S3_1869759
		S3_73607394
		S3_14076812
		S5_15885809
		S7_62980364
		S7_889916
		S7_9662527
		S1_74062606
		S3_53294386
		S2_61271030
		S1_78034831
		S6_52174170
		S8_4798395
		S1_3111471
		S4_38834217
		S1_64514455
		S1_67303970
		S4_3789931
		S1_12255235
		S9_7342521
		S1_74531599
		S8_61956186
		S4_62265479
		S9_41598411
		S8_57441521
		S2_60989152
		S2_13868825
		S6_2665922
		S9_58668999
		S6_39646060
		S3_56758960
		S2_46766398
		S4_10304304
		S3_73053305
		S3_65348743
		S2_66176000
		S1_50423331
		S2_5791855
		S1_16553183
		S5_67595963
		S3_71433050
		S1_20358330
		S1_10793332
		S3_68460867
		S7_1674588
		S5_68758198
		S2_34263685
		S1_19118158
		S1_16552947
		S3_57725050

Catogoery	Total	SNPs
		S6_54445803
		S2_59032528
		S8_58455798
		S2_75509937
		S7_8422784
		S1_27518592
		S9_38148379
		S4_3846718
		S8_61918672
		S1_66813862
		S2_65291559
		S1_75752514
		S3_68680002
		S1_60803184
		S1_12758271
		S1_66775172
		S2_18779393
		S1_56731892
		S3_73444872
		S5_71216694
		S7_57879510
		S8_1462792
		S8_54372914
		S8_51871022
		S1_60898587
		S3_57286183
		S1_11686407
		S8_5322991
		S6_28232346
		S3_58236244
		S2_14486711
		S3_60583219
		S9_5829133
		S5_1999526
		S8_48516654
		S2_63511600
		S3_6763720
		S1_9903436
		S5_62682852
		S3_620579
		S2_9671559
		S7_16068258
		S1_65256499
		S3_5561202
		S8_49573242
		S1_80424420
		S1_53479593
		S9_6694563
		S6_50919218
		S9_3025556
		S7_64807102
		S6_49691898
		S7_64098870
		S3_64841401
		S4_14976546
		S2_63081247
		S1_79965486
		S3_71419971
		S1_57882882
		S6_50647189
		S1_60005636
		S2_8370760
		S9_1646667
		S2_60136233
		S6_72144

Catogoery	Total	SNPs
		S1_17469476
		S2_28751282
		S9_52559323
		S9_54114066
		S8_4479870
		S2_59878110
		S4_2005774
		S1_15315421
		S1_8981815
		S1_7279190
		S1_8981560
		S6_35658927
		S1_8644842
		S6_50489403
		S6_16376578
		S9_1615518
		S8_61547472
		S6_54303425
		S6_2003404
		S2_10876295
		S8_56501449
		S2_4273050
		S3_20628603
		S5_66115704
		S3_61719161
		S2_66276037
		S7_62450372
		S2_1602557
		S9_50520624
		S3_73831916
		S7_58147435
		S6_48595446
		S5_61102870
		S3_74284920
		S7_7667152
		S1_8782341
		S2_65309973
		S4_2306076
		S3_64568827
		S6_50435855
		S9_2931139
		S2_61857000
		S8_9558461
		S3_4276131
		S3_4682321
		S1_32410059
		S7_14373151
		S5_65852079
		S7_62499103
		S5_5114695
		S1_61008560
		S4_11132983
		S1_14783761
		S8_3264580
		S1_10612387
		S2_60882835
		S6_54203494
		S1_71371597
		S1_72533095
		S1_72206057
		S1_16543100
		S2_62272869
		S2_75388429
		S7_55982518
		S6_37385043

Catogoery	Total	SNPs
		S8_4542283
		S3_60599099
		S1_66652234
		S4_42465602
		S6_51865298
		S1_3690023
		S3_57750640
		S7_930196
		S1_12797888
		S4_21545940
		S7_63431943
		S4_67680743
		S1_79700383
		S1_56195467
		S7_7590730
		S1_56295580
		S3_72504084
		S1_59824400
		S3_68836232
		S5_70791602
		S7_12203357
		S7_60706771
		S4_39258158
		S3_71419367
		S1_19932565
		S4_6756723
		S4_3924928
		S5_62155458
		S1_58840448
		S8_1997564
		S5_1641912
		S5_58573155
		S2_1306147
		S8_43503811
		S3_52260881
		S1_66026833
		S8_41592910
		S1_66898495
		S8_59192343
		S8_59928776
		S5_1795462
		S1_3455494
		S4_4321306
		S4_10307845
		S9_50757960
		S1_6357637
		S6_56756473
		S4_5042316
		S2_18996253
		S1_66339225
		S9_50874682
		S3_55885926
		S1_79154732
		S3_55278689
		S6_53273377
		S1_3976598
		S5_6008153
		S2_65235168
		S2_12675169
		S6_55277725
		S2_69829406
		S8_1753898
		S4_62988569
		S2_12688027
		S8_2271776

Catogoery	Total	SNPs
		S7_58665557
		S5_12857845
		S1_52769239
		S2_4727236
		S2_3294654
		S7_65179560
		S6_52621592
		S4_46961014
		S2_5489040
		S3_66023882
		S2_71595649
		S4_1064467
		S1_70246986
		S4_1121023
		S1_71730176
		S2_36482136
		S6_52777863
		S8_3691823
		S2_71699777
		S1_58913453
		S1_73467735
		S7_64554824
		S1_71946412
		S4_51151942
		S8_56867322
		S6_8550020
		S6_26278689
		S8_49763619
		S3_71689728
		S1_26239948
		S6_45146402
		S2_69602244
		S3_40603403
		S3_71236581
		S5_69880199
		S3_72851929
		S5_61127856
		S6_51870339
		S4_37491547
		S4_6504813
		S1_74360064
		S2_65407929
		S1_898865
		S9_1075111
		S5_2601116
		S3_67154235
		S3_55300985
		S4_1008650
		S2_66189807
		S1_2909521
		S5_43427619
		S1_60816161
		S1_52412950
		S1_59699989
		S9_6863007
		S1_78818475
		S4_836915
		S8_59990841
		S1_13325758
		S8_51865735
		S2_69018998
		S5_1121584
		S1_64392653
		S6_50684930
		S3_69734217

Catogoery	Total	SNPs
		S1_77956897
		S8_53641822
		S7_14376951
		S1_67713947
		S3_5106745
		S2_9815995
		S8_1172573
		S9_6854752
		S1_68248799
		S9_27627
		S9_6391698
		S5_65640096
		S3_59706466
		S4_52459678
		S5_69794968
		S2_60550166
		S8_60548752
		S2_467608
		S6_45510274
		S9_54080154
		S2_69076922
		S3_58281910
		S1_61737841
		S9_9649562
		S6_41265271
		S6_53466872
		S4_67973169
		S8_61636551
		S7_55750862
		S2_46793058
		S1_58165618
		S1_65740013
		S5_2074958
		S2_61148237
		S5_66487562
		S1_20349811
		S3_52431132
		S1_68798194
		S1_79844267
		S1_74471940
		S7_57379678
		S7_7008352
		S2_6239419
		S1_72605776
		S5_9537222
		S6_47785501
		S1_72724132
		S7_8924573
		S9_4238666
		S3_46856330
		S5_1625481
		S6_47539486
		S2_64342532
		S7_63065197
		S4_20710728
		S2_47634493
		S3_72310648
		S4_20035139
		S3_52168863
		S2_2237673
		S1_73871821
		S1_23892412
		S1_26252407
		S1_16641197
		S6_50838163

Catogoery	Total	SNPs
		S3_60800075
		S9_42032517
		S6_30087733
		S7_54525596
		S5_5812329
		S1_17426904
		S7_7749144
		S6_12448067
		S3_73925059
		S2_8235880
		S5_6894089
		S1_65245155
		S3_70765462
		S9_2619799
		S2_61492071
		S1_65145272
		S5_10382333
		S4_2893842
		S3_42058226
		S9_57745738
		S8_18743019
		S2_12858148
		S7_500830
		S3_69939580
		S3_64873877
		S3_61017599
		S2_59226709
		S2_802884
		S3_59670673
		S1_78565919
		S6_47539492
		S3_73845317
		S6_53954708
		S3_54224770
		S5_11169647
		S7_8824239
		S5_61338552
		S3_65912409
		S9_100159
		S7_1522084
		S1_79192234
		S2_58868341
		S5_67374292
		S4_56329791
		S2_6526531
		S7_8905186
		S4_450134
		S8_54537659
		S3_14045831
		S2_57493050
		S3_16632215
		S2_3998377
		S2_6099958
		S8_58744259
		S6_49403571
		S2_60550876
		S2_6687222
		S7_39887237
		S3_73239503
		S2_51560650
		S2_76943609
		S4_11180355
		S1_7961500
		S9_1260634
		S2_58604023

Catogoery	Total	SNPs
		S3_4584866
		S2_8879098
		S2_56440835
		S3_19522167
		S2_64072292
		S1_3611885
		S7_10069310
		S1_79507176
		S4_6414128
		S3_56681277
		S2_69844664
		S1_47588902
		S9_1106714
		S3_2360412
		S5_11706288
		S4_1121016
		S7_64968886
		S7_10074718
		S6_1586944
		S2_69148759
		S1_53773916
		S6_54641320
		S4_4816243
		S1_72489081
		S3_63300808
		S9_49345776
		S1_58096658
		S9_53171066
		S6_53866236
		S3_58305515
		S6_53848304
		S1_78708717
		S1_59471503
		S2_62493948
		S1_59457628
		S5_3818318
		S5_69893249
		S3_1871283
		S6_2155332
		S6_54737817
		S8_4401961
		S4_24571906
		S1_72052785
		S9_58600960
		S1_78558657
		S1_22035651
		S1_66101357
		S4_5579986
		S2_59247355
		S2_12746306
		S1_59976424
		S2_15328287
		S6_53664202
		S1_30244645
		S3_69282697
		S1_22272039
		S3_72308279
		S8_62112218
		S1_74788775
		S6_49485074
		S7_38848398
		S1_72593133
		S7_54573367
		S5_55200103
		S8_57571706

Catogoery	Total	SNPs
		S6_47043883
		S6_2155349
		S2_5523159
		S3_64877680
		S2_10813857
		S9_49965065
		S4_63417788
		S7_815959
		S4_46116735
		S2_10527929
		S9_54901516
		S7_58562730
		S4_53344052
		S1_73857351
		S9_51524286
		S5_12536144
		S2_59737799
		S6_45640374
		S1_19407750
		S4_2650388
		S9_74376
		S7_17083433
		S4_3627104
		S8_59746774
		S1_2328747
		S3_68664319
		S8_58035351
		S7_62396478
		S1_71371602
		S3_72827126
		S4_41650231
		S4_53281726
		S1_55857064
		S8_3380604
		S7_62925022
		S4_2851229
		S7_41122790
		S2_7004469
		S5_1526386
		S4_38716962
		S6_18421881
		S4_7233591
		S2_40344678
		S3_59599975
		S7_59366675
		S3_73887811
		S3_67541352
		S5_11328788
		S3_70519672
		S1_79156367
		S9_1073047
		S8_49933660
		S3_57258119
		S1_77114886
		S7_57230524
		S8_14164269
		S1_22272034
		S7_60578181
		#N/A
		S2_63477010
		S7_54854118
		S3_13238332
		S6_31398601
		S9_2931118
		S2_68948515

Catogoery	Total	SNPs
		S5_61217764
		S1_9140046
		S5_1160897
		S5_50562093
		S8_1341807
		S3_61852091
		S1_67562157
		S2_76833127
		S7_18347321
		S9_57611540
		S4_4827223
		S6_55684264
		S9_59195467
		S3_56758955
		S1_79859822
		S1_17276944
		S5_16084035
		S1_61353197
		S2_44672355
		S6_50717448
		S7_59307290
		S8_51183174
		S1_15779111
		S1_78288367
		S2_4396173
		S5_9422827
		S2_71652220
		S5_13393867
		S2_3405870
		S7_53056335
		S9_12191711
		S7_54411321
		S1_51575414
		S2_63669877
		S8_53860844
		S8_2321821
		S1_56201332
		S2_11785051
		S2_55048647
		S8_61960838
		S8_4310381
		S3_58246377
		S9_53925466
		S1_72245187
		S5_11195296
		S2_2831312
		S7_63028990
		S8_60452264
		S8_3510650
		S7_6373342
		S2_77007657
		S9_7816553
		S5_62379333
		S4_26389667
		S3_56159781
		S5_5812211
		S3_54705294
		S7_2771733
		S1_15897161
		S7_3694712
		S7_766144
		S5_11064453
		S3_57908874
		S2_59247247
		S4_49816083

Catogoery	Total	SNPs
		S1_9485124
		S5_10715093
		S1_51753653
		S3_53300152
		S2_58565145
		S4_10670186
		S7_62275425
		S3_914002
		S2_61706094
		S8_60087254
		S1_65383259
		S2_70927307
		S7_57067166
		S1_11198818
		S5_4702984
		S3_59636557
		S8_54655198
		S5_3397182
		S3_69734240
		S6_49476633
		S1_68854579
		S8_39928479
		S8_60362312
		S9_1203748
		S7_54947513
		S9_9793367
		S9_9282095
		S1_65251204
		S2_69020524
		S1_17902991
		S5_66441884
		S1_59383289
		S2_10894012
		S1_72242897
		S3_74068888
		S1_67683463
		S7_7795035
		S1_67714508
		S2_60845384
		S1_59338088
		S3_61294456
		S1_57419983
		S8_14700652
		S2_59734367
		S1_68781638
		S2_63938954
		S2_10330243
		S1_71749944
		S5_12833237
		S9_40978329
		S6_54651659
		S3_73146203
		S2_19732916
		S6_41273715
		S3_67518180
		S1_8881257
		S1_18880715
		S1_2989626
		S6_50356634
		S8_43909258
		S6_53151614
		S5_3384145
		S1_8441723
		S6_58881502
		S3_62067129

Catogoery	Total	SNPs
		S8_7197904
		S4_67083059
		S4_66158408
		S1_25582488
		S3_1905145
		S7_9515489
		S9_51417450
		S3_70860939
		S9_53720743
		S1_57873668
		S9_52932648
		S6_46577246
		S2_8430569
		S7_64825483
		S7_1548371
		S5_11199682
		S4_50974937
		S9_42074266
		S3_64318104
		S2_64072256
		S3_72108998
		S5_66405186
		S3_64452178
		S3_53551102
		S3_73563491
		S2_66175163
		S1_79036320
		S9_6579442
		S2_242190
		S2_66684351
		S9_51068873
		S4_66239810
		S2_61695480
		S2_6684686
		S6_40434367
		S3_73700078
		S6_2003403
		S3_34504581
		S5_68889557
		S7_40295160
		S4_39801955
		S6_56567268
		S2_6845044
		S1_61730039
		S7_59980985
		S1_72848013
		S2_61588992
		S7_887901
		S1_5700614
		S8_60254516
		S1_18916951
		S1_8138434
		S3_73325615
		S6_45640178
		S1_11800899
		S8_56411725
		S2_14805191
		S1_7313245
		S4_8528737
		S1_80001981
		S6_47343553
		S2_59576737
		S1_79401513
		S6_53622954
		S2_61594581

Catogoery	Total	SNPs
		S3_68170360
		S2_19295072
		S2_59197594
		S9_42731948
		S1_16552922
		S7_57573969
		S2_23846342
		S3_3757337
		S1_51003915
		S4_5534883
		S3_6081428
		S3_16632191
		S5_42505614
		S2_73694022
		S2_3883280
		S9_58170599
		S5_62922677
		S3_38419884
		S3_18753052
		S4_24584922
		S6_41350743
		S7_3930607
		S2_9866078
		S9_56968843
		S2_12734162
		S4_15902693
		S7_14448563
		S2_60086689
		S5_2693011
		S8_60849580
		S1_54392974
		S1_71967902
		S3_70599124
		S9_50442995
		S3_5627407
		S9_993059
		S5_14746358
		S2_6426108
		S7_64827187
		S3_56681994
		S9_8900776
		S6_51114432
		S3_61384361
		S1_1346273
		S4_67970776
		S1_12932384
		S1_63071897
		S2_7659353
		S2_60166319
		S9_52078646
		S7_56038957
		S3_71652590
		S7_53150022
		S3_4450674
		S6_2155308
		S6_53240804
		S6_49478723
		S2_63341195
		S8_58455797
		S1_49780390
		S7_64071095
		S1_6512010
		S6_47882287
		S1_80001982
		S5_1524869

Catogoery	Total	SNPs
		S3_63918249
		S6_60551583
		S7_55982431
		S1_59114718
		S8_41733830
		S2_53473934
		S1_53912594
		S4_49020487
		S2_6230175
		S1_51003644
		S6_16433312
		S3_57750613
		S3_13358801
		S8_62014446
		S6_40512582
		S1_57470112
		S3_71236540
		S4_3734957
		S8_1250269
		S1_64344982
		S3_57215994
		S7_41122776
		S3_72514879
		S7_8824290
		S7_64061478
		S1_29777712
		S3_60583196
		S6_27544291
		S7_1789978
		S8_53262103
		S2_62729834
		S8_56264326
		S3_54181139
		S2_12686995
		S4_15873814
		S4_52058869
		S1_19376071
		S1_3350850
		S9_5167971
		S4_3811340
		S3_64175744
		S8_30845529
		S1_63986676
		S8_4730319
		S4_61204129
		S3_71866553
		S2_73833803
		S4_38135069
		S6_46552008
		S4_56615481
		S1_22272042
		S6_50033377
		S6_25874717
		S3_52637792
		S2_70910769
		S1_59540885
		S2_59840089
		S4_55505265
		S4_6756610
		#N/A
		S1_17276957
		S1_58661410
		S2_67195395
		S7_5962647
		S5_10818597

Catogoery	Total	SNPs
		S3_14657393
		S4_67124108
		S1_71731910
		S8_49213291
		S3_69480184
		S1_70246991
		S2_56916042
		S6_28003012
		S3_70228324
		S4_41454672
		S2_15451049
		S9_57753567
		S3_68296162
		S1_19113331
		S2_53885680
		S6_1390457
		S2_49470483
		S9_5829125
		S9_5861371
		S1_72530875
		S1_66128657
		S2_60448359
		S3_54259972
		S7_1188661
		S5_11706324
		S9_58114237
		S2_18758071
		S1_63509659
		S3_70480226
		S7_943341
		S3_73733724
		S2_49424303
		S6_53866166
		S3_5640148
		S4_5902114
		S6_50919217
		S1_63075713
		S3_73046029
		S3_60996655
		S2_73699056
		S8_58455667
		S6_53605284
		S2_76496256
		S2_8383750
		S7_8018598
		S4_1338965
		S1_31441486
		S5_1446778
		S6_50487411
		S9_41598780
		S1_61680014
		S9_1594342
		S1_9068985
		S9_2781767
		S7_61408591
		S4_12948139
		S2_72265468
		S1_11198832
		S9_57784998
		S1_67332442
		S6_37400419
		S2_2834836
		S9_1238031
		S4_45714400
		S8_60087258

Catogoery	Total	SNPs
		S1_4153695
		S2_42024019
		S2_30234072
		S1_58913384
		S1_16387761
		S1_7505871
		S7_62458479
		S7_64774396
		S8_15754271
		S7_7936842
		S2_10525633
		S3_5626067
		S6_5328966
		S7_5919969
		S1_8916315
		S1_71946415
		S2_57728127
		S8_47521921
		S8_17999743
		S1_14285577
		S5_62628769
		S4_51774110
		S1_51888248
		S1_66528407
		S7_9889297
		S2_61029319
		S4_1499221
		S9_58293186
		S1_23907600
		S5_13409614
		S1_60835042
		S3_18235498
		S3_62407554
		S2_76435612
		S8_5320761
		S2_77443947
		S1_55512378
		S4_46117580
		S4_14183710
		S7_60488158
		S4_6054017
		S2_10485898
		S8_56293834
		S6_844919
		S1_7069607
		S2_56955025
		S5_10690099
		S3_68114833
		S2_2384480
		S2_71652247
		S4_52778178
		S5_13132525
		S2_66035565
		S9_55626937
		#N/A
		S8_60695662
		S3_53551113
		S8_19258158
		S6_382697
		S3_51972038
		S6_51851673
		S1_52755841
		S5_8848245
		S5_63235235
		S8_49628791

Catogoery	Total	SNPs
		S2_56230744
		S1_68963716
		S1_50852716
		S2_13987552
		S1_72186050
		S8_60599856
		S1_57740556
		S4_1338865
		S3_69068844
		S1_18535347
		S5_11314287
		S1_59644616
		S9_8182936
		S4_53889726
		S4_6504795
		S3_51838810
		S4_3833721
		S8_44849410
		S2_66175937
		S1_7619685
		S5_62503705
		S2_5632708
		S2_64088628
		S1_13832008
		S8_2309345
		S6_42017330
		S4_3811434
		S5_66068691
		S8_37595626
		S6_53848445
		S1_72061557
		S9_52582256
		S2_65228854
		S3_69633985
		S9_52880166
		S7_513891
		S3_74077605
		S3_16063294
		S2_64287878
		S4_1509394
		S5_67567203
		S1_80487238
		S5_61579928
		S1_77245029
		S2_75388436
		#N/A
		S3_570087
		S5_11779494
		S4_62264742
		S1_72949907
		S8_58488097
		S8_3369856
		S7_54411313
		S3_5944886
		S6_45540275
		S1_57110190
		S2_68946002
		S2_3390562
		S2_47634498
		S2_8111631
		S4_56679605
		S5_66607821
		S2_12607415
		S2_59199492
		S4_1110385

Catogoery	Total	SNPs
		S7_930813
		S1_72938614
		S1_18314231
		S1_51525808
		S9_54583690
		S2_65302363
		S5_7006744
		S6_15129526
		S7_62980401
		S8_49063908
		S1_52332804
		S8_61921709
		S4_3928114
		S7_64750999
		S1_74962140
		S1_24777317
		S1_8495622
		S6_3463573
		S1_2960173
		S2_61097980
		S4_3998847
		S1_12306189
		S5_69889730
		S8_53877328
		S7_55982577
		S4_53367484
		S1_76636740
		S1_19480868
		S2_56332752
		S2_10525712
		S5_69854897
		S2_64072284
		S7_2920284
		S3_74147920
		S6_8919203
		S1_2744771
		S7_8105768
		S4_62942542
		S7_1838966
		S3_40091659
		S2_6207071
		S3_61932002
		S3_74112321
		S8_3579334
		S2_1573591
		S3_6722739
		S1_10308541
		#N/A
		S6_36174912
		S4_42264592
		S1_3455496
		S9_59408381
		S2_65189301
		S3_51838770
		S8_47529562
		S4_67425450
		S8_4542288
		S7_63811338
		S7_56589392
		S1_10659544
		S3_6276892
		S1_46558451
		S1_2313837
		S7_9729311
		S4_51427629

Catogoery	Total	SNPs
		S9_2566585
		S8_56781138
		S3_68205645
		S5_11060012
		S3_52911837
		S3_68983671
		S8_1991807
		S6_29605537
		S2_75952184
		S2_64467126
		S2_60008765
		S8_5322966
		S1_24472020
		S1_20266390
		S3_70239280
		S7_62716947
		S4_52450527
		S9_2619773
		S5_3626695
		S2_76784102
		S8_3103591
		S2_60347978
		S7_51738003
		S8_54858069
		S9_42812117
		S5_8201145
		S5_67364866
		S8_5320755
		S5_36118895
		S1_62096445
		S6_27894408
		S5_4355102
		S3_73290458
		S5_38189514
		S8_9362665
		S1_65740549
		S8_49063914
		S2_56925061
		S1_30661969
		S5_61208203
		S5_69882454
		S9_58916305
		S1_15082356
		S5_11514082
		S1_72859888
		S5_65816968
		S1_78755143
		S1_79775165
		S1_68247465
		S5_36176214
		S5_65842217
		S3_15945840
		S1_80030410
		S4_2893832
		S6_18030211
		S8_58489541
		S1_59131476
		S1_72010269
		S2_6579268
		S8_34190221
		S7_7190627
		S1_21693809
		S4_52778236
		S1_15031609
		S3_53292782

Catogoery	Total	SNPs
		S3_67023424
		S7_6432632
		S8_58147348
		S1_66651016
		S1_57942921
		S4_6500621
		S2_61927641
		S2_12744934
		S8_61541274
		S2_18779552
		S2_59972371
		S8_50612384
		S1_16399202
		S3_62858488
		S2_47529028
		S2_68829261
		S4_56299947
		S3_55204440
		S6_1839013
		S8_59749205
		S7_62276698
		S1_72558803
		S1_73060851
		S1_78034859
		S2_7878108
		S2_57607894
		S3_5659686
		S4_66100408
		S7_17216587
		S9_50732842
		S5_7006744
		S1_51455889
		S6_53558258
		S8_55498852
		S4_25646774
		S2_9638391
		S2_4115213
		S7_2920277
		S3_71673905
		S1_12305221
		S1_75531352
		S2_13879046
		S1_7167203
		S5_3376379
		S2_12734125
		S1_7959919
		S7_12055070
		S2_1009112
		S2_64287854
		S2_65854326
		S2_72283209
		S5_4242507
		S1_7746883
		S1_9813897
		S2_59741731
		S1_77452345
		S6_47935167
		S2_11248060
		S5_62412834
		S3_57894798
		S1_21735691
		S4_58463694
		S1_27123142
		S5_4905498
		S1_79369154

Catogoery	Total	SNPs
		S3_66570473
		S7_54573276
		S2_76768797
		S5_62405549
		S1_17593459
		S5_8003220
		S3_69856686
		S3_57962137
		S5_62493126
		S9_1204243
		S8_60934202
		S9_58178266
		S6_39672553
		S1_11197481
		S2_9497075
		S6_53240800
		S1_16641334
		S1_61730041
		S8_49845530
		S2_37612410
		S2_73277909
		S1_46266986
		S2_14880562
		S3_70243058
		S2_75364740
		S9_7137419
		S7_646995
		S7_58534229
		S6_46764836
		#N/A
		S3_67154474
		S2_2021031
		S6_50266442
		S2_12733925
		S7_63118110
		S6_46801672
		S4_66240137
		S8_422821
		S5_6101722
		S1_77974658
		S8_60259425
		S6_1587011
		S3_67717984
		S8_557329
		S6_50914738
		S7_64669779
		S3_70598764
		S2_12927579
		S9_4249152
		S5_5172412
		S5_8211645
		S1_65736316
		S2_59755659
		S3_72104761
		S2_30234061
		S2_52371689
		S1_78126258
		S6_25538509
		S8_5160789
		S8_3173719
		S1_60720826
		S7_58195099
		S2_49264454
		S7_8824271
		S5_14747250

Catogoery	Total	SNPs
		S3_57930205
		S4_47808746
		S2_73675562
		S4_11474340
		S1_22627577
		S6_2003409
		S2_73888007
		S3_66788745
		S3_56957813
		S5_6868782
		S7_52170358
		S2_24396604
		S2_57518935
		S3_73231500
		S3_66408432
		S2_73858862
		S9_52077277
		S3_73791906
		S6_53317061
		S2_61550902
		S9_44566091
		S9_58967708
		S5_63419094
		S6_53396974
		S2_13066791
		S6_41878476
		S1_57676315
		S1_18923712
		S2_10926634
		S7_15560287
		S5_5366265
		S1_71728517
		S3_73887740
		S5_5114469
		S1_2989614
		S2_56955026
		S2_720693
		S4_5947093
		S7_63639772
		S2_66010335
		S4_62878059
		S1_16387713
		S3_73086679
		S7_62994928
		S3_55005191
		S3_54478382
		S1_2587276
		S7_62721732
		S1_16647165
		S2_6036459
		S9_3167364
		S8_44058554
		S2_75471801
		S3_67029957
		S8_61960539
		S3_56547004
		S6_51851707
		S7_64603325
		S4_41795064
		S6_53582925
		S9_8722177
		S3_59708053
		S4_24547814
		S6_27320004
		S5_9778787

Catogoery	Total	SNPs
		S9_52824096
		S9_48996400
		S1_80740375
		S1_72873848
		S3_73506878
		S2_29787192
		S2_6396288
		S2_55048591
		S4_66115249
		S2_74926631
		S3_71064417
		S1_58946598
		S1_10734489
		S1_55151233
		S4_11225623
		S1_59699619
		S4_66157028
		S9_48449
		S3_55904134
		S1_55873464
		S5_4754135
		S5_68867178
		S3_67843444
		S6_3903714
		S2_13459040
		S8_1993575
		S7_3637777
		S3_53583423
		S4_56600810
		S2_75369220
		S1_17931425
		S6_44606835
		S9_52837691
		S2_60965831
		S2_12351390
		S1_65742376
		S3_73934906
		S9_50653913
		S4_1328165
		S7_59024835
		S1_61559382
		S6_3463592
		S5_6868733
		S6_2665890
		S7_64061379
		S9_1260271
		S2_59734063
		S1_11197483
		S6_51919006
		S2_9370630
		S1_28678205
		S2_73738622
		S5_6809825
		S5_3388795
		S1_77200433
		S8_2082249
		S1_2909420
		S4_66601970
		S1_13324712
		S2_67307584
		S3_3904008
		S7_5795049
		S3_62678360
		S7_63317554
		S7_6595501

Catogoery	Total	SNPs
		S1_73811468
		S2_17954289
		S4_45343269
		S1_59558486
		S2_45978414
		S8_1239093
		S4_52473571
		S2_72317061
		S6_58336179
		S5_11931276
		S5_59317245
		S1_51841722
		S1_76020372
		S4_61260534
		S8_4310393
		S9_1594508
		S7_62611629
		S9_3620844
		S5_9550443
		S1_79165408
		S4_7474492
		S1_60601167
		S2_29869285
		S7_7940216
		S7_62476321
		S7_54775612
		S9_8179101
		S1_9413545
		S1_13350106
		S2_55524387
		S9_5252483
		S3_52431155
		S2_45978384
		S1_59685836
		S2_58201004
		S3_69365336
		S6_1668313
		S4_40898871
		S2_63693104
		S6_31095730
		S3_69598986
		S5_1248479
		S1_56909302
		S9_57542607
		S2_72315099
		S1_72534355
		S4_7548660
		S1_31205579
		S1_78743083
		S9_52079055
		S1_64159575
		S2_75804320
		S3_16421665
		S3_58846076
		S6_29516317
		S5_61936822
		S1_58025494
		S1_78929757
		S4_54040206
		S2_61586261
		S1_9364825
		S3_70013939
		S9_4037880
		S9_51890220
		S2_301701

Catogoery	Total	SNPs
		S1_79195146
		S1_19244109
		S9_1707496
		S3_53588190
		S9_54080250
		S1_8268987
		S9_50296452
		S3_59601930
		S8_3729359
		S3_56674505
		S9_2934170
		S6_53168222
		S5_8735164
		S1_77830606
		S1_10082072
		S4_836921
		S1_73581053
		S2_61969443
		S5_67362944
		S5_40616576
		S6_48241280
		S2_52366160
		S7_58482338
		S7_64473252
		S9_4743785
		S3_72322426
		S1_29777710
		S1_64437159
		S7_15438158
		S2_47198568
		S4_42859962
		S9_53867592
		S1_66109134
		S4_54095461
		S1_25897340
		S3_4276127
		S1_53473463
		S2_75991630
		S8_44424413
		S4_14183606
		S8_56721709
		S6_14855511
		S9_6792608
		S3_15945842
		S1_77814863
		S7_63639760
		S8_60450789
		S9_51543251
		S4_50802127
		S9_1115497
		S3_67871222
		S8_3457017
		S2_65352917
		S3_60936291
		S2_55384042
		S2_63821492
		S2_75894068
		S3_66417384
		S2_60563762
		S5_34571514
		S2_61420554
		S5_62728099
		S5_11781310
		S2_6271598
		S1_6040051

Catogoery	Total	SNPs
		S8_55930260
		S6_50487462
		S3_63545218
		S1_69096295
		S2_61444554
		S9_1065962
		S9_233810
		S1_74791728
		S2_10895625
		S8_33506696
		S5_450453
		S3_3902086
		S1_12965848
		S3_5645001
		S3_70640369
		S3_68840917
		S5_5812207
		S9_8924399
		S7_63156249
		S5_69893233
		S6_28742840
		S5_15247798
		S8_38812576
		S8_61641307
		S7_61414427
		S5_2829043
		S2_59948107
		S7_39233755
		S7_8640613
		S2_1052088
		S6_54390546
		S4_54867151
		S2_6542873
		S5_65774589
		S3_59270290
		S3_61458654
		S5_58355603
		S9_55626861
		S9_8512843
		S1_17585598
		S5_67495279
		S7_54775507
		S5_62355339
		S1_22035511
		S1_12758662
		S1_15041510
		S6_53168222
		S1_3051479
		S9_43903460
		S5_3621144
		S1_71946550
		S2_75589275
		S5_62530356
		S1_30465284
		S9_4314082
		S1_9153558
		S2_75658171
		S3_74078915
		S1_62732128
		S1_60353141
		S9_58601404
		S2_5948253
		S1_1547289
		S1_17125050
		S2_52740443

Catogoery	Total	SNPs
		S2_14775379
		S7_63794782
		S3_48311840
		S4_53238382
		S2_7708895
		S9_1695817
		S3_4816968
		S3_5521238
		S7_9525015
		S5_70957316
		S6_53106149
		S3_5944883
		S2_73335517
		S4_8758791
		S8_55614117
		S1_7313277
		S6_37385072
		S3_62067133
		S6_47065480
		S3_1969735
		S7_985874
		S5_8111481
		S2_75410032
		S2_59240142
		S1_72631506
		S2_53473320
		S3_74311135
		S2_16852138
		S8_1563128
		S5_14150707
		S7_9515435
		S1_13384303
		S3_3480351
		S8_5140437
		S4_44730238
		S9_50395279
		S6_17497707
		S9_5232673
		S4_48336940
		S1_9062575
		S3_14047185
		S9_47049868
		S3_31534391
		S2_63584033
		S9_50082999
		S8_39471897
		S6_53227234
		S3_56682906
		S8_54112604
		S8_40583335
		S3_59369084
		S7_62863091
		S1_7528644
		S7_59053640
		S9_5365184
		S6_51803456
		S5_3388738
		S6_49276391
		S1_10949886
		S4_4943961
		S6_1994542
		S8_48333991
		S1_10100857
		S9_5861345
		S1_3156917

Catogoery	Total	SNPs
		S8_56781020
		S2_12670126
		S3_72663258
		S8_61985644
		S2_9370717
		S6_47937456
		S1_31205588
		S2_40706120
		S6_40234814
		S9_54155511
		S2_60923684
		S6_47959714
		S6_47452039
		S5_61619404
		S2_60965626
		S1_16740490
		S1_65245227
		S5_16083957
		S1_77156204
		S6_45545079
		S1_77100621
		S4_21766043
		S6_47672893
		S2_6271148
		S1_51860514
		S2_59045053
		S2_12575090
		S1_63025375
		S3_64927243
		S3_72216862
		S4_9075831
		S1_73737334
		S1_74349802
		S4_1648085
		S2_75753467
		S1_76289052
		S6_41921475
		S5_11584642
		S2_6020529
		S3_58320546
		S2_10527144
		S2_61654770
		S3_68703327
		S1_18844958
		S8_1979362
		S2_6045456
		S7_64515396
		S6_44561295
		S4_2581827
		S1_79192235
		S3_72712962
		S1_32487013
		S9_10186146
		S1_73644714
		S4_5866920
		S3_15611318
		S3_69420892
		S4_47223315
		S1_6777454
		S3_72790078
		S2_66606181
		S1_59759825
		S8_4402301
		S7_62611891
		S3_69485308

Catogoery	Total	SNPs
		S6_53240817
		S1_72691373
		S5_65842219
		S2_48405116
		S1_18198945
		S2_58605696
		S3_62982619
		S5_24065931
		S4_51708722
		S7_1163209
		S1_63711706
		S5_58574522
		S5_3320545
		S3_69365389
		S8_32509676
		S5_70773493
		S4_51774122
		S2_42428729
		S6_52777719
		S6_2892438
		S2_52194343
		S4_52949062
		S7_40134314
		S4_2292610
		S6_1067418
		S1_19695252
		S2_47286424
		S6_32048804
		S8_50941253
		S1_21046819
		S4_15390402
		S7_7000622
		S7_62935590
		S3_61913511
		S2_67282450
		S1_79701404
		S2_6687147
		S4_51878031
		S2_18573452
		S1_6328129
		S2_61654279
		S7_58333566
		S1_72898846
		S8_60695650
		S6_17785753
		S2_69809319
		S9_1742383
		S5_65464729
		S2_72626252
		S5_1121584
		S2_41882481
		S7_60972647
		S6_51477214
		S1_80594326
		S8_57227150
		S3_56085489
		S2_50205553
		S2_67765537
		S4_46351759
		S1_64884880
		S8_60319357
		S8_56542105
		S2_56440766
		S8_5379894
		S2_63220915

Catogoery	Total	SNPs
		S3_6295436
		S2_74915763
		S2_77014523
		S1_79775767
		S7_62475177
		S7_5171922
		S5_59325591
		S5_50601106
		S7_2791382
		S2_8111628
		S3_73046081
		S6_44605496
		S1_74876555
		S1_78396281
		S2_3808830
		S3_59708025
		S3_65332754
		S2_77504388
		S6_1048128
		S5_59325685
		S9_49551667
		S1_77666329
		S7_58674046
		S1_16732988
		S7_62851076
		S7_703225
		S8_59750947
		S1_55504275
		S4_6221238
		S2_56332752
		S1_8975281
		S2_42945120
		S3_72878969
		S3_70825085
		S7_10273344
		S3_14089664
		S1_72454961
		S3_52050038
		S1_48286785
		S6_41603582
		S2_62483858
		S6_1316379
		S2_4749674
		S3_65428273
		S3_4851301
		S1_78715872
		S4_50273646
		S5_62646205
		S4_54195676
		S2_49887093
		S8_61951470
		S2_8383698
		S1_59313273
		S9_53406643
		S1_67593868
		S4_37597167
		S7_4832457
		S9_4382755
		S8_60362309
		S7_5588488
		S2_4555010
		S8_14857384
		S8_60160151
		S4_68098064
		S8_49212570

Catogoery	Total	SNPs
		S2_63426482
		S5_61127991
		S3_4315341
		S6_47955706
		S1_64298098
		S1_55512376
		S1_18880752
		S1_70466570
		S9_3540384
		S5_67938439
		S7_9515488
		S5_1729103
		S3_70901590
		S3_69681303
		S5_11792485
		S1_66362738
		S6_53836196
		S6_31172110
		S4_50569113
		S4_1372877
		S1_72373910
		S9_1111016
		S1_72605904
		S7_9418027
		S5_62383869
		S8_54104040
		S5_1160899
		S2_6178221
		S5_2693168
		S9_54120707
		S4_68010987
		S6_49955996
		S1_17700486
		S1_22272030
		S7_6595682
		S4_46872745
		S6_53004717
		S2_25439144
		S1_7769764
		S5_61615958
		S3_70528514
		S3_3480335
		S8_45536794
		S1_63259971
		S5_63463719
		S7_57835070
		S1_56731423
		S1_10715659
		S4_54204325
		S1_75624818
		S3_6754879
		S8_58069597
		S4_24756316
		S4_27834913
		S2_52396320
		S1_77914254
		S5_1971654
		S3_3517493
		S4_24560561
		S6_54381374
		S8_18048867
		S3_52852585
		S8_4993998
		S2_17634600
		S7_62909716

Catogoery	Total	SNPs
		S4_1570079
		S7_9515065
		S3_5618882
		S9_58728429
		S2_34961784
		S2_19726974
		S2_1333302
		S1_18912541
		S2_11851280
		S1_78319566
		S4_26064448
		S6_52537283
		S6_58812611
		S1_2053976
		S3_47202087
		S7_1163205
		S2_69027260
		S4_5107568
		S2_57728898
		S9_53867590
		S3_4682286
		S7_6679582
		S8_3230578
		S7_62925021
		S1_8022755
		S2_58016455
		S1_8879790
		S9_54882406
		S8_9758380
		S2_6684680
		S1_72537074
		S9_59171494
		S5_66075898
		S4_50758905
		S3_59697794
		S1_2909494
		S1_66309678
		S3_64701681
		S4_1893470
		S2_58202077
		S4_7134910
		S5_11328783
		S3_71370681
		S7_56997734
		S5_5251771
		S1_27546572
		S1_76479612
		S6_53273312
		S1_52708414
		S8_1425944
		S7_60487984
		S6_47114408
		S2_69327056
		S4_7327019
		S2_4555001
		S9_44564241
		S8_51715226
		S6_46536433
		S1_76258052
		S3_5585117
		S8_58124718
		S2_53885447
		S3_51971389
		S5_9537228
		S6_18382004

Catogoery	Total	SNPs
		S1_61008560
		S5_61102734
		S1_9756682
		S6_53299046
		S1_21236483
		S7_59398306
		S1_66723323
		S3_71510618
		S7_63994412
		S5_50472207
		S8_53877326
		S8_2271601
		S2_11592178
		S3_5659693
		S9_5041232
		S8_2625552
		S1_65245533
		S4_7538560
		S5_66622444
		S2_1587797
		S4_11117666
		S1_75039264
		S7_54787393
		S8_2195940
		S6_53317772
		S3_70752187
		S9_50873131
		S1_51753680
		S1_71385681
		S5_68228854
		S9_4266593
		S6_60460611
		S1_78288281
		S3_69704218
		S7_484176
		S7_59390485
		S5_35998639
		S2_63489891
		S9_2619802
		S7_7936843
		S1_53854289
		S1_10567571
		S6_58767474
		S5_10420160
		S2_9492263
		S4_3099793
		S5_1676876
		S3_71068228
		S6_30982177
		S1_9068944
		S1_8641374
		S1_77114897
		S2_62202862
		S4_25806588
		S4_38807111
		S6_47400171
		S9_41464504
		S8_32606385
		S5_66485329
		S3_19997877
		S8_56593463
		S6_15381905
		S3_61017880
		S4_53904102
		S1_68783467

Catogoery	Total	SNPs
		S2_58887566
		S6_8315928
		S1_26919018
		S4_43262152
		S7_58526153
		S9_54094858
		S1_67332873
		S2_73407582
		S4_9672748
		S4_53344046
		S1_10282633
		S3_53587439
		S9_52254298
		S1_60318236
		S1_16455699
		S9_3025216
		S1_24602892
		S2_63670640
		S5_3859405
		S2_56916035
		S4_47808767
		S2_69852902
		S1_77191303
		S2_67991593
		S1_8245887
		S4_5107609
		S3_19373227
		S3_52244722
		S2_19790570
		S3_47917586
		S1_72279633
		S7_3496231
		S2_72606441
		S3_55062362
		S1_11197156
		S6_60926390
		S1_73662967
		S7_278186
		S1_75682267
		S1_8981449
		S2_5673205
		S1_75620811
		S2_65487962
		S4_11197900
		S5_2915298
		S2_4727240
		S6_50005204
		S4_67708843
		S1_73467709
		S7_12688231
		S1_6462599
		S5_13606299
		S3_2098265
		S5_1730229
		S4_7277291
		S9_52077347
		S2_8890439
		S2_46938929
		S5_9550291
		S7_2118494
		S2_7684874
		S6_54381383
		S5_5114697
		S5_43253371
		S5_70773500

Catogoery	Total	SNPs
		S1_78126253
		S2_2315941
		S1_3529569
		S9_41289584
		S1_63014091
		S2_8871629
		S8_44674562
		S1_17220838
		S2_60131028
		S2_69602280
		S9_53301268
		S3_5138261
		S3_63284401
		S2_77032868
		S8_3491638
		S6_51320766
		S2_73412025
		S3_1964493
		S2_12688030
		S2_40687160
		S9_3184779
		S5_69794595
		S1_61774518
		S9_50596147
		S2_64144928
		S2_65383891
		S2_68588398
		S6_42399743
		S2_62739819
		S3_73010845
		S4_1275253
		S7_6326963
		S5_11014821
		S9_3616992
		S6_47043771
		S3_68174615
		S1_16553181
		S2_13177399
		S6_53273384
		S8_54859416
		S2_66175155
		S8_54372905
		S5_59326715
		S3_1842883
		S2_40566612
		S4_3253724
		S2_65271832
		S9_7313790
		S2_3285902
		S4_26502906
		S3_70752021
		S1_73548328
		S2_57607892
		S4_52778308
		S1_58632490
		S5_70773469
		S1_23977116
		S2_68830003
		S2_72258977
		S8_55498839
		S1_53572241
		S1_17276941
		S6_51800085
		S6_54583576
		S8_60255105

Catogoery	Total	SNPs
		S3_15528998
		S4_338525
		S1_60304824
		S2_71704739
		S3_4319213
		S3_55301003
		S4_15309316
		S9_52343007
		S5_6770022
		S3_71997021
		S9_50205670
		S1_76479744
		S9_1108217
		S2_61859445
		S2_11505492
		S2_56515276
		S5_65650492
		S1_18299355
		S3_59637656
		S1_57205855
		S5_65408937
		S9_2740181
		S7_5944360
		S3_58588320
		S6_35109718
		S1_4118977
		S1_59558607
		S4_3924946
		S1_78291203
		S3_72789820
		S1_19640910
		S8_57430143
		S2_8890440
		S6_47954598
		S2_76428058
		S4_5894348
		S8_5976483
		S6_3983902
		S8_46688718
		S5_68110816
		S6_51851630
		S5_70791601
		S2_65466849
		S4_9764518
		S5_1161446
		S4_50531326
		S6_45006481
		S8_4409258
		S3_69578486
		S8_48459085
		S3_1871219
		S8_59749966
		S4_855149
		S2_62309841
		S3_67496172
		S3_64477140
		S4_33698482
		S3_71926817
		S2_76427081
		S3_69541779
		S8_9587552
		S4_56424006
		S1_72859892
		S7_16116907
		S1_71767424

**Catogoery**	**Total**	**SNPs**
		S7_64371618
		S6_53253610
		S7_10063279
		S8_36638825
		S6_47179472
		S5_517170
		S1_11686442
		S9_2576945
		S3_2821847
		S5_11792643
		S4_5085101
		S1_7412651
		S2_61444381
		S4_50462430
		S4_25964143
		S3_68247995
		S4_2302893
		S3_71565451
		S1_5891909
		S8_50345740
		S7_58606390
		S1_17585360
		S7_64405072
		S2_63927721
		S7_18090744
		S6_42500953
		S2_77175534
		S8_4310176
		S2_75155378
		S2_54200742
		S2_6220981
		S6_41878431
		S1_71806232
		S1_15754763
		S3_69419246
		S2_59755623
		S3_57215375
		S3_59308323
		S1_59020555
		S6_44752160
		S1_26252330
		S2_451122
		S5_17987086
		S8_54372912
		S1_19100026
		S3_70480286
		S8_2374776
		S7_63896737
		S6_19129175
		S8_50345772
		S5_7341568
		S1_65250695
		S5_62911324
		S1_67198890
		S2_59276303
		S5_5812341
		S4_16299163
		S2_71485265
		S7_60709296
		S2_10906679
		S7_52328199
		S2_17999598
		S8_57429609
		S1_12305240
		S4_50974937

Catogoery	Total	SNPs
		S5_63374385
		S2_8715470
		S6_41265319
		S1_22810191
		S8_56642728
		S2_61444910
		S3_14150353
		S9_42813000
		S3_63048689
		S9_4531496
		S1_67487321
		S4_51479259
		S1_49924122
		S2_67800525
		S8_51822726
		S2_7096941
		S9_7788425
		S6_52285859
		S4_3371222
		S6_49955695
		S1_71371607
		S3_68814998
		S2_59538090
		S8_55721690
		S1_26854283
		S2_63302471
		S3_69835842
		S8_5441465
		S2_65603812
		S2_68591681
		S1_72434237
		S2_64350277
		S2_3808826
		S8_49963498
		S8_5010704
		S3_73926907
		S6_3228063
		S3_69587304
		S6_47179558
		S5_68952401
		S1_51021982
		S3_72659746
		S9_54020923
		S6_46372077
		S3_55300413
		S1_8879718
		S9_3880751
		S4_6415043
		S6_6658038
		S2_74965506
		S9_52076705
		S7_15658923
		S4_6004949
		S3_56674536
		S2_65651910
		S3_72668857
		S8_53262185
		S9_53672119
		S3_56003507
		S2_54410310
		S8_1179065
		S3_73328805
		S7_15971416
		S4_2273780
		S8_61559529

Catogoery	Total	SNPs
		S2_40336451
		S6_60839164
		S6_47081361
		S2_64360723
		S7_58298409
		S4_51688935
		S5_63651456
		S1_62408268
		S4_34716366
		S8_50645076
		S1_78630283
		S2_76475701
		S3_60719531
		S7_62860164
		S1_7585928
		S2_63512330
		S9_52343005
		S4_7889636
		S8_3219405
		S1_66568089
		S4_2811236
		S2_62320270
		S7_54371352
		S2_15960617
		S2_17162250
		S3_37040994
		S1_1547298
		S2_18779592
		S3_57497685
		S3_69900011
		S8_49214175
		S2_65330880
		S4_4827186
		S2_76492492
		S2_74747246
		S4_63522464
		S2_61785844
		S8_61636252
		S5_69794954
		S2_10527924
		S5_2619021
		S1_67902461
		S7_54307706
		S9_54909031
		S6_49307574
		S3_57925452
		S1_79963586
		S1_2117734
		S1_22272066
		S6_47179475
		S6_46372060
		S4_5579987
		S2_76412196
		S3_5626043
		S8_55617488
		S9_57272968
		S9_50150837
		S1_60836036
		S8_51047380
		S1_66978071
		S2_56976910
		S3_56069575
		S1_58208108
		S2_56600034
		S1_74449263

Catogoery	Total	SNPs
		S7_63699596
		S7_1639801
		S2_10329064
		S8_59586996
		S7_47765916
		S1_14331546
		S6_48553903
		S6_45540278
		S3_67880750
		S8_61581898
		S2_30234058
		S3_55276626
		S8_60394178
		S8_59192353
		S3_73332584
		S9_49551667
		S3_73335141
		S9_3351144
		S5_2681485
		S2_7716252
		S3_14132758
		S3_54098203
		S4_4961125
		S4_7327014
		S6_16411074
		S1_17276961
		S3_63012312
		S1_74327682
		S1_59558238
		S4_11513870
		S3_14144956
		S3_14145258
		S5_63235257
		S6_34857264
		S3_53202911
		S8_38356283
		S1_63672106
		S3_71244966
		S8_57782000
		S4_62988582
		S3_53292792
		S4_23852223
		S6_56756472
		S9_8213334
		S5_1835241
		S7_55993130
		S1_53970495
		S5_58437797
		S5_18181240
		S1_60349463
		S3_62863126
		S7_62377719
		S8_4491970
		S6_44776056
		S9_58114146
		S9_1606560
		S6_48097415
		S1_8504248
		S4_2283971
		S2_60192000
		S3_620445
		S9_57129152
		S6_27450341
		S6_52598639
		S9_51832596

Catogoery	Total	SNPs
		S4_2222112
		S2_63058877
		S8_18161203
		S6_36255788
		S3_14046882
		S1_16670020
		S3_73445244
		S1_3156775
		S8_55153936
		S5_63907964
		S2_57045024
		S7_61166631
		S4_2650385
		S4_9075836
		S7_61086649
		S5_63334850
		S5_5133640
		S1_79434749
		S1_59457263
		S2_10525651
		S2_74747245
		S7_9177631
		S2_60162528
		S2_62312594
		S7_63712274
		S6_54191396
		S2_73693395
		S5_63408376
		#N/A
		S2_11082362
		S6_17741207
		S1_23977139
		S4_13818575
		S8_42472507
		S6_53600221
		S7_58333576
		S9_8599286
		S9_52932651
		S3_48669502
		S9_53301247
		S9_9038596
		S5_1443717
		S8_1363941
		S7_55981150
		S9_52331396
		S6_45679461
		S2_42009082
		S3_61294472
		S5_4006208
		S9_7314053
		S8_3185668
		S5_2547496
		S5_63419128
		S5_69905681
		S3_4241910
		S1_7339830
		S2_75653700
		S1_20372053
		S7_3930636
		S1_7087791
		S8_16332234
		S1_76291130
		S9_41559060
		S8_60694658
		S5_8658676

Catogoery	Total	SNPs
		S2_57258318
		S9_40978341
		S1_6922989
		S5_58643197
		S1_65920552
		S3_15628071
		S6_41921488
		S3_51702763
		S9_51890194
		S5_1931793
		S1_67679852
		S6_58178545
		S4_4912022
		S2_65930401
		S9_55625332
		S1_78585068
		S3_59690589
		S2_7315084
		S1_68301120
		S2_72315421
		S4_66386091
		S2_3707622
		S1_21946040
		S4_66158495
		S2_55129068
		S8_5366723
		S5_6008204
		S5_63585923
		S1_75619549
		S2_3332853
		S2_53110314
		S2_6999141
		S2_57216741
		S2_61786338
		S7_63739876
		S3_67253090
		S3_70121051
		S1_72409294
		S3_26293820
		S4_53280862
		S2_57591453
		S6_6588006
		S5_69780724
		S5_62474024
		S4_53832145
		S3_13405224
		S3_70018317
		S2_60511006
		S4_6465641
		S2_8245706
		S9_51177803
		S9_43668134
		S1_66791770
		S2_14397066
		S2_61560060
		S2_63311591
		S6_51925790
		S9_51705802
		S1_59970698
		S9_54727380
		S4_45391777
		S1_13832002
		S4_4800524
		S4_62878053
		S7_41773164

Catogoery	Total	SNPs
		S1_7412845
		S2_61226303
		S7_58482271
		S4_5107561
		S1_18702722
		S3_68460858
		S1_66564759
		S6_42463476
		S1_13996776
		S8_61925285
		S6_1428755
		S1_2916811
		S8_56349320
		S7_40204431
		S4_62935341
		S9_214849
		S6_49703112
		S1_21735695
		S1_30324851
		S3_55403553
		#N/A
		S4_5530920
		S7_59048277
		S8_58973083
		S1_12305231
		S8_60010355
		S1_57208375
		S2_59125997
		S2_1030570
		S5_61944702
		S4_3998491
		S8_3846927
		S1_9897093
		S8_60934182
		S2_7315051
		S8_4310172
		S1_11100186
		S5_70003041
		S8_6282076
		S2_73694505
		S3_50993732
		S6_45102960
		S9_8924373
		S6_48595839
		S9_47005970
		S3_58315062
		S1_65465340
		S3_55801767
		S7_5919914
		S8_3492783
		S1_12369884
		S4_52350715
		S8_16340616
		S2_53430284
		S8_61980169
		S3_53529719
		S7_64118089
		S8_54372897
		S5_9478813
		S8_49406409
		S7_40979012
		S1_8644868
		S2_6027844
		S9_215162
		S1_59009827

Catogoery	Total	SNPs
		S7_436434
		S2_465954
		S2_72264309
		S6_52885186
		S3_72184013
		S6_49755988
		S8_5780812
		S1_61605967
		S2_61046083
		S8_49406643
		S7_1630104
		S1_10110954
		S8_55494902
		S1_60948205
		S2_73694514
		S2_17707472
		S3_3757318
		S3_64967142
		S8_59214626
		S1_77768523
		S7_2504095
		S6_53285093
		S2_59260788
		S7_2857918
		S2_62635170
		S1_67487338
		S1_66575575
		S6_50964176
		S5_63867640
		S6_47105011
		S4_33082862
		S2_72615812
		S3_55464918
		S7_130915
		S6_46381093
		S9_59169408
		S6_51266890
		S2_47286418
		S1_7998842
		S5_65909608
		S1_20249813
		S5_50559686
		S7_52306550
		S3_57320578
		S2_40321325
		S3_74143094
		S1_72303359
		S5_1836055
		S2_14236543
		S3_56319804
		S6_41984831
		S5_1625522
		S1_78711874
		S2_3209919
		S2_59913883
		S1_14285574
		S9_57068846
		S5_2693059
		S4_42501125
		S1_11197145
		S2_60038549
		S8_4716332
		S3_60935357
		S7_40355509
		S1_59644624

Catogoery	Total	SNPs
		S7_61422904
		S1_7826384
		S6_2003400
		S8_59749664
		S5_42766695
		S3_60581578
		S2_76913334
		S8_3379948
		S3_66708552
		S2_66185287
		S8_61264910
		S4_51276472
		S9_5180927
		S4_66243112
		S7_2696765
		S9_57544917
		S5_437471
		S4_4066702
		S9_54909039
		S8_60160187
		S1_18894024
		S2_12670246
		S6_5962335
		S4_12350785
		S7_574229
		S8_60140790
		S7_64508068
		S7_8824236
		S4_54040407
		S6_25540899
		S8_2472050
		S1_21380720
		S4_1570082
		S6_41858472
		S3_71517528
		S2_65127262
		S1_53853864
		S9_58179021
		S1_77452360
		S6_47071559
		S3_56075316
		S3_52457562
		S5_66622578
		S6_15495914
		S1_74903332
		S1_19244132
		S2_56431699
		S1_1828622
		S1_55705978
		S3_5630446
		S6_53297451
		S1_14437147
		S1_48615128
		S2_6271581
		S2_73412084
		S3_45630323
		S9_50178175
		S4_21046377
		S6_34147765
		S1_76021141
		S9_52172664
		S8_1108468
		S1_6335883
		S8_56141687
		S2_4081141

Catogoery	Total	SNPs
		S3_70238950
		S1_78200476
		S1_53480692
		S2_59576782
		S4_9460635
		S9_54094900
		S1_66128711
		S9_51286265
		S2_67837365
		S3_5067016
		S4_4224452
		S2_242218
		S3_70121176
		S8_1123249
		S9_49965066
		S8_509666
		S5_11029971
		S8_15919808
		S3_61234358
		S2_8124582
		S1_52089032
		S9_44580926
		S1_60697365
		S2_67810203
		S4_34735133
		S7_54506462
		S4_794903
		S1_19727357
		S6_32700344
		S7_55982568
		S7_6220167
		S3_57342087
		S5_69822711
		S3_63580964
		S9_52642715
		S7_64473268
		S2_60595347
		S9_9088196
		S1_79751950
		S1_5892117
		S5_66114510
		S1_24472049
		S1_24604821
		S9_8622289
		S1_78818473
		S2_5489027
		S6_50582469
		S2_60166325
		S3_64573605
		S8_9503617
		S1_71946444
		S6_1238532
		S1_16387720
		S7_54683424
		S2_8879241
		S2_6250941
		S5_67297307
		S4_16512373
		S1_63075710
		S8_3230922
		S1_58096649
		S8_5367448
		S8_57427021
		S1_52256323
		S6_35883518

Catogoery	Total	SNPs
		S4_2090481
		S8_49732598
		S6_54759102
		S7_61407736
		S9_52559127
		S1_61720513
		S3_2488029
		S9_6905169
		S9_54968379
		S2_60110417
		S3_71246859
		S3_811905
		S9_53209141
		S3_57237925
		S8_61921536
		S4_52342972
		S2_1031123
		S7_59040133
		S9_1115500
		S1_4201344
		S5_65910190
		S3_51899477
		S6_11244447
		S9_58070798
		S6_26213795
		S2_61537224
		S2_49535651
		S3_5643265
		S2_76812916
		S6_32048476
		S1_25904824
		S2_61739889
		S7_60216110
		S6_48555129
		S2_54248287
		S3_73362166
		S9_53532348
		S8_2016274
		S2_1587785
		S8_61559513
		S6_53027749
		S3_68941983
		S1_8644813
		S2_6045216
		S1_79775176
		S3_62459762
		S1_71485910
		S1_78035706
		S2_60530136
		S1_60912498
		S7_65343341
		S8_3193966
		S2_69154914
		S6_38360750
		S5_7886796
		S4_1340245
		S4_382282
		S1_74291734
		S8_1403555
		S2_67839036
		S6_2929514
		S6_47571789
		S9_8190316
		S3_57040996
		S1_8881263

Catogoery	Total	SNPs
		S1_20056788
		S5_61188818
		S2_19728007
		S4_51242963
		S7_61507080
		S5_67677746
		S3_6636613
		S4_26502908
		S1_67332439
		S3_5696725
		S4_14183721
		S7_53795480
		S1_65672112
		S2_3967059
		S6_49477829
		S2_10527930
		S8_60382416
		S5_62682853
		S1_29777715
		S1_79853180
		S1_67303950
		S4_7452255
		S2_74636781
		S2_56085782
		S2_4039437
		S8_60938254
		S4_51214783
		S4_56541068
		S1_78833617
		S6_32269412
		S8_61960559
		S1_57951244
		S6_6459379
		S5_62729353
		S2_6036354
		S6_53926664
		S4_13871525
		S6_47498363
		S7_64546561
		S5_5041149
		S1_12968388
		S1_25694541
		S1_60398678
		S1_6040259
		S6_53931716
		S9_8558365
		S5_3182206
		S8_1753895
		S4_43896171
		S4_10580568
		S1_68817204
		S8_49581752
		S7_62475592
		S3_6368786
		S4_51529415
		S4_11531002
		S6_18382058
		S1_2909518
		S8_49231987
		S2_70918227
		S1_53480530
		S9_53172721
		S6_30297630
		S4_8027065
		S2_62393738

Catogoery	Total	SNPs
		S5_6826573
		S8_49625314
		S1_57567720
		S1_79165445
		S6_48553816
		S9_58966344
		S9_4200069
		S7_57692563
		S6_51177337
		S1_56418084
		S1_77056895
		S4_37785228
		S6_53466826
		S3_73067843
		S5_3403485
		S7_7631215
		S2_19732944
		S1_6395571
		S8_1612770
		S1_6244945
		S9_51890219
		S1_73871893
		S3_6644264
		S1_78817982
		S6_42048303
		S4_67078890
		S4_50802865
		S4_53791376
		S7_41193049
		S1_78319569
		S5_2547507
		S1_1547291
		S7_62450371
		S8_53810274
		S6_51865307
		S7_278837
		S4_52473484
		S7_55135128
		S2_66323479
		S2_61525420
		S4_51082278
		S2_44916616
		S1_57101959
		S3_7387996
		S4_1645516
		S1_71994951
		S1_6034722
		S6_51836325
		S3_5130520
		S9_54094852
		S2_5814184
		S3_69587302
		S2_62974684
		S7_59858487
		S2_73102878
		S8_50020244
		S1_7822998
		S1_58036797
		S1_17844540
		S2_76838343
		S4_5855194
		S3_55204439
		S1_65680764
		S9_51417451
		S3_5537819

Catogoery	Total	SNPs
		S4_13278236
		S1_18894047
		S4_55519654
		S3_5787681
		S3_59702744
		S1_25582611
		S3_58247024
		S6_51192551
		S5_69419068
		S1_68798271
		S4_9886176
		S6_28834942
		S4_23819483
		S8_2374793
		S6_25374142
		S9_6694505
		S5_4241651
		S2_59247278
		S7_9919261
		S1_73857523
		S9_5831787
		S6_53740000
		S1_66340751
		S2_68830048
		S1_66894546
		S2_56916503
		S8_42936458
		S1_62720835
		S5_66114449
		S3_71247754
		S2_3478795
		S3_61521493
		S4_6084161
		S5_6750570
		S5_65852078
		S8_51865793
		S1_23964984
		S2_59698437
		S4_41391865
		S1_59791558
		S2_72258965
		S9_54080825
		S1_12595228
		S8_1384258
		S2_6523804
		S5_54661030
		S1_17276956
		S2_59657185
		S5_8838676
		S2_4752034
		S2_3979254
		S1_12933462
		S2_8239207
		S5_9180999
		S5_66360903
		S2_72264043
		S3_47918943
		S9_1260665
		S1_19554073
		S8_47530880
		S1_30172622
		S6_41707241
		S1_61730045
		S1_60717590
		S2_55522417

Catogoery	Total	SNPs
		S1_59539147
		S8_1885155
		S1_63743059
		S2_6178222
		S4_7030241
		S3_57945416
		S8_11324999
		S1_77191317
		S2_66092485
		S3_52362650
		S9_58679975
		S6_6889576
		S4_6019707
		S4_6253869
		S3_70907137
		S4_2086803
		S4_820328
		S6_50364589
		S2_59043393
		S7_10063271
		S9_57657917
		S8_60929764
		S3_64831966
		S2_75420974
		S4_48428694
		S8_54956215
		S9_4743782
		S6_53396973
		S2_59698403
		S9_53301399
		S2_62393743
		S2_60667981
		S2_4554914
		S2_75655114
		S1_63320366
		S4_7951071
		S3_64873886
		S4_11474028
		S8_55247693
		S3_62119853
		S1_7390197
		#N/A
		S2_1030621
		S3_73487649
		S3_20255634
		S8_43093404
		S2_61047525
		S9_1188903
		S2_59645040
		S5_15770649
		S8_3231031
		S5_62499848
		S8_54104110
		S2_75672241
		S5_11615610
		S1_6061365
		S9_52940766
		S9_4072715
		S7_63066163
		S2_115245
		S2_59129546
		S6_47800452
		S1_23907601
		S3_68982603
		S7_58155307

Catogoery	Total	SNPs
		S3_57215935
		S9_54881842
		S3_53480444
		S2_8047290
		S4_784865
		S3_55300986
		S9_8015716
		S2_6239315
		S3_66708042
		S6_47643257
		S1_10593919
		S9_3085649
		S9_43783700
		S1_64105432
		S1_63014083
		S3_71370678
		S4_3755712
		S4_53367562
		S5_65356290
		S4_8815512
		S6_48682625
		S4_66118743
		S1_2736243
		S7_52641880
		S7_62611871
		S2_61698454
		S2_11082334
		S2_62484777
		S3_15436049
		S3_5787681
		S7_54698236
		S7_8931614
		S2_64072296
		S4_41296710
		S1_56695760
		S7_64669445
		S2_47634942
		S4_4541948
		S1_56236426
		S1_61354706
		S9_57737692
		S1_74846159
		S5_22756292
		S6_612031
		S3_3941363
		S6_45545082
		S5_65930937
		S3_72225861
		S9_43147838
		S2_5685726
		S7_60006904
		S1_13463685
		S1_12797909
		S8_31431547
		S8_49124319
		S8_57602684
		S4_54150397
		S1_6034140
		S1_60760857
		S2_64382575
		S4_3911888
		S7_61343929
		S3_4951086
		S2_63692244
		S2_57862869

Catogoery	Total	SNPs
		S2_66175937
		S4_15096605
		S3_61904865
		S2_42945124
		S3_58173323
		S8_57771979
		S2_5720965
		S9_3351680
		S1_59792263
		S6_60934719
		S9_3073711
		S5_65638728
		S8_58744258
		S2_11273385
		S3_60922550
		S7_65097646
		S3_73283686
		S1_76017225
		S2_64258050
		S2_7708905
		S2_10916324
		S6_47674216
		S1_9413546
		S2_11942313
		S2_77014528
		S1_59338088
		S3_53052582
		S6_58569267
		S9_53748201
		S2_62028379
		S3_57879630
		S1_886190
		S1_19033237
		S4_23692085
		S9_49551667
		S9_2857156
		S2_6888032
		S9_51705606
		S3_71458392
		S4_67981583
		S4_62939887
		S8_60246832
		S9_3163089
		S1_17106191
		S3_65495599
		S3_52014443
		S4_66147744
		S1_56337764
		S9_51545688
		S1_14677385
		S8_49102787
		S3_52431137
		S3_4682283
		S1_61680085
		S3_5537863
		S7_64968887
		S2_8114733
		S3_2480055
		S2_12757496
		S6_56819448
		S9_8146994
		S1_8975277
		S2_64495141
		S2_76833038
		S1_59759829

Catogoery	Total	SNPs
		S7_58846925
		S6_42051620
		S1_80699916
		S2_59648488
		S1_6975748
		S2_3708122
		S2_72258972
		S2_72283288
		S4_62988598
		S1_50161476
		S2_25631652
		S5_6101729
		S6_47436662
		S1_59131464
		S2_10219317
		S9_1646681
		S4_7131977
		S2_58682683
		S6_18381935
		S1_2084553
		S2_66175174
		S7_15593373
		S7_58858166
		S6_57946929
		S1_65383287
		S1_16963226
		S7_62233817
		S4_3998844
		S2_64350260
		S7_63117093
		S7_2009638
		S3_73321298
		S3_50993550
		S8_54711057
		S1_11471981
		S4_50518960
		S6_1739549
		S2_66842274
		S7_58526157
		S6_28003890
		S1_16453933
		S2_44073978
		S2_77014530
		S1_15908042
		S1_8504236
		S5_61831127
		S4_1114872
		S2_66928053
		S9_58704691
		S2_65877787
		S1_57208443
		S2_72612719
		S1_65411534
		S1_22298403
		S3_64849472
		S9_51522136
		S6_45570601
		S5_1931846
		S6_27279751
		S1_76739514
		S7_62860157
		S1_48367726
		S1_66652241
		S2_76458521
		S9_504719

Catogoery	Total	SNPs
		S1_79283365
		S3_5111430
		S1_14092355
		S4_53835507
		S6_17003830
		S4_53113145
		S9_58728329
		S2_3759434
		S2_11010724
		S8_49213295
		S3_61719206
		S1_64632574
		S6_47418982
		S2_57518971
		S9_52327120
		S5_373658
		S2_64334141
		S7_59459124
		S7_52290735
		S3_13446365
		S1_22260270
		S5_10899928
		S7_890871
		S3_74316616
		S1_73790037
		S4_67078901
		S7_61602955
		S1_74880837
		S8_2374760
		S1_77861518
		S9_42251071
		S6_42051530
		S9_56932684
		S1_58096671
		S1_7634570
		S1_16543094
		S2_59822168
		S3_62430521
		S7_1789327
		S3_73331265
		S2_68059547
		S6_2843920
		S3_72123789
		S7_122969
		S1_59976393
		S7_42123553
		S8_53982131
		S1_57130604
		S9_51338503
		S7_6679582
		S1_72186042
		S4_39925549
		S6_51822464
		S3_59707976
		S4_44730237
		S2_64872142
		S6_58120478
		S5_3052818
		S3_68744457
		S7_1673369
		S2_10133300
		S1_18693872
		S7_62396533
		S1_79659895
		S7_59048259

Catogoery	Total	SNPs
		S4_5894336
		S3_51804497
		S4_66386092
		S8_55284995
		S5_10900195
		S1_61276186
		S1_22272033
		S2_6100094
		S8_60933907
		S1_20207360
		S1_57110293
		S4_48876251
		S7_64039247
		S2_58290740
		S6_51449251
		S4_66158413
		S3_62858496
		S5_12282140
		S4_6982753
		S8_2624237
		S2_12745591
		S4_12244012
		S1_11584759
		S2_55048564
		S4_54875067
		S2_3756524
		S9_43159605
		S6_53592915
		S4_37103142
		S5_62646211
		S8_55931676
		S2_63921079
		S1_65383260
		S2_56152896
		S3_3910446
		S1_26933887
		S5_3273390
		S1_60885684
		S5_66555617
		S1_75035047
		S2_63693237
		S3_74143088
		S6_17003820
		S4_3906151
		S5_1180493
		S6_58149976
		S3_5202296
		S7_55733924
		S5_70008114
		S9_57152529
		S2_67011044
		S4_11521370
		S5_5114448
		S7_62498278
		S2_17689614
		S4_53842228
		S5_12857226
		S7_55982555
		S1_64698906
		S1_73920054
		S1_11195673
		S2_74972181
		S1_71512907
		S1_72010258
		S6_49499451

Catogoery	Total	SNPs
		S1_57873654
		S2_65855595
		S9_5365187
		S4_53632712
		S1_18002156
		S4_12543731
		S6_53215986
		S1_7263762
		S1_59338088
		S1_12661643
		S2_3134706
		S5_67364913
		S2_12033748
		S4_67711663
		S2_69593747
		S6_50285824
		S8_59749333
		S2_6276353
		S2_59657170
		S2_59637036
		S1_74291730
		S3_68477980
		S1_19201363
		S5_10573573
		S7_60380871
		S8_50018495
		S1_76491708
		S1_73675613
		S6_20569979
		S3_67029958
		S6_50581973
		S9_1110855
		S4_33300738
		S8_55247702
		S4_59009811
		S3_60771055
		S2_59238560
		S8_49584565
		S2_1124539
		S8_48981850
		S1_30559870
		S2_61551394
		S9_53562448
		S2_4635412
		S6_47132684
		S1_16776829
		S2_12735120
		S5_16084008
		S1_59471493
		S8_60452257
		S9_50842437
		S2_60855852
		S2_66298278
		S2_63803732
		S2_57174252
		S1_51003638
		S7_55290255
		S4_44415511
		S9_8407297
		S3_3757322
		S1_16395806
		S2_10527932
		S8_42230584
		S6_53558811
		S2_3415373

Catogoery	Total	SNPs
		S8_53262100
		S3_56946501
		S7_62396712
		S9_6557111
		S2_60105134
		S5_1122051
		S4_4541652
		S3_65928597
		S8_3491414
		S6_52621650
		S7_6412750
		S1_12967861
		S6_7347910
		S9_55046608
		S3_62488498
		S9_8407187
		S2_67878704
		S3_54138339
		S6_49477996
		S6_47955699
		S2_12326360
		S2_60289409
		S2_9484894
		S4_42280501
		S9_44572776
		S1_79195289
		S3_61509339
		S2_62726266
		S6_49673566
		S1_1562902
		S5_4321633
		S7_63982649
		S3_71236541
		S3_57925397
		S1_23805675
		S3_51516403
		S4_53052854
		S3_57312489
		S4_62901565
		S1_66395145
		S2_66967826
		S1_65000081
		S1_7999484
		S2_61587350
		S2_3291295
		S1_11205576
		S5_66315924
		S2_59822415
		S7_65087061
		S6_50414942
		S1_2909496
		S8_49957966
		S9_43458051
		S5_42755170
		S4_54272634
		S8_60452263
		S1_75662789
		S8_55622489
		S2_63028340
		S9_2970250
		S1_71669046
		S2_40566604
		S6_48097603
		S3_4956340
		S6_56572626

Catogoery	Total	SNPs
		S1_78716210
		S8_58574326
		S3_58317335
		S9_5829126
		S8_5320768
		S6_50919259
		S9_1176602
		S5_12536561
		S1_60665087
		S1_16960799
		S1_75616274
		S9_54910908
		S5_37796382
		S2_4640512
		S7_9515478
		S5_63235246
		S2_11765673
		S1_17374447
		S2_66931869
		S7_4784265
		S6_50914994
		S7_60727531
		S1_16399201
		S1_67220503
		S5_63499307
		S1_51003643
		S1_27150331
		S1_2742238
		S2_72639799
		S8_54754698
		S6_51865012
		S1_56444106
		S6_27279746
		S6_48244471
		S1_63891419
		S4_53842236
		S2_12101690
		S3_57267477
		S2_9849110
		S4_3704674
		S4_5530981
		S2_16844370
		S4_52608924
		S8_2271824
		S4_6184903
		S9_25827565
		S3_72789872
		S3_53472851
		S4_3800779
		S6_54594772
		S8_5322980
		S5_12278992
		S3_73319074
		S1_8975375
		S4_7134877
		S1_10687793
		S3_67966292
		S1_74378033
		S2_63194976
		S1_52755678
		S8_46431808
		S9_57152424
		S1_7738894
		S5_2928742
		S6_54309975

Catogoery	Total	SNPs
		S1_2912083
		S9_57611548
		S8_44340134
		S6_35904840
		S7_6054967
		S2_6687129
		S3_4575085
		S3_73319061
		S2_67765540
		S6_45994088
		S1_65245253
		S4_24300024
		S7_10006159
		S1_53932713
		S5_1163412
		S8_56462535
		S2_68570843
		S1_19645665
		S8_54956242
		S3_60884259
		S4_14905200
		S2_6178215
		S2_64495147
		S4_4826801
		S3_54114695
		S6_13863333
		S5_61208220
		S3_3700896
		S8_7197901
		S1_7289723
		S2_54248345
		S8_54939564
		S1_61604438
		S6_53672974
		S7_41279614
		S4_66243123
		S9_54039444
		S8_519158
		S6_35292542
		S7_17960155
		S1_77764926
		S1_10648425
		S2_58238991
		S6_54250710
		S8_44938811
		S1_78565497
		S3_67760107
		S3_64354546
		S8_56721707
		S5_23666481
		S9_52837786
		S3_72109042
		S7_889904
		S5_62950665
		S7_15971452
		S6_47280460
		S6_47538929
		S9_52032957
		S1_17491359
		S5_65910308
		S8_493057
		S2_9569320
		S1_15851565
		S1_64159708
		S7_63982658

Catogoery	Total	SNPs
		S1_18923807
		S6_47693927
		S3_59723489
		S6_41984824
		S1_57222263
		S3_58246383
		S4_40271277
		S8_54927859
		S1_66894541
		S4_14983540
		S8_5255583
		S2_75649815
		S7_58606407
		S5_22467689
		S7_5154442
		S3_58381843
		S8_61767753
		S5_11114776
		S8_60949187
		S5_62473922
		S1_76020393
		S1_9903433
		S7_38972032
		S1_76495676
		S9_55582736
		S6_50435864
		S6_52118191
		S2_12744930
		S1_75513440
		S7_7631220
		S3_53323835
		S7_56728457
		S7_53150028
		S6_56806436
		S2_63676091
		S9_2228499
		S8_3217814
		S1_67679481
		S2_2752790
		S3_5130555
		S1_57380723
		S2_69858460
		S3_71378021
		S2_63377436
		S3_73610688
		S5_3179744
		S8_3379948
		S5_8655284
		S1_15798013
		S3_68478055
		S7_40823641
		S4_43064336
		S5_1443739
		S5_10819819
		S1_65742383
		S3_55403560
		S3_69631283
		S7_54307715
		S2_64350288
		S2_61444907
		S3_68057883
		S8_38967454
		S2_61785622
		S9_50895130
		S2_55354472

Catogoery	Total	SNPs
		S1_3539934
		S2_6611787
		S9_41589308
		S8_58489025
		S3_5130763
		S9_3351681
		S3_64874095
		S5_2693011
		S8_3491661
		S8_2615007
		S5_67071220
		S8_5376806
		S2_61508024
		S1_6313852
		S5_63115677
		S1_60148050
		S1_6512011
		S7_14780296
		S1_58913449
		S2_12670179
		S8_2287248
		S1_60517658
		S6_44752311
		S8_59681242
		S7_6679582
		S4_51398291
		S4_2641502
		S5_3230485
		S9_58926840
		S2_6684658
		S6_58759676
		S4_51612357
		S6_50656469
		S4_46974526
		S5_54661202
		S2_71081861
		S3_68465961
		S2_68991192
		S2_38295115
		S8_51715243
		S2_7878117
		S1_18535348
		S7_60488157
		S9_52833811
		S9_51705468
		S5_57207741
		S5_4752779
		S2_2007357
		S2_56077085
		S6_51221959
		S1_60960591
		S4_5025258
		S2_16111315
		S7_7287643
		S8_56933351
		S6_47955883
		S5_66213616
		S2_262334
		S6_50507779
		S5_10688308
		S1_61975278
		S5_5114692
		S2_8890437
		S1_52230640
		S1_51841734

Catogoery	Total	SNPs
		S4_3832201
		S1_70130035
		S1_67487755
		S2_62493236
		S2_66339833
		S6_47343543
		S1_49363695
		S2_3602213
		S5_18072670
		S9_51521876
		S9_50520646
		S7_64759051
		S6_37152360
		S9_1663475
		S6_9690316
		S2_60577683
		S6_39672528
		S1_66841125
		S2_59249478
		S2_12466183
		S5_38107233
		S4_1898068
		S4_52778240
		S3_73160953
		S1_12935959
		S2_11629112
		S9_8377122
		S3_74142894
		S5_61929618
		S3_66699219
		S2_66633915
		S3_73791996
		S2_15193073
		S1_76432131
		S8_56141648
		S7_62219208
		S7_52129253
		S1_59795617
		S1_30422284
		S5_61792509
		S7_58773838
		S2_56135667
		S2_10750453
		S9_1111023
		S5_11792657
		S2_75952044
		S5_62493268
		S4_1243400
		S7_17448672
		S2_12993202
		S3_66415870
		S3_6722673
		S6_58890943
		S1_3440415
		S2_62739822
		S9_49295377
		S1_6335866
		S9_58778873
		S9_49009554
		S4_39961391
		S7_16529379
		S1_67679849
		S6_1238591
		S1_74972012
		S1_70466571

Catogoery	Total	SNPs
		S6_48630850
		S2_75437432
		S7_62401302
		S6_1668328
		S8_9590455
		S9_43444602
		S2_72264265
		S5_18541784
		S8_3173770
		S1_15024028
		S8_2271664
		S5_23824099
		S3_58531268
		S3_68503276
		S1_4200397
		S4_10419212
		S2_73340287
		S4_52473572
		S1_25085168
		S2_7314946
		S7_41122205
		S4_5107397
		S1_58634022
		S5_4355103
		S9_53708344
		S9_1260248
		S3_57291583
		S1_49036439
		S1_56295521
		S3_55300420
		S8_2183187
		S2_63726308
		S7_6766153
		S1_63954166
		S2_6043994
		S1_65740937
		S9_50525497
		S4_54119598
		S4_49492100
		S1_59557503
		S2_60289520
		S6_53396918
		S5_69780736
		S3_67023354
		S8_47581924
		S4_58246341
		S1_56195470
		S5_70006547
		S3_66408366
		S4_702515
		S4_1788078
		S7_62999427
		S6_44836595
		S4_53482573
		S1_50787150
		S2_67332157
		S4_7452264
		S4_45562457
		S9_2931138
		S8_412098
		S9_58728450
		S9_54111774
		S2_59619346
		S4_3728352
		#N/A

Catogoery	Total	SNPs
		S5_61850789
		S2_62723328
		S7_41422252
		S6_1912550
		S5_62156728
		S2_61926691
		S8_45716385
		S5_9633712
		S6_1454183
		S2_11737283
		S4_2080718
		S7_38852212
		S1_58674956
		S1_6311224
		S7_42120079
		S1_47594626
		S4_42264570
		S8_3380610
		S4_43690888
		S1_72859895
		S1_75461955
		S1_61162251
		S1_7412560
		S9_55626936
		S1_15908608
		S1_30132769
		S9_57547454
		S4_4349277
		S6_6447848
		S4_1309488
		S3_46065624
		S1_76335136
		S1_51417984
		S5_1248457
		S9_7314093
		S1_6923034
		#N/A
		S4_4444705
		S4_5894343
		S8_3130446
		S2_59467313
		S7_278838
		S4_52124269
		S6_46381703
		S5_2245292
		S3_47617428
		S8_60379154
		S1_19376300
		S5_5612999
		S1_48857709
		S7_436433
		S8_11323079
		S9_3894718
		S3_4009002
		S4_53791389
		S8_60548722
		S1_71493837
		S7_17141128
		S3_56681929
		S3_55195204
		S1_11205227
		S1_8504148
		S1_14872704
		S2_7541520
		S8_9841350

Catogoery	Total	SNPs
		S3_74310313
		S2_69829331
		S3_55345691
		S2_59576786
		S4_1609525
		S6_49221232
		S4_54119514
		S5_55344505
		S1_57683404
		S1_56161575
		S3_58080843
		S6_36746042
		S4_61204000
		S8_49124295
		S1_11562514
		S3_61711170
		S8_50408500
		S2_6526451
		S3_63762616
		S5_16114160
		S2_62996588
		S1_74546930
		S6_30833300
		S8_1325632
		S2_67004382
		S9_50087816
		S6_53563554
		S2_42945119
		S8_44849405
		S9_7314159
		S4_6798777
		S7_15663001
		S2_50067109
		S8_41733692
		S3_60771044
		S3_63251877
		S1_71822906
		S1_60837174
		S9_49281138
		S3_53175444
		S1_77183319
		S1_73887181
		S2_6235913
		S5_20276467
		S4_6773641
		S2_75273455
		S8_5480227
		S5_2827550
		S2_2801809
		S8_3380026
		S9_50965430
		S1_76633532
		S2_4396183
		S7_6905754
		S4_23763103
		S7_58298410
		S3_55801617
		S8_58464015
		S9_51890198
		S1_58632047
		S1_10110951
		S7_5538179
		S9_3791615
		S4_39942869
		S9_53527845

Catogoery	Total	SNPs
		S9_51791427
		S1_74990701
		S1_74785516
		S3_62447135
		S1_73446736
		S1_24790295
		S5_68175308
		S3_5659671
		S1_15851565
		S9_52077318
		S4_34738677
		S7_1163367
		S8_57175660
		S9_44572778
		S4_4224476
		S1_59360157
		S1_9980134
		S5_1754475
		S4_10304304
		S7_6849693
		S3_2480018
		S3_55311781
		S1_49363714
		S2_6807050
		S9_54114067
		S3_55139288
		S2_12101818
		S4_37459978
		S6_953245
		S1_9066118
		S1_11197148
		S9_2984313
		S1_73639818
		S1_5800636
		S9_6344407
		S8_9590412
		S3_73634664
		S8_51188299
		S7_2920327
		S5_6730401
		S1_73324074
		S4_4083275
		S1_13284828
		S1_7093613
		S1_67715926
		S7_15926701
		S3_72659678
		S4_38930785
		S3_62383700
		S4_53344055
		S9_51277330
		S7_16083824
		S1_58036770
		S9_49058643
		S8_49399687
		S1_56030216
		S3_4756380
		S8_54218797
		S1_18079487
		S3_73239362
		S7_6679582
		S6_58739484
		S1_77324798
		S5_62503575
		S3_65633876

Catogoery	Total	SNPs
		S5_1608322
		S3_16637181
		S1_78784588
		S2_59619316
		S9_5861505
		S3_71323954
		S2_76499219
		S7_8994937
		S5_12536381
		S3_73010783
		S2_67930877
		S1_78397582
		S2_66185152
		S6_49816306
		S4_53778467
		S4_7562124
		S9_57791058
		S9_3272831
		S8_5743932
		S4_6756911
		S3_69569786
		S4_67316821
		S4_68098069
		S5_1754405
		S2_56440834
		S2_68059571
		S1_10100815
		S1_67381868
		S3_68981453
		S4_6371297
		S6_41007973
		S2_64072286
		S7_6633798
		S7_9425916
		S1_66791923
		S7_9788487
		S3_56758954
		S1_18657311
		S9_55067184
		S4_33504396
		S3_69396464
		S4_1053921
		S9_52246002
		S6_36790477
		S2_63356162
		S8_48161906
		S3_59368495
		S4_25645613
		S1_60849856
		S3_73014264
		S1_65289316
		S8_1876447
		S2_73694518
		S7_8438684
		S4_7273383
		S6_47074609
		S9_51911948
		S2_73277337
		S3_72184266
		S6_31304931
		S1_73686556
		S2_25439194
		S6_335477
		S2_74956028
		S3_4956349

Catogoery	Total	SNPs
		S3_15554312
		S3_71698461
		S8_52026396
		S3_72123808
		S4_9633339
		S7_63157581
		S5_1966414
		S4_50769723
		S2_75248874
		S9_217786
		S1_62536614
		S1_72790891
		S1_9259130
		S1_16552921
		S2_57264521
		S1_59140852
		S9_51524280
		S9_56116251
		S8_1172628
		S1_29430245
		S5_61634011
		S2_53112209
		S7_54573343
		S6_53439478
		S2_59370730
		S1_67610048
		S3_56869294
		S7_15591243
		S5_65221378
		S2_66967844
		S3_71247272
		S2_61589003
		S3_51970959
		S5_1666182
		S2_38768420
		#N/A
		S5_67634004
		S1_17486371
		S2_61333390
		S3_51903136
		S9_58179024
		S8_61256666
		S2_17689658
		S2_75055887
		S1_80075809
		S6_45006719
		S2_47145760
		S3_20255067
		S3_67762480
		S3_73887812
		S5_10900190
		S2_63933907
		S8_1664235
		S3_58432015
		S8_56379520
		S1_77081611
		S9_2931119
		S8_49214212
		S4_51082368
		S1_20207352
		S4_2628105
		S4_66144669
		S6_46696694
		S1_7643982
		S8_59751088

Catogoery	Total	SNPs
		S7_3637423
		S2_69025303
		S9_5152028
		S9_52207525
		S3_57894986
		S7_6905763
		S3_69833572
		S6_46735174
		S1_61659836
		S3_68460865
		S3_65838174
		S1_11192678
		S2_45643806
		S1_79164615
		S6_45570743
		S3_65332822
		S4_54072757
		S7_61282980
		S9_7342471
		S1_67311338
		S1_72577058
		S3_15665285
		S3_70245263
		S4_51115835
		S1_16415209
		S7_65086481
		S2_61444405
		S1_6336245
		S7_54573411
		S5_47910419
		S3_64197184
		S4_56426042
		S2_68815437
		S1_73770003
		S2_58302475
		S1_15851565
		S2_49470365
		S2_60589380
		S7_54797120
		S5_6847569
		S8_1753899
		S8_58489567
		S8_3510642
		S6_1375081
		S9_8129280
		S4_361725
		S1_49433062
		S2_2649202
		S4_1004444
		S3_65965667
		S6_41172852
		S5_66675924
		S2_58201045
		S8_57441596
		S2_67991002
		S2_75509968
		S1_56567575
		S3_65906476
		S4_7539024
		S7_58846903
		S1_13102064
		S9_51068872
		S7_61527360
		S6_45540268
		S6_54462516

Catogoery	Total	SNPs
		S1_12247835
		S3_73610514
		S9_52204483
		S4_23803467
		S2_19920309
		S6_48638199
		S5_9537589
		S7_2696762
		S1_67610048
		S5_62396398
		S7_62927197
		S3_69587279
		S2_60926850
		S2_62264946
		S2_6940567
		S3_67496145
		S4_12085536
		S3_68919633
		S4_23763108
		S8_42141496
		S9_50150034
		S7_52290584
		S2_66492135
		S3_51997014
		S2_68059557
		S8_61646345
		S3_56662681
		S9_54316399
		S9_4267920
		S2_68759229
		S3_67492554
		S6_40434590
		S3_70531672
		S5_61749004
		S1_7499624
		S2_64350143
		S1_59658667
		S2_59538585
		S8_61278748
		S6_30447404
		S5_62918887
		S3_60935357
		S5_37446310
		S1_73467704
		S8_4310173
		S7_54307646
		S8_48961244
		S6_31881958
		S4_19509318
		S1_14260296
		S8_56724646
		S3_57223922
		S7_62132143
		S7_62143571
		S4_7468080
		S6_493556
		S3_6386489
		S2_10327187
		S3_70604368
		S1_75746042
		S1_7957954
		S8_3049691
		S8_49625595
		S6_60763316
		S1_14080877

Catogoery	Total	SNPs
		S5_6769989
		S4_699734
		S2_69710836
		S8_49191739
		S3_64325427
		S2_75259010
		S3_16632220
		S4_3730651
		S5_68128176
		S5_4494471
		S6_45634750
		S4_13848449
		S9_48996408
		S9_3805176
		S1_18956124
		S1_65750442
		S6_53861099
		S6_53240809
		S1_63510574
		S2_59018267
		S6_14254960
		S4_39854462
		S2_33743321
		S3_48837756
		S7_62773862
		S4_11520801
		S8_15447548
		S7_63318981
		S3_4750842
		S1_28479320
		S2_72343648
		S1_78938511
		S8_62007963
		S8_3173716
		S2_33051954
		S7_59752055
		S7_6984853
		S2_41949447
		S3_72310947
		S1_8881260
		S2_57607808
		S8_47529566
		S1_25085170
		S5_69879740
		S2_61085918
		S1_18912744
		S1_9684123
		S7_60561333
		S7_60724031
		S1_63759391
		S6_53726923
		S1_71946409
		S3_72078402
		S9_54881865
		S1_15917434
		S4_66042654
		S5_70006530
		S4_54095343
		S2_62393739
		S9_9099733
		S2_3881144
		S6_52581223
		S1_65255404
		S7_14233709
		S1_17276951

Catogoery	Total	SNPs
		S1_48458062
		S8_60478514
		S7_15311029
		S2_63692311
		S1_64993139
		S1_73434975
		S1_5707279
		S4_2233567
		S3_70598789
		S5_67071220
		S1_11192727
		S2_76451579
		S8_41597145
		S1_2626426
		S7_58851771
		S5_66622083
		S6_52762904
		S2_5965525
		S2_69840253
		S3_71884884
		S1_46164045
		S6_60761011
		S2_60322406
		S5_69851434
		S6_49330746
		S8_15444542
		S2_3881068
		S4_66125775
		S5_68989246
		S9_51069061
		S8_50736890
		S1_67487688
		S4_34910956
		S1_77764816
		S3_58370459
		S9_53727734
		S3_55024346
		S1_74489537
		S8_41047154
		S1_73551524
		S9_3899031
		S2_61525630
		S4_55505648
		S1_78161439
		S1_4473679
		S2_66606189
		S2_76832011
		S7_987379
		S4_1836209
		S3_2016729
		S3_67503296
		S2_18761326
		S2_63028300
		S6_50364656
		S8_49933733
		S1_7639594
		S1_74100523
		S1_59791332
		S3_1851001
		S4_22999288
		S1_7509688
		S6_50932315
		S1_19664236
		S7_61501041
		S7_59397330

Catogoery	Total	SNPs
		S6_50656467
		S6_51420212
		S1_66022241
		S6_53397094
		S1_56744959
		S9_55048136
		S1_8998673
		S6_58767169
		S1_47338389
		S4_9075828
		S4_53344061
		S3_73241452
		S3_71866622
		S9_8034968
		S8_2614968
		S2_65318259
		S4_50273338
		S2_63354501
		S6_58117324
		S1_49925465
		S9_54120588
		S1_9811576
		S7_64211683
		S5_65464672
		S5_6176824
		S8_60351545
		S1_74326346
		S1_18421291
		S1_22272036
		S4_54004029
		S5_24096084
		S1_2659326
		S8_59983553
		S9_53934233
		S1_18914563
		S9_53950760
		S2_56869860
		S3_46901245
		S5_61208127
		S1_18956498
		S7_57892951
		S1_66676262
		S3_5643255
		S5_1052308
		S4_16183941
		S6_50396627
		S1_5703862
		S8_49214198
		S3_62176790
		S3_73087358
		S1_4120787
		S3_3900249
		S8_2194333
		S2_62264946
		S9_2531722
		S1_71946423
		S8_55256554
		S9_7538616
		S1_7268073
		S8_49730445
		S6_51193395
		S1_68301838
		S2_59799809
		S1_60577602
		S4_56200609

Catogoery	Total	SNPs
		S2_1030814
		S8_55614036
		S3_69485302
		S2_1587775
		S1_31205594
		S2_71569861
		S2_8890434
		S6_40512605
		S5_65413215
		S5_68080124
		S1_67221069
		S7_64417737
		S2_66708930
		S2_8879238
		S5_10814633
		S3_61877426
		#N/A
		S5_1326995
		S2_6271136
		S1_57194502
		S1_15854583
		S7_64826543
		S7_1640031
		S6_53901828
		S3_65912407
		S4_2627001
		S4_21235743
		S4_7868537
		S2_8114711
		S1_77343707
		S6_48241265
		S4_452117
		S4_41454729
		S2_4640610
		S1_72060316
		S4_1339470
		S8_55721683
		S9_52930421
		S5_67595989
		S1_69091174
		S3_73184096
		S1_18913447
		S3_68058304
		S1_57665164
		S6_38194504
		S3_6089452
		S1_4144105
		S2_72360180
		S1_15854575
		S8_50122445
		S5_1795528
		S9_7224688
		S7_5963718
		S9_56004249
		S2_65386763
		S6_50657604
		S6_7198291
		S2_64563633
		S2_76943615
		S3_57291959
		S2_60296104
		S5_61217576
		S2_73312593
		S5_10795307
		S7_39945489

Catogoery	Total	SNPs
		S4_67675597
		S8_61641337
		S2_67765560
		S1_5800637
		S2_11944478
		S2_60347306
		S8_5320758
		S8_56604812
		S6_46595596
		S2_40078239
		S3_70840763
		S6_3723381
		S2_57206195
		S1_18967442
		S1_20459763
		S1_17864319
		S2_4460772
		S4_958842
		S1_67928656
		S1_6777446
		S3_74143095
		S1_7499637
		S3_50578864
		S2_6799089
		S6_1168843
		S4_50116287
		S8_5322989
		S1_78509956
		S2_16136096
		S7_1630083
		S2_3332744
		S5_19750428
		S4_43107270
		S1_16439941
		S7_1768239
		S5_10561662
		S1_76291134
		S1_14875232
		S4_44067993
		S1_72373912
		S8_1637329
		S7_437490
		S1_15851565
		S1_65725783
		S3_52994987
		S9_767061
		S3_5787681
		S2_1462270
		S6_47306908
		S1_19154281
		S2_3785447
		S4_51951354
		S2_72324175
		S5_4006132
		S2_11508828
		S3_73010509
		S8_13070173
		S6_1744158
		S6_50437088
		S1_14092358
		S2_12746311
		S6_366829
		S2_73888898
		S1_66832550
		S1_12065426

Catogoery	Total	SNPs
		S7_63884790
		S1_10082060
		S6_17583119
		S5_15887134
		S2_70921760
		S6_50269789
		S5_11792660
		S8_2626842
		S1_7782199
		S6_25705367
		S4_68097216
		S3_73560198
		S7_58340549
		S7_58690963
		S5_1248469
		S9_59192519
		S7_52328074
		S4_23657080
		S7_61414435
		S2_58286569
		S3_6609398
		S7_6671340
		S9_58742723
		S3_73160961
		S2_359929
		S5_1795447
		S2_62746006
		S6_27279744
		S5_65772344
		S6_51419495
		S3_58736887
		S5_2935735
		S7_64067748
		S6_53600052
		S2_18494540
		S3_61852103
		S1_25897350
		S2_72265477
		S3_61014220
		S6_57948177
		S5_62950662
		S2_10269501
		S2_69866749
		S8_5140431
		S6_53857087
		S2_63583671
		S2_66429833
		S8_62112161
		S3_68742705
		S1_59538746
		S3_62273282
		S8_30901859
		S2_6396269
		S2_1612811
		S9_42812943
		S4_50569597
		S3_56099945
		S5_4405608
		S2_75768335
		S2_74073529
		S2_6687225
		S9_56968845
		S6_46372073
		S1_66128666
		S2_6685950

Catogoery	Total	SNPs
		S8_44848319
		S2_58210498
		S2_47680718
		S9_41261202
		S4_43670071
		S2_59425885
		S4_33300727
		S5_3393114
		S1_12797819
		S1_6229786
		S9_5910426
		S3_36119062
		S4_67087259
		S8_49406410
		S4_33543084
		S9_10120197
		S9_8407272
		S3_71236538
		S2_59706883
		S5_66471113
		S3_6277277
		S2_11550190
		S9_53303277
		S6_53622969
		S2_65386269
		S2_76832017
		S1_66564760
		S2_60561850
		S3_70039460
		S1_72061552
		S6_953249
		S1_11554766
		S1_2533247
		S6_44777441
		S5_2548697
		S3_38419865
		S6_50396678
		S3_63298224
		S7_60259800
		S1_17468708
		S8_39575798
		S8_61256624
		S4_42682269
		S4_56265756
		S3_69937671
		S1_17882143
		S1_16399139
		S9_50676351
		S2_55292382
		S6_54774497
		S5_66405185
		S1_68783468
		S1_31011799
		S6_22973974
		S3_65423107
		S2_42945129
		S1_23912907
		S1_24771019
		S5_59372522
		S2_25631612
		S2_61637226
		S1_59020559
		S3_61963281
		S5_62911220
		S8_11442936

Catogoery	Total	SNPs
		S1_51236860
		S8_60933918
		S1_51860580
		S4_62913267
		S6_41904470
		S3_4951086
		S6_52807552
		S8_60382360
		S9_57811715
		S4_41445290
		S8_55930248
		S8_53840222
		S2_9579744
		S1_77343724
		S3_4690805
		S3_48674579
		S1_73441345
		S8_49845547
		S2_59619400
		S9_3881599
		S7_59404098
		S8_45425620
		S5_8748932
		S2_76427351
		S6_47765264
		S3_60919675
		S6_46727121
		S2_9727776
		S4_48391116
		S4_38463048
		S4_12187607
		S4_15171204
		S2_9463521
		S1_496319
		S6_41598451
		S7_889718
		S1_16963241
		S1_7163375
		S1_59791555
		S7_14463384
		S9_981128
		S5_67940571
		S4_52875863
		S4_4847331
		S4_56437185
		S1_62823744
		S2_60130991
		S5_66607892
		S6_1325763
		S2_6136672
		S8_9637902
		S4_56610940
		S2_67873573
		S1_63672178
		S7_56731761
		S2_8258230
		S5_61209441
		S5_63419089
		S2_63195257
		S2_61561837
		S4_15604157
		S2_10221512
		S8_35621760
		S3_56243464
		S8_50611849

Catogoery	Total	SNPs
		S5_68176074
		S6_30404287
		S2_73494442
		S7_58333598
		S3_72534686
		S2_66175937
		S7_63671794
		S5_69082068
		S2_58395524
		S7_1488727
		S1_1836188
		S1_59338091
		S9_5153680
		S3_70528562
		S1_59567025
		S3_3757325
		S5_22297153
		S2_49330090
		S8_55516995
		S7_40497554
		S1_74291834
		S6_52285483
		S1_57347187
		S9_50425555
		S1_26887766
		S1_14515184
		S3_60771029
		S7_64774396
		S1_21826870
		S6_49768868
		S3_5009509
		S9_1085086
		S2_16380632
		S8_61165330
		S6_28748324
		S1_54031701
		S1_74530762
		S4_7327018
		S7_63982660
		S1_14860775
		S1_28678211
		S2_25324642
		S9_52077262
		S1_65259179
		S8_56504352
		S8_60139638
		S1_9822433
		S1_68684662
		S1_57130607
		S5_1761161
		S2_58201025
		S1_14483501
		S7_5302956
		S4_14790492
		S4_8110122
		S1_58096672
		S5_65658102
		S8_2121726
		S1_16456260
		S2_62264946
		S2_71486256
		S1_74510247
		S2_49872325
		S1_4473603
		S1_18967255

Catogoery	Total	SNPs
		S5_11706294
		S3_61818894
		S6_52206687
		S6_50307477
		S9_56108637
		S9_59378884
		S5_61168161
		S1_20207183
		S1_60912497
		S2_66633652
		S1_64307521
		S9_3025515
		S4_34652073
		S2_16479856
		S9_51493655
		S8_57232907
		S4_3846682
		S4_40968104
		S8_60452266
		S6_54203488
		S9_55626939
		S2_57442112
		S7_64603327
		S7_6882216
		S1_58192523
		S1_6466521
		S3_63172175
		S6_953241
		S2_75437401
		S3_71217382
		S2_68557075
		S7_19544751
		S1_10687796
		S2_67845962
		S8_31558483
		S9_54956455
		S2_1141781
		S3_57237925
		S2_11857317
		S9_1241143
		S8_2736401
		#N/A
		S7_58339399
		S3_61234358
		S1_56468386
		S4_55505263
		S3_68055680
		S2_66175542
		S1_26252299
		S5_3866567
		S2_60922431
		S4_8601477
		S5_62378840
		S1_50341798
		S8_1238929
		S3_73160929
		S1_66564576
		S1_2703706
		S8_61559518
		S2_60561607
		S7_63019083
		S1_46824068
		S7_56660943
		S8_6158339
		S7_15529674

Catogoery	Total	SNPs
		S3_5585586
		S2_59425912
		S3_61915753
		S7_61527358
		S7_57557687
		S8_1438074
		S3_6748292
		S8_3219404
		S9_57152517
		S7_54307714
		S9_7788418
		S2_3334098
		S1_62068108
		S4_53749628
		S1_11466449
		S2_71063628
		S3_70013910
		#N/A
		S9_50974213
		S1_66904973
		S2_488009
		S1_12305195
		S2_73366315
		S4_5866927
		S6_493568
		S2_65289544
		S4_1251236
		S1_52902344
		S8_1430488
		S1_2297968
		S4_33868461
		S4_62307778
		S3_56257040
		S1_66728699
		S3_60583220
		S9_53202191
		S1_72330084
		S3_61856025
		S4_49718639
		S8_47774235
		S6_61057499
		S7_62647371
		S2_72604952
		S1_61978777
		S8_42702399
		S7_61286154
		S2_67849734
		S2_62315733
		S2_76810674
		S3_45569125
		S1_72302170
		S6_60774378
		S9_5861467
		S4_4007324
		S9_1594516
		S1_64803278
		S3_56243557
		S1_77097342
		S1_20207213
		S3_50672317
		S3_51995650
		S6_38523821
		S4_52413681
		S3_72022935
		S1_63213910

Catogoery	Total	SNPs
		S2_73103121
		S9_51666254
		S5_61102574
		S2_9905517
		S1_52255941
		S3_1222907
		S1_11205152
		S8_35800478
		S5_5249179
		S4_34440059
		S1_12227044
		S2_65043004
		S5_6769987
		S4_6119849
		S1_4144034
		S6_16435911
		S7_62118571
		S7_774671
		S6_48595403
		S2_6237474
		S7_2492149
		S3_55300412
		S3_56064485
		S5_68815213
		S2_66469977
		S1_66245325
		S9_7538593
		S6_58811220
		S2_61240409
		S2_67873307
		S6_48313456
		S6_1299797
		S5_23969588
		S1_22746200
		S5_63826156
		S5_61792545
		S5_39368665
		S1_7993773
		S1_71823019
		S5_1931830
		S8_2610517
		S1_18967676
		S1_71371615
		S3_55024394
		S6_1312714
		S1_78032412
		#N/A
		S8_59178415
		S6_50647192
		S4_67082966
		S6_14254958
		S9_1124569
		S5_6919510
		S6_52598614
		S1_79052023
		S7_56589399
		S1_78190410
		S4_6174629
		S6_54759094
		S5_59372528
		S2_6691796
		S7_49107813
		S1_31593363
		S6_1668343
		S2_12927587

Catogoery	Total	SNPs
		S6_3537363
		S6_6585769
		S8_54104156
		S1_66110870
		S3_64984083
		S7_55105368
		S1_8482129
		S1_11582002
		S6_46372053
		S1_78999662
		S1_67623685
		S1_31765682
		S8_4131946
		S6_37905254
		S4_3766246
		S5_63238687
		S7_8118903
		S2_16270378
		S2_59982811
		S3_781111
		S5_61864606
		S9_972171
		S2_77032867
		S4_35510721
		S5_4062993
		S1_1346271
		S9_1176604
		S8_49983700
		S2_75155380
		S1_58349481
		S7_51847243
		S9_1675490
		S9_42753965
		S8_53754906
		S9_4787564
		S3_2117537
		S3_73319075
		S4_2627022
		S2_63124751
		S3_2219162
		S1_57944354
		S3_70215081
		S3_55801446
		S2_6235370
		S3_61517392
		S4_39051120
		S1_11122169
		S7_63698652
		S5_61064037
		S5_11029977
		S7_59142882
		S2_63489892
		S9_3741357
		S4_5085183
		S8_1840290
		S1_64803657
		S3_60919194
		S6_45542927
		S2_74874001
		S6_45102924
		S3_62273236
		S6_26308209
		S2_13167120
		S7_63618745
		S4_62882446

Catogoery	Total	SNPs
		S3_73160933
		S5_1931789
		S2_56931827
		S6_58812630
		S1_8437584
		S5_1068107
		S1_59759826
		S4_5855195
		S9_9099736
		S1_57111261
		S3_70474180
		S3_70901591
		S9_7311397
		S9_10737216
		S4_2504858
		S9_214871
		S1_51003648
		S6_48594515
		S1_15851565
		S8_2050987
		S2_62799835
		S9_52932640
		S9_6239278
		S3_71926891
		S8_57767399
		S8_57525282
		S7_53852802
		S2_73693973
		S3_73321285
		S7_436432
		S8_57452501
		S9_1826341
		S6_47063160
		S2_68092281
		S3_71247758
		S1_9921045
		S9_59383161
		S2_60191883
		S4_38438995
		S8_49663825
		S9_4314078
		S3_69484886
		S4_42875581
		S8_4935013
		S3_54099842
		S4_48336298
		#N/A
		S6_54795959
		S8_37963225
		S8_9934255
		S9_4741927
		S7_7936846
		S6_36459114
		S2_9601680
		S2_60529333
		S5_7351330
		S5_63408382
		S2_59737804
		S3_52176603
		S3_3776203
		S6_41984865
		S1_60816163
		S6_53240818
		S1_79155154
		S9_4072641

Catogoery	Total	SNPs
		S2_64335676
		S1_74349803
		S7_7731692
		S1_1215475
		S2_58290736
		S6_52635472
		S5_2098959
		S5_67727280
		S9_4067506
		S2_61444394
		S6_53439528
		S3_66031395
		S1_58035771
		S6_44551719
		S1_17864120
		S6_48601051
		S5_490635
		S1_56319068
		S2_76993554
		S2_75155374
		S6_38608650
		S4_51070157
		S2_10821483
		S8_60319425
		S5_62646209
		S8_60859209
		S1_68195770
		S3_68716197
		S2_5987373
		S7_62686466
		S4_61191759
		S8_49232006
		S2_62313057
		S1_77431975
		S8_60349072
		S1_71174524
		S7_52285821
		S1_49964333
		S2_75343609
		S6_50696375
		S2_62739821
		S9_3620818
		S6_51466250
		S9_3273276
		S6_57423777
		S5_69942045
		S1_79932548
		S5_3316420
		S9_50744168
		S9_215191
		S9_50596078
		S2_47464184
		S6_58765556
		S3_70711178
		S7_4745781
		S3_52457566
		#N/A
		S2_59648587
		S4_24832713
		S1_2923915
		S2_56953480
		S7_4787458
		S2_76812919
		S3_65872824
		S8_61925059

Catogoery	Total	SNPs
		S5_17987042
		S2_69327373
		S9_51890201
		S1_49433063
		S6_47762272
		S8_56501482
		S2_68922167
		S1_69096006
		S4_26025596
		S7_64104285
		S7_58153803
		S7_55763207
		S1_79932546
		S6_49768862
		S2_56301864
		S7_54578827
		S6_5574686
		S1_59699610
		S7_492506
		S4_2607123
		S9_11642223
		S8_61559340
		S2_71556735
		S2_5489087
		S6_53681755
		S6_14254986
		S4_49133916
		S4_4321251
		S9_55551710
		S4_67087271
		S4_50759136
		S6_58812599
		S1_60758077
		S1_59444145
		S2_73693934
		S2_7055330
		S3_17761838
		S2_10816904
		S1_76020314
		S3_70121219
		S3_45567833
		S3_811453
		S1_57109942
		S9_55004992
		S2_59247242
		S1_76635130
		S3_5625848
		S2_77540791
		S2_12326332
		S3_3770071
		S1_3363021
		S3_63091775
		S9_8111084
		S8_10085603
		S1_80077477
		S1_65259173
		S1_79965480
		S1_7225960
		S2_59791360
		S2_75684413
		S2_5830404
		S1_3248504
		S1_8480630
		S1_72563993
		S3_5458619

Catogoery	Total	SNPs
		S8_61922666
		S1_10689282
		S7_6984858
		S1_71838787
		S8_61980186
		S3_58325374
		S1_46165392
		S3_70711180
		S5_4509043
		S8_54820017
		S9_3026702
		S8_1698709
		S9_58576044
		S9_52028454
		S1_19017760
		S3_69847068
		S7_2771680
		S5_3055229
		S1_67679848
		S1_63345894
		S2_14168121
		S1_4086631
		S2_76706601
		S7_64554806
		S2_16850756
		S2_75672236
		S4_12408494
		S8_60693618
		S1_11205598
		S2_16622844
		S3_52046069
		S4_8918638
		S9_2661234
		S6_54462516
		S7_2907663
		S7_57691662
		S1_73675750
		S2_72264307
		S8_61921415
		S5_8068931
		S9_49346968
		S1_56982725
		S1_67864308
		S1_6131258
		S7_62832449
		S3_46175400
		S4_34754801
		S4_2837367
		S2_63218583
		S8_60362316
		S5_69822761
		S7_9237408
		S1_78005275
		S3_65425680
		S6_44752164
		S1_63458031
		S4_34646980
		S2_10289213
		S1_12922235
		S7_64737938
		S1_16415210
		S1_6336225
		S2_61427947
		S9_8377101
		S4_67082963

Catogoery	Total	SNPs
		S2_68005956
		S2_75975462
		S2_75510745
		S4_1193644
		S3_64337681
		S6_58178558
		S4_45750177
		S5_10052206
		S2_2488279
		S2_68991698
		S3_70121135
		S5_61776064
		S2_41437717
		S2_60448656
		S4_13027368
		S6_46735089
		S1_49363700
		S2_73694517
		S1_66791926
		S1_60184260
		S8_57571758
		S3_74004036
		S3_56869304
		S1_74305021
		S3_5202389
		S3_45627149
		S3_61017872
		S7_8905192
		S2_56105318
		S8_61711352
		S3_60614849
		S7_64554804
		S3_73319016
		S9_54094871
		S1_16805267
		S4_50759109
		S3_54957318
		S9_52932646
		S9_55071852
		S4_8777092
		S1_66310316
		S2_1031112
		S7_38853688
		S8_56815751
		S2_59737460
		S9_3063477
		S8_282509
		S7_2570178
		S5_20276604
		S1_59562687
		S1_72724385
		S5_56426784
		S1_12758664
		S2_40078287
		S6_45848079
		S9_54086121
		S2_52306755
		S1_79195058
		S7_3642286
		S8_15221200
		S2_10753312
		S5_62755992
		S4_3886747
		S7_9909545
		S4_4007315

Catogoery	Total	SNPs
		S2_60529380
		S8_1682462
		S4_54131995
		S2_58850684
		S1_57110287
		S8_1699029
		S9_51068866
		S4_48385682
		S6_54462516
		S8_49668148
		S3_61525149
		S5_2966607
		S7_10970451
		S2_19727177
		S6_6448056
		S2_75613716
		S4_2032167
		S3_68836250
		S3_50690630
		S2_3967057
		S4_11132821
		S2_55048579
		S1_2989623
		S5_15770651
		S1_8138446
		S1_72636869
		S3_55005182
		S1_59970646
		S1_8984791
		S4_12350799
		S1_10876477
		S8_51033659
		S3_62562052
		S6_47420129
		S5_15770219
		S6_54403109
		S1_49273728
		S1_68787903
		S8_59643429
		S1_73871939
		S1_12945080
		S6_16165137
		S2_66605161
		S2_7683145
		S3_5130542
		S3_61852114
		S8_53262247
		S7_2413924
		S2_45423790
		S8_56428235
		S7_52070571
		S6_49860668
		S6_48041575
		S6_41827749
		S7_54812556
		S8_55930254
		S1_71754916
		S8_4310384
		S6_45640375
		S1_5800574
		S3_65353632
		S5_66622366
		S2_27525007
		S1_52755760
		S2_64072287

Catogoery	Total	SNPs
		S9_2931120
		S2_18494608
		S3_68836234
		S4_3738465
		S8_223200
		S9_49280775
		S2_59661948
		S1_9002323
		S2_56332752
		S3_67966296
		S1_60697366
		S5_69905935
		S2_56069838
		S4_12354578
		S5_12146522
		S7_2321500
		S1_58165617
		S1_52089009
		S6_53582921
		S1_20685822
		S2_71522961
		S2_65254215
		S5_66052168
		S5_15655416
		S5_14150680
		S6_51225330
		S7_57678522
		S2_45678376
		S1_79576172
		S3_71574612
		S5_1021210
		S9_50757859
		S9_5464980
		S5_1966807
		S2_67195932
		S4_5065233
		S4_6520529
		S6_58759812
		S1_130563
		S6_37400416
		S4_34738206
		S8_3264581
		S7_2771879
		S2_59538534
		S5_12990689
		S2_63162315
		S8_4678937
		S2_66842280
		S1_62567647
		S8_3173718
		S3_42058238
		S7_7731684
		S6_42211078
		S8_3347577
		S9_55046675
		S5_3230485
		S3_70976224
		S1_73681263
		S1_17105539
		S1_69230705
		S3_65332790
		S1_29008208
		S1_13832003
		S6_53638846
		S2_12744926

Catogoery	Total	SNPs
		S2_60098712
		S2_19488887
		S3_69630915
		S2_3374523
		S7_9795549
		S2_61802674
		S5_11792650
		S6_3543611
		S2_63676093
		S3_13445824
		S1_4143996
		S7_64102150
		S6_56532366
		S2_6178216
		S3_69730929
		S1_7639593
		S8_2178414
		S3_58281920
		S3_70953040
		S6_52635361
		S4_3776398
		S1_24472007
		S3_66671225
		S4_56522570
		S2_4726282
		S3_70256135
		S1_21465989
		S9_2661073
		S5_11615614
		S4_16500155
		S7_57847730
		S2_75891954
		S4_402720
		S7_59944757
		S1_2908990
		S5_12857247
		S3_58246391
		S2_11782684
		S8_60548703
		S5_8752244
		S1_15798036
		S4_67087250
		S6_46460146
		S5_2935686
		S8_17009240
		S7_1488727
		S1_63860025
		S9_57771040
		S9_54094854
		S3_57954356
		S3_64927118
		S1_28214554
		S4_7273386
		S2_8890438
		S1_20013380
		S1_10286632
		S2_73693655
		S3_71379571
		S2_8050601
		S4_62898203
		S9_50296453
		S7_490140
		S7_1488838
		S5_63235219
		S3_53480797

Catogoery	Total	SNPs
		S8_1965361
		S2_57862881
		S4_56610937
		S1_60776180
		S6_53919471
		S1_65255750
		S9_4249153
		S2_59878112
		S3_57493338
		S3_2022904
		S4_39239647
		S8_43078289
		S2_4147833
		S4_12105330
		S1_61730044
		S2_60085398
		S4_6182402
		S8_5440991
		S1_71371527
		S7_62804550
		S1_80088274
		S1_72690855
		S3_65607075
		S2_1475057
		S1_58160395
		S4_813984
		S1_10116725
		S1_80002129
		S2_65792383
		S4_50569568
		S1_70286215
		S7_64737915
		S8_4716289
		S8_5156084
		S1_6785864
		S6_1307009
		S2_55476984
		S3_45569205
		S3_68919630
		S7_57835167
		S2_61949904
		S2_12683284
		S4_52381447
		S1_79438080
		S1_65740374
		S2_57493056
		S3_71412128
		S3_70794225
		S4_2284036
		S9_48996475
		S5_1676742
		S7_54757327
		S9_1010998
		S9_8213254
		S6_51434154
		S5_67670585
		S5_63682605
		S2_76835075
		S9_49350932
		S2_65219191
		S1_53202127
		S6_49718512
		S1_7228680
		S8_49204365
		S8_60931612

Catogoery	Total	SNPs
		S8_1952152
		S2_8263802
		S1_6831689
		S2_1032873
		S1_18035352
		S1_14515491
		S8_31558572
		S1_60849719
		S4_11121854
		S2_5685711
		S6_31579667
		S1_56681113
		S7_52636218
		S2_18758067
		S2_54254795
		S3_6278964
		S2_41116469
		S7_52292168
		S2_12796313
		S1_60778176
		S4_542753
		S6_51865304
		S3_67762397
		S9_40978350
		S5_11514096
		S1_8973951
		S3_5390781
		S2_58331834
		S3_5626201
		S2_73675571
		S4_3797177
		S6_53622974
		S1_18046915
		S1_14515170
		S3_68263803
		S6_31055012
		S5_23824094
		S1_63221551
		S2_68821224
		S6_53583032
		S1_79775166
		S5_67767178
		S3_74210043
		S1_59984410
		S3_55904135
		S6_53160286
		S5_15885719
		S2_4150257
		S1_53853728
		S4_6615700
		S7_1163355
		S7_1222834
		S6_17437405
		S5_9778321
		S2_64382571
		S6_49248215
		S2_12746315
		S4_40568279
		S3_66584119
		S8_4542281
		S1_65908296
		S1_63711688
		S5_2935750
		S1_80077472
		S2_7314941

Catogoery	Total	SNPs
		S5_12084547
		S2_75509979
		S5_51359674
		S4_6415489
		S1_64126481
		S3_59492421
		S9_1188069
		S2_68059555
		S5_11615619
		S8_57596411
		S4_40569381
		S6_47882324
		S7_15931278
		S5_63909592
		S9_55969251
		S6_51403975
		S4_47808745
		S1_59471506
		S2_56077481
		S1_11110383
		S1_9068974
		S9_51417453
		S2_6850385
		S3_527623
		S1_80207929
		S4_34266542
		S6_41350454
		S9_53860362
		S7_63318837
		S8_60382399
		S1_16552941
		S2_7878206
		S5_10814630
		S2_75783713
		S3_65364425
		S4_61260660
		S2_68827581
		S5_66405096
		S8_18551726
		S9_54870485
		S3_67154243
		S2_16391856
		S8_59214615
		S4_8057921
		S1_8022449
		S2_75688222
		S2_7792273
		S2_61695002
		S7_53162737
		S4_7538598
		S2_10327183
		S1_73551459
		S6_7198912
		S5_41687207
		S9_58906442
		S3_73934789
		S9_52479970
		S6_50817375
		S8_5157405
		S1_57837205
		S6_45274073
		S1_71839110
		S9_1237639
		S2_70921952
		S8_2271667

Catogoery	Total	SNPs
		S2_56440766
		S7_62194054
		S6_49477996
		S7_56997702
		S2_7731891
		S4_43252608
		S5_5838509
		S2_76491692
		S5_62919950
		S3_69485296
		S2_4273634
		S9_52891686
		S7_64024316
		S9_51522121
		S2_66291839
		S7_646994
		S2_12688054
		S6_51180115
		S2_56249041
		S2_74736982
		S3_71926635
		S1_56994820
		S9_55005002
		S3_57882259
		S4_18894234
		S3_53586526
		S8_55059607
		S8_3363349
		S6_56759309
		S2_75804561
		S2_63146949
		S9_42020474
		S2_6751691
		S2_57452954
		S1_22275498
		S5_42766706
		S9_10747726
		S9_4743769
		S4_44730247
		S3_20398599
		S5_63499312
		S2_6755757
		S1_76962283
		S7_54796980
		S5_66956048
		S3_50993812
		S3_73926843
		S7_57573963
		S3_69633709
		S5_69794461
		S1_66650943
		S3_72144463
		S6_41984844
		S2_77599222
		S2_9516937
		S1_68301249
		S1_72586847
		S4_54102694
		S1_66791916
		S3_57267475
		S2_14236540
		S2_75150870
		S7_2713711
		S3_63114143
		S2_6232437

Catogoery	Total	SNPs
		S4_52878575
		S9_51887444
		S1_79208565
		S1_63711692
		S1_25887594
		S4_2150999
		S1_9929639
		S9_53230199
		S9_11153304
		S7_17140457
		S6_50656541
		S1_63711701
		S3_73144781
		S4_4160683
		S9_58114228
		S2_8262806
		S3_51998709
		S8_49310940
		S3_73301754
		S6_32685399
		S1_54021171
		S4_54204338
		S5_1610018
		S2_60166324
		S1_24741104
		S6_29942002
		S2_6204331
		S4_50357692
		S1_73045662
		S8_4716255
		S8_3224907
		S1_8998684
		S4_6257765
		S1_51003639
		S2_10527131
		S4_13591642
		S7_40999377
		#N/A
		S5_18255728
		S9_4320288
		S2_4366810
		S7_51224549
		S1_78818482
		S1_18035109
		S3_64452220
		S5_51749742
		S1_54392123
		S4_13858546
		S5_5251676
		S7_58344828
		S5_62105133
		S2_65407931
		S8_60170401
		S4_3717314
		S1_71688849
		S2_61841928
		S6_53933708
		S3_34695638
		S3_70808997
		S3_74143090
		S2_60289478
		S4_33504378
		S5_67645764
		S7_41129828
		S3_1964490

Catogoery	Total	SNPs
		S1_46823915
		S3_6436600
		S7_55762989
		S7_6982494
		S3_6389180
		S3_61931996
		S3_5640602
		S7_10020959
		S2_2257592
		S4_24735556
		S1_73581174
		S1_6777461
		S1_21735688
		S7_63022632
		S5_9373075
		S1_58293482
		S3_61398774
		S1_78818474
		S2_66280872
		S3_60484050
		S4_5107246
		S7_4559738
		S8_55274629
		S4_16467066
		S9_58701441
		S2_8050306
		S9_1121474
		S6_26214034
		S9_5862586
		S1_21735703
		S1_11550678
		S2_58682745
		S5_63590157
		S4_3919990
		S4_7327011
		S5_8848240
		S9_3551764
		S4_50769444
		S9_5829134
		S5_7188315
		S5_12744712
		S8_60911980
		S5_69822719
		S1_64392722
		S3_57375566
		S8_2635773
		S1_79932484
		S1_7856720
		S1_63714590
		S7_62030134
		S5_6065161
		S6_60754171
		S5_63463771
		S3_53336022
		S4_8587461
		S3_5521169
		S1_50929763
		S8_56115055
		S6_52777861
		S4_50273636
		S8_9589208
		S6_55202549
		S8_58700466
		S8_50831055
		S2_61411701

Catogoery	Total	SNPs
		S5_4276198
		S3_56659017
		S3_63065598
		S4_49816090
		S2_4554981
		S1_74215847
		S2_63216535
		S1_59471449
		S2_2235731
		S5_50603258
		S8_49231974
		S1_66898497
		S2_8176173
		S4_10304304
		S1_22682507
		S3_1881422
		S9_1121432
		S5_3230240
		S1_67073827
		S6_60908647
		S4_56437060
		S2_12281263
		S7_1799592
		S7_63060601
		S1_65680792
		S7_52641865
		S2_76559708
		S8_56332073
		S1_23891846
		S3_71678237
		S6_13695604
		S2_59247353
		S3_67023597
		S3_70248349
		S5_1761313
		S6_51306866
		S1_49477391
		S3_5606510
		S4_46537718
		S5_1456057
		S3_68382271
		S7_62123296
		S3_62067145
		S3_6242252
		S2_66299352
		S3_69824236
		S6_47674369
		S1_9809403
		S3_72071851
		S9_52932650
		S1_1762148
		S1_11161127
		S2_59247282
		S8_4402322
		S6_45674967
		S1_30656583
		S6_38194239
		S6_46381107
		S2_61913376
		S9_5273142
		S1_67620431
		S3_61913537
		S4_27105275
		S7_61501047
		S8_59746772

Catogoery	Total	SNPs
		S8_9742760
		S8_61768103
		S4_8017373
		S7_54775506
		S1_56319317
		S8_56451439
		S1_60414041
		S1_66650751
		S2_75158462
		S1_8154246
		S4_53344054
		S2_65067965
		S2_4748202
		S9_59303007
		S3_72311101
		S2_68829234
		S3_5620550
		S5_19911062
		S2_23357734
		S3_74174559
		S2_58016446
		S3_73289925
		S7_58155367
		S3_65869610
		S3_70691352
		S8_2624370
		S2_4554928
		S7_60299061
		S6_53726912
		S1_1346265
		S1_58244106
		S1_68895463
		S8_55962892
		S3_52139909
		S9_511984
		S6_47625661
		S1_6034150
		S2_63670796
		S3_60246037
		S1_17276943
		S2_75768461
		S1_6466518
		S2_14269430
		S6_56333519
		S1_71946553
		S5_62509870
		S3_54820298
		S1_77100627
		S2_60577627
		S1_64159269
		S2_65323948
		S3_51514481
		S4_66147750
		S2_43983146
		S3_53583416
		S9_51173602
		S3_70388696
		S2_58845801
		S1_7547390
		S3_72789935
		S2_63426477
		S1_77325670
		S8_2374787
		S8_3457012
		S1_3053310

Catogoery	Total	SNPs
		S2_4554997
		S2_3785442
		S9_51545847
		S4_62270457
		S8_56847152
		S3_57896592
		S1_68089858
		S5_1729055
		S4_15693747
		S5_1698596
		S5_5213370
		S4_59510434
		S8_55598234
		S1_77914224
		S2_61368340
		S3_4879606
		S3_69734212
		S1_78989192
		S1_14483592
		S9_5861348
		S6_46723625
		S5_68170927
		S9_57611543
		S7_54775576
		S2_67732479
		S2_6178220
		S6_50704203
		S5_66445901
		S8_61767749
		S5_62355361
		S9_58679974
		S1_55992039
		S2_8928118
		S8_33535024
		S5_69893230
		S8_60532006
		S4_21754983
		S8_59968913
		S5_1375789
		S2_61833720
		S7_62237252
		S6_49906555
		S2_3776189
		S7_58333469
		S6_47940846
		S2_12754196
		S5_69847941
		S2_68830007
		S3_70120860
		S5_59317219
		S5_62505736
		S3_69488661
		S1_52256402
		S1_8644851
		S4_2002539
		S5_9851537
		S2_12683310
		S3_5659695
		S1_77861605
		S9_52469358
		S8_51715051
		S7_8881711
		S1_67547462
		S2_65296480
		S7_58333586

Catogoery	Total	SNPs
		S7_8824237
		S7_55763248
		S9_58723856
		S2_63584030
		S5_2668979
		S6_41220194
		S5_1481115
		S4_62270568
		S7_58297925
		S1_20397094
		S2_10266234
		S2_57591454
		S4_4896259
		S1_79877675
		S2_58604413
		S1_1544645
		S2_73414891
		S1_62245524
		S3_34260272
		S3_61017873
		S6_50583868
		S4_66243129
		S9_44339498
		S9_9098016
		S5_59447657
		S7_61403719
		S8_4934940
		S7_8910652
		S1_49363710
		S3_70510291
		S8_42931158
		S8_47685285
		S2_5570738
		S1_59338088
		S9_53299997
		S7_58345249
		S7_54785519
		S3_50689701
		S4_52875542
		S1_55830818
		S1_71822756
		S3_52822341
		S9_52077318
		S1_59892847
		S8_60937156
		S6_47893347
		S2_1510537
		S8_3363373
		S1_26853669
		S2_40389277
		S4_4321238
		S5_69989072
		S2_59790534
		S3_68563581
		S4_53934251
		S4_699735
		S1_22034623
		S5_69985222
		S8_2325864
		S7_52290733
		S1_9485124
		S1_75505621
		S3_5014234
		S2_10525740
		S3_73356722

Catogoery	Total	SNPs
		S2_77607000
		S9_51149487
		S6_15065603
		S2_1510482
		S8_58489005
		S2_7324196
		S6_42344102
		S7_63639765
		S2_67761344
		S3_73984384
		S2_67370659
		S3_70598784
		S8_47529492
		S6_46674681
		S2_16294972
		S3_6277274
		S2_69020528
		S4_41634262
		S1_7343307
		S3_70296850
		S8_5061409
		S1_9918876
		S7_64211704
		S2_62695655
		S8_43503763
		S1_74119552
		S6_47700025
		S2_13170990
		S3_69879206
		S8_47521893
		S8_3369796
		S9_50751625
		S5_5114771
		S2_56431467
		S2_60970193
		S6_50574074
		S1_6670021
		S3_5130557
		S9_3904678
		S8_57039811
		S3_6276825
		S1_21823590
		S3_71894926
		S1_71822749
		S3_19382499
		S7_17425482
		S1_15324261
		S7_58749815
		S1_56982721
		S7_39941591
		S1_31205593
		S8_60452253
		S5_32133
		S6_45102937
		S4_1086660
		S8_32609445
		S9_1241131
		S5_394560
		S2_61445045
		S6_48109603
		S8_49628999
		S1_13892002
		S8_5160841
		S2_73624793
		S7_8583728

Catogoery	Total	SNPs
		S9_54006387
		S1_57707471
		S1_2912082
		S2_17943594
		S2_16380629
		S5_7326415
		S9_9038531
		S7_6452669
		S9_52092816
		S2_65504229
		S2_64091958
		S8_5160745
		S3_52686855
		S9_53945911
		S6_1316376
		S6_47626934
		S3_72827593
		S1_7883656
		S4_58197122
		S2_4109298
		S8_56847139
		S7_574205
		S1_59878186
		S3_73362985
		S4_53265378
		S5_68207899
		S9_26781329
		S5_62642212
		S2_6396283
		S2_59247251
		S9_1022269
		#N/A
		S2_17332952
		S7_39231844
		S1_21236561
		S3_57954504
		S2_481677
		S9_3302218
		S4_34703032
		S6_41417370
		S7_54776950
		S5_66441689
		S1_79859827
		S1_50464435
		S3_56448616
		S2_75944717
		S6_41827678
		S6_51477217
		S2_11559900
		S9_53255125
		S8_5441957
		S5_69780337
		S3_6248577
		S4_7293716
		S2_64476803
		S3_4955743
		S4_14926828
		S7_40589250
		S1_53853890
		S7_62476164
		S3_20545482
		S3_54263378
		S6_47643430
		S1_8819071
		S8_61951473

Catogoery	Total	SNPs
		S9_51890189
		S4_7081484
		S1_75619537
		S6_26230280
		S3_73934903
		S2_58025289
		S6_3876509
		S5_62417159
		S1_130556
		S1_76491858
		S1_66140059
		S6_47606239
		S7_55434353
		S3_70839403
		S6_46801672
		S4_53281390
		S3_6748205
		S9_40807190
		S1_73662919
		S9_1789976
		S1_57102001
		S3_72228688
		S4_1359235
		S1_18172701
		S8_32613423
		S2_4109237
		S8_1840293
		S7_53990651
		S3_73765883
		S2_63077538
		S3_70375989
		S9_44198590
		S5_10490416
		S2_6526502
		S9_1646663
		S6_3201106
		S8_60931533
		S9_59378887
		S3_5603285
		S7_62804087
		S5_13394021
		S8_55707601
		S6_53194271
		S1_6678791
		S2_6015442
		S6_53558101
		S2_11097374
		S1_12932582
		S1_19341851
		S8_1876829
		S2_12434683
		S6_58851692
		S2_67842618
		S4_66239264
		S7_59752054
		S5_2693282
		S2_10329069
		S3_16168485
		S1_77343719
		S6_47601938
		S1_68831729
		S6_60926386
		S1_54569271
		S2_63476778
		S8_9354008

Catogoery	Total	SNPs
		S9_53933960
		S2_57607891
		S4_5947129
		S7_52290731
		S5_23554349
		S1_7810912
		S6_5806993
		S7_58295696
		S5_61161520
		S4_19696377
		S1_19349034
		S1_73111735
		S7_63076827
		S2_56870785
		S2_2675718
		S3_73506875
		S6_41925193
		S8_49573271
		S9_54111768
		S5_54661191
		S6_14683531
		S3_3757328
		S4_34291130
		S2_10525635
		S8_11325032
		S8_58151668
		S9_57907179
		S9_57670659
		S5_62754800
		S5_1583422
		S2_69858494
		S3_69764576
		S4_2650399
		S7_2559101
		S4_51492917
		S9_132613
		S2_12030815
		S3_72504729
		S3_71062035
		S4_67915656
		S7_54283697
		S6_47759975
		S3_64401496
		S8_57753842
		S5_5827141
		S3_61991478
		S1_79155140
		S4_6462490
		S4_8086336
		S1_66832479
		S1_73735445
		S2_383017
		S8_57441521
		S8_6158320
		S1_66827677
		S9_41543593
		S3_4576524
		S3_50412448
		S8_282502
		S3_14149923
		S9_52985983
		S6_53063905
		S4_1609547
		S3_13445822
		S1_9673646

Catogoery	Total	SNPs
		S1_9153558
		S5_61748996
		S4_61175432
		S6_31105440
		S1_6328095
		S1_65279050
		S2_61161419
		S1_8138443
		S5_58447864
		S4_37656031
		S1_46554134
		S1_64902343
		S1_2923879
		S8_31919595
		S5_3230485
		S6_46383729
		S2_74775075
		S2_63996161
		S5_66191097
		S3_53583417
		S3_73823762
		S5_5041456
		S2_12434779
		S2_66280870
		S2_68691274
		S3_62419921
		S2_11706104
		S1_61720424
		S3_70208131
		S2_56916040
		S1_63253438
		S4_9016555
		S5_1701005
		S6_49440336
		S1_56568047
		S3_570698
		S9_4231053
		S8_17494981
		S1_66113456
		S8_60912230
		S1_74291838
		S3_635012
		S3_74118632
		S8_3218250
		S2_63520543
		S6_45079160
		S2_17689479
		S3_65332778
		S2_2758326
		S9_54094900
		S1_19138572
		S2_65466848
		S1_59168128
		S9_11655997
		S5_59372532
		S2_4561670
		S6_40434377
		S9_11317066
		S9_49058689
		S8_60557451
		S5_61161548
		S7_58551454
		S7_65135510
		S9_53867595
		S3_69937429

Catogoery	Total	SNPs
		S9_1111032
		S3_61327884
		S1_60348989
		S9_6654273
		S5_9181000
		S1_7656296
		S6_53681790
		S9_10176659
		S9_972217
		S1_3156778
		S5_1249735
		S4_49329145
		S1_58593618
		S4_53399886
		S1_61978776
		S4_5614621
		S5_2601058
		S3_68015768
		S6_49220114
		S2_73675543
		S9_2327763
		S5_4241603
		S1_59114892
		S1_10876549
		S8_57429572
		S6_53401974
		S1_20207379
		S9_50217620
		S3_54404366
		S2_64287859
		S4_52612796
		S5_11792507
		S1_75861015
		S6_19399532
		S2_65920905
		S4_54132000
		S4_11240599
		S1_78794274
		S9_57247267
		S2_4282100
		S2_56301780
		S3_63115583
		S3_52430651
		S5_18541785
		S1_24133065
		S8_57588846
		S5_71216694
		S4_1111865
		S4_2762652
		S1_15411958
		S1_6335858
		S1_58165615
		S5_63298355
		S6_49254325
		S6_40940574
		S7_38617372
		S2_63670779
		S6_18788759
		S2_42023593
		S1_11197153
		S7_54787415
		S9_3156387
		S7_54811626
		S2_7249963
		S8_58700310

Catogoery	Total	SNPs
		S8_35008391
		S3_73765874
		S3_73337296
		S6_42023423
		S5_61217547
		S2_9816063
		S8_60373820
		S5_6101719
		S1_16395763
		S3_72851648
		S6_41350746
		S5_54661174
		S8_51033551
		S7_962862
		S1_77092828
		S4_10291302
		S6_17521556
		S4_67970848
		S6_53215457
		S4_66125723
		S4_54869312
		S3_73627586
		S7_1223274
		S4_51082362
		S6_53942654
		S2_61224566
		S8_3579334
		S4_816722
		S2_6092437
		S1_53472226
		S6_45147005
		S5_63307771
		S1_79161785
		S4_54427148
		S1_6034165
		S2_76621453
		S6_38113521
		S8_30197303
		S3_58220457
		S7_1488727
		S9_937983
		S2_4481946
		S2_61029321
		S5_2971416
		S3_72321756
		S6_1345307
		S8_60938237
		S5_3621112
		S6_44752211
		S6_5905497
		S3_56868575
		S8_32289907
		S4_66042980
		S2_74755655
		S4_50670396
		S1_63212216
		S3_47885493
		S2_39949383
		S9_7314288
		S7_6741174
		S2_61739830
		S8_5320757
		S2_65226823
		S3_52417875
		S1_56381395

Catogoery	Total	SNPs
		S3_70348699
		S6_52000455
		S4_874899
		S3_57267461
		S3_61913552
		S9_55626962
		S6_54465062
		S5_1224984
		S8_59672949
		S7_6672070
		S5_61210446
		S2_45094639
		S6_58535286
		S2_10826237
		S1_5703848
		S6_45647679
		S8_4456699
		S4_49489452
		S2_61551452
		S3_73506862
		S6_921590
		S7_65343697
		S3_4576496
		S3_57237990
		S5_70045218
		S2_63220919
		S3_56674527
		S6_33978630
		S1_28678201
		S7_54697811
		S5_1645233
		S9_54317249
		S9_10361537
		S2_15193128
		S2_68874617
		S1_64272984
		S3_59472401
		S2_11765739
		S3_2379501
		S3_6748318
		S5_22705785
		S8_4174346
		S5_61112740
		S5_63419166
		S2_59247274
		S6_58878792
		S2_74073524
		S5_68147786
		S1_59878209
		S1_54031706
		S2_73029921
		S7_64740357
		S5_6826517
		S1_12935953
		S7_64515391
		S2_41116481
		S7_8018586
		S6_58550619
		S2_45966888
		S6_47698020
		S3_450959
		S1_56224795
		S5_68349955
		S3_4606853
		S7_58534234

Catogoery	Total	SNPs
		S2_7829318
		S2_66010387
		S7_62611661
		S3_69263642
		S9_4037881
		S8_60393332
		S8_6158498
		S2_54244662
		S3_5630415
		S5_6991478
		S1_19426148
		S1_59655369
		S3_70902108
		S2_10269498
		S2_4554990
		S1_2464833
		S7_7347039
		S6_45540277
		S9_41580013
		S8_33553188
		S1_79585267
		S1_7957613
		S2_10290282
		S2_9219487
		S3_5130760
		S2_18779230
		S5_63506015
		S7_59024679
		S1_61639736
		S9_6671800
		S7_46938730
		S3_48021119
		S5_65945157
		S1_67610048
		S7_12470490
		S3_1969734
		S4_39035293
		S7_64100833
		S8_57310763
		S8_5976486
		S1_57003417
		S2_62484776
		S4_39654510
		S6_1067414
		S3_2000516
		S9_7314162
		S6_41600647
		S1_49024661
		S3_65987887
		S5_11514090
		S7_64858916
		S3_46121064
		S7_39233697
		S2_57035643
		S2_1953722
		S1_57110288
		S3_66606750
		S5_14900463
		S8_55615061
		S2_12509252
		S5_11063066
		S7_39531408
		S1_72238439
		S1_59792257
		S1_62915586

Catogoery	Total	SNPs
		S4_50587650
		S2_2888144
		S9_6372029
		S1_77674469
		S2_16622844
		S6_50647247
		S7_537132
		S1_72737995
		S7_6671226
		S4_3253625
		S8_56797548
		S3_73014943
		S9_99251
		S2_60678969
		S6_1668312
		S8_4717686
		S7_62494213
		S2_9672839
		S2_73925314
		S9_2887538
		S7_2743164
		S1_58096656
		S3_5563338
		S2_47634661
		S3_4576508
		S7_63683510
		S7_64926518
		S3_2480001
		S1_59359324
		S3_3060250
		S4_41454678
		S5_22028744
		S2_10506597
		S1_25619145
		S1_60837334
		S2_60970341
		S1_59562690
		S2_69829403
		S6_56513262
		S3_73925052
		S4_3924935
		S1_10949858
		S1_78161397
		S1_17429781
		S1_19640916
		S8_58676456
		S2_60033251
		S1_67400071
		S8_34601924
		S2_63820970
		S7_890860
		S3_73084464
		S1_9066126
		S8_3048619
		S2_75688223
		S9_54142091
		S2_6036366
		S5_62156689
		S3_73844392
		S1_17585359
		S1_21722341
		S3_50689696
		S8_60351257
		S1_73867697
		S4_14003018

Catogoery	Total	SNPs
		S1_80074599
		S3_763697
		S8_49940293
		S1_12730878
		S3_64873844
		S4_7131977
		S3_20073221
		#N/A
		S7_9517243
		S4_43252635
		S2_69344728
		S2_15331762
		S1_78489401
		S4_1580014
		S1_60823767
		S6_335651
		S7_63639812
		S5_65468104
		S3_65450374
		S2_57607886
		S4_10174007
		S1_12920471
		S8_4491973
		S1_7438342
		S1_6040053
		S1_12945075
		S4_23540849
		S2_7829335
		S3_62065689
		S2_57084258
		S7_16083825
		S3_70018105
		S6_54583563
		S5_63235229
		S1_58913438
		S2_7736121
		S8_55936311
		S2_75804441
		S2_31659186
		S7_40828113
		S6_49330742
		S5_3039780
		S5_61128777
		S4_61168892
		S9_52207519
		S6_6658005
		S3_6277278
		S9_53867596
		S1_10081307
		S3_18261078
		S9_54094900
		S5_11706309
		S1_62829520
		S1_59557502
		S9_7286524
		S7_60709257
		S1_65484296
		S1_66554365
		S3_73184466
		S4_12412362
		S2_75944724
		S3_72225862
		S4_2284035
		S4_66120974
		S7_57557650

Catogoery	Total	SNPs
		S2_60407380
		S3_72215324
		S4_21050206
		S2_54468087
		S8_61636555
		S1_11453226
		S2_56332752
		S2_60591418
		S2_61688151
		S6_8865355
		S9_51666268
		S8_62073103
		S8_50206686
		S2_65329906
		S1_16455471
		S1_63350656
		S1_59168697
		S7_61527389
		S6_1587000
		S9_54825464
		S2_18316316
		S2_14467594
		S2_45862613
		S6_54759106
		S3_68380599
		S2_75331083
		S9_54131197
		S7_60172879
		S3_73984345
		S4_2076439
		S6_52704230
		S1_16552929
		S2_61642473
		S9_1041166
		S8_58488860
		S4_1010651
		S7_2625909
		S9_5175899
		S4_6414089
		S6_45102955
		S3_55024365
		S5_4616881
		S1_56149077
		S9_41577335
		S7_63553910
		S4_62880549
		S7_8116333
		S5_32184
		S1_17145004
		S1_9578546
		S6_54583411
		S1_18002155
		S4_27411073
		S6_4445190
		S9_214852
		S4_13491113
		S8_58744218
		S1_11192692
		S3_73241354
		S6_58878804
		S9_57060473
		S1_4118123
		S3_69421195
		S1_2607592
		S3_51534602

Catogoery	Total	SNPs
		S2_11097359
		S6_54752858
		S7_64100640
		S9_55626943
		S9_2661393
		S1_79484339
		S5_61210449
		S5_2693097
		S2_1032809
		S3_57362898
		S1_3625723
		S5_70791593
		S4_56610936
		S3_58848295
		S2_68531047
		S2_76427931
		S2_8879057
		S2_60985657
		S4_13959925
		S1_64159638
		S7_62920632
		S5_67938440
		S2_69151559
		S3_71141263
		S9_53064747
		S9_59301045
		S6_47901258
		S8_6185570
		S5_41687210
		S9_10666774
		S5_3620282
		S2_76513418
		S7_9515626
		S4_1301357
		S7_425717
		S2_46939184
		S2_58288533
		S1_59700201
		S6_51864787
		S7_2796074
		S3_62538142
		S6_48605537
		S6_47762255
		S1_72372296
		S1_19382327
		S3_70519390
		S8_55498816
		S6_58599825
		S4_55592509
		S1_8881269
		S7_42430065
		S7_5932086
		S5_11615609
		S6_50925320
		S5_66386667
		S1_47689764
		S9_41578315
		S5_3818322
		S5_66097562
		S1_8819600
		S1_57379019
		S7_1839421
		S4_1778418
		S1_49924399
		S8_33188143

Catogoery	Total	SNPs
		S2_65031802
		S2_49922059
		S9_58738310
		S7_15415181
		S5_6769933
		S4_14354186
		S9_48860346
		S2_66175182
		S8_44868262
		S1_66821751
		S2_10525747
		S2_75649783
		S9_1110855
		S2_4555070
		S2_68059545
		S7_40498317
		S6_2665911
		S1_27202416
		S1_80030412
		S5_7310298
		S8_55614983
		S8_5259965
		S2_75689611
		S7_18168035
		S2_63675179
		S6_50583824
		S1_79434626
		S8_1341838
		S1_78225771
		S1_9066109
		S3_70640183
		S2_59240058
		S1_13392318
		S9_43534512
		S2_76835074
		S1_10899283
		S3_71247137
		S4_10418349
		S1_9844454
		S2_10897663
		S2_8823331
		S3_73040904
		S2_31813683
		S1_19100029
		S6_47343551
		S8_62000080
		S1_51003635
		S1_16203490
		S5_1922843
		S6_37330040
		S3_6350887
		S9_52806170
		S8_49231995
		S1_50322596
		S5_62493116
		S2_10836510
		S1_57872790
		S2_64465231
		S1_10116653
		S2_75672234
		S1_59698037
		S1_13593118
		S4_7173030
		S6_55685385
		S1_67487335

Catogoery	Total	SNPs
		S2_72265473
		S1_31205595
		S2_60289372
		S7_15931312
		S5_54661069
		S4_2627017
		S2_45423780
		S1_7960197
		S5_4557961
		S3_5644997
		S8_60829753
		S5_66455674
		S3_68492742
		S8_4627372
		S2_9725910
		S4_6465636
		S2_67977310
		S2_11055218
		S1_57882861
		S1_74475284
		S1_4638717
		S2_51561370
		S1_16395807
		S9_53532342
		S1_24623335
		S8_59643418
		S5_11514101
		S6_55684140
		S6_51320635
		S8_61669092
		S3_69485305
		S6_52401555
		S6_49330748
		S5_12445839
		S2_71569855
		S2_34431060
		S1_56295195
		S1_63709859
		S9_49296269
		S6_13915711
		S5_11594238
		S6_52694378
		S2_3809304
		S8_3480372
		S6_26052814
		S3_48916486
		S4_66306775
		S4_6052755
		S9_3913698
		S4_52949046
		S6_29550864
		S3_57896657
		S4_8017356
		S3_1866320
		S3_67910374
		S1_68851110
		S9_33147163
		S2_2753823
		S1_72558548
		S4_52606577
		S1_5949209
		S4_10665219
		S6_42206665
		S6_45197453
		S7_58606388

Catogoery	Total	SNPs
		S1_63866636
		S4_5534886
		S1_64546847
		S3_55142887
		S9_6734290
		S6_45749813
		S1_74992113
		S3_5156657
		S2_73345775
		S4_52436572
		S7_65301503
		S6_35891516
		S2_57607775
		S7_63156116
		S6_40234970
		S2_65882038
		S1_80064969
		S8_337905
		S2_75369037
		S3_56758962
		S9_53202190
		S1_75861013
		S1_16415207
		S1_6166295
		S9_1790011
		S3_56120313
		S3_14145196
		S4_2151638
		S3_59576790
		S1_7648984
		S7_59053500
		S3_621009
		S6_53857093
		S9_8620786
		S8_59762530
		S1_16399094
		S9_4072708
		S2_75791550
		S7_65097645
		S6_44803336
		S2_60530147
		S5_66622971
		S2_65921118
		S1_8975377
		S8_59178507
		S1_2617542
		S8_60914316
		S4_17110470
		S8_58489031
		S1_11127560
		S1_7343400
		S1_28678217
		#N/A
		S5_9695627
		S1_66568091
		S3_53254676
		S3_4609055
		S9_215036
		S1_60760763
		S7_8561593
		S2_56514513
		S1_9807891
		S5_62682854
		S3_72230992
		S2_2521455

Catogoery	Total	SNPs
		S9_53867598
		S2_10525744
		S1_79659910
		S5_62950680
		S1_77362323
		S8_48539249
		S7_10601867
		S1_59824405
		S8_4045225
		S2_60678937
		S3_53290359
		S8_32198727
		S1_74531576
		S4_35757032
		S2_6220930
		S2_72317059
		S7_52328134
		S5_10420977
		S1_11686432
		S9_2744903
		S2_73037436
		S2_41020390
		S7_2445274
		S7_62475284
		S2_6045460
		S5_10882161
		S8_5396867
		S1_57444326
		S5_1167074
		S5_14284051
		S5_58624992
		S2_12452488
		S7_54787488
		S8_38120356
		S4_7277286
		S1_11686417
		S1_57130594
		S1_59539143
		S9_51121324
		S8_60535646
		S7_64061378
		S6_1839009
		S1_8957823
		S8_5140499
		S2_16164509
		S8_55700903
		S2_56870780
		S2_58604317
		S5_1366772
		S6_53931653
		S6_52598622
		S9_58304888
		S7_63319021
		S5_4944951
		S4_5107398
		S1_22664001
		S6_453023
		S5_65523622
		S2_18758078
		S6_41273745
		S5_65930943
		S9_57983395
		S1_54441868
		S7_52290653
		S2_75324570

Catogoery	Total	SNPs
		S1_72898845
		S4_1497826
		S4_11521582
		S1_7868010
		S8_4542298
		S2_5417887
		S8_51822721
		S2_8672943
		S3_55345682
		S1_79361409
		S4_8919563
		S8_32606386
		S1_64479483
		S7_62113560
		S4_55505266
		S7_17079845
		S7_6679582
		S6_55690159
		#N/A
		S9_5027155
		S6_1653085
		S1_7480480
		S5_13134239
		S7_62156978
		S3_1866322
		S5_66681
		S1_7157302
		S3_59558286
		S2_76913338
		S4_2148016
		S8_51865760
		S1_72841958
		S1_46553994
		S9_5744810
		S1_54500148
		S1_67933626
		S3_45886174
		S1_11198825
		S3_67154237
		S1_74119560
		S2_74636782
		S4_17371114
		S2_7883415
		S2_63676099
		S8_14643690
		S1_67679299
		S7_63060604
		S3_57750642
		S4_2597429
		S2_12675931
		S4_3813732
		S4_40220195
		S5_6770019
		S2_70908175
		S6_49768863
		S2_56904578
		S3_73319086
		S2_5497560
		S1_79052080
		S6_53317793
		S1_66784571
		S1_64803267
		S1_8442021
		S6_49447623
		S1_3105890

Catogoery	Total	SNPs
		S2_73693576
		S3_73934266
		S8_56542078
		S2_53154202
		S3_54181146
		S1_66564570
		S8_4682766
		S5_3384254
		S2_41635222
		S5_67495273
		S6_53273379
		S2_44422137
		S8_51323028
		S5_15767954
		S2_54447948
		S2_43778859
		S3_69651292
		S4_1105183
		S8_62097089
		S1_18041462
		S1_14379247
		S2_64350280
		S5_61792602
		S3_73160956
		S9_52554856
		S6_1306505
		S1_30564804
		S2_55092908
		S5_18589880
		S5_9851513
		S2_13177352
		S1_59792117
		S9_51487891
		S5_66093429
		S2_60350052
		#N/A
		S4_19154948
		S3_72968826
		S3_68919627
		S7_59120760
		S5_63230685
		S9_54080778
		S2_10987696
		S1_78834488
		S5_66354573
		S5_62379303
		S5_13388921
		S2_65958949
		S3_64843459
		S9_1188166
		S2_59538097
		S1_68239196
		S9_51466866
		S2_64465164
		S3_57139104
		S5_51346348
		S2_66044602
		S1_59759836
		S7_60002687
		S2_11319041
		S1_59000568
		S1_78818477
		S1_27447669
		S2_44894287
		S1_48779668

Catogoery	Total	SNPs
		S4_12486717
		S8_57478646
		S1_16399190
		S2_63368936
		S6_41584341
		S5_16122898
		S1_66824840
		S4_26502932
		S4_52124164
		S9_44168086
		S1_72690852
		S4_1510611
		S3_5106809
		S2_6239310
		S4_67429468
		S1_13325758
		S8_3369478
		S4_1275251
		S2_10500251
		S3_56051375
		S6_45640369
		S1_19425992
		S9_220872
		S8_42768934
		S1_10408898
		S5_68228905
		S1_3096143
		S1_65680765
		S2_53430335
		S4_13304368
		S1_67487315
		S7_54307705
		S2_3776183
		S7_10069339
		S9_49587678
		S6_47937355
		S3_58281923
		S1_63011656
		S1_10979164
		S3_48591434
		S7_61602789
		S4_5107576
		S1_79165403
		S7_614081
		S4_61181881
		S1_12922223
		S1_67219966
		S7_50724290
		S1_78755153
		S4_47808795
		S6_5624819
		S1_13471116
		S5_6750570
		S5_61780010
		S9_2744858
		S3_58769509
		S2_64287857
		S3_72762492
		S5_22299559
		S3_58315115
		S3_22215848
		S1_7598861
		S2_65280421
		S2_9727785
		S1_26241108

Catogoery	Total	SNPs
		S9_43956720
		S7_62721914
		S3_71794788
		S8_3613896
		S3_2457531
		S4_51265940
		S3_59653276
		S9_50867940
		S1_72373891
		S1_13709165
		S1_24781455
		S9_3620848
		S1_24602896
		S2_67621155
		S1_71839393
		S9_2827097
		S4_7452276
		S1_71605999
		S2_63465054
		S2_2521513
		S2_61742415
		S7_54787411
		S6_2016264
		S2_6785048
		S4_2491202
		S1_8821784
		S9_51890212
		S3_61871239
		S2_76452094
		S3_56274486
		S4_38494454
		S5_61207846
		S3_1654836
		S1_78087739
		S6_50365018
		S2_59822693
		S1_76020317
		S1_62840626
		S3_52182293
		S4_1608063
		S5_5196151
		S8_54784243
		S2_3987755
		S5_62378841
		S1_9921049
		S8_37963191
		S2_11967541
		S2_65352917
		S5_58114031
		S7_60936302
		S6_51865296
		S1_68961427
		S3_58732636
		S3_70247458
		S2_3781160
		S1_16387875
		S1_69096003
		S2_68104970
		S3_2176138
		S2_49330081
		S5_15929075
		S3_51970974
		S1_65673461
		S4_5107066
		S2_4149877

Catogoery	Total	SNPs
		S9_1550727
		S8_51899473
		S2_61068475
		S1_9918960
		S3_69631018
		S6_42183794
		S5_70791604
		S3_55005980
		S9_43444554
		S6_55685309
		S3_16374122
		S1_46653304
		S2_30162543
		S1_56407921
		S1_57417687
		S4_2233506
		S6_51281862
		S3_61310133
		S2_54447947
		S2_73743298
		S3_5617899
		S8_14906598
		S6_51441222
		S3_58381845
		S7_58155355
		S3_56866642
		S1_8899999
		S1_19057299
		S3_5151221
		S4_54072600
		S4_873415
		S5_11706219
		S3_54260959
		S2_67297471
		S6_55708776
		S4_19160948
		S7_64935036
		S2_47253426
		S6_42206656
		S6_48690744
		S9_52796179
		S7_60709294
		S3_63287843
		S3_73363639
		S3_57894801
		S3_70883526
		S3_47220675
		S1_67311310
		S5_59401312
		S1_75662778
		S4_20365964
		S3_6277257
		S3_70818340
		S5_4220062
		S9_52122027
		S1_14438471
		S4_4804002
		S4_49133914
		S7_60023375
		S5_69067857
		S7_3706966
		S7_56660915
		S9_51523714
		S1_57130599
		S9_56933672

Catogoery	Total	SNPs
		S2_72265475
		S4_3931525
		S1_66340798
		S6_40967587
		S1_71371626
		S3_55386142
		S6_53317075
		S9_10234873
		S4_44730246
		S4_13853892
		S5_68742415
		S8_53982125
		S9_53299989
		S3_63142310
		S6_53583313
		S4_66977192
		S1_74538827
		S1_63447974
		S3_66606696
		S3_46856369
		S2_6183664
		S9_51548423
		S2_2644720
		S9_3026704
		S7_52314499
		S6_44836585
		S7_56233115
		S9_58926856
		S9_54111499
		S2_72624751
		S3_4266437
		S9_54956450
		S5_39609702
		S9_58736230
		S4_52411158
		S8_49006898
		S8_5366720
		S4_11117664
		S7_15698340
		S9_50255533
		S5_67558259
		S4_52350719
		S1_63320367
		S1_74542869
		S5_6770018
		S9_53527830
		S1_73064084
		S2_55476983
		S2_1482467
		S1_18853373
		S1_67337147
		S8_49977788
		S9_57212498
		S5_58574553
		S5_69989077
		S7_57847788
		S8_49628776
		S9_3828948
		S1_28678210
		S3_72662134
		S6_50914996
		S8_35580030
		S3_32969740
		S3_73319072
		S1_66996357

Catogoery	Total	SNPs
		S3_63284427
		S3_65664541
		S3_54220544
		S1_72938481
		S2_70188627
		S1_61008560
		S4_2233838
		S2_69151457
		S7_6859891
		S3_55139723
		S1_76479709
		S1_51067484
		S2_53473331
		S5_59436380
		S3_48915503
		S2_56916033
		S2_4554986
		S2_4812414
		S3_71483660
		S9_44339509
		S5_65814714
		S7_58579910
		S5_2549038
		S3_73887805
		S9_57273124
		S2_61705965
		S1_76292306
		S2_75152993
		S9_57797792
		S4_13491115
		S4_6124952
		S1_30330394
		S1_66555809
		S1_15897299
		S2_62095767
		S2_65603572
		S4_56200683
		S8_2068312
		S9_50178179
		S9_54655101
		S9_55626938
		S1_57485731
		S1_60915812
		S4_50305079
		S8_53646072
		S7_614086
		S2_3388919
		S3_73184361
		S3_57291957
		S7_3645345
		S7_574226
		S8_2016309
		S8_29984495
		S1_7810920
		S8_1876479
		S1_73639638
		S1_63402930
		S2_58025877
		S5_69851837
		S7_574219
		S7_54787360
		S4_2292151
		S4_26502904
		S2_3990948
		S2_18810921

Catogoery	Total	SNPs
		S2_8908610
		S4_12085525
		S5_7031007
		S2_8264180
		S6_50555340
		S7_61542699
		S2_64563632
		S8_1216814
		S8_60139667
		S1_59338090
		S1_78229004
		S5_69852503
		S2_12452485
		S3_70489532
		S3_72310757
		S5_68758403
		S4_3850074
		S3_56975930
		S1_65279181
		S4_1787962
		S6_18807165
		S5_63589130
		S3_15625105
		S1_75761773
		S9_53202193
		S5_61168249
		S6_60754933
		S8_9820831
		S1_6228482
		S2_8879063
		S9_57247173
		S1_5896344
		S5_4944814
		S6_53290673
		S5_12294691
		S7_58486832
		S6_45262279
		S7_6679582
		S1_19018104
		S2_76901396
		S1_59759834
		S6_48019735
		S3_16494786
		S7_41587363
		S5_20868111
		S6_30570846
		S1_72330083
		S7_6741723
		S3_68956518
		S2_61841929
		S2_66695445
		S1_72302171
		S2_8715502
		S5_46123247
		S7_62418797
		S6_72086
		S1_66894539
		S4_24385300
		S1_71606075
		S2_3998284
		S7_61571752
		S1_65570412
		S8_57452702
		S2_12670240
		S1_6331805

Catogoery	Total	SNPs
		S2_58887561
		S6_53240822
		S8_57571801
		S8_58836945
		S6_50005205
		S1_57444338
		S8_57452541
		S9_4743777
		S9_51879473
		S4_35632555
		S2_67837514
		S5_3181332
		S6_46381250
		S3_4583821
		S5_1999468
		S6_77555
		S8_51822724
		S1_66986048
		S5_67567225
		S2_47489192
		S6_51803535
		S9_58880878
		S3_73506871
		S4_51531866
		S8_53472459
		S6_55202612
		S1_73705952
		S2_28334844
		S4_10419232
		S6_41794304
		S2_61785876
		S8_57537535
		S8_282503
		S2_19732782
		S3_65910295
		S8_1554690
		S8_42371625
		S6_45704915
		S3_72184265
		S2_65330161
		S8_32420048
		S8_60599850
		S2_56963302
		S2_66185252
		S8_35248876
		S5_11683063
		S1_56982718
		S1_7164682
		S3_69941189
		S1_71747292
		S1_79962330
		S7_65097795
		S2_14130680
		S1_47978551
		S8_56781375
		S6_46801672
		S1_65691341
		S6_56547585
		S6_1316571
		S1_14876816
		S3_33670780
		S3_60583207
		S3_4484790
		S1_66395121
		S1_67623686

Catogoery	Total	SNPs
		S6_50119231
		S6_61123761
		S1_18967154
		S9_5167969
		S1_6465019
		S6_38194544
		S6_1668342
		S2_12509159
		S2_1510531
		S3_5138289
		S2_57847994
		S3_68460856
		S3_50593444
		S8_4310174
		S4_62878158
		S6_50507774
		S1_11205155
		S2_11010746
		S1_9751905
		S4_49719584
		S2_18141746
		S5_2927694
		S7_17164850
		S1_79647970
		S4_40568778
		S4_9760286
		S7_51847244
		S1_13284820
		S6_48602186
		S7_62966552
		S2_63676055
		S3_73315354
		S7_64515393
		S5_51346365
		S8_58029245
		S9_51887641
		S5_62639568
		S4_63627669
		S7_58611223
		S4_51688438
		S2_64076798
		S6_53726916
		S2_16961492
		S1_7426613
		S3_71729897
		S8_5076040
		S1_78496076
		S5_2547640
		S1_12853226
		S1_64451269
		S2_13459030
		S3_1906170
		#N/A
		S1_8644865
		S2_44177081
		S2_60118488
		S7_63671288
		S8_1839160
		S5_3393375
		S9_8622778
		S2_4812416
		S1_66568094
		S1_56567771
		S2_4155163
		S9_3540347

Catogoery	Total	SNPs
		S2_8370756
		S6_44722283
		S1_59697043
		S3_7387759
		S4_63405544
		S1_15854580
		S1_67679847
		S8_4678936
		S8_60911866
		S1_55301411
		S4_51119006
		S3_53212608
		S7_60598717
		S2_23311359
		S1_64803268
		S2_6178217
		S6_46569102
		S1_7639585
		S1_19054770
		S5_59317013
		S1_58913450
		S1_28059955
		S2_49781544
		S1_24603274
		S5_8995240
		S6_25378295
		S8_51884325
		S7_62194055
		S4_13287074
		S5_8655283
		S2_63115311
		S7_9515477
		S2_66925431
		S8_61954395
		S2_10025482
		S6_50881735
		S2_2521836
		S3_70242190
		S2_61047602
		S5_62493117
		S2_5537505
		S9_2744850
		S7_62030038
		S1_17468379
		S3_3061021
		S3_56869306
		S4_52877087
		S1_7499627
		S7_56269844
		S1_12968369
		S4_5107608
		S4_66386085
		S1_3220409
		#N/A
		S2_64464763
		S4_10689123
		S3_74143082
		S6_48690826
		S2_56134005
		S3_73765885
		S1_65494594
		S1_52802421
		S3_6756009
		S4_7780306
		S8_38329918

Catogoery	Total	SNPs
		S6_1067476
		S5_5081526
		S9_9086209
		S8_47777504
		S1_75661671
		S6_34857955
		S9_6694450
		S4_54085169
		S1_79193437
		S2_12357366
		S2_60480296
		S4_67115748
		S7_6670782
		S2_61333208
		S6_334686
		S9_59253274
		S5_7495189
		S4_66115060
		S2_65679876
		S2_10200335
		S3_6636661
		S6_2665892
		S3_52260885
		S5_12278986
		S4_56427448
		S4_33270781
		S8_42403746
		S2_2323113
		S1_59369684
		S7_63618750
		S9_5232688
		S5_67059022
		S2_3332851
		S6_47420126
		S1_53480996
		S2_16908345
		S7_63767166
		S3_56085487
		S8_60140365
		S3_68563611
		S2_59791373
		S2_61856952
		S1_67059674
		S3_71247764
		S2_59126029
		S2_8430562
		S6_55690184
		S2_59754261
		S3_67001538
		S1_77829783
		S1_20728054
		S5_11065579
		S3_69630951
		S8_51715246
		S8_6325816
		S2_66185156
		S9_8622301
		S7_59393537
		S9_54939923
		S1_14090640
		S4_1669742
		S7_5535267
		S8_61540936
		S1_14161999
		S1_55000306

Catogoery	Total	SNPs
		S6_35890005
		S9_2566744
		S2_67420556
		S4_2563779
		S1_7556615
		S2_59698435
		S2_67339166
		S1_57882865
		S6_11837401
		S3_637667
		S1_5727261
		S2_74965508
		S9_40810638
		S1_16740477
		S3_62988151
		S5_15821753
		S2_59226922
		S2_59878126
		S2_2758321
		S4_1054110
		S8_57316486
		S5_8068936
		S3_70489570
		S2_65881891
		S1_60838090
		S1_7992795
		S2_58576737
		S1_65583307
		S6_39651296
		S1_65740380
		S2_6237122
		S5_62379326
		S8_55144780
		S1_2469278
		S9_10773137
		S3_73184076
		S5_61217714
		S2_4886107
		S8_3018097
		S8_56764675
		S1_4579194
		S6_48247578
		S4_23915712
		S2_71486000
		S5_11791439
		S1_17864174
		S1_60370253
		S9_40978353
		S6_49691712
		S1_78798189
		S2_58140704
		S6_25586546
		S8_3609765
		S7_54708088
		S2_68021423
		S6_52704174
		S8_3491582
		S8_49317914
		S3_62421264
		S5_69794462
		S4_4809104
		S1_50852730
		S1_61978775
		S2_61551124
		S4_17525191

Catogoery	Total	SNPs
		S1_58471530
		S1_78711527
		S8_44955172
		S1_6922993
		S5_67404982
		S3_73170918
		S8_60478488
		S3_69421192
		S1_62967233
		S3_70711126
		S9_51705480
		S1_17489433
		S1_66984881
		S2_64518387
		S8_56721706
		S2_69858484
		S8_60222781
		S2_76974653
		S1_80311883
		S8_54133600
		S3_73826458
		S4_53344060
		S7_54411319
		S8_53860546
		S4_62988577
		S3_56656307
		S1_72637704
		S1_66791903
		S6_47179003
		S3_62054534
		S1_77425671
		S3_53397167
		S1_64409073
		S1_16550667
		S3_71369203
		S6_51477221
		S9_1071427
		S1_70431431
		S1_64145105
		S5_1545543
		S7_54787522
		S8_5018170
		S5_7341877
		S5_2920233
		S9_57812297
		S1_19018101
		S2_59247254
		S3_15611351
		S2_56135712
		S8_59648663
		S4_52450553
		S3_65364682
		S9_42245544
		S1_72715883
		S3_66671209
		S6_47955764
		S8_1876451
		S9_5862579
		S5_1530781
		S2_58202068
		S2_73693397
		S9_57657918
		S7_61282982
		S2_59237127
		S2_68060345

Catogoery	Total	SNPs
		S8_59837888
		S8_61551512
		S1_59471498
		S9_2228512
		S1_63253431
		S1_20843866
		S3_56946698
		S8_51316699
		S1_58913436
		S1_79053216
		S2_10723728
		S2_62683544
		S9_8192302
		S7_62909796
		S5_2693011
		S1_24777285
		S2_58531805
		S7_39828990
		S4_36546125
		S7_62611632
		S2_60985518
		S2_41395954
		S6_453002
		S7_9788490
		S1_1943117
		S5_12728756
		S1_2908045
		S6_58131391
		S1_53479622
		S8_53817170
		S5_58482169
		S1_46824118
		S8_2050989
		S5_24020388
		S8_39703007
		S4_10304304
		S1_11562512
		S9_5041227
		S9_52579118
		S5_4006132
		S1_72206034
		S2_12735173
		S3_73335294
		S2_7443274
		S3_66409387
		S8_10101601
		S1_71640633
		S9_52074449
		S1_17970168
		S1_60005820
		S3_68055671
		S1_76291133
		S1_65273064
		S1_22035123
		S1_77814847
		S4_772503
		S5_2596819
		S2_65043050
		S3_72515069
		S1_11195619
		S2_59310033
		S3_57776968
		S2_59470505
		S3_73010881
		S6_47179450

Catogoery	Total	SNPs
		S4_4140071
		S6_52635345
		S3_3915294
		S4_10202435
		S8_59348450
		S1_2730653
		S2_62795693
		S1_13460527
		S2_46915020
		S2_76899467
		S2_4001879
		S7_62852084
		S8_2879089
		S1_386131
		S5_12426840
		S1_79611756
		S1_78225751
		S6_50584567
		S1_72302167
		S7_313551
		S4_67115712
		S2_58011493
		S8_46349674
		S6_46372055
		S6_49220976
		S2_65855583
		S5_2966602
		S6_47181109
		S9_45624897
		S8_3183164
		S1_72346550
		S8_54222774
		S9_47005966
		S1_60720877
		S4_38749574
		S1_79033076
		S2_65232228
		S4_47808765
		S1_54031718
		S7_60841798
		S1_12968375
		S4_61863249
		S8_3369923
		S6_47072591
		S6_47319583
		S8_53840461
		S1_11499513
		S2_60290270
		S7_2117999
		S2_10517507
		S1_1652453
		S3_55801545
		S4_55489319
		S8_18336934
		S2_57607887
		S9_58179029
		S1_67667896
		S2_61694907
		S8_61922637
		S4_9760228
		S3_68263779
		S8_55049128
		S5_70008134
		S1_496319
		S7_7948243

Catogoery	Total	SNPs
		S4_52774711
		S1_77362374
		S4_5107452
		S6_51902863
		S5_62505738
		S7_58993343
		S1_2659325
		S8_49596268
		S1_75863305
		S3_72634771
		S4_388787
		S2_467604
		S5_1999477
		S3_3294740
		S3_68263795
		S2_1052023
		S2_77175538
		S8_53790529
		S1_12920445
		S3_811884
		S3_6212234
		S3_16632442
		S3_69530893
		S7_63082417
		S8_12577072
		S1_2909526
		S2_60677774
		S4_4321453
		S9_50596054
		S1_65273071
		S2_271651
		S2_49387071
		S2_53430284
		S2_73494261
		S3_69365417
		S1_8644870
		S2_72264311
		S3_72849719
		S4_41483966
		S4_7338338
		S2_59357527
		S3_64343656
		S8_2231159
		S4_4855493
		S9_1260362
		S6_56925712
		S4_5850163
		S8_6713614
		S4_7915118
		S2_6517006
		S3_3480315
		S4_650573
		S6_49474173
		S1_60698408
		S2_6179398
		S3_72171202
		S4_2151618
		S7_6922584
		S2_59341674
		S8_5320752
		S6_54759104
		S2_1030603
		S8_61921414
		S3_2576554
		S1_19462334

Catogoery	Total	SNPs
		S2_58591505
		S4_51951353
		S3_61824417
		S2_9663701
		S1_67949142
		S4_6773676
		S1_2496611
		S6_5765055
		S5_61217563
		S2_45629337
		S9_55976263
		S4_66199017
		S7_9519645
		S5_62755991
		S1_66880377
		S1_9921048
		S6_47699853
		S9_49058638
		S6_2599216
		S2_67574082
		S3_6759143
		S6_52683119
		S6_26020400
		S8_48459076
		S1_6466503
		S6_47331692
		S1_2730684
		S4_16183887
		S1_72898841
		S2_60986375
		S2_10200356
		S8_1753901
		S2_1032953
		S1_16963233
		S9_1071475
		S2_6070257
		S6_25538765
		S9_52837453
		S8_3347441
		S1_59892803
		S5_2619034
		S3_56160315
		S4_9760421
		S2_65774204
		S3_72790008
		S7_10969500
		S9_51467122
		S2_10821649
		S1_28576997
		S7_63072545
		S3_73920893
		S3_1905134
		S7_10006273
		S7_52729967
		S3_15435899
		S2_62936484
		S9_52930432
		S9_9099752
		S1_7822995
		S1_60005607
		S1_5806905
		S1_48528744
		S2_10269493
		S2_76427948
		S8_60337671

Catogoery	Total	SNPs
		S5_68881899
		S6_1555580
		S9_7887040
		S2_11561745
		S9_1707348
		S1_2646590
		S3_72128611
		S9_2531705
		S8_56576211
		S4_4031960
		S7_63975953
		S2_62051197
		S1_74940026
		S4_25787608
		S9_7800195
		S5_71216636
		S3_70239278
		S2_64144918
		S3_450985
		S7_62143572
		S7_58579945
		S1_16439897
		S9_215204
		S7_63639758
		S7_54787413
		S1_13832004
		S4_6615699
		S2_58418814
		S5_13410275
		S6_45570600
		S6_53396972
		S2_61695392
		S3_68058309
		S8_57785409
		S6_47539450
		S3_64927383
		S2_67327871
		S4_12354680
		S7_58665614
		S1_7960217
		S1_79155941
		S2_7820429
		S9_52064237
		S3_66606503
		S6_1112985
		S8_61642584
		S3_69365291
		S3_66015555
		S2_2383143
		S9_8377113
		S3_66017546
		S2_6004847
		S4_6414082
		S6_3914932
		S4_25806108
		S6_4457578
		S1_30656515
		S8_61922615
		S9_49705821
		S2_64165339
		S6_46483048
		S2_11952330
		S9_59301072
		S8_3671194
		S1_8973950

Catogoery	Total	SNPs
		S4_54492025
		S1_26890787
		S1_62552544
		S7_63739641
		S1_79485336
		S8_37897673
		S2_76914263
		S5_66785688
		S2_59948021
		S3_5130518
		S3_55204438
		S9_8267862
		S2_68554623
		S6_41288294
		S5_66622470
		S1_79355821
		S9_51545844
		S2_65028948
		S7_62131257
		S4_2302907
		S2_63520178
		S2_65295441
		S9_58916307
		S1_61354679
		S8_3579334
		S8_54938492
		S2_58996714
		S4_67081509
		S1_7025766
		S3_5787681
		S9_51450604
		S6_52730780
		S2_17601503
		S1_3128447
		S1_24129857
		S2_75151197
		S9_6694507
		S1_80207910
		S7_2413180
		S3_62556005
		S7_56589396
		S1_58350410
		S4_3380971
		S2_57493055
		S7_58340519
		S1_71830409
		S3_56295939
		S8_61636111
		S3_69937692
		S2_47739430
		S3_68263785
		S2_13108380
		S5_62376102
		S3_56160357
		S7_39932996
		S1_18913483
		S2_72264277
		S9_52980800
		S1_72010088
		S5_50489062
		S7_17493628
		S4_66243127
		S1_50341821
		S2_61270377
		S3_71795000

Catogoery	Total	SNPs
		S4_2253586
		S1_79010933
		S2_62310053
		S1_6512008
		S1_77329039
		S2_69602028
		S4_2284039
		S1_64631718
		S2_67807467
		S5_71216782
		S8_3477004
		S5_62493112
		S8_1991582
		S8_59672049
		S4_33082831
		S8_54319923
		S1_68247463
		S6_9992731
		S2_55043207
		S2_67837727
		S2_65234273
		S3_53323836
		S3_5620530
		S2_76974631
		S8_50018536
		S8_57753871
		S1_8495446
		S6_2665927
		S6_48158436
		S9_52077391
		S1_30227136
		S7_58845974
		S3_56719302
		S8_5259990
		S5_335559
		S2_2887307
		S6_54583403
		S3_15611130
		S6_933765
		S1_11205644
		S7_6326941
		S1_6331801
		S2_8050396
		S5_1326992
		S8_60931624
		S1_59936988
		S5_66622625
		S9_47051440
		S9_59378885
		S6_8301942
		S6_45570162
		S7_64417778
		S3_56385175
		S5_61103375
		S9_4374438
		S6_56333601
		S1_22243987
		S1_79438064
		S3_72184279
		S2_17768722
		S1_63291020
		S8_3193983
		S3_66415975
		S4_15178859
		S8_57948162

Catogoery	Total	SNPs
		S1_9068971
		S8_56419686
		S6_47764979
		S4_41650227
		S1_68725299
		S1_328700
		S1_74100405
		S3_70855645
		S5_10697870
		S2_75804562
		S9_51890192
		S2_68945998
		S9_5861369
		S2_62726284
		S6_55723646
		S2_1587787
		S1_27149566
		S9_7373715
		S4_56549689
		S4_66386089
		S2_68059554
		S6_38608647
		S1_2703546
		S2_40243565
		S6_57407435
		S7_64061431
		S4_4499981
		S9_54882387
		S1_77674496
		S9_8190458
		S3_59702762
		S5_62355356
		S2_77175549
		S3_5683126
		S6_49276525
		S3_45646225
		S6_51225105
		S2_18990355
		S1_73747456
		S2_5685702
		S1_68195770
		S2_71471884
		S2_73675565
		S1_64479609
		S2_74927262
		S1_24777327
		S3_52047912
		S3_57894973
		S4_3679221
		S7_62909767
		S5_515636
		S7_56660942
		S3_52014166
		S8_50831057
		S2_46766410
		S8_5062042
		S8_50855532
		S2_18811186
		S3_67154244
		S3_57144653
		S5_58297822
		S2_4704041
		S6_4444780
		S9_1202088
		S3_5151643

Catogoery	Total	SNPs
		S9_2970145
		S5_66007326
		S2_74965468
		S2_61964260
		S2_32083108
		S5_62396401
		S8_45517551
		S2_6230153
		S4_51960013
		S2_75368937
		S5_67481765
		S7_5181803
		S8_51309282
		S1_12967750
		S6_37906103
		S8_56800359
		S2_63996164
		S2_65359762
		S7_42590156
		S4_1009218
		S1_16453923
		S1_13471804
		S1_10734491
		S2_61424020
		S2_65725002
		S7_6984847
		S3_57257517
		S3_51465164
		S8_61847990
		S4_24706844
		S8_60695659
		S4_66295916
		S1_66340302
		S1_3156777
		S4_3715862
		S1_19554075
		S1_12595221
		S1_80479935
		S9_52158002
		S6_52691505
		S3_51636594
		S8_61547456
		S7_8561612
		S3_53583421
		S3_72414662
		S1_79193448
		S4_47808748
		S1_59892866
		S9_7276824
		S1_15673318
		S5_8157484
		S8_46599234
		S1_71946559
		S6_53240807
		S8_56797541
		S1_68301252
		S8_54939119
		S6_47101159
		S2_60166323
		S1_16529815
		S1_7843750
		S9_8151552
		S2_31659152
		S3_55904180
		S1_18109581

Catogoery	Total	SNPs
		S4_10304304
		S6_1238526
		S5_1248444
		S6_1375079
		S6_15431899
		S1_2952783
		S1_6648364
		S1_57661111
		S3_66675007
		S8_5157262
		S8_51123933
		S2_61712473
		S3_4276235
		S3_73319071
		S2_10152719
		S1_2909418
		S2_58210273
		S2_59538556
		S4_7333134
		S3_59542666
		S1_15897265
		S1_13449959
		S2_65965887
		S4_5828909
		S3_5130752
		S5_61998102
		S8_60901037
		S3_57760678
		S1_2685363
		S9_50874688
		S1_74940023
		S9_44042823
		S7_2620887
		S1_19341829
		S5_61187255
		S7_60723643
		S5_66267751
		S5_70796989
		S2_65453313
		S9_2984333
		S2_68991232
		S3_73925063
		S4_50273341
		S2_55094464
		S1_4473608
		S2_73831268
		S1_51841724
		S6_60460605
		S2_3415329
		S2_6045275
		S5_11706315
		S4_47808758
		S7_58608197
		S7_62935566
		S8_47523896
		S2_6890937
		S2_12683949
		S2_13167482
		S5_9550442
		S1_17468425
		S9_4067522
		S4_3928120
		S2_11765742
		S3_57882256
		S6_335453

Catogoery	Total	SNPs
		S6_32205882
		S1_78944590
		S9_1647377
		S1_10834765
		S2_12357916
		S7_63699327
		S8_49983668
		S4_66123849
		S2_67745014
		S8_60557485
		S1_57884249
		S2_75810625
		S5_1999460
		S3_3900246
		S8_18012323
		S5_14150678
		S4_66367328
		S2_58025293
		S1_79155248
		S4_66144678
		S5_64034493
		S5_4006132
		S2_61685838
		S2_12466186
		S1_67311309
		S1_17902701
		S4_10717505
		S1_18916948
		S3_572381
		S9_55043320
		S1_7586040
		S6_47959712
		S7_2029818
		S8_54348558
		S2_71517839
		S2_64227884
		S2_8217219
		S9_52924309
		S1_55830801
		S7_946462
		S4_53449457
		S5_62129130
		S1_14448331
		S3_63730959
		S8_44487811
		S1_52755744
		S6_57948178
		S7_39716042
		S1_75620805
		S4_51121190
		S8_53883246
		S1_59386679
		S1_8616815
		S9_57905473
		S4_46977096
		S7_64715098
		S3_57929041
		S6_41007972
		S2_9874449
		S5_66956000
		S1_19055442
		S1_64631745
		S8_56472834
		S4_7327010
		S3_69901732

Catogoery	Total	SNPs
		S5_15793521
		S4_51492933
		S1_10690759
		S4_699963
		S6_2806548
		S8_1238694
		S1_79194934
		S3_57340081
		S3_47202213
		S8_9147036
		S7_8905492
		S8_58489926
		S6_56806428
		S5_68975468
		S1_64422632
		S3_71236542
		S7_63554023
		S9_16384
		S4_19383673
		S1_15186216
		S1_19057253
		S2_65228856
		S4_7929373
		S2_7513769
		S4_4160579
		S5_2894417
		S1_3156780
		S1_67234301
		S9_59219291
		S1_7069594
		S7_41781613
		S7_64235720
		S1_12223183
		S7_15407642
		S8_53214565
		S2_8696581
		S2_58290735
		S2_11907351
		S9_54455328
		S7_16661368
		S1_24777313
		S9_45624884
		S7_9515492
		S1_56337775
		S2_59247243
		S9_10351575
		S5_67938252
		S1_78510628
		S4_34910960
		S6_46527356
		S1_78787985
		S2_65029152
		S2_60289368
		S1_13891947
		S1_78288332
		S4_66239236
		S2_10976622
		S2_6036380
		S9_2658134
		S2_57519656
		S5_10193360
		S7_56574480
		S9_54040228
		S2_9663705
		S2_73584609

Catogoery	Total	SNPs
		S2_64495132
		S6_56513261
		S3_3700787
		S2_5965529
		S2_61226303
		S3_66410437
		S3_2018396
		S9_54346798
		S2_75808057
		S9_1719150
		S4_50669747
		S8_60933905
		S1_58201059
		S8_55626604
		S1_9947811
		S2_60165276
		S2_66339817
		S5_2829045
		S3_73335148
		S6_60698202
		S7_6373423
		S1_59131455
		S2_26002703
		S2_61424032
		S1_67716050
		S2_2166982
		S2_72311014
		S3_53583434
		S6_50606431
		S1_59398817
		S6_40434369
		S1_79194029
		S1_66827660
		S3_68503336
		S8_39167934
		S2_62739820
		S9_53230189
		S2_63173794
		S2_8230798
		S1_15851565
		S8_59250985
		S9_6364897
		S1_52732849
		S1_59457247
		S5_10195573
		S6_10566953
		S8_2635781
		S5_18445944
		S5_71216694
		S1_9062576
		S2_3251754
		S2_64075795
		S1_15324252
		S8_57761344
		S1_57874615
		S6_44752163
		S7_60654674
		S9_8128137
		S2_3405866
		S9_5036353
		S1_61875842
		S1_58960870
		S5_6166269
		S8_15448441
		S6_35409254

Catogoery	Total	SNPs
		S3_67541328
		S7_9512691
		S2_41098655
		S8_44942779
		S2_75251212
		S7_273081
		S3_6212262
		S7_12092833
		S2_59247346
		S2_73117455
		S6_36178441
		S1_20564832
		S2_68021540
		S1_6491557
		S5_2640392
		S4_12350788
		S1_6845861
		S4_22245803
		S6_47955708
		S6_47064142
		S3_720630
		S3_58246378
		S3_61384354
		S6_53622973
		S7_58506917
		S5_65843249
		S2_59912776
		S3_65335756
		S1_66026879
		S5_11064943
		S8_49628994
		S2_65199732
		S6_45215504
		S5_2829075
		S2_32127476
		S2_61272138
		S9_20596494
		S4_12164779
		S3_58315056
		S3_6755993
		S6_50801591
		S1_16543107
		S1_53854275
		S4_8616435
		S6_5878724
		S9_53229793
		S1_66565349
		S5_15116558
		S9_7595990
		S3_73705007
		S4_7901410
		S9_58935184
		S2_66211803
		S3_68058267
		S1_46554366
		S1_59471495
		S7_64827128
		S2_75945253
		S4_66431349
		S6_49691899
		S3_72557573
		S2_1474484
		S4_41657121
		S2_426460
		S1_15854590

Catogoery	Total	SNPs
		S9_54947171
		S2_69327063
		S3_71689848
		S4_56306377
		S1_79659906
		S9_51890209
		S4_42275630
		S1_20014333
		S1_72605771
		S2_2644723
		S2_65877655
		S8_1876490
		S1_67316157
		S3_57770410
		S8_49983698
		S9_55056654
		S9_52654147
		S1_9751972
		S7_63318977
		S9_4690053
		S5_69804013
		S4_21145862
		S6_51255334
		S2_15285463
		S3_69630776
		S3_72791490
		S4_15604344
		S7_62611882
		S9_1750922
		S1_78019737
		S1_79576288
		S1_14320020
		S6_26180148
		S3_4970414
		S6_16291764
		S1_15025423
		S9_55046608
		S2_15337900
		S4_33082871
		S6_56572625
		S1_77100628
		S1_78290710
		S3_59558286
		S3_56064300
		S5_68815225
		S1_77100619
		S3_72404691
		S1_62732118
		S4_5107585
		S4_2605796
		S6_47278637
		S2_73212547
		S5_15793544
		S5_69851941
		S3_68460868
		S7_53150041
		S1_9366502
		S3_68243803
		S8_1172535
		S9_57678978
		S9_3881581
		S6_33755237
		S1_60834903
		S6_51488375
		S3_5751838

Catogoery	Total	SNPs
		S2_2323600
		S4_50974937
		S4_11072585
		S7_65312449
		S3_58877249
		S4_2360480
		S7_58153651
		S1_65736314
		S2_63426480
		S7_54684657
		S3_48838371
		S1_8138429
		S1_57665183
		S3_50412484
		S7_60181885
		S7_8523517
		S2_8089789
		S4_62988601
		S4_3789100
		S1_60005642
		S2_61085231
		S8_3379948
		S8_42616074
		S1_67191691
		S1_14872726
		S2_67621350
		S4_39801949
		S8_56144775
		S4_13422934
		S5_66375040
		S2_59199489
		S4_772578
		S5_7776373
		S5_15836224
		S7_62611640
		S4_66125740
		S1_79012120
		S2_14641119
		S3_71247635
		S8_53877602
		S4_51737955
		S2_56870783
		S6_28883214
		S2_16390665
		S9_50538325
		S4_25646758
		S6_45989586
		S9_57840065
		S7_64098592
		S5_3621087
		S5_4616949
		S1_68573569
		S3_65328198
		S1_66101338
		S1_7088189
		S7_8931621
		S5_5269875
		S1_12920865
		S5_68171090
		S9_49954
		S4_23819508
		S9_52461655
		S5_1921679
		S1_2923788
		S9_2327730

Catogoery	Total	SNPs
		S3_3902074
		S6_50267135
		S2_4554976
		S4_1646794
		S1_8745755
		S6_55684145
		S8_60911893
		S2_6799061
		S2_62729844
		S3_74011164
		S1_65984154
		S1_16741733
		S6_50396679
		S1_26930469
		S6_1941077
		S1_77335185
		S9_4575815
		S1_65672258
		S5_62636348
		S2_9492261
		S4_9075829
		S9_50154003
		S4_5534893
		S9_4389368
		S9_1240396
		S4_7562123
		S1_14152424
		S5_24345561
		S3_5083943
		S1_53455464
		S2_329524
		S2_63676075
		S3_69209170
		S2_10557070
		S9_2768876
		S6_51410261
		S2_76805384
		S9_57547417
		S7_63156082
		S7_2559103
		S8_53241142
		#N/A
		S6_54583385
		S2_61060074
		S7_63333776
		S1_2090043
		S8_4310375
		S1_1629569
		S8_57738531
		S7_60709313
		S2_75357100
		S7_816043
		S2_56134076
		S6_58535646
		S7_831859
		S9_214866
		S3_71372606
		S7_58434686
		S1_12369926
		S5_10561909
		S6_51477216
		S9_57363115
		S3_61246828
		S5_69851715
		S8_18210816

Catogoery	Total	SNPs
		S6_49755275
		S6_58049134
		S2_2241164
		S3_63357217
		S9_53672117
		S1_6462602
		S8_51438280
		S9_54947167
		S7_65242015
		S3_55300414
		S9_5861420
		S2_2007365
		S2_10525640
		S6_40396514
		S9_52356832
		S2_36124792
		S7_57879511
		S8_49596499
		S3_51904170
		S3_16637160
		S8_57589346
		S9_53672115
		S3_70252887
		S1_19608513
		S2_10398341
		S4_26443412
		S8_60383384
		S4_33698440
		S4_41758816
		S7_5813587
		S3_73010537
		S9_55817143
		S8_42700961
		S9_58293203
		S3_70528503
		S2_66492132
		S3_60919704
		S1_79194066
		S8_40677631
		S6_60594881
		S8_4993977
		S5_65658074
		S2_4555002
		S5_9571196
		S7_2321503
		S2_10242937
		S4_62936545
		S3_62407027
		S7_6206292
		S2_7112069
		S2_6379918
		S5_69879669
		S6_56806438
		S1_4084303
		S6_50286368
		S9_59318543
		S8_58745566
		S6_54738346
		S8_53840066
		S3_46943814
		S8_58890002
		S4_12412357
		S8_55972704
		S1_20255550
		S7_63713073

Catogoery	Total	SNPs
		S2_69698165
		S5_2920216
		S3_73765884
		S5_21779424
		S4_38744782
		S4_35510758
		S2_17689501
		S3_74112319
		S1_56701348
		S7_63448048
		S2_59216297
		S4_38807113
		S1_4473606
		S8_49629012
		S5_12294688
		S3_1245022
		S2_69155306
		S2_12078769
		S7_63639565
		S6_48693701
		S7_63968193
		S4_37200149
		S9_51705470
		S1_11756188
		S5_10052193
		S4_19153257
		S4_62942500
		S2_2392333
		S8_17185495
		S1_65672116
		S1_7559217
		S3_69847138
		S7_1525119
		S9_52076704
		S2_35149006
		S2_19798306
		S2_64413825
		S5_66098816
		S9_50014563
		S1_71946540
		S9_6862983
		S2_68554616
		S1_55830683
		S8_60170366
		S9_5231039
		S8_38036056
		S9_5184360
		S3_57958776
		S3_68055678
		S9_3881507
		S2_64181922
		S2_11944548
		S7_59400476
		S7_9519636
		S4_6119860
		S4_12086629
		S5_2966601
		S1_66978079
		S6_48638201
		S1_51003645
		S2_58682763
		S2_9485448
		S3_13362482
		S1_72865129
		S1_72859889

Catogoery	Total	SNPs
		S9_27329763
		S2_66465556
		S1_9760052
		S6_55690120
		S1_16387984
		S1_73622495
		S4_3882932
		S1_54120864
		S9_51705788
		S5_59401336
		S1_77161287
		S1_9996512
		S8_1216792
		S7_62470364
		S3_13434003
		S1_12054364
		S2_7541622
		S2_63547934
		S7_65081281
		S1_79932562
		S3_73765869
		S3_64927123
		S2_61527924
		S2_65335660
		S4_6371573
		S1_80001236
		S6_32591490
		S6_47306837
		S6_6448049
		S2_56515273
		S9_50465163
		S3_6599655
		S5_14948558
		S8_54939516
		S4_61180174
		S9_47214422
		S4_41756749
		S7_65289940
		S8_40583366
		S1_74546883
		S2_63583661
		S3_74146207
		S8_61551502
		S1_15039133
		S5_1005376
		S6_26180180
		S7_60933008
		S3_54099111
		S1_11612960
		S4_52816198
		S2_69037710
		S9_51836756
		S2_7112057
		S7_56590822
		S8_61918671
		S6_26020373
		S7_62143570
		S7_63217665
		S7_54787414
		S1_19645782
		S7_55982519
		S1_67303108
		S2_45306788
		S5_32174
		S8_4310383

Catogoery	Total	SNPs
		S1_79366006
		S6_453022
		S9_51454569
		S8_51309649
		S9_1663416
		S3_60599098
		S1_57535868
		S1_7961497
		S7_53871006
		S9_52471706
		S3_50995046
		S8_61621456
		S2_59247277
		S1_52350794
		S4_8586089
		S8_60349026
		S2_75563691
		S2_9529760
		S7_1845891
		S2_74972196
		S6_58178564
		S8_9589164
		S9_215193
		S4_66125722
		S2_64256828
		S3_57223919
		S2_59822692
		S7_492508
		S3_73701418
		S5_61208223
		S8_60933922
		S5_61073383
		S2_73151434
		S2_3774964
		S4_2161215
		S6_15381862
		S8_1689150
		S9_2233787
		S1_59824401
		S6_45715689
		S1_17468691
		S6_49768866
		S2_460677
		S1_3420291
		S7_9662487
		S5_69783488
		S5_61921365
		S4_42480874
		S8_46027815
		S4_66208717
		S2_69809422
		S9_1646650
		S7_64669574
		S1_73644741
		S2_12434688
		S5_62728463
		S8_3385214
		S3_6722666
		S8_56542079
		S4_11072584
		S8_1108418
		S6_7347926
		S3_5104638
		S1_6512013
		S8_58035784

Catogoery	Total	SNPs
		S6_1279560
		S7_9512668
		S5_3025832
		S1_63901731
		S5_6903572
		S5_17827329
		S9_6214190
		S1_65291588
		S4_49581043
		S3_59473346
		S1_19948006
		S6_8473181
		S6_16399982
		S6_28874151
		S4_53742484
		S3_70242200
		S5_11114834
		S1_30824654
		S4_4189781
		S3_58281922
		S1_12969775
		S2_65682126
		S1_60837455
		S8_2271662
		S7_57029228
		S8_30845438
		S8_60934216
		S5_69989076
		S3_52457541
		S3_3910496
		S2_16280523
		S1_10942940
		S9_51068875
		S4_25244982
		S2_75807159
		S2_3707568
		S2_60970181
		S1_60835043
		S9_1260266
		S6_31450139
		S4_7326974
		S8_59740114
		S6_58793136
		S4_1003011
		S7_52300385
		S9_3010056
		S6_30441715
		S1_1862843
		S5_12990692
		S9_58114225
		S4_48061038
		S9_214860
		S2_5489013
		S3_65353608
		S2_4594248
		S2_61550904
		S1_8579731
		S1_11686455
		S5_68952014
		S1_79401588
		S2_65854319
		S6_54381378
		S2_40141834
		S4_9492774
		S3_71490329

Catogoery	Total	SNPs
		S8_557297
		S9_4527037
		S9_44414317
		S3_71866728
		S5_62379371
		S7_12940756
		S1_6333165
		S4_52778238
		S5_490398
		S7_887919
		S6_45640370
		S7_9690830
		S7_7678366
		S5_59325685
		S3_52417775
		S3_7387989
		S3_72515105
		S9_40978344
		S9_7538608
		S4_61218160
		S8_1179165
		S2_11055028
		S1_914839
		S4_3814470
		S3_68460863
		S7_64858972
		S9_44038839
		S5_3184098
		S5_1326689
		S5_68864639
		S1_68311726
		S4_52413801
		S2_3760482
		S2_60356884
		S8_55144774
		S5_517168
		S1_59140569
		S4_18447995
		S5_1645212
		S6_38160460
		S4_9322342
		S7_60299059
		S3_69497837
		S2_5489037
		S3_56254519
		S1_75048528
		S3_13236935
		S5_15885755
		S4_16389249
		S8_2271777
		S3_52048043
		S8_58700463
		S2_10329041
		S1_1898800
		S1_73036656
		S2_73941954
		S4_26787063
		S5_1966403
		S8_62072396
		S1_78711530
		S3_65965756
		S1_17808949
		S3_69606289
		S6_44606876
		S1_8644843

Catogoery	Total	SNPs
		S1_78509948
		S8_53505592
		S2_68847631
		S2_43857414
		S4_66155190
		S8_4310187
		S5_1906020
		S2_68059577
		S8_59746742
		S1_54032120
		S1_58547313
		S1_22035534
		S1_15787311
		S6_53583314
		S2_50018963
		S7_54330413
		S7_63365633
		S1_11197431
		S2_68125410
		S8_18425705
		S9_55628268
		S4_3834023
		S6_61094910
		S9_54901651
		S2_60530173
		S4_63055590
		S5_3230485
		S7_39531433
		S7_6679582
		S9_7788424
		S1_62222525
		S7_63965032
		S9_8369040
		S2_1510467
		S4_20672544
		S5_66910020
		S1_68736161
		S1_72374415
		S3_1927690
		S3_70605640
		S7_54307713
		S5_68758435
		S8_38482059
		S2_73366238
		S1_71389911
		S2_68561607
		S5_11313777
		S3_55024298
		S7_61525351
		S1_61720681
		S1_2640029
		S8_59214640
		S5_65264993
		S2_76706601
		S4_52876172
		S5_3384251
		S1_20976808
		S2_57255571
		S1_79053206
		S2_75155375
		S9_57629133
		S3_70252944
		S5_1932964
		S1_1640901
		S2_68923542

Catogoery	Total	SNPs
		S2_73193875
		S5_11134561
		S7_64037716
		S2_60136233
		S4_701563
		S6_50435867
		S8_557204
		S1_1547293
		S4_4226842
		S6_46381721
		S6_51488194
		S9_1219589
		S8_55971124
		S4_5530923
		S2_58025890
		S2_18877865
		S2_67977263
		S6_53168311
		S7_6452781
		S7_1362414
		S7_8923621
		S3_68841081
		S3_70253248
		S5_4526956
		S5_61864586
		S6_45266130
		S5_10420029
		S9_8213270
		S2_3405860
		S1_73585248
		S5_8995210
		S6_2595755
		S1_10317815
		S8_59990944
		S1_30788456
		S2_55354433
		S7_6671223
		S6_47105347
		S5_58451299
		S1_1547296
		S1_74411182
		S5_65697673
		S8_62054305
		S9_44037326
		S1_60760852
		S4_66113260
		S1_66528467
		S2_63218425
		S9_55043671
		S3_64963628
		S1_72859908
		S1_80002017
		S2_10527889
		S9_52939463
		S7_437396
		S7_63713076
		S7_63318804
		S1_60803136
		S2_10147783
		S9_44414271
		S2_61060336
		S5_50603255
		S9_4365937
		S3_5617782
		S8_47531008

Catogoery	Total	SNPs
		S9_5861359
		S6_15065313
		S6_1047757
		S5_61897002
		S3_54091796
		S5_62505741
		S2_73050013
		S2_12802112
		S5_70051284
		S3_3063060
		S4_34694179
		S4_58231984
		S9_9829303
		S4_67087244
		S3_68336457
		S2_63377439
		S7_63787152
		S3_60935419
		S1_13912072
		S9_1026639
		S6_50435972
		S9_48956454
		S3_20545647
		S8_56411885
		S7_54409639
		S2_61654405
		S1_79843437
		S2_17761151
		S6_45174800
		S7_10063256
		S3_46175365
		S6_52704430
		S4_33582358
		S3_46805467
		S3_71061685
		S6_52885150
		S6_40940620
		S7_19536627
		S2_57862916
		S2_6249992
		S3_52629214
		S9_3351146
		S2_6019772
		S5_1120046
		S1_10949864
		S1_77745760
		S3_65348738
		S1_77795778
		S2_61742750
		S2_77054061
		S3_71422092
		S2_62683545
		S1_11686443
		S6_45570614
		S9_59378891
		S2_50067130
		S8_1438318
		S4_52949045
		S7_62611643
		S5_66666055
		S4_63395449
		S2_65280422
		S7_56006347
		S3_713463
		S1_11205600

Catogoery	Total	SNPs
		S2_59125994
		S8_46410411
		S3_71247204
		S7_63318967
		S1_19640917
		S6_52884464
		S9_50520539
		S4_37609907
		S7_61539100
		S8_48576692
		S6_47699772
		S1_30553536
		S2_67839041
		S5_11055594
		S1_75376227
		S4_13850995
		S9_53867594
		S2_75262818
		S1_67226051
		S3_69836928
		S4_53383879
		S7_1488482
		S8_60933468
		S1_63004834
		S3_54416444
		S1_75861427
		S5_3179799
		S2_69593618
		S2_56174112
		S8_57298718
		S9_50009010
		S1_74490535
		S2_65487914
		S1_7516398
		S8_407411
		S5_12029401
		S4_56306389
		S6_50756985
		S9_4238663
		S8_2195953
		S4_20618390
		S1_79195143
		S5_6176828
		S2_63059485
		S8_61603505
		S2_18095223
		S4_3418566
		S9_35287628
		S8_61925069
		S3_66606470
		S2_75048381
		S7_13052585
		S1_55830282
		S2_67873679
		S4_25645551
		S3_5132561
		S1_6787873
		S1_66986853
		S3_73926276
		S6_47800394
		S1_15673280
		S1_1862804
		S5_66097561
		S1_68301054
		S9_5829132

Catogoery	Total	SNPs
		S5_67398466
		S4_6415464
		S3_70567746
		S7_58350148
		S1_60517691
		S1_71946545
		S3_5041658
		S8_9739485
		S1_12305233
		S6_60774036
		S8_56721713
		S6_54339769
		S9_50744162
		S2_21219483
		S8_3823473
		S3_45571043
		S7_54947459
		S9_56115148
		S1_19055459
		S2_53430345
		S1_75386742
		S2_16844426
		S4_7030239
		S6_975652
		S2_75422105
		S1_46315663
		S3_47282697
		S9_50874528
		S2_4900417
		S2_125748
		S1_3574407
		S9_54056760
		S7_57208825
		S3_450978
		S3_70573558
		S1_66026945
		S4_50854148
		S2_76943639
		S3_811890
		S3_1964488
		S6_51255333
		#N/A
		S8_56411886
		S1_56441664
		S4_54430488
		S1_65681077
		S2_41529470
		S6_26447239
		S5_68110824
		S9_4346898
		S1_8138447
		S1_6575226
		S1_2271899
		S5_56426799
		S4_40248420
		S5_1966426
		S9_5861332
		S3_72123500
		S8_50444392
		S1_77821250
		S2_66008929
		S1_78754979
		S1_63709916
		S8_9841427
		S2_39452979

Catogoery	Total	SNPs
		S6_38609585
		S2_69860072
		S9_53720738
		S3_713219
		S8_56642681
		S3_61963286
		S9_9189070
		S8_57423880
		S1_70412987
		S9_58485777
		S2_65603581
		S3_69118027
		S8_1238706
		S4_52459369
		S2_2788908
		S2_2766846
		S5_3397181
		S2_13177158
		S1_22269381
		S3_71666503
		S8_5096384
		S2_3979068
		S6_31450157
		S3_51838794
		S3_5627477
		S5_1608375
		S6_34562228
		S2_56955030
		S6_38175740
		S3_52457569
		S4_1489084
		S7_64967353
		S8_58901760
		S2_63508527
		S4_53874427
		S2_1609868
		S4_38153471
		S1_11205229
		S2_10814277
		S1_66723308
		S9_217558
		S1_3461870
		S7_54573415
		S5_62156650
		S3_69587305
		S4_772486
		S1_2779864
		S1_50423092
		S7_64211680
		S1_79616176
		S3_56093936
		S2_75364723
		S7_62851628
		S3_72063884
		S9_52612589
		S1_68236849
		S2_63440609
		S8_47566791
		S4_10584423
		S8_54218801
		S5_66787690
		S6_38609495
		S9_5861330
		S4_14983375
		S8_56781181

Catogoery	Total	SNPs
		S1_8502990
		#N/A
		S1_55302655
		S9_58335928
		S1_31205508
		S3_72592872
		S3_55951443
		S2_62315822
		S8_18372179
		S1_22396999
		S2_7055333
		S7_40119253
		S8_1839669
		S1_55563985
		S6_60722895
		S4_14590894
		S8_3230456
		S6_38516007
		S2_36673863
		S4_6125077
		S1_80478109
		S3_53397172
		S8_48976756
		S9_52419184
		S1_1640927
		S4_2263675
		S3_5617829
		S1_49572605
		S4_3781528
		S6_53672942
		S8_60337795
		S5_42904854
		S9_41580235
		S8_51309179
		S2_7287972
		S5_1184479
		S7_62354784
		S5_1455516
		S1_4107720
		S6_48682549
		S3_70823363
		S1_49924115
		S3_69541081
		S1_7069629
		S2_12357519
		S4_3828977
		S3_53583427
		S1_51011160
		S8_54710919
		S4_10717609
		S6_53881182
		S4_67082938
		S6_2712183
		S3_72125651
		S9_8256332
		S7_1799568
		S2_75658158
		S4_61244329
		S6_41366890
		S6_40526478
		S1_17276939
		S5_9537230
		S6_44606951
		S1_31205648
		S1_79401097

Catogoery	Total	SNPs
		S7_311470
		S1_11122135
		S3_71433053
		S6_49333610
		S2_4202794
		S3_62407039
		S9_3171624
		S7_6594048
		S8_16250682
		S1_59020596
		S7_51986740
		S7_1837929
		S8_60514275
		S2_9466693
		S3_59704855
		S3_61024847
		S4_27293084
		S2_58846525
		S9_1073120
		S3_69681288
		S2_30608879
		S2_68561511
		S2_68591097
		S6_77603
		S3_60884251
		S2_5623004
		S1_22266138
		S6_16398520
		S1_11708046
		S4_3811442
		S3_55005235
		S7_2771641
		S2_6036484
		S3_566940
		S1_56681151
		S9_53527840
		S2_76427679
		S1_54569270
		S1_11624239
		S1_4640366
		S2_69852912
		S9_4037873
		S1_6916528
		S9_1594436
		S2_62315730
		S5_65910362
		S6_50119229
		S1_26991276
		S6_48509199
		S3_19271060
		S7_53150038
		S3_2031830
		S2_11745922
		S5_4319473
		S6_50703413
		S5_5663603
		S6_54250474
		S9_1750361
		S7_62862454
		S1_56522888
		S8_41027745
		S1_9811171
		S3_62837711
		S2_62210279
		S9_59160864

Catogoery	Total	SNPs
		S4_2578145
		S9_998325
		S8_53262102
		S8_56665470
		S1_8981785
		S5_69893232
		S1_72524829
		S1_79793862
		S1_9058954
		S2_55050297
		S2_62480615
		S5_54655125
		S6_28280873
		S2_62019577
		S2_1030525
		S1_49414959
		S2_16000975
		S4_11520753
		S1_16552919
		S1_3988993
		S4_58313633
		S1_4067389
		S4_6084601
		S1_63253435
		S1_77385754
		S3_60583208
		S5_57788015
		S9_2672403
		S5_3182184
		S4_6214446
		S3_55310604
		S3_69219294
		S1_15276988
		S8_5442003
		S1_8975368
		S7_54697852
		S2_75804318
		S1_16387755
		S2_41116446
		S2_63570714
		S5_2278275
		S9_58472528
		S9_8457593
		S5_60718426
		S4_66273499
		S7_1839451
		S2_71569848
		S6_51114431
		S6_44562450
		S9_43967837
		S1_11127551
		S2_76431907
		S4_42467714
		S2_10525871
		S4_2002539
		S7_64105584
		S2_73494137
		S1_7389960
		S9_53172719
		S6_29942022
		S4_2894286
		S2_6799092
		S2_64899574
		S6_31172034
		S3_71247836

Catogoery	Total	SNPs
		#N/A
		S1_77765783
		S1_19696998
		S7_65097487
		S1_1584924
		S5_6868776
		S4_45883566
		S5_50559765
		S5_58856853
		S1_51130129
		S7_62463810
		S5_1052258
		S6_31742442
		S6_60774392
		S7_40210287
		S9_4249072
		S2_10788405
		S7_59752049
		S5_11114869
		S9_11644995
		S1_26398934
		S9_2871421
		S3_20127142
		S4_53671341
		S2_3405867
		S9_44061221
		S4_53390516
		S1_26945868
		S6_50309381
		S5_68758417
		S5_61792614
		S3_68057708
		S8_61925269
		S8_61127303
		S4_1609516
		S2_67205947
		S7_64102027
		S9_54316358
		S5_490735
		S7_60007124
		S9_8722179
		S2_4205896
		S2_60595349
		S4_62879368
		S4_48061089
		S4_50252300
		S3_56860596
		S3_73272613
		S1_67933625
		S1_80068400
		S4_53742397
		S3_62552106
		S8_59834697
		S4_1083566
		S4_68098063
		S4_25919290
		S4_66232876
		S6_42013191
		S8_16550866
		S8_10158124
		S7_6373331
		S7_60598750
		S6_7193509
		S3_57213859
		S3_58320516

Catogoery	Total	SNPs
		S3_52430346
		S1_50852755
		S7_62208068
		S1_72558801
		S1_7165155
		S1_18917007
		S2_16883443
		S5_24452329
		S8_31501526
		S4_5085176
		S8_55707656
		S7_51379747
		S9_50296416
		S1_26887908
		S1_74808525
		S1_26890734
		S1_51357369
		S1_12945064
		S2_12675922
		S1_66564577
		S1_17276958
		S8_55988013
		S7_63029070
		S2_71254362
		S3_55005050
		S9_4377961
		S4_4321312
		S2_75791858
		S2_61633784
		S1_59350604
		S1_22051849
		S3_55171022
		S2_16852191
		S8_50346890
		S1_60150320
		S2_8341847
		S3_52133606
		S3_13494213
		S1_58585480
		S2_68922140
		S5_11615604
		S9_59378888
		S4_8603807
		S6_2023784
		S3_70013909
		S1_9153558
		S8_5062027
		S2_58118883
		S7_17216515
		S2_65036625
		S7_58579945
		S9_4382754
		S1_60677610
		S1_6634852
		S4_5850189
		S8_55494923
		S1_7586041
		S9_49417096
		S3_70752062
		S6_844919
		S5_70826181
		S2_16479835
		S6_14493351
		S8_49317737
		S6_47055810

Catogoery	Total	SNPs
		S6_53215585
		S3_73506877
		S5_2204448
		S1_9811149
		S8_51332886
		S3_20022278
		S1_18055112
		S3_72322424
		S7_63118108
		S7_64105676
		S7_9515578
		S4_2273780
		S2_4554912
		S9_31608848
		S5_61780010
		S1_10871229
		S4_53778227
		S3_63172075
		S1_62745939
		S8_37542146
		S1_52223355
		S6_60926391
		S1_914852
		S3_70255279
		S1_59658675
		S4_53861441
		S1_18040569
		S2_8111630
		S2_8892365
		S4_37014494
		S2_68918203
		S3_71794784
		S1_61874941
		S3_58281927
		S3_16334531
		S1_9809434
		S9_3924412
		S4_23737067
		S5_68130982
		#N/A
		S1_70159353
		S6_45146992
		S7_65343697
		S1_79165404
		S5_61208217
		S8_53982176
		S3_27939455
		S1_56442697
		S6_52626687
		S5_62503572
		S3_13358847
		S2_39479672
		S2_56963293
		S3_73700889
		S1_60414741
		S8_37228098
		S4_10511027
		S1_71564441
		S7_65097648
		S5_67495270
		S4_1126296
		S1_56695762
		S4_3846693
		S7_1825207
		S8_5320760

Catogoery	Total	SNPs
		S2_57607885
		S5_63235244
		S3_57777052
		S7_58295565
		S1_78510628
		S3_68278877
		S7_56171577
		S2_12687037
		S1_73467704
		S1_67947279
		S1_18115987
		S1_8441804
		S5_59271718
		S2_57495650
		S4_9319662
		S5_66622061
		S6_56333598
		S2_7878236
		S9_53720715
		S5_6750762
		S6_52889165
		S1_14320058
		S5_63235224
		S6_44752306
		S6_60532241
		S7_62354781
		S1_21301163
		S7_52170373
		S4_33590898
		S5_68989200
		S9_5153823
		S2_68588399
		S4_55519657
		S1_8478388
		S2_6204339
		S5_6770039
		S1_72051998
		S2_18811083
		S4_52778245
		S4_63384415
		S8_3579334
		S1_7225953
		S1_65380830
		S6_44752142
		S1_77277544
		S1_65673461
		S7_6594205
		S1_79962417
		S1_6226993
		S6_48693483
		S8_1958130
		S3_4951086
		S1_72373902
		S9_11675301
		S1_63011671
		S2_71463473
		S6_8473156
		S7_41367008
		S2_65927037
		S1_7505872
		S8_5367403
		S4_53778218
		S4_9708759
		S8_61576897
		S2_6239311

Catogoery	Total	SNPs
		S8_50019427
		S8_44942821
		S5_61073377
		S8_53851139
		S3_4484787
		S6_45540276
		S3_62407421
		S6_48553889
		S3_2047922
		S2_61314205
		S3_56448069
		S8_2635771
		S5_59444708
		S2_64458232
		S8_46348607
		S1_13373588
		S7_5962639
		S1_60005641
		S1_14462417
		S2_60973431
		S8_4310186
		S1_71839392
		S8_61462479
		S6_47043880
		S7_64048256
		S5_8604532
		S4_3998846
		S3_3757324
		S1_60912506
		S7_56371046
		S1_11686439
		S1_67946894
		S1_55046553
		S6_51412610
		S1_79438076
		S2_5698066
		S9_8337272
		S4_1111843
		S3_73887740
		S3_72310647
		S9_52157998
		S2_68759199
		S2_59011224
		S6_48553933
		S7_63886758
		S6_58890927
		S1_78288219
		S4_9322342
		S4_37411979
		S2_12858147
		S3_56147104
		S7_482754
		S1_65255277
		S2_361178
		S4_53381294
		S5_12294697
		S2_68868783
		S1_10687799
		S7_7749141
		S3_68970854
		S9_3184859
		S5_9478874
		S2_61083423
		S7_57211490
		S8_53579113

Catogoery	Total	SNPs
		S5_62388455
		S1_8374221
		S1_64802896
		S8_49666347
		S7_62861763
		S8_46696678
		S1_74089757
		S1_52231130
		S3_52669518
		S9_4249065
		S1_63011664
		S1_12864949
		S1_865
		S5_11062125
		S5_55344499
		S6_51862032
		S1_12212663
		S2_68533927
		S5_67620448
		S1_78998932
		S3_51972035
		S1_67610048
		S8_18742941
		S1_58609370
		S1_19137191
		S5_12855816
		S1_68798270
		S3_48916657
		S5_67938441
		S9_6659655
		S7_54525595
		S2_68109209
		S2_64350080
		S3_60583143
		S1_58218750
		S1_57535872
		S1_2909514
		S6_55746377
		S6_34253823
		S3_48449330
		S1_78182503
		S3_56323631
		S2_7286785
		S1_58193573
		S5_38107237
		S2_6888205
		S5_16385304
		S4_52949060
		S8_33751926
		S6_35891529
		S8_53940550
		S1_4086596
		S2_65766043
		S2_76492690
		S1_7823000
		S1_71371594
		S4_6465595
		S6_60726944
		S6_25475937
		S3_5611628
		S3_46805444
		S7_9428572
		S4_5855192
		S1_8572176
		S1_59398263

Catogoery	Total	SNPs
		S6_47178970
		S1_57183497
		S7_6432618
		S1_14320017
		S6_632993
		S5_3084145
		S1_18046968
		S4_39712401
		S8_57039685
		S3_73303536
		S2_56963335
		S1_66564629
		S9_51705476
		S3_64343657
		S6_56333519
		S7_7830205
		S7_17479036
		S5_61168362
		S6_47105318
		S5_65772572
		S7_6432600
		S1_12757857
		S9_51890183
		S9_8190427
		S5_5366267
		S7_63988072
		S7_40355454
		S5_65930939
		S1_20207370
		S1_26887190
		S2_61334753
		S8_61980174
		S2_72265481
		S9_58723867
		S3_72455729
		S1_52755847
		S1_78528692
		S2_12746307
		S3_71423949
		S9_53748465
		S3_64853104
		S3_6722735
		S6_2152471
		S8_56411894
		S7_63076827
		S9_41578314
		S5_36169959
		S6_51873090
		S6_53298824
		S8_45095978
		S4_33876538
		S6_51864817
		S8_57571789
		S2_59795405
		S6_50317995
		S3_64873878
		S8_18800270
		S1_18041984
		S6_48168018
		S1_61976676
		S2_4581141
		S2_6396279
		S1_75531355
		S2_75347491
		S6_58530950

Catogoery	Total	SNPs
		S3_70281299
		S5_69822718
		S4_24706940
		S1_77323327
		S2_9638388
		S3_67534163
		S6_11790106
		S3_5501156
		S5_20185608
		S4_52778178
		S5_2629325
		S9_54908027
		S8_1929026
		S6_47785233
		S6_50435866
		S3_73338811
		S5_62503696
		S1_59566985
		S2_6235068
		S9_53170541
		S9_6568982
		S3_70119915
		S1_79507187
		S3_67080370
		S1_78319943
		S5_9502837
		S7_62271877
		S2_4051998
		S1_79194545
		S5_2968902
		S5_11706314
		S6_27662183
		S2_10872845
		S1_78126243
		S2_31217128
		S1_52256410
		S9_51521422
		S7_1113530
		S2_24269444
		S2_59878122
		S3_69420804
		#N/A
		S2_64072291
		S9_50178753
		S5_5349938
		S1_74084268
		S4_51737955
		S8_1234427
		S2_12734005
		S1_78895135
		S6_30192949
		S9_54584011
		S5_66997588
		S2_60678273
		S1_14672789
		S7_64061440
		S9_51522067
		S2_2888145
		S7_61282969
		S9_3049285
		S3_73046036
		S1_4122315
		S9_57575548
		S8_7427004
		S1_64803273

Catogoery	Total	SNPs
		S1_11081322
		S1_66723328
		S8_60557454
		S2_63355054
		S4_51774663
		S5_1577806
		S5_3181235
		S5_11056386
		S2_72317645
		S8_4717652
		S6_53240813
		S3_73160936
		S2_46683793
		S7_7998506
		S6_1520074
		S8_61651316
		#N/A
		S4_66243128
		S1_22035650
		S2_8672942
		S9_50616436
		S5_58857036
		S1_55049722
		S1_62180898
		S1_72860182
		S1_5707324
		S7_54376137
		S7_7733694
		S3_57320614
		S8_2656534
		S5_68758327
		S6_57409372
		S7_59004208
		S1_18695982
		S2_61029334
		S2_76475832
		S1_2518733
		S8_2543517
		S5_9537221
		S6_6448065
		S5_20868103
		S2_74636783
		S2_71569854
		S8_44938824
		S3_69485306
		S5_9537225
		S5_6919494
		S8_49957615
		S2_12746310
		S6_47538927
		S6_50330502
		S4_13912434
		S8_58029241
		S2_12670156
		S3_72104766
		S1_59824191
		S1_2279889
		S4_9110924
		S4_11520791
		S2_460641
		S2_69537134
		S3_52260812
		S2_36755239
		S1_10118797
		S4_52473624

Catogoery	Total	SNPs
		S1_3620429
		S2_62936493
		S1_80734092
		S2_10623471
		S9_6579460
		S4_27979491
		S1_72010222
		S9_54909150
		S1_77814793
		S6_2681015
		S3_69833582
		S4_58300470
		S2_16548428
		S6_13904274
		S4_44730242
		S8_46038448
		S2_12734165
		S9_51418477
		S1_64099331
		S1_56567731
		S1_22269395
		S8_54927854
		S7_15941789
		S2_65888501
		S6_53582924
		S4_2650390
		S4_52500628
		S1_78509746
		S3_70135318
		S1_30345042
		S6_53638853
		S5_66045618
		S1_61730040
		S7_1789071
		S5_66798059
		S3_6599661
		S6_47382994
		S1_1353637
		S4_35135646
		S1_22272035
		S2_23251834
		S7_54365349
		S1_72558542
		S3_57342081
		S4_51427822
		S2_54200664
		S5_11706245
		S1_14606359
		S6_2003411
		S3_1935985
		S6_38608654
		S8_53877514
		S7_1163207
		S9_58105715
		S4_788818
		S3_48916501
		S2_49470482
		S1_70246992
		S7_9690909
		S1_62967240
		S9_8022436
		S7_1837919
		S8_9589083
		S8_31228446
		S5_3179697

Catogoery	Total	SNPs
		S5_1583423
		S1_10885253
		S8_2082250
		S1_8154245
		S4_51688464
		S2_58202069
		S1_2467755
		S7_61501575
		S7_64604566
		S3_70547340
		S4_50567707
		S3_5659685
		S5_68898231
		S2_6396289
		S8_2050976
		S7_58156515
		S4_39141178
		S4_45602443
		S7_62552269
		S9_51417449
		S1_3013239
		S1_59791386
		S2_67383619
		S2_67226081
		S7_54708076
		S5_6769991
		S7_54775532
		S4_50736515
		S4_37459977
		S2_2836530
		S1_75861428
		S1_16440125
		S6_55690124
		S8_10114912
		S6_45600975
		S1_24472061
		S2_56134025
		S4_51072602
		S3_52669566
		S3_73700079
		S9_42812091
		S6_2152823
		S3_70245187
		S7_64100625
		S9_6364806
		S8_56428378
		S1_78894799
		S3_69633144
		S4_7890026
		S2_63692211
		S3_48673672
		S3_2379436
		S1_21849056
		S5_66661698
		S8_34540372
		S1_64156475
		S1_56408468
		S4_50568986
		S6_53240803
		S1_59142204
		S5_62172180
		S2_63584031
		S1_4153459
		S3_6045891
		S3_65348748

Catogoery	Total	SNPs
		S2_69840250
		S6_48294185
		S3_537805
		S3_73335315
		S1_78034800
		S1_7499639
		S3_70976159
		S4_56526561
		S4_66356796
		S9_58916303
		S4_47827356
		S6_49469739
		S3_19353232
		S1_59338088
		S2_60921531
		S2_5965524
		S6_47901259
		S2_75048369
		S7_10000523
		S9_8337208
		S1_64171964
		S2_34331060
		S2_75048456
		S6_51419986
		S2_60965624
		S2_56290162
		S8_3477096
		S2_76855910
		S6_26052806
		S1_11562516
		S2_69104795
		S6_45716332
		S1_68730138
		S5_61787105
		S9_3933614
		S6_12177791
		S2_68092232
		S2_55477998
		S2_58202071
		S8_45159416
		S1_7509735
		S5_12389553
		S3_71926506
		S1_66340749
		S5_13393872
		S7_62837586
		S1_3016994
		S8_1123281
		S4_2578086
		S5_58574421
		S9_1241065
		S1_12144756
		S4_7273365
		S9_58471729
		S2_12858138
		S1_1033383
		S3_60719551
		S1_52089010
		S7_60725213
		S1_10100825
		S1_1910197
		S5_406860
		S8_249260
		S1_61680190
		S1_6922279

Catogoery	Total	SNPs
		S4_6256357
		S6_47331616
		S6_28307374
		S4_8587462
		S8_54218798
		S1_1560489
		S3_72306742
		S7_1488682
		S5_12294706
		S5_11706216
		S1_49489317
		S3_58406492
		S8_51309641
		S4_6463211
		S5_8748913
		S3_72257906
		S9_52327210
		S1_9726166
		S6_14498586
		S1_55509762
		S2_6423692
		S4_5855190
		S5_41234551
		S8_2292762
		S2_10926684
		S1_22269442
		S2_8867778
		S7_52170080
		S2_8854395
		S9_45853298
		S8_58069282
		S6_51517798
		S3_56913043
		S1_67476980
		S3_16373401
		S1_21725516
		S2_56439158
		S7_41960286
		S6_51865336
		S2_4639913
		S7_17613464
		S3_6089212
		S5_13393873
		S1_17426835
		S1_16455720
		S2_72317060
		S8_56595212
		S2_68564053
		S6_51320530
		S2_66092488
		S2_60677970
		S5_4246165
		S3_67762463
		S5_58548515
		S1_67372483
		S4_5888648
		S6_47882276
		S9_41051133
		S9_42000167
		S2_14775677
		S4_56611001
		S8_35816692
		S3_72514904
		S2_29436993
		S2_61551250

Catogoery	Total	SNPs
		S1_64099353
		S7_64024313
		S3_69209208
		S1_68783477
		S6_1047755
		S2_13167152
		S1_33259084
		S8_11105815
		S7_2894038
		S3_61711137
		S5_2639873
		S3_52430349
		S1_77710663
		S2_67043922
		S4_63405381
		S1_5668231
		S4_8155704
		S1_9153558
		S3_61723070
		S4_14088447
		S6_50801621
		S5_69985221
		S4_55549151
		S8_16169826
		S5_42766717
		S4_2853245
		S5_65842218
		S6_47285824
		S6_51464001
		S8_10241858
		S1_7768801
		S3_57466679
		S4_55505250
		S1_76660782
		S2_50263721
		S2_75356062
		S4_51278079
		S4_56534234
		S5_61897795
		S6_54400506
		S4_4199656
		S5_4346972
		S4_14983378
		S3_58246494
		S1_11205601
		S3_72463705
		S1_11207853
		S9_217571
		S3_68326354
		S9_5036352
		S2_66842275
		S2_73675553
		S4_8709338
		S8_1238560
		S1_24765200
		S9_53301253
		S2_62117392
		S3_70255133
		S8_46608657
		S6_58575563
		S7_40497528
		S9_50014563
		S3_66763972
		S5_1966404
		S8_3380530

Catogoery	Total	SNPs
		S9_6366929
		S1_30269149
		S8_53271847
		#N/A
		S1_9066124
		S6_49691842
		S9_11613682
		S1_79659905
		S3_68503204
		S9_7755287
		S5_12715858
		S2_59237228
		S1_71371601
		S1_20207353
		S3_70121250
		S5_3829963
		S2_9725897
		S3_63301738
		S6_52715043
		S2_49432619
		S9_57629292
		S3_57223920
		S5_665444
		S9_55551577
		S2_64563635
		S1_57379034
		S4_8058079
		S5_58524900
		S3_70134865
		S9_56542551
		S1_8688139
		S4_66125782
		S1_10913734
		S5_7452677
		S2_4286577
		S2_58837190
		S5_4616791
		S3_71236513
		S5_1835240
		S6_53739937
		S3_1866321
		S8_61678930
		S3_58246379
		S9_5861361
		S7_63699592
		S2_66967843
		S3_66632155
		S1_27268030
		S5_62812146
		S2_4554977
		S2_52306757
		S3_62087659
		S1_24465870
		S5_63936712
		S3_71574429
		S2_8713295
		S1_66026941
		S3_52430347
		S4_1663523
		S2_8262851
		S7_887735
		S3_72827464
		S6_14160321
		S2_3759415
		S7_60752447

Catogoery	Total	SNPs
		S4_66357298
		S2_1319623
		S5_37494688
		S6_46372063
		S3_68038109
		S1_13343743
		S1_67329584
		S1_49433061
		S5_15793605
		S6_46735219
		S3_68701726
		S8_57440371
		S2_68059558
		S3_1905139
		S2_1533216
		S2_46916695
		S4_50755815
		S6_44560672
		S1_55917475
		S6_50659189
		S5_65524061
		S4_68098079
		S4_50975002
		S1_2432777
		S1_59015825
		S6_47063088
		S9_2228554
		S2_47634497
		S3_57776911
		S7_54573414
		S2_69154885
		S3_57882257
		S9_58742693
		S2_63676097
		S4_3919978
		S9_43769781
		S4_61207878
		S4_42859906
		#N/A
		S7_52290689
		S6_47614080
		S2_63511664
		S2_47145719
		S2_10791232
		S8_4123759
		S2_7055328
		S2_75613714
		S8_34887989
		S6_60698168
		S5_69783484
		S5_638420
		S7_1525131
		S3_47278241
		S6_53396939
		S3_57210381
		S2_10527395
		S9_52343052
		S3_64198004
		S9_53720463
		S2_40389289
		S2_56386758
		S6_17558400
		S6_28883215
		S1_15851565
		S9_3084006

Catogoery	Total	SNPs
		S2_6651556
		S5_6907417
		S6_47641319
		S6_47538952
		S2_67217200
		S4_56512771
		S5_63115656
		S4_66989693
		S9_52831923
		S3_48633170
		S6_53600028
		S9_50757963
		S2_125749
		S9_57678968
		S3_59320416
		S5_1575107
		S7_55982564
		S8_60169748
		S9_2228551
		S4_7052677
		S3_72851631
		S2_5649106
		S4_46534616
		S5_67297029
		S5_2985693
		S6_47841193
		S3_71247756
		S5_62388706
		S7_15942677
		S9_51890389
		S8_2653799
		S7_2494698
		S1_3564599
		S6_58761978
		S8_2624156
		S5_5041429
		S7_59048287
		S7_57195006
		S1_72558549
		S3_5643185
		S4_53742478
		S2_5791851
		S3_74310289
		S1_75619555
		S3_67966294
		S4_58254087
		S3_14324640
		S8_36950546
		S8_49844511
		S1_29401048
		S8_61879425
		S9_52642720
		S2_41116493
		S6_57407490
		S6_47072562
		S3_54260833
		S6_48594449
		S6_47323538
		S8_1504850
		S5_61749005
		S2_12005177
		S1_8265490
		S4_4236145
		S3_64940216
		S8_32825886

Catogoery	Total	SNPs
		S2_9821428
		S3_54930701
		S1_55046556
		S1_77861608
		S5_10489204
		S3_62571146
		S6_51334145
		S1_14515489
		S9_4365934
		S4_37693632
		S9_2931137
		S1_65559535
		S2_68126990
		S4_11112032
		S1_8504003
		S5_62729335
		S9_2531806
		S3_55801543
		S4_3734981
		S1_75683209
		S3_2381808
		S8_54739940
		S2_5392239
		S7_9671933
		S3_73704997
		S4_12881120
		S2_8047297
		S7_64102163
		S7_56589388
		S2_61742412
		S2_75672279
		S3_57928886
		S2_42945128
		S3_5164615
		S2_7513738
		S2_56916506
		S8_7197892
		S1_71994509
		S3_60936339
		S3_55345696
		S6_35884867
		S9_52554856
		S2_69858461
		S4_9447483
		S7_62611892
		S2_62765240
		S3_57760798
		S7_62909752
		S5_258611
		S9_1111017
		S1_72009819
		S5_10490436
		S8_53982120
		S4_6899202
		S4_23266495
		S1_73093714
		S3_62377604
		S4_7608897
		S5_62376362
		S6_47074354
		S8_54218800
		S2_6140018
		S4_63395493
		S4_24160071
		S9_58335931

Catogoery	Total	SNPs
		S1_10523854
		S4_8528736
		S1_78563689
		S2_19726974
		S6_53857098
		S5_12039071
		S7_60725165
		S1_52255934
		S7_56997685
		S8_58455774
		S7_15925687
		S8_50298927
		S4_24548814
		S8_1039140
		S9_58293266
		S3_71412134
		S2_45247793
		S2_3107266
		S6_54390552
		S9_50444618
		S2_75347519
		S1_56307730
		S2_64287855
		S4_51427889
		S5_1122660
		S4_4912446
		S6_18377387
		S6_51488239
		S8_50018669
		S3_71926494
		S1_65740373
		#N/A
		S5_3865173
		S2_71456623
		S3_70691325
		S7_4559740
		S2_76889763
		S8_38975003
		S3_69476817
		S3_61458573
		S1_57208283
		S8_3219287
		S9_52805993
		S3_71412131
		S4_16065237
		S2_12357525
		S4_62988573
		S7_5588604
		S3_59503624
		S5_62412833
		S5_62388560
		S7_60213933
		S1_60005631
		S1_77709387
		S8_57752792
		S9_47028388
		S1_48929808
		S2_58875673
		S1_78642331
		S5_7341796
		S5_58388185
		S1_71592625
		S2_61084036
		S8_53515832
		S3_54354139

Catogoery	Total	SNPs
		S6_47674553
		S1_72586815
		S3_72414661
		S4_53281130
		S1_74961861
		S5_9537227
		S6_30774666
		S7_1999335
		S9_217671
		S8_57569054
		S6_952545
		S2_59592071
		S4_19719450
		S2_1937784
		S2_59800156
		S3_71689899
		S9_3351682
		S3_66645809
		S7_42860197
		S8_3018133
		S5_11792504
		S8_55936322
		S1_66850667
		S1_56161547
		S6_53857091
		S3_68919628
		S8_53944705
		S8_60851784
		S1_69096005
		S8_59192410
		S2_75283503
		S9_42812131
		S2_3949788
		S3_15689043
		S1_14483401
		S2_12434689
		S7_574228
		S6_46735585
		S4_13847426
		S1_24472012
		S3_1935984
		S3_63353066
		S3_4576394
		S9_53933961
		S1_65291615
		S8_1753902
		S4_12409949
		S4_67970883
		S6_48041380
		S1_58332927
		S2_75364709
		S9_6872810
		S5_62729512
		S1_4144003
		S8_46035410
		S1_66652230
		S1_7313837
		S2_8370764
		S5_9566460
		S4_52473425
		S1_59444149
		S3_58878592
		S2_64382563
		S4_51276314
		S5_9483724

Catogoery	Total	SNPs
		S9_8190483
		S5_66622947
		S2_66599828
		S3_57363285
		S9_51831218
		S8_55700916
		S8_45538679
		S5_9542627
		S2_64350282
		S1_8739815
		S3_58280905
		S1_78579624
		S1_73467704
		S3_19996493
		S1_18853129
		S3_72789812
		S1_51417960
		S6_15381906
		S2_11737292
		S1_72772991
		S3_52457555
		S1_47396151
		S9_4790827
		S3_4606942
		S5_11328785
		S2_4380076
		S2_12746320
		S1_24602891
		S4_33238299
		S1_26932174
		S1_50423397
		S8_3491564
		S2_17616412
		S8_5105185
		S1_66565565
		S4_7273388
		S6_45147206
		S8_16424241
		S1_78530441
		S3_65682610
		S1_1610381
		S6_30193235
		S3_61026544
		S2_3707582
		S4_52778237
		S1_8820891
		S2_66967743
		S7_58333580
		S4_66158427
		S4_51082367
		S6_2003398
		S6_53268101
		S1_63458063
		S3_70538008
		S1_72721939
		S8_60394250
		S2_9663585
		S7_1163372
		S1_77821288
		#N/A
		S3_73704919
		S1_18954654
		S5_68825049
		S2_19461446
		S2_65580348

Catogoery	Total	SNPs
		S2_66132129
		S3_65325361
		S5_10690012
		S7_58846078
		S2_58017911
		S2_56135667
		S1_57110285
		S3_62418173
		S1_49964344
		S2_4272697
		S7_64695666
		S1_79151556
		S6_3689227
		S2_38489657
		S1_27833202
		S5_71466801
		S9_52932642
		S9_58699258
		S1_11117077
		S6_42241235
		S1_7624974
		S4_3811451
		S7_59056668
		S1_18923680
		S1_71389906
		S8_1691519
		S6_60460610
		S4_13858547
		S1_78715573
		S2_5833746
		S2_5873888
		S4_6757047
		S1_1862844
		S8_3183154
		S4_48038005
		S2_73037936
		S8_1924399
		S5_62493253
		S1_72372294
		S9_50015152
		S8_57424083
		S2_61964113
		S5_4321548
		S1_68236829
		S3_4518130
		S5_6166265
		S6_50326263
		S6_5110082
		S5_42766694
		S8_32606395
		S1_74896361
		S6_39121283
		S2_70913158
		S6_47674414
		S1_80077473
		S9_3765739
		S1_45631297
		S1_77338688
		S2_71458097
		S6_53592585
		S8_56144772
		S1_78955779
		S8_31744536
		S4_37131506
		S1_50619486

Catogoery	Total	SNPs
		S4_52062305
		S6_453009
		S2_63195569
		S5_1456219
		S1_6512004
		S2_75207558
		S1_73622450
		S3_65321019
		S2_61654415
		S4_53381285
		S3_11300676
		S6_50656465
		S9_1173302
		S2_59921489
		S3_71678089
		S2_9854735
		S8_31303429
		S5_65519267
		S1_65324962
		S1_18426178
		S5_2169036
		S8_60362306
		S3_55024364
		S2_43547808
		S2_10740794
		S6_2665926
		S6_45638391
		S1_67059673
		S3_73325587
		S3_6066226
		S4_6371807
		S9_57542596
		S1_6013250
		S2_75759060
		S6_51803591
		S2_7875744
		S4_7340748
		S1_48929830
		S3_74143378
		S4_52935690
		S3_55308186
		S7_62726698
		S6_53246557
		S4_51082422
		S9_48956443
		S8_51525606
		S9_44168126
		S4_67973185
		S5_8695166
		S8_58489585
		S8_1234744
		S4_42718452
		S1_29681005
		S1_21725540
		S7_63118109
		S2_15331772
		S6_54583231
		S7_38853739
		S5_4288898
		S1_63952977
		S2_57862903
		S9_56116132
		S1_59985051
		S7_6927640
		S7_64102026

Catogoery	Total	SNPs
		S4_1602640
		S3_55300411
		S9_50020075
		S1_63714618
		S9_2935604
		S2_55478016
		S1_10949866
		S5_12084207
		S6_50696124
		S1_72791248
		S6_6522636
		S1_58165695
		S1_77966174
		S2_2521663
		S3_55204441
		S2_69291228
		S2_76937009
		S3_72123657
		S1_50830148
		S4_5451145
		S8_459275
		S1_9756085
		S3_45801219
		S7_6432631
		S7_38853664
		S3_59627758
		S9_3620853
		S2_75085330
		S2_10290314
		S1_841821
		S1_72692144
		S3_60919672
		S3_54114709
		S1_4120973
		S4_8892964
		S6_60754183
		S5_2829050
		S2_65368149
		S9_43549710
		S3_52859571
		S5_59401333
		S9_52824030
		S1_75685766
		S2_71569856
		S5_41687209
		S3_6636677
		S5_14305627
		S9_59253271
		S8_54064586
		S2_67191693
		S8_40879069
		S3_71245279
		S4_1447464
		S1_55566870
		S2_65329464
		S4_54063205
		S5_12297387
		S3_69360827
		S3_66617443
		S5_65496371
		S2_75650927
		S5_61209466
		S1_12305229
		S2_19318606
		S8_47570414

Catogoery	Total	SNPs
		S1_3551532
		S9_9617435
		S5_43971814
		S4_3370584
		S4_4004792
		S7_64024318
		S8_55589573
		S1_4277393
		S4_56306416
		S4_67081812
		S5_3320559
		S7_7936961
		S7_60973123
		S9_58742695
		S1_75823447
		S1_15754236
		S3_4610735
		S2_61990723
		S1_66827680
		S1_7624968
		S5_67213533
		S8_57674740
		S5_6759353
		S2_63174773
		S2_60356896
		S3_3853842
		S1_15907945
		S1_72859887
		S6_921577
		S2_57862876
		S9_50238927
		S2_13459041
		S5_62473917
		S3_16334544
		S5_1184469
		S9_5829129
		S1_59895603
		S4_3761288
		S8_54226123
		S3_1612713
		S5_399584
		S5_67596024
		S6_38608643
		S1_79198463
		S1_78182550
		S9_2661097
		S3_56254556
		S3_60935357
		S8_2271665
		S9_3959206
		S4_68089530
		S8_57441910
		S9_54947163
		S4_51311743
		S1_59558280
		S3_7384438
		S1_55490924
		S1_19099908
		S3_66033734
		S3_70976162
		S5_5284999
		S8_36888141
		S1_79962409
		S7_63635637
		S3_54404438

Catogoery	Total	SNPs
		S3_720608
		S7_54708118
		S6_54617213
		S1_16455407
		S3_70276670
		S4_51479260
		S8_57478082
		S2_8715482
		S2_60289362
		S2_56134078
		S9_7538590
		S9_141097
		S2_8430565
		S4_7134861
		S2_8262796
		S7_63448180
		S2_16844205
		S1_66791920
		S4_51951352
		S2_62019570
		S7_63930001
		S1_20122903
		S1_60677886
		S1_17585672
		S1_57485756
		S7_14233723
		S2_59921324
		S8_49625562
		S2_16198598
		S1_79155251
		S7_54789325
		S2_62691832
		S8_54855513
		S7_2939199
		S2_61105465
		S9_53732068
		S1_79853251
		S2_69135792
		S3_68752970
		S3_68664323
		S5_12297370
		S1_6634684
		S4_52440845
		S7_62402682
		S1_66898274
		S6_22973927
		S5_62355340
		S3_620393
		S7_10629032
		S4_3776391
		S6_37382712
		S8_59192379
		S1_76491856
		S6_47817296
		S2_68542250
		S2_69038951
		S1_60849842
		S4_50670428
		S4_66239289
		S3_51516170
		S4_8012696
		S4_53052855
		S7_40134316
		S2_65493428
		S3_73184124

Catogoery	Total	SNPs
		S1_71174780
		S2_59955472
		S9_52471684
		S9_55004990
		S9_7843726
		S6_1454192
		S6_36790476
		S7_1288935
		S6_5727443
		S3_73610565
		S5_9181165
		S4_7147179
		S4_7169878
		S5_61852100
		S9_50595947
		S1_56852716
		S2_66339825
		S9_53547775
		S2_66633520
		S2_65226801
		S7_4452979
		S1_1346280
		S5_16084012
		S6_51799478
		S6_54236872
		S1_13359323
		S6_27279742
		S8_54927810
		S3_58381848
		S2_6396286
		S9_58070673
		S6_46748383
		S9_3059895
		S9_44164626
		S2_63811602
		S3_69810211
		S6_44777232
		S7_51986778
		S1_66310194
		S1_60349497
		S1_72303505
		S3_66034072
		S2_57258314
		S1_59567018
		S2_76535117
		S3_3910359
		S5_67938449
		S2_53438887
		S1_80308012
		S1_58634921
		S4_40194129
		S7_54371446
		S5_7902906
		S7_4559744
		S1_3156774
		S4_54102455
		S5_68187151
		S9_42350657
		S2_6751683
		S3_63301477
		S1_74119559
		S8_53828769
		S2_59226965
		S6_60774033
		S9_3026579

Catogoery	Total	SNPs
		S2_63378611
		S6_26214010
		S1_76495058
		S1_59338093
		S1_58035523
		S1_15897157
		S9_1075022
		S1_59469994
		S5_4006209
		S3_6215214
		S6_47071644
		S1_11122066
		S6_52285501
		S4_2302894
		S6_26110481
		S1_1010720
		S4_37656059
		S2_61859442
		S9_32091329
		S5_63408375
		S2_61083201
		S5_4355106
		S2_3881066
		S6_42092249
		S2_65855593
		S8_60139638
		S1_8644857
		S4_1054272
		S3_53048118
		S6_3441011
		S3_70840790
		S1_53480678
		S4_20032282
		S7_7678356
		S5_10354363
		S5_56047207
		S1_19727288
		S2_58017187
		S6_55686282
		S4_56426080
		S7_437597
		S4_54095462
		S5_13210526
		S2_65359770
		S8_2297433
		S3_48311839
		S9_44446305
		S1_74294389
		S4_1603553
		S8_50539096
		S2_10517558
		S1_50783345
		S1_77172764
		S2_10564436
		S1_73890726
		S2_6423699
		S4_6798754
		S1_74730873
		S2_58682751
		S8_47592574
		S7_39095683
		S2_68817589
		S5_1729058
		S4_66357270
		S2_2521659

Catogoery	Total	SNPs
		S6_17055044
		S4_11549194
		S1_80674355
		S2_64342421
		S6_53317481
		S2_61420747
		S3_69485304
		S7_1630105
		S5_3105032
		S2_9463955
		S8_1840287
		S8_3063986
		S2_9529763
		S4_39141246
		S7_58652748
		S1_74546925
		S2_67839037
		S2_56191917
		S4_13959607
		S4_48708959
		S2_76657365
		S2_4196996
		S4_54197424
		S2_59320036
		S2_65386762
		S2_12757478
		S3_60695169
		S9_5909349
		S3_54416434
		S5_68026340
		S2_4554926
		S2_17761104
		S2_11765738
		S2_7878187
		S8_57571916
		S7_52306551
		S9_52763078
		S9_3540381
		S2_72619522
		S1_11686467
		S3_20195489
		S1_71822695
		S2_75378560
		S1_65259511
		S2_62422107
		S2_61695406
		S1_18115963
		S1_2090106
		S8_9527483
		S1_21784561
		S3_62065706
		S3_60607348
		S6_8865341
		S3_57466950
		S9_8337283
		S7_62309668
		S7_2920318
		S8_15299834
		S7_58333432
		S6_27279774
		S3_53290322
		S5_10714970
		S7_62259788
		S5_61792534
		S9_5180910

Catogoery	Total	SNPs
		S9_51277755
		S1_72558752
		S7_4784254
		S9_217559
		S5_21849706
		S2_64144914
		S7_38853637
		S1_10469941
		S4_67078898
		S1_22638608
		S4_66347323
		S3_71236536
		S6_51328387
		S1_11205120
		S7_8507302
		S8_32316953
		S1_67052701
		S7_9073782
		S1_78453360
		S6_54462516
		S2_63282846
		S7_63318998
		S1_59114763
		S3_74068823
		S3_72849718
		S5_63115742
		S2_454171
		S2_64350265
		S1_61848129
		S1_21722213
		S2_58025291
		S9_1646186
		S9_50594694
		S3_59557675
		S4_33698437
		S8_3065469
		S8_61921581
		S4_51529444
		S1_59471499
		S2_6887603
		S4_24729186
		S1_67317630
		S1_14872733
		S5_11465069
		S1_75765647
		S2_60202691
		S2_42945123
		S2_13133088
		S4_7236696
		S7_62032320
		S7_64968890
		S2_58605747
		S7_57691559
		S8_2167878
		S3_56098305
		S3_57501027
		S6_60768699
		S8_58050878
		S6_49955993
		S6_3352163
		S8_1403598
		S1_61774931
		S2_60290408
		S6_48693499
		S8_3301850

Catogoery	Total	SNPs
		S8_58586734
		S8_55247636
		S5_69847946
		S2_55471724
		S3_20545498
		S6_44752212
		S4_37522546
		S1_3462160
		S1_2730709
		S4_2076437
		S1_61854016
		S4_39769652
		S1_67679858
		S4_38495065
		S4_23841932
		S5_56030666
		S2_7314975
		S1_71946407
		S9_49551667
		S2_8867790
		S2_1007456
		S3_71666502
		S9_58294676
		S5_12364953
		S1_2909495
		S3_51321137
		S5_9537231
		S1_78728262
		S3_13445823
		S5_61188837
		S2_62996590
		S3_57244167
		S7_61482601
		S4_50643364
		S4_9648533
		S4_66386100
		S9_57068847
		S2_75642712
		S8_57441918
		S4_46685532
		S4_1645543
		S4_772911
		S1_64802943
		S2_75944690
		S2_6687218
		S4_5107115
		S8_16936393
		S2_6687226
		S1_17468710
		S6_57256119
		S9_3133792
		S8_1699053
		S4_16075049
		S4_33590906
		S4_7277432
		S8_16358356
		S5_32150
		S6_4500257
		S2_75378576
		S1_63011665
		S2_14746481
		S1_79357310
		S3_1936126
		S1_20976572
		S6_48291365

Catogoery	Total	SNPs
		S8_60211751
		S2_65330841
		S3_68460876
		S8_61959689
		S4_10444671
		S4_32849548
		S1_63075419
		S8_9691511
		S5_3049774
		S6_40234839
		S1_77814798
		S8_58700479
		S7_8168076
		S7_55995560
		S7_59048275
		#N/A
		S6_37970636
		S4_27411052
		S4_52417599
		S1_16805243
		S3_60935421
		S9_8361619
		S3_67975420
		S3_57213908
		S6_2003408
		S2_57734344
		S8_61101056
		S5_9905613
		S2_55292384
		S8_41088770
		S5_69847257
		S6_53857085
		S2_75804425
		S6_2652635
		S4_7138372
		S2_18811082
		S2_58201076
		S1_66564552
		S3_68095983
		S6_49477872
		S8_3173814
		S1_56066536
		S4_12986002
		S8_2323733
		S7_64417966
		S7_51294810
		S4_54876525
		S8_3665061
		S2_66931668
		S3_69633686
		S6_51427943
		S6_49768870
		S7_62721886
		S1_59557490
		S5_63685002
		S4_26502905
		S8_55617475
		S2_71481130
		S7_6432638
		S9_2744729
		S3_69598992
		S2_66107145
		S5_50603257
		S1_29338645
		S6_53758258

Catogoery	Total	SNPs
		S2_71258753
		S3_4484789
		S1_8975380
		S5_8841908
		S5_66045622
		S2_59878118
		S1_60150374
		S2_62083253
		S2_10294006
		S1_57181619
		S7_2779279
		S9_52757931
		S5_9873649
		S1_7953720
		S5_62875093
		#N/A
		S1_12757855
		S7_55786360
		S5_66635546
		S2_61508077
		S5_50603260
		S9_1019780
		S1_57666297
		S4_8237354
		S2_16380626
		S1_9811596
		S6_49221314
		S1_22458254
		S8_49625555
		S4_9075792
		S3_66031914
		S4_8026608
		S4_58482630
		S8_59983552
		S7_54409637
		S3_60789109
		S6_51839021
		S2_72648274
		S1_77833415
		S1_68725331
		S3_53026827
		S2_13120405
		S6_44562429
		S6_921591
		S8_56420706
		S1_47338395
		S1_78538580
		S5_11779490
		S7_3643072
		S2_69858493
		S6_1555567
		S7_614087
		S8_5710535
		S4_61175060
		S8_1753893
		S2_6850379
		S3_70376021
		S5_6583698
		S1_3455516
		S1_16440032
		S9_51890216
		S3_16630554
		S1_4121233
		S2_6988446
		S3_69824237

Catogoery	Total	SNPs
		S1_68245228
		S1_67311194
		S3_70519727
		S2_8332145
		S4_5655922
		S1_60720903
		S2_1319660
		S3_74071858
		S1_80143810
		S2_56600019
		S3_52003891
		S4_8815513
		S2_66339828
		S3_66409416
		S8_45698695
		S2_59197474
		S6_25836217
		S2_40566617
		S1_2744767
		S4_11117663
		S2_9579746
		S7_60709293
		S7_63731976
		S5_67526736
		S2_76427889
		S9_53945860
		S2_74683667
		S1_20291228
		S1_3457964
		S2_64413828
		S4_52350718
		S4_39239639
		S7_2504050
		S3_6724761
		S5_65265059
		S6_53553041
		S2_61785593
		S8_43754009
		S1_79591310
		S1_15897246
		S6_13904269
		S6_54595816
		S2_71441366
		S1_10611334
		S2_61424055
		S9_54881837
		S1_50042424
		S3_55545771
		S3_51972076
		S8_49677487
		S8_59250886
		S2_67991002
		S6_46483046
		S6_1374707
		S2_12434682
		S2_51852242
		S7_53795496
		S1_4476977
		S2_465815
		S1_49363692
		S3_61476681
		S1_78672306
		S7_9746662
		S2_10791271
		S5_12715835

Catogoery	Total	SNPs
		S4_20672534
		S2_61658774
		S1_59791544
		S5_3621114
		S7_62716943
		S1_48929913
		S1_18972584
		S1_67674101
		S1_61736207
		S6_2016126
		S1_6773438
		S1_60823731
		S1_58913445
		S3_61327884
		S7_54813178
		S2_68826907
		S6_2016167
		S4_68098059
		S6_53253611
		S2_73743231
		S1_4086629
		S2_7315066
		S3_3915198
		S5_15497313
		S5_58303851
		S5_12857235
		S6_S6_54254330
		S1_65259171
		S3_71245316
		S8_57430417
		S1_57494137
		S3_72534688
		S2_60969241
		S6_14375980
		S1_8998679
		S8_61099615
		S2_65855357
		S7_40225584
		S4_9322342
		S6_42116542
		S1_11205151
		S1_71839394
		S2_6561884
		S2_61444403
		S1_79360999
		S5_5827139
		S1_8975372
		S2_2758327
		S5_1761158
		S9_53867593
		S4_12331532
		S5_472313
		S9_5167775
		S8_1108592
		S3_16373405
		S2_75343607
		S9_50040743
		S1_57661191
		S1_6537372
		S6_1316570
		S8_50310408
		S2_77175535
		S5_1224982
		S2_68809043
		S5_67495285

Catogoery	Total	SNPs
		S5_11706320
		S5_61598040
		S1_71946442
		S1_11819527
		S3_54489364
		S1_6061229
		S2_59247306
		S2_9905519
		S9_3968407
		S2_64144916
		S2_61085922
		S2_77499746
		S9_4743762
		S1_51031410
		S8_3219289
		S7_10564018
		S6_54710402
		S5_69419060
		S3_73319063
		S3_57232278
		S6_50717739
		S1_33325665
		S4_9379524
		S8_2194097
		S9_52865570
		S4_6415121
		S1_7462346
		S3_4610612
		S3_51516244
		S1_75662805
		S1_15324225
		S6_1235361
		S7_57817722
		S5_63374354
		S5_69419065
		S3_52428176
		S8_53262201
		S6_47571572
		S9_58978056
		S1_21722381
		S1_57740926
		S5_67427044
		S8_58572856
		S1_64546850
		S7_17425476
		S1_74880932
		S2_56916038
		S8_59751393
		S4_6520539
		S2_9725903
		S6_16728625
		S6_53396946
		S1_77343713
		S6_55684222
		S2_11706092
		S9_49056200
		S3_72066874
		S7_2894068
		S3_5659701
		S3_73362986
		S7_6029446
		S2_75672242
		S1_50852753
		S8_57541077
		S5_68758459

Catogoery	Total	SNPs
		S9_2327765
		S4_27253758
		S8_58152081
		S1_57371712
		S4_6756706
		S9_42815669
		S3_72635971
		S4_1647986
		S8_56505729
		S1_60150451
		S2_10525637
		S2_4900399
		S1_67294933
		S1_72611070
		S6_47179559
		S9_5184290
		S8_245953
		S8_59681067
		S7_54660863
		S4_67573540
		S7_62614825
		S8_3218211
		S4_52612639
		S3_620339
		S9_215050
		S2_61527767
		S1_59759828
		S6_53439492
		S4_16982033
		S2_62493944
		S6_60722880
		S2_73816088
		S6_45274074
		S1_16456940
		S8_61195478
		S6_45198519
		S7_52328191
		S4_66239281
		S2_12734006
		S4_11121842
		S6_58530977
		S3_6109652
		S5_71466824
		S2_50763530
		S2_10895522
		S1_18002189
		S4_7914790
		S3_58315057
		S7_62143566
		S7_8824313
		S8_3219406
		S2_8144407
		S3_71926402
		S7_64105569
		S6_47762092
		S3_62986074
		S2_62729830
		S3_15625147
		S8_514226
		S4_1339586
		S3_72184233
		S3_73086678
		S1_59457262
		S1_13288834
		S2_37444600

Catogoery	Total	SNPs
		S2_426283
		S1_11686456
		S5_11465082
		S5_69854875
		S1_55519796
		S2_10906680
		S1_7092710
		S8_5432627
		S4_52949041
		S6_54248055
		S2_60969225
		S2_67455102
		S1_11205240
		S8_55615251
		S6_35658116
		S3_73271499
		S7_15692691
		S4_37747491
		S6_53168415
		S5_61334977
		S8_5441916
		S4_54046734
		S5_58422917
		S2_63676074
		S3_5561225
		S7_63639562
		S5_66114506
		S8_48961333
		S5_9537716
		S3_16057353
		S1_68309753
		S4_8057911
		S4_19689617
		S6_2003396
		S1_80624564
		S6_2712184
		S3_56110897
		S1_65843788
		S1_21106713
		S2_58617380
		S9_58471621
		S2_10787455
		S1_76027178
		S7_10006161
		S9_50594735
		S3_20545415
		S6_56573143
		S7_5157195
		S1_7202617
		S2_3425374
		S2_16071354
		S5_67024379
		S9_51983892
		S1_10697806
		S5_66661629
		S2_4419329
		S4_52778276
		S5_9889720
		S4_51423098
		S3_73362182
		S3_58729796
		S2_7314978
		S1_9916837
		S1_75721758
		S6_45509834

Catogoery	Total	SNPs
		S8_57535166
		S2_75343611
		S7_60488037
		S1_48989360
		S6_332648
		S7_9512658
		S8_53817206
		S1_63875095
		S5_7986511
		S9_57060469
		S1_77300385
		S2_318718
		S1_7607399
		S1_73873379
		S4_1497818
		S2_76832015
		S5_1443779
		S3_5477467
		S1_77343703
		S2_39136029
		S5_2629516
		S1_4144194
		S9_1010993
		S1_57535859
		S2_3881058
		S2_58209113
		S3_65633877
		S4_15887702
		S7_62131323
		S2_60970294
		S3_54964312
		S2_70921790
		S3_59581338
		S1_78759225
		S5_11706311
		S5_20868138
		S2_18573483
		S5_10900194
		S4_51774662
		S6_46475882
		S2_65219168
		S2_63811601
		S8_3185594
		S2_68826997
		S7_59307281
		S9_4377820
		S1_67311177
		S2_45862968
		S3_47946700
		S3_56293637
		S7_52302832
		S3_73610686
		S4_61207835
		S1_16960804
		S7_61478774
		S6_53396445
		S1_10464374
		S4_6084600
		S3_38419867
		S3_52260830
		S9_52313918
		S1_71730469
		S5_38667495
		S3_4266443
		S7_65171560

Catogoery	Total	SNPs
		S2_64088672
		S3_72455728
		S5_62682248
		S4_54084053
		S2_59576783
		S8_17983641
		S1_60924175
		S2_4422106
		S1_80001174
		S2_58604315
		S1_18905096
		S9_58949348
		S2_2235767
		S6_47065248
		S1_80626842
		S3_53590157
		S3_67871977
		S4_46657057
		S1_73037129
		S8_1567185
		S3_64532576
		S9_8192348
		S9_684461
		S2_10893714
		S9_8337208
		S6_54216572
		S1_24472046
		S1_79028506
		S1_78784589
		S1_31206016
		S2_69858489
		S2_69341796
		S2_4362996
		S4_33082835
		S1_79203130
		S1_75428057
		S2_59226951
		S1_7620913
		S2_77175504
		S1_52089021
		S5_63375552
		S6_53023374
		S7_2794659
		S7_64118084
		S1_78715895
		S5_15793554
		S1_45629970
		S4_832304
		S8_5157404
		S3_74054139
		S1_14320018
		S1_59562692
		S7_60796683
		S7_1962498
		S4_14235594
		S1_75699473
		S3_69885928
		S2_59247357
		S2_60791209
		S7_63066129
		S4_5850179
		S1_49433066
		S2_10832270
		S8_50539062
		S3_5065096

Catogoery	Total	SNPs
		S5_10635111
		S5_63235234
		S2_40078265
		S1_15851565
		S1_71640829
		S7_61282981
		S6_46786141
		S7_4745759
		S8_1341548
		S1_65672063
		S6_58831834
		S1_9701927
		S3_73874716
		S3_6278977
		S3_69937571
		S5_66191252
		S3_52995596
		S3_55801546
		S1_22051870
		S4_26389676
		S2_66341464
		S9_49909549
		S4_66125790
		S1_79576173
		S5_61990563
		S3_64447053
		S2_62997034
		S4_54041657
		S2_75689620
		S9_9038520
		S3_73325685
		S3_13241923
		S7_437396
		S8_58274259
		S9_7314204
		S1_59543906
		S2_7315359
		S9_58576046
		S7_5379609
		S4_63426488
		S5_65524064
		S9_57892385
		S3_65325182
		S2_62303166
		S2_67783981
		S3_59312944
		S5_10354374
		S9_1581180
		S8_49406639
		S8_61598424
		S1_15035499
		S1_9066111
		S1_10087620
		S1_49307780
		S9_59155261
		S7_7631218
		S9_1202165
		S3_69900008
		S1_62492709
		S2_1475386
		S6_41265311
		S4_54867180
		S9_55004998
		S3_69496539
		S1_21886694

Catogoery	Total	SNPs
		S2_41676142
		S2_3760427
		S3_55306487
		S2_61786337
		S2_75944694
		S9_2672406
		S3_49016157
		S8_59578887
		S2_2766843
		S1_72010107
		S3_54745131
		S3_71247759
		S1_11562517
		S1_61774983
		S2_52993698
		S1_7403656
		S7_54222397
		S7_54332945
		S7_60616810
		S1_9822499
		S1_71592626
		S1_74830318
		S1_66723316
		S6_48097499
		S5_38727157
		S6_13269947
		S1_59441710
		S1_72724752
		S2_62726243
		S1_63390309
		S4_14236318
		S2_69104796
		S1_52349781
		S9_4207682
		S8_36950441
		S2_53473317
		S2_45678323
		S2_74861830
		S1_19349242
		S7_63118564
		S1_80077551
		S2_8672941
		S1_63711620
		S9_58752954
		S7_54812068
		S6_60698167
		S6_952089
		S3_55062974
		S9_4419125
		S5_43080944
		S9_50536948
		S8_56504331
		S2_66606254
		S3_61901758
		S5_61102667
		S9_55067167
		S4_5107559
		S9_58471728
		S1_20086138
		S3_56255574
		S2_14168619
		S6_46381611
		S1_2740188
		S8_59765503
		S2_10378146

Catogoery	Total	SNPs
		S9_41358006
		S6_77547
		S4_35760599
		S9_10176701
		S8_61653281
		S3_49016138
		S3_67001463
		S2_4029571
		S1_26894176
		S3_60582763
		S1_68732721
		S1_66828707
		S3_53292798
		S6_44853188
		S1_8819456
		S6_46533127
		S2_73941972
		S1_57110290
		S8_57974128
		S9_5176021
		S1_52921005
		S2_73693384
		S5_66459550
		S3_61384437
		S4_9075839
		S1_20207356
		S1_16746045
		S1_4472261
		S8_16452618
		S4_1008292
		S2_10269489
		S8_1239079
		S6_3046256
		S5_67495275
		S2_36736707
		S2_4554897
		S1_59886728
		S1_16387980
		S4_42707669
		S8_55144785
		S4_46960996
		S3_4956343
		S6_47045062
		S8_36606229
		S7_54411324
		S2_50071217
		S7_2559963
		S3_69289582
		S2_66955986
		S3_71606853
		S6_47697910
		S2_67330587
		S4_66273498
		S8_55930246
		S3_58246629
		S2_39598805
		S3_69631048
		S8_60549078
		S2_34609313
		S7_12071794
		S2_61148235
		S6_53013283
		S2_65965883
		S3_72851798
		S9_1202128

Catogoery	Total	SNPs
		S3_57759697
		S8_3179565
		S4_5530921
		S5_4702991
		S1_16415208
		S5_32143
		S2_2758322
		S2_60882833
		S1_72938587
		S4_8012699
		S3_73160935
		S1_21178726
		S1_10687737
		S1_49256196
		S2_69315750
		S8_9589216
		S3_70581408
		S4_1337783
		S1_75542056
		S4_52500355
		S1_71730228
		S7_890901
		S7_1839455
		S5_7006744
		S3_59585312
		S4_5073866
		S5_10812291
		S2_13177269
		S7_10677566
		S1_11453221
		S1_72206067
		S1_75843677
		S1_66824882
		S2_71438162
		S1_28214656
		S6_49518315
		S1_1014
		S4_5085065
		S1_78642412
		S1_61659837
		S8_61545606
		S6_5727420
		S1_65273072
		S5_2619018
		S2_66372842
		S3_69847126
		S6_47158818
		S4_42280384
		S3_58901003
		S2_49582154
		S4_5850065
		S7_37667464
		S7_2621601
		S6_48605539
		S2_76859956
		S7_64424595
		S4_20584940
		S8_12577073
		S1_3420171
		S3_13433976
		S4_66125724
		S5_65772389
		S2_63674932
		S4_32984975
		S8_49625284

Catogoery	Total	SNPs
		S2_76887916
		S7_62851785
		S4_52986215
		S7_60709312
		S3_74131816
		S1_59444146
		S8_61917057
		S4_4207661
		S1_3150442
		S3_68835494
		S1_67562306
		S9_51418518
		S8_47828883
		S9_84468
		S3_68034233
		S1_80852685
		S6_36255774
		S7_8824146
		S1_51841718
		S7_55667982
		S2_72984208
		S8_48957431
		S3_4811513
		S4_4675143
		S3_55142243
		S2_10293959
		S2_69714842
		S6_53396941
		S8_47690808
		S1_53476313
		S3_19275042
		S9_3913758
		S8_61636546
		S3_68237783
		S8_1397395
		S1_S1_64604403
		S1_6512009
		S2_76656093
		S1_8644847
		S8_47828466
		S2_59948007
		S6_40930349
		S3_69847057
		S9_4637485
		S6_50704056
		S3_66031420
		S8_61951457
		S9_1258900
		S5_66245112
		S3_26476100
		S2_19732777
		S7_65086691
		S7_60260629
		S3_65403570
		S9_5931341
		S7_2491548
		S9_1188169
		S2_64413833
		S2_6178166
		S1_8502996
		S3_53210609
		S2_56692305
		S1_8568799
		S1_57133560
		S9_50014563

Catogoery	Total	SNPs
		S1_5892116
		S7_9289050
		S5_66663
		S9_52243881
		S4_25673975
		S1_68092920
		S2_17999597
		S9_1675487
		S7_58198808
		S4_55549145
		S5_62418971
		S6_47436654
		S2_3756122
		S1_13891951
		S4_7277331
		S5_63277836
		S2_61153419
		S5_67404861
		S1_58205051
		S6_31500864
		S2_67807370
		S1_73640848
		S7_53795538
		S5_5154168
		S1_66101361
		S3_56659055
		S2_55036954
		S9_42815569
		S1_27354797
		S8_520783
		S8_57948154
		S9_53171048
		S9_53183638
		S1_6985707
		S2_2521663
		S1_77861611
		S6_34559216
		S8_56642725
		S1_79194083
		S2_65076050
		S7_59956022
		S9_1110278
		S9_53229796
		S2_4581144
		S2_37573825
		S4_5107252
		S1_51003637
		S9_8722180
		S7_62494220
		S9_41543600
		S7_56029645
		S2_57444453
		S4_9434616
		S3_S3_625889
		S4_3813069
		S9_53554586
		S1_49232151
		S2_4363053
		S8_2195943
		S1_14543857
		S2_6684745
		S1_23892335
		S2_2753825
		S1_11205157
		S3_6373771

Catogoery	Total	SNPs
		S8_36934620
		S1_21951773
		S9_1176579
		S1_59892801
		S9_9596228
		S6_40868944
		S5_11062815
		S1_79438068
		S6_16400006
		S7_12761898
		S2_25098838
		S3_62273289
		S2_6012283
		S3_71574443
		S7_434761
		S7_6975469
		S1_16552916
		S1_8975379
		S6_48548069
		S6_51990257
		S9_52381116
		S1_21226206
		S9_19284693
		S2_2323466
		S8_42214820
		S9_217550
		S9_50884117
		S2_61595945
		S7_64750096
		S3_57750447
		S3_13358838
		S7_6632760
		S4_6125160
		S1_66309677
		S6_5752661
		S3_53295639
		S2_11629159
		S8_3480407
		S2_76833116
		S6_49842086
		S2_30234065
		S2_10330100
		S1_79961947
		S7_65097640
		S9_45361845
		S7_6679582
		S5_9695625
		S5_636512
		S1_74530777
		S8_56115061
		S3_51259353
		S7_1163196
		S4_26502907
		S7_65097488
		S3_70879391
		S8_49156114
		S3_52995539
		S2_60678276
		S6_40724114
		S1_12932610
		S5_4509043
		S1_10181337
		S7_60580566
		S5_65523629
		S5_62417140

Catogoery	Total	SNPs
		S3_69365322
		S1_3911606
		S5_11781311
		S2_73693382
		S4_51951356
		S1_14860763
		S9_5342152
		S3_6737465
		S9_49015597
		S5_3393162
		S6_46372048
		S2_67191690
		S2_59619957
		S5_6809702
		S2_6239308
		S6_26301521
		S1_1913204
		S4_56200684
		S7_57058365
		S5_68053717
		S4_24568412
		S1_59456579
		S2_61785834
		S9_6694506
		S4_54086420
		S8_3217766
		S6_6313777
		S4_1734540
		S6_53290691
		S6_41603254
		S6_50876032
		S2_6988395
		S2_66107577
		S1_63458065
		S7_59023258
		S5_1795457
		S1_12292614
		S1_15017476
		S1_55490884
		S5_2245607
		S7_2928583
		S1_59398818
		S6_55158791
		S1_67679324
		S1_16641373
		S8_4402281
		S4_52219850
		S8_57560277
		S9_44207227
		S2_63350493
		S3_48916483
		S3_60771077
		S9_3002317
		S6_4073738
		S6_41007970
		S5_66709108
		S1_72767177
		S2_60666672
		S2_6045000
		S2_61739812
		S3_3784661
		S5_61168151
		S2_11981316
		S3_5787681
		S3_55300427

Catogoery	Total	SNPs
		S4_8965192
		S7_62475317
		S3_69420805
		S2_21219485
		S3_4956350
		S7_56589402
		S1_66831013
		S6_40434372
		S1_74064538
		S9_5159832
		S4_989032
		S4_63124634
		S7_5174324
		S4_1004444
		S9_1115502
		S9_51178124
		S2_73743249
		S4_20144976
		S7_2771719
		S2_74899844
		S6_54381382
		S9_42061712
		S9_59340341
		S8_57738490
		S4_53777826
		S2_64382569
		S2_64144933
		S3_73984401
		S8_55729907
		S1_59020541
		S9_53929823
		S2_32921283
		S7_8168127
		S9_57575501
		S7_6594089
		S3_67762399
		S7_53162770
		S7_2625827
		S8_60130799
		S3_5130521
		S6_52369058
		S6_52691695
		S7_1489834
		S1_11209103
		S5_2204449
		S1_61875673
		S1_79161789
		S4_5018934
		S4_1778430
		S9_52795697
		S2_64589836
		S8_50612396
		S8_3264485
		S2_467615
		S3_13434100
		S8_60933913
		S3_56671287
		S4_24767372
		S9_55626966
		S3_71358565
		S1_12922230
		S5_65650624
		S8_51322924
		S6_41861965
		S6_50925294

Catogoery	Total	SNPs
		S8_4132787
		S7_57053067
		S7_64095478
		S1_51860543
		S1_8973949
		S3_6242206
		S8_4310149
		S7_9418008
		S1_55000243
		S9_51979200
		S4_13990882
		S9_47218885
		S2_57255731
		S2_57607777
		S1_6336349
		S3_68278905
		S7_61414424
		S1_72488308
		S4_44467255
		S4_47808747
		S8_60452270
		S9_53959940
		S8_3018098
		S2_72604960
		S3_4484811
		S6_18072558
		S3_53583413
		S5_65852089
		S1_78496211
		S1_6462600
		S7_63639767
		S4_19526458
		S9_215196
		S1_10593920
		S3_72184031
		S1_26239923
		S7_61642838
		S3_73160950
		S8_60911871
		S7_64105676
		S7_57111428
		S1_71592630
		S8_2374756
		S3_68263778
		S1_79478669
		S3_5202239
		S1_17106162
		S1_56695761
		S5_62754779
		S4_41413045
		S3_60581674
		S4_12419458
		S9_7342723
		S1_77870695
		S6_52639129
		S1_7624959
		S8_55571192
		S8_4993941
		S5_63070180
		S4_50670393
		S5_13319218
		S3_68983657
		S1_12969783
		S2_69714714
		S4_66114140

Catogoery	Total	SNPs
		S9_47005993
		S1_12305613
		S8_43753972
		S1_10162554
		S5_6750570
		S2_59610880
		S7_62966576
		S2_19728059
		S3_58281914
		S6_60460613
		S2_56870778
		S3_1945467
		S1_64803265
		S1_12592985
		S4_34368753
		S2_17761464
		S7_54683392
		S1_1033263
		S9_57780942
		S2_61435634
		S4_1053904
		S1_14438466
		S8_51719731
		S2_57258234
		S2_8887248
		S6_51187509
		S6_56302385
		S7_54797173
		S8_62110181
		S9_7351120
		S3_57375571
		S5_18589869
		S1_66898262
		S6_1047838
		S6_5575659
		S3_57929031
		S1_66650940
		S1_75521013
		S3_55049879
		S1_54021152
		S2_73193888
		S5_3621149
		S3_63073955
		S3_3757331
		S4_51648578
		S6_45647681
		S2_71634028
		S2_10905189
		S1_59444102
		S2_6396277
		S3_3480327
		S4_39961102
		S2_59738106
		S9_55890654
		S9_52508279
		S9_55837459
		S3_58878585
		S1_64159171
		S2_59657176
		S3_56003725
		S9_57905473
		S2_57705157
		S3_3480350
		S9_58926884
		S2_8888482

Catogoery	Total	SNPs
		S8_2374785
		S8_56112483
		S1_26240466
		S6_35584030
		S5_3397174
		S6_1279628
		S3_66051131
		S6_53639556
		S8_53214568
		S7_59026437
		S2_10527249
		S6_45544917
		S1_56295005
		S8_61876804
		S4_42263905
		S4_2306059
		S5_12147217
		S6_7193486
		S1_9485124
		S4_2233468
		S1_7992826
		S6_53253668
		S6_1374803
		S6_47101054
		S9_50009009
		S8_3366419
		S2_68059572
		S7_273075
		S5_63909553
		S1_29947527
		S1_68783521
		S4_19797059
		S1_1346263
		S3_57267443
		S3_18211926
		S1_57919321
		S5_8068932
		S2_10331508
		S1_18047014
		S4_2643672
		S1_12758279
		S1_18876428
		S3_57122013
		S1_59471494
		S7_9497070
		S8_45095927
		S5_50603256
		S9_3741359
		S1_68772896
		S1_60898437
		S6_50339577
		S1_67667926
		S8_54446720
		S4_20618385
		S6_47765301
		S1_76962293
		S6_52054314
		S5_68929153
		S1_65255041
		S2_72264271
		S7_58543527
		S3_52447823
		S2_75954404
		S7_62377709
		S3_13494232

Catogoery	Total	SNPs
		S3_61246805
		S5_69847943
		S8_48161909
		S1_60835052
		S3_56085489
		S2_6611935
		S3_62385589
		S1_18890747
		S1_2914728
		S4_67078902
		S7_58534236
		S3_4266391
		S8_60929822
		S6_54248147
		S3_14145258
		S3_69847298
		S7_62925024
		S5_62197722
		S1_7521184
		S3_60583150
		S5_9422820
		S8_3579328
		S1_6034160
		S1_71988273
		S1_53018631
		S6_175282
		S3_68130495
		S1_12945053
		S6_45006706
		S2_62264904
		S8_50612255
		S4_66386097
		S6_6448053
		S3_63388708
		S9_54086122
		S1_28124190
		S2_12670121
		S2_8229201
		S1_30708538
		S4_46534642
		S8_1928994
		S9_57874351
		S1_60877379
		S2_68931679
		S3_71652712
		S9_44042677
		S2_63811594
		S1_68783489
		S5_62385078
		S2_9672855
		S2_5869586
		S4_3797939
		S5_61187219
		S7_2616744
		S3_2379499
		S3_34579421
		S6_44551778
		S2_4554979
		S6_48686948
		S2_66956886
		S7_57227797
		S2_6221824
		S2_59125971
		S4_2626983
		S5_69780347

Catogoery	Total	SNPs
		S6_47432981
		S2_69151523
		S2_68060348
		S9_58935200
		S8_4402234
		S2_64413824
		S1_12837777
		S2_73366260
		S6_47954518
		S5_66684
		S2_75808124
		S9_5036378
		S3_70121236
		S7_39920848
		S1_20118144
		S5_61792608
		S7_53994045
		S5_1945863
		S7_18284296
		S6_8919203
		S6_38609510
		S2_44179858
		S6_28029606
		S7_15960434
		S5_12536512
		S1_78509961
		S1_63952974
		S1_80001211
		S5_2693093
		S7_57566800
		S1_7639596
		S3_51995693
		S3_69832091
		S9_55626950
		S1_10949880
		S6_53235416
		S2_49432601
		S3_15435904
		S5_63852003
		S4_46408295
		S6_47928174
		S2_65774199
		S2_9659482
		S8_60513478
		S1_18563299
		S1_12730872
		S3_62717648
		S6_8705589
		S2_52364662
		S1_53481344
		S9_51887661
		S8_47768635
		S5_13389973
		S1_13401163
		S6_47817289
		S8_54796806
		S9_52551803
		S7_60723643
		S8_13372533
		S9_54064272
		S2_44495698
		S9_55573838
		S6_48097408
		S4_48059837
		S1_74876107

Catogoery	Total	SNPs
		S7_62335137
		S4_10430680
		S6_45570612
		S1_10687809
		S8_59250720
		S6_25874630
		S2_19797997
		S4_51611510
		S8_3224948
		S6_1325919
		S1_49552257
		S9_3741354
		S2_6999171
		S7_60060824
		S4_56566643
		S9_53925520
		S9_1636348
		S4_9633340
		S2_56514927
		S7_3455863
		S6_1126722
		S5_69774352
		S1_72691386
		S3_71247686
		S9_214864
		S1_65259180
		S9_5861424
		S9_51559555
		S6_27218995
		S2_56085787
		S3_53546998
		S3_60619614
		S5_9180596
		S3_59754095
		S5_6766959
		S9_6569012
		S9_8190314
		S6_55277731
		S1_75594893
		S6_27965499
		S2_6881881
		S7_43128424
		S4_16367411
		S3_74119505
		S4_58181420
		S5_51255895
		S9_7342764
		S9_58471622
		S6_19445139
		S7_58690963
		S1_75390763
		S8_60933911
		S8_3675341
		S6_47955705
		S7_16019593
		S1_73585205
		S8_62107766
		S4_4541645
		S9_44575473
		S9_53172712
		S7_54573342
		S9_55067185
		S6_61056109
		S1_55566910
		S9_6659348

Catogoery	Total	SNPs
		S2_61859315
		S5_69822764
		S2_19732773
		S4_8721875
		S3_72534685
		S5_9501060
		S2_5489023
		S1_80482775
		S5_69854771
		S6_2560603
		S1_19642475
		S1_69959560
		S1_77795933
		S4_1042535
		S1_11272629
		S3_73765870
		S3_56448022
		S3_19564528
		S7_6766164
		S3_73215350
		S5_68797764
		S6_19232571
		S2_64518388
		S9_1258802
		S2_73375991
		S7_62686455
		S1_63011667
		S2_1954851
		S4_5107400
		S5_12614031
		S7_59751994
		S3_57340163
		S2_3774964
		S1_18113851
		S1_56407923
		S1_78749829
		S2_63294615
		S6_48155223
		S3_71678131
		S3_59633265
		S6_54611789
		S8_58151605
		S4_7562125
		S2_74869932
		S4_67087238
		S2_63384724
		S2_8235863
		S9_57152530
		S4_5894342
		S4_7333902
		S5_S5_49760004
		S9_52355443
		S5_59401311
		S3_54098274
		S1_62825660
		S2_72317058
		S3_52014442
		S9_5374228
		S3_65328162
		S4_50481512
		S2_65280424
		S5_11792508
		S2_6396281
		S6_42206654
		S1_79614721

Catogoery	Total	SNPs
		S1_71822725
		S3_62205651
		S1_79194823
		S4_7468079
		S6_51419894
		S2_57734381
		S6_53240810
		S3_45571080
		S8_2374791
		S6_31010280
		S8_4934942
		S4_3775185
		S4_8603111
		S6_56572627
		S5_4494472
		S3_6212246
		S7_6326967
		S1_17585592
		S9_2984314
		S2_10926679
		S5_12294705
		S2_S2_74010195
		S8_4491947
		S1_8918096
		S4_51985086
		S2_76913341
		S6_47065481
		S3_64649173
		S4_11132722
		S1_78743822
		S2_59249250
		S1_23956768
		S1_60414696
		S5_61102660
		S1_68301238
		S2_3641651
		S2_56916768
		S6_48693500
		S8_59879040
		S3_68919619
		S6_40126340
		S9_8256333
		S1_79961931
		S4_4754708
		S7_54322509
		S1_66643373
		S1_58035753
		S7_14232506
		S6_53177479
		S6_50576072
		S3_63632407
		S7_57643218
		S3_72659678
		S9_51890204
		S1_66791936
		S7_62126814
		S3_13362565
		S1_19069263
		S1_7225959
		S1_7810863
		S5_13320890
		S3_57795985
		S1_54392965
		S3_13649684
		S3_2112873

Catogoery	Total	SNPs
		S5_61944699
		S6_58178549
		S6_42350723
		S9_1241068
		S3_70242205
		S8_3691560
		S8_46410403
		S4_4991034
		S7_54939251
		S1_73514139
		S3_66415961
		S2_62118214
		S3_73160928
		S5_64011563
		S4_8017371
		S7_63060451
		S4_62923586
		S2_60970191
		S4_1703495
		S3_59637728
		S5_4870216
		S9_51521884
		S1_20087531
		S2_62264946
		S7_62271706
		S4_44730234
		S8_61615363
		S9_50744163
		S4_18594629
		S1_52089028
		S3_72440279
		S2_75720419
		S2_65043002
		S9_1071491
		S4_836922
		S8_54956239
		S2_64334144
		S3_3517493
		S7_6761409
		S5_63307347
		S6_47074433
		S1_64640035
		S1_19748639
		S2_60965617
		S4_2317163
		S9_1675494
		S8_3579334
		S1_20178900
		S1_72593131
		S2_2758328
		S1_77346170
		S6_54302368
		S8_55596035
		S5_66650990
		S8_57982271
		S4_25562082
		S2_392459
		S4_7563746
		S7_58526183
		S2_65107939
		S2_2901644
		S5_S5_49814039
		S5_65198578
		S7_61407617
		S5_9501145

Catogoery	Total	SNPs
		S1_56824560
		S1_72692120
		S2_67191648
		S5_9500800
		S7_36629285
		S7_59393553
		S8_50863469
		S2_60448440
		S2_76820831
		S4_20267236
		S2_12670180
		S7_61993332
		S6_54759103
		S3_69734204
		S3_60581681
		S1_56030210
		S3_57237992
		S1_7012936
		S4_2650392
		S7_38852322
		S1_71371613
		S8_6325918
		S5_62379418
		S2_75151126
		S2_67195407
		S1_78816216
		S3_57267602
		S5_61125541
		S9_52313744
		S3_54095474
		S8_2429042
		S4_54046224
		S1_76240937
		S4_5043343
		S1_8821784
		S2_58118896
		S7_2771587
		S5_61792005
		S2_65424895
		S4_44730218
		S7_64959364
		S8_37007706
		S1_12588454
		S6_58548261
		S9_S9_54280264
		S4_53812271
		S1_71831802
		S2_61153278
		S1_71998256
		S9_52880129
		S2_9725904
		S8_54959221
		S9_4037878
		S2_62493945
		S7_63618748
		S1_67311195
		S4_9434714
		S6_40434375
		S9_217549
		S1_16439922
		S2_40920935
		S1_19552536
		S2_67011044
		S3_55300421
		S7_51990916

Catogoery	Total	SNPs
		S2_59561870
		S2_76607095
		S7_2052615
		S5_69780729
		S9_10597714
		S2_6036251
		S8_3231060
		S4_7922554
		S2_53121078
		S3_51904673
		S2_65335659
		S1_65331498
		S8_1776400
		S4_12344560
		S2_56515274
		S3_73319060
		S1_63952276
		S9_54592509
		S8_48981868
		S6_51217660
		S4_388727
		S7_41144489
		S7_63889653
		S1_2312274
		S4_2641231
		S2_73675539
		S8_49124328
		S4_8027035
		S8_1252515
		S1_9002255
		S3_71519736
		S5_4130680
		S4_1004444
		S7_58774568
		S2_2888127
		S4_56306384
		S7_64366453
		S3_5751852
		S8_49977609
		S8_407461
		S6_51803412
		S2_12746316
		S5_66386669
		S5_4526596
		S3_11300663
		S4_7780853
		S5_8003227
		S1_65255621
		S3_1225826
		S2_4192760
		S3_54181137
		S2_63476776
		S1_10408831
		S7_54699104
		S8_61672040
		S1_72859890
		S1_60697364
		S7_38780069
		S1_19158075
		S6_2016238
		S7_10063274
		S4_12350791
		S3_72514855
		S8_55494919
		S3_3701003

Catogoery	Total	SNPs
		S6_46016091
		S9_56520935
		S3_55345680
		S6_25757585
		S2_10329067
		S1_60760849
		S2_3467457
		S1_72302172
		S6_53397096
		S1_77829870
		S1_74119554
		S8_45517554
		S2_1033058
		S2_73112202
		S2_10327318
		S1_77844844
		S9_1022399
		S1_63253460
		S5_8062626
		S2_4084454
		S7_59858470
		S3_20527194
		S1_57661166
		S2_5489030
		S3_71072286
		S7_433480
		S1_57873661
		S1_16550574
		S7_52290729
		S1_67713223
		S1_66821782
		S2_76459069
		S1_72938594
		S1_75371210
		S1_64803276
		S4_4912378
		S5_69780338
		S8_1964078
		S2_72627259
		S3_1612716
		S3_S3_69953690
		S3_54709278
		S3_54366761
		S1_7586033
		S2_3949815
		S3_51899482
		S3_70808967
		S3_70271997
		S4_27609152
		S1_55027975
		S2_43753638
		S6_25370209
		S5_9180586
		S8_60472299
		S1_62840640
		S6_18618253
		S9_11003881
		S7_64611983
		S1_57095619
		S5_68877568
		S1_67311181
		S9_215201
		S9_11048602
		S2_66010385
		S5_68949664

Catogoery	Total	SNPs
		S2_73743241
		S5_6868776
		S4_53841579
		S5_61921356
		S2_63220917
		S3_54181148
		S7_61598385
		S2_72257511
		S4_6982851
		S5_9501061
		S1_72249028
		S3_27332982
		S1_66894542
		S2_73566807
		S3_811888
		S1_77425569
		S1_56319080
		S5_61128718
		S7_2922548
		S6_53582910
		S8_59597206
		S1_15703776
		S3_67001530
		S3_73926857
		S6_47602809
		S6_4478300
		S1_11686495
		S2_67765557
		S2_4081146
		S5_1796145
		S4_34549626
		S5_18346264
		S5_61161417
		S6_15495927
		S8_60169744
		S1_16399200
		S6_47606086
		S7_9525011
		S5_69352315
		S4_40067750
		S3_14130075
		S1_68707540
		S4_6798591
		S9_54114044
		S3_55386147
		S3_69587303
		S8_61951464
		S7_64562615
		S2_66075052
		S4_67084076
		S3_58281905
		S7_62935595
		S6_1668341
		S1_48929901
		S3_73506864
		S5_68337776
		S1_78759222
		S5_9180587
		S6_50015665
		S9_51705478
		S4_6029220
		S1_63033810
		S9_696685
		S1_9980226
		S2_66067328

Catogoery	Total	SNPs
		S4_7538489
		S6_42478962
		S2_1124541
		S8_2063550
		S1_68317108
		S2_76493325
		S2_65371720
		S1_9068943
		S6_50818299
		S3_72757058
		S2_8823384
		S7_64104320
		S2_8114758
		S5_12278983
		S2_66139149
		S2_10822013
		S5_8179416
		S2_4554988
		S2_74073531
		S7_638468
		S3_34504594
		S4_55505690
		S7_52323628
		S6_46801672
		S1_67688619
		S8_3491614
		S4_66386087
		S8_57996517
		S3_64451731
		S3_64807784
		S1_69924407
		S9_50200470
		S1_71371621
		S2_381798
		S1_73581120
		S5_61217771
		S1_59792089
		S6_50704094
		S9_10737475
		S3_55801535
		S1_13350099
		S1_59360077
		S2_10992428
		S1_19645514
		S5_67495277
		S4_10444672
		S4_699886
		S8_61551510
		S1_11427964
		S4_52459577
		S6_41589913
		S2_5698087
		S7_1604728
		S4_52875870
		S1_60626360
		S1_71640607
		S7_518419
		S1_57095581
		S5_1625474
		S4_12312585
		S1_12141680
		S8_45237602
		S8_9637932
		S3_5617890
		S3_52432720

Catogoery	Total	SNPs
		S4_49581773
		S6_61215928
		S1_79967206
		S4_51951355
		S2_38766169
		S1_71839423
		S4_9016594
		S1_6777452
		S1_51003650
		S9_1209607
		S5_6101721
		S2_1518453
		S6_6459383
		S8_61955769
		S8_1273072
		S1_79424102
		S3_15424860
		S3_72634857
		S1_65259137
		S2_56923093
		S2_13608833
		S3_70242189
		S1_63714493
		S3_73319076
		S8_60851636
		S8_61831033
		S7_58298411
		S1_24189202
		S2_62404595
		S2_63675319
		S2_1529608
		S4_18387296
		S3_65880868
		S6_54237093
		S5_69847945
		S6_844919
		S2_68554612
		S6_53672975
		S6_44560701
		S7_5956975
		S5_61780045
		S8_58050879
		S5_63406233
		S6_61148455
		S1_7163414
		S1_55000298
		S2_60289804
		S2_10269500
		S4_4230825
		S4_21545784
		S3_53522681
		S4_41672886
		S4_2605772
		S2_56916118
		S1_76474483
		S1_51632507
		S8_3579230
		S4_6125199
		S6_56347575
		S5_39989649
		S5_11514079
		S5_18186632
		S3_51905751
		S9_58675219
		S7_63447975

Catogoery	Total	SNPs
		S8_2624230
		S4_67288927
		S2_31227632
		S2_3427375
		S8_57572575
		S6_18079701
		S1_57535863
		S2_8047288
		S4_9764500
		S2_7090830
		S1_77320609
		S3_6109980
		S6_2777752
		S6_6313589
		S9_5268906
		S9_2953842
		S5_3049767
		S2_77453399
		S2_61733853
		S4_61207866
		S2_67815306
		S6_47043894
		S3_54690289
		S3_15676468
		S1_61875784
		S7_60104728
		S4_50575672
		S3_14149882
		S2_26086455
		S2_67205941
		S6_45185334
		S1_9066149
		S1_22458253
		S1_58284768
		S5_69854905
		S6_28770344
		S2_56134011
		S5_35093851
		S2_3807179
		S2_8264189
		S5_65909593
		S8_48573325
		S4_9760328
		S9_1525044
		S1_74239859
		S8_60548718
		S9_52074477
		S2_5488312
		S1_61353199
		S1_68940149
		S2_16406565
		S9_54573728
		S2_61303348
		S2_66068675
		S4_24644227
		S2_8672338
		S1_24769366
		S9_54901526
		S2_59631771
		S3_5787681
		S1_79937172
		S6_5574713
		S4_51651301
		S1_18913481
		S2_65368155

Catogoery	Total	SNPs
		S1_72061574
		S8_1172545
		#N/A
		S3_61931999
		S4_61175063
		S6_8549958
		S4_13818619
		S1_69942785
		S8_60933919
		S1_73776791
		S7_1488727
		S8_3457008
		S3_62459763
		S2_58201045
		S2_64072238
		S1_66791777
		S9_54632794
		S3_2070608
		S5_61864593
		#N/A
		S1_59538745
		S1_60148695
		S7_17220489
		S6_46493884
		S3_71850326
		S1_11566641
		S1_77244925
		S9_44042849
		S8_59739821
		S2_57449697
		S5_11706240
		S8_37228096
		S1_12160232
		S7_54812619
		S2_58603907
		S4_11473135
		S7_60216274
		S1_68314299
		S4_50759253
		S4_9809279
		S1_70466569
		S4_44730215
		S2_7251638
		S6_53322407
		S2_69858490
		S2_62313039
		S1_22265957
		S1_20249962
		S7_54776852
		S1_7769638
		S5_10420074
		S7_62861600
		S2_65028854
		S6_37639750
		S4_40566681
		S8_33230819
		S2_12746321
		S2_58603902
		S2_11857642
		S1_62724352
		S6_334779
		S3_5683127
		S6_60774380
		S5_13190787
		S3_57777055

Catogoery	Total	SNPs
		S4_42294908
		S7_57691444
		S4_3998615
		S9_7290364
		S1_27144831
		S4_3227985
		S6_47306759
		S5_1444493
		S4_8253364
		S9_9648120
		S3_73817592
		S1_73462367
		S4_1111829
		S4_16471797
		S2_5649042
		S6_47984586
		S2_55351159
		S2_13869571
		S4_9770414
		S2_1031064
		S5_68814273
		S9_1675495
		S3_60919760
		S6_51486606
		S9_57575540
		S9_8722145
		S3_60583163
		S6_179752
		S6_1374724
		S2_73187616
		S7_62127570
		S2_66061447
		S7_42300098
		S2_3942161
		S9_54095056
		S5_63235242
		S5_400372
		S3_63116640
		S8_282506
		S9_49925961
		S3_57291958
		S6_45989914
		S1_17882144
		S4_50670455
		S3_53537478
		S7_7287651
		S6_46693987
		S5_15718254
		S2_16844037
		S2_4554991
		S1_2088839
		S1_70412989
		S3_5138622
		S3_66671222
		S1_27152261
		S2_12757616
		S6_53857084
		S2_30234071
		S5_10195408
		S1_58913442
		S1_65245565
		S6_6447842
		S2_3808824
		S9_6372646
		S1_61639729

Catogoery	Total	SNPs
		S4_26502893
		S3_58426089
		S2_66173264
		S7_8523552
		S2_57045031
		S5_1632250
		S6_52691502
		S6_18133171
		S2_58682738
		S5_61874437
		S1_6061250
		S4_43551741
		S1_27543185
		S5_2940193
		S6_48693485
		S7_58317823
		S4_2641039
		S3_74147892
		S1_59540878
		S8_56800357
		S3_72455090
		S3_52202504
		S1_62732270
		S1_16415247
		S6_1668323
		S4_18491108
		S1_2909457
		S3_70856980
		S1_6329239
		S5_62156695
		S6_47959857
		S1_2185240
		S1_59444106
		S2_10290306
		S1_80443338
		S2_11538180
		S3_61711105
		S8_55988803
		S2_16424450
		S9_49991092
		S1_45631298
		S2_60166321
		S5_15396398
		S8_49399727
		S6_38400852
		S9_937984
		S1_58855299
		S5_66356408
		S5_61217773
		S6_46372057
		S3_45569124
		S8_56141702
		S1_68317063
		S9_47028338
		S8_44365617
		S1_54719051
		S5_61338559
		S8_38398846
		S2_75369221
		S6_17437226
		S8_4310378
		S2_40478815
		S2_5814222
		S6_1668331
		S3_73363019

Catogoery	Total	SNPs
		S8_3217809
		S2_58290723
		S5_68989273
		S2_56191731
		S6_48693497
		S1_72558763
		S3_55005177
		S1_18040561
		S5_66648125
		S3_73160947
		S9_8256364
		S1_6512005
		S9_7342632
		S8_57578160
		S9_53303282
		S4_12354654
		#N/A
		S1_51003642
		S2_59616206
		S6_10649504
		S8_60201254
		S9_54870492
		S2_56869911
		S2_75155379
		S1_12267673
		S6_47901483
		S2_19155033
		S1_71711426
		S1_48286836
		S3_66685231
		S7_8427361
		S5_6772819
		S5_61112696
		S6_60453532
		S4_25292398
		S1_19187889
		S1_32611176
		S2_62729831
		S1_80826907
		S5_1160706
		S8_47148560
		S4_56306381
		S6_31579675
		S1_78922609
		S5_12290124
		S2_57585493
		S1_6362374
		S8_223170
		S4_2086898
		S2_1586050
		S2_69677492
		S2_6974937
		S2_74956031
		S9_54094900
		S6_47428407
		S1_16453891
		S8_37714468
		S1_10408843
		S6_54759098
		S1_50772956
		S4_67083987
		S8_50855534
		S2_8854394
		S2_73102905
		S4_5875165

Catogoery	Total	SNPs
		S6_41350789
		S4_51688437
		S3_62858453
		S9_57434563
		S8_59192389
		S2_2675715
		S6_47072413
		S4_10618423
		S1_77343710
		S3_54745112
		S1_29365925
		S1_1033410
		S3_22837801
		S2_67839039
		S4_5947055
		S1_29321274
		S1_75837680
		S1_61720517
		S1_56695773
		S2_71654312
		S2_17872803
		S1_14162142
		S1_28678204
		S9_52207281
		S4_16413681
		S4_7468085
		S6_48319033
		S1_54504949
		S5_62493036
		S8_4480782
		S2_60926834
		S9_59378883
		S2_61112485
		S6_582716
		S1_8644863
		S3_68755334
		S2_68946000
		S2_71569862
		S1_79438066
		S1_9116219
		S8_3429420
		S4_7334205
		S1_79201758
		S5_56030643
		S4_66384615
		S2_75347518
		S1_12305230
		S6_35831556
		S2_54243719
		S1_65923904
		S4_7539061
		S2_311588
		S1_7557040
		S1_27367102
		S1_8374220
		S2_4192750
		S3_20127125
		S3_54098258
		S1_64803277
		S7_7631211
		S3_72216861
		S2_58290730
		S6_27850393
		S4_37710153
		S3_5508147

Catogoery	Total	SNPs
		S1_5892123
		S1_10317819
		S8_3049256
		S8_4310165
		S9_54094873
		S8_3061622
		S6_1083434
		S2_64342471
		S3_70519676
		S9_57327912
		S4_13591799
		S2_12687026
		S3_2047928
		S7_8279791
		S4_45602602
		S3_56508833
		S2_3883280
		#N/A
		S3_62560224
		S8_1325537
		S6_47779156
		S4_4226844
		S3_57608920
		S4_2504903
		S6_52598613
		S1_48857704
		S4_53748218
		S1_71371614
		S2_18761325
		S8_49933732
		S2_67991002
		S8_59672737
		S5_9181142
		S8_33354675
		S6_54462516
		S7_60259939
		S9_50741975
		S4_27546389
		S2_67226080
		S8_44942788
		S3_69734236
		S9_3551801
		S3_73214830
		S3_63619178
		S1_65329488
		S8_3035049
		S2_13167151
		S5_50471736
		S2_67732479
		S2_59335727
		S4_50755716
		S3_71666499
		S9_51666248
		S1_11127550
		S1_5730741
		S7_58749724
		S3_56125153
		S7_6982472
		S9_54939922
		S4_53833056
		S6_38075645
		S2_55206102
		S2_6684685
		S7_53150006
		S1_7826453

Catogoery	Total	SNPs
		S1_2273073
		S9_49542327
		S1_8785558
		S6_58569147
		S1_14250878
		S4_26378376
		S3_69704218
		S6_44606988
		S8_4402092
		S7_1962165
		S6_13602651
		S8_54939830
		S9_50020478
		S1_66421404
		S6_51320776
		S9_5252452
		S9_58752343
		S4_3151402
		S8_60933440
		S4_66295700
		S1_2778776
		S4_22296506
		S1_53974667
		S8_2767489
		S8_49666349
		S2_65236004
		S2_59863358
		S8_60349238
		S4_58369895
		S8_2374797
		S8_60627561
		S9_57178153
		S4_20618405
		S3_15437691
		S6_47943768
		S6_41637974
		S3_58381849
		S3_19673542
		S7_62915731
		S7_64058338
		S9_8952572
		S1_9751902
		S4_50974937
		S4_11520800
		S7_57573962
		S3_74210043
		S2_3602200
		S3_41357827
		S8_9185750
		S8_16340632
		S3_66416002
		S2_14341061
		S7_60881347
		S6_5806282
		S4_66606720
		S2_28773371
		S9_6364899
		S6_1994917
		S6_26020408
		S3_56758868
		S5_3230559
		S7_53056341
		S3_16399910
		S2_57258315
		S1_77100624

Catogoery	Total	SNPs
		S6_50583825
		S3_1222908
		S2_9444315
		S2_3432028
		S6_33755271
		S2_60290265
		S5_65842231
		S1_28479321
		S4_10612003
		S5_61128740
		S2_58546698
		S1_16399087
		S9_42208745
		S5_1931829
		S1_9918963
		S3_68503204
		S2_11784981
		S7_53163001
		S2_57013246
		S2_476608
		S1_7639595
		S1_79932566
		S1_13015955
		S3_63436355
		S5_3230485
		S2_62729857
		S1_46554101
		S6_47323487
		S1_46564705
		S2_73341335
		S8_57423886
		S1_48780851
		S6_29670070
		S9_3156609
		S2_61085229
		S4_27223339
		S9_11627315
		S3_71247792
		S8_55282981
		S6_51912571
		S2_70915460
		S9_54916884
		S4_7276881
		S1_65725731
		S3_14131708
		S7_7590740
		S2_46699732
		S2_17508073
		S1_80030416
		S7_17425479
		S6_4091308
		S1_67220516
		S7_51288350
		S2_65466455
		S4_46060061
		S6_50307500
		S6_53942935
		S6_26232626
		S1_58377194
		S3_52428196
		S9_7314021
		S6_52885152
		S9_2931135
		S1_21035353
		S1_65259172

Catogoery	Total	SNPs
		S8_54552860
		S1_79932598
		S3_66638374
		S1_1616805
		S6_47072548
		S6_50656468
		S9_6791208
		S3_72789887
		S6_2016288
		S3_31894907
		S3_73335157
		S4_51278080
		S3_71926888
		S3_68981497
		S3_64927106
		S2_65965881
		S9_58955083
		S2_62235736
		S1_19184684
		S6_53168414
		S7_61501583
		S3_60599103
		S1_73787894
		S1_71946408
		S4_66135566
		S4_42464405
		S8_3018103
		S3_5434737
		S1_79484008
		S3_25732790
		S2_37442779
		S3_5659692
		S1_11702296
		S4_7013311
		S3_73085012
		S5_36118894
		S7_39933021
		S3_69281926
		S5_1966419
		S1_63011657
		S5_66997817
		S3_55024396
		S1_59557487
		S5_4915817
		S7_8523474
		S1_62493797
		S9_54111751
		S1_72373913
		S6_38160496
		S4_50759238
		S9_47050006
		S3_73494018
		#N/A
		S4_63000079
		S6_44752207
		S3_15435902
		S1_75048530
		S1_24765198
		S7_1522634
		S5_8752258
		S1_66652237
		S1_67220002
		S2_61053306
		S6_55723679
		S7_63635643

Catogoery	Total	SNPs
		S6_7009905
		S7_55982810
		S6_51619220
		S2_6950091
		S1_56853769
		S3_71236537
		S1_75403712
		S8_56606335
		S5_1795362
		S9_40978326
		S2_60585825
		S1_61976616
		S5_58549998
		S1_23907597
		S1_71603450
		S7_59043265
		S9_6799603
		S1_8627969
		S6_53914820
		S4_5530914
		S2_8854406
		S4_8158990
		S1_52755742
		S5_7986507
		S8_11020907
		S2_5801901
		S2_3998303
		S9_55067146
		S5_65524973
		S9_8945267
		S3_60614828
		S6_8888784
		S2_63676098
		S3_67027187
		S1_12758273
		S8_51659697
		S7_64079528
		S8_1238559
		S5_3230465
		S3_54927338
		S9_3063482
		S4_52778244
		S3_73160962
		S5_61407378
		S1_2467819
		S2_66606286
		S1_73887182
		S3_70902113
		S3_59634905
		S6_53341584
		S1_10998545
		S5_58550194
		S4_1114897
		S6_49221235
		S2_57591451
		S2_6396287
		S1_30100356
		S6_51464114
		S1_26918988
		S9_54316323
		S5_66075898
		S5_3044174
		S4_45750123
		S6_17003665
		S8_10349589

Catogoery	Total	SNPs
		S8_54927790
		S1_18074478
		S6_10574950
		S5_62499848
		S1_8138426
		S1_72603739
		S2_12744923
		S4_7562121
		S8_1172537
		S1_66564626
		S3_56682895
		S3_55301576
		S1_70466565
		S4_4321423
		S3_59636495
		S2_3415399
		S4_2303483
		S5_11706208
		S3_71247750
		S3_54934264
		S3_55003488
		S5_63392519
		S2_67205826
		S5_50560488
		S8_2121734
		S4_48520527
		S7_S7_585874
		S1_14458911
		S3_51506174
		S2_75358317
		S4_33326536
		S1_24472011
		S9_5167777
		S1_21886579
		S7_14910962
		S5_9053137
		S6_3980114
		S8_54749069
		S6_44752165
		S2_10587243
		S6_45174768
		S2_16390672
		S1_22260274
		S2_60295759
		S8_49628006
		S3_15686372
		S5_5335301
		S1_79291509
		S4_4321219
		S9_53301250
		S1_56695764
		S3_73846856
		S7_60412965
		S9_14174725
		S5_65636901
		S7_5970059
		S2_10826202
		S8_1234891
		S6_45989915
		S9_5153776
		S4_8528735
		S2_50018484
		S2_S2_15196384
		S5_63815784
		S2_75954389

Catogoery	Total	SNPs
		S3_5130725
		S7_5171928
		S3_53265799
		S1_5857742
		S7_17079939
		S3_62407523
		S1_74349810
		S7_60661589
		S6_51193377
		S5_16133509
		S8_1234810
		S9_56108757
		S8_15302543
		S6_332674
		S1_50221834
		S8_4670135
		S4_67241694
		S5_59444812
		S2_242165
		S5_62499848
		S3_65869614
		S4_2242674
		S7_59290017
		S7_5172204
		S4_33698477
		S2_26494607
		S5_2693247
		S3_57954364
		S9_52343089
		S7_63707853
		S7_64058328
		S2_65884861
		S6_7198292
		S4_13959653
		S6_6458386
		S2_10329076
		S2_73407534
		S3_5165375
		S2_3880374
		S1_30745497
		S9_50971984
		S4_1609526
		S6_60845204
		S4_16299163
		S8_570864
		S7_12005749
		S2_60118380
		S8_10136253
		S7_64079534
		S9_1663519
		S5_67765855
		S2_61974862
		S5_11792659
		S7_56844702
		S2_64350264
		S3_3722831
		S1_8228279
		S1_73037093
		S1_2600429
		S5_3340685
		S9_6236089
		S7_28802083
		S2_68561628
		S3_67966293
		S2_17982369

Catogoery	Total	SNPs
		S9_53929927
		S9_57139854
		S4_66158417
		S9_1187892
		S4_51427655
		S1_60005632
		S1_65333498
		S2_75061532
		S9_50520650
		S1_76914114
		S9_2661072
		S1_62724117
		S2_12509292
		S5_67059095
		S7_62070716
		S6_39393839
		S1_14080857
		S3_70480280
		S1_59824457
		S1_28678212
		S3_59662486
		S4_68098060
		S9_53085399
		S2_66089962
		S5_50603261
		S5_66405146
		S7_64050436
		S1_71632909
		S9_53527945
		S7_60884047
		S3_56295922
		S2_61999176
		S1_74291692
		S2_69174811
		S4_52380439
		S1_56295456
		S5_1966411
		S2_65883301
		S1_17752459
		S1_S1_14173301
		S1_66828431
		S6_47955800
		S1_13455799
		S7_1771733
		S1_76251095
		S3_16373393
		S2_60965630
		S1_59504138
		S3_71678255
		S1_1862840
		S8_61356263
		S3_5640566
		S7_61527372
		S6_51446436
		S6_49220919
		S2_59822161
		S3_14145258
		S3_20276874
		S8_53982170
		S1_15714576
		S4_66311102
		S1_80320574
		S1_20207188
		S3_69630919
		S5_67987896

Catogoery	Total	SNPs
		S1_20349823
		S3_51704387
		S5_63240122
		S6_50396681
		S6_47343576
		S2_10821448
		S1_2101066
		S2_67640679
		S6_49768869
		S6_46735578
		S2_67197992
		S8_51868331
		S9_54016285
		S4_61176012
		S8_6158495
		S8_51715231
		S9_44413801
		S5_63277814
		S2_65854400
		S5_11792649
		S6_44563330
		S1_77104893
		S3_70587471
		S2_75672237
		S2_65853422
		S7_60453848
		S3_46370577
		S6_2079215
		S3_69365438
		S4_1340145
		S2_59247272
		S3_70839264
		S6_1047746
		S1_53479604
		S3_62407709
		S1_77215568
		S4_836916
		S8_43055656
		S2_3388909
		S2_5733427
		S5_61080917
		S8_3231854
		S2_2241170
		S2_30234075
		S2_67991002
		S2_70915459
		S1_3624220
		S4_8218452
		S5_61932087
		S3_67762398
		S2_68005889
		S1_15897245
		S6_57413579
		S2_26292904
		S7_16835104
		S3_620437
		S3_5061152
		S8_3164711
		S4_40990773
		S3_70074373
		S2_61543617
		S9_2967322
		S8_1705171
		S5_63471132
		S1_62720834

Catogoery	Total	SNPs
		S3_73319062
		S1_71946556
		S4_53362412
		S6_53622975
		S9_50867919
		S1_62223020
		S2_10821564
		S2_5417752
		S2_75343559
		S8_61921714
		S4_7210462
		S3_59368492
		S1_10100856
		S9_58172353
		S8_61547295
		S8_452902
		S3_71689730
		S2_41949456
		S1_940233
		S2_48444914
		S4_40249823
		S5_6827018
		S6_2680976
		S9_4526982
		S5_7243138
		S8_56933258
		S2_6887602
		S7_61525358
		S2_56881328
		S5_8734126
		S2_64756277
		S8_19296595
		S2_63058877
		S1_69941662
		S1_61679998
		S6_49473952
		S4_15715321
		S2_77505472
		S5_66068685
		S6_28741708
		S5_61864584
		S5_67686263
		S1_63458067
		S1_22035208
		S3_57894810
		S2_65295494
		S3_68919631
		S3_56719282
		S5_57690130
		S4_27918718
		S1_6040046
		S6_41979847
		S4_67302241
		S5_11062821
		S8_49086953
		S1_74530857
		S2_63307905
		S3_70623205
		S1_70246976
		S3_55448916
		S8_56542076
		S2_2488280
		S4_34662277
		S8_51822714
		S6_38461519

Catogoery	Total	SNPs
		S2_11559918
		S1_4086630
		S8_3183161
		S1_18035353
		S8_61538954
		S9_55480032
		S3_5618886
		S1_55817591
		S5_67495269
		S3_56254558
		S6_47878999
		S9_58735793
		S5_10814631
		S7_6761398
		S2_4739832
		S3_3063086
		S3_60922561
		S2_75749557
		S3_73992110
		S3_73170741
		S3_71625629
		S8_1753891
		S1_6458070
		S6_51803534
		S5_5255223
		S3_71751768
		S4_5085177
		S1_71711435
		S4_48956949
		S4_6775390
		S7_3453248
		S4_21779044
		S2_8890439
		S1_72621969
		S2_66339829
		S5_1327108
		S9_8192355
		S9_51669599
		S1_15315608
		S4_4160425
		S2_68922342
		S8_2271668
		S5_6769982
		S6_16399579
		S9_52612562
		S1_67105468
		S8_49204354
		S3_52457550
		S2_60550163
		S6_47765275
		S2_9631256
		S3_73934570
		S1_55049708
		S9_50520621
		S9_59253270
		S6_13664837
		S9_48996474
		S1_73215664
		S7_52290642
		S3_70879261
		S2_60572608
		S2_61392140
		S7_42895126
		S3_61426781
		S6_51192698

Catogoery	Total	SNPs
		S2_59822171
		S2_12670858
		S7_12009561
		S3_14322126
		S2_56955036
		S1_55830178
		S7_64826329
		S4_3800775
		S1_71624871
		S8_56595065
		S4_13818532
		S1_1828197
		S1_23965059
		S8_53793095
		S6_3876226
		S2_57862882
		S6_50655163
		S2_66339824
		S8_1567174
		S2_73720002
		S8_47524031
		S7_10503099
		S3_428037
		S9_57905483
		S1_74815231
		S2_61060068
		S2_1911917
		S2_16386586
		S6_41984883
		S2_68821234
		S9_54552058
		S8_57440356
		S3_57350225
		S3_4759158
		S2_11505508
		S2_66173496
		S2_65307906
		S1_78032412
		S3_72444136
		S5_12536532
		S7_62396522
		S9_44413784
		S1_21735704
		S5_61598066
		S7_64649016
		S4_34694178
		S2_60118486
		S6_35897293
		S4_62878156
		S6_37970635
		S9_42251177
		S3_6089214
		S8_58050877
		S1_65769388
		S2_5389773
		S7_833959
		S6_54583335
		S3_68056180
		S4_51529409
		S1_13157810
		S6_45274072
		S8_53982123
		S2_75343608
		S1_59114756
		S5_61074242

Catogoery	Total	SNPs
		S2_1031138
		S2_12670176
		S7_52314496
		S4_39846767
		S1_55049823
		S3_48834363
		S4_26416603
		S2_38238686
		S3_55005196
		S3_2117223
		S4_66233649
		S2_61405281
		S5_9535528
		S7_55377787
		S2_49470484
		S1_60384389
		S8_53823127
		S8_49656293
		S6_60593908
		S1_48857662
		S7_56731708
		S3_74147896
		S2_68945897
		S1_9153558
		S8_59673221
		S2_60289479
		S1_71592624
		S9_57770839
		S1_21201484
		S4_66042972
		S1_52916600
		S6_47428500
		S2_64790488
		S9_8129247
		S6_1168781
		S7_6679582
		S3_19997901
		S2_61785882
		S2_7715740
		S3_57895062
		S2_68008179
		S6_56495756
		S1_7436908
		S7_501072
		S6_50647160
		S1_21735702
		S4_54131969
		S6_54617161
		S8_5255587
		S9_52364710
		S5_61579879
		S1_59566987
		S8_61165327
		S4_11499011
		S9_6375882
		S7_58534237
		S2_71484593
		S7_1495817
		S9_55626957
		S6_7162057
		S8_61925285
		S8_55596063
		S3_74068782
		S2_65643680
		S1_77869564

Catogoery	Total	SNPs
		S1_68689757
		S3_71850496
		S2_64461211
		S5_62173125
		S3_61246826
		S1_6777462
		S6_58540086
		S4_10207096
		S3_4757321
		S5_61873263
		S2_56915994
		S6_41925250
		S5_5114467
		S1_65287624
		S2_14259246
		S8_4413966
		S4_4063552
		S4_7890013
		S3_54541096
		S3_47935457
		S3_73610621
		S2_75411437
		S9_56553166
		S6_44606982
		S2_2753826
		S2_55354489
		S5_15273955
		S7_8635565
		S1_80706584
		S2_40141931
		S5_61572373
		S5_63691584
		S9_7314497
		S8_59750934
		S7_60704260
		S7_2117996
		S1_6518631
		S6_47072606
		S8_33521982
		S8_55247545
		S1_24741103
		S1_2909351
		S6_50435992
		S7_63635692
		S9_58935197
		S3_72268847
		S3_73887670
		S6_54267821
		S2_59247263
		S4_54131966
		S6_54726096
		S1_12757854
		S4_37411241
		S1_7621128
		S8_57985272
		S3_54213258
		S1_6537478
		S7_60181892
		S4_27570565
		S6_47308928
		S8_2016214
		S2_7736129
		S4_1193630
		S2_2004855
		S6_2712172

Catogoery	Total	SNPs
		S7_64024330
		S1_53480547
		S6_53238565
		S2_63826029
		S5_6528733
		S7_1380351
		S2_61923806
		S2_10821999
		S3_4756380
		S9_54603795
		S7_63675831
		S6_54759081
		S2_58212223
		S6_S6_3050150
		S2_10876306
		S6_26699321
		S2_74773882
		S9_11647601
		S9_58295272
		S4_51774094
		S3_57040385
		S8_2574580
		S5_12778535
		S7_52107054
		S4_2635001
		S5_10619197
		S2_65121309
		S2_75416237
		S2_4566127
		S1_71592628
		S1_26853748
		S4_772576
		S4_66043130
		S5_1649786
		S5_3265487
		S1_66791735
		S6_58739488
		S1_64993142
		S9_51177785
		S9_55573838
		S1_17584213
		S7_40498280
		S1_74228838
		S4_52472706
		S2_6755773
		S3_54260932
		S2_65345311
		S1_79267223
		S1_67933631
		S2_61685837
		S3_34504572
		S4_6084213
		S5_67059024
		S1_7781706
		S1_63951024
		S3_31783930
		S1_60150466
		S1_60959956
		S9_5232658
		S4_1260597
		S8_15919415
		S5_11615613
		S5_1041083
		S3_59406540
		S3_53562015

Catogoery	Total	SNPs
		S8_47521894
		S4_52963601
		S9_4454945
		S1_60414926
		S8_56500960
		S7_10048179
		S8_18048797
		S9_44572782
		S5_11447812
		S5_8085211
		S7_57052933
		S1_72303358
		S3_3757319
		S2_18810776
		S1_19988833
		S1_9751858
		S2_10527303
		S1_70772806
		S1_75035044
		S2_47145728
		S1_79932477
		S3_56719304
		S7_62611669
		S9_56571019
		S2_61654541
		S8_49573366
		S6_49758882
		S8_59643430
		S3_48916504
		S2_59247281
		S1_7008462
		S8_32316963
		S3_6242219
		S2_12102302
		S2_61856962
		S2_11055154
		S1_54540464
		S1_9814306
		S7_38972062
		S3_69421227
		S4_52342981
		S4_54168284
		S2_75048453
		S2_4085315
		S1_79203159
		S1_67623758
		S2_68759227
		S5_1102289
		S8_51333350
		S6_16547955
		S6_38341820
		S3_61426735
		S2_6015428
		S1_77430722
		S3_60719539
		S2_6139977
		S5_2042715
		S1_78755201
		S1_71174951
		S1_51542121
		S1_57003087
		S6_47800437
		S1_16415205
		S1_52089020
		S3_57771171

Catogoery	Total	SNPs
		S9_57773302
		S1_58193601
		S6_52639173
		S9_6372649
		S9_42514897
		S3_61904903
		S4_49581036
		S2_426294
		S8_19298116
		S3_70711179
		S4_51877969
		S1_17685407
		S5_67938258
		S3_70932047
		S1_11122066
		S2_59822500
		S1_9068998
		S1_14250878
		S3_72516599
		S6_50819934
		S6_54403110
		S1_50569296
		S2_25535062
		S2_6423691
		S1_8879768
		S4_11112203
		S3_65869628
		S2_71654320
		S9_4417556
		S4_23936262
		S7_6671220
		S7_62476177
		S2_12811762
		S6_51593732
		S7_6373392
		S9_35287601
		S4_2233503
		S6_37905932
		S1_77767786
		S1_73553532
		S1_17585361
		S8_3048650
		S3_3903998
		S2_69046096
		S3_47944827
		S5_58857005
		S6_35659689
		S3_57320576
		S3_52457567
		S8_58069327
		S2_62118216
		S9_57441013
		S3_46904270
		S6_3440975
		S3_2193794
		S4_58494908
		S1_71525802
		S2_57774731
		S2_76812920
		S7_15656493
		S2_69714835
		S5_4765654
		S5_2601102
		S1_19640563
		S2_3759397

Catogoery	Total	SNPs
		S1_12922234
		S2_75150871
		S4_7889919
		S4_1198822
		S1_66550225
		S3_70855646
		S1_78711838
		S8_50941728
		S1_60913882
		S6_58535252
		S4_1359213
		S2_59370706
		S2_3880149
		S4_57520764
		S4_470026
		S2_49630758
		S6_51464003
		S2_68916243
		S1_57837192
		S3_71247751
		S1_16395838
		S3_53308205
		S4_15605713
		S8_56576907
		S3_74131887
		S3_54922729
		S5_62642589
		S3_69709471
		S1_6574699
		S6_44731774
		S2_65685599
		S2_75975461
		S3_72635019
		S9_50369927
		S4_53381283
		S3_65906491
		S6_48019758
		S7_277809
		S3_56869303
		S1_10689214
		S1_11466427
		S3_58173834
		S6_46383808
		S5_665447
		S7_4778153
		S6_6448055
		S8_3415849
		S4_5467623
		S7_64554803
		S1_9932963
		S1_27157056
		S3_72514852
		S6_46545288
		S2_64350266
		S3_73216548
		S6_14141037
		S6_18140773
		S2_17211039
		S8_1699390
		S9_7790161
		S4_50669860
		S3_73356826
		S9_3351148
		S8_3185347
		S3_69847069

Catogoery	Total	SNPs
		S3_67841011
		S3_48915713
		S1_57391510
		S9_51091428
		S6_60905055
		S2_14724290
		S6_50310472
		S1_4119014
		S5_6101726
		S3_61485938
		S1_8917452
		S1_67918787
		S2_12927569
		S3_3904056
		S2_8264081
		S9_42812142
		S5_67722069
		S2_17211248
		S3_57368268
		S1_22272053
		S8_54927868
		S2_59125996
		S2_12687911
		S6_41417131
		S7_64061503
		S4_20093502
		S5_6176831
		S7_64751023
		S9_8945262
		S1_6512001
		S3_60533630
		S3_63115560
		S6_31579668
		S9_55626870
		S6_40780270
		S6_58135131
		S4_7338336
		S1_66986051
		S3_70398398
		S3_72311074
		S6_50646272
		S7_9590904
		S1_79057697
		S3_69598984
		S3_1942549
		S2_1462273
		S1_59699609
		S3_73925048
		S1_6331848
		S1_56910567
		S3_67503603
		S3_2073442
		S3_60800076
		S2_13167466
		S5_67934068
		S1_14150886
		S1_64086234
		S6_57207705
		S5_18513731
		S9_10240625
		S8_4682976
		S5_63406180
		S4_2762235
		S7_16752633
		S4_51877982

Catogoery	Total	SNPs
		S7_8471398
		S4_58205622
		S2_13868785
		S2_3760482
		S9_2931136
		S1_5800574
		S8_9147017
		S2_59247351
		S9_3184767
		S2_61333291
		S7_6432640
		S3_1871231
		S8_59243374
		S8_55622517
		S3_70253249
		S3_52824313
		S2_59755650
		S5_11514085
		S5_65640185
		S2_4809216
		S8_61954381
		S2_4109239
		S1_68940228
		S4_12727246
		S4_10304304
		S7_53150024
		S2_61561841
		S2_58201045
		S2_76616256
		S1_14860781
		S3_66774539
		S6_31579654
		S2_31207325
		S9_6434698
		S8_412290
		S7_59404118
		S4_51951345
		S3_2481071
		S2_3428389
		S1_11205597
		S1_21722507
		S2_73102860
		S4_33270710
		S2_10869925
		S8_45660560
		S1_66898494
		S7_39882178
		S8_61138115
		S9_9992418
		S7_2771934
		S3_70018725
		S3_68982640
		S4_61180155
		S3_73860602
		S1_57110286
		S5_10814493
		S6_6889651
		S2_61739709
		S2_75509978
		S3_46205847
		S1_66775244
		S4_1064477
		S5_69861647
		S1_13095889
		S5_11535077

Catogoery	Total	SNPs
		S3_65425737
		S5_54661180
		S2_9481036
		S9_58179027
		S8_46715023
		S3_5659675
		S5_2696740
		S5_383208
		S4_52343001
		S1_55504269
		S2_75613717
		S8_60246871
		S2_65383867
		S6_52697411
		S6_1668315
		S5_5791771
		S6_53396940
		S9_3702137
		S2_18573339
		S2_17760736
		S2_57705151
		S4_68098078
		S5_9535474
		S8_2653805
		S2_75369258
		S6_53600146
		S2_7736123
		S6_35332078
		S8_5260140
		S1_63346728
		S8_2237878
		S1_72249029
		S2_68095517
		S3_69847058
		S3_5507158
		S2_71417778
		S2_59305271
		S5_9053140
		S3_69394397
		S3_59406432
		S6_51318790
		S7_62450399
		S1_20194842
		S4_1645522
		S1_66813879
		S6_15439544
		S5_66622968
		S3_67154236
		S2_56870786
		S6_54627220
		S2_770745
		S3_57350309
		S2_3403375
		S1_78642346
		S1_12945087
		S1_72276683
		S5_69852350
		S3_14047214
		S2_72643076
		S8_45237846
		S1_71631728
		S1_15315621
		S6_47103799
		S9_8722181
		S2_10493857

Catogoery	Total	SNPs
		S4_12387241
		S2_4739909
		S8_61980276
		S1_S1_72932590
		S3_57215991
		S4_5534685
		S7_62904881
		S2_2901641
		S1_68781631
		S5_6868737
		S3_5643246
		S8_9836342
		S1_2754230
		S1_64420548
		S4_43994456
		S4_56236474
		S1_60778310
		S3_73765875
		S4_20672543
		S7_60654511
		S4_7781983
		S5_66614463
		S8_5263071
		S4_10277844
		S2_6396276
		S1_68781634
		S7_6373329
		S4_7539581
		S6_49447561
		S7_62494208
		S5_65814700
		S2_6395263
		S1_57951237
		S2_2515914
		S5_68988367
		S2_10329062
		S4_26417866
		S6_48555099
		S4_6937201
		S5_8727639
		S4_50917209
		S6_60926387
		S2_63927745
		S9_4217666
		S2_69710857
		S5_65468114
		S2_48387677
		S8_1921018
		S8_60932755
		S8_4413943
		S5_4557983
		S7_6670806
		S4_37522789
		S3_6328976
		S2_7685710
		S3_67027237
		S2_58290728
		S3_4565778
		S6_52928506
		S4_62880587
		S6_49437039
		S3_71315054
		S6_51320623
		S2_76431886
		S7_52306549

Catogoery	Total	SNPs
		S3_63007109
		S1_7164246
		S1_8122152
		S5_68867140
		S9_59378886
		S9_52932645
		S1_7822997
		S5_61188859
		S3_4576514
		S1_74360153
		S1_1344728
		S9_8407302
		S8_58455788
		S3_56098157
		S2_3807178
		S6_60799708
		S4_6899228
		S3_4583922
		S3_65718958
		S5_15116555
		S7_64475706
		S2_68053149
		S4_10610510
		S2_8707779
		S2_6996532
		S4_39854481
		S6_14683572
		S2_67455094
		S4_836917
		S4_19153289
		S2_7090969
		S7_52290732
		S8_60393358
		S2_59619957
		S7_57380759
		S4_11520897
		S2_9725906
		S6_58812609
		S5_12537139
		S6_1668318
		S3_59576841
		S1_66889169
		S2_8410253
		S3_14045862
		S3_74068891
		S2_54406572
		S8_59681050
		S3_19995039
		S8_61559333
		S3_56160357
		S1_79546077
		S1_20564749
		S6_50581941
		S9_9802214
		S7_63365633
		S7_61115672
		S2_73675544
		S7_6421784
		S2_71486246
		S1_64159579
		S1_79751945
		S8_53982128
		S9_11656014
		S1_64391968
		S1_55000331

Catogoery	Total	SNPs
		S2_71569846
		S4_51651387
		S2_73837940
		S1_57663604
		S2_56440839
		S1_18273223
		S2_70921955
		S4_10430516
		S2_4147081
		S8_51415236
		S3_51322650
		S8_32316970
		S1_4298509
		S8_45237635
		S6_58575642
		S7_54683265
		S3_72321755
		S1_11205156
		S1_19426120
		S2_56174093
		S1_72949976
		S2_9371768
		S5_65524489
		S9_56521857
		S9_1260336
		S6_45716352
		S1_75656628
		S8_61924362
		S3_62202798
		S1_8895498
		S2_53203178
		S8_56354233
		S3_65928542
		S3_55948602
		S5_8830726
		S3_6636551
		S8_5164998
		S7_61595357
		S5_10619304
		S6_53169233
		S2_74073525
		S1_20691936
		S9_51253879
		S3_3902056
		S1_75862501
		S4_61175058
		S2_68588401
		S6_40512586
		S4_3813212
		S3_45640159
		S1_15343971
		S3_19995106
		S2_1475429
		S6_2665895
		S3_53292786
		S5_1988139
		S4_1778425
		S1_66786128
		S1_31206015
		S6_26313471
		S7_7903951
		S7_5526821
		S3_46621182
		S6_46497374
		S9_48996396

Catogoery	Total	SNPs
		S1_75888895
		S7_62475183
		S2_74073532
		S1_6061339
		S1_479175
		S2_13166015
		S8_35632492
		S9_58742698
		S2_1007453
		S4_10430558
		S1_64484797
		S8_40856989
		S2_10899168
		S4_5107560
		S6_53253648
		S3_4274823
		S1_73661577
		S2_8451144
		S9_3917665
		S3_72293227
		S3_63673516
		S2_2005456
		S4_47808752
		S8_53262160
		S6_50696791
		S5_63651450
		S7_63639768
		S6_45572134
		S2_14677806
		S2_1969912
		S1_17276942
		S2_62925029
		S3_68957333
		S9_9099737
		S1_67311337
		S2_9492258
		S4_51648062
		S9_52306750
		S7_1674585
		S1_18040565
		S1_6512006
		S2_42595127
		S1_80734084
		S2_11550187
		S9_43817563
		S1_1862813
		S7_64515472
		S1_9893907
		S8_1664246
		S1_66351768
		S2_6396291
		S1_14090643
		S1_79156362
		S1_66898496
		S2_62128530
		S1_18202899
		S2_69053428
		S7_59354281
		S7_58846074
		S1_57681103
		S3_72851941
		S6_48553823
		S8_3665013
		S4_13177434
		S2_60105017

Catogoery	Total	SNPs
		S7_55976783
		S6_14489381
		S2_61642124
		S4_66983875
		S4_43691845
		S8_3230577
		S3_73319087
		S1_13425997
		S3_5561235
		S1_49271376
		S4_4541011
		S4_51278088
		S5_61210521
		S3_2480180
		S1_17702077
		S6_799609
		S5_65852068
		S1_68195770
		S8_49399779
		S6_60926393
		S3_2379500
		S8_38457783
		S8_38457825
		S6_54303424
		S3_70627433
		S3_13434179
		S9_4087533
		S9_52104458
		S9_57068845
		S2_73675542
		S1_72488980
		S3_67001570
		S2_6890939
		S9_41543945
		S4_56610976
		S3_70567772
		S2_69858495
		S5_10354351
		S9_5861395
		S5_65814694
		S6_58759791
		S1_78818476
		S6_17792747
		S5_56047102
		S2_75961503
		S1_60954587
		S8_48960972
		S8_4124307
		S2_61648917
		S6_1379474
		S6_58535254
		S7_2761989
		S3_3288715
		S7_63202506
		S1_77338732
		S9_5152245
		S2_58202074
		S1_987698
		S1_58937862
		S2_60289898
		S7_887791
		S2_28979172
		S1_30422311
		S5_2693011
		S8_3018099

Catogoery	Total	SNPs
		S3_69941298
		S5_51751535
		S1_77343717
		S2_734465
		S7_63747747
		S3_70855665
		S8_56707210
		S7_57111429
		S4_67979482
		S5_10619240
		S3_62065710
		S6_41262842
		S4_2236269
		S2_67621155
		S5_12537116
		S2_63846406
		S1_3156781
		S2_59912796
		S2_3251749
		S4_4868944
		S3_69744371
		S1_77431918
		S8_55144760
		S4_50063914
		S5_67567202
		S8_4445198
		S2_61207197
		S3_73704977
		S1_72938298
		S1_45697359
		S8_44227095
		S7_60580527
		S5_2916961
		S8_56411892
		S3_58169191
		S3_3288724
		S1_7960215
		S6_53273385
		S4_7868595
		S4_7277247
		S6_53906360
		S6_51255327
		S2_71595650
		S1_71946413
		S7_17425478
		S2_56870789
		S9_2825291
		S7_15970199
		S9_50370278
		S2_6027145
		S9_2906067
		S5_4898353
		S4_9770409
		S6_34406590
		S9_5342115
		S4_54102695
		S6_53080032
		S7_64025495
		S5_1481099
		S1_11550774
		S4_66231003
		S5_3175123
		S1_11198833
		S1_12587926
		S5_1456058

Catogoery	Total	SNPs
		S1_78509765
		S7_537129
		S3_3902078
		S5_10881181
		S3_68382271
		S1_5892120
		S6_44072840
		S2_75171615
		S3_72504747
		S1_26887850
		S1_65195985
		S6_58881504
		S8_61138133
		S7_60709311
		S1_66031407
		S8_32877095
		S2_64471798
		S1_13325758
		S2_59238551
		S2_4555218
		S2_61712089
		S8_1240725
		S2_40078289
		S7_63571791
		S4_23827327
		S2_76889756
		S3_58766058
		S1_57115004
		S4_67091986
		S1_7785112
		S5_62378797
		S3_70135397
		S7_60709192
		S4_66103988
		S1_59275320
		S3_73160959
		S7_52314599
		S1_78383756
		S3_5659687
		S2_63583705
		S3_62471596
		S6_47835218
		S4_22815610
		S1_64803271
		S8_7197883
		S5_61792605
		S3_69810210
		S9_8785348
		S2_52938118
		S9_51705474
		S2_54479250
		S6_6448058
		S4_39141180
		S1_59140879
		S1_60883941
		S3_73926885
		S8_51715222
		S2_12004889
		S1_72898842
		S6_47959762
		S9_58738390
		S4_1054265
		S5_10795310
		S8_48633710
		S2_60347236

Catogoery	Total	SNPs
		S2_2235732
		S8_57441920
		S9_58666230
		S4_52873201
		S8_50831064
		S9_3636133
		S1_8138427
		S7_10074836
		S9_58198140
		S4_1570085
		S3_69437897
		S1_4122224
		S1_78200364
		S2_75347489
		S6_53335448
		S1_10350959
		S8_48663945
		S1_13401158
		S6_54303427
		S1_4086704
		S1_59362348
		S4_35017563
		S3_13769795
		S1_25897346
		S6_49765295
		S3_51999871
		S8_60201201
		S4_21752427
		S5_66098808
		S4_950262
		S1_72925409
		S7_6633746
		S1_64629077
		S3_56719296
		S2_465794
		S1_12247817
		S4_2626970
		S2_62393752
		S4_7901377
		S2_55206127
		S1_59398393
		S1_28880161
		S3_5109480
		S9_44414325
		S6_29537505
		S8_51525744
		S1_14438987
		S1_60776170
		S2_471371
		S8_5015501
		S6_53573147
		S1_71822737
		S9_7286524
		S9_1663414
		S7_54092673
		S1_16552914
		S8_54628426
		S1_3175287
		S5_67494904
		S3_69744197
		S2_57519037
		S6_6401507
		S2_71634074
		S1_8973948
		S9_59155260

Catogoery	Total	SNPs
		S1_72350725
		S1_71708120
		S6_56333662
		S9_57840147
		S1_16547930
		S4_61260561
		S1_13188626
		S5_10091900
		S2_67205925
		S6_41984724
		S3_48302014
		S4_26734465
		S2_3715633
		S2_8047326
		S1_65484339
		S8_44849411
		S1_64159615
		S1_72373892
		S1_60414925
		S4_14926829
		S6_53396989
		S4_7169870
		S1_8644854
		S1_7557048
		S3_46907954
		S1_59977868
		S1_6521536
		S8_4414063
		S2_2675710
		S4_62789639
		S1_77764841
		S1_13426299
		S3_61933663
		S8_44365622
		S8_5164991
		S4_54492031
		S9_6371724
		S9_3357211
		S4_39854480
		S3_621000
		S1_71640628
		S4_38716991
		S8_57545948
		S4_55526477
		S7_62396535
		S7_313559
		S1_52991993
		S1_64156472
		S8_4281169
		S2_69560271
		S9_49551667
		S1_22791663
		S2_32224095
		S7_62832449
		S9_54909060
		S6_51334130
		S1_68321563
		S1_79165402
		S6_15530444
		S3_15662284
		S9_44566100
		S7_58283229
		S2_11097375
		S4_7889913
		S8_56847149

Catogoery	Total	SNPs
		S3_50748646
		S8_58034973
		S9_6434684
		S2_69020529
		S9_3162976
		S8_43354298
		S2_10290303
		S3_13318225
		S6_58812624
		S8_3173662
		S9_55626872
		S1_74490523
		S5_62355355
		S9_57641434
		S5_61930579
		S8_2724542
		S7_40498318
		S7_65081303
		S4_464195
		S3_61435793
		S3_3611574
		S4_52831420
		S7_57555560
		S7_64118068
		S6_54339812
		S8_57441914
		S2_7315383
		S2_11851256
		S6_6691600
		S4_53778208
		S3_58281906
		S1_7436819
		S3_19680865
		S4_54119103
		S8_49668122
		S3_73331300
		S6_3559484
		S3_73289913
		S2_4257875
		S2_7615324
		S1_59457264
		S8_4491948
		S1_62825680
		S2_3899853
		S2_64334139
		S1_2392323
		S1_73687923
		S4_5855189
		S8_3193975
		S6_48594444
		S7_60741571
		S8_59178445
		S1_80660369
		S2_62315849
		S2_8430561
		S2_64342420
		S1_18075356
		S7_41131271
		S6_47179478
		S2_62051205
		S6_51419987
		S6_6466675
		S2_41691968
		S5_61779846
		S7_55975788

Catogoery	Total	SNPs
		S1_72898836
		S9_52658542
		S2_65772070
		S1_59393980
		S3_4612580
		S7_9519617
		S8_50345484
		S4_67774402
		S1_2053226
		S3_73629105
		S4_33300734
		S2_61654245
		S2_6045011
		S8_4934939
		S1_8503092
		S2_61926722
		S5_69942042
		S8_60451711
		S1_57110298
		S5_24452322
		S4_43409081
		S2_6611936
		S4_67020059
		S5_8160946
		S8_56248491
		S3_66001122
		S1_66813861
		S4_67115738
		S6_41220193
		S5_69989073
		S1_59338094
		S1_5797104
		S5_66735452
		S3_16373419
		S2_74965511
		S1_7998057
		S1_59338088
		S4_52342998
		S5_65658215
		S9_5036351
		S2_4135291
		S3_73184545
		S5_67367063
		S6_41984827
		S1_56731835
		S5_4526938
		S3_60614840
		S6_53881946
		S1_74939956
		S3_61719259
		S2_5791959
		S2_65075302
		S2_16844396
		S1_64391925
		S2_73376028
		S3_62094729
		S6_1316170
		S3_72184267
		S6_50364693
		S1_68321503
		S5_63114372
		S9_8213283
		S7_47640666
		S9_57152579
		S9_6659349

Catogoery	Total	SNPs
		S5_11779485
		S2_3294511
		S9_141045
		S6_58535636
		S2_13679920
		S5_8594000
		S6_46789439
		S2_59237231
		S2_62938215
		S2_63455242
		S9_52533926
		S2_75151214
		S6_56333595
		S4_52459614
		S6_51404165
		S2_61062916
		S1_51003651
		S4_53597075
		S1_1881353
		S1_11427805
		S9_1173313
		S1_62732122
		S7_63217750
		S1_61789083
		S1_8669307
		S4_66042971
		S3_73457384
		S1_74119553
		S9_5152211
		S1_18299390
		S1_55509750
		S5_13388919
		S1_63011666
		S9_52010439
		S3_54663686
		S6_53168216
		S1_61353203
		S3_57760798
		S2_6396282
		S1_19104915
		S5_69893229
		S6_47927547
		S5_1931884
		S1_59152519
		S1_79435089
		S6_35289559
		S1_58284812
		S5_8655333
		S2_16380628
		S9_2687936
		S7_57573965
		S4_7338509
		S2_3950277
		S6_47674395
		S3_69847137
		S8_60452252
		S7_6594209
		S4_5866924
		S2_41616833
		S4_12983819
		S1_77861684
		S1_19099563
		S1_73672714
		S4_51082361
		S8_5140506

Catogoery	Total	SNPs
		S1_6848035
		S6_8907891
		S3_51977257
		S3_70948848
		S2_58394971
		S8_55971198
		S3_57342074
		S2_63467748
		S3_73888179
		S1_24765201
		S6_50609896
		S3_532798
		S3_61329316
		S1_10118771
		S1_21622873
		S3_73631945
		S2_8672940
		S1_16395887
		S1_56030720
		S8_49727293
		S9_54111743
		S7_12416067
		S4_62879182
		S9_1108096
		S3_6748205
		S3_58325374
		S2_65931169
		S1_9078418
		S3_69421196
		S1_28678216
		S8_61956267
		S7_64715094
		S2_59972395
		S1_51232617
		S6_58767164
		S7_64387698
		S3_56378414
		S2_72275606
		S1_67303110
		S4_11520760
		S6_53600141
		S8_40731118
		S5_65909604
		S1_68318544
		S8_61573782
		S9_44414315
		S9_40978346
		S4_5085056
		S2_59276323
		S3_31894951
		S1_2908995
		S9_9793361
		S2_40920934
		S5_59317066
		S9_49963609
		S1_75861019
		S1_79965941
		S1_11472076
		S2_68059566
		S5_59401334
		S4_6805496
		S1_71946542
		S1_6883874
		S5_3022172
		S9_7224708

Catogoery	Total	SNPs
		S2_56877729
		S3_55864263
		S6_58575640
		S2_73584602
		S8_15444581
		S1_67864295
		S6_48638203
		S1_77956940
		S2_76656539
		S7_42246008
		S1_79591397
		S4_7452622
		S4_11177092
		S1_6335856
		S1_67623684
		S1_47595874
		S1_22035545
		S1_9756166
		S1_46062797
		S1_26919001
		S1_53481304
		S3_73437291
		S7_38617665
		S1_77105691
		S3_69391851
		S5_68949782
		S2_59371846
		S7_2907701
		S3_62129057
		S2_66606191
		S2_59032604
		S2_75649806
		S4_3862091
		S2_75410029
		S1_59114893
		S4_46500765
		S2_75894067
		S2_56916082
		S2_12358024
		S8_1172614
		S1_77153016
		S6_52691479
		S4_702519
		S4_67087245
		S5_50559603
		S8_60383352
		S2_46874000
		S8_57571494
		S7_58155373
		S1_57529369
		S4_49808200
		S2_71084072
		S2_65368148
		S9_1064337
		S9_51890206
		S2_63350488
		S2_14360118
		S7_62722684
		S8_15298643
		S8_7197884
		S2_60587300
		S2_60166503
		S5_62156637
		S4_836923
		S1_21035053

Catogoery	Total	SNPs
		S1_62396224
		S9_1720596
		S2_16448227
		S3_60614859
		S3_5202445
		S6_47343552
		S7_7907778
		S8_1840341
		S3_70242188
		S2_76913340
		S5_58139695
		S3_51998930
		S2_18573733
		S1_12945069
		S3_65332757
		S1_11820390
		S1_1558047
		S1_55816200
		S3_1866323
		S6_34044460
		S1_26918982
		S1_14862357
		S6_49333608
		S5_1733030
		S5_11615621
		S3_74059083
		S4_20127120
		S6_38160511
		S2_18990434
		S3_70711190
		S1_72302173
		S7_41316335
		S8_1691541
		S9_52796177
		S2_58887582
		S5_8735490
		S1_74531577
		S9_1176594
		S1_77158703
		S3_1612715
		S6_53622351
		S6_46331289
		S2_3641563
		S7_56590788
		S2_25178173
		S8_61954382
		S1_55104885
		S2_6039513
		S9_2931114
		S1_61546663
		S5_2829054
		S1_57226946
		S8_56642724
		S8_4401950
		S2_10218881
		S1_14437130
		S5_70791605
		S1_80342352
		S7_64959341
		S1_53480581
		S5_65909601
		S8_1699653
		S4_52724301
		S4_2284046
		S4_35837432

Catogoery	Total	SNPs
		S3_58322223
		S8_60484312
		S7_7667258
		S2_76443733
		S8_56635786
		S2_64773224
		S4_10304304
		S9_215188
		S1_74360159
		S6_17315340
		S3_5659698
		S2_719971
		S2_6045459
		S5_20469492
		S1_11708196
		S2_73675555
		S4_24706915
		S8_53997236
		S2_61166571
		S3_72900195
		S5_18256923
		S4_6982850
		S1_60823773
		S2_60025620
		S8_54938546
		S2_62983144
		S1_9485124
		S4_33038814
		S7_15664013
		S2_76427079
		S2_4481983
		S5_68170908
		S8_38514767
		S3_73984994
		S2_10329068
		S4_10219595
		S2_8460031
		S4_6059949
		S1_11753813
		S5_62505748
		S6_60642037
		S1_65333422
		S4_52633385
		S9_54037157
		S1_63345863
		S2_67842683
		S2_12690934
		S3_68561041
		S1_3420278
		S9_7808883
		S7_6861854
		S4_1664586
		S2_55206156
		S7_59829610
		S7_1163213
		S9_8567807
		S1_911914
		S1_59457307
		S8_50152058
		S2_15270202
		S2_65228855
		S1_66564778
		S8_57596100
		S1_59393447
		S2_64350290

Catogoery	Total	SNPs
		S2_41676159
		S8_56141703
		S7_36983555
		S6_60453533
		S2_66701672
		S1_16440034
		S2_73437635
		S1_54500168
		S8_2062998
		S2_10133254
		S1_79028650
		S4_4541663
		S6_46548528
		S8_58488098
		S1_62493791
		S1_53932845
		S4_5107508
		S1_68787886
		S5_2092781
		S2_64563638
		S1_79195280
		S1_78236373
		S2_59240121
		S1_61847951
		S3_58247064
		S5_7341497
		S3_47930491
		S4_67124100
		S1_8181796
		S4_51368429
		S8_37714736
		S2_41116479
		S4_1008392
		S2_30162542
		S3_67082400
		S5_10052188
		S3_55202149
		S3_73321260
		S1_7620143
		S7_54697901
		S1_76240922
		S9_51521890
		S2_71595664
		S1_59558549
		S6_57413550
		S9_1110948
		S9_51591073
		S1_52708405
		S9_57434537
		S2_59538553
		S2_10832252
		S9_214850
		S4_12312580
		S9_50150048
		S1_66309634
		S2_2574942
		S7_490172
		S2_12754266
		S8_55144772
		S3_51506182
		S6_48608521
		S7_9729237
		S3_52046028
		S9_55626961
		S9_56031806

Catogoery	Total	SNPs
		S2_7829324
		S3_60919676
		S5_6101763
		S1_66791897
		S2_65280420
		S9_2884173
		S5_6051668
		S6_46327582
		S9_51178123
		S3_68919624
		S2_68021462
		S6_55700776
		S6_54203502
		S1_6537508
		S1_79932518
		S2_2382977
		S1_19067228
		S2_75603758
		S7_64727038
		S2_12757426
		S2_65882021
		S5_11465033
		S3_71232254
		S3_47963519
		S1_54555818
		S5_12294695
		S6_47570763
		S2_60529284
		S3_54214285
		S3_51804986
		S3_68199693
		S2_11658414
		S3_72187327
		S1_12305635
		S6_45146401
		S8_54852752
		S7_39762751
		S1_65216736
		S4_56425879
		S7_7936857
		S6_48246942
		S2_66967851
		S1_65259508
		S1_58165393
		S5_3384253
		S3_60599096
		S8_52025100
		S2_73366068
		S1_15897109
		S3_69848803
		S1_7640553
		S1_7509934
		S4_11092375
		S1_13471930
		S3_65910297
		S3_71232378
		S1_74652837
		S1_71831805
		S6_17967319
		S9_1072581
		S2_5556610
		S1_58204310
		S2_19732577
		S6_49469761
		S9_50874704

Catogoery	Total	SNPs
		S2_49671038
		S4_4943931
		S4_10419275
		S2_61060370
		S1_8034061
		S1_67679409
		S2_6542872
		S1_11169829
		S1_67679856
		S3_71247742
		S3_54260831
		S2_8111648
		S8_48975413
		S5_6176881
		S7_55763000
		S8_50018668
		S3_5644998
		S4_54062988
		S8_4445191
		S1_27444223
		S2_45816751
		S8_56232150
		S4_35465158
		S2_64287872
		S7_63586214
		S6_53761264
		S6_47626711
		S1_7115561
		S3_67154245
		S2_6036403
		S1_10689324
		S1_51003473
		S1_7229869
		S4_53381282
		S1_24741102
		S2_63186531
		S1_75685810
		S1_56295202
		S1_11198831
		S1_15302676
		S3_57760891
		S7_2009483
		S7_39251252
		S9_21055789
		S6_47418752
		S8_56542075
		S1_62950583
		S2_10329830
		S3_70711183
		S1_16455690
		S4_7277287
		S5_23083722
		S4_21050159
		S1_4083292
		S7_3930604
		S9_2773544
		S3_67873007
		S2_14775587
		S2_3333900
		S3_62361920
		S3_70038761
		S2_14797961
		S1_12197518
		S9_2619748
		S2_74867472

Catogoery	Total	SNPs
		S3_62406846
		S8_51183222
		S8_42209069
		S2_75153938
		S1_61730074
		S9_8623315
		S4_67160686
		S1_56604705
		S6_48690929
		S6_53938559
		S3_51838824
		S2_56538510
		S3_62564989
		S7_1279756
		S4_22715354
		S3_69832091
		S2_41395957
		S8_60207218
		S3_63071478
		S4_41454677
		S2_5824581
		S1_77183323
		S5_59401327
		S7_60487952
		S8_56538317
		S1_57092406
		S4_3715822
		S1_71994494
		S2_75975458
		S2_19476852
		S3_71926899
		S2_5705876
		S2_64350269
		S4_7668477
		S1_58192512
		S7_614433
		S1_8879733
		S2_52195636
		S1_6331790
		S6_53399556
		S4_39240806
		S9_4238665
		S9_6008673
		S3_73271598
		S1_13324687
		S6_54335237
		S8_16230528
		S4_58459238
		S1_71836550
		S2_12498605
		S8_1558777
		S5_6525290
		S5_1966412
		S5_2547658
		S7_64235979
		S1_77424002
		S2_75594115
		S6_35584098
		S2_8892376
		S1_11238370
		S8_32316988
		S4_14843533
		S2_6230170
		S6_50489251
		S8_61956252

Catogoery	Total	SNPs
		S1_59699960
		S2_66173304
		S6_52964331
		S1_58840405
		S2_72604685
		S3_60614847
		S3_67541353
		S6_55689760
		S2_12746305
		S1_64832960
		S1_24602897
		S8_42616073
		S2_74974016
		S1_76479740
		S7_62237204
		S1_7620707
		S7_62396521
		S4_13717841
		S7_61085965
		S1_56295006
		S1_73037148
		S2_59371894
		S1_6640248
		S9_58179030
		S6_52882996
		S3_57269911
		S1_78711863
		S2_61405339
		S1_26241771
		S2_73308341
		S6_51865297
		S6_16399734
		S7_53165088
		S1_59477815
		S9_52926561
		S2_58290731
		S6_50657531
		S4_26502645
		S2_3641627
		S8_4542292
		S2_7685693
		S3_64853283
		S2_58868320
		S4_56526571
		S1_3461757
		S1_3472477
		S2_59247356
		S5_8848246
		S1_74871004
		S2_9887291
		S5_63226057
		S4_20901103
		S5_63115674
		S7_5944330
		S8_60565276
		S2_72258954
		S7_62494215
		S9_57051085
		S6_55277730
		S9_50744164
		S7_59417731
		S2_76812936
		S6_50704210
		S8_2313213
		S2_60973403

Catogoery	Total	SNPs
		S2_56332752
		S3_38419868
		S9_54825465
		S1_4472359
		S9_6579472
		S2_16883657
		S4_55504242
		S4_48385711
		S2_64350286
		S5_62499700
		S2_54200583
		S4_53816486
		S5_1575470
		S1_13401162
		S3_4612565
		S5_665446
		S7_40999382
		S9_57152535
		S1_65672253
		S1_1558123
		S5_62950702
		S5_8789373
		S3_61329303
		S9_6366932
		S1_7804247
		S8_56411888
		S9_53396303
		S4_19466353
		S1_53903785
		S1_55817588
		S6_46372064
		S2_59755617
		S6_45640371
		S6_50286342
		S1_11604684
		S3_51905588
		S2_48994233
		S2_49667967
		S6_46718856
		S2_9445970
		S5_66098790
		S7_946508
		S1_17585659
		S7_58526177
		S8_55275193
		S9_6054644
		S9_57152442
		S4_54095472
		S4_2626984
		S1_60912509
		S1_26784236
		S8_49628041
		S2_61110102
		S9_9079282
		S6_42463480
		S2_3998285
		S1_57369312
		S9_50153470
		S1_55509767
		S2_16338963
		S6_3543026
		S2_73366261
		S3_57750561
		S9_52073343
		S2_1585997

Catogoery	Total	SNPs
		S4_65550373
		S4_12350798
		S7_8817901
		S3_67534159
		S2_6482508
		S2_9638839
		S4_50974937
		S2_63770458
		S3_59308284
		S1_66564835
		S8_18161225
		S6_14514588
		S7_484182
		S2_68095422
		S3_72268850
		S5_9397610
		S5_41622059
		S2_63549255
		S6_50925319
		S7_14459274
		S2_76513419
		S4_42741229
		S7_6633806
		S1_16774630
		S2_9463527
		S4_19726688
		S2_12670127
		S5_44191298
		S9_45618449
		S5_68825409
		S5_68429737
		S3_63313759
		S5_61610871
		S9_41603385
		S3_54091200
		S1_53976522
		S8_51865759
		S5_67427825
		S7_58746449
		S3_72504726
		S3_2197663
		S2_14786705
		S1_15851565
		S9_52707498
		S7_7590325
		S5_66374603
		S3_71232191
		S5_50601109
		S7_59043280
		S2_61685832
		S2_61053324
		S1_78749830
		S1_60760862
		S6_35567853
		S1_1950580
		S7_64522289
		S1_56241975
		S1_53854293
		S1_64993132
		S1_1564728
		S2_2694888
		S1_13015965
		S9_2663313
		S3_56905800
		S7_62611894

Catogoery	Total	SNPs
		S4_18802013
		S8_44769095
		S8_9653833
		S9_6484245
		S1_8998937
		S7_63553864
		S1_78528692
		S1_78961276
		S9_2228538
		S4_13491112
		S9_1750359
		S6_58739491
		S9_972209
		S1_71699603
		S6_58895014
		S2_60441055
		S1_60720931
		S1_62915583
		S3_2500312
		S9_4037875
		S3_14076828
		S1_6314150
		S4_46961002
		S5_15774660
		S3_51905751
		S6_58178561
		S8_44424521
		S8_35515965
		S3_70854519
		S1_67679349
		S4_4350641
		S1_19552902
		S4_56690809
		S3_6066202
		S2_62095908
		S2_5624818
		S8_56847140
		S3_14047815
		S2_32127431
		S8_50345776
		S4_26735364
		S7_58679364
		S5_62922697
		S5_12445825
		S3_62273241
		S1_72721931
		S5_66405156
		S4_464150
		S3_73881426
		S2_58887620
		S8_34672849
		S1_72277024
		S1_19104819
		S4_2217194
		S8_9730760
		S8_4480760
		S5_2640974
		S5_70045203
		S6_55684265
		S6_52777858
		S4_2650396
		S7_277812
		S2_63938287
		S5_5827150
		S8_51123931

Catogoery	Total	SNPs
		S3_54138326
		S5_68589521
		S5_5349939
		S9_49589820
		S2_62709023
		S5_7660669
		S2_75420934
		S4_52500356
		S2_61427933
		S1_58194753
		S5_12827487
		S2_3602284
		S3_14209267
		S2_62974129
		S5_62418982
		S9_4087521
		S4_9368765
		S3_64892600
		S4_61245757
		S2_8713284
		S4_9863006
		#N/A
		S2_6999145
		S4_7539842
		S4_25324588
		S9_9658600
		S8_34777494
		S1_18041328
		S5_15885817
		S1_57882862
		S1_65380831
		S5_66405051
		S2_66342905
		S4_67931669
		S2_59441647
		S5_71135323
		S7_52808212
		S4_34653111
		S3_62847092
		S1_52089027
		S6_49676499
		S6_60774038
		S2_67222574
		S2_6742074
		S2_60166328
		S7_59026164
		S9_59408470
		S7_59980984
		S6_42508334
		S1_80030411
		S9_47005992
		S8_1699194
		S5_11014866
		S8_48141968
		S7_12011818
		S3_71926570
		S1_67059671
		S3_56869309
		S7_6984854
		S7_62217660
		S4_8885598
		S2_8230918
		S1_8920631
		S1_64629058
		S5_63419064

Catogoery	Total	SNPs
		S6_52704343
		S4_1042511
		S6_1238535
		S9_1202162
		S3_68316972
		S4_53052857
		S4_15096639
		S9_7141855
		S2_6426060
		S8_61636549
		S3_53418611
		S1_8078530
		S1_67681240
		S3_65626321
		S1_72303354
		S7_63639811
		S1_48971890
		S5_9500870
		S2_77540789
		#N/A
		S4_52949048
		S2_16380631
		S2_68954479
		S1_9063641
		S1_20249772
		S9_57657558
		S2_64256814
		S1_57535860
		S5_62396377
		S1_19170594
		S2_69593728
		S4_41444242
		S2_65452009
		S5_68797764
		S8_61165324
		S3_61946051
		S6_50583829
		S3_3902063
		S2_10289216
		S5_12084309
		S9_1186735
		S1_59984932
		S1_32151961
		S8_2906004
		S2_8430568
		S8_49156093
		S1_8819524
		S8_48961259
		S5_5828244
		S1_68188447
		S1_19203870
		S7_60654665
		S9_51523708
		S5_3818319
		S1_59471501
		S4_12354575
		S6_46819376
		S1_50221757
		S4_4540956
		S2_5915571
		S3_69810209
		S5_1730801
		S5_13004777
		S3_66415878
		S4_51491905

Catogoery	Total	SNPs
		S6_47920292
		S5_61161450
		S2_65823099
		S7_15545781
		S5_10213609
		S2_455671
		S5_62378641
		S2_52405670
		S7_17303038
		S2_61153277
		S3_61017877
		S1_62732120
		S8_45237483
		S9_52079076
		S1_53974671
		S2_47603964
		S3_6756029
		S6_34253786
		S6_39668629
		S1_3156776
		S7_425715
		S8_58488041
		S4_11117662
		S3_71419965
		S5_6769951
		S7_2562555
		S8_60548859
		S2_1510569
		S9_8179133
		S5_61168362
		S7_537127
		S4_4189597
		S2_62051192
		S3_70857076
		S2_58007163
		S2_58202067
		S1_76496174
		S9_10160431
		S1_7640594
		S7_53162915
		S7_65081302
		S3_68983609
		S6_45079171
		S6_45144497
		S3_68038134
		S2_1474519
		S7_7574386
		S6_17957479
		S5_69776801
		S9_4637489
		S3_13584967
		S2_49470479
		S6_1566749
		S3_58846551
		S6_36174905
		S5_58523229
		S3_59688104
		S3_5404220
		S1_77830589
		S1_57508712
		S1_78182502
		S6_44072827
		S1_12758661
		S7_57111416
		S7_38852181

Catogoery	Total	SNPs
		S9_58315566
		S5_2829042
		S5_11195207
		S1_60147909
		S4_5560911
		S9_3540335
		S8_1991732
		S3_5521252
		S9_4686707
		S1_66553149
		S1_6223623
		S4_66158425
		S5_62646212
		S6_5980566
		S2_25439145
		S8_4402128
		S9_44414313
		S8_2306131
		S3_47935471
		S1_79194061
		S5_3044702
		S8_53211227
		S2_56440842
		S2_66633691
		S3_69421193
		S9_50538298
		S4_7276625
		S1_57816557
		S6_54390549
		S9_9486757
		S1_28062231
		S4_12708767
		S8_1397393
		S1_11639225
		S4_51082365
		S2_66173394
		S1_6777453
		S3_56448502
		S5_6868781
		S6_45540289
		S2_4382659
		S5_63240157
		S8_54927853
		S3_52836380
		S4_4541943
		S1_66791922
		S3_3700878
		S6_15180220
		S7_63982653
		S6_44605263
		S8_60010349
		S4_5907418
		S1_7093623
		S2_73693409
		S3_72851944
		S2_40078271
		S6_20647817
		S9_48237394
		S9_54591807
		S6_31790973
		S9_2931127
		S4_2292611
		S7_52314512
		S1_78724997
		S8_48961389

Catogoery	Total	SNPs
		S1_52643103
		S6_58831836
		S1_62967259
		S5_62376096
		S2_47500875
		S1_16641361
		S4_37014480
		S2_69155242
		S1_75513420
		S8_3230399
		S4_51772571
		S5_4319859
		S9_6690484
		S3_72541552
		S9_47005965
		S3_71247687
		S3_68919614
		S3_621147
		S5_11190831
		S1_19090501
		S8_49763461
		S3_5659699
		S4_9322342
		S1_56319014
		S1_6846195
		S1_20207362
		S2_7090894
		S9_59302926
		S6_49438539
		S2_14798025
		S2_8713306
		S2_59878127
		S2_426295
		S6_34041118
		S1_67715937
		S3_5535640
		S1_60758042
		S9_52073503
		S2_59247255
		S2_75613747
		S9_8512868
		S7_4554675
		S8_2068116
		S2_12690959
		S5_63500295
		S9_1658621
		S5_9430964
		S1_79961937
		S5_4616939
		S2_76887945
		S5_70796994
		S9_52245999
		S8_46044882
		S6_60926392
		S4_53085009
		S1_52350737
		S5_69822766
		S4_62932125
		S5_67566864
		S4_6125080
		S9_57629289
		S3_12719986
		S9_41427211
		S6_17055055
		S1_72374563

Catogoery	Total	SNPs
		S1_72663093
		S4_5536033
		S2_65451794
		S1_74939956
		S5_5045665
		S1_75620806
		S2_73498189
		S4_2222115
		S6_47181226
		S1_56549349
		S7_7841736
		S6_47955707
		S5_13005777
		S3_70581399
		S2_75893553
		S6_50696784
		S9_56116031
		S8_60168121
		S8_53528550
		S4_7301490
		S2_69327061
		S8_1238618
		S5_63419179
		S4_67110277
		S3_3666388
		S4_2151617
		S3_52258863
		S2_67837513
		S6_1586946
		S1_54031581
		S2_5766341
		S2_61857953
		S2_59125995
		S3_5606514
		S4_41400624
		S4_6465633
		S8_5460230
		S2_66675957
		S3_2360104
		S2_9854708
		S8_61541273
		S2_10189359
		S8_60362308
		S1_52089015
		S4_5107567
		S1_60720832
		S7_62194048
		S2_29555322
		S2_8264061
		S6_47043867
		S3_61511135
		S5_614154
		S5_70784704
		S5_2920250
		S5_69854747
		S7_54785628
		S4_52591185
		S9_57247174
		S4_12360026
		S6_1587117
		S6_52350684
		S4_6805620
		S2_77014526
		S5_69780354
		S5_61208162

Catogoery	Total	SNPs
		S1_71946549
		S4_958815
		S6_45989925
		S1_57591743
		S1_60837204
		S3_68014942
		S5_10900180
		S6_37943359
		S8_51525720
		S5_69851975
		S3_70630040
		S9_1202123
		S2_59045052
		S9_4365943
		S5_7641857
		S2_63811592
		S7_1571253
		S1_11562511
		S1_28670342
		S2_76499370
		S1_60626348
		S5_62918801
		S3_56545937
		S8_5379893
		S8_61606862
		S6_5924588
		S9_52915107
		S1_66553165
		S5_6675014
		S6_46372043
		S8_58455752
		S7_62217641
		S1_13401493
		S9_50744165
		S3_2480144
		S2_16428563
		S6_50331377
		S2_68931638
		S4_52440822
		S4_10304304
		S9_51277331
		S1_56561147
		S1_31205566
		S7_61539222
		S1_79793862
		S3_73887699
		S2_77007654
		S2_59921835
		S7_41781831
		S9_42359056
		S8_34312144
		S6_15439256
		S1_46939349
		S3_55375549
		S2_76197451
		S4_6982841
		S3_2070656
		S2_62313240
		S3_54709457
		S1_61547926
		S7_61083044
		S9_5862637
		S8_44421149
		S8_61954357
		S9_955352

Catogoery	Total	SNPs
		S1_1806213
		S2_62210938
		S2_10286024
		S8_4994009
		S5_67567207
		S2_71595604
		S2_63811616
		S2_61435639
		S5_1645052
		S7_55762900
		S2_60166517
		S8_56707237
		S5_63114322
		S2_61153287
		S3_54546385
		S2_67621081
		S4_52500456
		S3_5565466
		S2_61079646
		S3_61052935
		S6_1668314
		S2_29767549
		S4_12948172
		S2_66092481
		S4_2637694
		S6_51510561
		S9_1719163
		S7_493485
		S4_10811820
		S8_60488372
		S2_74073520
		S2_2263929
		S2_12746327
		S9_49290867
		S2_77607245
		S8_59749665
		S6_5878721
		S4_2484249
		S2_77031071
		S4_68010994
		S6_45262259
		S2_64334145
		S2_8166685
		S6_40987299
		S6_3463645
		S1_71946414
		S2_72612926
		S5_8068934
		S6_50583842
		S9_48956453
		S3_4576591
		S1_68247464
		S2_75151138
		S2_66606182
		S3_58426017
		S2_4149581
		S2_57862871
		S8_49581737
		S1_22260261
		S1_73463724
		S6_55746365
		S2_5389768
		S1_57378891
		S2_5384574
		S1_31874344

Catogoery	Total	SNPs
		S8_53521957
		S8_55614044
		S4_49719514
		S4_16075018
		S1_74523126
		S2_67753426
		S1_72898960
		S1_20178914
		S2_60166327
		S1_56444091
		S2_7594612
		S6_6466683
		S4_61180173
		S8_61636550
		S1_7961525
		S2_66469908
		S8_61980172
		S1_20313529
		S4_7961403
		S6_46551985
		S9_54081197
		S6_2023768
		S3_68981506
		S5_42766675
		S1_69096333
		S1_65255242
		S3_53323893
		S8_1991813
		S1_66238562
		S7_4559660
		S8_4542286
		S2_388603
		S2_66684321
		S3_54444363
		S2_64350284
		S6_54617144
		S2_59198427
		S2_54244649
		S9_9895096
		S9_52595111
		S4_43758956
		S6_42508335
		S8_56707189
		S1_24602656
		S5_8848244
		S6_47901265
		S3_72096072
		S2_64335808
		S6_58548345
		S5_63419062
		S1_11785211
		S5_6065434
		S1_2383264
		S9_56521102
		S8_42616078
		S1_9485301
		S5_66735262
		S6_2190888
		S2_10219275
		S9_2530175
		S4_2234438
		S3_38419872
		S2_57849447
		S1_62552543
		S9_58471643

Catogoery	Total	SNPs
		S3_69550952
		S1_58192514
		S2_59754253
		S2_72265472
		S1_66571295
		S5_5036249
		S8_34314314
		S2_67765552
		S3_56274509
		S1_11127578
		S2_8908541
		S4_8110171
		S8_60544600
		S8_5320753
		S4_56314566
		S1_66541914
		S6_34041196
		S7_60488156
		S3_58846980
		S2_56916036
		S5_66607805
		S2_6101615
		S1_59383256
		S1_72978923
		S4_6937207
		S4_7539069
		S2_8465240
		S2_62729860
		S1_51240453
		S6_53254399
		S2_69151661
		S1_7822994
		S3_5626007
		S1_15031506
		S9_8213286
		S5_14317684
		S1_59855676
		S7_38848503
		S1_71592571
		S9_8758472
		S8_1929008
		S1_78290735
		S6_47955898
		S2_63140337
		S2_16908340
		S1_17468703
		S1_60677933
		S4_13983788
		S3_72337404
		S3_71370674
		S2_66185301
		S5_61161563
		S8_58744275
		S2_10527936
		S4_55800445
		S1_29777711
		S6_35665080
		S3_57945448
		S3_61711082
		S6_58834159
		S8_17494938
		S8_3173777
		S8_61361343
		S2_66372868
		S2_61802667

Catogoery	Total	SNPs
		S6_53396986
		S2_76499200
		S8_2016336
		S8_58676468
		S2_62729853
		S2_6204342
		S5_68792057
		S6_41925274
		S9_55538776
		S3_48311974
		S9_51448772
		S3_70239277
		S4_22798184
		S9_47049412
		S3_70256140
		S2_5507288
		S8_57427012
		S6_16524419
		S6_44561302
		S2_12102140
		S4_3654663
		S3_72257560
		S3_62459758
		S7_15757049
		S7_6580377
		S4_3668113
		S6_38160463
		S5_65920015
		S3_64927458
		S4_6173942
		S3_3480332
		S6_58812612
		S4_10306161
		S3_4270600
		S1_10635448
		S2_16479990
		S2_63687680
		S2_8432703
		S1_30406457
		S6_2578875
		S2_56955027
		S1_19645395
		S6_41351918
		S2_64350274
		S2_72264268
		S4_26502903
		S4_3905746
		S2_17954478
		S7_722379
		S4_7890027
		S1_75521588
		#N/A
		S3_69847054
		S4_24704452
		S6_7198229
		S3_73031220
		S9_18304692
		S1_77361126
		S2_2831293
		S2_9463504
		S9_7462956
		S5_12303580
		S6_3914314
		S7_61525355
		S3_67966301

Catogoery	Total	SNPs
		S2_60296868
		S2_75364741
		S4_16234312
		S1_12922220
		S9_141062
		S1_17374356
		S9_4087687
		S2_69602097
		S6_26301547
		S5_68111696
		S1_6687552
		S2_6541578
		S1_61008560
		S4_53842345
		S9_55067186
		S9_57463613
		S1_74530890
		S2_10752422
		S1_77671533
		S9_140972
		S5_58548707
		S5_14213458
		S1_47698720
		S4_52380438
		S7_62712231
		S2_58017187
		S9_55969244
		S3_57516482
		S1_64482182
		S1_23805661
		S6_61062977
		S1_5806902
		S4_5893812
		S1_19154355
		S1_63714611
		S8_1430445
		S1_72713577
		S3_6247923
		S3_57213873
		S2_8111629
		S8_61641313
		S5_65524682
		S2_58021501
		S1_79195283
		S4_12639677
		S3_53551117
		S1_61856969
		S4_7790846
		S7_65063902
		S1_28775097
		S5_2696571
		S1_4473602
		S5_58573182
		S2_11106595
		S8_2895454
		S4_6415156
		S3_47964955
		S7_3694752
		S1_67476980
		S3_73432637
		S1_66382854
		S5_62388688
		S2_57728578
		S8_4280319
		S9_51890182

Catogoery	Total	SNPs
		S1_11686440
		S6_29733756
		S2_4696247
		S2_58538061
		S5_10818600
		S9_5036438
		S3_68982836
		S8_57430426
		S3_20545470
		S9_5355893
		S1_74084269
		S7_9694814
		S2_53473974
		S5_5366257
		S1_11127572
		S1_14330612
		S1_75393118
		S3_59368478
		S1_8740039
		S3_528499
		S7_2743149
		S2_9528935
		S7_58155361
		S1_75399294
		#N/A
		S1_78292927
		S5_66997926
		S4_1644280
		S7_56556536
		S6_6459342
		S9_51180661
		S2_4555000
		S8_51713935
		S3_72414665
		S6_58575355
		S3_2117224
		S1_3362985
		S1_64409056
		S9_47049871
		S6_53168384
		S1_79532538
		S6_52765265
		S2_16272024
		S4_53613308
		S1_11686454
		S6_27965484
		S1_16455741
		S1_14606421
		S8_58151619
		S6_53167049
		S6_6378099
		S2_460605
		S8_44870360
		S4_7169865
		S7_15415170
		S1_78161528
		S3_3776191
		S2_59238579
		S1_10734488
		S2_3808827
		S8_55494921
		S7_55762996
		S2_29767306
		S1_20358341
		S8_60932764

Catogoery	Total	SNPs
		S2_71486248
		S8_56103653
		S1_72859911
		S5_66386693
		S1_18956184
		S4_58269915
		S1_16399140
		S7_2413167
		S5_6101723
		S6_52583042
		S1_58208084
		S5_24851642
		S8_60169742
		S8_1839685
		S5_4803848
		S2_16406507
		S6_40434381
		S1_12666034
		S4_2233804
		S2_73666179
		S2_63387964
		S1_18853214
		S1_9068983
		S5_11014816
		S3_59633911
		S2_6999129
		S1_2901337
		S9_52527630
		S2_66173283
		S6_45570597
		S7_5535113
		S2_44536732
		S6_56333665
		S2_66066889
		S4_6008770
		S3_1935948
		S7_1639845
		S3_65718957
		S1_30465481
		S5_58548616
		S8_56411727
		S4_10710749
		S1_20350064
		S1_10286622
		S5_9535556
		S2_9466653
		S1_57102005
		S1_7390203
		S3_58173356
		S9_5771328
		S6_54302430
		S2_73675559
		S5_20978360
		S4_39246309
		S3_58877251
		S4_59507416
		S4_61175065
		S2_61368331
		S4_37432159
		S5_62509873
		S9_52313910
		S3_71378067
		S8_51868277
		S6_46718550
		S9_51892812

Catogoery	Total	SNPs
		S4_1669751
		S2_65383879
		S6_50487409
		S5_14948555
		S8_5242285
		S2_61333291
		S1_8099563
		S9_7538586
		S2_65888528
		S1_24000148
		S1_71997870
		S4_6288578
		S1_22490283
		S1_19017832
		S1_70086137
		S8_61768111
		S2_65028933
		S4_2893831
		S4_5085180
		S1_80487284
		S9_6568939
		S1_61606097
		S5_8796837
		S1_4111480
		S5_65909628
		S3_63142478
		S4_1120549
		S7_39076799
		S1_74876558
		S4_37131582
		S2_58007078
		S3_1871279
		S3_1227967
		S3_67782658
		S1_77184252
		S9_42348900
		S1_77750923
		S3_52014426
		S9_3881607
		S2_6550639
		S4_39534519
		S1_6876611
		S4_4321084
		S7_48385050
		S3_51972073
		S3_62588196
		S5_8111475
		S2_69037655
		S6_31579657
		S6_47925217
		S4_42197033
		S3_6692636
		S7_54775634
		S2_76831998
		S2_56056716
		S7_39882149
		S3_69365324
		S7_63118561
		S1_18956124
		S1_11127533
		S1_72937264
		S3_71627258
		S1_11127576
		S1_1547295
		S1_79508608

Catogoery	Total	SNPs
		S2_61785890
		S9_1258259
		S6_55689766
		S8_54218803
		S3_70657985
		S2_59642481
		S3_70581405
		S7_54684566
		S1_54392967
		S7_63712273
		S2_60441051
		S5_1971438
		S9_54346799
		S1_46548083
		S2_8672939
		S7_52328210
		S3_55276632
		S3_20013825
		S4_53344059
		S9_2931134
		S3_57350261
		S1_53974668
		S1_55917478
		S5_11314290
		S2_65881997
		S4_51278084
		S1_10408833
		S8_56411891
		S7_64750995
		S4_11520895
		S8_31558412
		S2_47145721
		S6_2712196
		S3_63301488
		S1_71831800
		S6_50964460
		S5_11358386
		S5_24345538
		S7_6700728
		S9_2687828
		S5_67567201
		S8_48678095
		S1_78489461
		S1_5800653
		S5_24020387
		S4_2190303
		S9_8512785
		S3_811418
		S7_311484
		S4_38154351
		S3_6000890
		S1_75035441
		S8_57039692
		S1_24784784
		S1_10882565
		S1_18956156
		S2_49652668
		S9_1260306
		S6_54381332
		S8_60393371
		S1_59567011
		S5_41904856
		S9_43100642
		S4_37132639
		S1_11755228

Catogoery	Total	SNPs
		S3_56869298
		S1_15866623
		S2_6890936
		S3_64831766
		S8_50345773
		S6_29503656
		S2_8707785
		S6_41588285
		S1_47839158
		S2_65965885
		S6_41593016
		S2_63140283
		S1_11753642
		S4_1108637
		S1_12968339
		S2_70923266
		S1_1640930
		S4_9900288
		S1_54032095
		S2_8451159
		S1_59966372
		S1_7436909
		S1_67679853
		S6_52694363
		S9_52824281
		S1_68236729
		S5_3184097
		S8_5157214
		S6_60453529
		S4_58254291
		S5_1836355
		S6_55684144
		S5_1729037
		S2_65504221
		S1_79961938
		S5_4006132
		S1_16415222
		S1_66322622
		S1_12662703
		S1_62967232
		S8_3429289
		S9_50538301
		S9_6791209
		S2_66185251
		S9_52335675
		S3_48834323
		S4_836918
		S3_13673312
		S7_8427369
		S1_57087863
		S8_57485729
		S5_21577177
		S2_50271912
		S9_49647214
		S8_60349261
		S6_31059831
		S2_73494268
		S7_5535109
		S5_3621087
		S3_71412250
		S3_72063882
		S4_52500357
		S2_57083772
		S6_2017946
		S6_3876513

Catogoery	Total	SNPs
		S6_52713831
		S2_6178219
		S4_7277449
		S2_3754352
		S8_51921700
		S6_36503458
		S9_51068876
		S1_75999833
		S4_67124097
		S3_72321764
		S7_41999335
		S2_58118894
		S4_5107562
		S9_58675219
		S5_2776587
		S9_3944153
		S1_71689034
		S2_10976538
		S6_47197520
		S1_73735400
		S2_13177160
		S4_50982592
		S9_41580225
		S3_56913053
		S2_77492952
		S8_60323631
		S8_2884925
		S5_3859510
		S4_6756681
		S3_57437973
		S5_61208224
		S8_47079736
		S5_11615568
		S7_63699595
		S3_56580180
		S4_4004790
		S6_2598774
		S4_4108894
		S3_61379084
		S4_6899200
		S1_78715911
		S7_16679302
		S6_61045772
		S2_11055053
		S1_10548272
		S7_887791
		S3_57921758
		S2_47145736
		S1_62915588
		S5_70791600
		S9_49964992
		S4_12792064
		S6_53756525
		S2_62726543
		S2_77014570
		S4_12409977
		S1_18877545
		S9_6761401
		S4_2627663
		S2_58202075
		S5_69890988
		S9_58114146
		S1_9665452
		S1_8816782
		S7_62605107

Catogoery	Total	SNPs
		S3_73084319
		S1_6040040
		S6_50436112
		S7_39882164
		S1_59009856
		S8_3895940
		S4_11136716
		S7_54307722
		S9_58603696
		S3_64460184
		S1_57666319
		S8_57810509
		S3_69900015
		S1_21476874
		S2_73187617
		S6_49399711
		S6_58767278
		S3_3757317
		S7_5171833
		S4_5855188
		S4_2504899
		S4_58269924
		S9_41572719
		S3_73968343
		S1_19122187
		S8_51017650
		S1_75403698
		S2_13459029
		S3_15625513
		S6_53931702
		S1_19376024
		S9_50014563
		S3_72900155
		S7_1630103
		S6_35453828
		S8_56115054
		S1_79005172
		S3_71018333
		S7_6594079
		S3_58220439
		S4_9434671
		S7_704393
		S5_11328782
		S6_60707194
		S9_5167705
		S6_26356814
		S9_4249082
		S2_57258290
		S3_73925036
		S2_58201045
		S5_62418995
		S3_59652744
		S1_17426837
		S4_33300775
		S3_57894603
		S2_65386118
		S8_1563150
		S7_55993142
		S2_64476786
		S4_66114153
		S5_65909609
		S1_68834396
		S5_61128740
		S6_51971883
		S2_13177373

Catogoery	Total	SNPs
		S8_1172618
		S1_18880782
		S9_51051466
		S1_72938607
		S4_52350714
		S4_48058365
		S2_16961370
		S7_63318989
		S2_8936707
		S1_78496212
		S2_10840308
		S7_42227184
		S9_196999
		S1_11174373
		S4_8886675
		S1_9066122
		S5_11792497
		S1_59180899
		S5_6809728
		S6_45989916
		S6_49875883
		S9_51545804
		S5_69847993
		S8_61980131
		S5_66999988
		S7_55982056
		S9_41316621
		S6_27217429
		S9_1111019
		S2_26494606
		S1_78032501
		S4_52307610
		S5_13605640
		S8_1800909
		S5_59271708
		S7_65097466
		S7_8583692
		S7_56555966
		S6_48041527
		S1_4472217
		S1_46165374
		S5_2693244
		S1_17106145
		S1_62720928
		S4_7539931
		S3_13436139
		S2_55206179
		S8_47589378
		S6_46815206
		S4_1664595
		S6_46372066
		S9_52654148
		S4_51878532
		S1_2054165
		S3_52245055
		S8_57233053
		S8_61541273
		S1_76644101
		S2_4380075
		S5_58436056
		S4_7341348
		S2_7703707
		S5_69943654
		S6_54784875
		S8_53940562

Catogoery	Total	SNPs
		S9_57785001
		S1_56030218
		S3_5505101
		S6_41007969
		S3_70902112
		S3_6350905
		S4_1645468
		S4_15107910
		S7_6922579
		S2_72264308
		S5_1481148
		S1_69924265
		S7_54371363
		S6_1233802
		S4_12075115
		S1_73446733
		S2_60118381
		S3_13445863
		S5_6962992
		S2_72630425
		S6_38191428
		S5_9814720
		S2_61427944
		S4_54186761
		S1_14285575
		S3_71232305
		S5_3626699
		S9_58179020
		S3_542076
		S8_56144774
		S1_57665470
		S4_51276410
		S1_72010036
		S4_22984120
		S8_50405742
		S1_1641080
		S5_61779947
		S8_42702383
		S3_66421777
		S3_57546532
		S1_8030714
		S5_8727625
		S2_73516749
		S2_61785853
		S6_60926385
		S1_60523777
		S5_15116557
		S7_63365633
		S3_63201732
		S2_62117394
		S7_63639736
		S2_62315828
		S7_61479086
		S7_62611641
		S9_52078336
		S3_56503358
		S3_61330360
		S6_41984832
		S1_51860544
		S3_71503525
		S5_6862847
		S1_11197144
		S6_38341789
		S6_47698109
		S4_7538559

Catogoery	Total	SNPs
		S1_21201436
		S7_5919923
		S9_54947164
		S2_17982378
		S3_4956345
		S3_69397378
		S4_53748207
		S1_19640912
		S5_61161421
		S5_67495128
		S4_61297110
		S1_68318606
		S6_49221316
		S1_7598857
		S1_68222646
		S2_75808181
		S8_29984545
		S2_60448663
		S3_4608884
		S6_5727412
		S1_14150865
		S1_59131351
		S2_69809431
		S7_39941570
		S2_63355958
		S1_60954417
		S1_56444084
		S5_4752843
		S6_14820911
		S1_9860647
		S4_51368484
		S9_54094859
		S6_1083465
		S7_61377715
		S1_56353408
		S7_62609663
		S7_537124
		S6_58831835
		S8_1979401
		S4_22341447
		S2_58541910
		S6_41273706
		S1_17468687
		S7_19191457
		S3_68981768
		S6_52620108
		S8_61356263
		S3_70252880
		S1_55102894
		S1_54500135
		S6_58767475
		S2_67952188
		S1_7120709
		S1_55900078
		S2_2323584
		S5_66267740
		S4_5107458
		S2_3251752
		S2_67335192
		S5_4616948
		S8_60349065
		S5_58574453
		S5_67722102
		S1_71831811
		S5_2181327

Catogoery	Total	SNPs
		S9_54898712
		S2_61110132
		S1_9822628
		S6_53558936
		S3_61017879
		S2_10753141
		S5_4258130
		S9_24554842
		S5_69812733
		S6_47065476
		S3_73485371
		S5_11364551
		S2_66365898
		S8_1325615
		S1_22035634
		S5_62418969
		S2_56964225
		S9_31020957
		S2_7878110
		S2_77505473
		S2_6526553
		S3_48834863
		S2_69327057
		S7_62377715
		S3_67154241
		S1_77782180
		S3_73239694
		S2_60131032
		S1_56994934
		S2_10200355
		S7_9189398
		S2_3641630
		S1_18972566
		S6_48605735
		S3_15437544
		S3_69907059
		S2_38300258
		S2_30699096
		S6_58131453
		S9_55949421
		S5_11594192
		S3_58350085
		S1_15031504
		S1_18107593
		S6_6108702
		S2_61153330
		S4_66311101
		S6_54803939
		S3_73290369
		S1_73467708
		S1_12967579
		S1_56730875
		S8_3103690
		S1_79995731
		S2_9371852
		S4_9886176
		S5_6809841
		S8_3665028
		S1_11197486
		S4_20672525
		S1_73899447
		S4_7929343
		S1_52916599
		S7_9086324
		S5_1169513

Catogoery	Total	SNPs
		S5_2693307
		S2_58290727
		S7_501072
		S7_10063505
		S9_2775584
		S1_13426290
		S7_62935563
		S5_69847947
		S4_7468099
		S1_26919016
		S2_4257650
		S8_57933818
		S2_12509279
		S4_66306689
		S2_65985230
		S2_3808802
		S4_45492092
		S3_69630810
		S1_65255766
		S1_54790770
		S1_57470094
		S6_53592346
		S2_65121323
		S1_14876817
		S2_58682756
		S3_57237987
		S2_60149964
		S3_70344725
		S2_12796189
		S8_4491958
		S1_2584538
		S3_73239700
		S6_53857096
		S8_56542068
		S8_61603519
		S8_54372906
		S6_50656541
		S5_67017673
		S1_48494267
		S7_500850
		S3_73032285
		S3_69885966
		S4_66295932
		S4_3370586
		S6_58575352
		S3_58315092
		S3_56637055
		S3_71412129
		S6_50005203
		S8_53272015
		S3_6109944
		S5_20276479
		S2_57511822
		S1_52223238
		S2_7324214
		S6_60926389
		S3_20558073
		S3_72850075
		S2_3641641
		S8_60349211
		S9_215288
		S4_12948212
		S7_63896736
		S7_38972016
		S3_72979964

Catogoery	Total	SNPs
		S1_12305235
		S8_61392776
		S3_62858492
		S2_73710475
		S3_3757333
		S7_62396525
		S1_16553032
		S2_60967872
		S8_2523744
		S1_12968332
		S7_62131254
		S3_56681967
		S2_8050324
		S8_1325659
		S2_59637039
		S4_1628629
		S3_52897544
		S3_5130769
		S8_509667
		S7_271845
		S9_52612570
		S2_5389764
		S9_53238263
		S3_19516362
		S3_60922412
		S2_65383892
		S7_63155680
		S4_6256358
		S4_66125718
		S9_48996210
		S4_7334217
		S1_59444147
		S7_27573469
		S2_61856128
		S9_5374266
		S1_68318541
		S4_10307887
		S9_3194724
		S2_58774007
		S1_71740501
		S4_38925910
		S8_54852729
		S7_57892718
		S4_8754346
		S3_64197871
		S4_50272842
		S2_57607821
		S1_12304254
		S1_10408825
		S8_1691578
		S6_1454196
		S2_12590234
		S6_53638838
		S4_1337809
		S4_46113408
		S9_215198
		S3_20022324
		S6_47538964
		S1_11198829
		S4_58370688
		S5_3049786
		S5_7641850
		S4_33238326
		S1_66568095
		S6_47901262

Catogoery	Total	SNPs
		S3_2047929
		S2_73888922
		S2_6235080
		S8_61581034
		S6_1404596
		S2_10605230
		S1_57165032
		S9_47051407
		S4_760009
		S3_2082853
		S1_56863319
		S8_54754698
		S3_69692795
		S5_2088546
		S6_49469737
		S8_50019355
		S8_53840504
		S3_68982837
		S7_59393451
		S2_61444382
		S6_41596963
		S6_50726472
		S9_44553513
		S2_64788046
		S5_69416793
		S1_1558048
		S2_4899971
		S1_59383253
		S2_66175561
		S2_56290196
		S1_80074678
		S1_19715049
		S7_6849719
		S5_8995237
		S3_72123659
		S7_6412747
		S1_26784233
		S2_14723723
		S2_63350458
		S5_11895978
		S1_72859898
		S2_65335662
		S9_50598324
		S5_58574593
		S5_11792642
		S6_42017365
		S2_61739830
		S6_3228064
		S4_52963595
		S3_56255580
		S2_61444909
		S1_10384100
		S1_72373895
		S1_60720835
		S1_61639297
		S7_15851897
		S3_53583422
		S6_5624830
		S7_63082417
		S3_59793455
		S3_53231110
		S1_12197519
		S2_1017459
		S1_57102111
		S2_5417865

Catogoery	Total	SNPs
		S1_74305733
		S7_831823
		S2_60965619
		S1_3880241
		S1_1610968
		S6_20585811
		S7_54409717
		S2_12351397
		S8_60362728
		S8_40677039
		S3_56160357
		S5_9181159
		S1_56905886
		S5_65934680
		S3_65325871
		S8_46648779
		S5_55896025
		S1_10140805
		S6_49377054
		S1_79965944
		S6_1549133
		S6_2087439
		S2_4109682
		S9_58742697
		S5_13584743
		S9_1115508
		S8_54927891
		S3_57929033
		S3_51904168
		S9_56968839
		S4_33698483
		S3_52430630
		S2_65643615
		S1_66632262
		S6_50791904
		S2_9466699
		S7_574225
		S1_74378860
		S8_2653827
		S1_2272160
		S5_58476777
		S9_4787767
		S9_57784870
		S7_52292165
		S3_52007781
		S3_51617000
		S9_49551667
		S1_51841723
		S3_72027865
		S9_3702126
		S1_80620950
		S8_41636798
		S9_51062286
		S8_1755828
		S3_58846200
		S9_141098
		S4_62988583
		S5_1327131
		S3_70621963
		S8_61471123
		S4_13913516
		S4_53868849
		S6_6770339
		S2_8264981
		S1_7509877

Catogoery	Total	SNPs
		S8_61925255
		S3_56125216
		S3_69698529
		S2_3883280
		S1_6264399
		S6_30072245
		S2_57146339
		S5_6171822
		S4_8592410
		S4_10628604
		S5_66298412
		S1_22682559
		S7_62806304
		S1_74198327
		S1_78619689
		S3_54220562
		S1_6707475
		S3_71378454
		S9_52837450
		S1_67678850
		S8_3018172
		S2_3107262
		S1_76495673
		S9_1663469
		S7_62354783
		S5_7020635
		S1_61596211
		S1_71552503
		S6_48635168
		S2_8144361
		S7_58607356
		S5_67495287
		S9_1260371
		S6_60840670
		S5_11706218
		S8_49677488
		S1_9413261
		S4_5085168
		S2_75155377
		S1_72206060
		S2_62351034
		S3_68130435
		S2_17975844
		S6_2193049
		S1_72488253
		S2_55384044
		S2_73407568
		S1_68831768
		S9_52207282
		S6_31746859
		S7_62805677
		S4_19519314
		S1_2471096
		S7_39247498
		S6_50435878
		S4_5534735
		S7_62494218
		S8_48960942
		S2_76837436
		S7_1639802
		S1_9153558
		S4_51877975
		S5_68228795
		S1_75656622
		S2_66280873

Catogoery	Total	SNPs
		S4_61260545
		S8_51822725
		S3_69281927
		S2_8879158
		S2_8647674
		S3_57750634
		S2_71704730
		S1_59562698
		S6_47419703
		S1_74545585
		S5_16529244
		S3_61990923
		S9_52122011
		S2_64144909
		S2_61060332
		S6_20775034
		S8_4204667
		S5_10561908
		S9_4531225
		S2_64287871
		S2_6092464
		S4_58382803
		S1_66791760
		S1_45869664
		S2_32127479
		S2_57862866
		S7_62862965
		S3_56681275
		S2_6396284
		S7_9683253
		S5_36651833
		S1_31148486
		S2_66010391
		S4_19667023
		S4_7273385
		S3_51997038
		S1_15854579
		S8_51921820
		S4_6006458
		S3_68664167
		S3_65633885
		S6_45160543
		S1_80019380
		S7_19015542
		S5_58383631
		#N/A
		S4_51951343
		S6_56806429
		S2_62139114
		S1_75861426
		S1_77768512
		S2_3668993
		S2_75343606
		S2_77014527
		S1_79943156
		S8_55930253
		S7_59108949
		S7_60487860
		S8_3369918
		S2_64495140
		S1_73467733
		S2_68954927
		S7_7590365
		S4_421485
		S5_63406179

Catogoery	Total	SNPs
		S8_60087256
		S2_6687221
		S2_2781006
		S2_5559853
		S8_61641330
		S2_69715795
		S3_72310961
		S1_11197129
		S3_53099808
		S1_13401161
		S2_67765555
		S2_75684336
		S2_3804890
		S8_61563154
		S4_5002524
		S1_80698628
		S7_58476579
		S1_69941660
		S2_6777530
		S2_65927019
		S4_45398405
		S5_63115845
		S5_54661078
		S2_12754265
		S1_65291607
		S3_5458052
		S1_46947912
		S5_1609630
		S6_50656463
		S1_16543092
		S9_10183696
		S1_64803254
		S5_68080102
		S5_3181354
		S3_60583148
		S4_23692086
		S4_38418049
		S7_2410791
		S8_3476907
		S2_42945125
		S6_4445113
		S4_15153065
		S6_45640372
		S7_54683228
		S1_16641343
		S6_50309411
		S9_51683414
		S2_68005959
		S2_65823100
		S1_60778163
		S3_55403546
		S6_48290431
		S6_8878438
		S1_58204300
		S2_69142747
		S1_27291973
		S2_63933908
		S1_65245223
		S8_55830413
		S8_60349179
		S1_79497968
		S4_4160530
		S4_5894393
		S7_9515054
		S1_74360070

Catogoery	Total	SNPs
		S2_53892623
		S2_73366029
		S3_51322636
		S1_63346901
		S6_1587007
		S2_65784140
		S1_21803414
		S8_5010701
		S5_61217883
		S2_6410123
		S9_40978332
		S2_64334070
		S1_2469678
		S2_76892564
		S4_12419362
		S6_42463477
		S1_53202075
		S3_73925062
		S4_19935198
		S1_56319843
		S1_53493941
		S7_54854114
		S2_58887619
		S2_77040502
		S1_68301256
		S3_68226242
		S4_50273627
		S4_3814370
		S7_64067686
		S5_2693154
		S6_47652162
		S9_4238664
		S3_48311988
		S1_19164973
		S5_69880109
		S8_3491753
		S3_45640097
		S7_12071667
		S4_57641142
		S1_65739450
		S7_6451557
		S2_77014529
		S6_6448051
		S7_41050487
		S7_54525594
		S3_5910192
		S4_66199040
		S1_9814582
		S3_70623255
		S4_5338202
		S1_8981788
		S1_20207309
		S4_6756835
		S2_18958476
		S2_3808776
		S4_2151872
		S6_55690118
		S6_58859929
		S4_788851
		S6_51222126
		S9_8183168
		S4_40883606
		S2_10329071
		S8_5323004
		S1_62068188

Catogoery	Total	SNPs
		S2_27305851
		S6_49469703
		S4_4905027
		S6_3200962
		S4_46500762
		S3_15482115
		S7_17140473
		S2_64342419
		S6_38162031
		S4_4101099
		S3_70247205
		S7_63156223
		S3_69365954
		S3_69734235
		S2_57265158
		S7_62309669
		S9_3351145
		S3_69907155
		S5_69822726
		S8_54711058
		S9_41579399
		S7_52290739
		S1_3129037
		S6_42013192
		S9_3356508
		S6_52602801
		S5_1966409
		S3_65425743
		S7_8879698
		S1_51003646
		S2_148211
		S2_6020544
		S7_1228633
		S1_59520765
		S1_2718975
		S1_65150396
		S5_8752438
		S6_51444760
		S6_52008526
		S8_2543424
		S2_73941891
		S1_54120865
		S2_72984930
		S9_4249062
		S1_57208278
		S2_61091696
		S1_77726708
		S3_68919617
		S8_50831088
		S4_1008264
		S1_59338088
		S1_6922963
		S1_75619552
		S7_59034813
		S8_5459211
		S6_47071575
		S2_5718968
		S6_51925840
		S8_54443128
		S8_3510734
		S2_426296
		S1_76025214
		S6_13863920
		S1_59338088
		S3_62407405

Catogoery	Total	SNPs
		S1_11341545
		S4_24560536
		S1_26890759
		S4_52219851
		S5_69851828
		S1_16203470
		S8_3194215
		S6_53285177
		S2_64495138
		S1_79194040
		S6_45540295
		S9_53976810
		S7_62806268
		S7_38852478
		S4_1114894
		S8_11323084
		S8_1840299
		S3_5138621
		S2_55048559
		S1_67610048
		S8_42502436
		S7_60259959
		S1_45644473
		S4_44730236
		S8_55617456
		S8_1438147
		S2_68005900
		S1_53974674
		S1_15031642
		S2_54406632
		S6_26232684
		S2_8111650
		S3_69630946
		S1_11609239
		S1_79237043
		S2_67383620
		S2_2235766
		S5_62365032
		S3_73216547
		S3_5645004
		S1_21849053
		S7_54698258
		S1_24001945
		S1_66813884
		S5_3181265
		S9_2934155
		S2_72612789
		S9_47049870
		S2_52687193
		S3_6265398
		S8_12577083
		S2_68826929
		S2_3878658
		S6_52322439
		S2_16428875
		S9_2327729
		S4_50974937
		S2_4581138
		S6_47626934
		S1_68321716
		S6_1668347
		S3_70531686
		S7_55751099
		S3_4796612
		S4_66147731

Catogoery	Total	SNPs
		S2_57174253
		S6_4442513
		S1_66385266
		S5_4915818
		S1_76291128
		S1_30509277
		S8_56144778
		S7_65079939
		S2_54462752
		S8_48981817
		S2_18128420
		S3_16334414
		S3_56295016
		S5_66648034
		S7_57153244
		S2_73339407
		S5_61208123
		S6_49441701
		S1_45971188
		S6_50583878
		S6_53881269
		S2_69841555
		S4_3862205
		S6_40369810
		S2_5489028
		S9_1594466
		S9_54606938
		S1_11472022
		S2_14723733
		S2_65031797
		S3_64840354
		S2_68923590
		S4_56596124
		S1_11127575
		S8_49317971
		S8_60933906
		S6_45570895
		S1_71939527
		S2_6271132
		S2_46699698
		S2_16386795
		S4_54102693
		S1_14045536
		S2_61029335
		S3_73887813
		S4_33650266
		S4_53791375
		S8_49663779
		S3_73041210
		S1_73630367
		S2_59240066
		S3_53516781
		S1_64993143
		S8_2543525
		S1_17443807
		S6_1325917
		S7_62396520
		S1_6916528
		S9_44572781
		S1_2617002
		S3_53406090
		S8_53982171
		S4_12310860
		S9_233795
		S7_59397308

Catogoery	Total	SNPs
		S9_2941941
		S1_57719236
		S9_231437
		S1_2719289
		S8_56112430
		S4_2762236
		S4_8902810
		S3_15835622
		S9_133865
		S9_57172609
		S4_6937226
		S1_10317816
		S7_9515067
		S7_61575919
		S8_60549301
		S2_1124540
		S3_71378059
		S3_59580190
		S3_55801623
		S2_74867452
		S1_66790757
		S4_9378890
		S4_54042987
		S6_25597790
		S1_66791929
		S2_62925059
		S1_11670273
		S5_61161427
		S2_3405847
		S4_54119446
		S2_10525883
		S4_67124107
		S4_54204330
		S9_3712868
		S5_61061266
		S7_59390700
		S4_7889631
		S1_52333065
		S8_49156092
		S4_54168292
		S4_6773693
		S5_10689984
		S7_58197967
		S3_5626232
		S5_36036411
		S6_53021321
		S2_61859441
		S2_12687920
		S2_59238559
		S7_60259943
		S1_72346592
		S2_6232488
		S3_1964492
		S7_63553898
		S2_2888142
		S4_12409929
		S2_7728452
		S9_54094900
		S1_20249734
		S1_77319295
		S1_61690539
		S2_69699397
		S2_59249465
		S8_61921671
		S6_49842109

Catogoery	Total	SNPs
		S4_50356860
		S1_49924064
		S8_37271043
		S6_47281814
		S1_17240682
		S2_17761094
		S5_65909600
		S2_9809711
		S2_2757424
		S8_53524630
		S4_11121847
		S2_57774725
		S1_77955074
		S5_58548619
		S3_74112316
		S4_33268537
		S2_72330856
		S5_12791199
		S1_61976820
		S9_10342681
		S1_50829743
		S4_52949049
		S1_3350852
		S1_7412098
		S2_62729836
		S1_49506809
		S9_11486145
		S3_14076800
		S4_42348263
		S4_9492723
		S9_51890207
		S5_1836125
		S9_7538589
		S6_2016236
		S2_52684834
		S2_1482416
		S6_45715649
		S8_54938227
		S3_73796746
		S2_67640674
		S6_45540273
		S2_17486805
		S8_5156073
		S1_79664740
		S8_61651495
		S9_10515717
		S3_52805301
		S4_68098061
		S3_68919642
		S6_60908523
		S1_66650934
		S2_47772288
		S5_11465084
		S2_69327058
		S6_51114433
		S2_60325751
		S2_59538572
		S1_18563113
		S4_61260537
		S1_13095933
		S5_58530078
		S8_41591125
		S7_62354782
		S4_21050153
		S1_11198830

Catogoery	Total	SNPs
		S1_10165582
		S6_47800577
		S3_71678101
		S1_79742671
		S1_80516739
		S2_66092476
		S6_53013024
		S9_2941947
		S2_16197933
		S6_1400240
		S9_6652170
		S1_73770236
		S8_60695655
		S3_53472788
		S2_66931853
		S1_7617414
		S9_2781769
		S9_5464988
		S1_46058280
		S1_55237118
		S1_26887718
		S8_54927635
		S2_60595348
		S2_67383434
		S2_12744933
		S3_70245320
		S3_57777058
		S4_63522423
		S4_67082649
		S6_54462516
		S1_12247836
		S9_41549884
		S8_11441485
		S8_1964090
		S1_960
		S1_53480551
		S1_47839042
		S6_47334104
		S4_7539064
		S2_63692227
		S1_67487322
		S5_58464432
		S6_52777830
		S1_8623566
		S9_53925541
		S4_42467713
		S1_65451869
		S1_56695759
		S6_3327783
		S9_50451624
		S2_75153917
		S3_5563108
		S5_69822710
		S1_79363869
		S2_62726297
		S5_67526718
		S8_4409253
		S6_51419252
		S2_68021425
		S1_58919229
		S8_61921535
		S5_15767957
		S1_7784945
		S6_51865306
		S7_60488051

Catogoery	Total	SNPs
		S1_2084561
		S1_9665480
		S2_64334142
		S9_47050005
		S6_1668317
		S8_1217630
		S1_3564598
		S1_76635128
		S2_67990166
		S4_55505698
		S1_52255945
		S2_6045215
		S2_63520617
		S1_11550661
		S9_44414316
		S3_59368480
		S1_49363686
		S7_42665535
		S8_43756154
		S8_61541278
		S4_9749759
		S4_50569794
		S2_65407920
		S4_62905809
		S3_69395371
		S1_6512012
		S7_56660947
		S2_8713194
		S2_75055835
		S3_70242187
		S1_58516035
		S1_75758901
		S1_67833574
		S1_22064317
		S5_68736161
		S8_1250267
		S6_46327583
		S3_51906747
		S2_64423336
		S1_78711457
		S8_386636
		S4_5948026
		S3_73506876
		S1_76426417
		S3_52047978
		S2_2323576
		S8_1664237
		S2_75456079
		S8_55521891
		S2_77540788
		S6_52532715
		S6_60774035
		S7_60464193
		S6_25370204
		S4_67981546
		S8_12577082
		S2_8905291
		S5_66506941
		S7_7284142
		S8_53754850
		S1_1615847
		S3_70276037
		S4_58313633
		S2_4726271
		S9_52796178

Catogoery	Total	SNPs
		S1_57110292
		S2_72643068
		S8_1445369
		S9_4037876
		S1_17585586
		S1_21722447
		S1_71946541
		S5_6051657
		S1_21722222
		S5_11615594
		S2_12326071
		S9_10597793
		S1_7825162
		S6_46330277
		S1_52770144
		S2_14570058
		S2_63384767
		S2_792974
		S1_71640608
		S2_58682748
		S2_10976514
		S7_8905175
		S2_62739814
		S4_11072583
		S4_2086806
		S3_47964987
		S4_13818618
		S5_61217899
		S3_51971849
		S6_29799844
		S8_49231972
		S2_64072282
		S1_66401228
		S5_69707345
		S1_11107623
		S6_49937723
		S2_12509171
		S6_54302476
		S1_77745742
		S4_54127043
		S2_67873637
		S4_52420789
		S9_52207273
		S6_47071572
		S7_65058059
		S2_10926664
		S2_63447402
		S3_58983687
		S1_4121230
		S9_2892001
		S6_844947
		S8_56542072
		S2_5705846
		S2_8335682
		S4_3415428
		S8_57974123
		S2_10976476
		S3_59337963
		S3_59653421
		S2_12434763
		S2_61068197
		S5_14317535
		S2_26086649
		S8_60343439
		S2_60608445

Catogoery	Total	SNPs
		S7_40355519
		S7_63639764
		S1_77832704
		S2_60986382
		S8_51183051
		S3_74189833
		S2_10269497
		S9_50009011
		S2_58237159
		S9_51545815
		S6_50343494
		S6_3543624
		S6_47743052
		S2_57591447
		S6_32048479
		S4_4855505
		S5_63471138
		S8_412196
		S9_51890218
		S4_53265404
		S5_13606286
		S6_1067428
		S2_75343557
		S3_73704913
		S9_43747362
		S1_66554503
		S8_61598107
		S9_1715377
		S3_71926817
		S8_60259355
		S3_55208515
		S2_8672301
		S8_51822454
		S3_63397859
		S1_66813882
		S8_4542276
		S1_7822999
		S4_58178148
		S5_1740605
		S3_70239282
		S1_16440035
		S7_16083870
		S4_66233677
		S4_7340732
		S6_41588906
		S2_12357528
		S1_49924249
		S4_67767151
		S7_57239996
		S2_7541609
		S6_57409372
		S9_52612567
		S2_58996775
		S1_8730054
		S1_59131478
		S9_960486
		S9_52642714
		S2_67564229
		S6_6448048
		S9_53172723
		S3_63324258
		S5_5114468
		S2_62729828
		S7_60488159
		S1_30330390

Catogoery	Total	SNPs
		S4_32979112
		S4_3714646
		S1_60666857
		S2_63174777
		S1_7279183
		S1_6634728
		S8_2408903
		S3_52014069
		S8_2082251
		S1_63951051
		S2_6751723
		S4_52241249
		S6_47609611
		S2_41143803
		S1_46947963
		S2_6741894
		S5_2074868
		S3_58281901
		S2_16421903
		S6_41274367
		S1_73855378
		S8_58744271
		S5_59317005
		S1_49924073
		S6_50704068
		S2_56332760
		S6_27279775
		S2_7624976
		S2_1587784
		S9_57629290
		S6_50364575
		S3_72125680
		S8_44334959
		S5_62396386
		S4_22815586
		S7_815922
		S1_17028957
		S9_58701490
		S1_7619687
		S5_1326748
		S4_9492748
		S8_49677481
		S1_77100610
		S8_56714892
		S1_67713212
		S6_51865058
		S6_13460856
		S2_60569440
		S2_66599825
		S1_14055723
		S1_59009818
		S7_63117143
		S7_62611667
		S2_58996672
		S4_43486097
		S9_47028337
		S9_56031807
		S1_79937197
		S3_5130766
		S7_5171859
		S3_48311973
		S1_9824814
		S1_66571295
		S9_56538258
		S3_73214858

Catogoery	Total	SNPs
		S4_12181342
		S2_62051320
		S4_5065211
		S1_62724398
		S5_18541786
		S9_6791211
		S4_12085423
		S3_5787681
		S1_57183495
		S1_6543990
		S2_39983779
		S4_6504873
		S3_51899452
		S3_13439076
		S1_18894104
		S6_45570838
		S3_55142236
		S6_48097597
		S1_59383279
		S2_6045432
		S1_30656561
		S7_52893272
		S1_18659611
		S6_51320771
		S5_63235237
		S3_55300423
		S2_57607817
		S6_56573974
		S2_11784868
		S4_6170737
		S4_5880769
		S2_74032451
		S3_4577762
		S6_49519554
		S1_76491690
		S5_13393874
		S1_54854707
		S3_59542656
		S1_59471526
		S7_1526684
		S7_8869943
		S8_53877300
		S2_58410744
		S3_44538953
		S6_53622978
		S2_66428963
		S4_44730217
		S3_46230006
		S3_70256166
		#N/A
		S1_20371836
		S2_75152903
		S6_38194563
		S3_3757347
		S1_69962275
		S7_56573360
		S1_60912500
		S1_64156473
		S1_52230850
		S1_2717909
		S1_24765202
		S2_67839040
		S4_68098068
		S1_18956450
		S6_53926676

Catogoery	Total	SNPs
		S3_60581629
		S1_61559393
		S6_52635446
		S3_1964436
		S1_13977290
		S4_51951349
		S3_71698458
		S6_52123049
		S9_29772
		S2_73103089
		S1_58661417
		S7_889829
		S2_69151417
		S6_49473228
		S8_45517601
		S4_2891356
		S7_63289533
		S7_5538178
		S7_19536757
		S7_58298370
		S2_61153594
		S2_1482429
		S5_12426868
		S9_54147364
		S5_62378796
		S2_10286020
		S6_3533941
		S2_6864352
		S9_51666241
		S3_811886
		S9_55056612
		S1_26312896
		S9_6694503
		S1_66261086
		S2_66179390
		S2_66068667
		S3_16534458
		S8_61612370
		S2_1462262
		S5_67297112
		S2_75688219
		S2_8264056
		S2_72329360
		S1_14490575
		S8_61615380
		S9_55862987
		S2_61331927
		S6_2315241
		S6_50704102
		S1_63004827
		S1_71823042
		S6_54216701
		S6_1047867
		S9_51890391
		S3_54220547
		S1_10408826
		S3_5912684
		S1_67679850
		S3_4577752
		S2_6423690
		S4_7190534
		S1_79150108
		S4_49016699
		S2_68868784
		S1_333918

Catogoery	Total	SNPs
		S7_60709310
		S1_2909515
		S4_63395491
		S5_65525009
		S9_960483
		S3_5494790
		S5_65636818
		S8_44421982
		S6_53028154
		S7_8994939
		S4_12089828
		S2_747497
		S5_61888632
		S1_14437145
		S8_58464093
		S1_15294116
		S5_8792407
		S6_1112995
		S1_55028385
		S8_51047640
		S2_6139989
		S1_59360154
		S6_53953449
		S3_51961245
		S4_66243116
		S7_5742635
		S2_2752791
		S5_6760145
		S4_51492934
		S6_53023293
		S3_48963079
		S4_19921059
		S1_10043532
		S7_887735
		S1_14437143
		S3_70825086
		S4_24706946
		S6_3327800
		S7_54092688
		S5_62355332
		S3_55005178
		S7_437596
		S7_53655578
		S2_76475949
		S9_59378892
		S5_5791644
		S4_7334061
		S1_61730047
		S2_72315446
		S5_1117822
		S7_62309625
		S5_61188832
		S1_8502986
		S1_61690550
		S7_12580350
		S4_7474886
		S1_66304424
		S6_45647662
		S2_11944518
		S6_53583029
		S7_60709193
		S7_64861050
		S9_49054130
		S2_3602204
		S8_43885947

Catogoery	Total	SNPs
		S3_16168485
		S2_49695837
		S5_63408417
		S2_66173362
		S8_55010534
		S9_47028104
		S4_12350787
		S8_55700365
		S1_63711697
		S4_37131455
		S8_1438074
		S1_910340
		S1_73324075
		S1_19187868
		S7_58534243
		S3_60583195
		S1_80001209
		S2_2908971
		S2_25439170
		S7_51315479
		S3_13633038
		S9_58179013
		S2_3979059
		S5_5366266
		S8_4310379
		S8_57577210
		S6_37970618
		S8_29313203
		S8_58455817
		S2_17607226
		S4_47808763
		S7_39756853
		S3_61711103
		S2_59576785
		S3_59723583
		S3_61529263
		S3_59558281
		S1_4121029
		S2_17604003
		S2_74633746
		S1_79961941
		S4_45589496
		S1_25481585
		S9_50741996
		S1_22055486
		S7_2337911
		S1_60758110
		S1_68781639
		S9_2825198
		S2_5800302
		S4_5894349
		S8_60633458
		S6_14955515
		S3_72514850
		S7_41773316
		S5_9847071
		S6_58881505
		S1_12160126
		S1_79793862
		S3_60996629
		S5_3134146
		S9_58179023
		S2_69858485
		S7_60654448
		S3_65364577

Catogoery	Total	SNPs
		S9_8407140
		S9_51545855
		S3_62273239
		S7_58482206
		S1_59562729
		S4_36127776
		S5_8696593
		S3_69578569
		S3_57892980
		S8_61256663
		S7_52300499
		S3_60800068
		S3_56085485
		S6_50919262
		S2_66402258
		S2_17758048
		S5_5838434
		S2_68021590
		S5_1326987
		S5_2966559
		S5_10658811
		S1_20207351
		S8_58132073
		S2_10398341
		S1_56568041
		S9_214859
		S4_7273390
		S4_2071213
		S1_61875711
		S1_14515341
		S1_7557049
		S8_51188299
		S2_66008964
		S1_7325638
		S3_68840444
		S9_51890197
		S4_35249172
		S4_2634973
		S4_7875466
		S1_20588671
		S3_69696812
		S2_74969935
		S5_40969699
		S4_2626997
		S7_8820124
		S3_59601690
		S1_16543097
		S2_67807415
		S4_34910951
		S6_53606859
		S1_62732121
		#N/A
		S8_51931123
		S2_7541379
		S5_8191437
		S4_20672540
		S2_57444433
		S1_72143340
		S1_7438347
		S2_53430284
		S1_25606798
		S4_789275
		S3_73160937
		S2_61525426
		S2_8089789

Catogoery	Total	SNPs
		S3_60614839
		S5_12719280
		S7_63695458
		S3_51906283
		S2_68561616
		S4_450155
		S8_49317852
		S1_51003652
		S5_9537224
		S5_69794598
		S2_66092842
		S8_49573273
		S3_4956348
		S7_2771640
		S4_3740629
		S5_2829033
		S1_61737886
		S3_58901020
		S4_68013347
		S2_50018483
		S7_64098865
		S7_54936127
		S4_48703811
		S1_22272060
		S1_49398330
		S5_11931189
		S1_12967574
		S6_58739489
		S2_59738103
		S1_57204835
		S6_5825412
		S2_58604311
		S3_57223923
		S1_66113545
		S2_12744929
		S1_7313243
		S1_60913873
		S8_59643425
		S3_57312478
		S3_70558683
		S2_4770026
		S3_20255100
		S4_66311108
		S8_59140955
		S6_32251766
		S6_48650753
		S2_73366262
		S3_73860575
		S8_2050975
		S2_7112091
		S2_64467121
		S3_37771987
		S2_69604838
		S5_1052272
		S9_42452595
		S4_47808749
		S7_2041040
		S6_42351178
		S3_55062395
		S6_51404279
		S1_53165738
		S8_56144773
		S5_2693011
		S5_62499848
		S2_12744922

Catogoery	Total	SNPs
		S9_58471730
		S5_2693011
		S8_54939807
		S2_63179735
		S1_11205235
		S2_39666062
		S4_7961336
		S3_15945865
		S1_7390211
		S2_75672247
		S9_55004996
		S2_16410491
		S7_3694745
		S7_492238
		S3_68380196
		S9_617374
		S3_70199859
		S6_49293469
		S4_62909168
		S1_26240615
		S2_49670786
		S3_47609412
		S3_56719299
		S3_59368484
		S2_65921300
		S8_1753900
		S2_60678952
		S2_72263934
		S3_58269747
		S8_49843769
		S9_57657554
		S4_7169881
		S4_63399375
		S1_6320905
		S2_37492567
		S7_8905183
		S4_5583349
		S7_2791371
		S7_62135105
		S5_15774692
		S3_6460983
		S3_69679841
		S8_11441489
		S5_63240149
		S7_9445308
		S4_56672401
		S2_75679392
		S9_2935583
		S9_5189116
		S8_3193974
		S7_58658372
		S9_59017346
		S2_70918428
		S2_74869400
		S1_57118658
		S1_67369763
		S8_57721063
		S1_912098
		S1_18188882
		S7_65177282
		S9_57833927
		#N/A
		S4_68013506
		S7_56556498
		S3_67979331

Catogoery	Total	SNPs
		S1_8998940
		S4_7468081
		S1_79507184
		S3_70621955
		S2_8258220
		S2_6204309
		S2_16390734
		S9_5175947
		S1_64832858
		S2_75304464
		S2_59945456
		S4_58313633
		S3_3910437
		S7_56233120
		S7_39944504
		S8_3492501
		S8_44848319
		S2_13459043
		S3_69698464
		S1_10949868
		S7_60216273
		S9_57122032
		S1_11205159
		S2_68991226
		S2_7661217
		S7_64370508
		S8_56642722
		S1_28394437
		S1_16387880
		S2_58007153
		S3_63678519
		S1_5806908
		S6_48554934
		S4_67083988
		S9_6695478
		S4_51193942
		S2_4192739
		S4_54095475
		S8_4310180
		S8_5442011
		S5_6101734
		S1_21699549
		S6_31693721
		S3_65461421
		S2_43991234
		S3_51704463
		S1_6647890
		S1_4120802
		S6_51803537
		S5_15774718
		S1_72743597
		S3_16373402
		S1_2470473
		S2_10527309
		S3_71421253
		S7_17425477
		S1_11003300
		S6_53861164
		S4_67774409
		S6_8865333
		S6_34041179
		S2_12690898
		S2_65727831
		S2_59751634
		S8_61955788

Catogoery	Total	SNPs
		S7_59222359
		S8_3219256
		S1_51494601
		S9_51461440
		S2_75892353
		S2_59706876
		S5_11615623
		S8_49663783
		S1_72477148
		S6_40434484
		S3_620581
		S9_214870
		S2_1583803
		S2_8884920
		S6_50876030
		S4_39239611
		S1_7277851
		S3_69264647
		S6_4444721
		S7_56589400
		S3_71232951
		S4_5107457
		S2_60712345
		S6_53169697
		S5_11064448
		S2_68917365
		S6_14154677
		S4_3797212
		S1_72171167
		S9_51033824
		S1_71360478
		S6_45197534
		S8_5157398
		S1_10688128
		S5_61178507
		S6_35904793
		S1_73662893
		S4_8591990
		S2_37984046
		S2_17910951
		S7_15560272
		S1_6512007
		S6_7201687
		S7_31841327
		S6_51972688
		S3_6329021
		S3_3757329
		S1_9822648
		S1_26599117
		S4_66114186
		S5_62378662
		S1_79591370
		S2_60688144
		S9_44572785
		S1_57111251
		S3_1871543
		S9_5829138
		S2_12226544
		S2_4173200
		S5_15465367
		S4_3370558
		S8_60686472
		S3_64874933
		S4_51451388
		S1_61008560

Catogoery	Total	SNPs
		S2_3932828
		S2_62312363
		S3_5563246
		S2_75808113
		S4_56200682
		S3_68981481
		S6_46381581
		S4_12244051
		S3_69074842
		S2_60448542
		S5_62950664
		S9_141001
		S2_8114708
		S3_66788731
		S1_22260275
		S7_55981265
		S2_63584040
		S6_52566346
		S7_6679582
		S2_2019952
		S1_4086618
		S1_12969785
		S5_1835244
		S7_63982659
		S5_65910307
		S9_52032956
		S6_47538930
		S1_52089018
		S3_13445826
		S8_2574518
		S4_2363627
		S6_52621661
		S8_56184625
		S5_66665
		S5_12294710
		S7_16529366
		S4_2234093
		S4_40959015
		S7_538485
		S1_70042898
		S4_7210468
		S9_5861342
		S9_41589311
		S3_14076815
		S1_78565888
		S3_59503660
		S3_70599250
		S2_2758853
		S2_66280876
		S6_1067499
		S6_26230378
		S1_63004841
		S2_72265433
		S1_1841296
		S8_48982694
		S6_1316318
		S2_66044603
		S1_79546076
		S5_20867288
		S8_3380057
		S1_8441887
		S9_6375917
		S1_6336325
		S5_61209476
		S2_52993967

Catogoery	Total	SNPs
		S8_1885298
		S2_58025889
		S8_54938468
		S6_38113611
		S4_52370214
		S4_11078662
		S3_3060535
		S1_11472026
		S6_50914866
		S7_57643247
		S2_64382561
		S5_38107234
		S6_48697422
		S9_3620810
		S4_48061021
		S7_38847247
		S4_38175943
		S8_282505
		S2_7888589
		S4_66127415
		S3_55054917
		S6_58780630
		S2_136129
		S6_6721493
		S2_67562680
		S6_35667175
		S9_48996207
		S4_13858548
		S4_39588867
		S1_14754465
		S4_50273507
		S7_52323595
		S8_49319158
		S1_10593918
		S8_1234431
		S3_67287116
		S2_62315745
		S8_18771955
		S2_11880714
		S1_10040698
		S2_6609171
		S3_56466358
		S4_57520677
		S9_18287228
		S3_58381846
		S7_59354280
		S1_80341688
		S2_67991615
		S8_1991537
		S8_5062138
		S8_1397399
		S9_50250186
		S2_5375854
		S8_2374758
		S4_421367
		S5_18485235
		S3_59472358
		S5_10354436
		S1_60837330
		S1_57114982
		S7_59404214
		S2_6405475
		S2_62196277
		S6_51902532
		S1_72721957

Catogoery	Total	SNPs
		S1_49222049
		S8_38468157
		S9_4690233
		S9_51705469
		S1_9658746
		S6_51414932
		S9_52932644
		S1_77184163
		S6_50647081
		S1_55470038
		S6_19148640
		S6_53664190
		S8_56707291
		S3_5659708
		S2_13888238
		S9_1621819
		S1_55566871
		S6_52401555
		S5_63764878
		S1_60725448
		S2_61047590
		S4_7169832
		S5_11056410
		S7_58483106
		S3_73887808
		S5_65640662
		S2_76860062
		S9_8190256
		S1_16963246
		S2_16391633
		S7_2923789
		S4_44730220
		S8_9503549
		S3_60767448
		S2_6220144
		S3_57367779
		S5_1032849
		S4_4189610
		S1_56066521
		S5_61188850
		S8_3491503
		S2_60448683
		S7_6761223
		S2_63081247
		S2_61742737
		S6_44848564
		S9_59361523
		S6_35940543
		S4_54020041
		S5_69854906
		S6_50914827
		S5_41687237
		S5_472262
		S8_4409239
		S6_53622983
		S1_74107224
		S1_8973947
		S3_62027599
		S4_46475399
		S6_41172809
		S3_72849756
		S2_63474717
		S1_24465846
		S3_3289226
		S1_58165033

Catogoery	Total	SNPs
		S2_61083422
		S2_63520541
		S2_55477871
		S1_64633173
		S1_59700373
		S9_10386388
		S6_18140742
		S3_57050123
		S5_2274371
		S5_1761379
		S2_73584592
		S6_3533824
		S7_56464910
		S7_59298818
		S3_56674629
		S9_57463539
		S9_58659593
		S1_55709382
		S8_61603522
		S4_1114898
		S3_56085491
		S9_74418
		S5_5366264
		S1_62068102
		S1_66524970
		S4_14843506
		S1_71640640
		S1_65251039
		S7_2771642
		S6_13863814
		S8_60010369
		S3_4009001
		S3_57959196
		S7_9515074
		S4_6465627
		S6_2665924
		S6_4442724
		S4_7390356
		S9_1031041
		S1_8502993
		S6_55746356
		S9_54561026
		S1_61733342
		S9_9099732
		S6_51114635
		S6_38194473
		S3_71232302
		S2_39452676
		S5_7341495
		S2_59538538
		S1_63860019
		S4_52778242
		S2_18810757
		S1_386127
		S1_67679844
		S6_47043895
		S6_42013182
		S4_19685217
		S4_52350711
		S4_56610952
		S1_74524432
		S8_51316743
		S6_72131
		S6_56537412
		S1_15854586

Catogoery	Total	SNPs
		S9_2744777
		S2_61561743
		S5_69067575
		S3_4484788
		S2_64497549
		S2_25316480
		S4_37922295
		S5_40774495
		S2_69112159
		S3_58080846
		S1_52726167

Table S7 QTL mapping and MTA analysis identified common candidate genes from both studies

Names	Total	Gene Ids
**MTAs & QTL**	12	Sobic.010G205900
		Sobic.010G241100
		Sobic.010G189200
		Sobic.010G251800
		Sobic.010G245800
		Sobic.010G201100
		Sobic.010G254200
		Sobic.010G267600
		Sobic.010G254100
		Sobic.010G205600
		Sobic.010G264400
		Sobic.010G276600
**MTAs**	21	Sobic.010G222600
		Sobic.010G158400
		Sobic.010G212100
		Sobic.010G231800
		Sobic.010G241200
		Sobic.010G226400
		Sobic.010G206200
		Sobic.010G231900
		Sobic.010G191800
		Sobic.010G216200
		Sobic.010G174100
		Sobic.010G206300
		Sobic.010G191700
		Sobic.010G167500
		Sobic.010G173400
		Sobic.010G172700
		Sobic.010G231100
		Sobic.010G196500
		Sobic.010G172000
		Sobic.010G273300
		Sobic.010G163600
**QTL**	52	Sobic.010G271500
		Sobic.010G261200
		Sobic.010G273700
		Sobic.010G231700
		Sobic.010G167900
		Sobic.010G189300
		Sobic.010G271800
		Sobic.010G265500
		Sobic.010G266600
		Sobic.010G208600
		Sobic.010G235500
		Sobic.010G196900
		Sobic.010G270300

Names	Total	Gene Ids
		Sobic.010G261900
		Sobic.010G267300
		Sobic.010G279100
		Sobic.010G191100
		Sobic.010G268400
		Sobic.010G260200
		Sobic.010G272800
		Sobic.010G260300
		Sobic.010G254800
		Sobic.010G215700
		Sobic.010G273800
		Sobic.010G250400
		Sobic.010G192400
		Sobic.010G271700
		Sobic.010G256700
		Sobic.010G271400
		Sobic.010G254700
		Sobic.010G255700
		Sobic.010G261800
		Sobic.010G249600
		Sobic.010G258600
		Sobic.010G195600
		Sobic.010G234000
		Sobic.010G189600
		Sobic.010G202700
		Sobic.010G235800
		Sobic.010G249800
		Sobic.010G234400
		Sobic.010G259200
		Sobic.010G198000
		Sobic.010G205800
		Sobic.010G249500
		Sobic.010G267700
		Sobic.010G272700
		Sobic.010G265600
		Sobic.010G202100
		Sobic.010G246400
		Sobic.010G272600
		Sobic.010G192100

Table S8:Linkage map with marker distances and the segregation distortion of 262 SNP-SSR markers on 152 F_2 recombinant progeny and their chi square values and significance

			Alleles %				
S.No.	Locus 3.1v	Position (cM)	RSG04008-6	Heterozygote	J2614-11	χ^2	Signif.
1	S10_48868738	0	19	24	75	94.7	*******
2	S10_45951367	0.575	31	19	81	104.2	*******
3	S10_48402220	1.374	28	17	59	65.6	*******
4	S10_46043449	2.047	32	14	46	48.8	*******
5	S10_50029310	2.691	24	7	37	47.9	*******
6	S10_48930598	4.118	40	30	41	23.4	*******
7	S10_48930598	4.815	40	30	39	22.1	*******
8	S10_48237824	5.77	23	6	43	61.1	*******
9	S10_46110057	6.247	27	8	59	86.5	*******
10	S10_48237937	7.457	32	18	76	95	*******
11	S10_51082196	8.036	15	5	52	91.4	*******
12	S10_48226499	8.206	22	23	61	62.7	*******
13	S10_51971962	9.373	24	13	57	72.4	*******
14	S10_48237953	10.331	32	24	69	69.3	*******
15	S10_48237948	10.366	32	23	70	73	*******
16	S10_49332738	11.638	27	19	72	88.6	*******
17	S10_51339000	12.358	31	29	73	68.8	*******
18	S10_49342560	12.877	26	22	61	61.2	*******
19	S10_51082222	13.419	17	13	79	133.7	*******
20	S10_45354184	13.877	17	5	47	76.5	*******
21	S10_46680940	14.172	18	6	50	79.6	*******
22	S10_45814238	14.434	21	8	48	67.3	*******
23	S10_52214688	15.06	15	18	75	114.7	*******
24	S10_48215508	15.53	20	40	86	89.5	*******
25	S10_47253248	16.084	23	22	77	97.7	*******
26	S10_51082261	16.653	10	2	58	128.1	*******
27	S10_48215488	16.881	22	16	85	131.9	*******
28	S10_48154038	17.657	18	57	76	53.6	*******
29	S10_51537075	18.488	23	24	65	68.1	*******
30	S10_48074234	19.403	28	35	76	67.4	*******
31	S10_50508494	20.076	27	23	66	68.5	*******
32	S10_50725358	20.887	23	18	75	101.8	*******
33	S10_50365958	21.352	25	13	72	104.3	*******
34	S10_50365959	21.44	25	14	71	99.6	*******
35	S10_49884947	22.034	27	19	70	84.3	*******
36	S10_51006848	22.76	21	19	64	77.4	*******
37	S10_IS10263_49925321	23.029	21	51	79	60.5	*******
38	S10_49100577	23.962	24	26	76	86.4	*******
39	S10_49536872	24.664	23	9	51	69.8	*******
40	S10_48208212	24.963	28	14	64	81.8	*******
41	S10_51345335	25.88	30	33	65	49.2	*******
42	S10_51501680	26.918	28	17	62	71.4	*******
43	S10_52322143	27.524	24	18	62	72.2	*******
44	S10_52972082	27.785	32	19	69	78.8	*******

			Alleles %				
S.No.	Locus 3.1v	Position (cM)	RSG04008-6	Heterozygote	J2614-11	χ^2	Signif.
45	S10_51497655	28.41	26	20	83	111.8	*******
46	S10_52316579	29.023	24	8	78	133.3	*******
47	S10_52973075	29.365	15	3	51	95.1	*******
48	S10_52972135	29.715	23	4	48	76.5	*******
49	S10_53556850	30.175	33	9	61	85.4	*******
50	S10_51245140	30.462	36	12	67	88.7	*******
51	S10_52199633	30.959	30	10	57	76.2	*******
52	S10_53148305	31.827	37	20	75	86	*******
53	S10_53077439	32.291	23	20	84	118.2	*******
54	S10_53080452	32.558	22	43	84	78.2	*******
55	S10_46358525	33.08	23	16	63	79.4	*******
56	S10_50394264	33.837	26	19	70	85.2	*******
57	S10_55164775	33.902	7	1	74	187.5	*******
58	S10_55164753	33.948	7	1	75	190.5	*******
59	S10_51163559	34.686	21	2	45	77.2	*******
60	S10_53973163	34.972	29	19	65	72.7	*******
61	S10_50413373	35.288	9	6	56	111.3	*******
62	S10_53454779	35.678	16	17	102	185.1	*******
63	S10_54118313	35.758	20	3	43	70.6	*******
64	S10_52971581	35.997	18	3	48	83.6	*******
65	S10_53353127	36.145	26	5	42	61.4	*******
66	S10_54575547	36.401	15	11	90	173.2	*******
67	S10_45765686	36.843	21	4	44	69.3	*******
68	S10_52969717	37.229	32	22	66	67.4	*******
69	S10_49621607	37.73	15	6	53	91	*******
70	S10_54005372	38.079	22	5	48	74.4	*******
71	S10_54491305	38.342	20	7	49	72.7	*******
72	S10_53108642	38.346	14	2	49	94.9	*******
73	S10_54139341	38.66	28	33	84	86.3	*******
74	S10_50505399	39.38	18	7	50	76.9	*******
75	S10_50505397	39.38	18	7	50	76.9	*******
76	S10_53839087	39.394	14	4	63	125.1	*******
77	S10_52995995	39.782	24	9	60	88.3	*******
78	S10_53235767	40.377	24	5	44	65.3	*******
79	S10_55181668	40.677	20	2	43	73.5	*******
80	S10_53870950	40.76	24	7	55	82.6	*******
81	S10_54388058	41.371	28	13	59	74	*******
82	S10_Xgap001_54813392	42.833	32	44	74	49.1	*******
83	S10_54838977	43.958	41	16	70	84.3	*******
84	S10_54891180	44.416	30	10	59	80	*******
85	S10_54841288	44.945	30	14	65	82.7	*******
86	S10_54491782	45.857	30	30	81	83.4	*******
87	S10_54491775	45.985	30	30	80	81.4	*******
88	S10_54491422	46.461	23	3	53	90.2	*******
89	S10_55270382	47.424	32	19	76	92.9	*******
90	S10_55278625	48.021	17	7	52	82.8	*******

S.No.	Locus 3.1v	Position (cM)	Alleles % RSG04008-6	Heterozygote	J2614-11	χ^2	Signif.
91	S10_54553482	48.532	29	5	52	79.5	*******
92	S10_54841484	49.165	28	7	46	63.4	*******
93	S10_53972403	49.555	15	5	62	117.1	*******
94	S10_54834121	49.678	27	5	58	92.5	*******
95	S10_54841727	50.491	45	18	58	62.5	*******
96	S10_54841789	51.036	34	24	71	72.1	*******
97	S10_55355408	51.386	31	14	66	84.1	*******
98	S10_54890508	52.221	33	14	64	79.4	*******
99	S10_55181794	53.005	19	5	47	74.5	*******
100	S10_55320722	53.54	34	27	72	68.6	*******
101	S10_54529963	54.416	29	9	60	84.9	*******
102	S10_54491644	55.076	14	6	53	92.6	*******
103	S10_54491183	55.529	24	4	41	62.3	*******
104	S10_54491653	55.779	16	6	56	96.9	*******
105	S10_55375263	56.465	33	9	60	83.5	*******
106	S10_54938287	57.098	30	3	39	62.8	*******
107	S10_54493420	57.606	27	10	55	73.4	*******
108	S10_54493421	57.743	27	9	53	71.8	*******
109	S10_55586987	58.521	34	9	66	94.8	*******
110	S10_55674363	60.56	36	24	78	84.3	*******
111	S10_55691249	62.051	28	3	41	65.2	*******
112	S10_55481308	62.658	30	0	42	76	*******
113	S10_55998859	63.545	35	4	52	82	*******
114	S10_55904321	64.305	43	15	59	69.1	*******
115	S10_55905116	65.957	44	25	73	71.5	*******
116	S10_55680523	66.472	40	8	32	52.8	*******
117	S10_56417482	67.237	24	8	40	50.7	*******
118	S10_55973848	68.043	38	8	48	66.8	*******
119	S10_55953518	69.328	45	8	38	62.9	*******
120	S10_56414677	70.055	37	8	44	61	*******
121	S10_55999093	70.634	30	3	40	64.2	*******
122	S10_55778193	71.482	42	9	44	62.5	*******
123	S10_56302132	72.248	40	15	62	73	*******
124	S10_56511507	73.641	38	8	56	78.9	*******
125	S10_56608572	74.202	32	19	62	65.7	*******
126	S10_56352583	75.152	51	13	55	73	*******
127	S10_56653607	76.646	55	20	67	75.3	*******
128	S10_56641589	77.204	41	8	42	61.8	*******
129	S10_56508509	77.614	35	1	45	79.5	*******
130	S10_56466518	78.719	40	7	45	66.7	*******
131	S10_56466497	78.725	41	7	45	67.5	*******
132	S10_56641518	79.367	35	6	37	56	*******
133	S10_56749818	80.008	39	11	55	70.5	*******
134	S10_56476051	80.752	26	8	52	72.7	*******
135	S10_56475108	81.702	45	15	52	60.9	*******
136	S10_56464599	82.18	52	12	58	79.3	*******

			Alleles %				
S.No.	Locus 3.1v	Position (cM)	RSG04008-6	Heterozygote	J2614-11	χ^2	Signif.
137	S10_56692703	82.729	35	4	39	63.2	*******
138	S10_56989399	83.885	53	33	58	42.6	*******
139	S10_56989401	84.793	49	17	51	59	*******
140	S10_56989405	84.947	51	17	51	60.7	*******
141	S10_56918503	86.428	55	14	59	78.4	*******
142	S10_57093649	86.987	31	2	44	73.6	*******
143	S10_56508615	87.779	37	4	29	56.7	*******
144	S10_56299271	88.617	39	7	32	53.8	*******
145	S10_56854454	89.482	43	5	34	65.2	*******
146	S10_57663572	90.684	18	4	77	154	*******
147	S10_57600325	91.295	45	7	51	77.6	*******
148	S10_57590596	92.115	64	17	64	85	*******
149	S10_57404251	93.122	59	31	62	53.4	*******
150	S10_57809613	94.409	42	2	37	73.8	*******
151	S10_57590618	94.987	61	4	48	100.6	*******
152	S10_57590703	95.618	59	5	49	95.7	*******
153	S10_57381554	95.877	37	1	39	73.2	*******
154	S10_57347175	96.055	37	3	35	63.6	*******
155	S10_57804193	96.424	42	6	32	60.3	*******
156	S10_57804222	96.622	43	6	31	61.4	*******
157	S10_57660753	97.265	70	17	62	89.6	*******
158	S10_58704603	97.876	15	2	63	129.8	*******
159	S10_57809876	98.396	35	2	33	62.3	*******
160	S10_57806877	98.701	58	6	51	93.1	*******
161	S10_57507647	99.044	50	2	46	90.5	*******
162	S10_57692900	99.608	65	21	56	71.6	*******
163	S10_57714076	100.398	71	15	55	91	*******
164	S10_57783305	101.244	62	8	57	97.4	*******
165	S10_58908285	102.44	39	2	21	64.7	*******
166	S10_58610366	103.078	55	8	49	82.9	*******
167	S10_58276305	103.623	47	5	36	71.9	*******
168	S10_58596541	104.412	46	3	35	75.3	*******
169	S10_58565687	104.802	42	3	42	75.4	*******
170	S10_Xtxp141_484999266	105.596	71	19	62	86.6	*******
171	S10_Xiabt_58564309	105.94	69	17	49	81.5	*******
172	S10_58758633	106.676	55	2	28	94.3	*******
173	S10_59265767	107.172	63	6	30	98.5	*******
174	S10_59104911	107.396	35	0	41	77	*******
175	S10_58728876	107.796	63	11	39	83.5	*******
176	S10_58610981	108.032	28	2	31	53.6	*******
177	S10_58758082	108.357	57	0	38	102.6	*******
178	S10_58950644	108.615	62	7	33	92.4	*******
179	S10_60117639	109.008	50	4	32	78.3	*******
180	S10_59422845	109.224	40	4	42	70.8	*******
181	S10_59113364	109.466	58	6	41	87.9	*******
182	S10_58694071	109.749	40	2	27	66.1	*******

			Alleles %				
S.No.	Locus 3.1v	Position (cM)	RSG04008-6	Heterozygote	J2614-11	χ^2	Signif.
183	S10_58920175	109.901	49	2	20	86.9	*******
184	S10_59593663	110.125	42	6	38	64	*******
185	S10_59298151	110.337	50	10	46	70.1	*******
186	S10_59342868	110.528	41	3	30	65.8	*******
187	S10_58634498	110.886	46	3	30	73.9	*******
188	S10_60117590	111.191	46	1	33	80.3	*******
189	S10_59113218	111.359	72	13	49	94.9	*******
190	S10_59294116	111.735	49	3	35	79.9	*******
191	S10_59294324	112.082	75	19	54	87.7	*******
192	S10_59336236	112.3	69	14	49	88	*******
193	S10_59837691	112.633	32	5	43	64.3	*******
194	S10_59498752	113.022	57	9	45	80.5	*******
195	S10_59498757	113.057	57	10	44	77.7	*******
196	S10_58933428	113.284	57	6	41	86.3	*******
197	S10_58901865	113.523	50	6	37	74.2	*******
198	S10_59697086	113.698	72	2	35	126.3	*******
199	S10_59831050	113.992	66	8	43	96.2	*******
200	S10_59482408	114.325	69	11	46	94.2	*******
201	S10_59620312	114.593	39	5	36	61.5	*******
202	S10_59848048	114.809	69	13	48	90	*******
203	S10_59491003	115.101	38	8	29	48.6	*******
204	S10_59620328	115.321	69	11	46	94.2	*******
205	S10_59850129	115.589	70	7	46	106	*******
206	S10_59850128	115.632	69	7	47	104.5	*******
207	S10_59849054	116.207	59	8	37	83.8	*******
208	S10_59849056	116.207	59	8	37	83.8	*******
209	S10_60091545	116.518	68	7	43	102.3	*******
210	S10_59696189	116.702	46	6	33	66.7	*******
211	S10_59799283	116.941	61	4	43	98.6	*******
212	S10_59974908	117.319	58	4	19	103.3	*******
213	S10_59749988	117.456	77	17	44	94.2	*******
214	S10_59892311	117.745	61	10	34	82.7	*******
215	S10_59690880	117.994	39	0	23	70.3	*******
216	S10_60106142	118.156	51	7	42	75.6	*******
217	S10_59854935	118.271	63	6	40	96	*******
218	S10_60120147	118.575	52	6	34	76.6	*******
219	S10_60058846	118.728	49	4	29	76.5	*******
220	S10_60091616	119.005	71	15	36	89.5	*******
221	S10_60150914	119.287	38	5	22	54.4	*******
222	S10_59892977	119.541	64	18	40	70.1	*******
223	S10_60110925	119.868	42	5	33	63.3	*******
224	S10_60513711	120.044	51	9	30	67.4	*******
225	S10_59844168	120.325	70	11	39	96	*******
226	S10_60059042	120.618	36	3	29	58	*******
227	S10_61153461	120.867	62	13	41	77.4	*******
228	S10_61153460	121.02	63	12	41	81.3	*******

			Alleles %				
S.No.	Locus 3.1v	Position (cM)	RSG04008-6	Heterozygote	J2614-11	χ^2	Signif.
229	S10_61153456	121.034	63	13	41	79	*******
230	S10_60613735	121.363	33	3	20	50.7	*******
231	S10_60173717	121.68	68	15	40	83.1	*******
232	S10_60135251	121.911	38	3	32	62.5	*******
233	S10_60551643	122.169	38	4	31	59.2	*******
234	S10_60593638	122.46	73	23	40	75.6	*******
235	S10_60231331	122.798	80	3	9	190	*******
236	S10_60593601	122.951	62	14	32	75.9	*******
237	S10_60308400	123.601	78	30	41	71.5	*******
238	S10_60602919	124.099	42	7	27	56.5	*******
239	S10_60695074	124.349	77	31	38	69.2	*******
240	S10_61190725	124.648	73	29	46	64.6	*******
241	S10_60468748	124.892	48	6	29	69.4	*******
242	S10_60468746	124.978	47	6	29	67.7	*******
243	S10_60902159	125.224	36	6	35	54.9	*******
244	S10_60513644	125.463	41	8	36	56.6	*******
245	S10_60577527	125.703	60	13	46	76	*******
246	S10_60922122	126.247	36	4	30	55.9	*******
247	S10_60572000	126.61	71	7	35	109.7	*******
248	S10_60593652	127.043	63	10	33	86.8	*******
249	S10_61027454	127.635	69	16	43	82.6	*******
250	S10_60566724	128.102	73	13	42	96.3	*******
251	S10_60649673	128.373	65	11	43	87.2	*******
252	S10_60612267	128.942	77	12	46	105.5	*******
253	S10_60557352	129.512	49	7	46	76.1	*******
254	S10_60619195	129.798	51	3	34	83	*******
255	S10_60333222	130.063	39	3	35	65.9	*******
256	S10_60770004	130.34	58	9	38	79.7	*******
257	S10_60231203	130.887	56	13	47	71.2	*******
258	S10_60231220	131.167	62	7	39	91.6	*******
259	S10_60973291	131.9	16	0	16	32	*******
260	S10_Xisep1011_61017707	132.224	71	34	42	53.9	*******
261	S10_60214866	132.579	45	6	24	64.7	*******
262	S10_60518102	133.343	38	5	25	54.4	*******
263	S10_60623608	133.711	75	0	3	210.9	*******
264	S10_60872509	133.934	41	4	37	67.2	*******
265	S10_60654069	135.692	5	0	42	105.3	*******

*** highly significantly deviated from 1:2:1 F2 segregation ratio.
